# Drug screening identifies tazarotene and bexarotene as therapeutic agents in multiple sulfatase deficiency

**DOI:** 10.15252/emmm.202114837

**Published:** 2023-02-15

**Authors:** Lars Schlotawa, Karolina Tyka, Matthias Kettwig, Rebecca C Ahrens‐Nicklas, Matthias Baud, Tea Berulava, Nicola Brunetti‐Pierri, Alyssa Gagne, Zackary M Herbst, Jean A Maguire, Jlenia Monfregola, Tonatiuh Pena, Karthikeyan Radhakrishnan, Sophie Schröder, Elisa A Waxman, Andrea Ballabio, Thomas Dierks, André Fischer, Deborah L French, Michael H Gelb, Jutta Gärtner

**Affiliations:** ^1^ Department of Paediatrics and Adolescent Medicine University Medical Centre Göttingen Göttingen Germany; ^2^ Division of Human Genetics and Metabolism The Children's Hospital of Philadelphia Philadelphia PA USA; ^3^ School of Chemistry and Institute for Life Sciences University of Southampton Southampton UK; ^4^ Department for Epigenetics and Systems Medicine in Neurodegenerative Diseases German Centre for Neurodegenerative Diseases Göttingen Germany; ^5^ Telethon Institute of Genetics and Medicine Pozzuoli Italy; ^6^ Department of Translational Medicine University of Naples Federico II Naples Italy; ^7^ Center for Cellular and Molecular Therapeutics The Children's Hospital of Philadelphia Philadelphia PA USA; ^8^ Department of Pathology and Laboratory Medicine The Children's Hospital of Philadelphia Philadelphia PA USA; ^9^ Department of Chemistry University of Washington Seattle WA USA; ^10^ Bioinformatics Unit German Centre for Neurodegenerative Diseases Göttingen Germany; ^11^ Faculty of Chemistry, Biochemistry I Bielefeld University Bielefeld Germany; ^12^ Department of Molecular and Human Genetics and Neurological Research Institute Baylor College of Medicine Houston TX USA; ^13^ Department of Psychiatry and Psychotherapy University Medical Center Göttingen Göttingen Germany; ^14^ Multiscale Bioimaging Cluster of Excellence, University Medical Center Göttingen University of Göttingen Göttingen Germany

**Keywords:** drug screening, formylglycine‐generating enzyme, lysosomal disorder, retinoids, sulfatase‐modifying factor 1, Genetics, Gene Therapy & Genetic Disease, Neuroscience

## Abstract

Multiple sulfatase deficiency (MSD, MIM #272200) results from pathogenic variants in the SUMF1 gene that impair proper function of the formylglycine‐generating enzyme (FGE). FGE is essential for the posttranslational activation of cellular sulfatases. MSD patients display reduced or absent sulfatase activities and, as a result, clinical signs of single sulfatase disorders in a unique combination. Up to date therapeutic options for MSD are limited and mostly palliative. We performed a screen of FDA‐approved drugs using immortalized MSD patient fibroblasts. Recovery of arylsulfatase A activity served as the primary readout. Subsequent analysis confirmed that treatment of primary MSD fibroblasts with tazarotene and bexarotene, two retinoids, led to a correction of MSD pathophysiology. Upon treatment, sulfatase activities increased in a dose‐ and time‐dependent manner, reduced glycosaminoglycan content decreased and lysosomal position and size normalized. Treatment of MSD patient derived induced pluripotent stem cells (iPSC) differentiated into neuronal progenitor cells (NPC) resulted in a positive treatment response. Tazarotene and bexarotene act to ultimately increase the stability of FGE variants. The results lay the basis for future research on the development of a first therapeutic option for MSD patients.

## Graphical abstract

The ultra‐rare lysosomal disease Multiple Sulfatase Deficiency (MSD) is a progressive currently incurable neurodegenerative disorder. Here we demonstrate that retinoids tazarotene and bexarotene, identified by a high‐throughput drug screening, are active therapeutic agents for MSD *in‐vitro*.

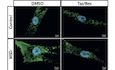

**The paper explained**

**Problem**

No curative therapy exists for Multiple Sulfatase Deficiency (MSD), an ultra‐rare lysosomal disorder. MSD is caused by a defect of the posttranslational activation of all cellular sulfatases through the formylglycine‐generating enzyme (FGE) in the endoplasmic reticulum. FGE is encoded by the *SUMF1* gene and *SUMF1* mutations in MSD patients lead to impaired function of FGE and reduced or absent sulfatase activities in every cell. MSD patients present with a combination of signs and symptoms of single sulfatase deficiencies like developmental delay, neurodegeneration, skeletal abnormalities, and ichthyosis among others in a progressive, very severe disease.

**Results**

We developed a high‐throughput screening assay using MSD patient‐derived fibroblasts and investigated the rescue of the enzymatic function of one defective sulfatase, arylsulfatase A (ARSA), as primary readout. Applying the assay we screened a library of 785 licensed drugs and detected two retinoids, tazarotene and bexarotene to increase ARSA activity. In subsequent analysis, both drugs proved to be effective in reversing cellular pathology in MSD fibroblasts and neuronal progenitor cells. Both drugs work via the stabilization of misfolded FGE proteins to increase sulfatase activities in MSD cells.

**Impact**

Our study reveals a new mechanism of retinoids in MSD pathology. Furthermore, we identified the first described agents to correct MSD pathology *in vitro*. Our data lay the basis for future research on therapeutic approaches for MSD and the identification of targets that mediate retinoid treatment response.

## Introduction

Multiple sulfatase deficiency (MSD, MIM #272200) is an ultra‐rare lysosomal disorder caused by pathogenic variants in the *SUMF1* gene encoding the formylglycine‐generating enzyme (FGE; Cosma *et al*, 2003; Dierks *et al*, 2003). FGE is localized in the endoplasmic reticulum (ER) and is required for the activation of all newly synthesized sulfatases. FGE oxidizes a conserved cysteine in the active site of every sulfatase to formylglycine, which is required for catalytic activity (Dierks *et al*, 2005). Sulfatases are a group of 17 enzymes in humans necessary for the catalytic breakdown of sulfated substrates. The majority are localized in lysosomes, while others are found in the ER, Golgi, and on the cell surface (Diez‐Roux & Ballabio, 2005). Most *SUMF1* pathogenic variants are single amino acid substitutions that lead to FGE protein misfolding (Schlotawa *et al*, 2011, 2020). Improperly folded FGE protein retains some residual activity, leading to some degree of downstream residual sulfatase activities (Schlotawa *et al*, 2011). Misfolded FGE variants interact with protein disulfide isomerase (PDI) in the ER where PDI targets non‐natural disulfide bridges formed in FGE because of misfolding and determines early degradation of FGE variants. Knockdown or pharmacological inhibition of PDI partially rescues sulfatase activities in MSD patient‐derived cells (Schlotawa *et al*, 2018).

The combined and variable deficiency of all cellular sulfatases leads to a complex clinical presentation in MSD patients with signs of single sulfatase deficiencies like metachromatic leukodystrophy (MLD), several mucopolysaccharidosis subtypes (MPS II, IIIa, IIId, IVa, VI), X‐linked recessive chondrodysplasia punctata type 1 (CDPX1), and X‐linked ichthyosis (XLI) (Adang *et al*, 2020; Cappuccio *et al*, 2020; Schlotawa *et al*, 2020; Verheyen *et al*, 2021). MSD is an early‐onset progressive disease. Natural disease history data reveal that the mean survival of MSD patients is 13 years. Currently, more than 50 patients are known worldwide and nearly 150 MSD cases have been described in the literature since its first characterization (Adang *et al*, 2020; Cappuccio *et al*, 2020; Schlotawa *et al*, 2020). MSD disease severity correlates with *SUMF1* mutation severity: unstable FGE variants with extremely reduced activity cause severe forms of MSD, whereas variants with higher residual activity and stability result in attenuated phenotypes (Adang *et al*, 2020; Schlotawa *et al*, 2020). The most severe cases were found to entirely lack FGE function (Busche *et al*, 2009; Schlotawa *et al*, 2019).

There is currently no disease‐modifying therapy for MSD and the only treatment options are symptomatic and palliative (Ahrens‐Nicklas *et al*, 2018). One potential drug development strategy in ultra‐rare disorders such as MSD is drug repurposing, alternatively called repositioning. Licensed drugs are screened for their potential as a treatment for different diseases beyond their original indication. Because safety and efficacy data have been obtained in previous studies and do not necessarily need to be generated again, drug repurposing is time‐effective but also cost‐effective compared with the development of new drugs. This is especially attractive for treating rare diseases with comparatively low commercial interest and devastating diseases without any existing therapy (Strittmatter, 2014; Pushpakom *et al*, 2019).

In this study, we present the results from a high‐throughput phenotypic screen of 785 FDA‐approved drugs on MSD patient cells, resulting in the discovery of two structurally and mechanistically related retinoid drugs reversing the cellular MSD phenotype.

## Results

### A screen of 785 FDA‐approved drugs reveals 13 hits that increase arylsulfatase A activity in MSD patient cells

In preparation for our drug screen, we adopted a method developed by Geng *et al* based on a diagnostic routine arylsulfatase A (ARSA) activity assay for use in 96‐well plates with lysates of MSD patient‐derived cells (see Appendix Supplementary Methods for details and Fig S1A; Baum *et al*, 1959; Geng *et al*, 2011). This spectrophotometric assay detects changes in optical density (OD) at 515 nm when a sulfate from the synthetic substrate p‐nitrocatechol sulfate (pNCS) is enzymatically cleaved off by ARSA, resulting in the generation of the colored p‐nitrocatechol (pNC) product as a quantifiable readout. In this assay, an increase in OD indicates higher pNC levels, correlating with an increased ARSA activity (Baum *et al*, 1959). We used an immortalized MSD patient‐derived primary fibroblast line (MSDi) with a homozygous *SUMF1* missense mutation (c.463C > T, p.Ser155Pro; Cosma *et al*, 2003).

Mean baseline OD of all dimethyl sulfoxide (DMSO) treated controls was 0.03 (standard deviation (SD) 0.004, median 0.03, minimum 0.01, maximum 0.04, *n* = 108, Appendix Fig S1B). MSDi cells stably expressing FGEHis wild‐type protein thereby rescuing ARSA activity served as a positive control and were used to determine the upper OD limit (mean 0.16, SD 0.04, median 0.15, minimum 0.09, maximum 0.25, *n* = 28).

**Figure 1:**
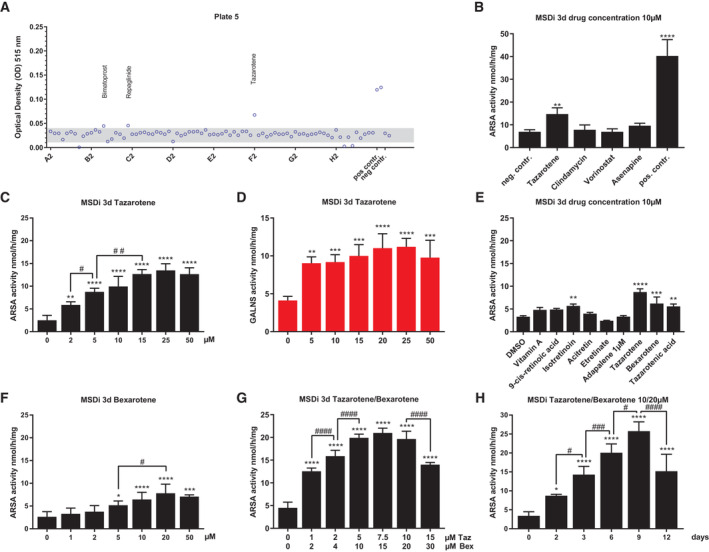
Drug screen and evaluation of positive hit drugs using immortalized MSD patient‐derived cells Indicative plot of one 96‐well screening plate of the MSD‐ARSA high‐throughput screening assay with negative and positive controls included. Individual OD values were given for each well (circles) and indicate ARSA activity (gray area: baseline activity). Three hit drugs exceeded the upper baseline OD range. *N* = 1 experiment per well and drug treatment, final concentration of each drug 10 μM, treatment time 48 h.ARSA activity quantification (nmol/h/mg) after treatment of MSDi cells with a selection of four positive hit drugs at a final concentration of 10 μM on MSDi cells in 25 cm^2^ cell culture flasks for 3 days. Data represent mean ± SD of seven independent experiments (biological replicates). One‐way ANOVA followed by the Tukey's test for multiple comparisons. Difference against negative control: **P < 0.01, ****P < 0.0001. See details on *P*‐values in Appendix Table S3.ARSA activity quantification (nmol/h/mg) after treatment of MSDi cells with increasing concentrations of tazarotene for 3 days. Data represent mean ± SD of 3–9 independent experiments (biological replicates). One‐way ANOVA followed by the Tukey's test for multiple comparisons. Displayed are significance levels for the next significant difference between adjacent concentrations. # *P* < 0.05, ## *P* < 0.01. Difference against 0 μM control: **P < 0.01, ****P < 0.0001. See details on *P*‐values in Appendix Table S4.GALNS activity quantification after treatment of MSDi cells with increasing concentrations of tazarotene for 3 days. Data represent mean ± SD of four independent experiments (biological replicates). One‐way ANOVA followed by the Tukey's test for multiple comparisons. Difference against 0 μM control: **P < 0.01, ***P < 0.001, ****P < 0.0001. See details on *P*‐values in Appendix Table S5.Analysis of different retinoids' potential to restore ARSA activity in MSDi cells in comparison to tazarotene after treatment for 3 days at a final concentration 10 μM of each drug (Adapalene 1 μM). Data represent mean ± SD of three independent experiments (biological replicates). One‐way ANOVA followed by the Tukey's test for multiple comparisons. Difference against DMSO control: **P < 0.01, ***P < 0.001, ****P < 0.0001. See details on *P*‐values in Appendix Table S6.ARSA activity quantification after treatment of MSDi cells with increasing concentrations of bexarotene for 3 days. Data represent mean ± SD of 3–10 independent experiments (biological replicates). One‐way ANOVA followed by the Tukey's test for multiple comparisons. Displayed are significance levels for the next significant difference between adjacent concentrations. # *P* < 0.05. Difference against 0 μM control: *P < 0.05, ***P < 0.001, ****P < 0.0001. See details on *P*‐values in Appendix Table S7.ARSA activity quantification after simultaneous treatment of MSDi cells with increasing concentrations of tazarotene and bexarotene in a fixed combination of 1:2 for 3 days. Data represent mean ± SD of 3–6 independent experiments (biological replicates). One‐way ANOVA followed by the Tukey's test for multiple comparisons. Displayed are significance levels for the next significant difference between adjacent concentrations. #### *P* < 0.0001. Difference against 0/0 μM control: ****P < 0.0001. See details on *P*‐values in Appendix Table S8.Analysis and quantification of a time‐dependent increase in ARSA activity in MSDi cells simultaneously treated with 10 and 20 μM tazarotene and bexarotene, respectively. Data represent mean ± SD of 3–10 independent experiments (biological replicates). One‐way ANOVA followed by the Tukey's test for multiple comparisons. Displayed are significance levels for the next significant difference between treatment times. # *P* < 0.05, ### *P* < 0.001, #### *P* < 0.0001. Difference against 0 days control: *P < 0.05, ****P < 0.0001. See details on *P*‐values in Appendix Table S9. Source data are available online for this figure.

We performed a primary screen using an FDA‐approved drug library that was gifted by LifeArc, London, UK, containing 785 licensed drugs (1 mM stocks in DMSO, see details in the experimental section) at a final concentration of 10 μM (1% DMSO content). We identified 13 drugs that exceeded the upper limit of baseline OD values in lysates of treated cells (Fig 1A, Appendix Figs S1C and D, S2, and S3).

Drugs that resulted in ODs below baseline impaired cell viability and were screened again at a final concentration of 1 and 0.1 μM but revealed no further hits (Appendix Fig S4A and B). All hit drugs were counterscreened under standard assay conditions, devoid of cells to detect any interference with the ARSA assay and artificial OD increase, but no such false positive hits were detected (Appendix Fig S4C). We chose tazarotene, clindamycin, vorinostat, and asenapine as the first selection of hit drugs to be included in follow‐up experiments.

### Tazarotene and bexarotene effectively increase the activity of lysosomal sulfatases in immortalized MSD patient cells

**Figure EV1:**
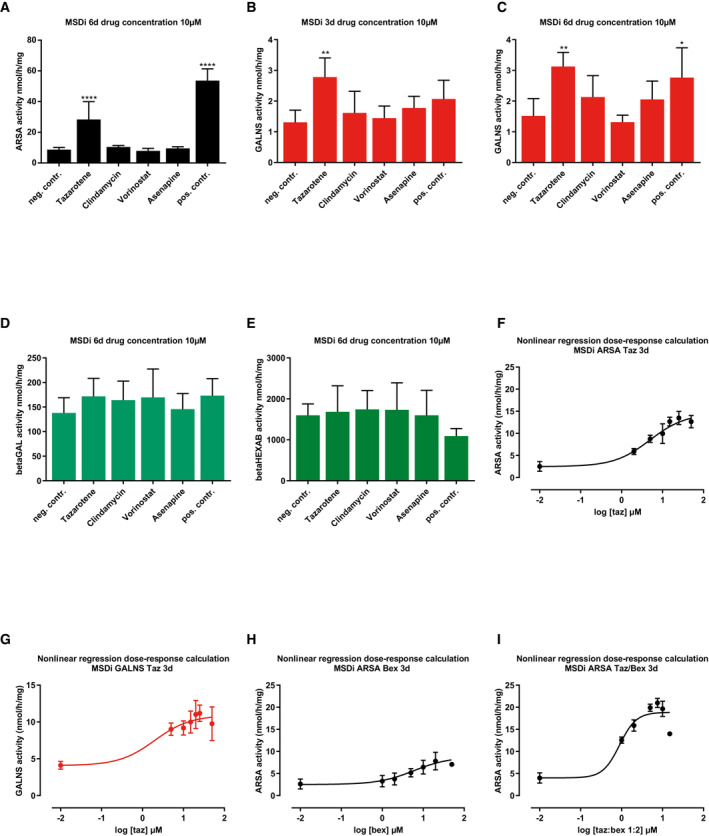
Hit drug evaluation on immortalized MSD fibroblasts ARSA activity quantification (nmol/h/mg) after treatment of MSDi cells in 25 cm^2^ cell culture flasks with a selection of four positive hit drugs at a final concentration of 10 μM for 6 days. Data represent mean ± SD of seven independent experiments (biological replicates). One‐way ANOVA followed by Tukey's test for multiple comparisons. Difference against negative control: ****P < 0.0001. See details on *P*‐values in Appendix Table S31.GALNS activity quantification (nmol/h/mg) after treatment of MSDi cells with a selection of four positive hit drugs at a final concentration of 10 μM for 3 days. Data represent mean ± SD of five independent experiments (biological replicates). One‐way ANOVA followed by Tukey's test for multiple comparisons. Difference against negative control: **P < 0.001. See details on *P*‐values in Appendix Table S32.GALNS activity quantification (nmol/h/mg) after treatment of MSDi cells with a selection of four positive hit drugs at a final concentration of 10 μM for 6 days. Data represent mean ± SD of five independent experiments (biological replicates). One‐way ANOVA followed by Tukey's test for multiple comparisons. Difference against negative control: * *P* < 0.05, ** *P* < 0.001. See details on *P*‐values in Appendix Table S33.β‐galactosidase (betaGAL) activity quantification (nmol/h/mg) after treatment of MSDi cells with a selection of four positive hit drugs at a final concentration of 10 μM for 3 days. Data represent mean ± SD of five independent experiments (biological replicates). One‐way ANOVA followed by Tukey's test for multiple comparisons.ß‐hexosaminidase A and B (betaHEXAB) activity quantification (nmol/h/mg) after treatment of MSDi cells with a selection of four positive hit drugs at a final concentration of 10 μM for 3 days. Data represent mean ± SD of five independent experiments (biological replicates). One‐way ANOVA followed by Tukey's test for multiple comparisons.Dose‐response curve of ARSA activity calculated from data displayed in Fig 1C (MSDi cells, tazarotene treatment) by nonlinear regression analysis. Drug concentrations are displayed after transformation into log10 values and baseline activity (negative control, DMSO‐only treatment) was manually referred to log‐2. Dots and error bars represent mean ± SD.Dose‐response curve of GALNS activity calculated from data displayed in Fig 1D (MSDi cells, tazarotene treatment) by nonlinear regression analysis. Drug concentrations are displayed after transformation into log10 values and baseline activity (negative control, DMSO‐only treatment) was manually referred to log‐2. Dots and error bars represent mean ± SD.Dose‐response curve of ARSA activity calculated from data displayed in Fig 1F (MSDi cells, bexarotene treatment) by nonlinear regression analysis. Drug concentrations are displayed after transformation into log10 values and baseline activity (negative control, DMSO‐only treatment) was manually referred to log‐2. Dots and error bars represent mean ± SD.Dose‐response curve of ARSA activity calculated from data displayed in Fig 1G (MSDi cells, tazarotene/bexarotene treatment) by nonlinear regression analysis. Drug concentrations are displayed after transformation into log10 values and baseline activity (negative control, DMSO‐only treatment) was manually referred to log‐2. Dots and error bars represent mean ± SD. Source data are available online for this figure.

To analyze whether the treatment response was reproducible outside of the 96‐well format, MSDi cells were treated with fresh stocks of commercially available selected hit drugs at a final concentration of 10 μM of each for 3 and 6 days in cell culture flasks. Samples were analyzed by standard diagnostic lysosomal enzyme activity assays in cell lysates (see Materials and Methods for details). Only tazarotene showed a significant increase in ARSA and N‐acetylgalactosamine‐6‐sulfatase (GALNS) activity assays (Figs 1B and EV1A–C). Activities of nonsulfatase lysosomal hydrolases β‐hexosaminidase A and B (betaHEXAB) and β‐galactosidase (betaGAL) did not significantly change upon drug treatment compared with DMSO‐only treated controls (Fig EV1D and E). MSDi cells, treated for 3 days with different tazarotene concentrations, displayed a dose‐dependent significant increase in ARSA activities as compared to baseline. Increased activity was noted at a drug concentration as low as 2 μM for ARSA and 5 μM for GALNS (ARSA activity: EC50 4.9 μM; GALNS activity: EC50 2 μM, Figs 1C and D, and EV1F and G).

Tazarotene belongs to the 3^rd^ generation of retinoids, compounds synthesized from vitamin A (Khalil *et al*, 2017). To analyze the potential of other retinoids to increase ARSA activity in MSDi cells, we used compounds of every retinoid generation applying the same treatment conditions. In addition, tazarotenic acid, the biologically active form of tazarotene after first‐pass metabolism in organisms (Tang‐Liu *et al*, 1999), was included. Besides tazarotenic acid, 3^rd^ generation retinoid bexarotene and 2^nd^ generation retinoid isotretinoin led to a significant ARSA activity increase as compared to the control treatment. Other retinoids failed to increase ARSA activity significantly (Fig 1E). Based on these results, we next tested bexarotene for a dose‐dependent effect on ARSA activity in MSDi cells and detected a significant increase (EC50 5.9 μM, Figs 1F and EV1H). Tazarotene and bexarotene are well‐known cell active agonists of retinoic acid receptors (RAR) and retinoid X receptors (RXR), respectively (Miller *et al*, 1997; Hofmann *et al*, 1999). We investigated the effect of treatment on ARSA activity with a fixed combination of tazarotene and bexarotene at a ratio of 1:2, chosen based on doses that previously increased ARSA activity when given individually. We observed a dose‐dependent, significant increase in ARSA activity starting at concentrations as low as 1 μM tazarotene and 2 μM bexarotene. (ARSA activity: EC50 0.9/1.8 μM tazarotene/bexarotene, Figs 1G and EV1I). Finally, we used 10/20 μM tazarotene/bexarotene for analyzing a time‐dependent response of ARSA activity. ARSA activity went up 7.6‐fold to a maximum of 25.8 nmol/h/mg (SD 2.3) after 9 days of treatment (Fig 1H).

### Tazarotene and bexarotene increase sulfatase activities and reduce LAMP1 staining and GAG storage in primary MSD patient cells

**Figure 2:**
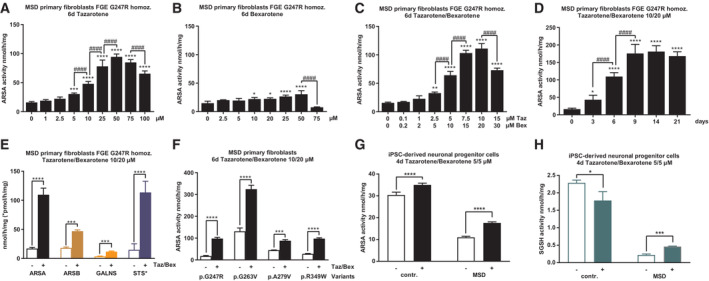
Tazarotene and bexarotene increase sulfatase activities in MSD primary fibroblasts and MSD iPSC‐derived NPCs ARSA activity quantification after treatment of MSD primary fibroblasts (variant FGE Gly247Arg homozygous) with increasing concentrations of tazarotene for 6 days. Data represent mean ± SD of 3–7 independent experiments (biological replicates). One‐way ANOVA followed by the Tukey's test for multiple comparisons. Displayed are significance levels for the next significant difference between adjacent concentrations. #### *P* < 0.0001. Difference against 0 μM control: ***P < 0.001, ****P < 0.0001. See details on *P*‐values in Appendix Table S10.ARSA activity quantification after treatment of MSD primary fibroblasts (variant FGE Gly247Arg homozygous) with increasing concentrations of bexarotene for 6 days. Data represent mean ± SD of 3–9 independent experiments (biological replicates). One‐way ANOVA followed by the Tukey's test for multiple comparisons. Displayed are significance levels for the next significant difference between adjacent concentrations. #### *P* < 0.0001. Difference against 0 μM control: *P < 0.05, ****P < 0.0001. See details on *P*‐values in Appendix Table S11.ARSA activity quantification after simultaneous treatment of MSD primary fibroblasts (variant FGE Gly247Arg homozygous) with increasing concentrations of tazarotene and bexarotene in a fixed combination of 1:2 for 6 days. Data represent mean ± SD of 3–6 independent experiments (biological replicates). One‐way ANOVA followed by the Tukey's test for multiple comparisons. Displayed are significance levels for the next significant difference between adjacent concentrations. #### *P* < 0.0001. Difference against 0/0 μM control: **P < 0.01, ****P < 0.0001. See details on *P*‐values in Appendix Table S12.Analysis and quantification of a time‐dependent increase in ARSA activity in MSD primary fibroblasts (variant FGE Gly247Arg homozygous) simultaneously treated with 10 and 20 μM tazarotene and bexarotene, respectively, up to 21 days. Data represent mean ± SD of 3–6 independent experiments (biological replicates). One‐way ANOVA followed by the Tukey's test for multiple comparisons. Displayed are significance levels for the next significant difference between adjacent treatment times. #### *P* < 0.0001. Difference against 0 days control: *P < 0.05, ****P < 0.0001. See details on *P*‐values in Appendix Table S13.Analysis and quantification of increased sulfatase activities different to ARSA, namely ARSB, GALNS, and STS in MSD primary fibroblasts (variant FGE Gly247Arg homozygous) after 6 days of simultaneous treatment with tazarotene/bexarotene 10/20 μM. Data represent mean ± SD of 3–6 independent experiments (biological replicates). One‐way ANOVA followed by the Tukey's test for multiple comparisons. ***P < 0.001, ****P < 0.0001. See details on *P*‐values in Appendix Table S14.Quantification of ARSA activities in MSD primary fibroblasts with different homozygous *SUMF1* mutations (FGE Gly247Arg, FGE Gly263Val, FGE Ala279Val, FGE Arg349Trp) after 6 days of simultaneous treatment with tazarotene/bexarotene 10/20 μM. Data represent mean ± SD of three independent experiments (biological replicates). One‐way ANOVA followed by the Tukey's test for multiple comparisons. ****P < 0.0001. See details on *P*‐values in Appendix Table S15.Quantification of ARSA activity in MSD patient‐derived iPSCs differentiated into NPCs and unaffected control NPCs controls. Simultaneous treatment with 5 μM tazarotene and 5 μM bexarotene for 4 days. Data represent mean ± SD of six independent experiments (biological replicates). Unpaired *t*‐test. ****P < 0.0001. See details on *P*‐values in Appendix Table S16.Quantification of SGSH activity in MSD patient‐derived iPSCs differentiated into NPCs and unaffected control NPCs controls. Simultaneous treatment with 5 μM tazarotene and 5 μM bexarotene for 4 days. Data represent mean ± SD of three independent experiments (biological replicates). Unpaired *t*‐test. *P < 0.05, ***P < 0.001. See details on *P*‐values in Appendix Table S17. Source data are available online for this figure.

**Figure EV2:**
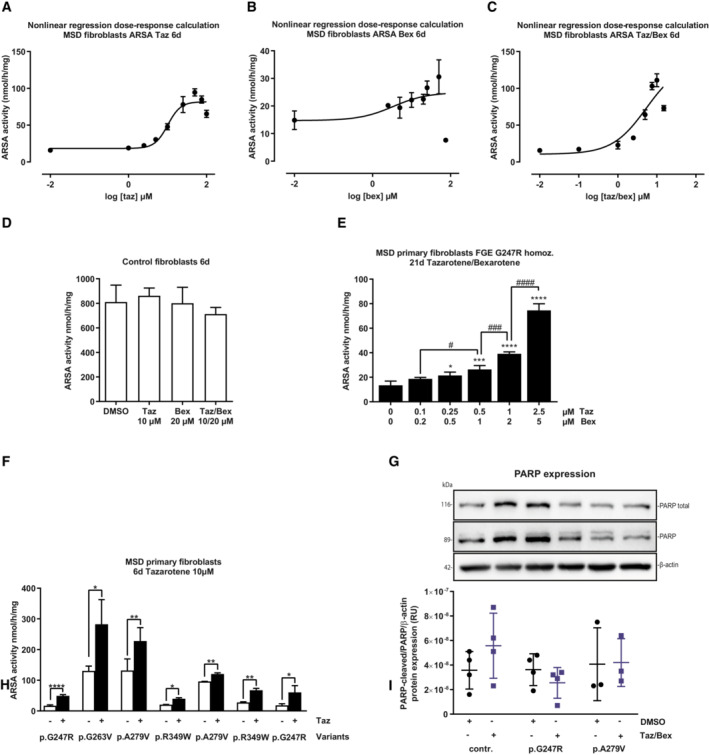
Treatment response and toxicity assessment Dose‐response curve of ARSA activity calculated from data displayed in Fig 2A (variant FGE Gly247Arg homozygous, tazarotene treatment) by nonlinear regression analysis. Drug concentrations are displayed after transformation into log10 values and baseline activity (negative control, DMSO‐only treatment) was manually referred to log‐2. Dots and error bars represent mean ± SD.Dose‐response curve of ARSA activity calculated from data displayed in Fig 2B (variant FGE Gly247Arg homozygous, bexarotene treatment) by nonlinear regression analysis. Drug concentrations are displayed after transformation into log10 values and baseline activity (negative control, DMSO‐only treatment) was manually referred to log‐2. Dots and error bars represent mean ± SD.Dose‐response curve of ARSA activity calculated from data displayed in Fig 2C (variant FGE Gly247Arg homozygous, tazarotene/bexarotene treatment) by nonlinear regression analysis. Drug concentrations are displayed after transformation into log10 values and baseline activity (negative control, DMSO‐only treatment) was manually referred to log‐2. Dots and error bars represent mean ± SD.ARSA activity quantification after treatment of five different control, non‐MSD, fibroblast lines with tazarotene, bexarotene, and tazarotene/bexarotene in combination for 6 days referred to β‐actin amounts and calculation of ARSA activity based on ARSA protein amount (specific ARSA activity). Data represent mean ± SD of five independent experiments (biological replicates) in duplicates to determine the range of normal ARSA activities and treatment response as the basis for the calculation of residual activities in MSD fibroblasts.ARSA activity quantification after simultaneous treatment of MSD primary fibroblasts (variant FGE Gly247Arg homozygous) with increasing concentrations of tazarotene and bexarotene in a fixed combination of 1:2 for 21 days. Data represent mean ± SD of four independent experiments (biological replicates). One‐way ANOVA followed by Tukey's test for multiple comparisons. Displayed are significance levels for the next significant difference between adjacent concentrations/conditions. # *P* < 0.05, ### *P* < 0.001, #### *P* < 0.0001. Difference against 0/0 μM control: *P < 0.05, ***P < 0.001, ****P < 0.0001. See details on *P*‐values in Appendix Table S34.Quantification of ARSA activities in MSD primary fibroblasts with different homozygous *SUMF1* mutations (FGE Gly247Arg, FGE Gly263Val, FGE Ala279Val, FGE Arg349Trp) after 6 days of treatment with tazarotene 10 μM. Data represent mean ± SD of 2–5 independent experiments (biological replicates). One‐way ANOVA followed by Tukey's test for multiple comparisons. *P < 0.05, **P < 0.01, ****P < 0.0001. See details on *P*‐values in Appendix Table S35.Upper panel: Representative pictures of Western Blot analysis of (PARP) and cleaved PARP in tazarotene/bexarotene‐treated MSD primary fibroblasts (variant FGE Gly247Arg, FGE Ala279Val, homozygous) and control fibroblasts. β‐actin expression served as loading control. Lower panel: Quantification of protein amounts from western blots displayed as ratio cleaved PARP to total PARP expression normalized to β‐actin. Data represent mean ± SD of 3–4 independent experiments (biological replicates). Unpaired *t*‐tests. No statistical differences. RU, relative units. Source data are available online for this figure.

To investigate the effect of tazarotene and bexarotene on primary, nonimmortalized MSD fibroblasts, we treated a previously described patient‐derived fibroblast line with the severe homozygous *SUMF1* mutation (c.739G > C, pGly.247Arg; Schlotawa *et al*, 2011) with increasing concentrations of tazarotene and bexarotene at an extended standard treatment time of 6 days. Tazarotene treatment led to a dose‐dependent, significant increase in ARSA activity at concentrations as low as 5 μM (EC50 10.1 μM, Figs 2A and EV2A). Applying the same experimental conditions, bexarotene increased ARSA activity slightly (EC 50 3.4 μM, Figs 2B and EV2B). However, again, the combination of both drugs at a fixed combination 1:2 of tazarotene:bexarotene led to a dose‐dependent increase in ARSA activity with significant differences against DMSO‐treated controls as low as 2.5/5 μM tazarotene/bexarotene (EC 50 5.3/10.6 μM, Figs 2C and EV2C). To assess time dependency, we extended treatment times at a concentration of 10 μM tazarotene and 20 μM bexarotene up to 21 days. A maximum ARSA activity was reached after 9 days with no changes up to 21 days of treatment (ARSA activity 9 days: 175.8 nmol/h/mg (SD 25.9 nmol/h/mg), 10.9‐fold increase, Fig 2D). This activity reached 21.6% of mean ARSA activity determined in five untreated non‐LSD primary fibroblast lines (810.9 nmol/h/mg, SD 136.9 nmol/h/mg, Fig EV2D).

Next, we used lower concentrations of tazarotene and bexarotene ranging from 0.1/0.2 to 2.5/5 μM and extended the treatment time to 21 days to assess the lower range of working concentrations. We still detected a significant increase in ARSA activity at concentrations as low as 0.25/0.5 μM tazarotene/bexarotene (Fig EV2E). To test the drugs' effect on multiple sulfatases, we treated cells with 10 μM tazarotene and 20 μM bexarotene for 6 days and saw a significant increase in activities of lysosomal sulfatases arylsulfatase B (ARSB, 2.6‐fold) and GALNS (3.3‐fold), as well as the nonlysosomal sulfatase steryl sulfatase (STS, 7.7‐fold, Fig 2E).

To explore whether the activity increase occurs with other *SUMF1* variants, we treated four different homozygous primary MSD patient fibroblast lines under standard conditions (10/20 μM tazarotene/bexarotene, 6 days). Baseline ARSA activities varied between 17.7 and 131.2 nmol/h/mg reaching 2 to 16% of control activities in non‐MSD fibroblasts (Figs 2F and EV2D). All fibroblast lines showed a significant increase in ARSA activity between 2‐fold (line 3, FGE Ala279Val) and 5.5‐fold (line 1, FGE Gly247Arg; Fig 2F). The highest ARSA activity upon treatment was 324.5 nmol/h/mg, reaching 40% of control activities. Treatment of seven different homozygous primary MSD fibroblast lines with 10 μM tazarotene confirmed a mutation‐independent response also to single treatment (ARSA activity increase 1.3–3.3‐fold, maximum activity 282.4 nmol/h/mg, 35% of control activity, Fig EV2F).

As a side effect, a 6‐day treatment with tazarotene, bexarotene, and tazarotene/bexarotene resulted in reduced cell proliferation when compared to cells treated with DMSO only. Cell culture conditions (cell density, cultivation time) did not influence endogenous ARSA activity in MSD fibroblasts until 9 days of cultivation (Appendix Supplementary Results, and Figs S5A–D and S6A–F). To determine whether programmed cell death contributed to the observed decrease in cell growth we quantified cleaved Poly(ADP‐ribose) polymerase (PARP) expression levels, a marker of programmed cell death (Duriez & Shah, 1997). No significant differences in PARP levels between tazarotene/bexarotene and DMSO‐treated MSD and control fibroblasts were detected (Fig EV2G).

To evaluate the efficacy of tazarotene and bexarotene on cell types different from fibroblasts, an iPSC line was generated from a MSD patient (compound heterozygous for *SUMF1* mutations c.463T > C, p.Ser155Pro and c.1034G > A, p.Arg345His, Appendix Fig S7A–F) Control and MSD patient‐derived iPSC lines were differentiated into neuronal progenitor cells (NPCs) and treated with 5/5 μM tazarotene/bexarotene for 4 days. Baseline activities of ARSA and N‐sulfoglucosamine sulfohydrolase (SGSH) in MSD NPCs were 11 (SD 0.49) and 0.22 (SD 0.03) nmol/h/mg, reaching 34 and 9.6% of control activities, respectively. Both activities significantly increased upon treatment (ARSA 17.6 nmol/h/mg, SD 0.5‐, 1.6‐fold; SGSH 0.46 nmol/h/mg, SD 0.01, 2.1‐fold). Control iPSCs showed a slight increase in ARSA activity (1.2‐fold) and slightly reduced SGSH activity (1.3‐fold) upon treatment (Fig 2G and H).

**Figure 3:**
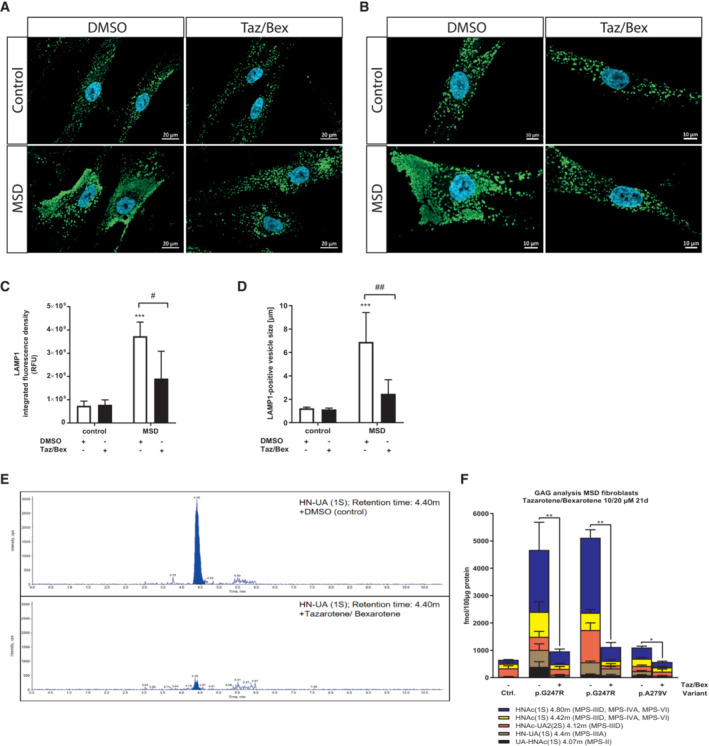
Tazarotene and bexarotene reverse lysosomal pathophysiology in MSD primary fibroblasts Representative confocal images of control and MSD fibroblasts with either tazarotene/bexarotene (10/20 μM, 6 days). Labelling with anti‐LAMP1 antibody (green fluorescence) and DAPI (nuclei, blue). Scale bar = 20 μm.Representative confocal images of control and MSD fibroblasts with either tazarotene/bexarotene (10/20 μM, 6 days). Labelling with anti‐LAMP1 antibody (green fluorescence) and DAPI (nuclei, blue). Scale bar = 10 μm.Quantification of the total intensity of LAMP1‐green fluorescence. *N* = 20 images and 13 z‐series optical sections per condition with a step size of 0.26 μm, displayed at maximum extension and automated equalization of brightness. Data represent mean ± SD of three independent experiments (biological replicates). One‐way ANOVA followed by the Tukey's test for multiple comparisons. ***P < 0.001 (DMSO‐treated MSD cells compared with DMSO‐treated control cells), #*P* < 0.05 (MSD DMSO vs. MSD treated). RFU, relative fluorescence units. See details on *P*‐values in Appendix Table S18.Quantification of LAMP1‐green fluorescence vesicle size (μm). *N* = 20 images and 13 z‐series optical sections per condition with a step size of 0.26 μm, displayed at maximum extension and automated equalization of brightness. Data represent mean ± SD of three independent experiments (biological replicates). One‐way ANOVA followed by the Tukey's test for multiple comparisons. ***P < 0.001 (DMSO‐treated MSD cells compared with DMSO‐treated control cells), ## *P* < 0.01 (MSD DMSO vs. MSD treated). See details on *P*‐values in Appendix Table S19.Representative spectra of the heparan sulfate‐derived oligosaccharide HN‐UA(1 S) as GAG marker analyzed via mass spectrometry in DMSO (control) treated MSD primary fibroblasts (variant FGE Gly247Arg homozygous, upper panel) and in the same cell line simultaneously treated with 10 and 20 μM tazarotene and bexarotene for 21 days (lower panel). Integrated peak areas correspond to the amount of HN‐UA(1S).Quantification of specific oligosaccharide markers for GAG species in three different MSD primary fibroblast lines with FGE variants as indicated and one control fibroblast line after 21‐day treatment with tazarotene/bexarotene. 10/20 μM. Data represent mean ± SD of 3–8 independent experiments (biological replicates). The unpaired *t*‐test compares DMSO conditions of control and MSD fibroblasts and treated and DMSO control conditions in every MSD cell line for every marker. Details on significance levels for each marker are summarized in Table EV1. The lowest significance levels among individual markers for treatment and DMSO condition in MSD fibroblast lines are displayed. *P < 0.05, **P < 0.01. Source data are available online for this figure.

Enlarged lysosomes are a hallmark of cellular pathophysiology in lysosomal disorders including MSD (Xu *et al*, 2014). To analyze the effect of tazarotene and bexarotene treatment on enlarged lysosomes we quantified lysosomal‐associated membrane protein 1 (LAMP1) integrated fluorescence density in MSD and control fibroblasts (Fig 3A and B). Vehicle‐treated MSD fibroblasts displayed increased LAMP1 integrated fluorescence density as compared to control fibroblasts. Upon treatment with tazarotene and bexarotene we observed a significant reduction in LAMP1 integrated fluorescence intensity compared with DMSO‐treated MSD fibroblasts. Fluorescence intensity of tazarotene‐ and bexarotene‐treated control fibroblasts was unchanged compared with DMSO‐treated control fibroblasts (integrated fluorescence density control fibroblasts: DMSO 7.4 × 10^8^, Taz/Bex 7.9 × 10^8^; MSD fibroblasts: DMSO 3.7 × 10^9^, Taz/Bex 1.9 × 10^9^, Fig 3C). In addition, the size of lysosomes in MSD fibroblasts was increased compared with non‐MSD fibroblasts under DMSO conditions. We observed a significant reduction in lysosomal size upon treatment with tazarotene and bexarotene as compared to untreated MSD fibroblasts. Again, we detected no differences in tazarotene and bexarotene versus DMSO‐treated control fibroblasts (control fibroblasts: DMSO: 1.23 μm, ±0.1 μm, Taz/Bex: 1.14 μm, ±0.1 μm, MSD fibroblasts: DMSO: 6.9 μm, ±2.5 μm, Taz/Bex: 2.47 μm, ±1.2 μm; Fig 3D).

In MSD patients, glycosaminoglycans (GAGs) and sulfatides accumulate in tissues and organs (Guerra *et al*, 1990; Macaulay *et al*, 1998). Although we did not detect any sulfatides in MSD or control fibroblasts, we detected five different GAG subspecies in control and MSD primary fibroblasts by adapting a mass spectrometry method for GAG detection for its use in lysates from fibroblasts (Fuller *et al*, 2004; see Materials and Methods for details). The amount of all five GAG subspecies was increased in one MSD primary fibroblasts line (MSD2, p.Gly247Arg) compared with unaffected control fibroblasts. The amount of four different GAG subspecies was elevated in two more MSD fibroblast lines compared with control fibroblasts (MSD1, p.Gly247Arg, MSD3 p.Ala279Val; Fig 3E and F, and Table EV1).

To evaluate whether tazarotene and bexarotene could reduce GAG accumulation, cells were treated for 21 days to allow sufficient time for the clearance of accumulated storage material. We detected a significant reduction in all glycosaminoglycan subspecies in three primary MSD fibroblast lines compared with DMSO treatment (Fig 3E and F, and Table EV1).

### Tazarotene and bexarotene work via retinoic acid receptors and induce gene expression in MSD patient cells

**Figure 4:**
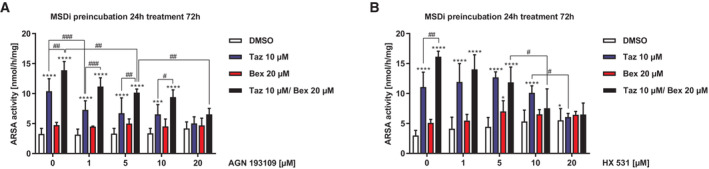
Tazarotene and bexarotene work through retinoid receptors in MSD fibroblasts ARSA activity quantification upon treatment of MSDi cells with 10 μM tazarotene, 20 μM bexarotene, and 10 μM tazarotene and 20 μM bexarotene in combination with increasing concentrations of the pan‐RAR receptor antagonist AGN 193109 (24 h pretreatment) for 72 h. Data represent mean ± SD of 3–11 independent experiments (biological replicates). Two‐way ANOVA followed by the Tukey's test for multiple comparisons. Displayed are significance levels for the next significant difference between adjacent concentrations/conditions. # *P* < 0.05, ## *P* < 0.01, ### *P* < 0.001. Difference against 0 μM DMSO control: **P < 0.01, ***P < 0.001, ****P < 0.0001. See details on *P*‐values in Appendix Table S20.ARSA activity quantification upon treatment of MSDi cells with 10 μM tazarotene, 20 μM bexarotene, and 10 μM tazarotene and 20 μM bexarotene in combination with increasing concentrations of the pan‐RXR receptor antagonist HX 531 (24 h pretreatment) for 72 h. Data represent mean ± SD of 4–14 independent experiments (biological replicates). Two‐way ANOVA followed by Tukey's test for multiple comparisons. Displayed are significance levels for the next significant difference between adjacent concentrations/conditions. # *P* < 0.05, ## *P* < 0.01. Difference against 0 μM DMSO control: ****P < 0.0001. See details on *P*‐values in Appendix Table S21. Source data are available online for this figure.

Retinoids bind to the retinoic acid receptors RAR and RXR, which, after homo‐ or heterodimerization, bind to DNA elements in promotors and initiate transcription (di Masi *et al*, 2015). To elucidate, which retinoic acid receptors are involved in the treatment response, MSDi cells were pretreated with AGN193109, a pan‐RAR antagonist (Standeven *et al*, 1996), and HX531, a pan‐RXR antagonist (Yotsumoto *et al*, 2005), followed by additional treatment with tazarotene and bexarotene alone or in combination, respectively. Incubation of MSDi cells with increasing concentrations of AGN193109 as single agent did not affect ARSA activity. Tazarotene‐induced ARSA activity increase was abolished upon AGN193109 treatment. Bexarotene treatment showed no significant increase in ARSA activity in this experiment and no detectable changes with additional AGN193109 treatment. Tazarotene/bexarotene‐induced ARSA activity increase was abolished upon AGN193109 treatment in a dose‐dependent manner but still higher than tazarotene alone except for the highest AGN193109 concentration (Fig 4A). HX531 as single agent showed minimal agonistic function and increased ARSA activities at 20 μM concentration. Tazarotene‐induced ARSA activity increase was abolished but only at the highest HX531 concentration (20 μM). Again, bexarotene‐only treatment did not increase ARSA activity with no significant changes upon HX531 treatment. However, increasing HX531 doses reduced the ARSA activity increase by tazarotene/bexarotene thereby abolishing the additional ARSA activity increase compared with tazarotene‐only treatment (Fig 4B). In summary, blocking of RAR receptors inhibited ARSA activity increase through tazarotene/bexarotene and tazarotene, RXR receptor blocking predominantly inhibited tazarotene/bexarotene response. No significant differences were detected with the blocking of both receptors and treatment with bexarotene only.

**Figure EV3:**
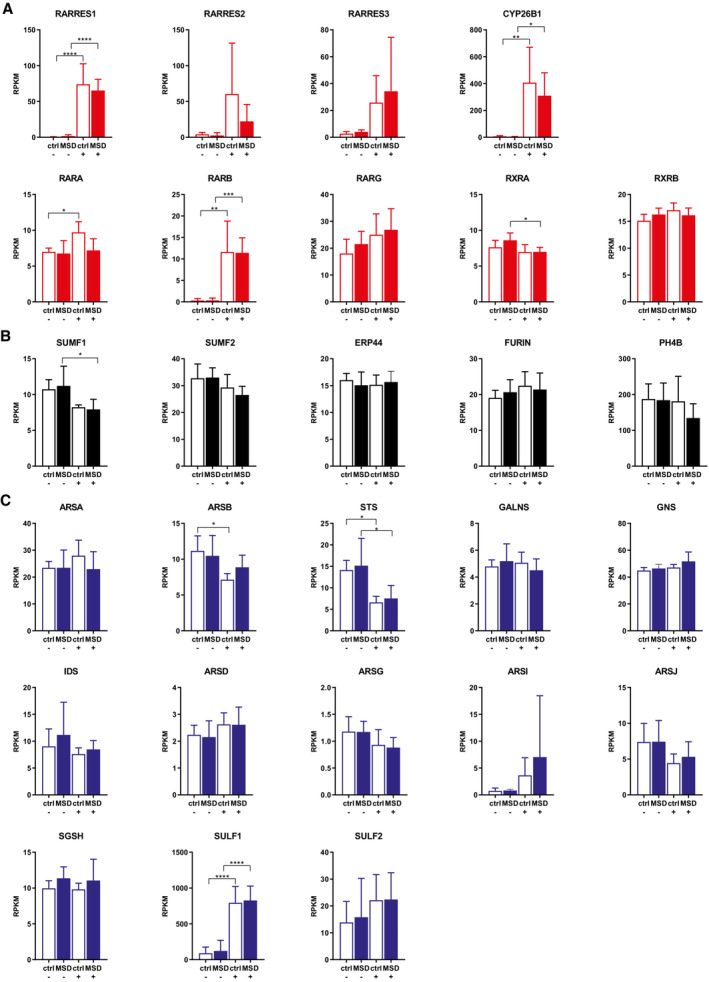
Transcriptional response of MSD and retinoic acid gene targets upon tazarotene/bexarotene treatment Gene expression analysis of genes in relation to retinoic acid receptor signaling of six different MSD primary fibroblast lines and five different control fibroblast lines after 6 days of treatment with tazarotene/bexarotene (10/20 μM) and DMSO, respectively. Changes in RPKM (reads per kilobase million) are displayed as mean ± SD of three independent experiments (biological replicates). One‐way ANOVA test followed by Tukey's test for multiple comparisons. *P < 0.05, **P < 0.01, ***P < 0.001, ****P < 0.0001. See details on *P*‐values in Appendix Table S36.Gene expression analysis of *SUMF1*‐ and FGE‐interacting partners (nonsulfatases) of six different MSD primary fibroblast lines and five different control fibroblast lines after 6 days of treatment with tazarotene/bexarotene (10/20 μM) and DMSO, respectively. Changes in RPKM (reads per kilobase million) are displayed as mean ± SD of three independent experiments (biological replicates). One‐way ANOVA test followed by Tukey's test for multiple comparisons. *P < 0.05. See details on *P*‐values in Appendix Table S37.Gene expression analysis of sulfatases of six different MSD primary fibroblast lines and five different control fibroblast lines after 6 days of treatment with tazarotene/bexarotene (10/20 μM) and DMSO, respectively. Changes in RPKM (reads per kilobase million) are displayed as mean ± SD of three independent experiments (biological replicates). One‐way ANOVA test followed by Tukey's test for multiple comparisons. *P < 0.05, ****P < 0.0001. See details on *P*‐values in Appendix Table S38. Source data are available online for this figure.

To explore how tazarotene/bexarotene treatment affected gene expression in MSD cells we subjected treated and untreated MSD and control fibroblasts to RNAseq analysis and were able to analyze the expression of 16,385 genes. Selected genes, well‐known to react to retinoic acid treatment (Napoli, 2017), showed increased transcription upon tazarotene/bexarotene treatment thereby indicating a successful treatment response. RNA expression of *RARRES1*, *CYP26B1*, and *RARB* was concordantly significantly increased for both MSD and control fibroblasts upon treatment compared with untreated controls. RNA expression of *RARRES 2* and *3* showed a concordant trend towards increased expression. RNA expression of other RAR and RXR receptors was unchanged except for *RARA* that was only increased in treated control fibroblasts and *RXRA* that was decreased in treated MSD fibroblasts. No expression of *RXRG* could be detected in either condition (Fig EV3A).

We assumed that increased transcription of sulfatases genes, known interacting partners of FGE, or *SUMF1*, the only known activating factor for sulfatases, could be an underlying cause for the sulfatase activity increase upon tazarotene and bexarotene treatment in MSD cells. However, RNA‐expression analysis revealed significantly decreased *SUMF1* transcription in MSD fibroblasts upon treatment and no changes in transcription levels of detectable genes for FGE‐interacting partners *SUMF2*, *P4HB*, *ERP44*, and *FURIN* (Fig EV3B). From 17 encoded sulfatases in the human genome, all transcripts except four (*ARSE*, *ARSF*, *ARSH*, *ARSK*) could be detected. Transcription of those sulfatases that showed increased catalytic activity upon tazarotene/bexarotene treatment was unchanged (*ARSA*, *ARSB*, *GALNS*), while *STS* transcription was significantly reduced. Among other sulfatases, *ARSI* and *SULF2* showed a trend towards increased transcription, and *SULF1* was the only sulfatase with increased transcription in MSD fibroblasts upon treatment (Fig EV3C).

**Figure 5:**
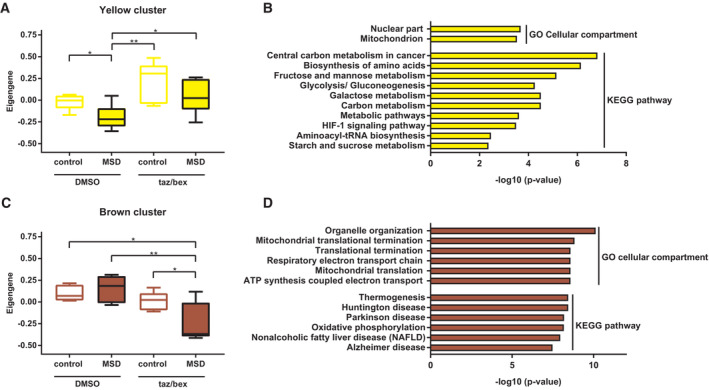
Transcription response of tazarotene and bexarotene treatment in MSD fibroblasts Eigengene analysis of the yellow gene cluster as determined by WGCN analysis after RNA sequencing of six different MSD primary fibroblast lines and five different control fibroblast lines and treatment with tazarotene/bexarotene 10/20 μM or DMSO only, respectively, for 6 days. Data represent min to max box and whisker blots of Eigengene values ± SD of three independent experiments (biological replicates). One‐way ANOVA test followed by Tukey's test for multiple comparisons. *P < 0.05, **P < 0.01. See details on *P*‐values in Appendix Table S22.GO and KEGG pathway analysis of genes in the yellow cluster and log10 value of *P*‐values.Eigengene analysis of the yellow gene cluster as determined by WGCN analysis after RNA sequencing of six different MSD primary fibroblast lines and five different control fibroblast lines and treatment with tazarotene/bexarotene 10/20 μM or DMSO only, respectively, for 6 days. Data represent min to max box and whisker blots of Eigengene values ± SD of three independent experiments (biological replicates). One‐way ANOVA test followed by Tukey's test for multiple comparisons. *P < 0.05, **P < 0.01. See details on *P*‐values in Appendix Table S23.GO and KEGG pathway analysis of genes in the brown cluster and log10 value of *P*‐values. Source data are available online for this figure.

**Figure EV4:**
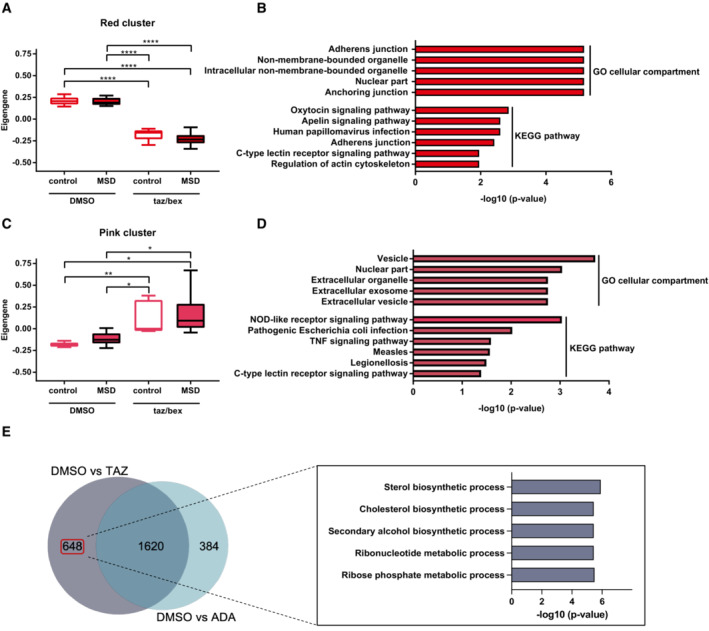
Transcriptional response upon tazarotene and bexarotene treatment in MSD and control fibroblasts and differential transcriptional response in MSD fibroblasts upon treatment with tazarotene and adapalene Eigengene analysis of the red gene cluster as determined by WGCN analysis after RNA sequencing of six different MSD primary fibroblast lines and five different control fibroblast lines and treatment with tazarotene/ bexarotene 10/20 μM or DMSO only, respectively, for 6 days. Data represent min to max box and whisker blots of Eigengene values ± SD of three independent experiments (biological replicates). One‐way ANOVA test followed by Tukey's test for multiple comparisons. ****P < 0.0001. See details on *P*‐values in Appendix Table S39.GO and KEGG pathway analysis of genes in the red cluster and log10 value of *P*‐values. GO and KEGG pathway analysis of genes in the yellow cluster and their log10 value of *P*‐values as the display for changes in gene expression.Eigengene analysis of the red gene cluster as determined by WGCN analysis after RNA sequencing of six different MSD primary fibroblast lines and five different control fibroblast lines and treatment with tazarotene/bexarotene 10/20 μM or DMSO only, respectively, for 6 days. Data represent min to max box and whisker blots of Eigengene values ± SD of three independent experiments (biological replicates). One‐way ANOVA test followed by Tukey's test for multiple comparisons. *P < 0.05, **P < 0.01. See details on *P*‐values in Appendix Table S40.GO and KEGG pathway analysis of genes in the red cluster and log10 value of *P*‐values.Differential gene expression analysis after treatment of seven MSD primary fibroblast lines with tazarotene (sulfatase activity response) and adapalene (no sulfatase activity response) in triplicates for 6 days. Treatment with DMSO served as a negative control. Venn diagram and number of exclusively regulated genes for tazarotene treatment (TAZ) versus DMSO condition (left) and adapalene treatment (ADA) versus DMSO (right), as well as the number of overlapping genes identically regulated by both tazarotene and adapalene. GO pathway analysis and log10 value of *P*‐values for tazarotene‐only regulated genes. Source data are available online for this figure.

To gain further insights into differential gene expression, we performed a weighted correlation network (WGCN) co‐expression analysis from the RNAseq data and identified 16 co‐expression modules. Using the Eigen‐expression values of these modules for comparison, we detected four clusters that exhibited significant differences among groups, namely the yellow, brown, red, and pink modules. Whereas the red and pink clusters showed concordant expression in untreated control and MSD cells followed by concordant deregulation upon treatment (Fig EV4A and C), the yellow and brown clusters, most interestingly, showed significant differences in the deregulation of genes between MSD and control fibroblasts: The yellow cluster was downregulated when comparing DMSO‐treated MSD patient cells (disease condition) to DMSO‐treated control fibroblasts (normal condition) and this difference was ameliorated after tazarotene/bexarotene treatment (Fig 5A). These data suggest that the tazarotene/bexarotene treatment helps to reinstate physiological gene expression. GO‐term and pathway analysis showed that genes of the yellow cluster represent the nuclear and mitochondrial compartment and pathways linked to metabolic processes and HIF‐1 signaling (Fig 5B). The genes of the brown cluster were significantly downregulated in tazarotene/bexarotene‐treated patient cells only (Fig 5C) and also represent mitochondria, while the functional pathways are mainly linked to pathological conditions (Fig 5D). Genes in the red cluster were significantly downregulated upon treatment in control and patient cells and were linked to organelle transport and intracellular signaling pathways (Fig EV4A and B). Genes in the pink cluster showed a significantly increased expression in treated control fibroblasts and MSD fibroblasts. Pathway analysis revealed intracellular signaling pathways, too, in addition to pathologies resulting from infectious diseases and genes representing intra‐ and extracellular vesicles (Fig EV4C and D).

In an attempt to further identify genes and pathways that are mediating sulfatase activity restoration upon treatment, we compared the transcriptional response of tazarotene (“positive” for sulfatase activity restoration) and the response of a retinoid that is incapable (“negative”) of restoring sulfatase activities in MSD cells (Fig 1E). Through a set of preparatory experiments, we chose adapalene to serve as a control retinoid that provokes expression of retinoid targets genes in MSD cells without increasing sulfatase activities (please see Appendix Supplementary Results for details, and Figs S8A and B, S9A–D, and S10). We treated seven MSD fibroblast lines with either adapalene, tazarotene, or DMSO (control) in triplicates for 6 days and referred all samples to total RNA sequencing and differential gene expression analysis (Appendix Fig S11A). We identified the expression of 10,992 genes. For quality control, we analyzed a subset of retinoid target genes, which were significantly upregulated (*RARB, CYP26B1, RARRES1*) for both tazarotene and adapalene treatment compared with DMSO conditions, indicating a positive treatment response (Appendix Fig S11B). Analyzing all cell lines we found 1,042 genes differentially regulated when we compared gene expression between tazarotene and adapalene treatment (Appendix Fig S11C). GO biological process pathway analysis of the significantly differentially expressed genes by tazarotene (positive retinoid) revealed sterol synthesis and cholesterol synthesis pathways (Appendix Fig S11D) whereas pathways regulated by adapalene were mostly developmental pathways (Appendix Fig S11D). When comparing tazarotene‐ and adapalene‐induced gene expression against DMSO conditions (untreated), respectively, 2,268 genes were regulated upon tazarotene treatment and 2004 upon adapalene treatment compared with DMSO conditions. 1,620 genes were identically regulated by both tazarotene or adapalene treatment, whereas 684 genes were exclusively regulated upon tazarotene treatment and 384 genes exclusively upon adapalene treatment (Fig EV4E and Appendix Fig S11F and G). GO biological pathway analysis of genes exclusively regulated by adapalene treatment identified phosphatidylinositol‐mediated signaling pathways (Appendix Fig S11G). We focused on genes exclusively regulated upon tazarotene treatment because of its positive action on sulfatase activity restoration and identified 313 upregulated and 335 downregulated genes. GO biological process pathway analysis in the group of genes exclusively regulated by tazarotene treatment identified again sterol and cholesterol biosynthesis (Fig EV4E). Upregulated individual genes in these pathways, among others, were *SREBPF1*, *SREBF2*, and *INSIG1* (Appendix Fig S11H) coding for sterol regulatory element‐binding proteins 1 and 2 (SREBP1, SREBP2) and insulin‐induced gene proteins (INSIG). Together with the SREBP cleavage‐activating protein (SCAP), these proteins are part of the SREBP‐SCAP‐INSIG complex in the ER sensing and controlling ER and cellular cholesterol content (Brown *et al*, 2018; Yang *et al*, 2002). In order to reveal any functional involvement of the complex in tazarotene‐mediated sulfatase activity restoration we treated immortalized MSD cells with tazarotene and fatostatin for 3 days. Fatostatin suppresses SCAP/SREBP translocation (Cheng *et al*, 2018). Whereas treatment with increasing concentrations of fatostatin did not increase ARSA activity, tazarotene did. Simultaneous treatment with tazarotene (10 μM) and increasing fatostatin concentrations caused a dose‐dependent ARSA activity decrease (Appendix Fig S11I).

### Tazarotene and bexarotene require residual FGE function and increase the half‐life of MSD‐causing FGE variants

**Figure 6:**
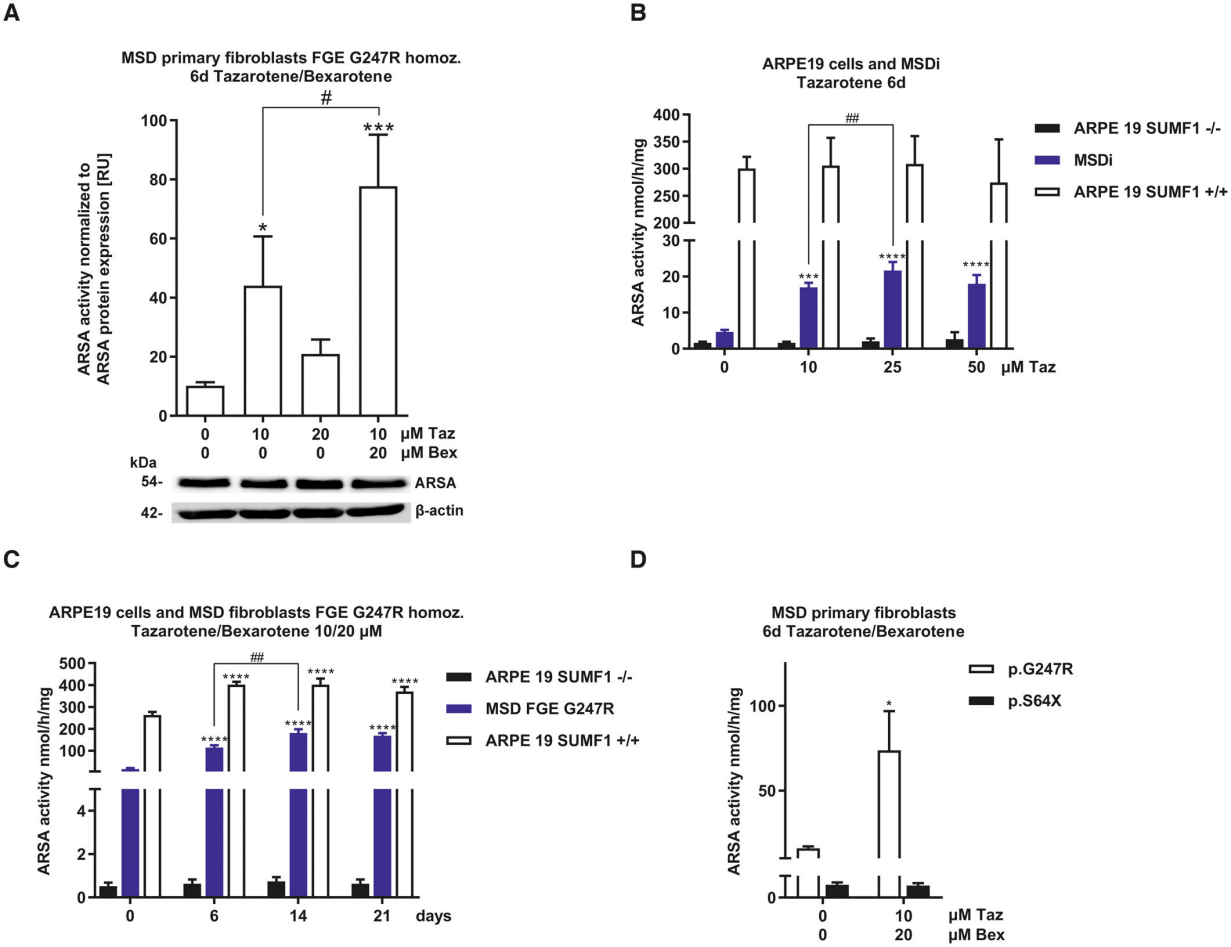
Tazarotene and bexarotene treatment response in MSD fibroblasts requires residual FGE function ARSA protein amount quantification after treatment of MSD primary fibroblasts (variant FGE Gly247Arg homozygous) with tazarotene, bexarotene, and tazarotene/bexarotene in combination for 6 days referred to β‐actin amounts and normalization of ARSA activity based on ARSA protein amount (specific ARSA activity). Data represent mean ± SD of three independent experiments (biological replicates). One‐way ANOVA followed by Tukey's test for multiple comparisons. Displayed are significance levels for the next significant difference between adjacent concentrations. # *P* < 0.05. Difference against 0/0 μM control: * *P* < 0.05, *** *P* < 0.001. See details on *P*‐values in Appendix Table S24.Quantification of ARSA activities in CRISPR/Cas9 generated ARPE19 SUMF1 −/− cells and appropriate controls (ARPE19 wild‐type, MSDi) after 6 days of simultaneous treatment with increasing concentration of tazarotene. Data represent mean ± SD of three independent experiments (biological replicates). One‐way ANOVA followed by Tukey's test for multiple comparisons. Displayed are significance levels for the next significant difference between adjacent concentrations. ## *P* < 0.01. Difference against 0 μM control: ***P < 0.001, ****P < 0.0001. See details on *P*‐values in Appendix Table S25.Quantification of ARSA activities in CRISPR/Cas9 generated ARPE19 SUMF1 −/− cells and appropriate controls (ARPE19 wild‐type, MSD primary fibroblasts (variant FGE Gly247Arg homozygous)) after 6 days of simultaneous treatment with tazarotene/bexarotene 10/20 μM for up to 21 days. Data represent mean ± SD of three independent experiments (biological replicates). One‐way ANOVA followed by Tukey's test for multiple comparisons. Displayed are significance levels for the next significant difference between adjacent concentrations. ## *P* < 0.01. Difference against 0 days control: ****P < 0.0001. See details on *P*‐values in Appendix Table S26.ARSA activity quantification after simultaneous treatment of MSD primary fibroblasts (variants FGE Gly247Arg homozygous, FGE Ser64Ter homozygous) with tazarotene and bexarotene. Treatment time 6 days. Data represent mean ± SD of three independent experiments (biological replicates). One‐way ANOVA followed by Tukey's test for multiple comparisons. *P < 0.05. See details on *P*‐values in Appendix Table S27. Source data are available online for this figure.

**Figure EV5:**
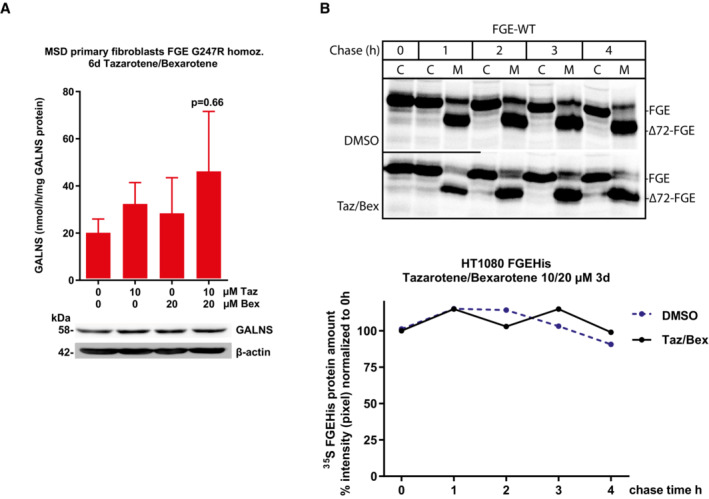
Sulfatase‐specific activity and protein expression and protein stability of wildtype FGE upon treatment GALNS protein amount quantification after treatment of MSD primary fibroblasts (variant FGE Gly247Arg homozygous) with tazarotene, bexarotene, and tazarotene/bexarotene in combination for 6 days referred to β‐actin amounts and calculation of GALNS activity based on GALNS protein amount (specific GALNS activity). Data represent mean ± SD three independent experiments (biological replicates). One‐way ANOVA followed by Tukey's test for multiple comparisons.Pulse‐chase‐experiment in HT 1080 FGE wild‐type (wt) cells after pretreatment with tazarotene/bexarotene and DMSO (control) for 3 days. Upper panel: autoradiogram of intracellular (full‐length FGE, C) and cleaved and secreted (Δ72 FGE, M) ^35^S isotope labeled FGE protein in either condition with a chase time of 4 h. Lower panel: Quantification of the autoradiogram, *n* = 1 experiment. Source data are available online for this figure.

To further elucidate how tazarotene/bexarotene increase sulfatase activities and improve lysosomal pathology in MSD cells despite no changes in the transcription of respective genes, we analyzed whether treatment led to an increase in sulfatase protein levels. ARSA and GALNS protein levels were unaltered upon treatment of MSD fibroblasts with tazarotene/bexarotene using standard concentrations and treatment times (Figs 6A and EV5A, lower panels). However, when we analyzed specific ARSA activity by normalizing sulfatase activity to the ARSA protein amount, specific ARSA activity increased significantly in MSD fibroblasts after 6 days of tazarotene and tazarotene/bexarotene treatment (Fig 6A upper panel). Specific GALNS activity showed a trend towards increased activity (Fig EV5A upper panel). The increase in sulfatase activities without changes in protein expression levels suggests that tazarotene and bexarotene positively influence the activation process of sulfatases and act to boost FGE activity, the only known enzyme to activate cellular sulfatases (Cosma *et al*, 2003). To explore whether FGE mediates the response to tazarotene and bexarotene in MSD cells, we used ARPE19 retinal pigment epithelial cells with CRISPR/Cas9 generated *SUMF1*‐gene knock‐out (ARPE19 SUMF1^−/−^, Appendix Fig S12) and appropriate controls (ARPE19 wt, MSDi) and treated with increasing concentrations of tazarotene. Remarkably, no increase in ARSA activity was observed when *SUMF1* knock‐out cells were treated with tazarotene (Fig 6B). Treatment with tazarotene and bexarotene at standard concentrations up to 21 days also failed to increase ARSA activity in ARPE19 *SUMF1* ^−/−^ cells (Fig 6C). In addition, treatment of a previously described primary MSD patient‐derived fibroblast line with a homozygous stop mutation and no FGE expression (FGE p.Ser64Ter; Schlotawa *et al*, 2019) also did not lead to any increase in ARSA activity (Fig 6D).

The majority of MSD cases are caused by hypomorphic *SUMF1* mutations resulting in instability and early degradation of FGE variants (Schlotawa *et al*, 2011). Increased intracellular half‐life of FGE variants has been shown to correlate with increased sulfatase activities. PDI is a pivotal interacting partner of FGE that preferentially binds misfolded FGE proteins, impairs their residual enzyme activity, and determines their fate by early degradation (Schlotawa *et al*, 2018). We analyzed the role of PDI in treated MSD cells. In addition to unchanged PDI transcription (*PH4B*, see above), we could not detect any differences in PDI protein expression upon tazarotene/bexarotene treatment (see Appendix Supplementary Results for details and Fig S13; Schlotawa *et al*, 2018). However, tazarotene/bexarotene treatment decreased a PDI‐mediated inhibition of FGE variants' residual activity (Appendix Supplementary Results and Fig S14A–D).

**Figure 7:**
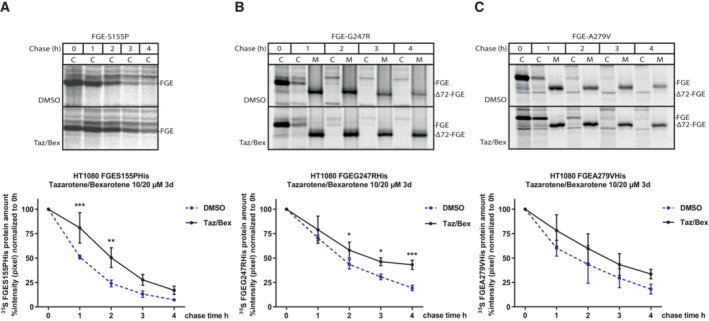
Improved FGE variant protein stability after tazarotene and bexarotene treatment Pulse‐chase‐experiment in HT1080 FGE Ser155Pro cells after pretreatment with tazarotene/bexarotene and DMSO (control) for 3 days. Upper panel: representative autoradiogram of intracellular (C) ^35^S isotope labeled FGE protein in either condition with a chase time of 4 h. Lower panel: quantification of ^35^S isotope labeled intracellular FGE protein amounts. Data represent mean ± SD of three independent experiments (biological replicates). One‐way ANOVA followed by Tukey's test for multiple comparisons. **P < 0.01, ***P < 0.001. See details on *P*‐values in Appendix Table S28.Pulse‐chase‐experiment in HT 1080 FGE Gly247Arg cells after pretreatment with tazarotene/bexarotene and DMSO (control) for 3 days. Upper panel: representative autoradiogram of intracellular (full‐length FGE, C) and cleaved and secreted (Δ72 FGE, M) ^35^S isotope labeled FGE protein in either condition with a chase time of 4 h. Lower panel: quantification of ^35^S isotope labeled intracellular and secreted FGE protein amounts. Data represent mean ± SD of three independent experiments (biological replicates). One‐way ANOVA followed by Tukey's test for multiple comparisons. *P < 0.05, ***P < 0.001. See details on *P*‐values in Appendix Table S29.Pulse‐chase‐experiment in HT 1080 FGE Ala279Val cells after pretreatment with tazarotene/bexarotene and DMSO (control) for 3 days. Upper panel: representative autoradiogram of intracellular (full‐length FGE, C) and cleaved and secreted (Δ72 FGE, M) ^35^S isotope labeled FGE protein in either condition with a chase time of 4 h. Lower panel: quantification of ^35^S isotope labeled intracellular and secreted FGE protein amounts. Data represent mean ± SD of three independent experiments (biological replicates). One‐way ANOVA followed by Tukey's test for multiple comparisons. See details on *P*‐values in Appendix Table S30. Source data are available online for this figure.

Such an increase in FGE activity could be a result of less PDI interaction due to improved FGE variant protein stability. We finally assessed whether the FGE half‐life changed upon tazarotene and bexarotene treatment performing a previously described pulse‐chase experiment with HT1080 cells stably expressing FGE variants as a cell model (Schlotawa *et al*, 2011, 2018). Because wild‐type FGE and variant FGE, depending on the type of mutation, is also secreted upon overexpression we assessed levels of intracellular and secreted FGE protein. FGE half‐life was determined after 3 days of treatment with tazarotene/bexarotene 10/20 μM and DMSO controls. All cell lines except HT1080‐FGESer155Pro secreted a truncated form of FGE and the protein half‐life, calculated from intracellular and secreted FGE protein amounts, significantly increased for FGESer155Pro (2‐fold, Fig 7A) and FGE Gly247Arg (1.5‐fold, Fig 7B). The half‐life of FGE Ala279Val showed a trend towards an increase (1.7‐fold, Fig 7C). Half‐life of FGE wild‐type was unchanged upon treatment (Fig EV5B).

## Discussion

MSD is a fatal and untreatable disease. To develop treatment approaches for MSD, we performed a screen of FDA‐approved drugs and discovered the retinoic acid derivative tazarotene to partially restore ARSA activity in MSD patient cells. Subsequent testing revealed that tazarotene in combination with another retinoic acid derivative, bexarotene, partially but significantly restored different lysosomal and nonlysosomal sulfatase activities in MSD patient‐derived fibroblasts, independent of the disease‐causing *SUMF1* mutation. Treatment effects were both dose‐ and time‐dependent. Only cell lines with homozygous null alleles and no FGE expression failed to respond to treatment suggesting an FGE‐mediated mechanism of drug response (see below). Drug treatment reduced pathologic accumulation of total GAGs, reduced lysosomal size, and normalized lysosomal positioning. The treatment effect was also observed in NPCs differentiated from MSD patient‐derived iPSCs, which indicates a molecular mechanism and treatment response across multiple cell types. Taken together, we provide *in vitro* evidence for the first potential small molecule therapy that could translate into a promising strategy for MSD patients *in vivo* given sustained supportive data in subsequent analysis. Nevertheless, the identification of targets, mediating tazarotene and bexarotene response in MSD cells that are amenable to alternative small molecule treatment with more favorable unwanted effects, is desirable.

### Molecular mechanisms of tazarotene and bexarotene in MSD

Tazarotene and bexarotene are both retinoids that bind to the retinoic acid receptors RAR and RXR, respectively. Ligands that bind to RXR receptors are also called rexinoids. For each class of receptor, three subtypes α, β, and γ with multiple isoforms exist. Upon ligand binding, the majority of RARs hetero‐dimerize with RXRs, although the formation of homodimers has also been observed. In its canonical mode of action, RARs and RXRs recruit co‐activating complexes or co‐suppressors and bind to retinoic acid response elements (RARE) in DNA promotor regions thereby regulating transcription. Additionally, RAR/RXR heterodimers activate kinase cascades converging at the nucleus where they regulate transcription through the activation of co‐activators and ‐repressors (di Masi *et al*, 2015).

Among all retinoids tested on MSDi cells, tazarotene was most effective in restoring sulfatase activities. Tazarotene is known to preferentially bind to RAR‐β and ‐γ thereby initiating heterodimerization with RXR receptors. Bexarotene preferably binds to RXR receptors (Miller *et al*, 1997). In MSDi cells, bexarotene treatment response was relatively minor compared with tazarotene treatment and nearly absent in MSD primary fibroblasts, but, interestingly, the combination of tazarotene/bexarotene yielded the highest increase in sulfatase activities. Pretreatment of cells with the pan‐RXR antagonist HX531 did not alter the sulfatase activity response to bexarotene treatment but reduced the tazarotene‐mediated ARSA activity increase in a dose‐dependent manner and the tazarotene/bexarotene response. However, pretreatment with a pan‐RAR antagonist abrogated the response to tazarotene and tazarotene/bexarotene suggesting that RAR receptors are indispensable for mediating drug‐induced sulfatase responses in MSD cells. Based on these results we speculate that sulfatase activation in MSD cells is mediated through RAR/RXR heterodimers. These observations are consistent with previous studies of other systems showing increased efficacy of combination retinoid/rexinoid therapy (Evans & Mangelsdorf, 2014; le Maire *et al*, 2019). However, specific drug‐receptor relationships are complicated by the fact that RAR and RXR ligands could be less receptor‐subtype specific at high concentrations, antagonists could exert agonistic functions, and receptor subtypes show redundancy (di Masi *et al*, 2015). Future research on the retinoic acid response in MSD will be critical, as well as worthwhile because the identification of receptors, and co‐regulators could reveal additional or alternative potential downstream targets for therapeutic intervention.

Transcriptome data from this study and cellular compartment analysis of regulated genes identify mitochondria, vesicles and vesicular, and organelle trafficking. All compartments are closely linked to lysosomal function and pathology (Trivedi *et al*, 2020). In addition, genes known to be related to pathological conditions like Alzheimer's, Parkinson's, and Huntington's disease, indicative of severe cellular pathology, were downregulated in treated MSD fibroblasts (Lloyd‐Evans & Haslett, 2016). Moreover, treatment with tazarotene/bexarotene normalizes the expression of deregulated genes in metabolic and signaling pathways in MSD fibroblasts with the reinstatement of a physiological expression pattern. In summary, the transcriptome analysis reveals improved cellular pathophysiology upon tazarotene/bexarotene treatment in MSD fibroblasts.

The interesting observation that the restoration of sulfatase activities in MSD cells was not general but restricted to a few retinoids was used to analyze differences between the transcriptional responses of a “positive” and a “negative” retinoid. We performed a set of preparatory experiments to identify adapalene as “negative” retinoid. We validated that the compound used is able to enter the cells, binds to RAR and RXR receptors and provokes a transcription response on established targets. However, we did not assess and compare properties like drug stability in cell culture medium, concentrations of drug metabolites in cells, or binding kinetics for the drugs and resulting in an impact on transcriptional response. Cell culture conditions, especially the presence of fetal calf serum and BSA in media, have been analyzed to beneficially influence the stability of retinoids from older pharmacological generations (Klaassen *et al*, 1999). Data for 3^rd^ generation retinoids like tazarotene and bexarotene are missing. Although we cannot exclude that our experimental approach reveals nonphysiological effects, the preparatory measures for selecting the “negative” retinoid and an identical transcription response on known gene targets of retinoids, we propose that the clear differences in transcriptional response between tazarotene and adapalene treatment are physiological effects rather than artifacts. Focussing the transcriptome analysis on genes that are exclusively upregulated by tazarotene such an approach identified genes encoding members of the SREBP‐SCAP‐INSIG complex. The complex's intrinsic function is the transcriptional regulation of sterol and cholesterol synthesis and the sensing of lipid contents in ER membranes (Brown *et al*, 2018). This function, controlled by feedback mechanisms and cellular transcription pathways could be functionally linked to cholesterol storage in MSD cells (Eid *et al*, 2017). Cholesterol storage resulting from impaired digestion and redistribution of membrane contents is a known downstream effect in various lysosomal storage disorders (Platt *et al*, 2012). Details for tazarotene influencing the SREBP‐SCAP‐INSIG complex in the MSD cellular context could be manifold given the orchestrated self‐regulation of the complex (McPherson & Gauthier, 2004) and will be the subject of future analysis. Treatment with fatostatin, an inhibitor of SREBP translocation, revealed blocking of the ARSA activity restoration by tazarotene (see Appendix Supplementary Results) though details are yet to be elucidated in MSD cells. Besides effects indirectly mediated by increased transcription, direct effects of retinoids on mitochondria, autophagy or intracellular signaling pathways have been described: RAR‐β promotes mitochondrial membrane depolarization and transport in neurites through HIF1α (Trigo *et al*, 2019). RARRES1 (retinoic acid response element 1), a primary response protein of tazarotene, induces autophagy in cervical cells through TMEM192, a lysosomal membrane protein (Shyu *et al*, 2016). Bexarotene treatment showed an effect on autophagy and mitophagy in Presenilin 1 deficient iPSC‐derived neural stem cells (Martin‐Maestro *et al*, 2019). Furthermore, retinoic acids have been described to facilitate the mannose‐6‐phosphate receptor‐dependent intracellular trafficking of lysosomal hydrolases (Kang *et al*, 1998). Direct retinoid effects apart from transcriptional control may therefore contribute to an improved cellular pathology in MSD cells.

Upregulated transcription of *SUMF1*, FGE‐interacting proteins, and sulfatases initiated by tazarotene and bexarotene could have been an obvious explanation for downstream drug effects in MSD cells. However, the addition of tazarotene/bexarotene did not alter the transcription levels of these targets. Furthermore, we found no increase in sulfatase protein levels, suggesting that these drugs do not act by increasing the cellular half‐life or intracellular retention of sulfatases. Despite no changes in sulfatase protein amounts, we did detect an increase in sulfatase activities. Such an effect requires FGE function, which is the only protein that is known to activate sulfatases (Cosma *et al*, 2003; Dierks *et al*, 2003). Our observations strongly suggest that tazarotene/bexarotene act to increase the activity of FGE. FGE misfolding due to *SUMF1* mutations affects its stability followed by accelerated degradation. Despite the residual catalytic activity of FGE variants, accelerated degradation results in impaired sulfatase activities (Schlotawa *et al*, 2013). PDI has been identified to play a pivotal role in mediating FGEs intracellular degradation. Decelerated degradation and prolonged half‐life of misfolded FGE results in higher sulfatase activities (Schlotawa *et al*, 2018). Here, treatment with tazarotene and bexarotene increased the half‐life of misfolded FGE, likely enhancing residual FGE activity that partially restores sulfatase activities in MSD cells. This hypothesis is further supported by the observation that tazarotene/bexarotene failed to increase sulfatase activation in cells that lack FGE. Tazarotene/bexarotene‐induced increased FGE variant half‐life could result from several factors including impaired protein degradation, improved protein folding, or both. Interestingly, based on our results, PDI seems not to be a direct target of tazarotene/bexarotene treatment. However, the effects on ER quality control mechanisms like the unfolded protein response or beneficial induction of ER stress (Mollereau *et al*, 2014) could be possible explanations. If existing functional links between ER stress and regulation of lipid metabolism involving the SREBP‐SCAP‐INISG complex (Moncan *et al*, 2021) connect FGE stabilization and regulation of sterol and cholesterol pathway regulation, both induced by tazarotene treatment in MSD cells, need to be elucidated by future experiments.

Because retinoids mediate a plethora of intracellular actions via a multitude of intracellular pathways, our discoveries, yet to be described mechanistically, are not comprehensive. (di Masi *et al*, 2015). Direct effects on FGE and additional indirect retinoid mechanisms would have likely contributed to the restoration of sulfatase activities in combination with the amelioration of cellular pathophysiology in MSD cells. We could demonstrate that cell quantity and cultivation time did not affect ARSA activity in MSD cells and treatment effects were reproducible across different MSD model cell lines and experimental conditions. However, any influence of *in vitro* conditions on cellular mechanisms cannot entirely be ruled out. Future studies, that aim to delineate the molecular mechanism(s) of retinoid treatment in MSD, will be necessary to identify direct targets for alternative therapeutic intervention.

### Potential as a therapy for MSD patients

Tazarotene and bexarotene are both currently used in clinical applications. Bexarotene is approved for the treatment of cutaneous T‐cell lymphoma, while tazarotene is used for topical skin treatment of psoriasis, acne, and photodamage (Duvic *et al*, 2001a; Talpur *et al*, 2009). Tazarotene has successfully passed phase III trials as an oral treatment for psoriasis but has not been approved for this application because of existing treatment alternatives and concerns about possible unwanted side effects intrinsic to the group of retinoids in general (Carlson, 2004).

Reported unwanted side effects include teratogenicity, liver toxicity, hyperlipidaemia, impairment of endocrine, visual and auditory function, and bone changes including mineralization, bone growth, hyperostosis, premature growth plate closure, and ligament calcification (David *et al*, 1988).

Nevertheless, the side effects in the completed phase III trials for oral tazarotene and bexarotene have been reported as mild (Duvic *et al*, 2001b; Weindl *et al*, 2006). None of the drugs has been tested in trials on children. *In vitro*, retinoids could affect cell growth, differentiation, and death (di Masi *et al*, 2015). Although we detected reduced cell proliferation of MSD cells at concentrations increasing sulfatase activities in our experiments, but we did not detect apoptosis rendering tazarotene and bexarotene treatment safe at least *in vitro*.

This study provides several lines of evidence that tazarotene and the combination tazarotene/bexarotene could be beneficial in MSD. Pharmacokinetics from phase I trial for tazarotene and bexarotene in adult probands with advanced cancer showed no toxicity at plasma concentrations that restored sulfatase activities *in vitro* (Miller *et al*, 1997; Jones *et al*, 2003). The active form of tazarotene in the systemic circulation, tazarotenic acid, also increased ARSA activity in MSDi cells. The observed time‐dependent increase in sulfatase activities upon tazarotene/bexarotene treatment in MSD fibroblasts is promising because even sustained low concentrations of tazarotene/bexarotene could result in meaningful clinical improvement in MSD patients. As a notable example, slightly increasing enzyme stability has proven efficacious in another lysosomal storage disorder, Fabry disease. Specifically, a small molecule induced increase in residual activities of alpha‐galactosidase A variants by 3% *in vitro* correlated with meaningful results in Fabry patients (Wu *et al*, 2011; Germain *et al*, 2012). Retinoic acid derivatives are able to cross the blood–brain barrier (Dos Santos Guilherme *et al*, 2019) and tazarotene and bexarotene increased activities of sulfatases in undifferentiated NPCs in a proof‐of‐principle experiment as described above. However, if both drugs would work on neurons and astrocytes *in vitro* and *in vivo* and would be able to reach the central nervous system, the organ system predominantly contributing to the clinical presentation of MSD patients (Ahrens‐Nicklas *et al*, 2018), the evidence needed to be generated that drug concentrations in the CNS will be high enough to result in effective sulfatase activation. Data from healthy subjects treated with standard concentrations of bexarotene revealed a low penetrance of the drug into the CNS and concentrations of only approximately 20 nM (Ghosal *et al*, 2016).

Based on current knowledge more than 95% of MSD patients harbor at least one *SUMF1* missense allele and therefore may benefit from tazarotene/bexarotene treatment, which, according to our data, could act by stabilization of hypomorphic FGE variants (Schlotawa *et al*, 2020). Interestingly, topical tazarotene treatment improved X‐linked ichthyosis, caused by deficiency of steroid sulfatase, activated by FGE, and retinoid treatment increased STS activity via RARα and RXR receptors involving PI3 kinase and ERK‐MAP kinase pathways in myeloid leukemia cells that have been discussed to evolve from FGE activity increase (Hofmann *et al*, 1999; Hughes *et al*, 2006). Although further experimental evidence supporting this hypothesis is missing, it may be possible that the intracellular mechanisms mediating the response to retinoic acid treatment in myeloid leukemia cells are similar to the action of tazarotene and bexarotene in MSD cells (Hughes *et al*, 2006).

While we aimed for identifying licensed drugs through drug screening that could be repurposed to treat MSD time‐ and effort‐efficiently and identified tazarotene and bexarotene, the clinical application of these drugs requires a careful approach. *In vivo* studies should reveal the true potential of tazarotene and bexarotene treatment in MSD. Whereas toxicity data for both tazarotene and bexarotene have been generated as a prerequisite for the licensing of either drug the proof of principle that our *in vitro* data prove true in organisms are yet to be generated. The first step would be preclinical proof in suitable MSD animal models. A *SUMF1* knock‐out mouse model and a *SUMF1* knock‐out zebrafish model are not amenable to treatment as they both lack hypomorph FGE variants but are recently described new MSD mouse models with hypomorphic *SUMF1* variants (Settembre *et al*, 2007; Fleming *et al*, 2022; Sorrentino *et al*, 2022). Assuming a positive outcome of preclinical assessment, treatment of MSD patients with tazarotene and bexarotene and especially its combination would require all phases of clinical research involving MSD patients in childhood. In addition, a new formulation of either drug suitable for children and oral treatment would need to be tested. If either drug would be amenable to drug repurposing would mostly rely on further proof‐of‐principle results.

### Conclusion and future perspectives

In conclusion, the data presented here might be a first step towards the development of a future therapy for MSD. Future *in vivo* studies of tazarotene and bexarotene are needed to evaluate systemic efficacy and overall adverse events or toxicity.

Moreover, this study reveals the hitherto unknown role and molecular mechanisms of retinoids in the pathophysiology of MSD and potentially other related LSDs. The identification of more mechanistic details unraveling alternative treatment targets should be the subject of further research.

## Materials and Methods

### Cell culture

Cell lines used were grown in cell culture as previously described and regularly checked to exclude mycoplasma contamination (Schlotawa *et al*, 2011, 2018). Andrea Ballabio kindly provided MSDi cells. Primary fibroblasts were grown from historical MSD patient samples collected for diagnostic purpose and approved for their use in research projects by the local IRB board (IRB board UMG Goettingen, amendments 3/9/17 and 33/2/21). Please see details on the generation of MSD patient‐derived iPSCs and the origin of ARPE 19 cells further down.

### Arylsulfatase A 96‐well screening assay

We adapted Geng's protocol for high‐throughput screening in MLD for its use in MSD (Geng *et al*, 2011). MSDi cells (*SUMF1* variant c.463C > T, p.Ser155Pro) were plated out in transparent 96‐well plates (Sarstedt, Nürnbrecht, Germany) at a density of 5 × 10^4^ cells in 200 μl cell culture medium per well. In two wells, MSDi cells stably expressing C‐terminally 6‐his tagged wild‐type FGE (MSDi‐FGEHis) were plated out at the same density and served as a positive control for rescued MSD cells (Schlotawa *et al*, 2018). After settling for 2 h 2 μl of each drug from the library (1 mM stocks in DMSO, see above) was added to the cell culture medium (200 μl) towards a final drug concentration of 10 μM and DMSO content of 1%. Controls were treated with DMSO only (see Appendix Fig S1A for the plate design). Cells were incubated for 48 h and then washed twice with Dulbecco PBS (Sigma‐Aldrich Merck, Darmstadt Germany). After complete removal of PBS, 40 μl of lysis buffer (Cell lytic M, Sigma‐Aldrich Merck, Darmstadt Germany + protease inhibitor Roche Complete easypack, Sigma‐Aldrich Merck, Darmstadt Germany) was added, and plates were incubated for 2 h on ice allowing complete cell lysis. After cell lysis 40 μl of substrate buffer (10 mM p‐nitrocatechol sulfate (pNCS, Sigma‐Aldrich Merck, Darmstadt Germany), 0.5 M sodium‐acetate pH 5.0, 0.5 mM sodium‐pyrophosphate, 1.7 M sodium‐chloride) were added to each well except wells A1‐H1 and A12‐D12, which were used for generating an optical density and extinction standard curve. Plates were shaken for 2 h at 300 rpm on an orbital shaker and incubated for 16 h at 37°C, 5% CO^2^, and > 98% humidity. Finally, 120 μl of 1 N NaOH was supplied to each well to stop the enzymatic reaction. A pNC product (Sigma‐Aldrich Merck, Darmstadt Germany) dilution series in substrate buffer (A1, B1: no pNC, C1, D1: pNC 20 μM, E1, F1: pNC 78 μM, G1, H1: pNC 156 μM, A12, B12: 313 μM, C12, D12: 625 μM) was added after supplying 120 μl of 1 N NaOH to prevent pNCS turnover by cell lysates. Plates were centrifuged at 1,160 *g* for 15 min. Supernatant (190 μl) from each well was transferred to a new 96‐well plate without touching the bottom of the original 96‐well plate to avoid suction of remaining cell debris. Air bubbles were manually removed, and the optical density and extinction were analyzed at 515 nm using a plate reader (Synergy Mx, BioTek, Winooski, USA). All pipetting was done using calibrated multi‐channel pipettes (Eppendorf, Hamburg, Germany).

### Drug treatment

LifeArc (London, UK) supplied a screening library in 96‐well plates with 785 licensed drugs dissolved in 100% DMSO at a concentration of 1 mM of each drug (see Dataset EV1 for details on drug library). Hit drugs were identified from the primary screen, and additional drugs were purchased from commercial suppliers (purity ≥ 98%, Appendix Table S1) and used as supplied. All drugs were dissolved in DMSO at 100 or 10 mM stocks according to solubility and stored in aliquots at −80°C until usage. Working concentrations for cellular assays were generated by further dilution with DMSO. The maximum final amount of DMSO in the screening assay was 1%, while all secondary screens and mechanistic studies employed DMSO contents ≤ 0.1%. Drugs in DMSO were applied to the cell culture medium resulting in final concentrations as indicated between 1 and 100 μM. Media supplemented with DMSO alone served as a control treatment. Treatment times varied between a minimum of 24 h and a maximum of 21 days with the renewal of medium and drugs every 3 days and splitting and plating out when cells were confluent.

### Western blotting

MSDi cells and MSD primary fibroblasts were incubated with 10 μM tazarotene, 20 μM bexarotene, or a combination of both for 3 or 6 days. Cells were collected and lysed in ice‐cold lysis buffer containing protease inhibitor (50 mM Tris, 300 mM NaCl, 5 mM EDTA, 1% Triton X‐100, 1% NP‐40, 1% Protease inhibitor mix, Roche, Mannheim, Germany). After clearing by centrifugation (16,000 *g*, 5 min) at 4°C protein concentration was determined by BCA assay (Interchim, Montluçon, France). Total protein amounts between 10 and 40 μg were resolved by SDS–polyacrylamide gel electrophoresis (12% gel for FGE and 10% gel for ARSA, GALNS, and SGSH protein detection), then transferred onto a nitrocellulose membrane (GE Healthcare Life Science, Pittsburgh, USA) and blocked 1 h at room temperature in 5% nonfat milk in TBS‐T (20 mM Tris, 150 mM NaCl, 0.1% Tween 20 (v/v), pH 7.6). Immunodetection was performed by incubation with primary antibodies against ARSA (polyclonal anti‐rabbit, HPA005554, Sigma‐Aldrich, St. Louis, USA; dilution 1:1,000), GALNS (polyclonal anti‐rabbit, PA5‐22098, ThermoFisher Scientific, Waltham, USA; dilution 1:1,000), PARP (monoclonal anti‐mouse, sc‐74,470, Santa Cruz Biotechnology, Dallas, USA; dilution 1:1,000), PARP‐cleaved (monoclonal anti‐rabbit, mAB #5625, Cell Signaling Technology, Danvers, USA; dilution 1:1,000), and beta‐actin (monoclonal anti‐rabbit, mAB #5625, Cell Signaling Technology, Danvers, USA; dilution 1:1,000) followed by incubation with species specific HRP‐conjugated secondary antibodies (goat anti‐rabbit, 111‐035‐003, Jackson Immunoresearch, West Grove, USA; dilution 1:5,000; goat anti‐mouse, 115‐035‐146, Jackson Immunoresearch, West Grove, USA; dilution 1:5,000). Blots were visualized using the Lumi‐Light chemiluminescence detection kit (Roche, Mannheim, Germany) and captured by the chemiluminescence detection system (GE FujiFilm LAS‐4000 Luminescent Image Analyzer, GE Healthcare Life Science, Pittsburgh, USA).

### Lysosomal enzyme activity assays

Arylsulfatase A, arylsulfatase B, galactose‐6‐sulfate sulfatase activity, and sulfamidase activity were determined following previously published protocols (Baum *et al*, 1959; Steckel *et al*, 1983; van Diggelen *et al*, 1990; Karpova *et al*, 1996). For arylsulfatase C activity analysis fibroblasts from a confluent 75 cm^2^ flask were harvested after washing with PBS and lysed in 100 μl ice‐cold NaCl 0.9% plus 0.1% (v/v) Triton X‐100 and sonication (3 × 10 s). After centrifugation in a tabletop centrifuge at 1,000 *g* at 4°C the protein concentration of the supernatant was determined by BCA assay (see above). Supernatant (40 μg), diluted to a final volume of 50 μl in BSA/NaCl/TX buffer (0.2% BSA (m/v), 0.9% NaCl (m/v), 0.1% Triton X‐100 (v/v)), was incubated with 50 μl of substrate buffer (100 mM NaH_2_PO_4_, 1 mM K4‐MUF (7‐hydroxy‐4‐methyl‐coumarin)‐sulfate (Sigma‐Aldrich Merck, Darmstadt, Germany)) pH 8 at 37°C for 18 h in a black 96‐well reaction plate (Greiner bio‐one, Kremsmünster, Austria). Wells with 50 μl substrate buffer and 50 μl substrate buffer plus 3 mM dehydroepiandosteronsulfate and wells with substrate buffer and BSA/NaCl/TX solution without cell lysate served as negative controls. A product dilution series of 4‐methylumbelliferone (4‐MU; Sigma‐Aldrich Merck, Darmstadt, Germany) served as standard. All reactions were stopped by adding 120 μl 0.5 M EDTA pH 11.2–12.0 per well. Readout was done using a fluorescent plate reader (Synergy Mx, BioTek, Winooski, USA) with excitation at 360 nm and emission at 460 nm. For β‐hexosaminidase A + B activity, cells were prepared as described above. Cell lysates (2 μg) were diluted with substrate buffer (0.1 M citrate–phosphate pH 4.5, 2 mM 4‐MU‐2‐acetoamido‐2‐deoxy‐β‐D‐glucoside, Calbiochem Merck, Darmstadt, Germany) to 40 μl final volume in wells of a black 96‐well plate. A standard product dilution series with 4‐MU (Sigma‐Aldrich Merck, Darmstadt, Germany) diluted in H_2_O plus 0.05 M Tris–pH 8.0 was added. After incubation of 30 min at 37°C the reaction was stopped with 150 μl stop buffer (0.17 M glycine‐carbonate), and plates were centrifuged for 15 min at 1,160 *g*. Readout was done using a fluorescent plate reader (Synergy Mx, BioTek, Winooski, USA) with excitation at 360 nm and emission at 460 nm. The same protocol was used for β‐galactosidase activity with a different substrate buffer (0.1 M citrate–phosphate pH 4.5, 2 mM 4‐MU‐β‐D‐galactopyranoside). Activities were determined by referring to changes in OD or fluorescence, respectively, to total protein amounts. For the calculation of specific sulfatase activities, the sulfatase amount in cell lysates was determined in Western blots after quantification of the intensities of specific bands using ImageJ software. Activities were expressed as changes in OD or fluorescence divided by the amount of protein as determined by quantification of Western blots and incubation time.

### Immunofluorescence

Fibroblasts were treated with tazarotene 10 μM and bexarotene 20 μM for 6 days. Cells were plated on cover slips on day 6 in a 24‐well plate (Greiner bio‐one, Kremsmünster, Austria) and allowed to attach for 24 h maintaining treatment conditions. Controls were treated with DMSO only. After washing with PBS, cells were fixed with 4% (v/v) PFA (Süsse, Gudensberg, Germany) in PBS for 20 min at 37°C, washed once again with PBS at 37°C and incubated for 10 min with 50 mM NH_4_Cl. Next, cover slips were washed 3 × 5 min with PBS and incubated for 1 h with 10% horse serum (v/v, Gibco, Carlsbad, USA) and 0.2% saponin (m/v, Sigma‐Aldrich Merck, Darmstadt, Germany) in PBS, followed by two washes with PBS/0.1% saponin (m/v). Cover slips were incubated for 1 h at room temperature with anti‐LAMP1 mouse monoclonal antibody (BD Biosciences, San Jose, USA, 1:500 dilution in PBS/0.1% saponin (m/v)), washed 3× (PBS/0.1% saponin (m/v)), and incubated with Alexa Fluor 488 conjugated goat anti‐mouse secondary antibody (MoBiTec, Göttingen, Germany; 1:1000 in PBS/0.1% saponin (m/v)) for 45 min. Cover slips were finally washed 3× (PBS/0.1% saponin) and 2× with PBS and mounted on slides with prolonged gold mountant +/− DAPI (Invitrogen, Carlsbad, USA). Fluorescence microscopy was performed using a Zeiss Definite Focus.2 confocal inverted microscope (Zeiss, Göttingen, Germany). Images were taken with a Plan‐APOCHROMAT 63 × 1.4 numerical aperture oil‐immersion objective (Zeiss, Göttingen, Germany) using the ApoTome system. For each image, 13 z‐series optical sections were collected with a step size of 0.26 μm. Z‐series are displayed as maximum z‐projections, and brightness and contrast were adjusted identically for each image set using ZEN Pro software (Zeiss, Göttingen, Germany). The total fluorescence intensity of the cells and the size of the LAMP1‐positive particles were analyzed from a minimum of 20 cells for each treatment (*n* = 3 independent experiments (biological replicates)).

### Glycosaminoglycan quantification

Glycosaminoglycan analysis was performed by adapting a protocol established by Fuller *et al* (2004). MSD primary fibroblasts and control fibroblasts were grown in T75 cell culture flasks (CellStar, Greiner bio‐one, Kremsmünster, Austria) with tazarotene/bexarotene 10/20 μM or DMSO as the control for 21 days. Cells from confluent flasks were harvested, and protein concentration was measured by BCA assay after lysis of 1/5^th^ of the cells. 4/5^th^ of the cells were frozen at −20°C and stored until further processing.

After thawing, cell pellets were resuspended in 50 μl PBS per 120 μg total protein. Fifty microliter of each sample was dried using a centrifugal concentrator under vacuum and reconstituted in 100 μl of 0.25 M PMP solution (0.25 M 1‐phenyl‐3‐methyl‐5‐pyrazolone (PMP)) in 0.4 M ammonia solution (11.95 ml of MeOH and 2.59 ml of ammonium hydroxide (28–30% ammonia) added to 35.5 ml MilliQ water (pH 9.5–10) containing 1 μM of internal standard (chondroitin disaccharide di‐4 S [CAS 136144‐56‐4], Carbosynth Ref: OC28898)). Samples were vortexed, sonicated, and mixed prior to 90 min incubation on a PCR thermocycler at 70°C and cooling for 10 min. Samples were acidified with 500 μl of 0.2 M formic acid, and PMP was extracted from the acidified samples by adding 500 μl chloroform and shaking for 1 min. Samples were centrifuged for 5 min at 13,000 *g* to separate the layers and the bottom organic layer was discarded. The procedure was repeated four times for each sample to completely remove PMP. The remaining aqueous layer (600 μl for each sample) was concentrated to 80 μl using a centrifugal concentrator under vacuum. After centrifugation for an additional 5 min at 13,000 *g* the supernatant (at least 60 μl) of every sample was referred to LC–MS/MS analysis on an Agilent UPLC system (Agilent Pursuit 3 PFP 2.0 ×100 mm 3 μm Column (Agilent, Santa Clara, USA)) and AB Sciex 6500 TQ Mass Spec System (Sciex, Framingham, USA).

### Cell proliferation analysis

MSDi cells and MSD primary fibroblasts were seeded at a concentration of 3,000 and 2,000 cells/well, respectively, in 96‐well microplates and allowed to attach for 24 h. Cells were incubated with 10 μM tazarotene, 20 μM bexarotene, or a combination of both for 3 days (MSDi) or 6 days (fibroblasts). Cell proliferation during incubation was determined by XTT [sodium 3′‐[1‐(phenylaminocarbonyl)‐3,4‐tetrazolium]‐bis (4‐methoxy‐6‐nitro) benzene sulfonic acid hydrate] assay according to the manufacturer's protocol (AppliChem, Darmstadt, Germany). Absorbance was measured at 450 nm (reference wavelength 650 nm). Proliferation was expressed as a percentage of control cells treated with DMSO. Manual counts of cells were performed after trypsinization of cells, centrifugation, and resuspension of cell pellets in 5 ml PBS. Ten microliter of the suspension was pipetted onto a Neubauer counting chamber followed by manual cell counting using a light microscope.

### iPSC generation

The generation of an iPSC line from a MSD patient was done following a previously published protocol (Maguire *et al*, 2016). In brief, blood was collected from a patient with MSD harboring compound heterozygote *SUMF1* variants (c.463T > C, p.Ser155Pro/c.1034G > A, p.Arg345His). This research was approved by the Institutional Review Board at the Children's Hospital of Philadelphia (IRB #09‐00742) Cellular reprogramming was performed using ficoll‐purified mononuclear cells from whole blood that were expanded for transduction with Sendai viral vectors expressing human OCT3/4, SOX2, KLF4, and cMYC according to the manufacturer's instructions (ThermoFisher Scientific). Transduced cells were plated on culture dishes containing murine embryonic fibroblasts (MEFs) and maintained in a medium containing 10 ng/ml bFGF. The medium was replenished daily for 3 weeks. Cells were maintained in these conditions until uniform colonies were generated and colonies were mechanically isolated for expansion on MEFs. Single colony subcloning was performed at early passages and tested for clearance of the Sendai reprogramming vectors using real‐time RT–PCR (Appendix Fig S7A). The authentication of each clone confirming identity to the original patient cells was performed by DNA fingerprinting using PCR (Appendix Fig S7B). Mutation verification was also performed on genomic DNA by PCR amplification and sequence analyses (Appendix Fig S7C). Karyotype analysis was performed by Cell Line Genetics (Madison, WI). Stemness surface markers were performed by flow cytometry and mycoplasma was tested by PCR (Appendix Fig S7D and E).

### Differentiation of iPSCs into neural progenitor cells

Differentiation of iPSCs into NPCs was initiated, as previously described (Maguire *et al*, 2016) with indicated modification. Briefly, cultures were treated with daily media changes containing SB431542 (10 μM; Tocris), LDN193189 (1 μM, Tocris), and *endo*‐IWR1 (1.5 μM; Tocris) and supplemented with B27 without vitamin A (Invitrogen) and passaged at days 4 and 8 of differentiation. From days 8 to 14 of differentiation, NPCs were expanded in Invitrogen neural expansion media, containing Neural Induction Supplement in Advanced DMEM/F12 and Neurobasal Medium (Invitrogen, per manufacturer instructions). On Day 14, NPCs were cryopreserved after confirmation of NPC identity with > 90% expression of Forse‐1.

### NPC drug treatment

For drug treatments, NPCs were retrieved into neural expansion media containing 5 μM Y‐27632 (Tocris), followed by 4 days of daily media changes containing tazarotene (5 μM) and bexarotene (5 μM) or the equivalent concentration of DMSO (0.01%) in neural expansion media. NPCs were subsequently harvested using accutase, washed with PBS, pelleted, and frozen at −80°C until analysis. All conditions were performed in triplicate.

### ARPE19 *SUMF1* ^−/−^ cell line generation

ARPE19 (ATCC, Manassas, USA, Cat. No. CRL‐2302) cells were referred to CRISPR/Cas9‐mediated knock‐out of the *SUMF1* gene to generate the ARPE19 SUMF1^−/−^ cell line. The gRNA sequence was determined by using the CRISPOR online tool (http://crispor.tefor.net/crispor.py; Concordet & Haeussler, 2018) and selected based on the lowest off‐target score. The gRNA with the 5′‐3′ sequence CCCTTGCGGGTTCTTGCGGCTGC was used in an “all‐in‐one” vector additionally encoding Cas9 linked to green‐fluorescent protein (Cas9‐GFP) (Sigma‐Aldrich, St. Louis, USA). Plasmid‐DNA was electroporated into ARPE19 cells using the Amaxa system and a nucleofection kit (Lonza, Basel, Switzerland, Cat. No. VCA‐1003) following the manufacturers' instructions. GFP‐positive cells were sorted by fluorescent‐activated‐cell‐sorting (FACS) into 96‐well plates. Single‐cell derived colonies were screened for deletion mutations in the *SUMF1* gene after extraction of genomic DNA, amplification of the target region by PCR (forward primer hSUMF1KOup, 5′‐3′‐sequence: cagcgccaaagaagtacctg, reverse primer hSUMF1KOlow, 5′‐3′‐sequence: tcggaggaatcgatggagc), followed by Sanger sequencing using the same primers. A cell clone carrying a homozygous deletion in the *SUMF1* gene (c.139delCG, p.Ala47GlyfsTer74) leading to a premature stop codon was selected and expanded. Cells of the respective clone were subjected to cell lysis, protein estimation, and Western Blot analysis as described above using a SUMF1 antibody (R&D Systems, Minneapolis, USA, Cat No AF3680) to verify absent FGE protein expression (Appendix Fig S12).

### cDNA synthesis and RT–PCR

Total RNA was isolated using NucleoSpin RNA preparation kit (Macherey‐Nagel, Düren, Germany). After quality control by optical density (OD) measurement, 1 or 2 μg of RNA was reverse transcribed using SuperScript III First‐strand Synthesis system for RT–PCR (Invitrogen, Karlsruhe, Germany) according to the manufacturer's instruction. Real‐Time PCR analysis for *CYP26B1*, *RARB*, *RARRES1*, *RARRES2*, and *RARRES3* was performed using Quant Studio 3 Real‐Time PCR System (ThermoFisher Scientific, Braunschweig, Germany) according to the manufacturer's recommendations in reactions containing 20 ng cDNA. The gene expression of targeted genes was normalized to the housekeeping gene as indicated in the figure legends. Primer sequences are given in Appendix Table S2. Relative gene expression was analyzed by QuantStudio Design and Analysis software v1.4.3 (ThermoFisher Scientific, Braunschweig, Germany) and quantified using the ΔΔ*C*_t_ method.

### Transcriptome and pathway analysis

For our first experiment (comparison of tazarotene/bexarotene treatment to untreated condition), six different MSD fibroblast lines and five control fibroblast lines were treated with DMSO or tazarotene/bexarotene 10/20 μM for 6 days in triplicates. Cells were harvested and processed for RNA isolation and RNA sequencing as described (Martinez Hernandez *et al*, 2018). cDNA libraries were established using a TrueSeq Stranded Total RNA library kit (#20020596, Illumina) and sequenced using a Illumina HiSeq 2000. For expression analysis, reads were mapped to the human genome (hg38) using STAR aligner (v.2.7.3a). Mapped reads were sorted and indexed with SAMtools (v.1.10) and gene counts were generated with Featurecounts (v.1.5.1). Low‐quality samples were removed from the analyses. Sequencing data from the first experiment was analyzed using co‐expression analysis, given our interest in finding clusters of genes that had similar correlation patterns. In specific, the so‐called WGCNA (weighted‐gene co‐expression analysis) was performed with the homonymous R‐package (v.1.68) and applying the following steps: (i) the analysis was done on normalized expression values obtained with DESeq2 (v.1.68). (ii) genes were filtered out from the analysis if they did not have, on average, at least 20 normalized counts per sample. (iii) the power parameter of the network topology was estimated with the function “pickSoftThreshold.” Finally, gene clusters were inferred with the function “blockwiseModules” with parameters “maxBlockSiz=7000, power=14, minModuleSize=60, and mergeCutHeight=0.25.” Gene ontology enrichment analysis on resulting clusters was performed with the ShinyGO online webtool (v.0.61) hosted by the University of South Dakota, US (http://bioinformatics.sdstate.edu/go/).

For our second experiment (comparison of tazarotene to adapalene treatment), seven different MSD fibroblast lines were treated with tazarotene 10 μM or adapalene 5 μM, respectively, for 6 days in triplicates. DMSO‐treated cells served as a control condition. Sequencing data from the second experiment were analyzed using a differential gene expression (DGE) approach and for such purpose, the aforementioned DESeq2 package was used. Samples were quality controlled using PCA analysis on gene count data, with specific gene counts themselves filtered out if they had less than 30 reads on average across all samples. The resulting count matrix was normalized and used as input for DGE. The design function took into account that samples came from seven different cell lines in triplicates. Tazarotene and adapalene treatments were compared using the contrast function embedded within the DESeq2 package.

### PDI expression and STS‐specific activity assay

PDI expression in MSDi cells and inducible expression of STS and FGE for the determination of STS‐specific activity by western blots and STS‐activity assays were done as described before (Schlotawa *et al*, 2018) following tazarotene/bexarotene treatment for a total time of 3 days.

### Pulse‐Chase experiments

HT1080 cell lines stably expressing FGE variants Ser155Pro, Gly247Arg and Ala279Val were either treated with 10 μM tazarotene and 20 μM bexarotene or DMSO (as control) for 3 days prior to the start of pulse‐chase experiments. Of note, the presence of the drug or DMSO was maintained in all supplemented media throughout the experiment. After starving for 1 h in a medium depleted of methionine and cysteine, the cells were pulsed with ^35^S‐methionine/cysteine (Hartmann Analytic) for 30 min. Cells and media were collected after incubation for various time points in unlabeled medium (chase). Cell lysis, FGE immunoprecipitation from cell lysate and media, SDS–PAGE and autoradiography, and image analysis using ImageJ software were as previously described (Schlotawa *et al*, 2018).

### Image quantification

Western Blots and immunofluorescence images were not blinded quantitatively analyzed using Fiji software (Schindelin *et al*, 2012). Statistical analysis was performed using Prism (GraphPad software, San Diego, USA).

### Statistical analysis

Results were calculated from a minimum of three independent experiments (biological replicates), and replicate measurements were summarized as mean values in respective calculations. Exemptions in the number of experiments are indicated in respective figure captions. All statistical analysis was performed using Prism (GraphPad software, San Diego, USA). Comparison of two independent experimental conditions was done using an unpaired *t*‐test, and paired *t*‐tests were used for dependent variables. Comparison of multiple experimental conditions (> 2) was executed by one‐way ANOVA tests followed by the Tukey's multiple comparison test according to the program's settings. Two‐way ANOVA followed by the Tukey's multiple comparison test was used when results depended on two parameters (treatment vs. untreated and time in pulse‐chase experiments). For nonlinear regression calculation, drug concentrations were transformed to log10, manually referring activity responses to DMSO treatment as log −2 concentrations, and calculated using the program's predefined settings. Data were expressed and displayed as mean and standard deviation. Significance levels were displayed as follows: *P < 0.05, **P > 0.01, ***P < 0.001, ****P < 0.0001 or # *P* < 0.05, ## *P* < 0.01, ### *P* < 0.001, #### *P* < 0.0001, respectively, for differences indicated in the figure legends. Details on *P*‐values of significant differences only for all figures are summarized in Appendix Tables S3–S46.

## Author contributions

**Lars Schlotawa:** Conceptualization; data curation; formal analysis; supervision; validation; investigation; visualization; methodology; writing – original draft; project administration; writing – review and editing. **Karolina Tyka:** Conceptualization; data curation; formal analysis; supervision; validation; investigation; visualization; methodology; writing – review and editing. **Matthias Kettwig:** Conceptualization; data curation; formal analysis; supervision; validation; investigation; visualization; methodology; writing – review and editing. **Rebecca C Ahrens‐Nicklas:** Investigation. **Matthias Baud:** Conceptualization; validation; writing – review and editing. **Tea Berulava:** Data curation; formal analysis; validation; investigation; visualization; methodology; writing – original draft. **Nicola Brunetti‐Pierri:** Validation; investigation; writing – review and editing. **Alyssa Gagne:** Validation; methodology; writing – review and editing. **Zackary M Herbst:** Data curation; formal analysis; validation; investigation; methodology; writing – original draft; writing – review and editing. **Jean A Maguire:** Data curation; formal analysis; validation; investigation; methodology; writing – review and editing. **Jlenia Monfregola:** Resources; validation; methodology; writing – original draft; writing – review and editing. **Tonatiuh Pena:** Data curation; formal analysis; validation; investigation; visualization; methodology; writing – original draft; writing – review and editing. **Karthikeyan Radhakrishnan:** Conceptualization; data curation; formal analysis; supervision; validation; investigation; visualization; methodology; writing – review and editing. **Sophie Schröder:** Data curation; formal analysis; investigation; visualization; writing – review and editing. **Elisa A Waxman:** Data curation; formal analysis; validation; investigation; visualization; methodology; writing – original draft. **Andrea Ballabio:** Resources; supervision; validation; methodology; writing – review and editing. **Thomas Dierks:** Resources; supervision; validation. **André Fischer:** Supervision; validation; investigation; visualization; methodology; writing – original draft; writing – review and editing. **Deborah L French:** Supervision; investigation; methodology; writing – review and editing. **Michael H Gelb:** Supervision; validation; investigation; methodology; writing – review and editing. **Jutta Gärtner:** Conceptualization; formal analysis; supervision; funding acquisition; validation; methodology; writing – original draft; project administration; writing – review and editing.

## Disclosure and competing interests statement

LS, MK, JG, TD, KR, and MB have filed a patent application based on parts of the results from this study (US patent application number 16591051). AB is a co‐founder of CASMA Therapeutics and Advisory Board member of Avilar Therapeutics and of Next Generation Diagnostics. MHG is a consultant for PerkinElmer Corp. and a co‐founder of GelbChem LLC. ZMH is a consultant for GelbChem LLC. All other authors declare that they have no conflict of interest.

## For more information

MSD Action Foundation: www.savingdylan.com; United MSD Foundation: https://curemsd.org

## Supporting information



Appendix

Expanded View Figures PDF

Table EV1

Dataset EV1

Source Data for Expanded View and Appendix

PDF+

Source Data for Figure 1

Source Data for Figure 2

Source Data for Figure 3

Source Data for Figure 4

Source Data for Figure 5

Source Data for Figure 6

Source Data for Figure 7

## Data Availability

RNAseq data are available via the GEO database (GEO accession GSE205555 and GSE205556, https://www.ncbi.nlm.nih.gov/geo/).
