# Supplementary material for: Drug screening identifies tazarotene and bexarotene as therapeutic agents in multiple sulfatase deficiency
Source: EMBO Mol Med. 2023 Feb 15;15(3):e14837. doi: 10.15252/emmm.202114837 (PMC9994482; doi:10.15252/emmm.202114837)
Supplement: Supplementary file 1 — Appendix [file EMMM-15-e14837-s007.pdf]

**Appendix**

**Content:**

Appendix supplementary results, pages 1-6

Appendix supplementary figures, pages 7-19

Appendix supplementary figure legends, pages 20-28

Appendix supplementary tables, pages 29-67

**Appendix supplementary results**

**Effects of tazarotene and bexarotene treatment on cell proliferation in MSD and control fibroblasts**

MSD fibroblasts (p.Gly247Arg) and control fibroblasts were plated out at day 0 at standard cell quantity ($9x10^5$, medium) and additional quantities ($4.5x10^5$, low; $13.5x10^5$, high) and grown in presence of 0.1 % DMSO (v/v). Cells were harvested on day 3, 6, and 9, and analysed for cell count, total protein amount and ARSA activity in order to measure effects of cell density on all parameters. In parallel, MSD fibroblasts (p.Gly247Arg) and control fibroblasts were grown under standard conditions ($9x10^5$ at day 0) treated with 10 μM tazarotene, 20 μM bexarotene, and 10/20 μM tazarotene/bexarotene, respectively. Cells were harvested at the same time points for analysis of the same parameters.

We detected a time dependent significant increase of cell quantity in DMSO only treated MSD fibroblasts compared to day 0 independent of the starting cell quantity. Medium and high cell quantities led to a plateauing increase of cell quantity from day 3 onwards. Cell counts at day 9 were not significantly different in DMSO treated MSD fibroblasts (Appendix Fig. S5A, Appendix Table S3). Control fibroblasts also showed a time dependent significant increase of cell quantity in DMSO only treated independent of the starting cell quantity. The increase plateaued from day 3 onwards for DMSO treated cells plated at medium and high

quantity whereas low quantity cells showed persistent increase of cell number until day 9. Cell counts at day 9 were not significantly different in DMSO treated control fibroblasts (Appendix Fig. S5B, Appendix Table S4) Please note that MSD fibroblasts grew faster than control fibroblasts (Appendix Fig. S5A,B; Appendix Table S3,S4). Treatment with tazarotene, bexarotene and tazarotene/bexarotene, respectively, significantly influenced cell count in both lines with strongest effects in tazarotene/bexarotene treated cells (MSD fibroblasts day 9, DMSO medium: 37.8 x $10^5$ cells, Taz: 24.9 x $10^5$ cells, Bex: 26.3 x $10^5$ cells, Taz/Bex: 20.1x10x105 cells).

Total protein also increased over time in MSD and control fibroblasts under DMSO conditions independent of cell quantity at start. There were no significant differences in total protein amount in DMSO treated MSD fibroblasts at day 6 and 9 and no differences for control fibroblasts from day 3-9 (Appendix Fig. S5C,D; Appendix Table S5). However, under treatment conditions, total protein amounts were significantly reduced in control fibroblasts treated with tazarotene or tazarotene/bexarotene at day 6 and at day 9 compared to DMSO treated cells plated at medium quantity (Appendix Fig. S5D, Appendix Table S6). Interestingly, protein amounts in treated MSD fibroblasts were only significantly reduced at day 6 when treated with tazarotene/bexarotene and compared to DMSO treated cells plated at medium and high quantity. This reduction resolved over time and neither treatment showed significant differences in total protein amount in MSD fibroblasts compared to for DMSO treated cells plated at medium and high quantity (Appendix Fig. S5C, Appendix Table S5).

ARSA activity did not show a time and cell-quantity dependent increase in MSD fibroblasts under DMSO only conditions (Appendix Fig. S5E, Appendix Table S7). However, there was a significant increase of ARSA activity starting at day 3 for tazarotene/bexarotene treated cells compared to DMSO conditions. From day 6 onwards tazarotene treatment led to a significant increase of ARSA activity compared to DMSO conditions. The highest increase

for tazarotene treated cells was 4.5-fold (day 9) and 7.8-fold for tazarotene/bexarotene (day 9), significantly higher than tazarotene treated cells (Appendix Fig. S5E, Appendix Table S7). Interestingly, endogenous ARSA activity was significantly increased at day 6 and 9 between DMSO only treated control fibroblasts plated at low quantity compared to for DMSO treated cells plated at medium and high quantity DMSO medium and high conditions (maximum 1.8-fold increase). Treatment with tazarotene, bexarotene or tazarotene/bexarotene led to no further increase compared to for DMSO treated cells plated at medium quantity conditions (Appendix Fig. S5F, Appendix Table S8).

To validate cell proliferation with a method that does not rely on maunual cell counting, we performed XTT assays in control and MSD fibroblasts. Of note, xtt assays are dependent on cellular metabolism, which in turn is influenced by cell count, size and protein amount. 6-day treatment with tazarotene, bexarotene, and tazarotene/bexarotene resulted in reduced cell proliferation of control fibroblasts when compared to cells treated with DMSO only. Changes in treated MSD fibroblasts were not significantly different (Appendix Fig. S6 A,C). Treatment with different concentrations of tazarotene/bexarotene for 6 days showed significantly reduced cell proliferation compared to DMSO treatment starting at 1/2 μM tazarotene/bexarotene in control fibroblasts (minimal proliferation 65% of DMSO control at 5/10 μM tazarotene/bexarotene). Again, no significant differences appeared in MSD primary fibroblasts (Appendix Fig. S6 B,D).

**Adapalene increases transcription of retinoid target genes via RAR receptors without increasing sulfatase activities in MSD primary fibroblast cell lines**

To identify a retinoid that worked on MSD cells but did not increase sulfatase activities we treated primary MSD fibroblasts (p.Gly247Arg) with a selection of retinoids representing generations of pharmacological development (1st generation tretinoin, 2nd generation

acitretin, 3rd generation adapalene) for six days at concentrations of 10 µM (adapalene 5 µM) and analysed three different sulfatase activities (ARSA, GALNS, STS). Tazarotene treatment (10 µM) served as positive control, DMSO treated cells as negative control. Neither retinoid was able to increase ARSA, GALNS, or STS activities (Appendix Fig. S7A). Next, we used the same retinoids and treatment conditions to analyse gene expression of retinoid targets by RT-PCR to prove that the selected retinoids entered treated cells and were effective without increasing sulfatase activities. Tretinoin, acitretein, and adapalene were all able to increase *RARB*, *CYP26B1*, and *RARRES1-3* gene expression compared to untreated controls. Significant differences of gene expression were seen for *CYP26B1* and *RARRES3* that was highest upon tazarotene treatment. *RARB*, *RARRES1* and *RARRES2* showed increased but no significantly different gene expression regardless of the retinoid used (Appendix Fig. S7B). Based on the results we chose to use adapalene, which belongs to the same pharmacological group of retinoid development like tazarotene and bexarotene (3rd generation retinoids), as a control retinoid for further experiments. Next, we wanted to rule out that we have missed the right dose for an effect on sulfatase activities in MSD fibroblasts and treated cells with four different concentrations of adapalene (1, 5, 10, 20 µM) for six days. Again, tazarotene treatment served as control. Neither ARSA nor GALNS activity increase could be detected at either concentration used (appendix figure S8A). Gene expression analysis of retinoid targets upon treatment with increasing concentrations showed increased expression levels at all four adapalene concentrations compared to DMSO conditions but significantly reduced expression levels at 20 µM concentration compared to lower concentrations likely because of visual adapalene toxicity on cells. *RARRES3* showed a trend towards differences only (Appendix Fig. S8B). Based on these results we chose a submaximal concentration of adapalene (5µM) for further experiments to avoid toxicity. Treatment of eight different MSD fibroblast lines with adapalene showed no ARSA activity increase regardless of the *SUMF1* mutation (Appendix Fig. S8C thereby ruling out mutation specific differences. A refined dose-

concentration analysis with adapalene treatment of MSD fibroblasts with concentrations starting as low as 100 nM and up tp 20 µM for six days still excluded any ARSA activity increase (Appendix Fig. S8D).Finally, we pretreated cells for 24 hours with the pan-RAR antagonist AGN193109 followed by 72 hours of simultaneous treatment with adapalene (control: tazarotene). Incubation with AGN193109 abrogated both adapalene and tazarotene induced retinoid target gene expression in MSD fibroblasts (Appendix Fig. S9) thereby proving that adapalene, like tazarotene, increased retinoid target gene expression in MSD fibroblasts via RAR receptors.

**Tazarotene/bexarotene treatment did not change endogenous PDI protein expression but decreased PDI-mediated inhibition of FGE variants residual activity**

PDI has been described as a pivotal interacting partner of FGE-variants with impact on residual FGE and dependent sulfatase activities by binding to misfolded FGE variants and referring them to early degradation (Schlotawa *et al*, 2018). In order to see if tazarotene/bexarotene treatment reduces PDI expression resulting in less FGE variant binding and increased FGE activity we analysed three different MSD primary fibroblast lines treated with either DMSO or tazarotene/bexarotene treatment for six days and one control fibroblast line by western blot. No differences could be detected thereby ruling out an effect of tazarotene/bexarotene on PDI expression (Appendix Fig. S12).

To further analyse how PDI interaction with FGE variants changes upon tazarotene/bexarotene treatment we repeated an experiment from our previous publication (Schlotawa *et al.*, 2018). We were aiming to see differences on the activation of steroid sulfatase (STS) by FGE-Ser155Pro variant with and without PDI co-expression and treatment of cells with either DMSO (vehicle control) or tazarotene/bexarotene (Appendix Fig. S13A,B). As compared to DMSO treated cells, tazarotene/bexarotene consistently led to an

increase in STS activity with and without FGE or FGE-variant co-expression. When compared to respective DMSO controls, in FGE-Ser155Pro alone expressing cells, tazarotene/bexarotene treatment led to an approx. 4.5-fold increase in activity while co-expression of PDI in drug treated cells led to a 7.5-fold increase (Appendix Fig. S13C). However, note that, this apparent increase in activity upon PDI co-expression in drug treated cells is due to the decrease in activity of FGE-Ser155Pro when PDI is co-expressed in DMSO treated cells. In DMSO treated cells, co-expression of PDI and FGE-Ser155Pro led to a 3-fold loss of Ser155Pro variants activity, in agreement to our previous work. Such a loss in activity is also observed in drug treated cells, however, only to 2-fold (Appendix Figure S13D). This indicates that co-expression of PDI has a minor effect on prohibiting FGE-Ser155Pro induced STS activation when cells are treated with tazarotene/bexarotene compared to DMSO treated cells. Supported by our data that tazarotene/bexarotene treatment improves the intracellular stability of FGE variants (Fig. 4), we think that it is plausible that tazarotene/bexarotene treatment reduces the proportion of misfolded FGE variants that bind to PDI and increase the proportion able to escape PDI recognition due to improved folding. This results in increased FGE functionality and the observed increase in sulfatase activities upon tazarotene/bexarotene treatment.

**Appendix supplementary figures**

Appendix figure S1

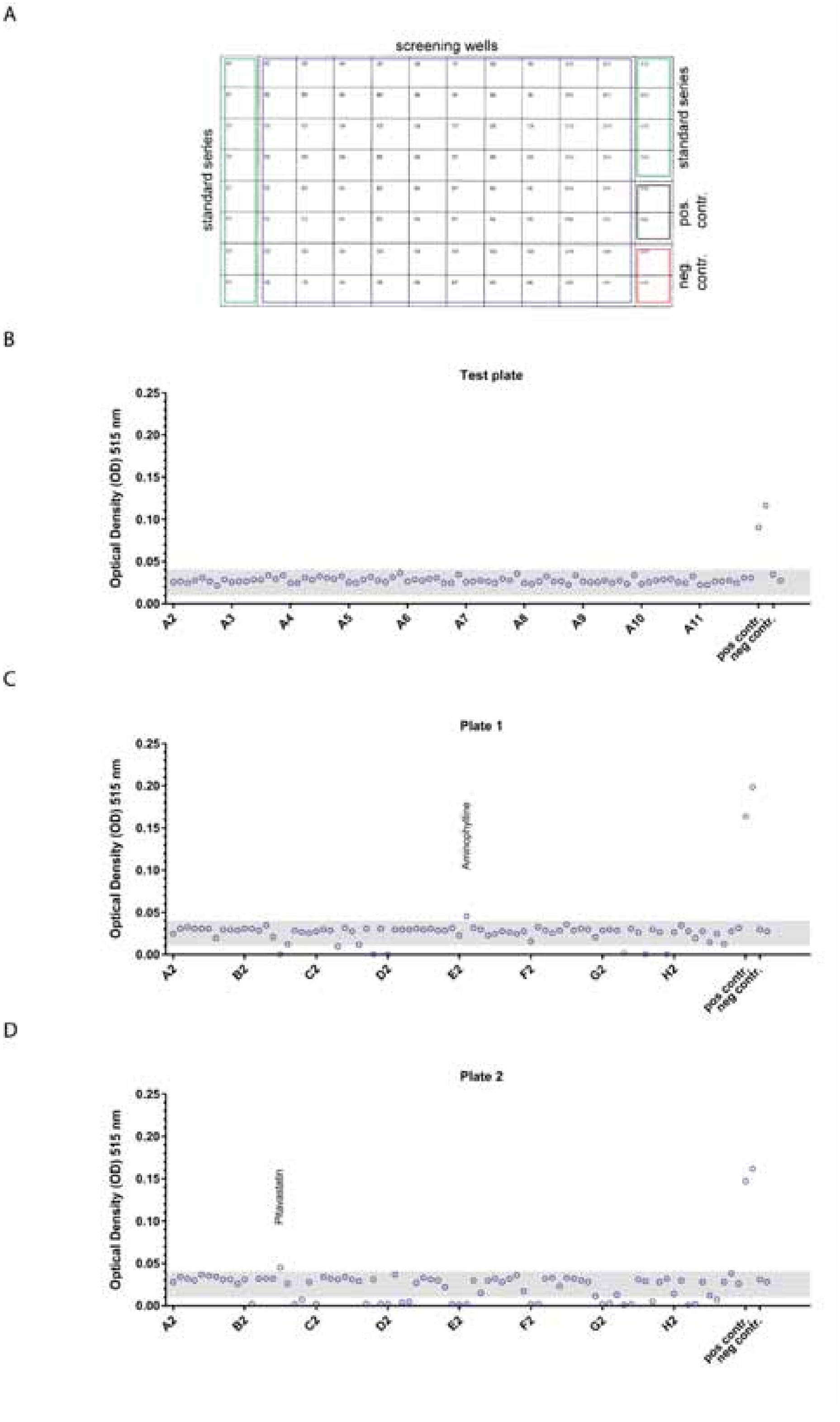

Appendix figure S2

A

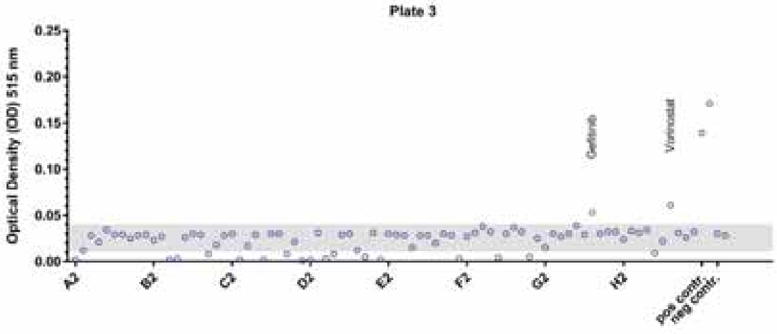


B

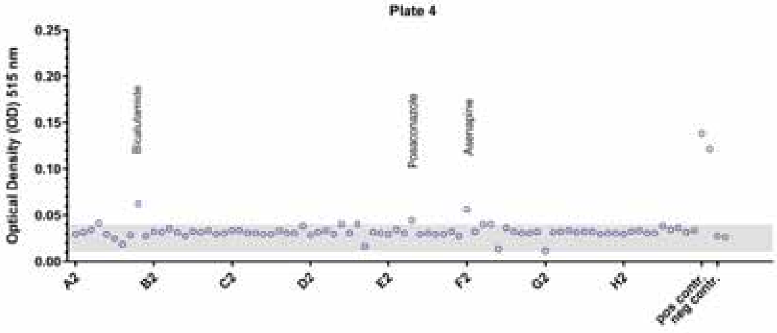


C

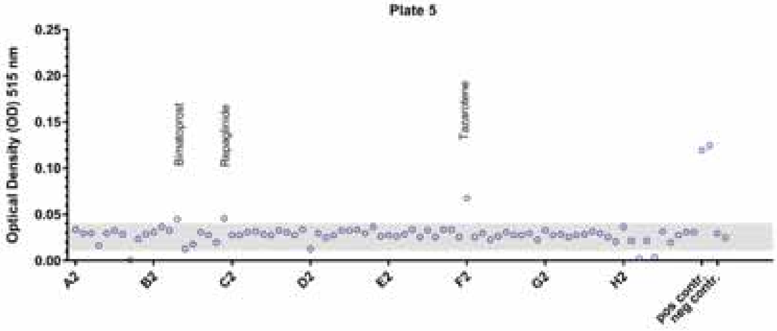


D

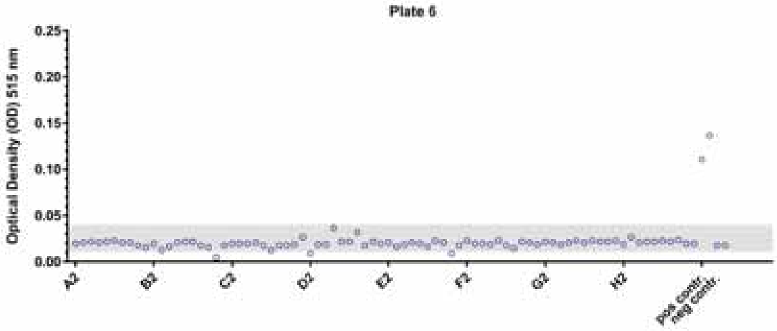

Appendix figure S3

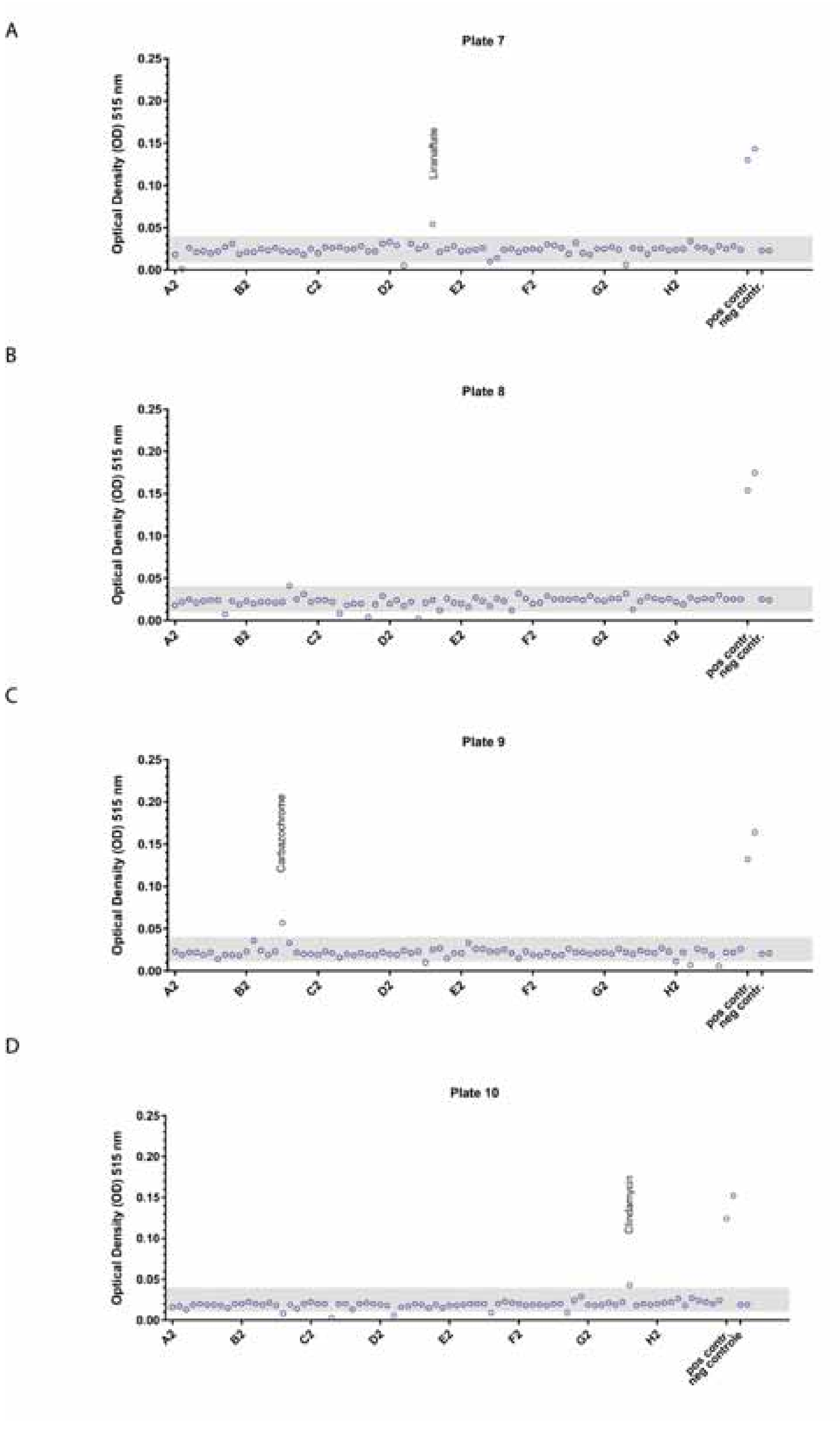

Appendix figure S4

A

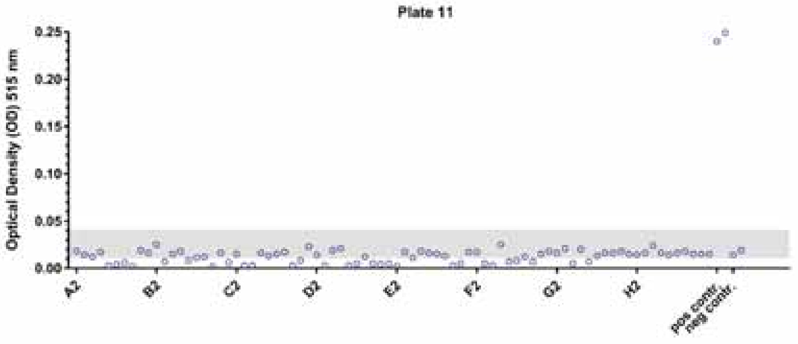


B

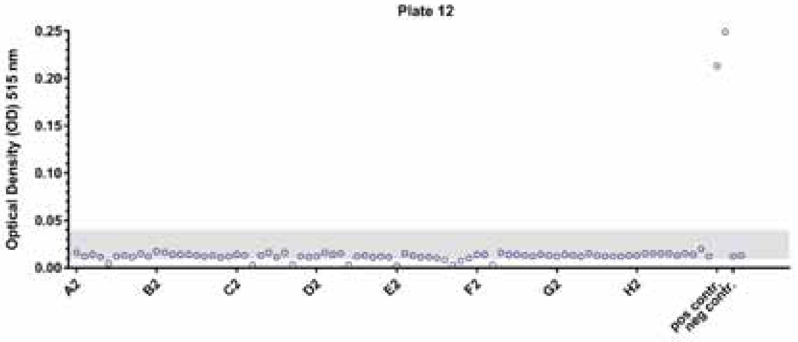


C

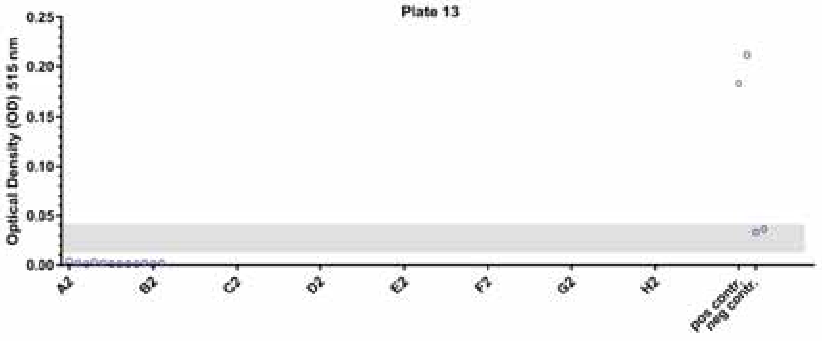

Appendix figure S5

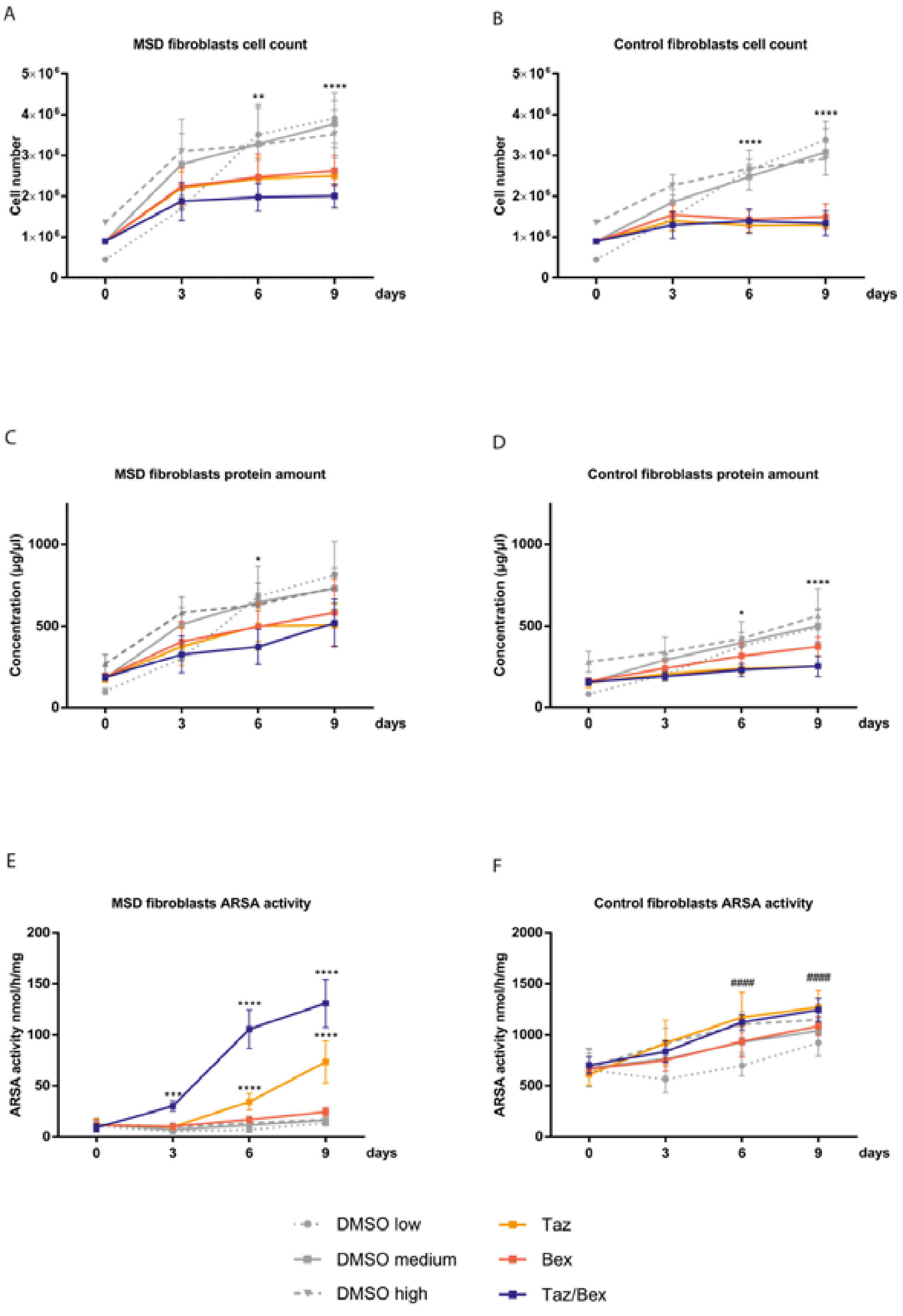

Appendix figure S6

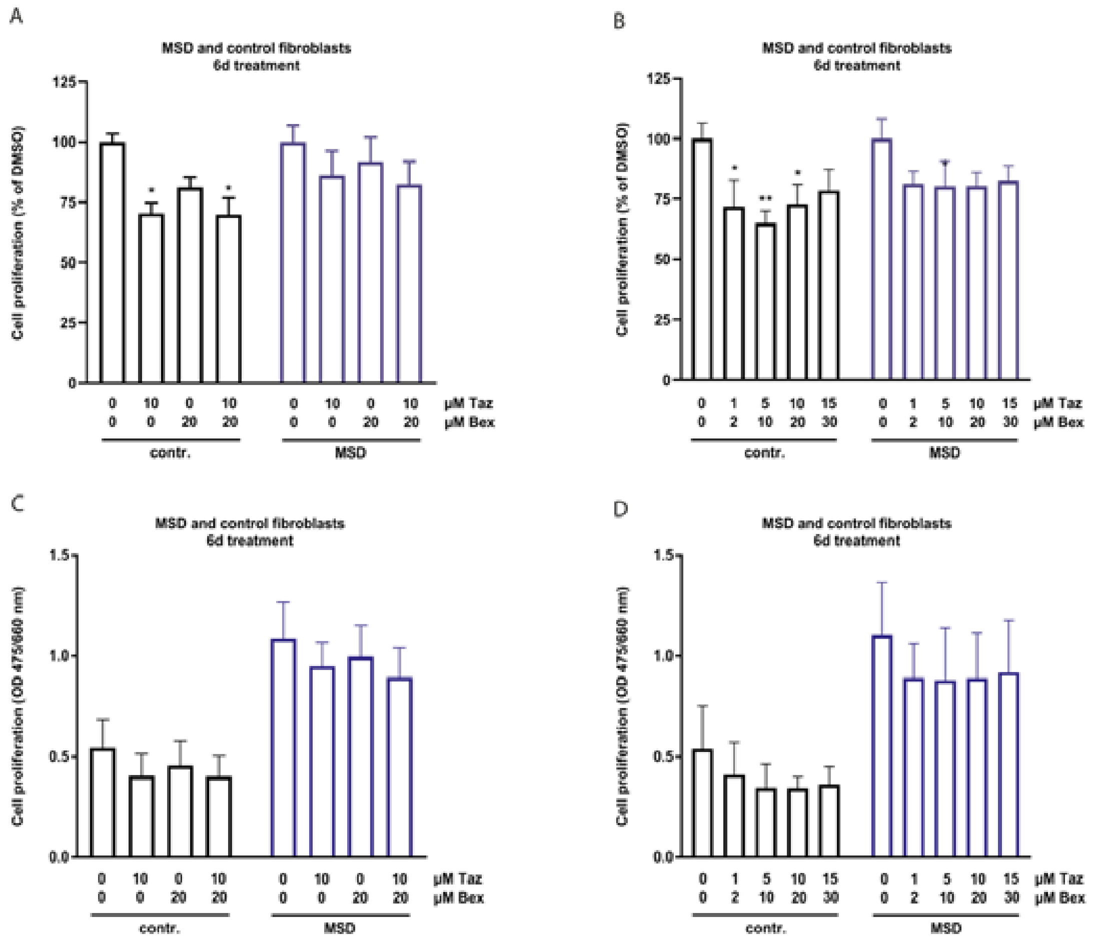

Appendix figure S7

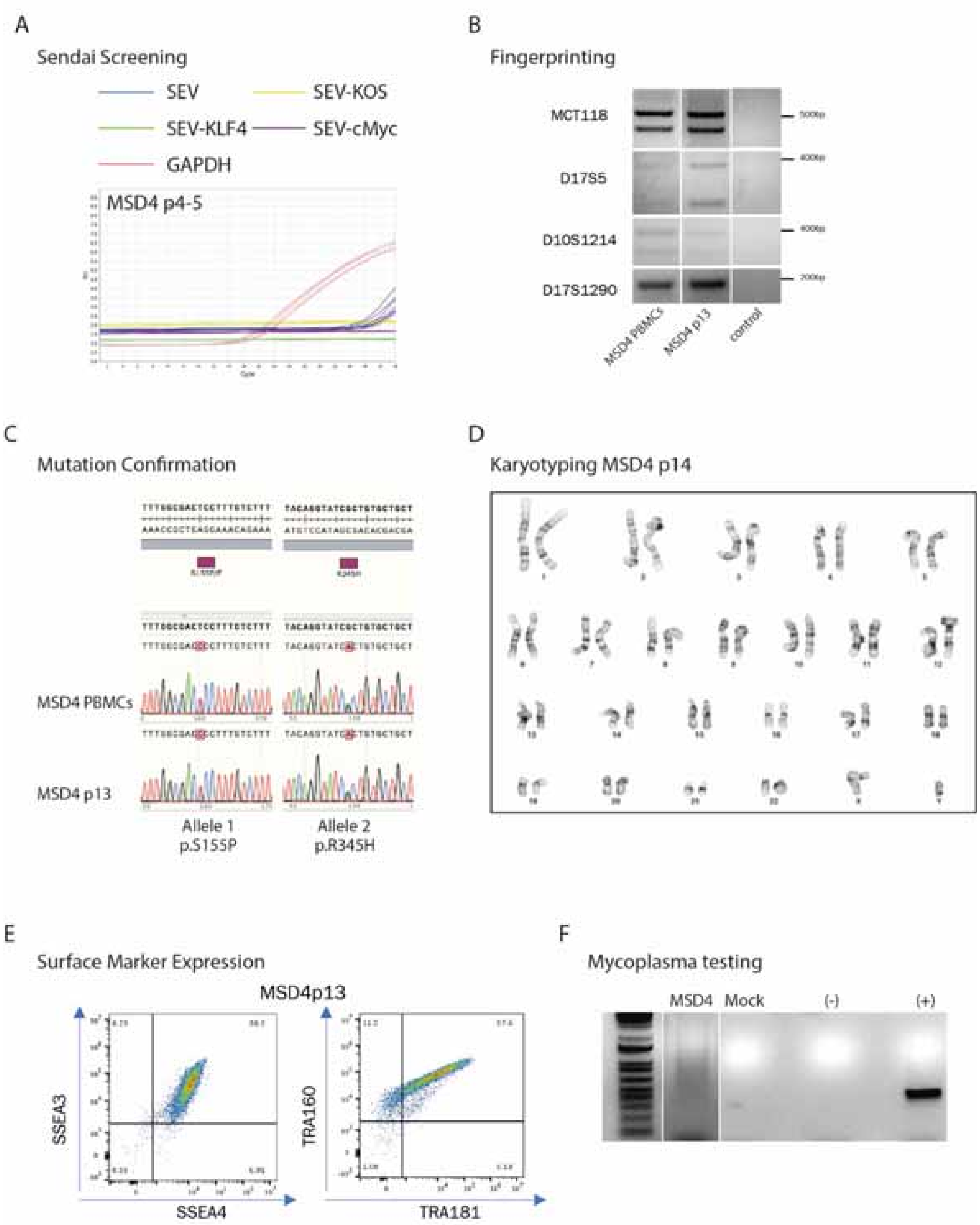

Appendix figure S8

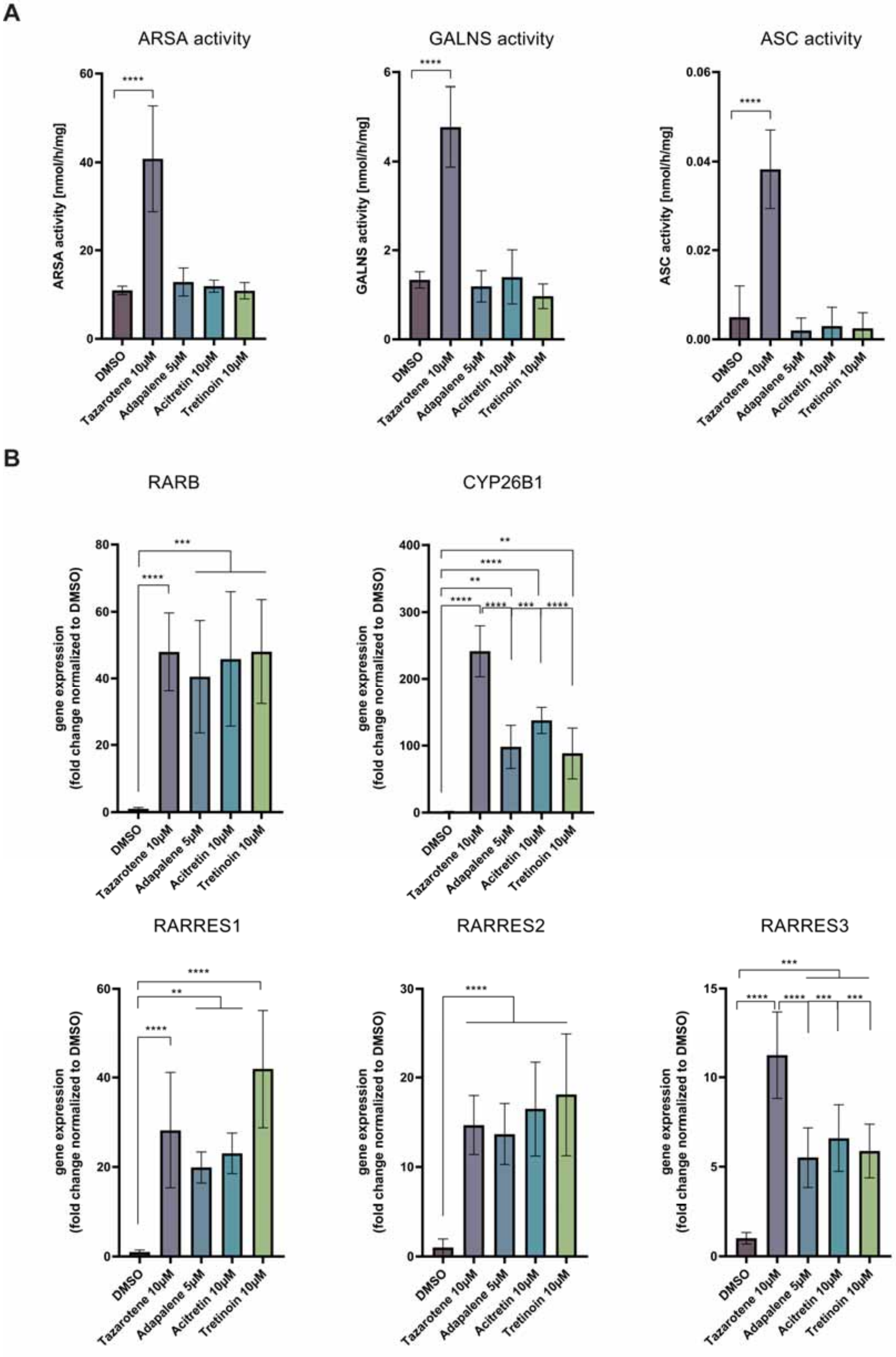

Appendix figure S9

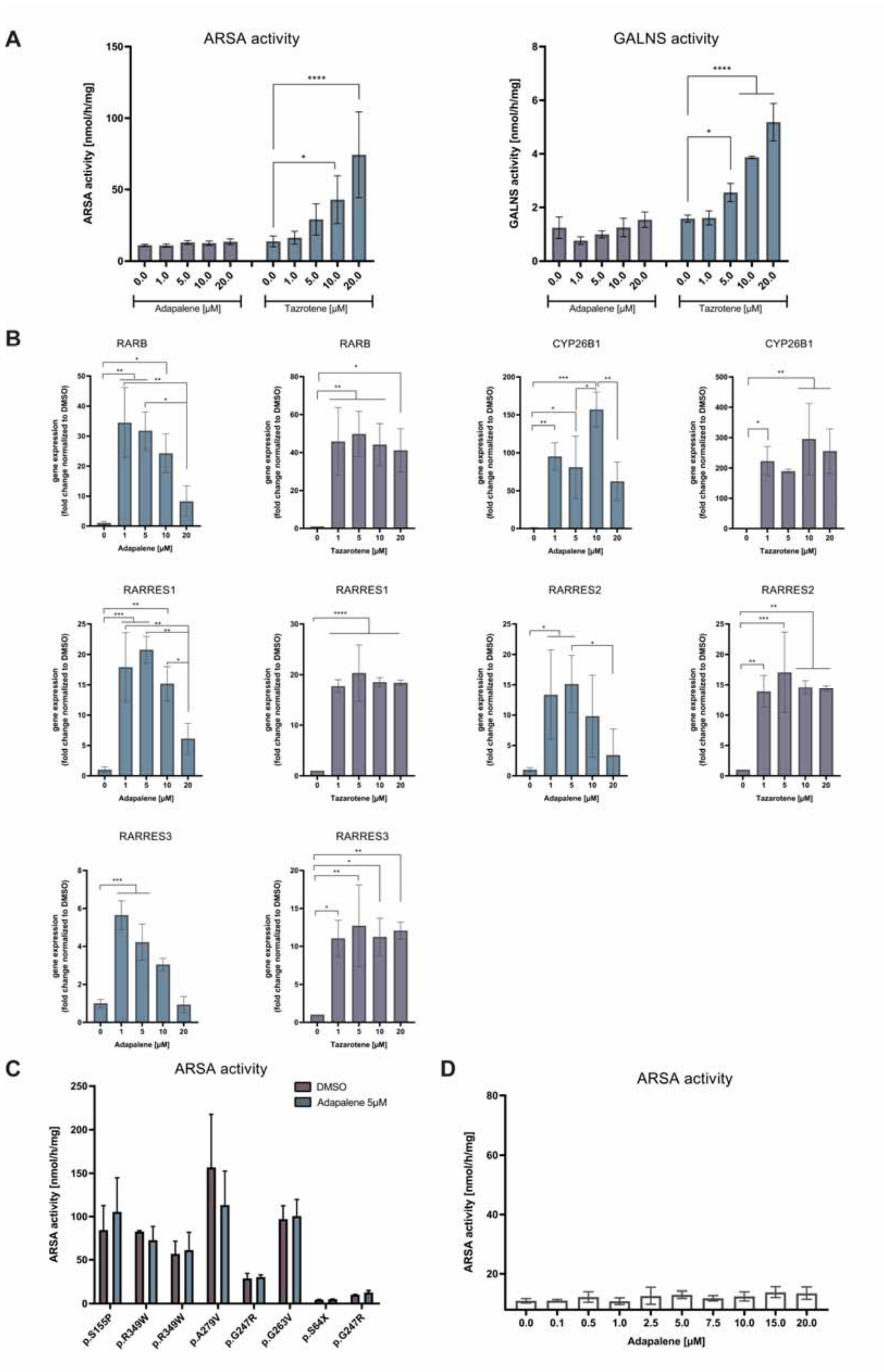

Appendix figure S10

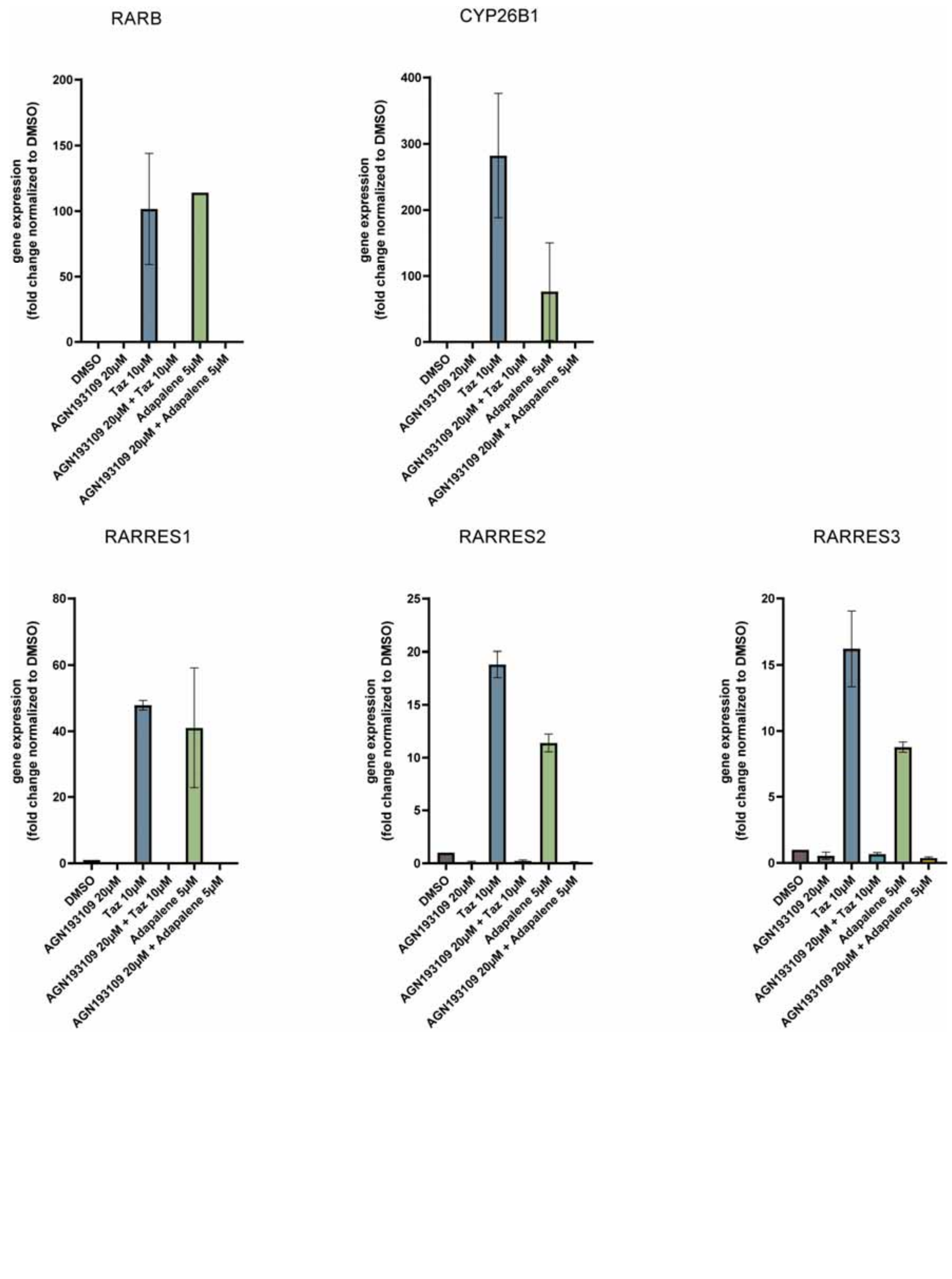

Appendix figure S11

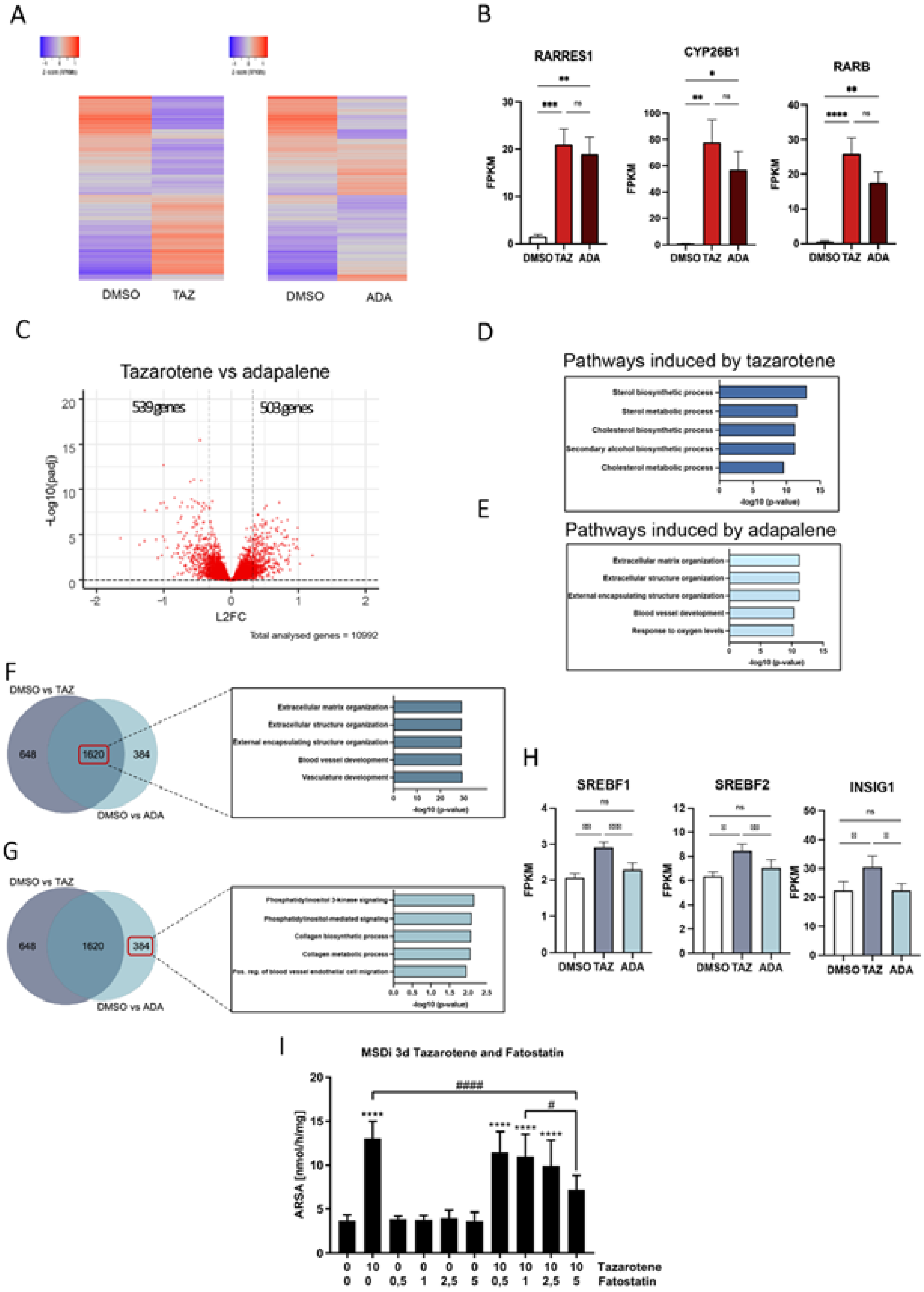

Appendix figure S12

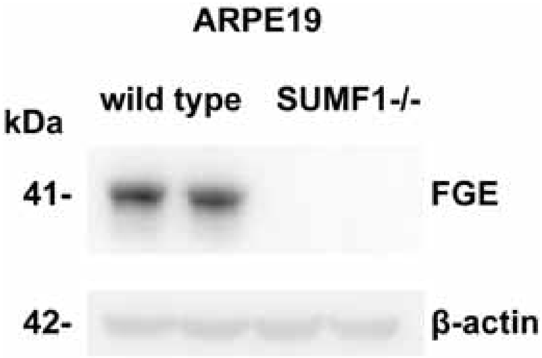


Appendix figure S13

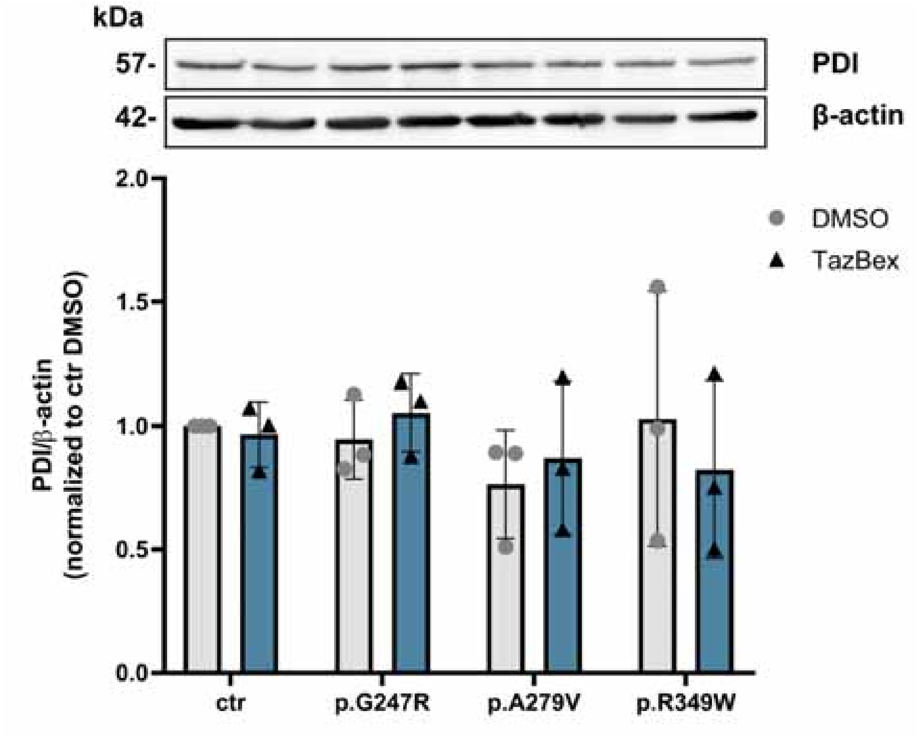

Appendix figure S14

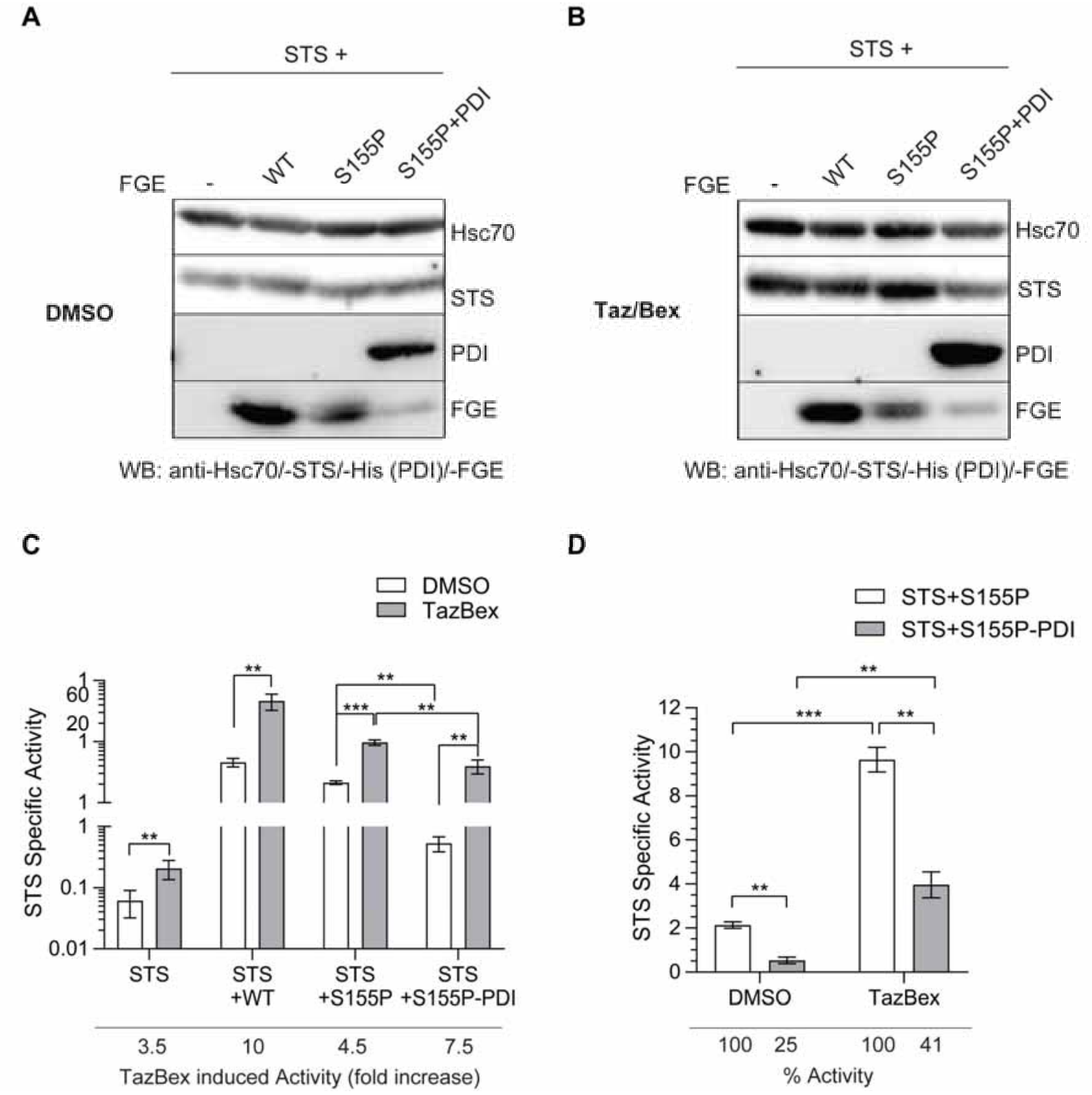

## Appendix supplementary figure legends

**Appendix figure S1: FDA approved drug library screen on MSD cells – plate layout, test plate and screening results plates 1 and 2.**

(**A**) Plate layout for the 96-well ARSA screening assay. (**B**) Representative plot of a screening plate with DMSO only treatment. ARSA activity is displayed as OD (y-axis) in every well as circle (x-axis). The grey colored area represents the upper and lower limit of baseline ARSA activity. N = 1 plate with 84 independent ARSA activity assays. (**C, D**) Original plots from screening a library of 785 licensed drugs with 80 drugs per plate at a final concentration of 10 μM in DMSO (DMSO concentration 1% per well) and incubation for 48 hours. Drugs that exceeded the upper OD limit of baseline were annotated as hits. N = 1 experiment per well.

**Appendix figure S2: FDA approved drug library screen on MSD cells – screening results plates 3-6.**

(**A-D**) Original plots from screening a library of 785 licensed drugs with 80 drugs per plate at a final concentration of 10 μM in DMSO (DMSO concentration 1% per well) and incubation for 48 hours. Drugs that exceeded the upper OD limit of baseline were annotated as hits. N = 1 experiment per well.

**Appendix figure S3: FDA approved drug library screen on MSD cells – screening results plates 7-10.**

(**A-D**) Original plots from screening a library of 785 licensed drugs with 80 drugs per plate at a final concentration of 10 μM in DMSO (DMSO concentration 1% per well) and incubation

for 48 hours. Drugs that exceeded the upper OD limit of baseline were annotated as hits. N = 1 experiment per well.

**Appendix figure S4: FDA approved drug library screen on MSD cells – rescreening and counterscreen plates 11-13**

(**A**) Original plot from rescreening of drugs with ODs below the lower activity range in the initial screen because of toxicity. Cells were incubated at 1 μM final concentration of each drug for 48 hours. N = 1 experiment per well. (**B**) Original plot from rescreening of drugs with ODs below the lower activity range in the initial screen because of toxicity. Cells were incubated at 0,1 μM final concentration of each drug for 48 hours. N = 1 experiment per well. (**C**) Original plot from counter screening of hit drugs at a concentration of 10 μM per well (1% DMSO content) for 48 hours devoid of cells. (N= 1 experiment per well).

**Appendix figure S5: Comparison of cell count, total protein content, and ARSA activity changes over time in MSD and control fibroblasts under treatment and control conditions**

(**A**) Manual cell count of MSD primary fibroblasts (FGE p.Gly247Arg) plated out at day 0 at standard cell quantities (medium) and additional low and high cell quantitiy. Cells were grown for 3, 6, and 9 days in presence of 0.1% (v/v) DMSO or at standard treatment conditions (medium cell quantity and 10 μM tazarotene, 20 μM bexarotene or 10/20 μM tazarotene/bexarotene, respectively). (**B**) Manual cell count of control fibroblasts plated out at day 0 at standard cell quantities (medium) and additional low and high cell quantitiy. Cells were grown for 3, 6, and 9 days in presence of DMSO or at standard treatment conditions (medium cell quantity and 10 μM tazarotene, 20 μM bexarotene or 10/20 μM tazarotene/bexarotene, respectively). (**C**) Total protein amount of MSD primary fibroblasts

(FGE p.Gly247Arg) plated out at day 0 at standard cell quantities (medium) and additional low and high cell quantitiy. Cells were grown for 3, 6, and 9 days in presence of DMSO or at standard treatment conditions (medium cell quantity and 10 µM tazarotene, 20 µM bexarotene or 10/20 µM tazarotene/bexarotene, respectively). (**D**) Total protein amount of control fibroblasts plated out at day 0 at standard cell quantities (medium) and additional low and high cell quantitiy. Cells were grown for 3, 6, and 9 days in presence of DMSO or at standard treatment conditions (medium cell quantity and 10 µM tazarotene, 20 µM bexarotene or 10/20 µM tazarotene/bexarotene, respectively). (**E**) ARSA activity in MSD primary fibroblasts (FGE p.Gly247Arg) plated out at day 0 at standard cell quantities (medium) and additional low and high cell quantitiy. Cells were grown for 3, 6, and 9 days in presence of DMSO or at standard treatment conditions (medium cell quantity and 10 µM tazarotene, 20 µM bexarotene or 10/20 µM tazarotene/bexarotene, respectively). (**F**) ARSA activity in control fibroblasts plated out at day 0 at standard cell quantities (medium) and additional low and high cell quantitiy. Cells were grown for 3, 6, and 9 days in presence of DMSO or at standard treatment conditions (medium cell quantity middle and 10 µM tazarotene, 20 µM bexarotene or 10/20 µM tazarotene/bexarotene, respectively). All data represent mean ±SD of 5-6 independent experiments. Two-way ANOVA followed by Tukey's test for multiple comparisons. Panel (A-E): Difference of tazarotene/bexarotene treatment against 0/0 µM treatment at the same time point, * $p<0.05$, ** $p<0.01$, *** $p<0.001$, **** $p<0.0001$. Panel F: Difference of DMSO low at day 9 against DMSO low at day 0. ** $p<0.01$, *** $p<0.001$, #### $p<0.0001$. Please see Appendix tables S41-S46 for full list of statistics of all comparisons (significant differences only).

**Appendix figure S6: Cell proliferation assessed by XTT-assay**

(**A**) Quantification of cell proliferation of MSD primary fibroblasts (variant FGE Gly247Arg homozygous) and control fibroblasts after 6 days of treatment with tazarotene, bexarotene, and tazrotene/bexarotene assessed by XTT assay (see materials and methods). Data represent mean ±SD of 8-20 independent experiments displayed as percentage of DMSO treated cells (negative control, 100% cell proliferation). One-way ANOVA followed by Tukey's test for multiple comparisons. * $p<0.05$. (**B**) Quantification of cell proliferation of MSD primary fibroblasts (variant FGE Gly247Arg homozygous) and control fibroblasts after 6 days of simultaneous treatment with increasing concentrations of tazarotene and bexarotene in a fixed combination of 1:2 assessed by XTT assay (see materials and methods). (**C,D**) Raw OD data without normalization to DMSO treatment for panel A and B, respectively. Statistical analysis was performed on normalized data only. All data represent mean ±SD of 8-12 independent experiments displayed as percentage of DMSO treated cells (negative control, 100% cell proliferation). One-way ANOVA followed by Tukey's test for multiple comparisons. * $p<0.05$, ** $p<0.01$. (**I**)

**Appendix figure S7: MSD iPSC line characterization.**

(**A**) Representative RT-PCR plot confirming the clearance of Sendai viral vectors in MSD4 iPSC at passages 4-5 by RT-PCR. (**B**) Representative PCR products using primer sets detecting short tandem repeat (STR) sequences from DNA fingerprinting of MSD patient derived iPSCs in comparison to PBMCs used for reprogramming. (**C**) Representative pictures of sequence analysis of *SUMF1* mutations in patient PBMCs and iPSC line. (**D**) Representative picture of G-band analysis for karyotyping MSD patient derived iPSCs (MSD4 p14). Normal karyotype (46,XY). (**E**) Representative flow cytometry plots of the expression of the stem cell surface markerson MSD4 iPSCs. SSEA3/4 and Tra160/81 were all

>95%. , as shown by flow cytometry. (**F**) Representative gel of PCR testing for mycoplasma infection in MSD4 cells and mock, positive and negative controls, respectively.

**Appendix figure S8: Sulfatase activities and transcriptional response of retinoids targets in MSD fibroblasts**

**(A)** Treatment of MSD primary fibroblasts (FGE p.Gly247Arg) for six days with 10 µM final concentration of four different retinoids. Only tazarotene significantly increased ARSA, GALNS, and ASC (steroidsulfatase) activities. Data represent mean ±SD of 3 independent experiments. One-way ANOVA followed by Tukey's test for multiple comparisons. Difference against DMSO control: **** $p<0.0001$. (**B**) Transcriptional response (gene expression) as determined by RT-PCR of selected targets of retinoids in MSD primary fibroblasts (FGE p.Gly247Arg) for six days with 10 µM final concentration of four different retinoids. Data represent mean ±SD of 3 independent experiments. One-way ANOVA followed by Tukey's test for multiple comparisons. ** $p<0.01$, *** $p<0.001$, **** $p<0.0001$,

**Appendix figure S9: Sulfatase activities and retinoid target gene expression upon different doses of adapalene and treatment response in different MSD primary fibroblast lines.**

**(A)** Treatment of MSD primary fibroblasts (FGE p.Gly247Arg) for six days with differeet concentrations of adapalene and tazarotene as control. Adapalene did not increase ARSA and GALNS activities. Data represent mean ±SD of 3 independent experiments. One-way ANOVA followed by Tukey's test for multiple comparisons. Difference against DMSO control: * $p<0.05$, **** $p<0.0001$. **(B)** Retinoid target gene expression in MSD primary fibroblasts (FGE p.Gly247Arg) after six days treatment with increasing concentrations of

adapalene and tazarotene. Adapalene was able to increase retinoid target gene expression. Data represent mean ±SD of 3 independent experiments. One-way ANOVA followed by Tukey's test for multiple comparisons. Difference against DMSO control: * p<0.05, *** p<0.01, *** p< 0.01, **** p<0.0001. **(C)** Treatment of eight MSD primary fibroblasts lines for six days with 5 µM adapalene. Adapalene did not increase ARSA activity in either cell line. Data represent mean ±SD of 3 independent experiments. Unpaired t-test. **(D)** Refined dose analysis of adapalene treatment in MSD primary fibroblasts (FGE p.Gly247Arg) for six days. Adapalene concentrations between 100 nM and 20 µM did not increase ARSA activity. Data represent mean ±SD of 3 independent experiments. One-way ANOVA followed by Tukey's test for multiple comparisons.

**Appendix figure S10: Adapalene increased retinoid target gene expression is mediated via RAR receptors.**

24 hours pre-treatment of of MSD primary fibroblasts (FGE p.Gly247Arg) with the pan-RAR receptor antagonist AGN193109 followed by treatment with 10 µM tazarotene and 5 µM adaplane, respectively, for 72 hours. Retinoid target gene expression was analysed by RT-PCR. AGN193109 treatment abrogated tazarotene and adapalene induced gene expression. Data represent mean ±SD of 3 independent experiments. RARB and adapalene control n=1 experiment.

**Appendix figure S11: Total RNAseq and differential gene expression from MSD fibroblast lines treated with tazarotene, adapalene, and DMSO.**

(**A**) Heatmaps illustrating differences in gene expression between seven different MSD fibroblast lines treated with tazarotene, adapalene, and DMSOin triplicates, respectively, and

analysed by total RNA sequencing (RNAseq). Differential gene expresion was analysed versus treatment condition and DMSO, respectively. (**B**) Expression levels indicated as fragments per kilobase million (FPKM) of retinioid response markers in all cell lines and treatment conditions. Data represent mean from one total RNAseq analysis in triplicates. Paired t-test. Difference against DMSO control: * $p<0.05$, *** $p<0.01$, *** $p< 0.01$, **** $p<0.0001$. (**C**) Transcriptomic changes of tazarotene and adapalene terated samples displayed as volcano plot and number of up- and down-regulated genes (tazarotene versus adapalene). (**D,E**) GO pathway analysis of genes differentially regulated after comparison of tazarotene and adapalene treatment and log10 value of p-values. (**F,G**) Venn diagram and number of exclusively regulated genes for tazarotene treatment (TAZ) versus DMSO condition (left) and adapalene treatment (ADA) versus DMSO (right) as well as number of overlapping genes identically regulated by both tazarotene and adapalene. (**F**) Subsequent GO pathway analysis and log10 value of p-values for identically regulated genes. (**G**) GO pathway analysis and log10 value of p-values for adapalene only regulated genes. (**H**) Expression levels indicated as fragments per kilobase million (FPKM) of tazarotene only regulated genes in all cell lines. Data represent mean from one total RNAseq analysis in triplicates. Paired t-test. Difference against DMSO control: * $p<0.05$, ** $p<0.01$, *** $p< 0.01$. (**I**) Quantification of ARSA activities in ARSA activity quantification after treatment of MSDi cells with increasing concentrations of fatostatin with and without simultaneous treatment of 10 µM tazarotene for 3 days. Data represent mean ±SD of 4 independent experiments. One-way ANOVA followed by Tukey's test for multiple comparisons. Displayed are significance levels for significant differences between tazarotene control and adjacent concentrations of combined tazarotene and fatostatin treatment. # $p<0.05$, #### $p<0.0001$. Difference against 0/0 µM control: **** $p<0.0001$.

**Appendix figure S12: SUMF1 -/- cell line evaluation**

Representative western blot pictures of CRISPR/Cas9 generated ARPE19 SUMF1 -/- cells and ARPE19 wild type control cells. No FGE-expression at 41 kDa in knock-out cells using an anti-FGE antibody. Detection of β-actin served as loading control.

**Appendix figure S13: PDI protein expression upon tazarotene/bexarotene treatment in MSD cell lines**

Representative western blot pictures and quantification of PDI protein expression in three MSD primary fibroblast cell lines and one control fibroblast line treated with tazarotene 10 µM and bexarotene 20 µM for six days compared to DMSO treated cells. Detection of β-actin served as loading control. Data represent mean ±SD of 3 independent experiments. One-way ANOVA followed by Tukey's test for multiple comparisons. No statistical significant differences were detected.

**Appendix figure S14: Expression of PDI mitigates tazarotene/bexarotene-mediated effect of sulfatase activation.**

**(A,B)** MSDi cells were treated with either DMSO or tazarotene 10 µM and bexarotene 20 µM for two days and transiently co-transfected with pBI-plasmids that express either steroid sulfatase (STS) alone or together with FGE-WT (wildtype), FGE-Ser155Pro variant or FGE-Ser155Pro and His-tagged PDI from a bi-directional doxycycline inducible promotor. 4h post-transfection, expression was induced with 1 µg/ml doxycycline for 24 h and cells harvested. Of note, either DMSO or tazarotene/bexarotene were present in all steps till the cells were harvested. STS activity assay was performed in cell lysates and equal amount of total protein from all lysates were resolved in SDS-PAGE and western blot probed with anti-STS, anti-FGE, anti-His and anti-Hsc70 (as loading control). The panels show a representative result from one experiment. (**C,D**) STS specific activity calculated based on quantification of

western blot signals that correspond to STS (after normalization to that of Hsc70). N=3 independet experiments, error bars represent mean ±SEM. One-way ANOVA followed by Tukey's test for multiple comparisons. ** $p<0.01$, *** $p<0.001$.

**Appendix supplementary tables**

**Appendix table S1:** List of drugs, suppliers and catalog numbers

| Drug | Supplier | Cat. No. |
|---|---|---|
| Vorinostat | Sigma-Aldrich | SML0061 |
| Clindamycine | Sigma-Aldrich | C5269 |
| Asenapine | Sigma-Aldrich | A7861 |
| Tazarotene | Sigma-Aldrich | T7080 |
| Vitamine A | Sigma-Aldrich | 7235-407 |
| 9-cis retinoic acid | Sigma-Aldrich | R4643 |
| Isotretinoin | Sigma-Aldrich | R3255 |
| Acitretin | Sigma-Aldrich | 44707 |
| Etretinate | Toronto Research Chemicals | E938000 |
| Adapalene | Sigma-Aldrich | A7486 |
| Bexarotene | Sigma-Aldrich | SML0282 |
| Tazarotenic acid | Sigma-Aldrich | SML1619 |
| Fatostatin | Sigma-Aldrich | 341329 |
| AGN 193109 | Tocris | 5758 |
| HX 531 | Sigma-Aldrich | SML2170 |
| DMSO | Serva | 39757 |

**Appendix table S2:** RT-PCR primers and primer sequences (5'>3')

| | | |
|---|---|---|
| beta-actin | Fwd | TGACCCAGATCATGTTTGAG |
| beta-actin | Rev | ATCACGATGCCAGTGGTA |
| RARB | Fwd | GTCACCGAGATAAGAACTGTGTTA |
| RARB | Rev | ACTCAGCTGTCATTTCATAGCTCTC |
| CYP26B1 | Fwd | TGGACCTCCTCATTGAGAGCA |
| CYP26B1 | Rev | GGCATAGGCCGCAAAGATCA |
| RARRES1 | Fwd | AAACCCCTTGGAAATAGTCAGC |
| RARRES1 | Rev | GGAAAGCCAAATCCCAGATGAG |
| RARRES2 | Fwd | AGAAACCCGAGTGCAAAGTCA |
| RARRES2 | Rev | AGAAACCCGAGTGCAAAGTCA |
| RARRES3 | Fwd | AACAGTGCAGAGGTGAAACGG |
| RARRES3 | Rev | GTTGGTACTCATGGTCCAAGC |

**Appendix table S3**: Mean values and significance levels for Figure 1 panel B (significant differences only)

| Comparison | Mean 1 | Mean 2 | Summary | Adjusted P Value |
|---|---|---|---|---|
| | | | | |
| neg. contr. vs. Tazarotene | 6,917 | 14,72 | ** | 0,0016 |
| neg. contr. vs. pos. contr. | 6,917 | 40,27 | **** | <0,0001 |
| Tazarotene vs. Clindamycin | 14,72 | 7,814 | ** | 0,0064 |
| Tazarotene vs. Vorinostat | 14,72 | 6,957 | ** | 0,0017 |
| Tazarotene vs. pos. contr. | 14,72 | 40,27 | **** | <0,0001 |
| Clindamycin vs. pos. contr. | 7,814 | 40,27 | **** | <0,0001 |
| Vorinostat vs. pos. contr. | 6,957 | 40,27 | **** | <0,0001 |
| Asenapine vs. pos. contr. | 9,617 | 40,27 | **** | <0,0001 |

**Appendix table S4**: Mean values and significance levels for Figure 1 panel C (significant differences only)

| Comparison | Mean 1 | Mean 2 | Summary | Adjusted P Value |
|---|---|---|---|---|
| | | | | |
| 0 vs. 2 | 2,511 | 5,895 | ** | 0,0076 |
| 0 vs. 5 | 2,511 | 8,759 | **** | <0,0001 |
| 0 vs. 10 | 2,511 | 9,942 | **** | <0,0001 |
| 0 vs. 15 | 2,511 | 12,67 | **** | <0,0001 |
| 0 vs. 25 | 2,511 | 13,48 | **** | <0,0001 |
| 0 vs. 50 | 2,511 | 12,66 | **** | <0,0001 |
| 2 vs. 5 | 5,895 | 8,759 | * | 0,0474 |
| 2 vs. 10 | 5,895 | 9,942 | *** | 0,001 |
| 2 vs. 15 | 5,895 | 12,67 | **** | <0,0001 |
| 2 vs. 25 | 5,895 | 13,48 | **** | <0,0001 |
| 2 vs. 50 | 5,895 | 12,66 | **** | <0,0001 |
| 5 vs. 15 | 8,759 | 12,67 | ** | 0,0071 |
| 5 vs. 25 | 8,759 | 13,48 | *** | 0,0008 |
| 5 vs. 50 | 8,759 | 12,66 | ** | 0,0074 |
| 10 vs. 25 | 9,942 | 13,48 | * | 0,0136 |

**Appendix table S5**: Mean values and significance levels for Figure 1 panel D (significant differences only)

| Comparison | Mean 1 | Mean 2 | Summary | Adjusted P Value |
|---|---|---|---|---|
| | | | | |

| | | | | |
|---|---|---|---|---|
| 0 vs. 5 | 4,121 | 9,026 | ** | 0,0014 |
| 0 vs. 10 | 4,121 | 9,185 | *** | 0,0009 |
| 0 vs. 15 | 4,121 | 9,998 | *** | 0,0002 |
| 0 vs. 20 | 4,121 | 11,02 | **** | <0,0001 |
| 0 vs. 25 | 4,121 | 11,19 | **** | <0,0001 |
| 0 vs. 50 | 4,121 | 9,775 | *** | 0,0003 |

**Appendix table S6**: Mean values and significance levels for Figure 1 panel E (significant differences only)

| **Comparison** | **Mean 1** | **Mean 2** | **Summary** | **Adjusted P Value** |
|---|---|---|---|---|
| | | | | |
| DMSO vs. Isotretinoin | 3,303 | 5,667 | ** | 0,0031 |
| DMSO vs. Tazarotene | 3,303 | 8,72 | **** | <0,0001 |
| DMSO vs. Bexarotene | 3,303 | 6,2 | *** | 0,0003 |
| DMSO vs. Tazarotenic acid | 3,303 | 5,577 | ** | 0,0046 |
| Vitamin A vs. Etretinate | 4,797 | 2,41 | ** | 0,0028 |
| Vitamin A vs. Tazarotene | 4,797 | 8,72 | **** | <0,0001 |
| 9-cis-retinoic acid vs. Etretinate | 4,893 | 2,41 | ** | 0,0018 |
| 9-cis-retinoic acid vs. Tazarotene | 4,893 | 8,72 | **** | <0,0001 |
| Isotretinoin vs. Etretinate | 5,667 | 2,41 | **** | <0,0001 |
| Isotretinoin vs. Adapalene 1µM | 5,667 | 3,33 | ** | 0,0035 |
| Isotretinoin vs. Tazarotene | 5,667 | 8,72 | *** | 0,0001 |
| Acitretin vs. Tazarotene | 3,963 | 8,72 | **** | <0,0001 |
| Acitretin vs. Bexarotene | 3,963 | 6,2 | ** | 0,0054 |
| Etretinate vs. Tazarotene | 2,41 | 8,72 | **** | <0,0001 |
| Etretinate vs. Bexarotene | 2,41 | 6,2 | **** | <0,0001 |
| Etretinate vs. Tazarotenic acid | 2,41 | 5,577 | **** | <0,0001 |
| Adapalene 1µM vs. Tazarotene | 3,33 | 8,72 | **** | <0,0001 |
| Adapalene 1µM vs. Bexarotene | 3,33 | 6,2 | *** | 0,0003 |
| Adapalene 1µM vs. Tazarotenic acid | 3,33 | 5,577 | ** | 0,0052 |
| Tazarotene vs. Bexarotene | 8,72 | 6,2 | ** | 0,0015 |
| Tazarotene vs. Tazarotenic acid | 8,72 | 5,577 | **** | <0,0001 |

**Appendix table S7**: Mean values and significance levels for Figure 1 panel F (significant differences only)

| **Comparison** | **Mean 1** | **Mean 2** | **Summary** | **Adjusted P Value** |
|---|---|---|---|---|
| | | | | |
| 0 vs. 5 | 2,633 | 5,175 | * | 0,0253 |
| 0 vs. 10 | 2,633 | 6,451 | **** | <0,0001 |
| 0 vs. 20 | 2,633 | 7,802 | **** | <0,0001 |

| | | | | |
|---|---|---|---|---|
| 0 vs. 50 | 2,633 | 7,067 | *** | 0,0008 |
| 1 vs. 10 | 3,283 | 6,451 | * | 0,0307 |
| 1 vs. 20 | 3,283 | 7,802 | *** | 0,0006 |
| 1 vs. 50 | 3,283 | 7,067 | * | 0,0416 |
| 2 vs. 20 | 3,75 | 7,802 | ** | 0,0025 |
| 5 vs. 20 | 5,175 | 7,802 | * | 0,0189 |

**Appendix table S8**: Mean values and significance levels for Figure 1 panel G (significant differences only)

| **Comparison** | **Mean 1** | **Mean 2** | **Summary** | **Adjusted P Value** |
|---|---|---|---|---|
| | | | | |
| 0/0 vs 1/2 | 4,515 | 12,56 | **** | <0,0001 |
| 0/0 vs 2/4 | 4,515 | 15,88 | **** | <0,0001 |
| 0/0 vs 5/10 | 4,515 | 19,9 | **** | <0,0001 |
| 0/0 vs 7.5/15 | 4,515 | 20,98 | **** | <0,0001 |
| 0/0 vs 10/20 | 4,515 | 19,65 | **** | <0,0001 |
| 0/0 vs 15/30 | 4,515 | 14 | **** | <0,0001 |
| 1/2 vs 2/4 | 12,56 | 15,88 | *** | 0,0003 |
| 1/2 vs 5/10 | 12,56 | 19,9 | **** | <0,0001 |
| 1/2 vs 7.5/15 | 12,56 | 20,98 | **** | <0,0001 |
| 1/2 vs 10/20 | 12,56 | 19,65 | **** | <0,0001 |
| 2/4 vs 5/10 | 15,88 | 19,9 | **** | <0,0001 |
| 2/4 vs 7.5/15 | 15,88 | 20,98 | **** | <0,0001 |
| 2/4 vs 10/20 | 15,88 | 19,65 | *** | 0,0007 |
| 5/10 vs 15/30 | 19,9 | 14 | **** | <0,0001 |
| 7.5/15 vs 15/30 | 20,98 | 14 | **** | <0,0001 |
| 10/20 vs 15/30 | 19,65 | 14 | **** | <0,0001 |

**Appendix table S9**: Mean values and significance levels for Figure 1 panel H (significant differences only)

| **Comparison** | **Mean 1** | **Mean 2** | **Summary** | **Adjusted P Value** |
|---|---|---|---|---|
| | | | | |
| 0 vs. 2 | 3,415 | 8,71 | * | 0,0196 |
| 0 vs. 3 | 3,415 | 14,29 | **** | <0,0001 |
| 0 vs. 6 | 3,415 | 20,06 | **** | <0,0001 |
| 0 vs. 9 | 3,415 | 25,76 | **** | <0,0001 |
| 0 vs. 12 | 3,415 | 15,18 | **** | <0,0001 |
| 2 vs. 3 | 8,71 | 14,29 | * | 0,0123 |
| 2 vs. 6 | 8,71 | 20,06 | **** | <0,0001 |
| 2 vs. 9 | 8,71 | 25,76 | **** | <0,0001 |

| | | | | |
|---|---|---|---|---|
| 2 vs. 12 | 8,71 | 15,18 | ** | 0,0081 |
| 3 vs. 6 | 14,29 | 20,06 | *** | 0,0002 |
| 3 vs. 9 | 14,29 | 25,76 | **** | <0,0001 |
| 6 vs. 9 | 20,06 | 25,76 | * | 0,0132 |
| 6 vs. 12 | 20,06 | 15,18 | * | 0,011 |
| 9 vs. 12 | 25,76 | 15,18 | **** | <0,0001 |

**Appendix table S10**: Mean values and significance levels for Figure 2 panel A (significant differences only)

| **Comparison** | **Mean 1** | **Mean 2** | **Summary** | **Adjusted P Value** |
|---|---|---|---|---|
| | | | | |
| 0 vs. 5 | 15,9 | 30,45 | *** | 0,001 |
| 0 vs. 10 | 15,9 | 47,89 | **** | <0,0001 |
| 0 vs. 25 | 15,9 | 78 | **** | <0,0001 |
| 0 vs. 50 | 15,9 | 94,45 | **** | <0,0001 |
| 0 vs. 75 | 15,9 | 84,8 | **** | <0,0001 |
| 0 vs. 100 | 15,9 | 65,39 | **** | <0,0001 |
| 1 vs. 10 | 18,91 | 47,89 | **** | <0,0001 |
| 1 vs. 25 | 18,91 | 78 | **** | <0,0001 |
| 1 vs. 50 | 18,91 | 94,45 | **** | <0,0001 |
| 1 vs. 75 | 18,91 | 84,8 | **** | <0,0001 |
| 1 vs. 100 | 18,91 | 65,39 | **** | <0,0001 |
| 2.5 vs. 10 | 22,34 | 47,89 | **** | <0,0001 |
| 2.5 vs. 25 | 22,34 | 78 | **** | <0,0001 |
| 2.5 vs. 50 | 22,34 | 94,45 | **** | <0,0001 |
| 2.5 vs. 75 | 22,34 | 84,8 | **** | <0,0001 |
| 2.5 vs. 100 | 22,34 | 65,39 | **** | <0,0001 |
| 5 vs. 10 | 30,45 | 47,89 | **** | <0,0001 |
| 5 vs. 25 | 30,45 | 78 | **** | <0,0001 |
| 5 vs. 50 | 30,45 | 94,45 | **** | <0,0001 |
| 5 vs. 75 | 30,45 | 84,8 | **** | <0,0001 |
| 5 vs. 100 | 30,45 | 65,39 | **** | <0,0001 |
| 10 vs. 25 | 47,89 | 78 | **** | <0,0001 |
| 10 vs. 50 | 47,89 | 94,45 | **** | <0,0001 |
| 10 vs. 75 | 47,89 | 84,8 | **** | <0,0001 |
| 10 vs. 100 | 47,89 | 65,39 | **** | <0,0001 |
| 25 vs. 50 | 78 | 94,45 | *** | 0,0009 |
| 25 vs. 100 | 78 | 65,39 | * | 0,0193 |
| 50 vs. 100 | 94,45 | 65,39 | **** | <0,0001 |
| 75 vs. 100 | 84,8 | 65,39 | *** | 0,0003 |

**Appendix table S11**: Mean values and significance levels for Figure 2 panel B (significant differences only)

| Comparison | Mean 1 | Mean 2 | Summary | Adjusted P Value |
|---|---|---|---|---|
| | | | | |
| 0 vs. 10 | 14,84 | 22,17 | * | 0,0112 |
| 0 vs. 20 | 14,84 | 22,48 | * | 0,0156 |
| 0 vs. 25 | 14,84 | 26,6 | **** | <0,0001 |
| 0 vs. 50 | 14,84 | 30,58 | **** | <0,0001 |
| 2.5 vs. 50 | 20,2 | 30,58 | ** | 0,0032 |
| 2.5 vs. 75 | 20,2 | 7,603 | ** | 0,0017 |
| 5 vs. 25 | 19,37 | 26,6 | ** | 0,0018 |
| 5 vs. 50 | 19,37 | 30,58 | **** | <0,0001 |
| 5 vs. 75 | 19,37 | 7,603 | *** | 0,0002 |
| 10 vs. 50 | 22,17 | 30,58 | ** | 0,0061 |
| 10 vs. 75 | 22,17 | 7,603 | **** | <0,0001 |
| 20 vs. 50 | 22,48 | 30,58 | * | 0,0177 |
| 20 vs. 75 | 22,48 | 7,603 | **** | <0,0001 |
| 25 vs. 75 | 26,6 | 7,603 | **** | <0,0001 |
| 50 vs. 75 | 30,58 | 7,603 | **** | <0,0001 |

**Appendix table S12**: Mean values and significance levels for Figure 2 panel C (significant differences only)

| Comparison | Mean 1 | Mean 2 | Summary | Adjusted P Value |
|---|---|---|---|---|
| | | | | |
| 0/0 vs 2/5 | 15,59 | 32,75 | ** | 0,0016 |
| 0/0 vs 5/10 | 15,59 | 64,17 | **** | <0,0001 |
| 0/0 vs 7/5 | 15,59 | 103,1 | **** | <0,0001 |
| 0/0 vs 10/20 | 15,59 | 111 | **** | <0,0001 |
| 0/0 vs 15/30 | 15,59 | 73,08 | **** | <0,0001 |
| 0.1/0.2 vs 2.5/5 | 17,28 | 32,75 | * | 0,021 |
| 0.1/0.2 vs 5/10 | 17,28 | 64,17 | **** | <0,0001 |
| 0.1/0.2 vs 7.5/10 | 17,28 | 103,1 | **** | <0,0001 |
| 0.1/0.2 vs 10/20 | 17,28 | 111 | **** | <0,0001 |
| 0.1/0.2 vs 15/30 | 17,28 | 73,08 | **** | <0,0001 |
| 1/2 vs 5/10 | 22,83 | 64,17 | **** | <0,0001 |
| 1/2 vs 7/5 | 22,83 | 103,1 | **** | <0,0001 |
| 1/2 vs 10/20 | 22,83 | 111 | **** | <0,0001 |
| 1/2 vs 15/30 | 22,83 | 73,08 | **** | <0,0001 |
| 2.5/5 vs 5/10 | 32,75 | 64,17 | **** | <0,0001 |
| 2.5/5 vs 7.5/15 | 32,75 | 103,1 | **** | <0,0001 |
| 2.5/5 vs 10/20 | 32,75 | 111 | **** | <0,0001 |
| 2.5/5 vs 15/30 | 32,75 | 73,08 | **** | <0,0001 |
| 5/10 vs 7/5 | 64,17 | 103,1 | **** | <0,0001 |

| | | | | |
|---|---|---|---|---|
| 5/10 vs 10/20 | 64,17 | 111 | **** | <0,0001 |
| 7.5/15 vs 15/30 | 103,1 | 73,08 | **** | <0,0001 |
| 10/20 vs 15/30 | 111 | 73,08 | **** | <0,0001 |

**Appendix table S13**: Mean values and significance levels for Figure 2 panel D (significant differences only)

| **Comparison** | **Mean 1** | **Mean 2** | **Summary** | **Adjusted P Value** |
|---|---|---|---|---|
| | | | | |
| 0 vs. 3 | 16,09 | 43,1 | * | 0,0443 |
| 0 vs. 6 | 16,09 | 109,5 | **** | <0,0001 |
| 0 vs. 9 | 16,09 | 175,8 | **** | <0,0001 |
| 0 vs. 14 | 16,09 | 181,2 | **** | <0,0001 |
| 0 vs. 21 | 16,09 | 168,3 | **** | <0,0001 |
| 3 vs. 6 | 43,1 | 109,5 | **** | <0,0001 |
| 3 vs. 9 | 43,1 | 175,8 | **** | <0,0001 |
| 3 vs. 14 | 43,1 | 181,2 | **** | <0,0001 |
| 3 vs. 21 | 43,1 | 168,3 | **** | <0,0001 |
| 6 vs. 9 | 109,5 | 175,8 | **** | <0,0001 |
| 6 vs. 14 | 109,5 | 181,2 | **** | <0,0001 |
| 6 vs. 21 | 109,5 | 168,3 | *** | 0,0001 |

**Appendix table S14**: Mean values and significance levels for Figure 2 panel E (significant differences only)

| **Comparison** | **Mean 1** | **Mean 2** | **Summary** | **Adjusted P Value** |
|---|---|---|---|---|
| | | | | |
| ARSA - vs. + | 17,06 | 109,5 | **** | <0,0001 |
| ARSB - vs. + | 18,09 | 46,96 | *** | 0,0001 |
| GALNS - vs. + | 3,54 | 11,67 | *** | 0,0003 |
| STS - vs. + | 14,8 | 113,6 | **** | <0,0001 |

**Appendix table S15**: Mean values and significance levels for Figure 2 panel F (significant differences only)

| **Comparison** | **Mean 1** | **Mean 2** | **Summary** | **Adjusted P Value** |
|---|---|---|---|---|
| | | | | |
| p.G247R - vs. + | 17,71 | 98,09 | **** | <0,0001 |
| p.G263V - vs. + | 131,2 | 324,5 | **** | <0,0001 |
| p.A279V - vs. + | 45 | 88 | *** | 0,0005 |

| | | | | |
|---|---|---|---|---|
| p.R349W - vs. + | 27,89 | 97,87 | **** | <0,0001 |

**Appendix table S16**: Mean values and significance levels for Figure 2 panel G (significant differences only)

| **Comparison** | **Mean 1** | **Mean 2** | **Summary** | **Adjusted P Value** |
|---|---|---|---|---|
| | | | | |
| contr. - vs. + | 30,38 | 35,02 | **** | <0,0001 |
| MSD - vs. + | 10,97 | 17,57 | **** | <0,0001 |

**Appendix table S17**: Mean values and significance levels for Figure 2 panel H (significant differences only)

| **Comparison** | **Mean 1** | **Mean 2** | **Summary** | **Adjusted P Value** |
|---|---|---|---|---|
| | | | | |
| contr. - vs. + | 2,287 | 1,78 | * | 0,0303 |
| MSD - vs. + | 0,219 | 0,4617 | *** | 0,0002 |

**Appendix table S18**: Mean values and significance levels for Figure 3 panel C (significant differences only)

| **Comparison** | **Mean 1** | **Mean 2** | **Summary** | **Adjusted P Value** |
|---|---|---|---|---|
| | | | | |
| ctr dmso vs. MSD dmso | 740085758 | 3733811299 | *** | 0,0002 |
| MSD dmso vs. ctr tazbex | 3733811299 | 785298854 | *** | 0,0002 |
| MSD dmso vs. msd tazbex | 3733811299 | 1912504979 | * | 0,0115 |

**Appendix table S19**: Mean values and significance levels for Figure 3 panel D (significant differences only)

| **Comparison** | **Mean 1** | **Mean 2** | **Summary** | **Adjusted P Value** |
|---|---|---|---|---|
| | | | | |
| ctr dmso vs. MSD dmso | 740085758 | 3733811299 | *** | 0,0002 |
| MSD dmso vs. ctr tazbex | 3733811299 | 785298854 | *** | 0,0002 |
| MSD dmso vs. msd tazbex | 3733811299 | 1912504979 | * | 0,0115 |

**Appendix table S20**: Mean values and significance levels for Figure 4 panel A (significant differences only)

| Comparison | Mean 1 | Mean 2 | Summary | Adjusted P Value |
|---|---|---|---|---|
| | | | | |
| 0:DMSO vs. 0:Tazarotene 10 µM | 3,263 | 10,38 | **** | <0,0001 |
| 0:DMSO vs. 0:Tazarotene 10 µM Bexarotene 20µM | 3,263 | 13,89 | **** | <0,0001 |
| 0:DMSO vs. 1:Tazarotene 10 µM | 3,263 | 7,273 | **** | <0,0001 |
| 0:DMSO vs. 1:Tazarotene 10 µM Bexarotene 20µM | 3,263 | 11,18 | **** | <0,0001 |
| 0:DMSO vs. 5:Tazarotene 10 µM | 3,263 | 6,725 | **** | <0,0001 |
| 0:DMSO vs. 5:Tazarotene 10 µM Bexarotene 20µM | 3,263 | 10,16 | **** | <0,0001 |
| 0:DMSO vs. 10:Tazarotene 10 µM | 3,263 | 6,546 | **** | <0,0001 |
| 0:DMSO vs. 10:Tazarotene 10 µM Bexarotene 20µM | 3,263 | 9,414 | **** | <0,0001 |
| 0:DMSO vs. 20:Tazarotene 10 µM Bexarotene 20µM | 3,263 | 6,536 | ** | 0,0021 |
| 0:Tazarotene 10 µM vs. 0:Bexarotene 20 µM | 10,38 | 4,74 | **** | <0,0001 |
| 0:Tazarotene 10 µM vs. 0:Tazarotene 10 µM Bexarotene 20µM | 10,38 | 13,89 | ** | 0,0011 |
| 0:Tazarotene 10 µM vs. 1:DMSO | 10,38 | 3,151 | **** | <0,0001 |
| 0:Tazarotene 10 µM vs. 1:Tazarotene 10 µM | 10,38 | 7,273 | *** | 0,0004 |
| 0:Tazarotene 10 µM vs. 1:Bexarotene 20 µM | 10,38 | 4,493 | **** | <0,0001 |
| 0:Tazarotene 10 µM vs. 5:DMSO | 10,38 | 3,326 | **** | <0,0001 |
| 0:Tazarotene 10 µM vs. 5:Tazarotene 10 µM | 10,38 | 6,725 | **** | <0,0001 |
| 0:Tazarotene 10 µM vs. 5:Bexarotene 20 µM | 10,38 | 4,983 | **** | <0,0001 |
| 0:Tazarotene 10 µM vs. 10:DMSO | 10,38 | 3,378 | **** | <0,0001 |
| 0:Tazarotene 10 µM vs. 10:Tazarotene 10 µM | 10,38 | 6,546 | **** | <0,0001 |
| 0:Tazarotene 10 µM vs. 10:Bexarotene 20 µM | 10,38 | 4,517 | **** | <0,0001 |
| 0:Tazarotene 10 µM vs. 20:DMSO | 10,38 | 4,195 | **** | <0,0001 |
| 0:Tazarotene 10 µM vs. 20:Tazarotene 10 µM | 10,38 | 5,021 | **** | <0,0001 |
| 0:Tazarotene 10 µM vs. 20:Bexarotene 20 µM | 10,38 | 4,693 | **** | <0,0001 |
| 0:Tazarotene 10 µM vs. 20:Tazarotene 10 µM Bexarotene 20µM | 10,38 | 6,536 | *** | 0,0002 |
| 0:Bexarotene 20 µM vs. 0:Tazarotene 10 µM Bexarotene 20µM | 4,74 | 13,89 | **** | <0,0001 |
| 0:Bexarotene 20 µM vs. 1:Tazarotene 10 µM Bexarotene 20µM | 4,74 | 11,18 | **** | <0,0001 |
| 0:Bexarotene 20 µM vs. 5:Tazarotene 10 µM Bexarotene 20µM | 4,74 | 10,16 | **** | <0,0001 |
| 0:Bexarotene 20 µM vs. 10:Tazarotene 10 µM Bexarotene 20µM | 4,74 | 9,414 | *** | 0,0008 |
| 0:Tazarotene 10 µM Bexarotene 20µM vs. 1:DMSO | 13,89 | 3,151 | **** | <0,0001 |

| | | | | |
|---|---|---|---|---|
| 0:Tazarotene 10 µM<br>Bexarotene 20µM vs. 1:Tazarotene 10 µM | 13,89 | 7,273 | **** | <0,0001 |
| 0:Tazarotene 10 µM<br>Bexarotene 20µM vs. 1:Bexarotene 20 µM | 13,89 | 4,493 | **** | <0,0001 |
| 0:Tazarotene 10 µM<br>Bexarotene 20µM vs. 5:DMSO | 13,89 | 3,326 | **** | <0,0001 |
| 0:Tazarotene 10 µM<br>Bexarotene 20µM vs. 5:Tazarotene 10 µM | 13,89 | 6,725 | **** | <0,0001 |
| 0:Tazarotene 10 µM<br>Bexarotene 20µM vs. 5:Bexarotene 20 µM | 13,89 | 4,983 | **** | <0,0001 |
| 0:Tazarotene 10 µM<br>Bexarotene 20µM vs. 5:Tazarotene 10 µM<br>Bexarotene 20µM | 13,89 | 10,16 | ** | 0,0034 |
| 0:Tazarotene 10 µM<br>Bexarotene 20µM vs. 10:DMSO | 13,89 | 3,378 | **** | <0,0001 |
| 0:Tazarotene 10 µM<br>Bexarotene 20µM vs. 10:Tazarotene 10 µM | 13,89 | 6,546 | **** | <0,0001 |
| 0:Tazarotene 10 µM<br>Bexarotene 20µM vs. 10:Bexarotene 20 µM | 13,89 | 4,517 | **** | <0,0001 |
| 0:Tazarotene 10 µM<br>Bexarotene 20µM vs. 10:Tazarotene 10 µM<br>Bexarotene 20µM | 13,89 | 9,414 | **** | <0,0001 |
| 0:Tazarotene 10 µM<br>Bexarotene 20µM vs. 20:DMSO | 13,89 | 4,195 | **** | <0,0001 |
| 0:Tazarotene 10 µM<br>Bexarotene 20µM vs. 20:Tazarotene 10 µM | 13,89 | 5,021 | **** | <0,0001 |
| 0:Tazarotene 10 µM<br>Bexarotene 20µM vs. 20:Bexarotene 20 µM | 13,89 | 4,693 | **** | <0,0001 |
| 0:Tazarotene 10 µM<br>Bexarotene 20µM vs. 20:Tazarotene 10 µM<br>Bexarotene 20µM | 13,89 | 6,536 | **** | <0,0001 |
| 1:DMSO vs. 1:Tazarotene 10 µM | 3,151 | 7,273 | **** | <0,0001 |
| 1:DMSO vs. 1:Tazarotene 10 µM<br>Bexarotene 20µM | 3,151 | 11,18 | **** | <0,0001 |
| 1:DMSO vs. 5:Tazarotene 10 µM | 3,151 | 6,725 | **** | <0,0001 |
| 1:DMSO vs. 5:Tazarotene 10 µM<br>Bexarotene 20µM | 3,151 | 10,16 | **** | <0,0001 |
| 1:DMSO vs. 10:Tazarotene 10 µM | 3,151 | 6,546 | **** | <0,0001 |
| 1:DMSO vs. 10:Tazarotene 10 µM<br>Bexarotene 20µM | 3,151 | 9,414 | **** | <0,0001 |
| 1:DMSO vs. 20:Tazarotene 10 µM<br>Bexarotene 20µM | 3,151 | 6,536 | ** | 0,0011 |
| 1:Tazarotene 10 µM vs. 1:Tazarotene 10 µM<br>Bexarotene 20µM | 7,273 | 11,18 | *** | 0,0001 |
| 1:Tazarotene 10 µM vs. 5:DMSO | 7,273 | 3,326 | **** | <0,0001 |
| 1:Tazarotene 10 µM vs. 5:Tazarotene 10 µM<br>Bexarotene 20µM | 7,273 | 10,16 | * | 0,0229 |
| 1:Tazarotene 10 µM vs. 10:DMSO | 7,273 | 3,378 | **** | <0,0001 |

| | | | | |
|---|---|---|---|---|
| 1:Tazarotene 10 µM vs. 20:DMSO | 7,273 | 4,195 | *** | 0,0002 |
| 1:Bexarotene 20 µM vs. 1:Tazarotene 10 µM Bexarotene 20µM | 4,493 | 11,18 | **** | <0,0001 |
| 1:Bexarotene 20 µM vs. 5:Tazarotene 10 µM Bexarotene 20µM | 4,493 | 10,16 | **** | <0,0001 |
| 1:Bexarotene 20 µM vs. 10:Tazarotene 10 µM Bexarotene 20µM | 4,493 | 9,414 | *** | 0,0003 |
| 1:Tazarotene 10 µM Bexarotene 20µM vs. 5:DMSO | 11,18 | 3,326 | **** | <0,0001 |
| 1:Tazarotene 10 µM Bexarotene 20µM vs. 5:Tazarotene 10 µM | 11,18 | 6,725 | **** | <0,0001 |
| 1:Tazarotene 10 µM Bexarotene 20µM vs. 5:Bexarotene 20 µM | 11,18 | 4,983 | **** | <0,0001 |
| 1:Tazarotene 10 µM Bexarotene 20µM vs. 10:DMSO | 11,18 | 3,378 | **** | <0,0001 |
| 1:Tazarotene 10 µM Bexarotene 20µM vs. 10:Tazarotene 10 µM | 11,18 | 6,546 | **** | <0,0001 |
| 1:Tazarotene 10 µM Bexarotene 20µM vs. 10:Bexarotene 20 µM | 11,18 | 4,517 | **** | <0,0001 |
| 1:Tazarotene 10 µM Bexarotene 20µM vs. 20:DMSO | 11,18 | 4,195 | **** | <0,0001 |
| 1:Tazarotene 10 µM Bexarotene 20µM vs. 20:Tazarotene 10 µM | 11,18 | 5,021 | **** | <0,0001 |
| 1:Tazarotene 10 µM Bexarotene 20µM vs. 20:Bexarotene 20 µM | 11,18 | 4,693 | **** | <0,0001 |
| 1:Tazarotene 10 µM Bexarotene 20µM vs. 20:Tazarotene 10 µM Bexarotene 20µM | 11,18 | 6,536 | **** | <0,0001 |
| 5:DMSO vs. 5:Tazarotene 10 µM | 3,326 | 6,725 | **** | <0,0001 |
| 5:DMSO vs. 5:Tazarotene 10 µM Bexarotene 20µM | 3,326 | 10,16 | **** | <0,0001 |
| 5:DMSO vs. 10:Tazarotene 10 µM | 3,326 | 6,546 | **** | <0,0001 |
| 5:DMSO vs. 10:Tazarotene 10 µM Bexarotene 20µM | 3,326 | 9,414 | **** | <0,0001 |
| 5:DMSO vs. 20:Tazarotene 10 µM Bexarotene 20µM | 3,326 | 6,536 | ** | 0,0029 |
| 5:Tazarotene 10 µM vs. 5:Tazarotene 10 µM Bexarotene 20µM | 6,725 | 10,16 | ** | 0,0016 |
| 5:Tazarotene 10 µM vs. 10:DMSO | 6,725 | 3,378 | **** | <0,0001 |
| 5:Tazarotene 10 µM vs. 20:DMSO | 6,725 | 4,195 | ** | 0,007 |
| 5:Bexarotene 20 µM vs. 5:Tazarotene 10 µM Bexarotene 20µM | 4,983 | 10,16 | **** | <0,0001 |
| 5:Bexarotene 20 µM vs. 10:Tazarotene 10 µM Bexarotene 20µM | 4,983 | 9,414 | ** | 0,0021 |
| 5:Tazarotene 10 µM Bexarotene 20µM vs. 10:DMSO | 10,16 | 3,378 | **** | <0,0001 |
| 5:Tazarotene 10 µM Bexarotene 20µM vs. 10:Tazarotene 10 µM | 10,16 | 6,546 | *** | 0,0006 |

| | | | | |
|---|---|---|---|---|
| 5:Tazarotene 10 µM Bexarotene 20µM vs. 10:Bexarotene 20 µM | 10,16 | 4,517 | **** | <0,0001 |
| 5:Tazarotene 10 µM Bexarotene 20µM vs. 20:DMSO | 10,16 | 4,195 | **** | <0,0001 |
| 5:Tazarotene 10 µM Bexarotene 20µM vs. 20:Tazarotene 10 µM | 10,16 | 5,021 | **** | <0,0001 |
| 5:Tazarotene 10 µM Bexarotene 20µM vs. 20:Bexarotene 20 µM | 10,16 | 4,693 | **** | <0,0001 |
| 5:Tazarotene 10 µM Bexarotene 20µM vs. 20:Tazarotene 10 µM Bexarotene 20µM | 10,16 | 6,536 | ** | 0,0054 |
| 10:DMSO vs. 10:Tazarotene 10 µM | 3,378 | 6,546 | *** | 0,0001 |
| 10:DMSO vs. 10:Tazarotene 10 µM Bexarotene 20µM | 3,378 | 9,414 | **** | <0,0001 |
| 10:DMSO vs. 20:Tazarotene 10 µM Bexarotene 20µM | 3,378 | 6,536 | ** | 0,0038 |
| 10:Tazarotene 10 µM vs. 10:Tazarotene 10 µM Bexarotene 20µM | 6,546 | 9,414 | * | 0,0244 |
| 10:Tazarotene 10 µM vs. 20:DMSO | 6,546 | 4,195 | * | 0,0197 |
| 10:Bexarotene 20 µM vs. 10:Tazarotene 10 µM Bexarotene 20µM | 4,517 | 9,414 | *** | 0,0003 |
| 10:Tazarotene 10 µM Bexarotene 20µM vs. 20:DMSO | 9,414 | 4,195 | **** | <0,0001 |
| 10:Tazarotene 10 µM Bexarotene 20µM vs. 20:Tazarotene 10 µM | 9,414 | 5,021 | **** | <0,0001 |
| 10:Tazarotene 10 µM Bexarotene 20µM vs. 20:Bexarotene 20 µM | 9,414 | 4,693 | *** | 0,0006 |

**Appendix table S21**: Mean values and significance levels for Figure 4 panel B (significant differences only)

| **Comparison** | **Mean 1** | **Mean 2** | **Summary** | **Adjusted P Value** |
|---|---|---|---|---|
| | | | | |
| 0:DMSO vs. 0:Tazarotene 10 µM | 2,984 | 11,07 | **** | <0,0001 |
| 0:DMSO vs. 0:Tazarotene 10 µM Bexarotene 20µM | 2,984 | 16,14 | **** | <0,0001 |
| 0:DMSO vs. 1:Tazarotene 10 µM | 2,984 | 11,93 | **** | <0,0001 |
| 0:DMSO vs. 1:Tazarotene 10 µM Bexarotene 20µM | 2,984 | 14,02 | **** | <0,0001 |
| 0:DMSO vs. 5:Tazarotene 10 µM | 2,984 | 12,7 | **** | <0,0001 |
| 0:DMSO vs. 5:Bexarotene 20 µM | 2,984 | 7,002 | * | 0,0176 |
| 0:DMSO vs. 5:Tazarotene 10 µM Bexarotene 20µM | 2,984 | 11,86 | **** | <0,0001 |
| 0:DMSO vs. 10:Tazarotene 10 µM | 2,984 | 10,11 | **** | <0,0001 |

| | | | | |
|---|---|---|---|---|
| 0:DMSO vs. 10:Tazarotene 10 µM Bexarotene 20µM | 2,984 | 7,54 | *** | 0,0005 |
| 0:DMSO vs. 20:DMSO | 2,984 | 5,528 | * | 0,0336 |
| 0:DMSO vs. 20:Tazarotene 10 µM Bexarotene 20µM | 2,984 | 6,49 | * | 0,0337 |
| 0:Tazarotene 10 µM vs. 0:Bexarotene 20 µM | 11,07 | 5,085 | *** | 0,0002 |
| 0:Tazarotene 10 µM vs. 0:Tazarotene 10 µM Bexarotene 20µM | 11,07 | 16,14 | ** | 0,0013 |
| 0:Tazarotene 10 µM vs. 1:DMSO | 11,07 | 4,131 | **** | <0,0001 |
| 0:Tazarotene 10 µM vs. 1:Bexarotene 20 µM | 11,07 | 5,477 | *** | 0,0007 |
| 0:Tazarotene 10 µM vs. 5:DMSO | 11,07 | 4,441 | **** | <0,0001 |
| 0:Tazarotene 10 µM vs. 10:DMSO | 11,07 | 5,317 | **** | <0,0001 |
| 0:Tazarotene 10 µM vs. 10:Bexarotene 20 µM | 11,07 | 6,532 | * | 0,0197 |
| 0:Tazarotene 10 µM vs. 20:DMSO | 11,07 | 5,528 | **** | <0,0001 |
| 0:Tazarotene 10 µM vs. 20:Tazarotene 10 µM | 11,07 | 6,067 | *** | 0,0007 |
| 0:Tazarotene 10 µM vs. 20:Bexarotene 20 µM | 11,07 | 6,448 | * | 0,0154 |
| 0:Tazarotene 10 µM vs. 20:Tazarotene 10 µM Bexarotene 20µM | 11,07 | 6,49 | ** | 0,007 |
| 0:Bexarotene 20 µM vs. 0:Tazarotene 10 µM Bexarotene 20µM | 5,085 | 16,14 | **** | <0,0001 |
| 0:Bexarotene 20 µM vs. 1:Tazarotene 10 µM | 5,085 | 11,93 | **** | <0,0001 |
| 0:Bexarotene 20 µM vs. 1:Tazarotene 10 µM Bexarotene 20µM | 5,085 | 14,02 | **** | <0,0001 |
| 0:Bexarotene 20 µM vs. 5:Tazarotene 10 µM | 5,085 | 12,7 | **** | <0,0001 |
| 0:Bexarotene 20 µM vs. 5:Tazarotene 10 µM Bexarotene 20µM | 5,085 | 11,86 | **** | <0,0001 |
| 0:Bexarotene 20 µM vs. 10:Tazarotene 10 µM | 5,085 | 10,11 | ** | 0,0044 |
| 0:Tazarotene 10 µM Bexarotene 20µM vs. 1:DMSO | 16,14 | 4,131 | **** | <0,0001 |
| 0:Tazarotene 10 µM Bexarotene 20µM vs. 1:Tazarotene 10 µM | 16,14 | 11,93 | * | 0,0226 |
| 0:Tazarotene 10 µM Bexarotene 20µM vs. 1:Bexarotene 20 µM | 16,14 | 5,477 | **** | <0,0001 |
| 0:Tazarotene 10 µM Bexarotene 20µM vs. 5:DMSO | 16,14 | 4,441 | **** | <0,0001 |
| 0:Tazarotene 10 µM Bexarotene 20µM vs. 5:Tazarotene 10 µM Bexarotene 20µM | 16,14 | 11,86 | * | 0,0321 |
| 0:Tazarotene 10 µM Bexarotene 20µM vs. 10:DMSO | 16,14 | 5,317 | **** | <0,0001 |
| 0:Tazarotene 10 µM Bexarotene 20µM vs. 10:Tazarotene 10 µM | 16,14 | 10,11 | **** | <0,0001 |
| 0:Tazarotene 10 µM Bexarotene 20µM vs. 10:Bexarotene 20 µM | 16,14 | 6,532 | **** | <0,0001 |
| 0:Tazarotene 10 µM Bexarotene 20µM vs. 10:Tazarotene 10 µM Bexarotene 20µM | 16,14 | 7,54 | **** | <0,0001 |

| | | | | |
|---|---|---|---|---|
| 0:Tazarotene 10 µM Bexarotene 20µM vs. 20:DMSO | 16,14 | 5,528 | **** | <0,0001 |
| 0:Tazarotene 10 µM Bexarotene 20µM vs. 20:Tazarotene 10 µM | 16,14 | 6,067 | **** | <0,0001 |
| 0:Tazarotene 10 µM Bexarotene 20µM vs. 20:Bexarotene 20 µM | 16,14 | 6,448 | **** | <0,0001 |
| 0:Tazarotene 10 µM Bexarotene 20µM vs. 20:Tazarotene 10 µM Bexarotene 20µM | 16,14 | 6,49 | **** | <0,0001 |
| 1:DMSO vs. 1:Tazarotene 10 µM | 4,131 | 11,93 | **** | <0,0001 |
| 1:DMSO vs. 1:Tazarotene 10 µM Bexarotene 20µM | 4,131 | 14,02 | **** | <0,0001 |
| 1:DMSO vs. 5:Tazarotene 10 µM | 4,131 | 12,7 | **** | <0,0001 |
| 1:DMSO vs. 5:Tazarotene 10 µM Bexarotene 20µM | 4,131 | 11,86 | **** | <0,0001 |
| 1:DMSO vs. 10:Tazarotene 10 µM | 4,131 | 10,11 | **** | <0,0001 |
| 1:DMSO vs. 10:Tazarotene 10 µM Bexarotene 20µM | 4,131 | 7,54 | * | 0,0464 |
| 1:Tazarotene 10 µM vs. 1:Bexarotene 20 µM | 11,93 | 5,477 | **** | <0,0001 |
| 1:Tazarotene 10 µM vs. 5:DMSO | 11,93 | 4,441 | **** | <0,0001 |
| 1:Tazarotene 10 µM vs. 5:Bexarotene 20 µM | 11,93 | 7,002 | ** | 0,0061 |
| 1:Tazarotene 10 µM vs. 10:DMSO | 11,93 | 5,317 | **** | <0,0001 |
| 1:Tazarotene 10 µM vs. 10:Bexarotene 20 µM | 11,93 | 6,532 | ** | 0,0013 |
| 1:Tazarotene 10 µM vs. 10:Tazarotene 10 µM Bexarotene 20µM | 11,93 | 7,54 | * | 0,0131 |
| 1:Tazarotene 10 µM vs. 20:DMSO | 11,93 | 5,528 | **** | <0,0001 |
| 1:Tazarotene 10 µM vs. 20:Tazarotene 10 µM | 11,93 | 6,067 | **** | <0,0001 |
| 1:Tazarotene 10 µM vs. 20:Bexarotene 20 µM | 11,93 | 6,448 | *** | 0,001 |
| 1:Tazarotene 10 µM vs. 20:Tazarotene 10 µM Bexarotene 20µM | 11,93 | 6,49 | *** | 0,0003 |
| 1:Bexarotene 20 µM vs. 1:Tazarotene 10 µM Bexarotene 20µM | 5,477 | 14,02 | **** | <0,0001 |
| 1:Bexarotene 20 µM vs. 5:Tazarotene 10 µM | 5,477 | 12,7 | **** | <0,0001 |
| 1:Bexarotene 20 µM vs. 5:Tazarotene 10 µM Bexarotene 20µM | 5,477 | 11,86 | **** | <0,0001 |
| 1:Bexarotene 20 µM vs. 10:Tazarotene 10 µM | 5,477 | 10,11 | * | 0,0147 |
| 1:Tazarotene 10 µM Bexarotene 20µM vs. 5:DMSO | 14,02 | 4,441 | **** | <0,0001 |
| 1:Tazarotene 10 µM Bexarotene 20µM vs. 5:Bexarotene 20 µM | 14,02 | 7,002 | **** | <0,0001 |
| 1:Tazarotene 10 µM Bexarotene 20µM vs. 10:DMSO | 14,02 | 5,317 | **** | <0,0001 |
| 1:Tazarotene 10 µM Bexarotene 20µM vs. 10:Bexarotene 20 µM | 14,02 | 6,532 | **** | <0,0001 |
| 1:Tazarotene 10 µM Bexarotene 20µM vs. 10:Tazarotene 10 µM Bexarotene 20µM | 14,02 | 7,54 | **** | <0,0001 |

| | | | | |
|---|---|---|---|---|
| 1:Tazarotene 10 µM<br>Bexarotene 20µM vs. 20:DMSO | 14,02 | 5,528 | **** | <0,0001 |
| 1:Tazarotene 10 µM<br>Bexarotene 20µM vs. 20:Tazarotene 10 µM | 14,02 | 6,067 | **** | <0,0001 |
| 1:Tazarotene 10 µM<br>Bexarotene 20µM vs. 20:Bexarotene 20 µM | 14,02 | 6,448 | **** | <0,0001 |
| 1:Tazarotene 10 µM<br>Bexarotene 20µM vs. 20:Tazarotene 10 µM<br>Bexarotene 20µM | 14,02 | 6,49 | **** | <0,0001 |
| 5:DMSO vs. 5:Tazarotene 10 µM | 4,441 | 12,7 | **** | <0,0001 |
| 5:DMSO vs. 5:Tazarotene 10 µM<br>Bexarotene 20µM | 4,441 | 11,86 | **** | <0,0001 |
| 5:DMSO vs. 10:Tazarotene 10 µM | 4,441 | 10,11 | **** | <0,0001 |
| 5:Tazarotene 10 µM vs. 5:Bexarotene 20 µM | 12,7 | 7,002 | *** | 0,0005 |
| 5:Tazarotene 10 µM vs. 10:DMSO | 12,7 | 5,317 | **** | <0,0001 |
| 5:Tazarotene 10 µM vs. 10:Bexarotene 20 µM | 12,7 | 6,532 | **** | <0,0001 |
| 5:Tazarotene 10 µM vs. 10:Tazarotene 10 µM<br>Bexarotene 20µM | 12,7 | 7,54 | *** | 0,0009 |
| 5:Tazarotene 10 µM vs. 20:DMSO | 12,7 | 5,528 | **** | <0,0001 |
| 5:Tazarotene 10 µM vs. 20:Tazarotene 10 µM | 12,7 | 6,067 | **** | <0,0001 |
| 5:Tazarotene 10 µM vs. 20:Bexarotene 20 µM | 12,7 | 6,448 | **** | <0,0001 |
| 5:Tazarotene 10 µM vs. 20:Tazarotene 10 µM<br>Bexarotene 20µM | 12,7 | 6,49 | **** | <0,0001 |
| 5:Bexarotene 20 µM vs. 5:Tazarotene 10 µM<br>Bexarotene 20µM | 7,002 | 11,86 | * | 0,013 |
| 5:Tazarotene 10 µM<br>Bexarotene 20µM vs. 10:DMSO | 11,86 | 5,317 | **** | <0,0001 |
| 5:Tazarotene 10 µM<br>Bexarotene 20µM vs. 10:Bexarotene 20 µM | 11,86 | 6,532 | ** | 0,0032 |
| 5:Tazarotene 10 µM<br>Bexarotene 20µM vs. 10:Tazarotene 10 µM<br>Bexarotene 20µM | 11,86 | 7,54 | * | 0,0279 |
| 5:Tazarotene 10 µM<br>Bexarotene 20µM vs. 20:DMSO | 11,86 | 5,528 | **** | <0,0001 |
| 5:Tazarotene 10 µM<br>Bexarotene 20µM vs. 20:Tazarotene 10 µM | 11,86 | 6,067 | **** | <0,0001 |
| 5:Tazarotene 10 µM<br>Bexarotene 20µM vs. 20:Bexarotene 20 µM | 11,86 | 6,448 | ** | 0,0024 |
| 5:Tazarotene 10 µM<br>Bexarotene 20µM vs. 20:Tazarotene 10 µM<br>Bexarotene 20µM | 11,86 | 6,49 | *** | 0,001 |
| 10:DMSO vs. 10:Tazarotene 10 µM | 5,317 | 10,11 | **** | <0,0001 |
| 10:Tazarotene 10 µM vs. 20:DMSO | 10,11 | 5,528 | *** | 0,0001 |
| 10:Tazarotene 10 µM vs. 20:Tazarotene 10 µM | 10,11 | 6,067 | * | 0,0203 |

**Appendix table S22**: Mean values and significance levels for Figure 5 panel A (significant differences only)

| Comparison | Mean 1 | Mean 2 | Summary | Adjusted P Value |
|---|---|---|---|---|
| | | | | |
| control DMSO vs. MSD DMSO | -0,01671 | -0,1948 | * | 0,0411 |
| MSD DMSO vs. control taz/bex | -0,1948 | 0,2035 | ** | 0,0074 |
| MSD DMSO vs. MSD taz/bex | -0,1948 | 0,0391 | * | 0,0404 |

**Appendix table S23**: Mean values and significance levels for Figure 5 panel C (significant differences only)

| Comparison | Mean 1 | Mean 2 | Summary | Adjusted P Value |
|---|---|---|---|---|
| | | | | |
| control DMSO vs. MSD taz/bex | 0,1004 | -0,245 | * | 0,0107 |
| MSD DMSO vs. MSD taz/bex | 0,1558 | -0,245 | ** | 0,0045 |
| control taz/bex vs. MSD taz/bex | 0,006763 | -0,245 | * | 0,0478 |

**Appendix table S24**: Mean values and significance levels for Figure 6 panel A (significant differences only)

| Comparison | Mean 1 | Mean 2 | Summary | Adjusted P Value |
|---|---|---|---|---|
| | | | | |
| 0/0 vs 10/0 | 10,13 | 44 | * | 0,0402 |
| 0/0 vs.10/20 | 10,13 | 77,65 | *** | 0,0007 |
| 10/0 vs. 10/20 | 44 | 77,65 | * | 0,0414 |
| 20/0 vs. 10/20 | 20,91 | 77,65 | ** | 0,0022 |

**Appendix table S25**: Mean values and significance levels for Figure 6 panel B (significant differences only)

| Comparison | Mean 1 | Mean 2 | Summary | Adjusted P Value |
|---|---|---|---|---|
| | | | | |
| MSDi | | | | |
| 0 vs. 10 | 4,693 | 14,64 | *** | 0,0001 |
| 0 vs. 25 | 4,693 | 21,09 | **** | <0,0001 |
| 0 vs. 50 | 4,693 | 19,18 | **** | <0,0001 |
| 10 vs. 25 | 14,64 | 21,09 | ** | 0,0048 |

**Appendix table S26**: Mean values and significance levels for Figure 6 panel C (significant differences only)

| Comparison | Mean 1 | Mean 2 | Summary | Adjusted P Value |
|---|---|---|---|---|
| | | | | |
| MSD FGE G247R | | | | |
| 0 vs. 6 | 15,88 | 113,9 | **** | <0,0001 |
| 0 vs. 14 | 15,88 | 181,2 | **** | <0,0001 |
| 0 vs. 21 | 15,88 | 168,3 | **** | <0,0001 |
| 6 vs. 14 | 113,9 | 181,2 | **** | <0,0001 |
| 6 vs. 21 | 113,9 | 168,3 | *** | 0,0003 |
| ARPE 19 SUMF1 +/+ | | | | |
| 0 vs. 6 | 263,5 | 401,1 | **** | <0,0001 |
| 0 vs. 14 | 263,5 | 401,4 | **** | <0,0001 |
| 0 vs. 21 | 263,5 | 369,8 | **** | <0,0001 |
| 6 vs. 21 | 401,1 | 369,8 | * | 0,0421 |
| 14 vs. 21 | 401,4 | 369,8 | * | 0,0399 |

**Appendix table S27**: Mean values and significance levels for Figure 6 panel D (significant differences only)

| Comparison | Mean 1 | Mean 2 | Summary | Adjusted P Value |
|---|---|---|---|---|
| | | | | |
| p.G247R 0/0 vs. 10/20 | 15,97 | 73,76 | * | 0,0124 |

**Appendix table S28**: Mean values and significance levels for Figure 7 panel A (significant differences only)

| Comparison | Mean 1 | Mean 2 | Summary | Adjusted P Value |
|---|---|---|---|---|
| | | | | |
| DMSO0 vs. DMSO1 | 100 | 51,07 | **** | <0,0001 |
| DMSO0 vs. DMSO2 | 100 | 24,04 | **** | <0,0001 |
| DMSO0 vs. DMSO3 | 100 | 13,15 | **** | <0,0001 |
| DMSO0 vs. DMSO4 | 100 | 7,216 | **** | <0,0001 |
| DMSO0 vs. TB1 | 100 | 80,88 | * | 0,0427 |
| DMSO0 vs. TB2 | 100 | 50,37 | **** | <0,0001 |
| DMSO0 vs. TB3 | 100 | 27,69 | **** | <0,0001 |
| DMSO0 vs. TB4 | 100 | 16,98 | **** | <0,0001 |
| DMSO1 vs. DMSO2 | 51,07 | 24,04 | ** | 0,0017 |
| DMSO1 vs. DMSO3 | 51,07 | 13,15 | **** | <0,0001 |

| | | | | |
|---|---|---|---|---|
| DMSO1 vs. DMSO4 | 51,07 | 7,216 | **** | <0,0001 |
| DMSO1 vs. TB0 | 51,07 | 100 | **** | <0,0001 |
| DMSO1 vs. TB1 | 51,07 | 80,88 | *** | 0,0005 |
| DMSO1 vs. TB3 | 51,07 | 27,69 | ** | 0,0077 |
| DMSO1 vs. TB4 | 51,07 | 16,98 | **** | <0,0001 |
| DMSO2 vs. TB0 | 24,04 | 100 | **** | <0,0001 |
| DMSO2 vs. TB1 | 24,04 | 80,88 | **** | <0,0001 |
| DMSO2 vs. TB2 | 24,04 | 50,37 | ** | 0,0023 |
| DMSO3 vs. TB0 | 13,15 | 100 | **** | <0,0001 |
| DMSO3 vs. TB1 | 13,15 | 80,88 | **** | <0,0001 |
| DMSO3 vs. TB2 | 13,15 | 50,37 | **** | <0,0001 |
| DMSO4 vs. TB0 | 7,216 | 100 | **** | <0,0001 |
| DMSO4 vs. TB1 | 7,216 | 80,88 | **** | <0,0001 |
| DMSO4 vs. TB2 | 7,216 | 50,37 | **** | <0,0001 |
| DMSO4 vs. TB3 | 7,216 | 27,69 | * | 0,0251 |
| TB0 vs. TB1 | 100 | 80,88 | * | 0,0427 |
| TB0 vs. TB2 | 100 | 50,37 | **** | <0,0001 |
| TB0 vs. TB3 | 100 | 27,69 | **** | <0,0001 |
| TB0 vs. TB4 | 100 | 16,98 | **** | <0,0001 |
| TB1 vs. TB2 | 80,88 | 50,37 | *** | 0,0004 |
| TB1 vs. TB3 | 80,88 | 27,69 | **** | <0,0001 |
| TB1 vs. TB4 | 80,88 | 16,98 | **** | <0,0001 |
| TB2 vs. TB3 | 50,37 | 27,69 | * | 0,0102 |
| TB2 vs. TB4 | 50,37 | 16,98 | *** | 0,0001 |

**Appendix table S29**: Mean values and significance levels for Figure 7 panel B (significant differences only)

| **Comparison** | **Mean 1** | **Mean 2** | **Summary** | **Adjusted P Value** |
|---|---|---|---|---|
| | | | | |
| DMSO0 vs. DMSO1 | 100 | 70,59 | *** | 0,0002 |
| DMSO0 vs. Taz/Bex1 | 100 | 79,03 | ** | 0,0086 |
| DMSO0 vs. DMSO2 | 100 | 43,51 | **** | <0,0001 |
| DMSO0 vs. Taz/Bex2 | 100 | 58,05 | **** | <0,0001 |
| DMSO0 vs. DMSO3 | 100 | 30,77 | **** | <0,0001 |
| DMSO0 vs. Taz/Bex3 | 100 | 46,25 | **** | <0,0001 |
| DMSO0 vs. DMSO4 | 100 | 19,43 | **** | <0,0001 |
| DMSO0 vs. Taz/Bex4 | 100 | 43,13 | **** | <0,0001 |
| Taz/Bex0 vs. DMSO1 | 100 | 70,59 | *** | 0,0002 |
| Taz/Bex0 vs. Taz/Bex1 | 100 | 79,03 | ** | 0,0086 |
| Taz/Bex0 vs. DMSO2 | 100 | 43,51 | **** | <0,0001 |
| Taz/Bex0 vs. Taz/Bex2 | 100 | 58,05 | **** | <0,0001 |
| Taz/Bex0 vs. DMSO3 | 100 | 30,77 | **** | <0,0001 |
| Taz/Bex0 vs. Taz/Bex3 | 100 | 46,25 | **** | <0,0001 |
| Taz/Bex0 vs. DMSO4 | 100 | 19,43 | **** | <0,0001 |

| | | | | |
|---|---|---|---|---|
| Taz/Bex0 vs. Taz/Bex4 | 100 | 43,13 | **** | <0,0001 |
| DMSO1 vs. DMSO2 | 70,59 | 43,51 | *** | 0,0005 |
| DMSO1 vs. DMSO3 | 70,59 | 30,77 | **** | <0,0001 |
| DMSO1 vs. Taz/Bex3 | 70,59 | 46,25 | ** | 0,0018 |
| DMSO1 vs. DMSO4 | 70,59 | 19,43 | **** | <0,0001 |
| DMSO1 vs. Taz/Bex4 | 70,59 | 43,13 | *** | 0,0004 |
| Taz/Bex1 vs. DMSO2 | 79,03 | 43,51 | **** | <0,0001 |
| Taz/Bex1 vs. Taz/Bex2 | 79,03 | 58,05 | ** | 0,0085 |
| Taz/Bex vs. DMSO3 | 79,03 | 30,77 | **** | <0,0001 |
| Taz/Bex1 vs. Taz/Bex3 | 79,03 | 46,25 | **** | <0,0001 |
| Taz/Bex1 vs. DMSO4 | 79,03 | 19,43 | **** | <0,0001 |
| Taz/Bex1 vs. Taz/Bex4 | 79,03 | 43,13 | **** | <0,0001 |
| DMSO2 vs. DMSO4 | 43,51 | 19,43 | ** | 0,0021 |
| Taz/Bex2 vs. DMSO3 | 58,05 | 30,77 | *** | 0,0005 |
| Taz/Bex2 vs. DMSO4 | 58,05 | 19,43 | **** | <0,0001 |
| Taz/Bex3 vs. DMSO4 | 46,25 | 19,43 | *** | 0,0006 |
| DMSO4 vs. Taz/Bex4 | 19,43 | 43,13 | ** | 0,0025 |

**Appendix table S30**: Mean values and significance levels for Figure 7 panel C (significant differences only)

| **Comparison** | **Mean 1** | **Mean 2** | **Summary** | **Adjusted P Value** |
|---|---|---|---|---|
| | | | | |
| DMSO0 vs. DMSO1 | 100 | 60,06 | ** | 0,0068 |
| DMSO0 vs. DMSO2 | 100 | 43,42 | *** | 0,0001 |
| DMSO0 vs. Taz/Bex2 | 100 | 59,69 | ** | 0,0062 |
| DMSO0 vs. DMSO3 | 100 | 29,36 | **** | <0,0001 |
| DMSO0 vs. Taz/Bex3 | 100 | 43,26 | *** | 0,0001 |
| DMSO0 vs. DMSO4 | 100 | 18,16 | **** | <0,0001 |
| DMSO0 vs. Taz/Bex4 | 100 | 33,55 | **** | <0,0001 |
| Taz/Bex0 vs. DMSO1 | 100 | 60,06 | ** | 0,0068 |
| Taz/Bex0 vs. DMSO2 | 100 | 43,42 | *** | 0,0001 |
| Taz/Bex0 vs. Taz/Bex2 | 100 | 59,69 | ** | 0,0062 |
| Taz/Bex0 vs. DMSO3 | 100 | 29,36 | **** | <0,0001 |
| Taz/Bex0 vs. Taz/Bex3 | 100 | 43,26 | *** | 0,0001 |
| Taz/Bex0 vs. DMSO4 | 100 | 18,16 | **** | <0,0001 |
| Taz/Bex0 vs. Taz/Bex4 | 100 | 33,55 | **** | <0,0001 |
| DMSO1 vs. DMSO4 | 60,06 | 18,16 | ** | 0,0042 |
| Taz/Bex1 vs. DMSO2 | 78,12 | 43,42 | * | 0,0241 |
| Taz/Bex1 vs. DMSO3 | 78,12 | 29,36 | *** | 0,0008 |
| Taz/Bex1 vs. Taz/Bex3 | 78,12 | 43,26 | * | 0,0232 |
| Taz/Bex1 vs. DMSO4 | 78,12 | 18,16 | **** | <0,0001 |
| Taz/Bex1 vs. Taz/Bex4 | 78,12 | 33,55 | ** | 0,0022 |
| Taz/Bex2 vs. DMSO4 | 59,69 | 18,16 | ** | 0,0046 |

**Appendix table S31**: Mean values and significance levels for EV Figure 1 panel A (significant differences only)

| Comparison | Mean 1 | Mean 2 | Summary | Adjusted P Value |
|---|---|---|---|---|
| | | | | |
| neg. contr. vs. Tazarotene | 8,696 | 28,37 | **** | <0,0001 |
| neg. contr. vs. pos. contr. | 8,696 | 53,63 | **** | <0,0001 |
| Tazarotene vs. Clindamycin | 28,37 | 10,46 | **** | <0,0001 |
| Tazarotene vs. Vorinostat | 28,37 | 7,913 | **** | <0,0001 |
| Tazarotene vs. Asenapine | 28,37 | 9,489 | **** | <0,0001 |
| Tazarotene vs. pos. contr. | 28,37 | 53,63 | **** | <0,0001 |
| Clindamycin vs. pos. contr. | 10,46 | 53,63 | **** | <0,0001 |
| Vorinostat vs. pos. contr. | 7,913 | 53,63 | **** | <0,0001 |
| Asenapine vs. pos. contr. | 9,489 | 53,63 | **** | <0,0001 |

**Appendix table S32**: Mean values and significance levels for EV Figure 1 panel B (significant differences only)

| Comparison | Mean 1 | Mean 2 | Summary | Adjusted P Value |
|---|---|---|---|---|
| | | | | |
| neg. contr. vs. Tazarotene | 1,307 | 2,782 | ** | 0,0025 |
| Tazarotene vs. Clindamycin | 2,782 | 1,616 | * | 0,0223 |
| Tazarotene vs. Vorinostat | 2,782 | 1,445 | ** | 0,0068 |

**Appendix table S33**: Mean values and significance levels for EV Figure 1 panel C (significant differences only)

| Comparison | Mean 1 | Mean 2 | Summary | Adjusted P Value |
|---|---|---|---|---|
| | | | | |
| neg. contr. vs. Tazarotene | 1,517 | 3,125 | ** | 0,0055 |
| neg. contr. vs. pos. contr. | 1,517 | 2,766 | * | 0,0446 |
| Tazarotene vs. Vorinostat | 3,125 | 1,315 | ** | 0,0016 |
| Vorinostat vs. pos. contr. | 1,315 | 2,766 | * | 0,0141 |

**Appendix table S34**: Mean values and significance levels for EV Figure 2 panel E (significant differences only)

| Comparison | Mean 1 | Mean 2 | Summary | Adjusted P Value |
|---|---|---|---|---|

| | | | | |
|---|---|---|---|---|
| | | | | |
| 0/0 vs 0.25/0.5 | 13,43 | 21,47 | * | 0,0252 |
| 0/0 vs 0.5/1 | 13,43 | 26,38 | *** | 0,0003 |
| 0/0 vs 1/2 | 13,43 | 39,13 | **** | <0,0001 |
| 0/0 vs 2.5/5 | 13,43 | 74,57 | **** | <0,0001 |
| 0.1/0.2 vs 0.5/1 | 18,71 | 26,38 | * | 0,0352 |
| 0.1/0.2 vs 1/2 | 18,71 | 39,13 | **** | <0,0001 |
| 0.1/0.2 vs 2.5/5 | 18,71 | 74,57 | **** | <0,0001 |
| 0.25/0.5 vs 1/2 | 21,47 | 39,13 | **** | <0,0001 |
| 0.25/0.5 vs 2.5/5 | 21,47 | 74,57 | **** | <0,0001 |
| 0.5/1 vs 1/2 | 26,38 | 39,13 | *** | 0,0003 |
| 0.5/1 vs 2.5/5 | 26,38 | 74,57 | **** | <0,0001 |
| 1/2 vs 2.5/5 | 39,13 | 74,57 | **** | <0,0001 |

**Appendix table S35**: Mean values and significance levels for EV Figure 2 panel F (significant differences only)

| **Comparison** | **Mean 1** | **Mean 2** | **Summary** | **Adjusted P Value** |
|---|---|---|---|---|
| | | | | |
| p.G247R - vs. + | 17,0 | 49,2 | **** | <0,0001 |
| p.G263V - vs. + | 130,5 | 282,4 | **** | <0,0001 |
| p.A279V - vs. + | 132,0 | 227,8 | *** | 0,0007 |
| p.R349W - vs. + | 20,5 | 39,8 | * | 0,0152 |
| p.A279V - vs. + | 96,0 | 120,6 | ** | 0,001 |
| p.R349W - vs. + | 27,9 | 67,5 | ** | 0,0015 |
| p.G247R - vs. + | 18,1 | 60,3 | * | 0,0132 |

**Appendix table S36**: Mean values and significance levels for EV Figure 3 panel A (significant differences only)

| **Comparison** | **Mean 1** | **Mean 2** | **Summary** | **Adjusted P Value** |
|---|---|---|---|---|
| | | | | |
| RARRES1 | | | | |
| ctrl- vs. ctrl+ | 0,5626 | 73,99 | **** | <0,0001 |
| ctrl- vs. MSD+ | 0,5626 | 65,22 | **** | <0,0001 |
| MSD- vs. ctrl+ | 1,696 | 73,99 | **** | <0,0001 |
| MSD- vs. MSD+ | 1,696 | 65,22 | **** | <0,0001 |
| | | | | |
| CYP26B1 | | | | |
| ctrl- vs. ctrl+ | 7,032 | 406,7 | ** | 0,0033 |
| ctrl- vs. MSD+ | 7,032 | 309,4 | * | 0,0209 |
| MSD- vs. ctrl+ | 4,105 | 406,7 | ** | 0,0021 |

| | | | | |
|---|---|---|---|---|
| MSD- vs. MSD+ | 4,105 | 309,4 | * | 0,014 |

**Appendix table S37**: Mean values and significance levels for EV Figure 3 panel B (significant differences only)

| Comparison | Mean 1 | Mean 2 | Summary | Adjusted P Value |
|---|---|---|---|---|
| | | | | |
| SUMF1 | | | | |
| MSD- vs. MSD+ | 11,21 | 7,926 | * | 0,0213 |

**Appendix table S38**: Mean values and significance levels for EV Figure 3 panel C (significant differences only)

| Comparison | Mean 1 | Mean 2 | Summary | Adjusted P Value |
|---|---|---|---|---|
| | | | | |
| ARSB | | | | |
| ctrl- vs. ctrl+ | 11,16 | 7,122 | * | 0,0278 |
| | | | | |
| STS | | | | |
| ctrl- vs. ctrl+ | 14,12 | 6,614 | * | 0,0331 |
| MSD- vs. ctrl+ | 15,14 | 6,614 | * | 0,0101 |
| MSD- vs. MSD+ | 15,14 | 7,528 | * | 0,0165 |
| | | | | |
| SULF1 | | | | |
| ctrl- vs. ctrl+ | 89,04 | 793,3 | **** | <0,0001 |
| ctrl- vs. MSD+ | 89,04 | 826,2 | **** | <0,0001 |
| MSD- vs. ctrl+ | 121,3 | 793,3 | **** | <0,0001 |
| MSD- vs. MSD+ | 121,3 | 826,2 | **** | <0,0001 |

**Appendix table S39**: Mean values and significance levels for EV Figure 4 panel A (significant differences only)

| Comparison | Mean 1 | Mean 2 | Summary | Adjusted P Value |
|---|---|---|---|---|
| | | | | |
| control DMSO vs. MSD DMSO | 0,2 | 0,2 | ns | 0,9972 |
| control DMSO vs. control taz/bex | 0,2085 | -0,1749 | **** | <0,0001 |
| control DMSO vs. MSD taz/bex | 0,2085 | -0,2289 | **** | <0,0001 |
| MSD DMSO vs. control taz/bex | 0,2009 | -0,1749 | **** | <0,0001 |
| MSD DMSO vs. MSD taz/bex | 0,2009 | -0,2289 | **** | <0,0001 |
| control taz/bex vs. MSD taz/bex | -0,1749 | -0,2289 | ns | 0,518 |

**Appendix table S40**: Mean values and significance levels for EV Figure 4 panel C (significant differences only)

| Comparison | Mean 1 | Mean 2 | Summary | Adjusted P Value |
|---|---|---|---|---|
| | | | | |
| control DMSO vs. control taz/bex | -0,1835 | 0,1222 | ** | 0,0081 |
| control DMSO vs. MSD taz/bex | -0,1835 | 0,1664 | * | 0,0147 |
| MSD DMSO vs. control taz/bex | -0,1153 | 0,1222 | * | 0,0215 |
| MSD DMSO vs. MSD taz/bex | -0,1153 | 0,1664 | * | 0,0275 |

**Appendix table S41**: Mean values and significance levels for MSD fibroblast cell count comparison (Appendix fig. S5A, significant differences only)

| Comparison | Mean 1 | Mean 2 | Summary | Adjusted P Value |
|---|---|---|---|---|
| | | | | |
| 0d:MSD DMSO low vs. 3d:MSD DMSO low | 450000 | 1693000 | ** | 0,008 |
| 0d:MSD DMSO low vs. 3d:MSD DMSO medium | 450000 | 2787167 | **** | <0,0001 |
| 0d:MSD DMSO low vs. 3d:MSD DMSO high | 450000 | 3110833 | **** | <0,0001 |
| 0d:MSD DMSO low vs. 3d:MSD Taz | 450000 | 2204167 | **** | <0,0001 |
| 0d:MSD DMSO low vs. 3d:MSD Bex | 450000 | 2237500 | **** | <0,0001 |
| 0d:MSD DMSO low vs. 3d:MSD Taz/Bex | 450000 | 1879167 | *** | 0,0007 |
| 0d:MSD DMSO low vs. 6d:MSD DMSO low | 450000 | 3514000 | **** | <0,0001 |
| 0d:MSD DMSO low vs. 6d:MSD DMSO medium | 450000 | 3279333 | **** | <0,0001 |
| 0d:MSD DMSO low vs. 6d:MSD DMSO high | 450000 | 3255167 | **** | <0,0001 |
| 0d:MSD DMSO low vs. 6d:MSD Taz | 450000 | 2434333 | **** | <0,0001 |
| 0d:MSD DMSO low vs. 6d:MSD Bex | 450000 | 2474333 | **** | <0,0001 |
| 0d:MSD DMSO low vs. 6d:MSD Taz/Bex | 450000 | 1977167 | *** | 0,0002 |
| 0d:MSD DMSO low vs. 9d:MSD DMSO low | 450000 | 3909667 | **** | <0,0001 |
| 0d:MSD DMSO low vs. 9d:MSD DMSO medium | 450000 | 3771250 | **** | <0,0001 |
| 0d:MSD DMSO low vs. 9d:MSD DMSO high | 450000 | 3527833 | **** | <0,0001 |
| 0d:MSD DMSO low vs. 9d:MSD Taz | 450000 | 2496667 | **** | <0,0001 |
| 0d:MSD DMSO low vs. 9d:MSD Bex | 450000 | 2628833 | **** | <0,0001 |
| 0d:MSD DMSO low vs. 9d:MSD Taz/Bex | 450000 | 2005917 | *** | 0,0001 |
| 0d:MSD DMSO medium vs. 3d:MSD DMSO medium | 900000 | 2787167 | **** | <0,0001 |
| 0d:MSD DMSO medium vs. 3d:MSD DMSO high | 900000 | 3110833 | **** | <0,0001 |
| 0d:MSD DMSO medium vs. 3d:MSD Taz | 900000 | 2204167 | ** | 0,0037 |
| 0d:MSD DMSO medium vs. 3d:MSD Bex | 900000 | 2237500 | ** | 0,0024 |

| | | | | |
|---|---|---|---|---|
| 0d:MSD DMSO medium vs. 6d:MSD DMSO low | 900000 | 3514000 | **** | <0,0001 |
| 0d:MSD DMSO medium vs. 6d:MSD DMSO medium | 900000 | 3279333 | **** | <0,0001 |
| 0d:MSD DMSO medium vs. 6d:MSD DMSO high | 900000 | 3255167 | **** | <0,0001 |
| 0d:MSD DMSO medium vs. 6d:MSD Taz | 900000 | 2434333 | *** | 0,0001 |
| 0d:MSD DMSO medium vs. 6d:MSD Bex | 900000 | 2474333 | **** | <0,0001 |
| 0d:MSD DMSO medium vs. 9d:MSD DMSO low | 900000 | 3909667 | **** | <0,0001 |
| 0d:MSD DMSO medium vs. 9d:MSD DMSO medium | 900000 | 3771250 | **** | <0,0001 |
| 0d:MSD DMSO medium vs. 9d:MSD DMSO high | 900000 | 3527833 | **** | <0,0001 |
| 0d:MSD DMSO medium vs. 9d:MSD Taz | 900000 | 2496667 | **** | <0,0001 |
| 0d:MSD DMSO medium vs. 9d:MSD Bex | 900000 | 2628833 | **** | <0,0001 |
| 0d:MSD DMSO medium vs. 9d:MSD Taz/Bex | 900000 | 2005917 | * | 0,0389 |
| 0d:MSD DMSO high vs. 3d:MSD DMSO medium | 1350000 | 2787167 | *** | 0,0006 |
| 0d:MSD DMSO high vs. 3d:MSD DMSO high | 1350000 | 3110833 | **** | <0,0001 |
| 0d:MSD DMSO high vs. 6d:MSD DMSO low | 1350000 | 3514000 | **** | <0,0001 |
| 0d:MSD DMSO high vs. 6d:MSD DMSO medium | 1350000 | 3279333 | **** | <0,0001 |
| 0d:MSD DMSO high vs. 6d:MSD DMSO high | 1350000 | 3255167 | **** | <0,0001 |
| 0d:MSD DMSO high vs. 6d:MSD Taz | 1350000 | 2434333 | * | 0,0488 |
| 0d:MSD DMSO high vs. 6d:MSD Bex | 1350000 | 2474333 | * | 0,0318 |
| 0d:MSD DMSO high vs. 9d:MSD DMSO low | 1350000 | 3909667 | **** | <0,0001 |
| 0d:MSD DMSO high vs. 9d:MSD DMSO medium | 1350000 | 3771250 | **** | <0,0001 |
| 0d:MSD DMSO high vs. 9d:MSD DMSO high | 1350000 | 3527833 | **** | <0,0001 |
| 0d:MSD DMSO high vs. 9d:MSD Taz | 1350000 | 2496667 | * | 0,0248 |
| 0d:MSD DMSO high vs. 9d:MSD Bex | 1350000 | 2628833 | ** | 0,0051 |
| 0d:MSD Taz vs. 3d:MSD DMSO medium | 900000 | 2787167 | **** | <0,0001 |
| 0d:MSD Taz vs. 3d:MSD DMSO high | 900000 | 3110833 | **** | <0,0001 |
| 0d:MSD Taz vs. 3d:MSD Taz | 900000 | 2204167 | ** | 0,0037 |
| 0d:MSD Taz vs. 3d:MSD Bex | 900000 | 2237500 | ** | 0,0024 |
| 0d:MSD Taz vs. 6d:MSD DMSO low | 900000 | 3514000 | **** | <0,0001 |
| 0d:MSD Taz vs. 6d:MSD DMSO medium | 900000 | 3279333 | **** | <0,0001 |
| 0d:MSD Taz vs. 6d:MSD DMSO high | 900000 | 3255167 | **** | <0,0001 |
| 0d:MSD Taz vs. 6d:MSD Taz | 900000 | 2434333 | *** | 0,0001 |
| 0d:MSD Taz vs. 6d:MSD Bex | 900000 | 2474333 | **** | <0,0001 |
| 0d:MSD Taz vs. 9d:MSD DMSO low | 900000 | 3909667 | **** | <0,0001 |
| 0d:MSD Taz vs. 9d:MSD DMSO medium | 900000 | 3771250 | **** | <0,0001 |
| 0d:MSD Taz vs. 9d:MSD DMSO high | 900000 | 3527833 | **** | <0,0001 |
| 0d:MSD Taz vs. 9d:MSD Taz | 900000 | 2496667 | **** | <0,0001 |
| 0d:MSD Taz vs. 9d:MSD Bex | 900000 | 2628833 | **** | <0,0001 |
| 0d:MSD Taz vs. 9d:MSD Taz/Bex | 900000 | 2005917 | * | 0,0389 |
| 0d:MSD Bex vs. 3d:MSD DMSO medium | 900000 | 2787167 | **** | <0,0001 |

| | | | | |
|---|---|---|---|---|
| 0d:MSD Bex vs. 3d:MSD DMSO high | 900000 | 3110833 | **** | <0,0001 |
| 0d:MSD Bex vs. 3d:MSD Taz | 900000 | 2204167 | ** | 0,0037 |
| 0d:MSD Bex vs. 3d:MSD Bex | 900000 | 2237500 | ** | 0,0024 |
| 0d:MSD Bex vs. 6d:MSD DMSO low | 900000 | 3514000 | **** | <0,0001 |
| 0d:MSD Bex vs. 6d:MSD DMSO medium | 900000 | 3279333 | **** | <0,0001 |
| 0d:MSD Bex vs. 6d:MSD DMSO high | 900000 | 3255167 | **** | <0,0001 |
| 0d:MSD Bex vs. 6d:MSD Taz | 900000 | 2434333 | *** | 0,0001 |
| 0d:MSD Bex vs. 6d:MSD Bex | 900000 | 2474333 | **** | <0,0001 |
| 0d:MSD Bex vs. 9d:MSD DMSO low | 900000 | 3909667 | **** | <0,0001 |
| 0d:MSD Bex vs. 9d:MSD DMSO medium | 900000 | 3771250 | **** | <0,0001 |
| 0d:MSD Bex vs. 9d:MSD DMSO high | 900000 | 3527833 | **** | <0,0001 |
| 0d:MSD Bex vs. 9d:MSD Taz | 900000 | 2496667 | **** | <0,0001 |
| 0d:MSD Bex vs. 9d:MSD Bex | 900000 | 2628833 | **** | <0,0001 |
| 0d:MSD Bex vs. 9d:MSD Taz/Bex | 900000 | 2005917 | * | 0,0389 |
| 0d:MSD Taz/Bex vs. 3d:MSD DMSO medium | 900000 | 2787167 | **** | <0,0001 |
| 0d:MSD Taz/Bex vs. 3d:MSD DMSO high | 900000 | 3110833 | **** | <0,0001 |
| 0d:MSD Taz/Bex vs. 3d:MSD Taz | 900000 | 2204167 | ** | 0,0037 |
| 0d:MSD Taz/Bex vs. 3d:MSD Bex | 900000 | 2237500 | ** | 0,0024 |
| 0d:MSD Taz/Bex vs. 6d:MSD DMSO low | 900000 | 3514000 | **** | <0,0001 |
| 0d:MSD Taz/Bex vs. 6d:MSD DMSO medium | 900000 | 3279333 | **** | <0,0001 |
| 0d:MSD Taz/Bex vs. 6d:MSD DMSO high | 900000 | 3255167 | **** | <0,0001 |
| 0d:MSD Taz/Bex vs. 6d:MSD Taz | 900000 | 2434333 | *** | 0,0001 |
| 0d:MSD Taz/Bex vs. 6d:MSD Bex | 900000 | 2474333 | **** | <0,0001 |
| 0d:MSD Taz/Bex vs. 9d:MSD DMSO low | 900000 | 3909667 | **** | <0,0001 |
| 0d:MSD Taz/Bex vs. 9d:MSD DMSO medium | 900000 | 3771250 | **** | <0,0001 |
| 0d:MSD Taz/Bex vs. 9d:MSD DMSO high | 900000 | 3527833 | **** | <0,0001 |
| 0d:MSD Taz/Bex vs. 9d:MSD Taz | 900000 | 2496667 | **** | <0,0001 |
| 0d:MSD Taz/Bex vs. 9d:MSD Bex | 900000 | 2628833 | **** | <0,0001 |
| 0d:MSD Taz/Bex vs. 9d:MSD Taz/Bex | 900000 | 2005917 | * | 0,0389 |
| 3d:MSD DMSO low vs. 3d:MSD DMSO medium | 1693000 | 2787167 | * | 0,044 |
| 3d:MSD DMSO low vs. 3d:MSD DMSO high | 1693000 | 3110833 | *** | 0,0008 |
| 3d:MSD DMSO low vs. 6d:MSD DMSO low | 1693000 | 3514000 | **** | <0,0001 |
| 3d:MSD DMSO low vs. 6d:MSD DMSO medium | 1693000 | 3279333 | **** | <0,0001 |
| 3d:MSD DMSO low vs. 6d:MSD DMSO high | 1693000 | 3255167 | **** | <0,0001 |
| 3d:MSD DMSO low vs. 9d:MSD DMSO low | 1693000 | 3909667 | **** | <0,0001 |
| 3d:MSD DMSO low vs. 9d:MSD DMSO medium | 1693000 | 3771250 | **** | <0,0001 |
| 3d:MSD DMSO low vs. 9d:MSD DMSO high | 1693000 | 3527833 | **** | <0,0001 |
| 3d:MSD DMSO medium vs. 9d:MSD DMSO low | 2787167 | 3909667 | * | 0,0325 |
| 3d:MSD DMSO high vs. 3d:MSD Taz/Bex | 3110833 | 1879167 | ** | 0,0092 |
| 3d:MSD DMSO high vs. 6d:MSD Taz/Bex | 3110833 | 1977167 | * | 0,0287 |
| 3d:MSD DMSO high vs. 9d:MSD Taz/Bex | 3110833 | 2005917 | * | 0,0393 |
| 3d:MSD Taz vs. 6d:MSD DMSO low | 2204167 | 3514000 | ** | 0,0034 |
| 3d:MSD Taz vs. 9d:MSD DMSO low | 2204167 | 3909667 | **** | <0,0001 |
| 3d:MSD Taz vs. 9d:MSD DMSO medium | 2204167 | 3771250 | **** | <0,0001 |

| | | | | |
|---|---|---|---|---|
| 3d:MSD Taz vs. 9d:MSD DMSO high | 2204167 | 3527833 | ** | 0,0028 |
| 3d:MSD Bex vs. 6d:MSD DMSO low | 2237500 | 3514000 | ** | 0,0052 |
| 3d:MSD Bex vs. 9d:MSD DMSO low | 2237500 | 3909667 | **** | <0,0001 |
| 3d:MSD Bex vs. 9d:MSD DMSO medium | 2237500 | 3771250 | *** | 0,0001 |
| 3d:MSD Bex vs. 9d:MSD DMSO high | 2237500 | 3527833 | ** | 0,0044 |
| 3d:MSD Taz/Bex vs. 6d:MSD DMSO low | 1879167 | 3514000 | **** | <0,0001 |
| 3d:MSD Taz/Bex vs. 6d:MSD DMSO medium | 1879167 | 3279333 | ** | 0,001 |
| 3d:MSD Taz/Bex vs. 6d:MSD DMSO high | 1879167 | 3255167 | ** | 0,0014 |
| 3d:MSD Taz/Bex vs. 9d:MSD DMSO low | 1879167 | 3909667 | **** | <0,0001 |
| 3d:MSD Taz/Bex vs. 9d:MSD DMSO medium | 1879167 | 3771250 | **** | <0,0001 |
| 3d:MSD Taz/Bex vs. 9d:MSD DMSO high | 1879167 | 3527833 | **** | <0,0001 |
| 6d:MSD DMSO low vs. 6d:MSD Taz/Bex | 3514000 | 1977167 | *** | 0,0001 |
| 6d:MSD DMSO low vs. 9d:MSD Taz/Bex | 3514000 | 2005917 | *** | 0,0002 |
| 6d:MSD DMSO medium vs. 6d:MSD Taz/Bex | 3279333 | 1977167 | ** | 0,0038 |
| 6d:MSD DMSO medium vs. 9d:MSD Taz/Bex | 3279333 | 2005917 | ** | 0,0054 |
| 6d:MSD DMSO high vs. 6d:MSD Taz/Bex | 3255167 | 1977167 | ** | 0,0051 |
| 6d:MSD DMSO high vs. 9d:MSD Taz/Bex | 3255167 | 2005917 | ** | 0,0074 |
| 6d:MSD Taz vs. 9d:MSD DMSO low | 2434333 | 3909667 | *** | 0,0003 |
| 6d:MSD Taz vs. 9d:MSD DMSO medium | 2434333 | 3771250 | ** | 0,0024 |
| 6d:MSD Taz vs. 9d:MSD DMSO high | 2434333 | 3527833 | * | 0,0444 |
| 6d:MSD Bex vs. 9d:MSD DMSO low | 2474333 | 3909667 | *** | 0,0006 |
| 6d:MSD Bex vs. 9d:MSD DMSO medium | 2474333 | 3771250 | ** | 0,004 |
| 6d:MSD Taz/Bex vs. 9d:MSD DMSO low | 1977167 | 3909667 | **** | <0,0001 |
| 6d:MSD Taz/Bex vs. 9d:MSD DMSO medium | 1977167 | 3771250 | **** | <0,0001 |
| 6d:MSD Taz/Bex vs. 9d:MSD DMSO high | 1977167 | 3527833 | *** | 0,0001 |
| 9d:MSD DMSO low vs. 9d:MSD Taz | 3909667 | 2496667 | *** | 0,0008 |
| 9d:MSD DMSO low vs. 9d:MSD Bex | 3909667 | 2628833 | ** | 0,0049 |
| 9d:MSD DMSO low vs. 9d:MSD Taz/Bex | 3909667 | 2005917 | **** | <0,0001 |
| 9d:MSD DMSO medium vs. 9d:MSD Taz | 3771250 | 2496667 | ** | 0,0054 |
| 9d:MSD DMSO medium vs. 9d:MSD Bex | 3771250 | 2628833 | * | 0,0261 |
| 9d:MSD DMSO medium vs. 9d:MSD Taz/Bex | 3771250 | 2005917 | **** | <0,0001 |
| 9d:MSD DMSO high vs. 9d:MSD Taz/Bex | 3527833 | 2005917 | *** | 0,0002 |

**Appendix table S42**: Mean values and significance levels for control fibroblast cell count comparison (Appendix fig. S5B, significant differences only)

| **Comparison** | **Mean 1** | **Mean 2** | **Summary** | **Adjusted P Value** |
|---|---|---|---|---|
| | | | | |
| 0d:Ctrl. DMSO low vs. 0d:Ctrl. DMSO high | 450000 | 1350000 | *** | 0,0005 |
| 0d:Ctrl. DMSO low vs. 3d:Ctrl. DMSO low | 450000 | 1483800 | **** | <0,0001 |
| 0d:Ctrl. DMSO low vs. 3d:Ctrl. DMSO medium | 450000 | 1854200 | **** | <0,0001 |
| 0d:Ctrl. DMSO low vs. 3d:Ctrl. DMSO high | 450000 | 2272200 | **** | <0,0001 |
| 0d:Ctrl. DMSO low vs. 3d:Ctrl. Taz | 450000 | 1394200 | *** | 0,0002 |
| 0d:Ctrl. DMSO low vs. 3d:Ctrl. Bex | 450000 | 1548200 | **** | <0,0001 |
| 0d:Ctrl. DMSO low vs. 3d:Ctrl. Taz/Bex | 450000 | 1293200 | ** | 0,0018 |

| | | | | |
|---|---|---|---|---|
| 0d:Ctrl. DMSO low vs. 6d:Ctrl. DMSO low | 450000 | 2646000 | **** | <0,0001 |
| 0d:Ctrl. DMSO low vs. 6d:Ctrl. DMSO medium | 450000 | 2476000 | **** | <0,0001 |
| 0d:Ctrl. DMSO low vs. 6d:Ctrl. DMSO high | 450000 | 2682000 | **** | <0,0001 |
| 0d:Ctrl. DMSO low vs. 6d:Ctrl. Taz | 450000 | 1285200 | ** | 0,0021 |
| 0d:Ctrl. DMSO low vs. 6d:Ctrl. Bex | 450000 | 1436800 | **** | <0,0001 |
| 0d:Ctrl. DMSO low vs. 6d:Ctrl. Taz/Bex | 450000 | 1393400 | *** | 0,0002 |
| 0d:Ctrl. DMSO low vs. 9d:Ctrl. DMSO low | 450000 | 3385000 | **** | <0,0001 |
| 0d:Ctrl. DMSO low vs. 9d:Ctrl. DMSO medium | 450000 | 3084600 | **** | <0,0001 |
| 0d:Ctrl. DMSO low vs. 9d:Ctrl. DMSO high | 450000 | 2919600 | **** | <0,0001 |
| 0d:Ctrl. DMSO low vs. 9d:Ctrl. Taz | 450000 | 1288600 | ** | 0,0019 |
| 0d:Ctrl. DMSO low vs. 9d:Ctrl. Bex | 450000 | 1498400 | **** | <0,0001 |
| 0d:Ctrl. DMSO low vs. 9d:Ctrl. Taz/Bex | 450000 | 1340600 | *** | 0,0006 |
| 0d:Ctrl. DMSO medium vs. 3d:Ctrl. DMSO medium | 900000 | 1854200 | *** | 0,0001 |
| 0d:Ctrl. DMSO medium vs. 3d:Ctrl. DMSO high | 900000 | 2272200 | **** | <0,0001 |
| 0d:Ctrl. DMSO medium vs. 6d:Ctrl. DMSO low | 900000 | 2646000 | **** | <0,0001 |
| 0d:Ctrl. DMSO medium vs. 6d:Ctrl. DMSO medium | 900000 | 2476000 | **** | <0,0001 |
| 0d:Ctrl. DMSO medium vs. 6d:Ctrl. DMSO high | 900000 | 2682000 | **** | <0,0001 |
| 0d:Ctrl. DMSO medium vs. 9d:Ctrl. DMSO low | 900000 | 3385000 | **** | <0,0001 |
| 0d:Ctrl. DMSO medium vs. 9d:Ctrl. DMSO medium | 900000 | 3084600 | **** | <0,0001 |
| 0d:Ctrl. DMSO medium vs. 9d:Ctrl. DMSO high | 900000 | 2919600 | **** | <0,0001 |
| 0d:Ctrl. DMSO high vs. 3d:Ctrl. DMSO high | 1350000 | 2272200 | *** | 0,0003 |
| 0d:Ctrl. DMSO high vs. 6d:Ctrl. DMSO low | 1350000 | 2646000 | **** | <0,0001 |
| 0d:Ctrl. DMSO high vs. 6d:Ctrl. DMSO medium | 1350000 | 2476000 | **** | <0,0001 |
| 0d:Ctrl. DMSO high vs. 6d:Ctrl. DMSO high | 1350000 | 2682000 | **** | <0,0001 |
| 0d:Ctrl. DMSO high vs. 9d:Ctrl. DMSO low | 1350000 | 3385000 | **** | <0,0001 |
| 0d:Ctrl. DMSO high vs. 9d:Ctrl. DMSO medium | 1350000 | 3084600 | **** | <0,0001 |
| 0d:Ctrl. DMSO high vs. 9d:Ctrl. DMSO high | 1350000 | 2919600 | **** | <0,0001 |
| 0d:Ctrl. Taz vs. 3d:Ctrl. DMSO medium | 900000 | 1854200 | *** | 0,0001 |
| 0d:Ctrl. Taz vs. 3d:Ctrl. DMSO high | 900000 | 2272200 | **** | <0,0001 |
| 0d:Ctrl. Taz vs. 6d:Ctrl. DMSO low | 900000 | 2646000 | **** | <0,0001 |
| 0d:Ctrl. Taz vs. 6d:Ctrl. DMSO medium | 900000 | 2476000 | **** | <0,0001 |
| 0d:Ctrl. Taz vs. 6d:Ctrl. DMSO high | 900000 | 2682000 | **** | <0,0001 |
| 0d:Ctrl. Taz vs. 9d:Ctrl. DMSO low | 900000 | 3385000 | **** | <0,0001 |
| 0d:Ctrl. Taz vs. 9d:Ctrl. DMSO medium | 900000 | 3084600 | **** | <0,0001 |
| 0d:Ctrl. Taz vs. 9d:Ctrl. DMSO high | 900000 | 2919600 | **** | <0,0001 |
| 0d:Ctrl. Bex vs. 3d:Ctrl. DMSO medium | 900000 | 1854200 | *** | 0,0001 |
| 0d:Ctrl. Bex vs. 3d:Ctrl. DMSO high | 900000 | 2272200 | **** | <0,0001 |
| 0d:Ctrl. Bex vs. 6d:Ctrl. DMSO low | 900000 | 2646000 | **** | <0,0001 |
| 0d:Ctrl. Bex vs. 6d:Ctrl. DMSO medium | 900000 | 2476000 | **** | <0,0001 |
| 0d:Ctrl. Bex vs. 6d:Ctrl. DMSO high | 900000 | 2682000 | **** | <0,0001 |
| 0d:Ctrl. Bex vs. 9d:Ctrl. DMSO low | 900000 | 3385000 | **** | <0,0001 |
| 0d:Ctrl. Bex vs. 9d:Ctrl. DMSO medium | 900000 | 3084600 | **** | <0,0001 |
| 0d:Ctrl. Bex vs. 9d:Ctrl. DMSO high | 900000 | 2919600 | **** | <0,0001 |
| 0d:Ctrl. Taz/Bex vs. 3d:Ctrl. DMSO medium | 900000 | 1854200 | *** | 0,0001 |

| | | | | |
|---|---|---|---|---|
| 0d:Ctrl. Taz/Bex vs. 3d:Ctrl. DMSO high | 900000 | 2272200 | **** | <0,0001 |
| 0d:Ctrl. Taz/Bex vs. 6d:Ctrl. DMSO low | 900000 | 2646000 | **** | <0,0001 |
| 0d:Ctrl. Taz/Bex vs. 6d:Ctrl. DMSO medium | 900000 | 2476000 | **** | <0,0001 |
| 0d:Ctrl. Taz/Bex vs. 6d:Ctrl. DMSO high | 900000 | 2682000 | **** | <0,0001 |
| 0d:Ctrl. Taz/Bex vs. 9d:Ctrl. DMSO low | 900000 | 3385000 | **** | <0,0001 |
| 0d:Ctrl. Taz/Bex vs. 9d:Ctrl. DMSO medium | 900000 | 3084600 | **** | <0,0001 |
| 0d:Ctrl. Taz/Bex vs. 9d:Ctrl. DMSO high | 900000 | 2919600 | **** | <0,0001 |
| 3d:Ctrl. DMSO low vs. 3d:Ctrl. DMSO high | 1483800 | 2272200 | ** | 0,0055 |
| 3d:Ctrl. DMSO low vs. 6d:Ctrl. DMSO low | 1483800 | 2646000 | **** | <0,0001 |
| 3d:Ctrl. DMSO low vs. 6d:Ctrl. DMSO medium | 1483800 | 2476000 | **** | <0,0001 |
| 3d:Ctrl. DMSO low vs. 6d:Ctrl. DMSO high | 1483800 | 2682000 | **** | <0,0001 |
| 3d:Ctrl. DMSO low vs. 9d:Ctrl. DMSO low | 1483800 | 3385000 | **** | <0,0001 |
| 3d:Ctrl. DMSO low vs. 9d:Ctrl. DMSO medium | 1483800 | 3084600 | **** | <0,0001 |
| 3d:Ctrl. DMSO low vs. 9d:Ctrl. DMSO high | 1483800 | 2919600 | **** | <0,0001 |
| 3d:Ctrl. DMSO medium vs. 6d:Ctrl. DMSO low | 1854200 | 2646000 | ** | 0,0051 |
| 3d:Ctrl. DMSO medium vs. 6d:Ctrl. DMSO high | 1854200 | 2682000 | ** | 0,0024 |
| 3d:Ctrl. DMSO medium vs. 9d:Ctrl. DMSO low | 1854200 | 3385000 | **** | <0,0001 |
| 3d:Ctrl. DMSO medium vs. 9d:Ctrl. DMSO medium | 1854200 | 3084600 | **** | <0,0001 |
| 3d:Ctrl. DMSO medium vs. 9d:Ctrl. DMSO high | 1854200 | 2919600 | **** | <0,0001 |
| 3d:Ctrl. DMSO high vs. 3d:Ctrl. Taz | 2272200 | 1394200 | *** | 0,0008 |
| 3d:Ctrl. DMSO high vs. 3d:Ctrl. Bex | 2272200 | 1548200 | * | 0,0189 |
| 3d:Ctrl. DMSO high vs. 3d:Ctrl. Taz/Bex | 2272200 | 1293200 | **** | <0,0001 |
| 3d:Ctrl. DMSO high vs. 6d:Ctrl. Taz | 2272200 | 1285200 | **** | <0,0001 |
| 3d:Ctrl. DMSO high vs. 6d:Ctrl. Bex | 2272200 | 1436800 | ** | 0,0021 |
| 3d:Ctrl. DMSO high vs. 6d:Ctrl. Taz/Bex | 2272200 | 1393400 | *** | 0,0008 |
| 3d:Ctrl. DMSO high vs. 9d:Ctrl. DMSO low | 2272200 | 3385000 | **** | <0,0001 |
| 3d:Ctrl. DMSO high vs. 9d:Ctrl. DMSO medium | 2272200 | 3084600 | ** | 0,0034 |
| 3d:Ctrl. DMSO high vs. 9d:Ctrl. Taz | 2272200 | 1288600 | **** | <0,0001 |
| 3d:Ctrl. DMSO high vs. 9d:Ctrl. Bex | 2272200 | 1498400 | ** | 0,0073 |
| 3d:Ctrl. DMSO high vs. 9d:Ctrl. Taz/Bex | 2272200 | 1340600 | *** | 0,0003 |
| 3d:Ctrl. Taz vs. 6d:Ctrl. DMSO low | 1394200 | 2646000 | **** | <0,0001 |
| 3d:Ctrl. Taz vs. 6d:Ctrl. DMSO medium | 1394200 | 2476000 | **** | <0,0001 |
| 3d:Ctrl. Taz vs. 6d:Ctrl. DMSO high | 1394200 | 2682000 | **** | <0,0001 |
| 3d:Ctrl. Taz vs. 9d:Ctrl. DMSO low | 1394200 | 3385000 | **** | <0,0001 |
| 3d:Ctrl. Taz vs. 9d:Ctrl. DMSO medium | 1394200 | 3084600 | **** | <0,0001 |
| 3d:Ctrl. Taz vs. 9d:Ctrl. DMSO high | 1394200 | 2919600 | **** | <0,0001 |
| 3d:Ctrl. Bex vs. 6d:Ctrl. DMSO low | 1548200 | 2646000 | **** | <0,0001 |
| 3d:Ctrl. Bex vs. 6d:Ctrl. DMSO medium | 1548200 | 2476000 | *** | 0,0003 |
| 3d:Ctrl. Bex vs. 6d:Ctrl. DMSO high | 1548200 | 2682000 | **** | <0,0001 |
| 3d:Ctrl. Bex vs. 9d:Ctrl. DMSO low | 1548200 | 3385000 | **** | <0,0001 |
| 3d:Ctrl. Bex vs. 9d:Ctrl. DMSO medium | 1548200 | 3084600 | **** | <0,0001 |
| 3d:Ctrl. Bex vs. 9d:Ctrl. DMSO high | 1548200 | 2919600 | **** | <0,0001 |
| 3d:Ctrl. Taz/Bex vs. 6d:Ctrl. DMSO low | 1293200 | 2646000 | **** | <0,0001 |
| 3d:Ctrl. Taz/Bex vs. 6d:Ctrl. DMSO medium | 1293200 | 2476000 | **** | <0,0001 |
| 3d:Ctrl. Taz/Bex vs. 6d:Ctrl. DMSO high | 1293200 | 2682000 | **** | <0,0001 |
| 3d:Ctrl. Taz/Bex vs. 9d:Ctrl. DMSO low | 1293200 | 3385000 | **** | <0,0001 |

| | | | | |
|---|---|---|---|---|
| 3d:Ctrl. Taz/Bex vs. 9d:Ctrl. DMSO medium | 1293200 | 3084600 | **** | <0,0001 |
| 3d:Ctrl. Taz/Bex vs. 9d:Ctrl. DMSO high | 1293200 | 2919600 | **** | <0,0001 |
| 6d:Ctrl. DMSO low vs. 6d:Ctrl. Taz | 2646000 | 1285200 | **** | <0,0001 |
| 6d:Ctrl. DMSO low vs. 6d:Ctrl. Bex | 2646000 | 1436800 | **** | <0,0001 |
| 6d:Ctrl. DMSO low vs. 6d:Ctrl. Taz/Bex | 2646000 | 1393400 | **** | <0,0001 |
| 6d:Ctrl. DMSO low vs. 9d:Ctrl. DMSO low | 2646000 | 3385000 | * | 0,0143 |
| 6d:Ctrl. DMSO low vs. 9d:Ctrl. Taz | 2646000 | 1288600 | **** | <0,0001 |
| 6d:Ctrl. DMSO low vs. 9d:Ctrl. Bex | 2646000 | 1498400 | **** | <0,0001 |
| 6d:Ctrl. DMSO low vs. 9d:Ctrl. Taz/Bex | 2646000 | 1340600 | **** | <0,0001 |
| 6d:Ctrl. DMSO medium vs. 6d:Ctrl. Taz | 2476000 | 1285200 | **** | <0,0001 |
| 6d:Ctrl. DMSO medium vs. 6d:Ctrl. Bex | 2476000 | 1436800 | **** | <0,0001 |
| 6d:Ctrl. DMSO medium vs. 6d:Ctrl. Taz/Bex | 2476000 | 1393400 | **** | <0,0001 |
| 6d:Ctrl. DMSO medium vs. 9d:Ctrl. DMSO low | 2476000 | 3385000 | *** | 0,0004 |
| 6d:Ctrl. DMSO medium vs. 9d:Ctrl. Taz | 2476000 | 1288600 | **** | <0,0001 |
| 6d:Ctrl. DMSO medium vs. 9d:Ctrl. Bex | 2476000 | 1498400 | **** | <0,0001 |
| 6d:Ctrl. DMSO medium vs. 9d:Ctrl. Taz/Bex | 2476000 | 1340600 | **** | <0,0001 |
| 6d:Ctrl. DMSO high vs. 6d:Ctrl. Taz | 2682000 | 1285200 | **** | <0,0001 |
| 6d:Ctrl. DMSO high vs. 6d:Ctrl. Bex | 2682000 | 1436800 | **** | <0,0001 |
| 6d:Ctrl. DMSO high vs. 6d:Ctrl. Taz/Bex | 2682000 | 1393400 | **** | <0,0001 |
| 6d:Ctrl. DMSO high vs. 9d:Ctrl. DMSO low | 2682000 | 3385000 | * | 0,0275 |
| 6d:Ctrl. DMSO high vs. 9d:Ctrl. Taz | 2682000 | 1288600 | **** | <0,0001 |
| 6d:Ctrl. DMSO high vs. 9d:Ctrl. Bex | 2682000 | 1498400 | **** | <0,0001 |
| 6d:Ctrl. DMSO high vs. 9d:Ctrl. Taz/Bex | 2682000 | 1340600 | **** | <0,0001 |
| 6d:Ctrl. Taz vs. 9d:Ctrl. DMSO low | 1285200 | 3385000 | **** | <0,0001 |
| 6d:Ctrl. Taz vs. 9d:Ctrl. DMSO medium | 1285200 | 3084600 | **** | <0,0001 |
| 6d:Ctrl. Taz vs. 9d:Ctrl. DMSO high | 1285200 | 2919600 | **** | <0,0001 |
| 6d:Ctrl. Bex vs. 9d:Ctrl. DMSO low | 1436800 | 3385000 | **** | <0,0001 |
| 6d:Ctrl. Bex vs. 9d:Ctrl. DMSO medium | 1436800 | 3084600 | **** | <0,0001 |
| 6d:Ctrl. Bex vs. 9d:Ctrl. DMSO high | 1436800 | 2919600 | **** | <0,0001 |
| 6d:Ctrl. Taz/Bex vs. 9d:Ctrl. DMSO low | 1393400 | 3385000 | **** | <0,0001 |
| 6d:Ctrl. Taz/Bex vs. 9d:Ctrl. DMSO medium | 1393400 | 3084600 | **** | <0,0001 |
| 6d:Ctrl. Taz/Bex vs. 9d:Ctrl. DMSO high | 1393400 | 2919600 | **** | <0,0001 |
| 9d:Ctrl. DMSO low vs. 9d:Ctrl. Taz | 3385000 | 1288600 | **** | <0,0001 |
| 9d:Ctrl. DMSO low vs. 9d:Ctrl. Bex | 3385000 | 1498400 | **** | <0,0001 |
| 9d:Ctrl. DMSO low vs. 9d:Ctrl. Taz/Bex | 3385000 | 1340600 | **** | <0,0001 |
| 9d:Ctrl. DMSO medium vs. 9d:Ctrl. Taz | 3084600 | 1288600 | **** | <0,0001 |
| 9d:Ctrl. DMSO medium vs. 9d:Ctrl. Bex | 3084600 | 1498400 | **** | <0,0001 |
| 9d:Ctrl. DMSO medium vs. 9d:Ctrl. Taz/Bex | 3084600 | 1340600 | **** | <0,0001 |
| 9d:Ctrl. DMSO high vs. 9d:Ctrl. Taz | 2919600 | 1288600 | **** | <0,0001 |
| 9d:Ctrl. DMSO high vs. 9d:Ctrl. Bex | 2919600 | 1498400 | **** | <0,0001 |
| 9d:Ctrl. DMSO high vs. 9d:Ctrl. Taz/Bex | 2919600 | 1340600 | **** | <0,0001 |

**Appendix table S43**: Mean values and significance levels for MSD fibroblast total protein comparison (Appendix fig. S5C, significant differences only)

| Comparison | Mean 1 | Mean 2 | Summary | Adjusted P Value |
|---|---|---|---|---|
| | | | | |
| 0d:MSD DMSO low vs. 3d:MSD DMSO medium | 99,51 | 512,1 | **** | <0,0001 |
| 0d:MSD DMSO low vs. 3d:MSD DMSO high | 99,51 | 583,4 | **** | <0,0001 |
| 0d:MSD DMSO low vs. 3d:MSD Taz | 99,51 | 375,5 | * | 0,012 |
| 0d:MSD DMSO low vs. 3d:MSD Bex | 99,51 | 405,2 | ** | 0,0023 |
| 0d:MSD DMSO low vs. 6d:MSD DMSO low | 99,51 | 683,8 | **** | <0,0001 |
| 0d:MSD DMSO low vs. 6d:MSD DMSO medium | 99,51 | 648 | **** | <0,0001 |
| 0d:MSD DMSO low vs. 6d:MSD DMSO high | 99,51 | 632,2 | **** | <0,0001 |
| 0d:MSD DMSO low vs. 6d:MSD Taz | 99,51 | 502,1 | **** | <0,0001 |
| 0d:MSD DMSO low vs. 6d:MSD Bex | 99,51 | 497,2 | **** | <0,0001 |
| 0d:MSD DMSO low vs. 6d:MSD Taz/Bex | 99,51 | 373,4 | * | 0,0134 |
| 0d:MSD DMSO low vs. 9d:MSD DMSO low | 99,51 | 811,7 | **** | <0,0001 |
| 0d:MSD DMSO low vs. 9d:MSD DMSO medium | 99,51 | 731,3 | **** | <0,0001 |
| 0d:MSD DMSO low vs. 9d:MSD DMSO high | 99,51 | 737,2 | **** | <0,0001 |
| 0d:MSD DMSO low vs. 9d:MSD Taz | 99,51 | 507,8 | **** | <0,0001 |
| 0d:MSD DMSO low vs. 9d:MSD Bex | 99,51 | 582,8 | **** | <0,0001 |
| 0d:MSD DMSO low vs. 9d:MSD Taz/Bex | 99,51 | 520,4 | **** | <0,0001 |
| 0d:MSD DMSO medium vs. 3d:MSD DMSO medium | 181,1 | 512,1 | *** | 0,0005 |
| 0d:MSD DMSO medium vs. 3d:MSD DMSO high | 181,1 | 583,4 | **** | <0,0001 |
| 0d:MSD DMSO medium vs. 6d:MSD DMSO low | 181,1 | 683,8 | **** | <0,0001 |
| 0d:MSD DMSO medium vs. 6d:MSD DMSO medium | 181,1 | 648 | **** | <0,0001 |
| 0d:MSD DMSO medium vs. 6d:MSD DMSO high | 181,1 | 632,2 | **** | <0,0001 |
| 0d:MSD DMSO medium vs. 6d:MSD Taz | 181,1 | 502,1 | ** | 0,0023 |
| 0d:MSD DMSO medium vs. 6d:MSD Bex | 181,1 | 497,2 | ** | 0,0012 |
| 0d:MSD DMSO medium vs. 9d:MSD DMSO low | 181,1 | 811,7 | **** | <0,0001 |
| 0d:MSD DMSO medium vs. 9d:MSD DMSO medium | 181,1 | 731,3 | **** | <0,0001 |
| 0d:MSD DMSO medium vs. 9d:MSD DMSO high | 181,1 | 737,2 | **** | <0,0001 |
| 0d:MSD DMSO medium vs. 9d:MSD Taz | 181,1 | 507,8 | ** | 0,0016 |
| 0d:MSD DMSO medium vs. 9d:MSD Bex | 181,1 | 582,8 | **** | <0,0001 |
| 0d:MSD DMSO medium vs. 9d:MSD Taz/Bex | 181,1 | 520,4 | *** | 0,0003 |
| 0d:MSD DMSO high vs. 3d:MSD DMSO high | 268,4 | 583,4 | ** | 0,0013 |
| 0d:MSD DMSO high vs. 6d:MSD DMSO low | 268,4 | 683,8 | **** | <0,0001 |
| 0d:MSD DMSO high vs. 6d:MSD DMSO medium | 268,4 | 648 | **** | <0,0001 |
| 0d:MSD DMSO high vs. 6d:MSD DMSO high | 268,4 | 632,2 | **** | <0,0001 |
| 0d:MSD DMSO high vs. 9d:MSD DMSO low | 268,4 | 811,7 | **** | <0,0001 |

| | | | | |
|---|---|---|---|---|
| 0d:MSD DMSO high vs. 9d:MSD DMSO medium | 268,4 | 731,3 | **** | <0,0001 |
| 0d:MSD DMSO high vs. 9d:MSD DMSO high | 268,4 | 737,2 | **** | <0,0001 |
| 0d:MSD DMSO high vs. 9d:MSD Bex | 268,4 | 582,8 | ** | 0,0014 |
| 0d:MSD DMSO high vs. 9d:MSD Taz/Bex | 268,4 | 520,4 | * | 0,0396 |
| 0d:MSD Taz vs. 3d:MSD DMSO medium | 178,4 | 512,1 | *** | 0,0004 |
| 0d:MSD Taz vs. 3d:MSD DMSO high | 178,4 | 583,4 | **** | <0,0001 |
| 0d:MSD Taz vs. 6d:MSD DMSO low | 178,4 | 683,8 | **** | <0,0001 |
| 0d:MSD Taz vs. 6d:MSD DMSO medium | 178,4 | 648 | **** | <0,0001 |
| 0d:MSD Taz vs. 6d:MSD DMSO high | 178,4 | 632,2 | **** | <0,0001 |
| 0d:MSD Taz vs. 6d:MSD Taz | 178,4 | 502,1 | ** | 0,0019 |
| 0d:MSD Taz vs. 6d:MSD Bex | 178,4 | 497,2 | ** | 0,0011 |
| 0d:MSD Taz vs. 9d:MSD DMSO low | 178,4 | 811,7 | **** | <0,0001 |
| 0d:MSD Taz vs. 9d:MSD DMSO medium | 178,4 | 731,3 | **** | <0,0001 |
| 0d:MSD Taz vs. 9d:MSD DMSO high | 178,4 | 737,2 | **** | <0,0001 |
| 0d:MSD Taz vs. 9d:MSD Taz | 178,4 | 507,8 | ** | 0,0014 |
| 0d:MSD Taz vs. 9d:MSD Bex | 178,4 | 582,8 | **** | <0,0001 |
| 0d:MSD Taz vs. 9d:MSD Taz/Bex | 178,4 | 520,4 | *** | 0,0002 |
| 0d:MSD Bex vs. 3d:MSD DMSO medium | 187,7 | 512,1 | *** | 0,0008 |
| 0d:MSD Bex vs. 3d:MSD DMSO high | 187,7 | 583,4 | **** | <0,0001 |
| 0d:MSD Bex vs. 6d:MSD DMSO low | 187,7 | 683,8 | **** | <0,0001 |
| 0d:MSD Bex vs. 6d:MSD DMSO medium | 187,7 | 648 | **** | <0,0001 |
| 0d:MSD Bex vs. 6d:MSD DMSO high | 187,7 | 632,2 | **** | <0,0001 |
| 0d:MSD Bex vs. 6d:MSD Taz | 187,7 | 502,1 | ** | 0,0032 |
| 0d:MSD Bex vs. 6d:MSD Bex | 187,7 | 497,2 | ** | 0,0018 |
| 0d:MSD Bex vs. 9d:MSD DMSO low | 187,7 | 811,7 | **** | <0,0001 |
| 0d:MSD Bex vs. 9d:MSD DMSO medium | 187,7 | 731,3 | **** | <0,0001 |
| 0d:MSD Bex vs. 9d:MSD DMSO high | 187,7 | 737,2 | **** | <0,0001 |
| 0d:MSD Bex vs. 9d:MSD Taz | 187,7 | 507,8 | ** | 0,0024 |
| 0d:MSD Bex vs. 9d:MSD Bex | 187,7 | 582,8 | **** | <0,0001 |
| 0d:MSD Bex vs. 9d:MSD Taz/Bex | 187,7 | 520,4 | *** | 0,0005 |
| 0d:MSD Taz/Bex vs. 3d:MSD DMSO medium | 185,9 | 512,1 | *** | 0,0007 |
| 0d:MSD Taz/Bex vs. 3d:MSD DMSO high | 185,9 | 583,4 | **** | <0,0001 |
| 0d:MSD Taz/Bex vs. 6d:MSD DMSO low | 185,9 | 683,8 | **** | <0,0001 |
| 0d:MSD Taz/Bex vs. 6d:MSD DMSO medium | 185,9 | 648 | **** | <0,0001 |
| 0d:MSD Taz/Bex vs. 6d:MSD DMSO high | 185,9 | 632,2 | **** | <0,0001 |
| 0d:MSD Taz/Bex vs. 6d:MSD Taz | 185,9 | 502,1 | ** | 0,0029 |
| 0d:MSD Taz/Bex vs. 6d:MSD Bex | 185,9 | 497,2 | ** | 0,0017 |
| 0d:MSD Taz/Bex vs. 9d:MSD DMSO low | 185,9 | 811,7 | **** | <0,0001 |
| 0d:MSD Taz/Bex vs. 9d:MSD DMSO medium | 185,9 | 731,3 | **** | <0,0001 |
| 0d:MSD Taz/Bex vs. 9d:MSD DMSO high | 185,9 | 737,2 | **** | <0,0001 |
| 0d:MSD Taz/Bex vs. 9d:MSD Taz | 185,9 | 507,8 | ** | 0,0022 |
| 0d:MSD Taz/Bex vs. 9d:MSD Bex | 185,9 | 582,8 | **** | <0,0001 |
| 0d:MSD Taz/Bex vs. 9d:MSD Taz/Bex | 185,9 | 520,4 | *** | 0,0004 |
| 3d:MSD DMSO low vs. 3d:MSD DMSO high | 300,4 | 583,4 | ** | 0,0082 |
| 3d:MSD DMSO low vs. 6d:MSD DMSO low | 300,4 | 683,8 | **** | <0,0001 |
| 3d:MSD DMSO low vs. 6d:MSD DMSO | 300,4 | 648 | *** | 0,0002 |

| | | | | |
|---|---|---|---|---|
| medium | | | | |
| 3d:MSD DMSO low vs. 6d:MSD DMSO high | 300,4 | 632,2 | *** | 0,0005 |
| 3d:MSD DMSO low vs. 9d:MSD DMSO low | 300,4 | 811,7 | **** | <0,0001 |
| 3d:MSD DMSO low vs. 9d:MSD DMSO medium | 300,4 | 731,3 | **** | <0,0001 |
| 3d:MSD DMSO low vs. 9d:MSD DMSO high | 300,4 | 737,2 | **** | <0,0001 |
| 3d:MSD DMSO low vs. 9d:MSD Bex | 300,4 | 582,8 | ** | 0,0085 |
| 3d:MSD DMSO medium vs. 9d:MSD DMSO low | 512,1 | 811,7 | ** | 0,0033 |
| 3d:MSD DMSO high vs. 3d:MSD Taz/Bex | 583,4 | 326,5 | * | 0,0313 |
| 3d:MSD Taz vs. 6d:MSD DMSO low | 375,5 | 683,8 | ** | 0,002 |
| 3d:MSD Taz vs. 6d:MSD DMSO medium | 375,5 | 648 | * | 0,0144 |
| 3d:MSD Taz vs. 6d:MSD DMSO high | 375,5 | 632,2 | * | 0,0317 |
| 3d:MSD Taz vs. 9d:MSD DMSO low | 375,5 | 811,7 | **** | <0,0001 |
| 3d:MSD Taz vs. 9d:MSD DMSO medium | 375,5 | 731,3 | *** | 0,0001 |
| 3d:MSD Taz vs. 9d:MSD DMSO high | 375,5 | 737,2 | **** | <0,0001 |
| 3d:MSD Bex vs. 6d:MSD DMSO low | 405,2 | 683,8 | * | 0,0104 |
| 3d:MSD Bex vs. 9d:MSD DMSO low | 405,2 | 811,7 | **** | <0,0001 |
| 3d:MSD Bex vs. 9d:MSD DMSO medium | 405,2 | 731,3 | *** | 0,0007 |
| 3d:MSD Bex vs. 9d:MSD DMSO high | 405,2 | 737,2 | *** | 0,0005 |
| 3d:MSD Taz/Bex vs. 6d:MSD DMSO low | 326,5 | 683,8 | **** | <0,0001 |
| 3d:MSD Taz/Bex vs. 6d:MSD DMSO medium | 326,5 | 648 | *** | 0,0009 |
| 3d:MSD Taz/Bex vs. 6d:MSD DMSO high | 326,5 | 632,2 | ** | 0,0023 |
| 3d:MSD Taz/Bex vs. 9d:MSD DMSO low | 326,5 | 811,7 | **** | <0,0001 |
| 3d:MSD Taz/Bex vs. 9d:MSD DMSO medium | 326,5 | 731,3 | **** | <0,0001 |
| 3d:MSD Taz/Bex vs. 9d:MSD DMSO high | 326,5 | 737,2 | **** | <0,0001 |
| 3d:MSD Taz/Bex vs. 9d:MSD Bex | 326,5 | 582,8 | * | 0,0322 |
| 6d:MSD DMSO low vs. 6d:MSD Taz/Bex | 683,8 | 373,4 | ** | 0,0017 |
| 6d:MSD DMSO medium vs. 6d:MSD Taz/Bex | 648 | 373,4 | * | 0,0129 |
| 6d:MSD DMSO high vs. 6d:MSD Taz/Bex | 632,2 | 373,4 | * | 0,0286 |
| 6d:MSD Taz vs. 9d:MSD DMSO low | 502,1 | 811,7 | ** | 0,0042 |
| 6d:MSD Bex vs. 9d:MSD DMSO low | 497,2 | 811,7 | ** | 0,0014 |
| 6d:MSD Taz/Bex vs. 9d:MSD DMSO low | 373,4 | 811,7 | **** | <0,0001 |
| 6d:MSD Taz/Bex vs. 9d:MSD DMSO medium | 373,4 | 731,3 | **** | <0,0001 |
| 6d:MSD Taz/Bex vs. 9d:MSD DMSO high | 373,4 | 737,2 | **** | <0,0001 |
| 9d:MSD DMSO low vs. 9d:MSD Taz | 811,7 | 507,8 | ** | 0,0057 |
| 9d:MSD DMSO low vs. 9d:MSD Taz/Bex | 811,7 | 520,4 | ** | 0,0052 |

**Appendix table S44**: Mean values and significance levels for control fibroblast total protein comparison (Appendix fig. S5D, significant differences only)

| **Comparison** | **Mean 1** | **Mean 2** | **Summary** | **Adjusted P Value** |
|---|---|---|---|---|
| | | | | |
| 0d:Ctrl. DMSO low vs. 0d:Ctrl. DMSO high | 81,28 | 279,2 | *** | 0,0009 |
| 0d:Ctrl. DMSO low vs. 3d:Ctrl. DMSO medium | 81,28 | 291,5 | *** | 0,0002 |

| | | | | |
|---|---|---|---|---|
| 0d:Ctrl. DMSO low vs. 3d:Ctrl. DMSO high | 81,28 | 340,4 | **** | <0,0001 |
| 0d:Ctrl. DMSO low vs. 6d:Ctrl. DMSO low | 81,28 | 376,2 | **** | <0,0001 |
| 0d:Ctrl. DMSO low vs. 6d:Ctrl. DMSO medium | 81,28 | 397,2 | **** | <0,0001 |
| 0d:Ctrl. DMSO low vs. 6d:Ctrl. DMSO high | 81,28 | 422,7 | **** | <0,0001 |
| 0d:Ctrl. DMSO low vs. 6d:Ctrl. Bex | 81,28 | 316,4 | **** | <0,0001 |
| 0d:Ctrl. DMSO low vs. 9d:Ctrl. DMSO low | 81,28 | 488,7 | **** | <0,0001 |
| 0d:Ctrl. DMSO low vs. 9d:Ctrl. DMSO medium | 81,28 | 501,3 | **** | <0,0001 |
| 0d:Ctrl. DMSO low vs. 9d:Ctrl. DMSO high | 81,28 | 563,2 | **** | <0,0001 |
| 0d:Ctrl. DMSO low vs. 9d:Ctrl. Taz | 81,28 | 251,4 | * | 0,0215 |
| 0d:Ctrl. DMSO low vs. 9d:Ctrl. Bex | 81,28 | 373,4 | **** | <0,0001 |
| 0d:Ctrl. DMSO low vs. 9d:Ctrl. Taz/Bex | 81,28 | 251,7 | * | 0,0209 |
| 0d:Ctrl. DMSO medium vs. 3d:Ctrl. DMSO high | 150,9 | 340,4 | ** | 0,0019 |
| 0d:Ctrl. DMSO medium vs. 6d:Ctrl. DMSO low | 150,9 | 376,2 | **** | <0,0001 |
| 0d:Ctrl. DMSO medium vs. 6d:Ctrl. DMSO medium | 150,9 | 397,2 | **** | <0,0001 |
| 0d:Ctrl. DMSO medium vs. 6d:Ctrl. DMSO high | 150,9 | 422,7 | **** | <0,0001 |
| 0d:Ctrl. DMSO medium vs. 6d:Ctrl. Bex | 150,9 | 316,4 | * | 0,0303 |
| 0d:Ctrl. DMSO medium vs. 9d:Ctrl. DMSO low | 150,9 | 488,7 | **** | <0,0001 |
| 0d:Ctrl. DMSO medium vs. 9d:Ctrl. DMSO medium | 150,9 | 501,3 | **** | <0,0001 |
| 0d:Ctrl. DMSO medium vs. 9d:Ctrl. DMSO high | 150,9 | 563,2 | **** | <0,0001 |
| 0d:Ctrl. DMSO medium vs. 9d:Ctrl. Bex | 150,9 | 373,4 | *** | 0,0002 |
| 0d:Ctrl. DMSO high vs. 9d:Ctrl. DMSO low | 279,2 | 488,7 | *** | 0,0003 |
| 0d:Ctrl. DMSO high vs. 9d:Ctrl. DMSO medium | 279,2 | 501,3 | **** | <0,0001 |
| 0d:Ctrl. DMSO high vs. 9d:Ctrl. DMSO high | 279,2 | 563,2 | **** | <0,0001 |
| 0d:Ctrl. Taz vs. 3d:Ctrl. DMSO high | 149,4 | 340,4 | ** | 0,0038 |
| 0d:Ctrl. Taz vs. 6d:Ctrl. DMSO low | 149,4 | 376,2 | *** | 0,0001 |
| 0d:Ctrl. Taz vs. 6d:Ctrl. DMSO medium | 149,4 | 397,2 | **** | <0,0001 |
| 0d:Ctrl. Taz vs. 6d:Ctrl. DMSO high | 149,4 | 422,7 | **** | <0,0001 |
| 0d:Ctrl. Taz vs. 6d:Ctrl. Bex | 149,4 | 316,4 | * | 0,0457 |
| 0d:Ctrl. Taz vs. 9d:Ctrl. DMSO low | 149,4 | 488,7 | **** | <0,0001 |
| 0d:Ctrl. Taz vs. 9d:Ctrl. DMSO medium | 149,4 | 501,3 | **** | <0,0001 |
| 0d:Ctrl. Taz vs. 9d:Ctrl. DMSO high | 149,4 | 563,2 | **** | <0,0001 |
| 0d:Ctrl. Taz vs. 9d:Ctrl. Bex | 149,4 | 373,4 | *** | 0,0004 |
| 0d:Ctrl. Bex vs. 3d:Ctrl. DMSO high | 159,6 | 340,4 | ** | 0,0091 |
| 0d:Ctrl. Bex vs. 6d:Ctrl. DMSO low | 159,6 | 376,2 | *** | 0,0004 |
| 0d:Ctrl. Bex vs. 6d:Ctrl. DMSO medium | 159,6 | 397,2 | **** | <0,0001 |
| 0d:Ctrl. Bex vs. 6d:Ctrl. DMSO high | 159,6 | 422,7 | **** | <0,0001 |
| 0d:Ctrl. Bex vs. 9d:Ctrl. DMSO low | 159,6 | 488,7 | **** | <0,0001 |
| 0d:Ctrl. Bex vs. 9d:Ctrl. DMSO medium | 159,6 | 501,3 | **** | <0,0001 |
| 0d:Ctrl. Bex vs. 9d:Ctrl. DMSO high | 159,6 | 563,2 | **** | <0,0001 |
| 0d:Ctrl. Bex vs. 9d:Ctrl. Bex | 159,6 | 373,4 | ** | 0,0011 |
| 0d:Ctrl. Taz/Bex vs. 3d:Ctrl. DMSO high | 154,4 | 340,4 | ** | 0,0059 |
| 0d:Ctrl. Taz/Bex vs. 6d:Ctrl. DMSO low | 154,4 | 376,2 | *** | 0,0002 |
| 0d:Ctrl. Taz/Bex vs. 6d:Ctrl. DMSO medium | 154,4 | 397,2 | **** | <0,0001 |
| 0d:Ctrl. Taz/Bex vs. 6d:Ctrl. DMSO high | 154,4 | 422,7 | **** | <0,0001 |
| 0d:Ctrl. Taz/Bex vs. 9d:Ctrl. DMSO low | 154,4 | 488,7 | **** | <0,0001 |

| | | | | |
|---|---|---|---|---|
| 0d:Ctrl. Taz/Bex vs. 9d:Ctrl. DMSO medium | 154,4 | 501,3 | **** | <0,0001 |
| 0d:Ctrl. Taz/Bex vs. 9d:Ctrl. DMSO high | 154,4 | 563,2 | **** | <0,0001 |
| 0d:Ctrl. Taz/Bex vs. 9d:Ctrl. Bex | 154,4 | 373,4 | *** | 0,0007 |
| 3d:Ctrl. DMSO low vs. 6d:Ctrl. DMSO low | 200,4 | 376,2 | ** | 0,0068 |
| 3d:Ctrl. DMSO low vs. 6d:Ctrl. DMSO medium | 200,4 | 397,2 | *** | 0,001 |
| 3d:Ctrl. DMSO low vs. 6d:Ctrl. DMSO high | 200,4 | 422,7 | **** | <0,0001 |
| 3d:Ctrl. DMSO low vs. 9d:Ctrl. DMSO low | 200,4 | 488,7 | **** | <0,0001 |
| 3d:Ctrl. DMSO low vs. 9d:Ctrl. DMSO medium | 200,4 | 501,3 | **** | <0,0001 |
| 3d:Ctrl. DMSO low vs. 9d:Ctrl. DMSO high | 200,4 | 563,2 | **** | <0,0001 |
| 3d:Ctrl. DMSO low vs. 9d:Ctrl. Bex | 200,4 | 373,4 | * | 0,0172 |
| 3d:Ctrl. DMSO medium vs. 9d:Ctrl. DMSO low | 291,5 | 488,7 | *** | 0,0009 |
| 3d:Ctrl. DMSO medium vs. 9d:Ctrl. DMSO medium | 291,5 | 501,3 | *** | 0,0003 |
| 3d:Ctrl. DMSO medium vs. 9d:Ctrl. DMSO high | 291,5 | 563,2 | **** | <0,0001 |
| 3d:Ctrl. DMSO high vs. 9d:Ctrl. DMSO medium | 340,4 | 501,3 | * | 0,024 |
| 3d:Ctrl. DMSO high vs. 9d:Ctrl. DMSO high | 340,4 | 563,2 | **** | <0,0001 |
| 3d:Ctrl. Taz vs. 6d:Ctrl. DMSO low | 205,3 | 376,2 | * | 0,0201 |
| 3d:Ctrl. Taz vs. 6d:Ctrl. DMSO medium | 205,3 | 397,2 | ** | 0,0036 |
| 3d:Ctrl. Taz vs. 6d:Ctrl. DMSO high | 205,3 | 422,7 | *** | 0,0003 |
| 3d:Ctrl. Taz vs. 9d:Ctrl. DMSO low | 205,3 | 488,7 | **** | <0,0001 |
| 3d:Ctrl. Taz vs. 9d:Ctrl. DMSO medium | 205,3 | 501,3 | **** | <0,0001 |
| 3d:Ctrl. Taz vs. 9d:Ctrl. DMSO high | 205,3 | 563,2 | **** | <0,0001 |
| 3d:Ctrl. Taz vs. 9d:Ctrl. Bex | 205,3 | 373,4 | * | 0,0424 |
| 3d:Ctrl. Bex vs. 6d:Ctrl. DMSO medium | 238,1 | 397,2 | * | 0,0488 |
| 3d:Ctrl. Bex vs. 6d:Ctrl. DMSO high | 238,1 | 422,7 | ** | 0,0067 |
| 3d:Ctrl. Bex vs. 9d:Ctrl. DMSO low | 238,1 | 488,7 | **** | <0,0001 |
| 3d:Ctrl. Bex vs. 9d:Ctrl. DMSO medium | 238,1 | 501,3 | **** | <0,0001 |
| 3d:Ctrl. Bex vs. 9d:Ctrl. DMSO high | 238,1 | 563,2 | **** | <0,0001 |
| 3d:Ctrl. Taz/Bex vs. 6d:Ctrl. DMSO low | 190,1 | 376,2 | ** | 0,0058 |
| 3d:Ctrl. Taz/Bex vs. 6d:Ctrl. DMSO medium | 190,1 | 397,2 | *** | 0,0009 |
| 3d:Ctrl. Taz/Bex vs. 6d:Ctrl. DMSO high | 190,1 | 422,7 | **** | <0,0001 |
| 3d:Ctrl. Taz/Bex vs. 9d:Ctrl. DMSO low | 190,1 | 488,7 | **** | <0,0001 |
| 3d:Ctrl. Taz/Bex vs. 9d:Ctrl. DMSO medium | 190,1 | 501,3 | **** | <0,0001 |
| 3d:Ctrl. Taz/Bex vs. 9d:Ctrl. DMSO high | 190,1 | 563,2 | **** | <0,0001 |
| 3d:Ctrl. Taz/Bex vs. 9d:Ctrl. Bex | 190,1 | 373,4 | * | 0,0141 |
| 6d:Ctrl. DMSO low vs. 9d:Ctrl. DMSO high | 376,2 | 563,2 | ** | 0,0025 |
| 6d:Ctrl. DMSO medium vs. 6d:Ctrl. Taz | 397,2 | 236,8 | * | 0,0444 |
| 6d:Ctrl. DMSO medium vs. 6d:Ctrl. Taz/Bex | 397,2 | 228,3 | * | 0,0236 |
| 6d:Ctrl. DMSO medium vs. 9d:Ctrl. DMSO high | 397,2 | 563,2 | * | 0,0157 |
| 6d:Ctrl. DMSO high vs. 6d:Ctrl. Taz | 422,7 | 236,8 | ** | 0,006 |
| 6d:Ctrl. DMSO high vs. 6d:Ctrl. Taz/Bex | 422,7 | 228,3 | ** | 0,0029 |
| 6d:Ctrl. DMSO high vs. 9d:Ctrl. Taz | 422,7 | 251,4 | * | 0,0196 |
| 6d:Ctrl. DMSO high vs. 9d:Ctrl. Taz/Bex | 422,7 | 251,7 | * | 0,0202 |
| 6d:Ctrl. Taz vs. 9d:Ctrl. DMSO low | 236,8 | 488,7 | **** | <0,0001 |
| 6d:Ctrl. Taz vs. 9d:Ctrl. DMSO medium | 236,8 | 501,3 | **** | <0,0001 |
| 6d:Ctrl. Taz vs. 9d:Ctrl. DMSO high | 236,8 | 563,2 | **** | <0,0001 |
| 6d:Ctrl. Bex vs. 9d:Ctrl. DMSO low | 316,4 | 488,7 | * | 0,0181 |

| | | | | |
|---|---|---|---|---|
| 6d:Ctrl. Bex vs. 9d:Ctrl. DMSO medium | 316,4 | 501,3 | ** | 0,0065 |
| 6d:Ctrl. Bex vs. 9d:Ctrl. DMSO high | 316,4 | 563,2 | **** | <0,0001 |
| 6d:Ctrl. Taz/Bex vs. 9d:Ctrl. DMSO low | 228,3 | 488,7 | **** | <0,0001 |
| 6d:Ctrl. Taz/Bex vs. 9d:Ctrl. DMSO medium | 228,3 | 501,3 | **** | <0,0001 |
| 6d:Ctrl. Taz/Bex vs. 9d:Ctrl. DMSO high | 228,3 | 563,2 | **** | <0,0001 |
| 9d:Ctrl. DMSO low vs. 9d:Ctrl. Taz | 488,7 | 251,4 | **** | <0,0001 |
| 9d:Ctrl. DMSO low vs. 9d:Ctrl. Taz/Bex | 488,7 | 251,7 | **** | <0,0001 |
| 9d:Ctrl. DMSO medium vs. 9d:Ctrl. Taz | 501,3 | 251,4 | **** | <0,0001 |
| 9d:Ctrl. DMSO medium vs. 9d:Ctrl. Taz/Bex | 501,3 | 251,7 | **** | <0,0001 |
| 9d:Ctrl. DMSO high vs. 9d:Ctrl. Taz | 563,2 | 251,4 | **** | <0,0001 |
| 9d:Ctrl. DMSO high vs. 9d:Ctrl. Bex | 563,2 | 373,4 | ** | 0,0043 |
| 9d:Ctrl. DMSO high vs. 9d:Ctrl. Taz/Bex | 563,2 | 251,7 | **** | <0,0001 |

**Appendix table S45**: Mean values and significance levels for MSD fibroblast ARSA activity comparison (Appendix fig. S5E, significant differences only)

| **Comparison** | **Mean 1** | **Mean 2** | **Summary** | **Adjusted P Value** |
|---|---|---|---|---|
| | | | | |
| 0d:MSD DMSO low vs. 3d:MSD Taz/Bex | 10,36 | 30,16 | * | 0,0159 |
| 0d:MSD DMSO low vs. 6d:MSD Taz | 10,36 | 34,32 | *** | 0,0006 |
| 0d:MSD DMSO low vs. 6d:MSD Taz/Bex | 10,36 | 105,6 | **** | <0,0001 |
| 0d:MSD DMSO low vs. 9d:MSD Taz | 10,36 | 73,39 | **** | <0,0001 |
| 0d:MSD DMSO low vs. 9d:MSD Taz/Bex | 10,36 | 130,8 | **** | <0,0001 |
| 0d:MSD DMSO medium vs. 6d:MSD Taz | 12,1 | 34,32 | ** | 0,0026 |
| 0d:MSD DMSO medium vs. 6d:MSD Taz/Bex | 12,1 | 105,6 | **** | <0,0001 |
| 0d:MSD DMSO medium vs. 9d:MSD Taz | 12,1 | 73,39 | **** | <0,0001 |
| 0d:MSD DMSO medium vs. 9d:MSD Taz/Bex | 12,1 | 130,8 | **** | <0,0001 |
| 0d:MSD DMSO high vs. 6d:MSD Taz | 12,28 | 34,32 | ** | 0,003 |
| 0d:MSD DMSO high vs. 6d:MSD Taz/Bex | 12,28 | 105,6 | **** | <0,0001 |
| 0d:MSD DMSO high vs. 9d:MSD Taz | 12,28 | 73,39 | **** | <0,0001 |
| 0d:MSD DMSO high vs. 9d:MSD Taz/Bex | 12,28 | 130,8 | **** | <0,0001 |
| 0d:MSD Taz vs. 3d:MSD Taz/Bex | 11,59 | 30,16 | * | 0,0363 |
| 0d:MSD Taz vs. 6d:MSD Taz | 11,59 | 34,32 | ** | 0,0017 |
| 0d:MSD Taz vs. 6d:MSD Taz/Bex | 11,59 | 105,6 | **** | <0,0001 |
| 0d:MSD Taz vs. 9d:MSD Taz | 11,59 | 73,39 | **** | <0,0001 |
| 0d:MSD Taz vs. 9d:MSD Taz/Bex | 11,59 | 130,8 | **** | <0,0001 |
| 0d:MSD Bex vs. 3d:MSD Taz/Bex | 11,23 | 30,16 | * | 0,0287 |
| 0d:MSD Bex vs. 6d:MSD Taz | 11,23 | 34,32 | ** | 0,0013 |
| 0d:MSD Bex vs. 6d:MSD Taz/Bex | 11,23 | 105,6 | **** | <0,0001 |
| 0d:MSD Bex vs. 9d:MSD Taz | 11,23 | 73,39 | **** | <0,0001 |
| 0d:MSD Bex vs. 9d:MSD Taz/Bex | 11,23 | 130,8 | **** | <0,0001 |
| 0d:MSD Taz/Bex vs. 3d:MSD Taz/Bex | 9,065 | 30,16 | ** | 0,0062 |
| 0d:MSD Taz/Bex vs. 6d:MSD Taz | 9,065 | 34,32 | *** | 0,0002 |
| 0d:MSD Taz/Bex vs. 6d:MSD Taz/Bex | 9,065 | 105,6 | **** | <0,0001 |
| 0d:MSD Taz/Bex vs. 9d:MSD Taz | 9,065 | 73,39 | **** | <0,0001 |

| | | | | |
|---|---|---|---|---|
| 0d:MSD Taz/Bex vs. 9d:MSD Taz/Bex | 9,065 | 130,8 | **** | <0,0001 |
| 3d:MSD DMSO low vs. 3d:MSD Taz/Bex | 5,343 | 30,16 | *** | 0,0003 |
| 3d:MSD DMSO low vs. 6d:MSD Taz | 5,343 | 34,32 | **** | <0,0001 |
| 3d:MSD DMSO low vs. 6d:MSD Taz/Bex | 5,343 | 105,6 | **** | <0,0001 |
| 3d:MSD DMSO low vs. 9d:MSD Taz | 5,343 | 73,39 | **** | <0,0001 |
| 3d:MSD DMSO low vs. 9d:MSD Bex | 5,343 | 24,37 | * | 0,027 |
| 3d:MSD DMSO low vs. 9d:MSD Taz/Bex | 5,343 | 130,8 | **** | <0,0001 |
| 3d:MSD DMSO medium vs. 3d:MSD Taz/Bex | 7,02 | 30,16 | ** | 0,0012 |
| 3d:MSD DMSO medium vs. 6d:MSD Taz | 7,02 | 34,32 | **** | <0,0001 |
| 3d:MSD DMSO medium vs. 6d:MSD Taz/Bex | 7,02 | 105,6 | **** | <0,0001 |
| 3d:MSD DMSO medium vs. 9d:MSD Taz | 7,02 | 73,39 | **** | <0,0001 |
| 3d:MSD DMSO medium vs. 9d:MSD Taz/Bex | 7,02 | 130,8 | **** | <0,0001 |
| 3d:MSD DMSO high vs. 3d:MSD Taz/Bex | 9,503 | 30,16 | ** | 0,0086 |
| 3d:MSD DMSO high vs. 6d:MSD Taz | 9,503 | 34,32 | *** | 0,0003 |
| 3d:MSD DMSO high vs. 6d:MSD Taz/Bex | 9,503 | 105,6 | **** | <0,0001 |
| 3d:MSD DMSO high vs. 9d:MSD Taz | 9,503 | 73,39 | **** | <0,0001 |
| 3d:MSD DMSO high vs. 9d:MSD Taz/Bex | 9,503 | 130,8 | **** | <0,0001 |
| 3d:MSD Taz vs. 3d:MSD Taz/Bex | 9,762 | 30,16 | * | 0,0103 |
| 3d:MSD Taz vs. 6d:MSD Taz | 9,762 | 34,32 | *** | 0,0004 |
| 3d:MSD Taz vs. 6d:MSD Taz/Bex | 9,762 | 105,6 | **** | <0,0001 |
| 3d:MSD Taz vs. 9d:MSD Taz | 9,762 | 73,39 | **** | <0,0001 |
| 3d:MSD Taz vs. 9d:MSD Taz/Bex | 9,762 | 130,8 | **** | <0,0001 |
| 3d:MSD Bex vs. 3d:MSD Taz/Bex | 10,12 | 30,16 | * | 0,0134 |
| 3d:MSD Bex vs. 6d:MSD Taz | 10,12 | 34,32 | *** | 0,0005 |
| 3d:MSD Bex vs. 6d:MSD Taz/Bex | 10,12 | 105,6 | **** | <0,0001 |
| 3d:MSD Bex vs. 9d:MSD Taz | 10,12 | 73,39 | **** | <0,0001 |
| 3d:MSD Bex vs. 9d:MSD Taz/Bex | 10,12 | 130,8 | **** | <0,0001 |
| 3d:MSD Taz/Bex vs. 6d:MSD DMSO low | 30,16 | 7,14 | ** | 0,0014 |
| 3d:MSD Taz/Bex vs. 6d:MSD DMSO medium | 30,16 | 11,21 | * | 0,0284 |
| 3d:MSD Taz/Bex vs. 6d:MSD Taz/Bex | 30,16 | 105,6 | **** | <0,0001 |
| 3d:MSD Taz/Bex vs. 9d:MSD Taz | 30,16 | 73,39 | **** | <0,0001 |
| 3d:MSD Taz/Bex vs. 9d:MSD Taz/Bex | 30,16 | 130,8 | **** | <0,0001 |
| 6d:MSD DMSO low vs. 6d:MSD Taz | 7,14 | 34,32 | **** | <0,0001 |
| 6d:MSD DMSO low vs. 6d:MSD Taz/Bex | 7,14 | 105,6 | **** | <0,0001 |
| 6d:MSD DMSO low vs. 9d:MSD Taz | 7,14 | 73,39 | **** | <0,0001 |
| 6d:MSD DMSO low vs. 9d:MSD Taz/Bex | 7,14 | 130,8 | **** | <0,0001 |
| 6d:MSD DMSO medium vs. 6d:MSD Taz | 11,21 | 34,32 | ** | 0,0013 |
| 6d:MSD DMSO medium vs. 6d:MSD Taz/Bex | 11,21 | 105,6 | **** | <0,0001 |
| 6d:MSD DMSO medium vs. 9d:MSD Taz | 11,21 | 73,39 | **** | <0,0001 |
| 6d:MSD DMSO medium vs. 9d:MSD Taz/Bex | 11,21 | 130,8 | **** | <0,0001 |
| 6d:MSD DMSO high vs. 6d:MSD Taz | 13,55 | 34,32 | ** | 0,0079 |
| 6d:MSD DMSO high vs. 6d:MSD Taz/Bex | 13,55 | 105,6 | **** | <0,0001 |
| 6d:MSD DMSO high vs. 9d:MSD Taz | 13,55 | 73,39 | **** | <0,0001 |
| 6d:MSD DMSO high vs. 9d:MSD Taz/Bex | 13,55 | 130,8 | **** | <0,0001 |
| 6d:MSD Taz vs. 6d:MSD Taz/Bex | 34,32 | 105,6 | **** | <0,0001 |
| 6d:MSD Taz vs. 9d:MSD DMSO low | 34,32 | 13,27 | ** | 0,0064 |
| 6d:MSD Taz vs. 9d:MSD DMSO medium | 34,32 | 16,23 | * | 0,0493 |

| | | | | |
|---|---|---|---|---|
| 6d:MSD Taz vs. 9d:MSD Taz | 34,32 | 73,39 | **** | <0,0001 |
| 6d:MSD Taz vs. 9d:MSD Taz/Bex | 34,32 | 130,8 | **** | <0,0001 |
| 6d:MSD Bex vs. 6d:MSD Taz/Bex | 16,87 | 105,6 | **** | <0,0001 |
| 6d:MSD Bex vs. 9d:MSD Taz | 16,87 | 73,39 | **** | <0,0001 |
| 6d:MSD Bex vs. 9d:MSD Taz/Bex | 16,87 | 130,8 | **** | <0,0001 |
| 6d:MSD Taz/Bex vs. 9d:MSD DMSO low | 105,6 | 13,27 | **** | <0,0001 |
| 6d:MSD Taz/Bex vs. 9d:MSD DMSO medium | 105,6 | 16,23 | **** | <0,0001 |
| 6d:MSD Taz/Bex vs. 9d:MSD DMSO high | 105,6 | 16,67 | **** | <0,0001 |
| 6d:MSD Taz/Bex vs. 9d:MSD Taz | 105,6 | 73,39 | **** | <0,0001 |
| 6d:MSD Taz/Bex vs. 9d:MSD Bex | 105,6 | 24,37 | **** | <0,0001 |
| 6d:MSD Taz/Bex vs. 9d:MSD Taz/Bex | 105,6 | 130,8 | *** | 0,0002 |
| 9d:MSD DMSO low vs. 9d:MSD Taz | 13,27 | 73,39 | **** | <0,0001 |
| 9d:MSD DMSO low vs. 9d:MSD Taz/Bex | 13,27 | 130,8 | **** | <0,0001 |
| 9d:MSD DMSO medium vs. 9d:MSD Taz | 16,23 | 73,39 | **** | <0,0001 |
| 9d:MSD DMSO medium vs. 9d:MSD Taz/Bex | 16,23 | 130,8 | **** | <0,0001 |
| 9d:MSD DMSO high vs. 9d:MSD Taz | 16,67 | 73,39 | **** | <0,0001 |
| 9d:MSD DMSO high vs. 9d:MSD Taz/Bex | 16,67 | 130,8 | **** | <0,0001 |
| 9d:MSD Taz vs. 9d:MSD Bex | 73,39 | 24,37 | **** | <0,0001 |
| 9d:MSD Taz vs. 9d:MSD Taz/Bex | 73,39 | 130,8 | **** | <0,0001 |
| 9d:MSD Bex vs. 9d:MSD Taz/Bex | 24,37 | 130,8 | **** | <0,0001 |

**Appendix table S46**: Mean values and significance levels for control fibroblast ARSA activity comparison (Appendix fig. S5F, significant differences only)

| **Comparison** | **Mean 1** | **Mean 2** | **Summary** | **Adjusted P Value** |
|---|---|---|---|---|
| 0d:Ctrl. DMSO low vs. 6d:Ctrl. DMSO high | 654,9 | 1102 | **** | <0,0001 |
| 0d:Ctrl. DMSO low vs. 6d:Ctrl. Taz | 654,9 | 1171 | **** | <0,0001 |
| 0d:Ctrl. DMSO low vs. 6d:Ctrl. Taz/Bex | 654,9 | 1124 | **** | <0,0001 |
| 0d:Ctrl. DMSO low vs. 9d:Ctrl. DMSO medium | 654,9 | 1040 | ** | 0,0019 |
| 0d:Ctrl. DMSO low vs. 9d:Ctrl. DMSO high | 654,9 | 1147 | **** | <0,0001 |
| 0d:Ctrl. DMSO low vs. 9d:Ctrl. Taz | 654,9 | 1271 | **** | <0,0001 |
| 0d:Ctrl. DMSO low vs. 9d:Ctrl. Bex | 654,9 | 1087 | *** | 0,0005 |
| 0d:Ctrl. DMSO low vs. 9d:Ctrl. Taz/Bex | 654,9 | 1244 | **** | <0,0001 |
| 0d:Ctrl. DMSO medium vs. 6d:Ctrl. DMSO high | 679,3 | 1102 | *** | 0,0003 |
| 0d:Ctrl. DMSO medium vs. 6d:Ctrl. Taz | 679,3 | 1171 | **** | <0,0001 |
| 0d:Ctrl. DMSO medium vs. 6d:Ctrl. Taz/Bex | 679,3 | 1124 | *** | 0,0003 |
| 0d:Ctrl. DMSO medium vs. 9d:Ctrl. DMSO medium | 679,3 | 1040 | ** | 0,0059 |
| 0d:Ctrl. DMSO medium vs. 9d:Ctrl. DMSO high | 679,3 | 1147 | **** | <0,0001 |
| 0d:Ctrl. DMSO medium vs. 9d:Ctrl. Taz | 679,3 | 1271 | **** | <0,0001 |
| 0d:Ctrl. DMSO medium vs. 9d:Ctrl. Bex | 679,3 | 1087 | ** | 0,0017 |
| 0d:Ctrl. DMSO medium vs. 9d:Ctrl. Taz/Bex | 679,3 | 1244 | **** | <0,0001 |
| 0d:Ctrl. DMSO high vs. 6d:Ctrl. DMSO high | 680,6 | 1102 | *** | 0,0003 |
| 0d:Ctrl. DMSO high vs. 6d:Ctrl. Taz | 680,6 | 1171 | **** | <0,0001 |
| 0d:Ctrl. DMSO high vs. 6d:Ctrl. Taz/Bex | 680,6 | 1124 | *** | 0,0003 |

| | | | | |
|---|---|---|---|---|
| 0d:Ctrl. DMSO high vs. 9d:Ctrl. DMSO medium | 680,6 | 1040 | ** | 0,0063 |
| 0d:Ctrl. DMSO high vs. 9d:Ctrl. DMSO high | 680,6 | 1147 | **** | <0,0001 |
| 0d:Ctrl. DMSO high vs. 9d:Ctrl. Taz | 680,6 | 1271 | **** | <0,0001 |
| 0d:Ctrl. DMSO high vs. 9d:Ctrl. Bex | 680,6 | 1087 | ** | 0,0018 |
| 0d:Ctrl. DMSO high vs. 9d:Ctrl. Taz/Bex | 680,6 | 1244 | **** | <0,0001 |
| 0d:Ctrl. Taz vs. 3d:Ctrl. Taz | 609,6 | 921 | * | 0,0437 |
| 0d:Ctrl. Taz vs. 6d:Ctrl. DMSO medium | 609,6 | 927,9 | * | 0,0338 |
| 0d:Ctrl. Taz vs. 6d:Ctrl. DMSO high | 609,6 | 1102 | **** | <0,0001 |
| 0d:Ctrl. Taz vs. 6d:Ctrl. Taz | 609,6 | 1171 | **** | <0,0001 |
| 0d:Ctrl. Taz vs. 6d:Ctrl. Bex | 609,6 | 935,8 | * | 0,0444 |
| 0d:Ctrl. Taz vs. 6d:Ctrl. Taz/Bex | 609,6 | 1124 | **** | <0,0001 |
| 0d:Ctrl. Taz vs. 9d:Ctrl. DMSO low | 609,6 | 922,1 | * | 0,042 |
| 0d:Ctrl. Taz vs. 9d:Ctrl. DMSO medium | 609,6 | 1040 | *** | 0,0002 |
| 0d:Ctrl. Taz vs. 9d:Ctrl. DMSO high | 609,6 | 1147 | **** | <0,0001 |
| 0d:Ctrl. Taz vs. 9d:Ctrl. Taz | 609,6 | 1271 | **** | <0,0001 |
| 0d:Ctrl. Taz vs. 9d:Ctrl. Bex | 609,6 | 1087 | **** | <0,0001 |
| 0d:Ctrl. Taz vs. 9d:Ctrl. Taz/Bex | 609,6 | 1244 | **** | <0,0001 |
| 0d:Ctrl. Bex vs. 6d:Ctrl. DMSO high | 664,4 | 1102 | *** | 0,0004 |
| 0d:Ctrl. Bex vs. 6d:Ctrl. Taz | 664,4 | 1171 | **** | <0,0001 |
| 0d:Ctrl. Bex vs. 6d:Ctrl. Taz/Bex | 664,4 | 1124 | *** | 0,0004 |
| 0d:Ctrl. Bex vs. 9d:Ctrl. DMSO medium | 664,4 | 1040 | ** | 0,0066 |
| 0d:Ctrl. Bex vs. 9d:Ctrl. DMSO high | 664,4 | 1147 | **** | <0,0001 |
| 0d:Ctrl. Bex vs. 9d:Ctrl. Taz | 664,4 | 1271 | **** | <0,0001 |
| 0d:Ctrl. Bex vs. 9d:Ctrl. Bex | 664,4 | 1087 | ** | 0,0019 |
| 0d:Ctrl. Bex vs. 9d:Ctrl. Taz/Bex | 664,4 | 1244 | **** | <0,0001 |
| 0d:Ctrl. Taz/Bex vs. 6d:Ctrl. DMSO high | 702,5 | 1102 | ** | 0,0024 |
| 0d:Ctrl. Taz/Bex vs. 6d:Ctrl. Taz | 702,5 | 1171 | *** | 0,0002 |
| 0d:Ctrl. Taz/Bex vs. 6d:Ctrl. Taz/Bex | 702,5 | 1124 | ** | 0,002 |
| 0d:Ctrl. Taz/Bex vs. 9d:Ctrl. DMSO medium | 702,5 | 1040 | * | 0,0294 |
| 0d:Ctrl. Taz/Bex vs. 9d:Ctrl. DMSO high | 702,5 | 1147 | *** | 0,0003 |
| 0d:Ctrl. Taz/Bex vs. 9d:Ctrl. Taz | 702,5 | 1271 | **** | <0,0001 |
| 0d:Ctrl. Taz/Bex vs. 9d:Ctrl. Bex | 702,5 | 1087 | ** | 0,0091 |
| 0d:Ctrl. Taz/Bex vs. 9d:Ctrl. Taz/Bex | 702,5 | 1244 | **** | <0,0001 |
| 3d:Ctrl. DMSO low vs. 3d:Ctrl. DMSO high | 562,8 | 913,5 | ** | 0,0091 |
| 3d:Ctrl. DMSO low vs. 3d:Ctrl. Taz | 562,8 | 921 | ** | 0,0066 |
| 3d:Ctrl. DMSO low vs. 6d:Ctrl. DMSO medium | 562,8 | 927,9 | ** | 0,0049 |
| 3d:Ctrl. DMSO low vs. 6d:Ctrl. DMSO high | 562,8 | 1102 | **** | <0,0001 |
| 3d:Ctrl. DMSO low vs. 6d:Ctrl. Taz | 562,8 | 1171 | **** | <0,0001 |
| 3d:Ctrl. DMSO low vs. 6d:Ctrl. Bex | 562,8 | 935,8 | ** | 0,0074 |
| 3d:Ctrl. DMSO low vs. 6d:Ctrl. Taz/Bex | 562,8 | 1124 | **** | <0,0001 |
| 3d:Ctrl. DMSO low vs. 9d:Ctrl. DMSO low | 562,8 | 922,1 | ** | 0,0063 |
| 3d:Ctrl. DMSO low vs. 9d:Ctrl. DMSO medium | 562,8 | 1040 | **** | <0,0001 |
| 3d:Ctrl. DMSO low vs. 9d:Ctrl. DMSO high | 562,8 | 1147 | **** | <0,0001 |
| 3d:Ctrl. DMSO low vs. 9d:Ctrl. Taz | 562,8 | 1271 | **** | <0,0001 |
| 3d:Ctrl. DMSO low vs. 9d:Ctrl. Bex | 562,8 | 1087 | **** | <0,0001 |
| 3d:Ctrl. DMSO low vs. 9d:Ctrl. Taz/Bex | 562,8 | 1244 | **** | <0,0001 |
| 3d:Ctrl. DMSO medium vs. 6d:Ctrl. DMSO high | 762,2 | 1102 | * | 0,0143 |

| | | | | |
|---|---|---|---|---|
| 3d:Ctrl. DMSO medium vs. 6d:Ctrl. Taz | 762,2 | 1171 | ** | 0,0016 |
| 3d:Ctrl. DMSO medium vs. 6d:Ctrl. Taz/Bex | 762,2 | 1124 | * | 0,0116 |
| 3d:Ctrl. DMSO medium vs. 9d:Ctrl. DMSO high | 762,2 | 1147 | ** | 0,002 |
| 3d:Ctrl. DMSO medium vs. 9d:Ctrl. Taz | 762,2 | 1271 | **** | <0,0001 |
| 3d:Ctrl. DMSO medium vs. 9d:Ctrl. Bex | 762,2 | 1087 | * | 0,0472 |
| 3d:Ctrl. DMSO medium vs. 9d:Ctrl. Taz/Bex | 762,2 | 1244 | **** | <0,0001 |
| 3d:Ctrl. DMSO high vs. 9d:Ctrl. Taz | 913,5 | 1271 | * | 0,0138 |
| 3d:Ctrl. DMSO high vs. 9d:Ctrl. Taz/Bex | 913,5 | 1244 | * | 0,038 |
| 3d:Ctrl. Taz vs. 9d:Ctrl. Taz | 921 | 1271 | * | 0,0185 |
| 3d:Ctrl. Taz vs. 9d:Ctrl. Taz/Bex | 921 | 1244 | * | 0,0495 |
| 3d:Ctrl. Bex vs. 6d:Ctrl. DMSO high | 751,4 | 1102 | * | 0,0179 |
| 3d:Ctrl. Bex vs. 6d:Ctrl. Taz | 751,4 | 1171 | ** | 0,0022 |
| 3d:Ctrl. Bex vs. 6d:Ctrl. Taz/Bex | 751,4 | 1124 | * | 0,0142 |
| 3d:Ctrl. Bex vs. 9d:Ctrl. DMSO high | 751,4 | 1147 | ** | 0,0028 |
| 3d:Ctrl. Bex vs. 9d:Ctrl. Taz | 751,4 | 1271 | **** | <0,0001 |
| 3d:Ctrl. Bex vs. 9d:Ctrl. Taz/Bex | 751,4 | 1244 | **** | <0,0001 |
| 3d:Ctrl. Taz/Bex vs. 9d:Ctrl. Taz | 836,1 | 1271 | ** | 0,0011 |
| 3d:Ctrl. Taz/Bex vs. 9d:Ctrl. Taz/Bex | 836,1 | 1244 | ** | 0,0035 |
| 6d:Ctrl. DMSO low vs. 6d:Ctrl. DMSO high | 699 | 1102 | *** | 0,0008 |
| 6d:Ctrl. DMSO low vs. 6d:Ctrl. Taz | 699 | 1171 | **** | <0,0001 |
| 6d:Ctrl. DMSO low vs. 6d:Ctrl. Taz/Bex | 699 | 1124 | *** | 0,0007 |
| 6d:Ctrl. DMSO low vs. 9d:Ctrl. DMSO medium | 699 | 1040 | * | 0,0137 |
| 6d:Ctrl. DMSO low vs. 9d:Ctrl. DMSO high | 699 | 1147 | **** | <0,0001 |
| 6d:Ctrl. DMSO low vs. 9d:Ctrl. Taz | 699 | 1271 | **** | <0,0001 |
| 6d:Ctrl. DMSO low vs. 9d:Ctrl. Bex | 699 | 1087 | ** | 0,004 |
| 6d:Ctrl. DMSO low vs. 9d:Ctrl. Taz/Bex | 699 | 1244 | **** | <0,0001 |
| 6d:Ctrl. DMSO medium vs. 9d:Ctrl. Taz | 927,9 | 1271 | * | 0,0241 |
| 9d:Ctrl. DMSO low vs. 9d:Ctrl. Taz | 922,1 | 1271 | * | 0,0193 |
