# Supplementary material for: Drug screening identifies tazarotene and bexarotene as therapeutic agents in multiple sulfatase deficiency
Source: EMBO Mol Med. 2023 Feb 15;15(3):e14837. doi: 10.15252/emmm.202114837 (PMC9994482; doi:10.15252/emmm.202114837)
Supplement: Supplementary file 2 — Expanded View Figures PDF [file EMMM-15-e14837-s012.pdf]

Expanded View Figures

Figure EV1. Hit drug evaluation on immortalized MSD fibroblasts.

A ARSA activity quantification (nmol/h/mg) after treatment of MSDi cells in 25 cm^2 cell culture flasks with a selection of four positive hit drugs at a final concentration of 10 μM for 6 days. Data represent mean ± SD of seven independent experiments (biological replicates). One-way ANOVA followed by Tukey's test for multiple comparisons. Difference against negative control: ****$P < 0.0001$. See details on P-values in Appendix Table S31.

B GALNS activity quantification (nmol/h/mg) after treatment of MSDi cells with a selection of four positive hit drugs at a final concentration of 10 μM for 3 days. Data represent mean ± SD of five independent experiments (biological replicates). One-way ANOVA followed by Tukey's test for multiple comparisons. Difference against negative control: **$P < 0.001$. See details on P-values in Appendix Table S32.

C GALNS activity quantification (nmol/h/mg) after treatment of MSDi cells with a selection of four positive hit drugs at a final concentration of 10 μM for 6 days. Data represent mean ± SD of five independent experiments (biological replicates). One-way ANOVA followed by Tukey's test for multiple comparisons. Difference against negative control: * $P < 0.05$, ** $P < 0.001$. See details on P-values in Appendix Table S33.

D β-galactosidase (betaGAL) activity quantification (nmol/h/mg) after treatment of MSDi cells with a selection of four positive hit drugs at a final concentration of 10 μM for 3 days. Data represent mean ± SD of five independent experiments (biological replicates). One-way ANOVA followed by Tukey's test for multiple comparisons.

E ß-hexosaminidase A and B (betaHEXAB) activity quantification (nmol/h/mg) after treatment of MSDi cells with a selection of four positive hit drugs at a final concentration of 10 μM for 3 days. Data represent mean ± SD of five independent experiments (biological replicates). One-way ANOVA followed by Tukey's test for multiple comparisons.

F Dose-response curve of ARSA activity calculated from data displayed in Fig 1C (MSDi cells, tazarotene treatment) by nonlinear regression analysis. Drug concentrations are displayed after transformation into log10 values and baseline activity (negative control, DMSO-only treatment) was manually referred to log-2. Dots and error bars represent mean ± SD.

G Dose-response curve of GALNS activity calculated from data displayed in Fig 1D (MSDi cells, tazarotene treatment) by nonlinear regression analysis. Drug concentrations are displayed after transformation into log10 values and baseline activity (negative control, DMSO-only treatment) was manually referred to log-2. Dots and error bars represent mean ± SD.

H Dose-response curve of ARSA activity calculated from data displayed in Fig 1F (MSDi cells, bexarotene treatment) by nonlinear regression analysis. Drug concentrations are displayed after transformation into log10 values and baseline activity (negative control, DMSO-only treatment) was manually referred to log-2. Dots and error bars represent mean ± SD.

I Dose-response curve of ARSA activity calculated from data displayed in Fig 1G (MSDi cells, tazarotene/bexarotene treatment) by nonlinear regression analysis. Drug concentrations are displayed after transformation into log10 values and baseline activity (negative control, DMSO-only treatment) was manually referred to log-2. Dots and error bars represent mean ± SD.

Source data are available online for this figure.

 © 2023 The Authors

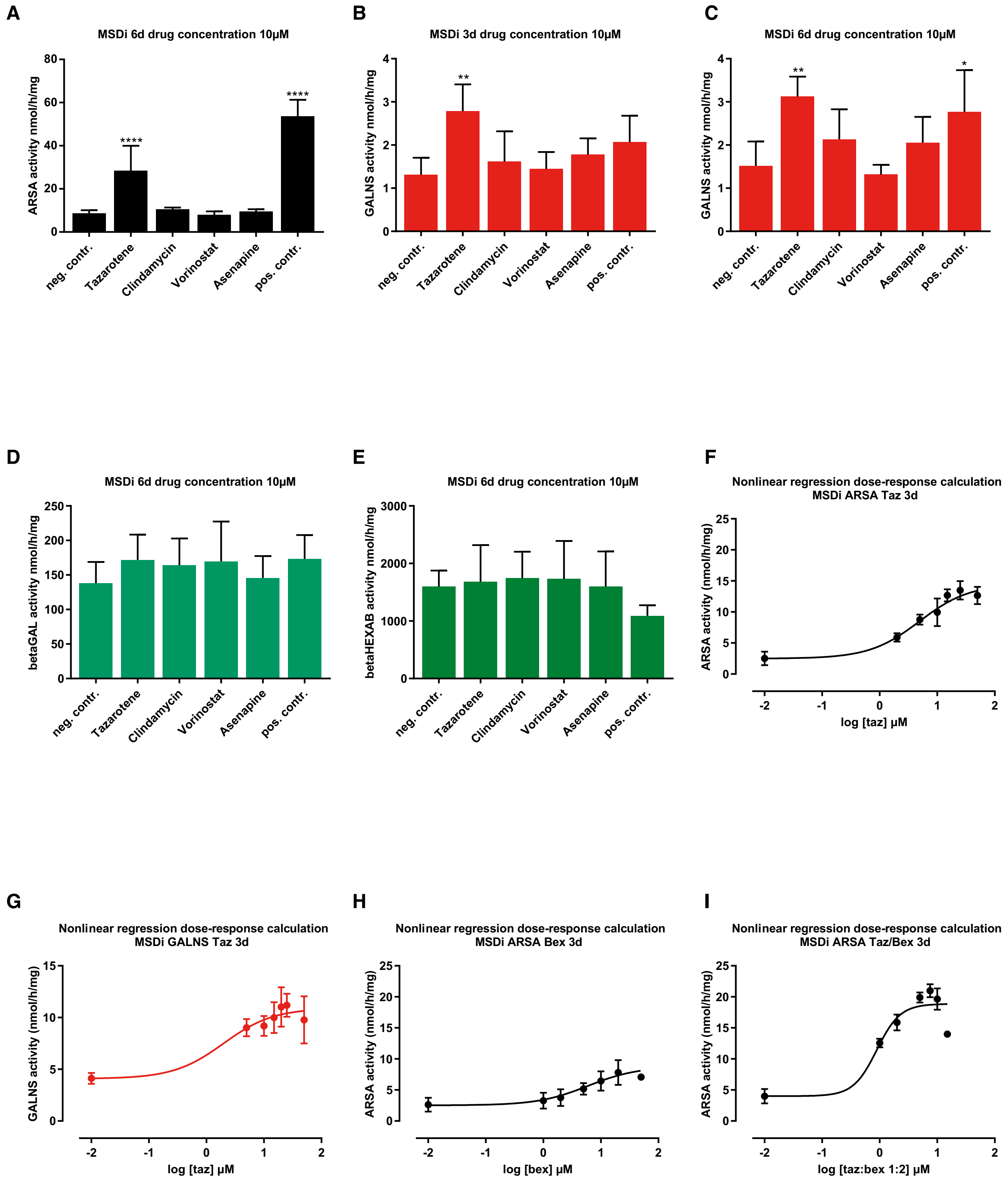
A
MSDi 6d drug concentration 10µM
ARSA activity nmol/h/mg
****
****
neg. contr.
Tazarotene
Clindamycin
Vorinostat
Asenapine
pos. contr.
B
MSDi 3d drug concentration 10µM
GALNS activity nmol/h/mg
**
C
MSDi 6d drug concentration 10µM
GALNS activity nmol/h/mg
**
*
D
MSDi 6d drug concentration 10µM
betaGAL activity nmol/h/mg
E
MSDi 6d drug concentration 10µM
betaHEXAB activity nmol/h/mg
F
Nonlinear regression dose-response calculation
MSDi ARSA Taz 3d
ARSA activity (nmol/h/mg)
log [taz] µM
G
Nonlinear regression dose-response calculation
MSDi GALNS Taz 3d
GALNS activity (nmol/h/mg)
log [taz] µM
H
Nonlinear regression dose-response calculation
MSDi ARSA Bex 3d
ARSA activity (nmol/h/mg)
log [bex] µM
I
Nonlinear regression dose-response calculation
MSDi ARSA Taz/Bex 3d
ARSA activity (nmol/h/mg)
log [taz:bex 1:2] µM

Figure EV1.

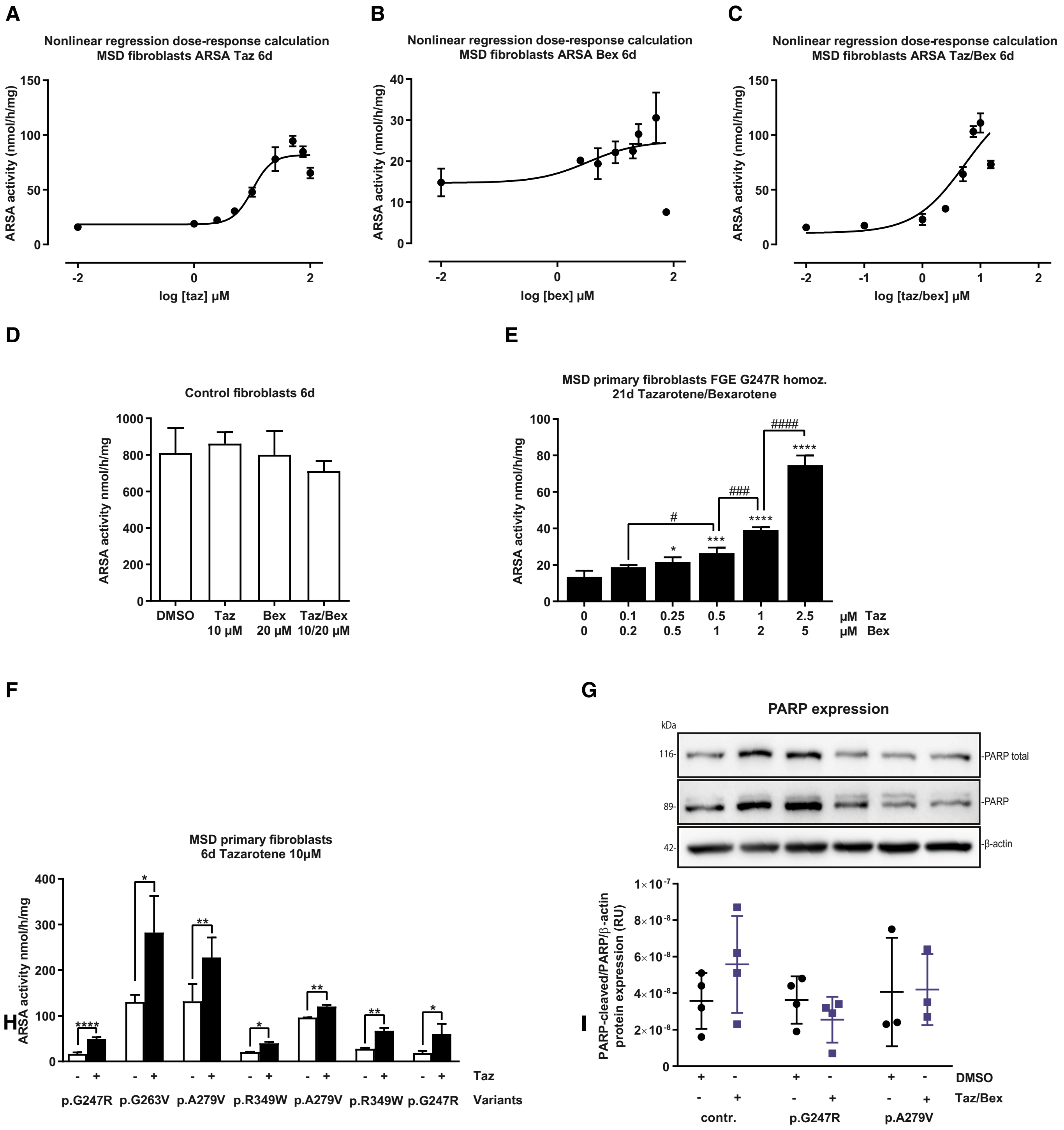

Figure EV2.

 © 2023 The Authors

Figure EV2. Treatment response and toxicity assessment.

A Dose-response curve of ARSA activity calculated from data displayed in Fig 2A (variant FGE Gly247Arg homozygous, tazarotene treatment) by nonlinear regression analysis. Drug concentrations are displayed after transformation into log10 values and baseline activity (negative control, DMSO-only treatment) was manually referred to log-2. Dots and error bars represent mean ± SD.

B Dose-response curve of ARSA activity calculated from data displayed in Fig 2B (variant FGE Gly247Arg homozygous, bexarotene treatment) by nonlinear regression analysis. Drug concentrations are displayed after transformation into log10 values and baseline activity (negative control, DMSO-only treatment) was manually referred to log-2. Dots and error bars represent mean ± SD.

C Dose-response curve of ARSA activity calculated from data displayed in Fig 2C (variant FGE Gly247Arg homozygous, tazarotene/bexarotene treatment) by nonlinear regression analysis. Drug concentrations are displayed after transformation into log10 values and baseline activity (negative control, DMSO-only treatment) was manually referred to log-2. Dots and error bars represent mean ± SD.

D ARSA activity quantification after treatment of five different control, non-MSD, fibroblast lines with tazarotene, bexarotene, and tazarotene/bexarotene in combination for 6 days referred to β-actin amounts and calculation of ARSA activity based on ARSA protein amount (specific ARSA activity). Data represent mean ± SD of five independent experiments (biological replicates) in duplicates to determine the range of normal ARSA activities and treatment response as the basis for the calculation of residual activities in MSD fibroblasts.

E ARSA activity quantification after simultaneous treatment of MSD primary fibroblasts (variant FGE Gly247Arg homozygous) with increasing concentrations of tazarotene and bexarotene in a fixed combination of 1:2 for 21 days. Data represent mean ± SD of four independent experiments (biological replicates). One-way ANOVA followed by Tukey's test for multiple comparisons. Displayed are significance levels for the next significant difference between adjacent concentrations/conditions. # $P < 0.05$, ### $P < 0.001$, #### $P < 0.0001$. Difference against 0/0 μM control: *$P < 0.05$, ***$P < 0.001$, ****$P < 0.0001$. See details on *P*-values in Appendix Table S34.

F Quantification of ARSA activities in MSD primary fibroblasts with different homozygous *SUMF1* mutations (FGE Gly247Arg, FGE Gly263Val, FGE Ala279Val, FGE Arg349Trp) after 6 days of treatment with tazarotene 10 μM. Data represent mean ± SD of 2–5 independent experiments (biological replicates). One-way ANOVA followed by Tukey's test for multiple comparisons. *$P < 0.05$, **$P < 0.01$, ****$P < 0.0001$. See details on *P*-values in Appendix Table S35.

G Upper panel: Representative pictures of Western Blot analysis of (PARP) and cleaved PARP in tazarotene/bexarotene-treated MSD primary fibroblasts (variant FGE Gly247Arg, FGE Ala279Val, homozygous) and control fibroblasts. β-actin expression served as loading control. Lower panel: Quantification of protein amounts from western blots displayed as ratio cleaved PARP to total PARP expression normalized to β-actin. Data represent mean ± SD of 3–4 independent experiments (biological replicates). Unpaired *t*-tests. No statistical differences. RU, relative units.

Source data are available online for this figure.

Figure EV3. Transcriptional response of MSD and retinoic acid gene targets upon tazarotene/bexarotene treatment.

A Gene expression analysis of genes in relation to retinoic acid receptor signaling of six different MSD primary fibroblast lines and five different control fibroblast lines after 6 days of treatment with tazarotene/bexarotene (10/20 μM) and DMSO, respectively. Changes in RPKM (reads per kilobase million) are displayed as mean ± SD of three independent experiments (biological replicates). One-way ANOVA test followed by Tukey's test for multiple comparisons. *$P < 0.05$, **$P < 0.01$, ***$P < 0.001$, ****$P < 0.0001$. See details on *P*-values in Appendix Table S36.

B Gene expression analysis of *SUMF1*- and FGE-interacting partners (nonsulfatases) of six different MSD primary fibroblast lines and five different control fibroblast lines after 6 days of treatment with tazarotene/bexarotene (10/20 μM) and DMSO, respectively. Changes in RPKM (reads per kilobase million) are displayed as mean ± SD of three independent experiments (biological replicates). One-way ANOVA test followed by Tukey's test for multiple comparisons. *$P < 0.05$. See details on *P*-values in Appendix Table S37.

C Gene expression analysis of sulfatases of six different MSD primary fibroblast lines and five different control fibroblast lines after 6 days of treatment with tazarotene/bexarotene (10/20 μM) and DMSO, respectively. Changes in RPKM (reads per kilobase million) are displayed as mean ± SD of three independent experiments (biological replicates). One-way ANOVA test followed by Tukey's test for multiple comparisons. *$P < 0.05$, ****$P < 0.0001$. See details on *P*-values in Appendix Table S38.

Source data are available online for this figure.

© 2023 The Authors

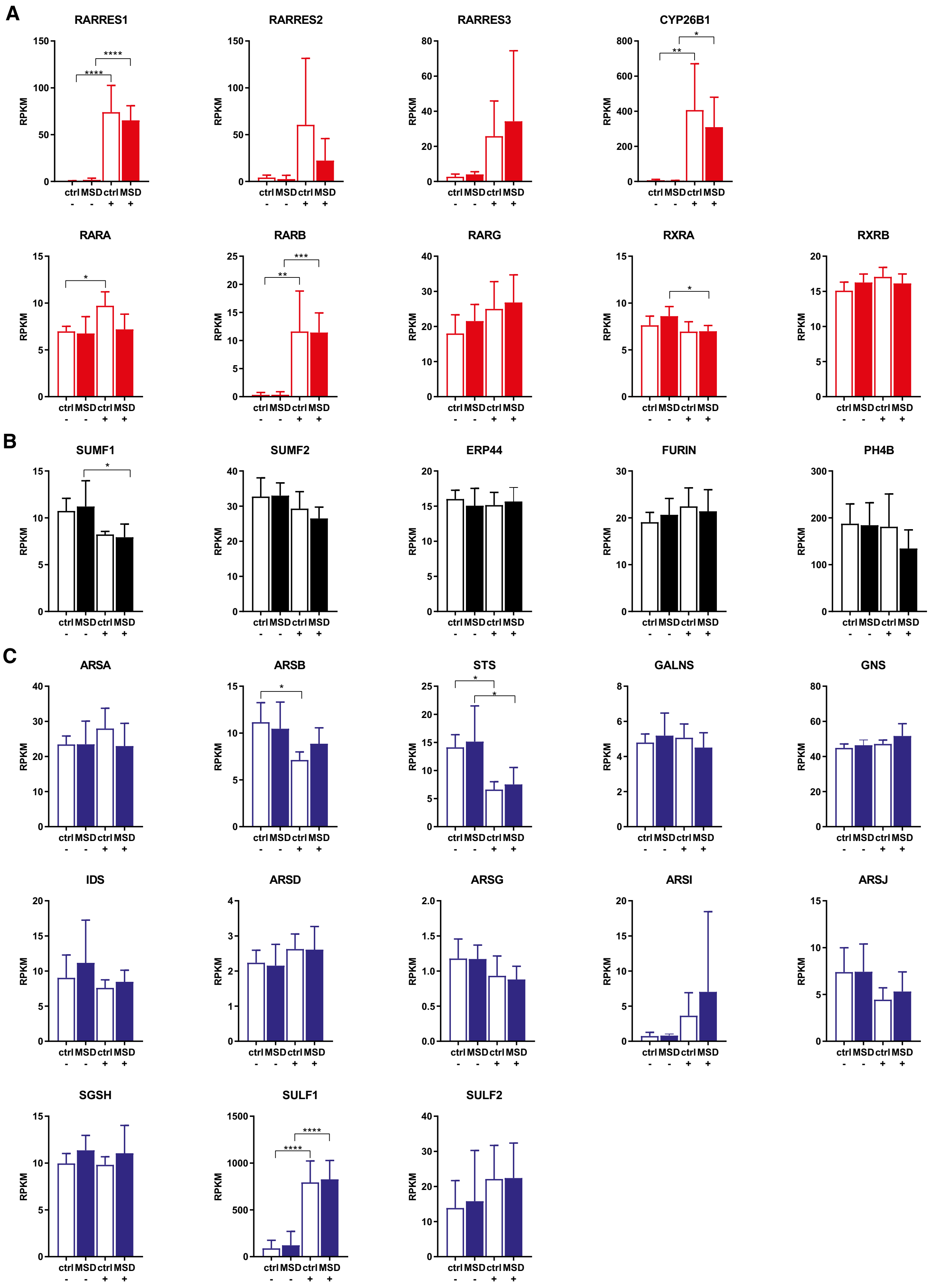

Figure EV3.

 © 2023 The Authors

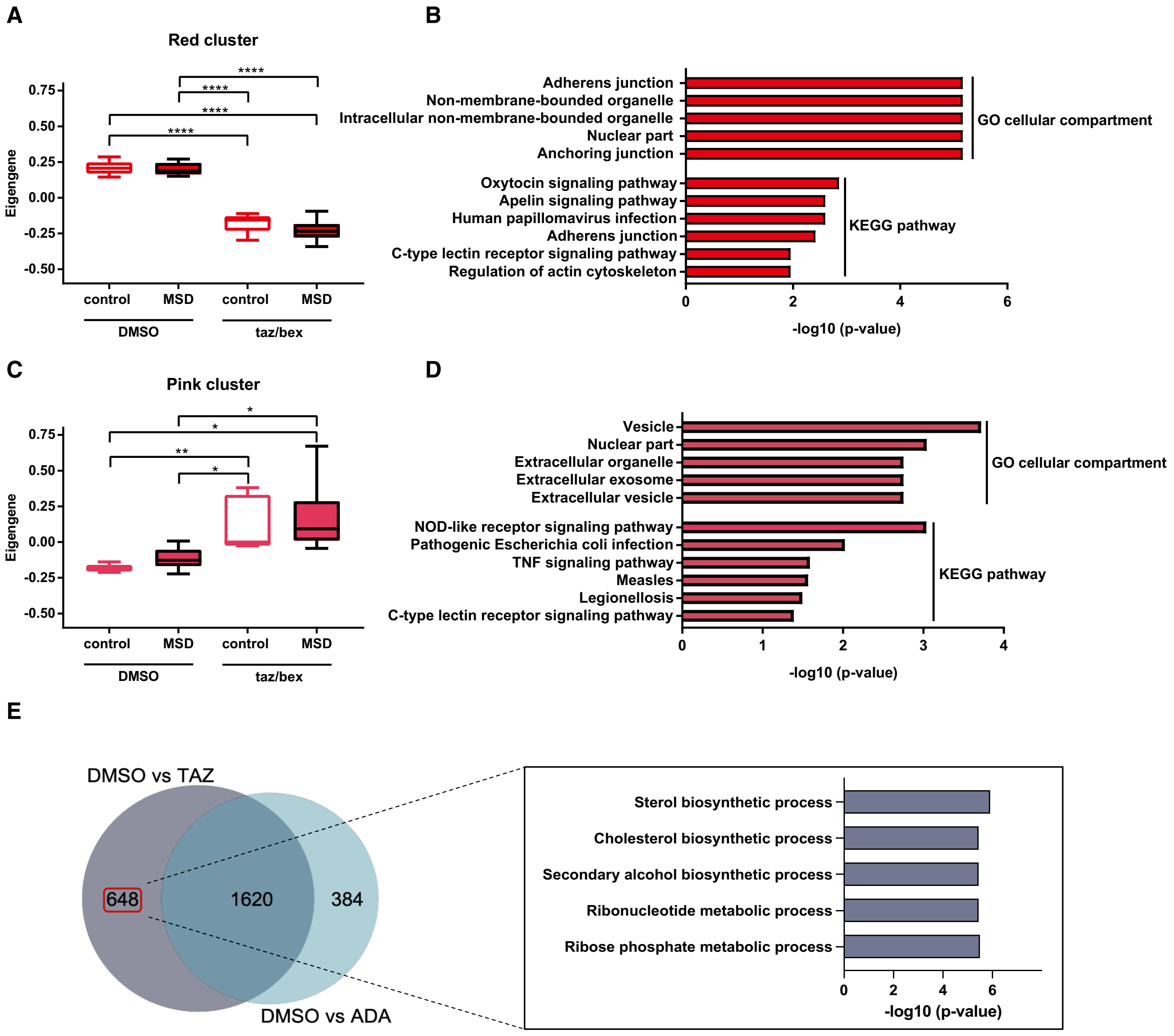

Figure EV4. Transcriptional response upon tazarotene and bexarotene treatment in MSD and control fibroblasts and differential transcriptional response in MSD fibroblasts upon treatment with tazarotene and adapalene.

A Eigengene analysis of the red gene cluster as determined by WGCN analysis after RNA sequencing of six different MSD primary fibroblast lines and five different control fibroblast lines and treatment with tazarotene/ bexarotene 10/20 μM or DMSO only, respectively, for 6 days. Data represent min to max box and whisker blots of Eigengene values ± SD of three independent experiments (biological replicates). One-way ANOVA test followed by Tukey's test for multiple comparisons. ****$P < 0.0001$. See details on P-values in Appendix Table S39.

B GO and KEGG pathway analysis of genes in the red cluster and log10 value of P-values. GO and KEGG pathway analysis of genes in the yellow cluster and their log10 value of P-values as the display for changes in gene expression.

C Eigengene analysis of the red gene cluster as determined by WGCN analysis after RNA sequencing of six different MSD primary fibroblast lines and five different control fibroblast lines and treatment with tazarotene/bexarotene 10/20 μM or DMSO only, respectively, for 6 days. Data represent min to max box and whisker blots of Eigengene values ± SD of three independent experiments (biological replicates). One-way ANOVA test followed by Tukey's test for multiple comparisons. *$P < 0.05$, **$P < 0.01$. See details on P-values in Appendix Table S40.

D GO and KEGG pathway analysis of genes in the red cluster and log10 value of P-values.

E Differential gene expression analysis after treatment of seven MSD primary fibroblast lines with tazarotene (sulfatase activity response) and adapalene (no sulfatase activity response) in triplicates for 6 days. Treatment with DMSO served as a negative control. Venn diagram and number of exclusively regulated genes for tazarotene treatment (TAZ) versus DMSO condition (left) and adapalene treatment (ADA) versus DMSO (right), as well as the number of overlapping genes identically regulated by both tazarotene and adapalene. GO pathway analysis and log10 value of P-values for tazarotene-only regulated genes.

Source data are available online for this figure.

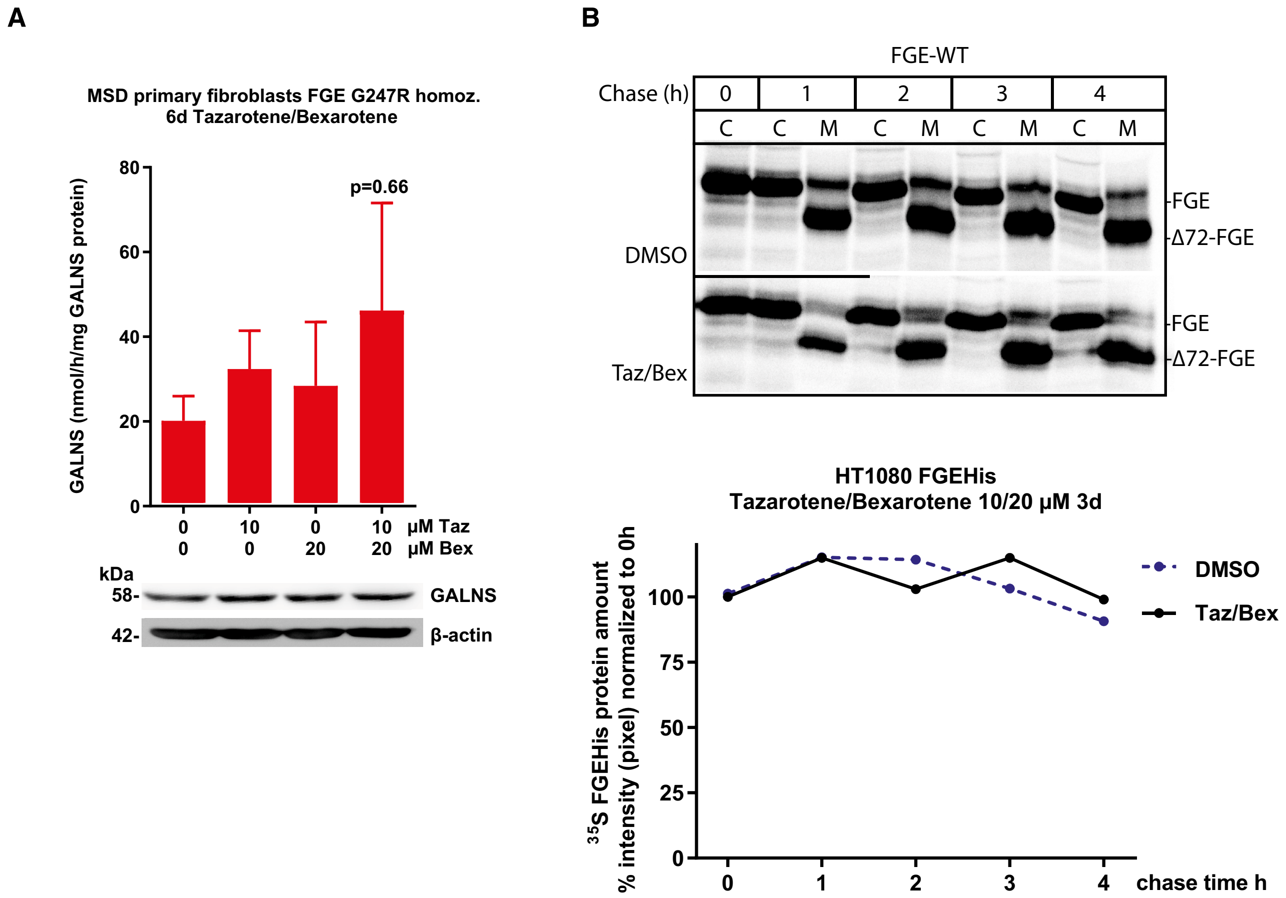

Figure EV5. Sulfatase-specific activity and protein expression and protein stability of wildtype FGE upon treatment.

A GALNS protein amount quantification after treatment of MSD primary fibroblasts (variant FGE Gly247Arg homozygous) with tazarotene, bexarotene, and tazarotene/bexarotene in combination for 6 days referred to β-actin amounts and calculation of GALNS activity based on GALNS protein amount (specific GALNS activity). Data represent mean ± SD three independent experiments (biological replicates). One-way ANOVA followed by Tukey's test for multiple comparisons.

B Pulse-chase-experiment in HT 1080 FGE wild-type (wt) cells after pretreatment with tazarotene/bexarotene and DMSO (control) for 3 days. Upper panel: autoradiogram of intracellular (full-length FGE, C) and cleaved and secreted (Δ72 FGE, M) ^{35}S isotope labeled FGE protein in either condition with a chase time of 4 h. Lower panel: Quantification of the autoradiogram, $n = 1$ experiment.

Source data are available online for this figure.

 © 2023 The Authors
