# Supplementary material for: Drug screening identifies tazarotene and bexarotene as therapeutic agents in multiple sulfatase deficiency
Source: EMBO Mol Med. 2023 Feb 15;15(3):e14837. doi: 10.15252/emmm.202114837 (PMC9994482; doi:10.15252/emmm.202114837)
Supplement: Supplementary file 6 — PDF+ [file EMMM-15-e14837-s010.pdf]

Article

SOURCE DATA | TRANSPARENT PROCESS | OPEN ACCESS

EMBO Molecular Medicine

Drug screening identifies tazarotene and bexarotene as therapeutic agents in multiple sulfatase deficiency

Lars Schlotawa[1,*], Karolina Tyka[1], Matthias Kettwig[1], Rebecca C Ahrens-Nicklas[2], Matthias Baud[3], Tea Berulava[4], Nicola Brunetti-Pierri[5,6], Alyssa Gagne[7,8], Zackary M Herbst[9], Jean A Maguire[7,8], Jlenia Monfregola[5,6], Tonatiuh Pena[4,10], Karthikeyan Radhakrishnan[11], Sophie Schröder[4], Elisa A Waxman[7,8], Andrea Ballabio[5,6,12], Thomas Dierks[11], André Fischer[4,13,14], Deborah L French[7,8], Michael H Gelb[9] & Jutta Gärtner[1]

Abstract

Multiple sulfatase deficiency (MSD, MIM #272200) results from pathogenic variants in the SUMF1 gene that impair proper function of the formylglycine-generating enzyme (FGE). FGE is essential for the posttranslational activation of cellular sulfatases. MSD patients display reduced or absent sulfatase activities and, as a result, clinical signs of single sulfatase disorders in a unique combination. Up to date therapeutic options for MSD are limited and mostly palliative. We performed a screen of FDA-approved drugs using immortalized MSD patient fibroblasts. Recovery of arylsulfatase A activity served as the primary readout. Subsequent analysis confirmed that treatment of primary MSD fibroblasts with tazarotene and bexarotene, two retinoids, led to a correction of MSD pathophysiology. Upon treatment, sulfatase activities increased in a dose- and time-dependent manner, reduced glycosaminoglycan content decreased and lysosomal position and size normalized. Treatment of MSD patient derived induced pluripotent stem cells (iPSC) differentiated into neuronal progenitor cells (NPC) resulted in a positive treatment response. Tazarotene and bexarotene act to ultimately increase the stability of FGE variants. The results lay the basis for future research on the development of a first therapeutic option for MSD patients.

Keywords drug screening; formylglycine-generating enzyme; lysosomal disorder; retinoids; sulfatase-modifying factor 1
Subject Categories Genetics, Gene Therapy & Genetic Disease; Neuroscience
DOI 10.15252/emmm.202114837 | Received 12 July 2021 | Revised 9 December 2022 | Accepted 9 January 2023 | Published online 15 February 2023
EMBO Mol Med (2023) 15: e14837

Introduction

Multiple sulfatase deficiency (MSD, MIM #272200) is an ultra-rare lysosomal disorder caused by pathogenic variants in the *SUMF1* gene encoding the formylglycine-generating enzyme (FGE; Cosma *et al*, 2003; Dierks *et al*, 2003). FGE is localized in the endoplasmic reticulum (ER) and is required for the activation of all newly synthesized sulfatases. FGE oxidizes a conserved cysteine in the active site of every sulfatase to formylglycine, which is required for catalytic activity (Dierks *et al*, 2005). Sulfatases are a group of 17 enzymes in humans necessary for the catalytic breakdown of sulfated substrates. The majority are localized in lysosomes, while others are found in the ER, Golgi, and on the cell surface (Diez-Roux & Ballabio, 2005). Most *SUMF1* pathogenic variants are single amino acid substitutions that lead to FGE protein misfolding (Schlotawa *et al*, 2011, 2020). Improperly folded FGE protein retains some

1 Department of Paediatrics and Adolescent Medicine, University Medical Centre Göttingen, Göttingen, Germany
2 Division of Human Genetics and Metabolism, The Children's Hospital of Philadelphia, Philadelphia, PA, USA
3 School of Chemistry and Institute for Life Sciences, University of Southampton, Southampton, UK
4 Department for Epigenetics and Systems Medicine in Neurodegenerative Diseases, German Centre for Neurodegenerative Diseases, Göttingen, Germany
5 Telethon Institute of Genetics and Medicine, Pozzuoli, Italy
6 Department of Translational Medicine, University of Naples Federico II, Naples, Italy
7 Center for Cellular and Molecular Therapeutics, The Children's Hospital of Philadelphia, Philadelphia, PA, USA
8 Department of Pathology and Laboratory Medicine, The Children's Hospital of Philadelphia, Philadelphia, PA, USA
9 Department of Chemistry, University of Washington, Seattle, WA, USA
10 Bioinformatics Unit, German Centre for Neurodegenerative Diseases, Göttingen, Germany
11 Faculty of Chemistry, Biochemistry I, Bielefeld University, Bielefeld, Germany
12 Department of Molecular and Human Genetics and Neurological Research Institute, Baylor College of Medicine, Houston, TX, USA
13 Department of Psychiatry and Psychotherapy, University Medical Center Göttingen, Göttingen, Germany
14 Multiscale Bioimaging Cluster of Excellence, University Medical Center Göttingen, University of Göttingen, Göttingen, Germany
*Corresponding author. Tel: +49551398036; E-mail: lars.schlotawa@med.uni-goettingen.de

© 2023 The Authors. Published under the terms of the CC BY 4.0 license.

residual activity, leading to some degree of downstream residual sulfatase activities (Schlotawa *et al*, 2011). Misfolded FGE variants interact with protein disulfide isomerase (PDI) in the ER where PDI targets non-natural disulfide bridges formed in FGE because of misfolding and determines early degradation of FGE variants. Knockdown or pharmacological inhibition of PDI partially rescues sulfatase activities in MSD patient-derived cells (Schlotawa *et al*, 2018).

The combined and variable deficiency of all cellular sulfatases leads to a complex clinical presentation in MSD patients with signs of single sulfatase deficiencies like metachromatic leukodystrophy (MLD), several mucopolysaccharidosis subtypes (MPS II, IIIa, IIId, IVa, VI), X-linked recessive chondrodysplasia punctata type 1 (CDPX1), and X-linked ichthyosis (XLI) (Adang *et al*, 2020; Cappuccio *et al*, 2020; Schlotawa *et al*, 2020; Verheyen *et al*, 2021). MSD is an early-onset progressive disease. Natural disease history data reveal that the mean survival of MSD patients is 13 years. Currently, more than 50 patients are known worldwide and nearly 150 MSD cases have been described in the literature since its first characterization (Adang *et al*, 2020; Cappuccio *et al*, 2020; Schlotawa *et al*, 2020). MSD disease severity correlates with *SUMF1* mutation severity: unstable FGE variants with extremely reduced activity cause severe forms of MSD, whereas variants with higher residual activity and stability result in attenuated phenotypes (Adang *et al*, 2020; Schlotawa *et al*, 2020). The most severe cases were found to entirely lack FGE function (Busche *et al*, 2009; Schlotawa *et al*, 2019).

There is currently no disease-modifying therapy for MSD and the only treatment options are symptomatic and palliative (Ahrens-Nicklas *et al*, 2018). One potential drug development strategy in ultra-rare disorders such as MSD is drug repurposing, alternatively called repositioning. Licensed drugs are screened for their potential as a treatment for different diseases beyond their original indication. Because safety and efficacy data have been obtained in previous studies and do not necessarily need to be generated again, drug repurposing is time-effective but also cost-effective compared with the development of new drugs. This is especially attractive for treating rare diseases with comparatively low commercial interest and devastating diseases without any existing therapy (Strittmatter, 2014; Pushpakom *et al*, 2019).

In this study, we present the results from a high-throughput phenotypic screen of 785 FDA-approved drugs on MSD patient cells, resulting in the discovery of two structurally and mechanistically related retinoid drugs reversing the cellular MSD phenotype.

Results

A screen of 785 FDA-approved drugs reveals 13 hits that increase arylsulfatase A activity in MSD patient cells

In preparation for our drug screen, we adopted a method developed by Geng *et al* based on a diagnostic routine arylsulfatase A (ARSA) activity assay for use in 96-well plates with lysates of MSD patient-derived cells (see Appendix Supplementary Methods for details and Fig S1A; Baum *et al*, 1959; Geng *et al*, 2011). This spectrophotometric assay detects changes in optical density (OD) at 515 nm when a sulfate from the synthetic substrate p-nitrocatechol sulfate (pNCS) is enzymatically cleaved off by ARSA, resulting in the generation of the colored p-nitrocatechol (pNC) product as a quantifiable readout. In this assay, an increase in OD indicates higher pNC levels, correlating with an increased ARSA activity (Baum *et al*, 1959). We used an immortalized MSD patient-derived primary fibroblast line (MSDi) with a homozygous *SUMF1* missense mutation (c.463C > T, p.Ser155Pro; Cosma *et al*, 2003).

Mean baseline OD of all dimethyl sulfoxide (DMSO) treated controls was 0.03 (standard deviation (SD) 0.004, median 0.03, minimum 0.01, maximum 0.04, $n = 108$, Appendix Fig S1B). MSDi cells stably expressing FGEHis wild-type protein thereby rescuing ARSA activity served as a positive control and were used to determine the upper OD limit (mean 0.16, SD 0.04, median 0.15, minimum 0.09, maximum 0.25, $n = 28$).

We performed a primary screen using an FDA-approved drug library that was gifted by LifeArc, London, UK, containing 785 licensed drugs (1 mM stocks in DMSO, see details in the experimental section) at a final concentration of 10 μM (1% DMSO content). We identified 13 drugs that exceeded the upper limit of baseline OD values in lysates of treated cells (Fig 1A, Appendix Figs S1C and D, S2, and S3).

Drugs that resulted in ODs below baseline impaired cell viability and were screened again at a final concentration of 1 and 0.1 μM but revealed no further hits (Appendix Fig S4A and B). All hit drugs were counterscreened under standard assay conditions, devoid of cells to detect any interference with the ARSA assay and artificial OD increase, but no such false positive hits were detected (Appendix Fig S4C). We chose tazarotene, clindamycin, vorinostat, and asenapine as the first selection of hit drugs to be included in follow-up experiments.

Tazarotene and bexarotene effectively increase the activity of lysosomal sulfatases in immortalized MSD patient cells

To analyze whether the treatment response was reproducible outside of the 96-well format, MSDi cells were treated with fresh stocks of commercially available selected hit drugs at a final concentration of 10 μM of each for 3 and 6 days in cell culture flasks. Samples were analyzed by standard diagnostic lysosomal enzyme activity assays in cell lysates (see Materials and Methods for details). Only tazarotene showed a significant increase in ARSA and N-acetylgalactosamine-6-sulfatase (GALNS) activity assays (Figs 1B and EV1A–C). Activities of nonsulfatase lysosomal hydrolases β-hexosaminidase A and B (betaHEXAB) and β-galactosidase (betaGAL) did not significantly change upon drug treatment compared with DMSO-only treated controls (Fig EV1D and E). MSDi cells, treated for 3 days with different tazarotene concentrations, displayed a dose-dependent significant increase in ARSA activities as compared to baseline. Increased activity was noted at a drug concentration as low as 2 μM for ARSA and 5 μM for GALNS (ARSA activity: EC50 4.9 μM; GALNS activity: EC50 2 μM, Figs 1C and D, and EV1F and G).

Tazarotene belongs to the 3rd generation of retinoids, compounds synthesized from vitamin A (Khalil *et al*, 2017). To analyze the potential of other retinoids to increase ARSA activity in MSDi cells, we used compounds of every retinoid generation applying the same treatment conditions. In addition, tazarotenic acid, the biologically active form of tazarotene after first-pass metabolism in organisms

 © 2023 The Authors

(Tang-Liu *et al*, 1999), was included. Besides tazarotenic acid, 3rd generation retinoid bexarotene and 2nd generation retinoid isotretinoin led to a significant ARSA activity increase as compared to the control treatment. Other retinoids failed to increase ARSA activity significantly (Fig 1E). Based on these results, we next tested bexarotene for a dose-dependent effect on ARSA activity in MSDi cells and detected a significant increase (EC50 5.9 μM, Figs 1F and EV1H). Tazarotene and bexarotene are well-known cell active agonists of retinoic acid receptors (RAR) and retinoid X receptors (RXR), respectively (Miller *et al*, 1997; Hofmann *et al*, 1999). We investigated the effect of treatment on ARSA activity with a fixed combination of tazarotene and bexarotene at a ratio of 1:2, chosen based on doses that previously increased ARSA activity when given individually. We observed a dose-dependent, significant increase in ARSA activity starting at concentrations as low as 1 μM tazarotene and 2 μM bexarotene. (ARSA activity: EC50 0.9/1.8 μM tazarotene/bexarotene, Figs 1G and EV1I). Finally, we used 10/20 μM tazarotene/bexarotene for analyzing a time-dependent response of ARSA activity. ARSA activity went up 7.6-fold to a maximum of 25.8 nmol/h/mg (SD 2.3) after 9 days of treatment (Fig 1H).

Tazarotene and bexarotene increase sulfatase activities and reduce LAMP1 staining and GAG storage in primary MSD patient cells

To investigate the effect of tazarotene and bexarotene on primary, nonimmortalized MSD fibroblasts, we treated a previously described patient-derived fibroblast line with the severe homozygous *SUMF1* mutation (c.739G > C, pGly.247Arg; Schlotawa *et al*, 2011) with increasing concentrations of tazarotene and bexarotene at an extended standard treatment time of 6 days. Tazarotene treatment led to a dose-dependent, significant increase in ARSA activity at concentrations as low as 5 μM (EC50 10.1 μM, Figs 2A and EV2A). Applying the same experimental conditions, bexarotene increased ARSA activity slightly (EC 50 3.4 μM, Figs 2B and EV2B). However, again, the combination of both drugs at a fixed combination 1:2 of tazarotene:bexarotene led to a dose-dependent increase in ARSA activity with significant differences against DMSO-treated controls as low as 2.5/5 μM tazarotene/bexarotene (EC 50 5.3/10.6 μM, Figs 2C and EV2C). To assess time dependency, we extended treatment times at a concentration of 10 μM tazarotene and 20 μM

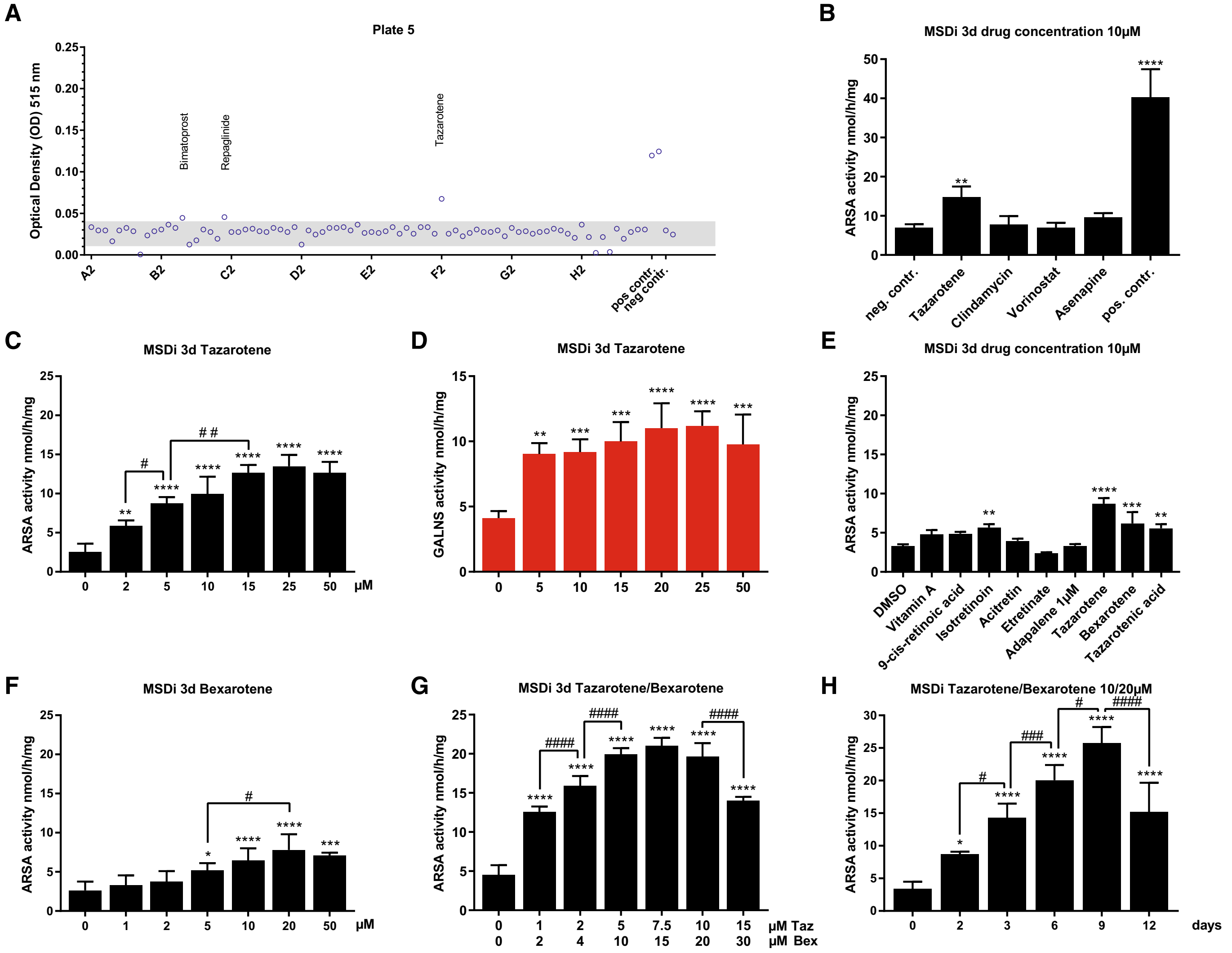

Figure 1.

Figure 1. Drug screen and evaluation of positive hit drugs using immortalized MSD patient-derived cells.

A Indicative plot of one 96-well screening plate of the MSD-ARSA high-throughput screening assay with negative and positive controls included. Individual OD values were given for each well (circles) and indicate ARSA activity (gray area: baseline activity). Three hit drugs exceeded the upper baseline OD range. $N = 1$ experiment per well and drug treatment, final concentration of each drug 10 μM, treatment time 48 h.

B ARSA activity quantification (nmol/h/mg) after treatment of MSDi cells with a selection of four positive hit drugs at a final concentration of 10 μM on MSDi cells in 25 cm^2 cell culture flasks for 3 days. Data represent mean ± SD of seven independent experiments (biological replicates). One-way ANOVA followed by the Tukey's test for multiple comparisons. Difference against negative control: $^{**}P < 0.01$, $^{****}P < 0.0001$. See details on *P*-values in Appendix Table S3.

C ARSA activity quantification (nmol/h/mg) after treatment of MSDi cells with increasing concentrations of tazarotene for 3 days. Data represent mean ± SD of 3–9 independent experiments (biological replicates). One-way ANOVA followed by the Tukey's test for multiple comparisons. Displayed are significance levels for the next significant difference between adjacent concentrations. # $P < 0.05$, ## $P < 0.01$. Difference against 0 μM control: $^{**}P < 0.01$, $^{****}P < 0.0001$. See details on *P*-values in Appendix Table S4.

D GALNS activity quantification after treatment of MSDi cells with increasing concentrations of tazarotene for 3 days. Data represent mean ± SD of four independent experiments (biological replicates). One-way ANOVA followed by the Tukey's test for multiple comparisons. Difference against 0 μM control: $^{**}P < 0.01$, $^{***}P < 0.001$, $^{****}P < 0.0001$. See details on *P*-values in Appendix Table S5.

E Analysis of different retinoids' potential to restore ARSA activity in MSDi cells in comparison to tazarotene after treatment for 3 days at a final concentration 10 μM of each drug (Adapalene 1 μM). Data represent mean ± SD of three independent experiments (biological replicates). One-way ANOVA followed by the Tukey's test for multiple comparisons. Difference against DMSO control: $^{**}P < 0.01$, $^{***}P < 0.001$, $^{****}P < 0.0001$. See details on *P*-values in Appendix Table S6.

F ARSA activity quantification after treatment of MSDi cells with increasing concentrations of bexarotene for 3 days. Data represent mean ± SD of 3–10 independent experiments (biological replicates). One-way ANOVA followed by the Tukey's test for multiple comparisons. Displayed are significance levels for the next significant difference between adjacent concentrations. # $P < 0.05$. Difference against 0 μM control: $^{*}P < 0.05$, $^{***}P < 0.001$, $^{****}P < 0.0001$. See details on *P*-values in Appendix Table S7.

G ARSA activity quantification after simultaneous treatment of MSDi cells with increasing concentrations of tazarotene and bexarotene in a fixed combination of 1:2 for 3 days. Data represent mean ± SD of 3–6 independent experiments (biological replicates). One-way ANOVA followed by the Tukey's test for multiple comparisons. Displayed are significance levels for the next significant difference between adjacent concentrations. #### $P < 0.0001$. Difference against 0/0 μM control: $^{****}P < 0.0001$. See details on *P*-values in Appendix Table S8.

H Analysis and quantification of a time-dependent increase in ARSA activity in MSDi cells simultaneously treated with 10 and 20 μM tazarotene and bexarotene, respectively. Data represent mean ± SD of 3–10 independent experiments (biological replicates). One-way ANOVA followed by the Tukey's test for multiple comparisons. Displayed are significance levels for the next significant difference between treatment times. # $P < 0.05$, ### $P < 0.001$, #### $P < 0.0001$. Difference against 0 days control: $^{*}P < 0.05$, $^{****}P < 0.0001$. See details on *P*-values in Appendix Table S9.

Source data are available online for this figure.

bexarotene up to 21 days. A maximum ARSA activity was reached after 9 days with no changes up to 21 days of treatment (ARSA activity 9 days: 175.8 nmol/h/mg (SD 25.9 nmol/h/mg), 10.9-fold increase, Fig 2D). This activity reached 21.6% of mean ARSA activity determined in five untreated non-LSD primary fibroblast lines (810.9 nmol/h/mg, SD 136.9 nmol/h/mg, Fig EV2D).

Next, we used lower concentrations of tazarotene and bexarotene ranging from 0.1/0.2 to 2.5/5 μM and extended the treatment time to 21 days to assess the lower range of working concentrations. We still detected a significant increase in ARSA activity at concentrations as low as 0.25/0.5 μM tazarotene/bexarotene (Fig EV2E). To test the drugs' effect on multiple sulfatases, we treated cells with 10 μM tazarotene and 20 μM bexarotene for 6 days and saw a significant increase in activities of lysosomal sulfatases arylsulfatase B (ARSB, 2.6-fold) and GALNS (3.3-fold), as well as the nonlysosomal sulfatase steryl sulfatase (STS, 7.7-fold, Fig 2E).

To explore whether the activity increase occurs with other *SUMF1* variants, we treated four different homozygous primary MSD patient fibroblast lines under standard conditions (10/20 μM tazarotene/bexarotene, 6 days). Baseline ARSA activities varied between 17.7 and 131.2 nmol/h/mg reaching 2 to 16% of control activities in non-MSD fibroblasts (Figs 2F and EV2D). All fibroblast lines showed a significant increase in ARSA activity between 2-fold (line 3, FGE Ala279Val) and 5.5-fold (line 1, FGE Gly247Arg; Fig 2F). The highest ARSA activity upon treatment was 324.5 nmol/h/mg, reaching 40% of control activities. Treatment of seven different homozygous primary MSD fibroblast lines with 10 μM tazarotene confirmed a mutation-independent response also to single treatment (ARSA activity increase 1.3–3.3-fold, maximum activity 282.4 nmol/h/mg, 35% of control activity, Fig EV2F).

As a side effect, a 6-day treatment with tazarotene, bexarotene, and tazarotene/bexarotene resulted in reduced cell proliferation when compared to cells treated with DMSO only. Cell culture conditions (cell density, cultivation time) did not influence endogenous ARSA activity in MSD fibroblasts until 9 days of cultivation (Appendix Supplementary Results, and Figs S5A–D and S6A–F). To determine whether programmed cell death contributed to the observed decrease in cell growth we quantified cleaved Poly(ADP-ribose) polymerase (PARP) expression levels, a marker of programmed cell death (Duriez & Shah, 1997). No significant differences in PARP levels between tazarotene/bexarotene and DMSO-treated MSD and control fibroblasts were detected (Fig EV2G).

To evaluate the efficacy of tazarotene and bexarotene on cell types different from fibroblasts, an iPSC line was generated from a MSD patient (compound heterozygous for *SUMF1* mutations c.463T > C, p.Ser155Pro and c.1034G > A, p.Arg345His, Appendix Fig S7A–F) Control and MSD patient-derived iPSC lines were differentiated into neuronal progenitor cells (NPCs) and treated with 5/5 μM tazarotene/bexarotene for 4 days. Baseline activities of ARSA and N-sulfoglucosamine sulfohydrolase (SGSH) in MSD NPCs were 11 (SD 0.49) and 0.22 (SD 0.03) nmol/h/mg, reaching 34 and 9.6% of control activities, respectively. Both activities significantly increased upon treatment (ARSA 17.6 nmol/h/mg, SD 0.5-, 1.6-fold; SGSH 0.46 nmol/h/mg, SD 0.01, 2.1-fold). Control iPSCs showed a slight increase in ARSA activity (1.2-fold) and slightly reduced SGSH activity (1.3-fold) upon treatment (Fig 2G and H).

Enlarged lysosomes are a hallmark of cellular pathophysiology in lysosomal disorders including MSD (Xu *et al*, 2014). To analyze the effect of tazarotene and bexarotene treatment on enlarged lysosomes we quantified lysosomal-associated membrane protein 1

 © 2023 The Authors

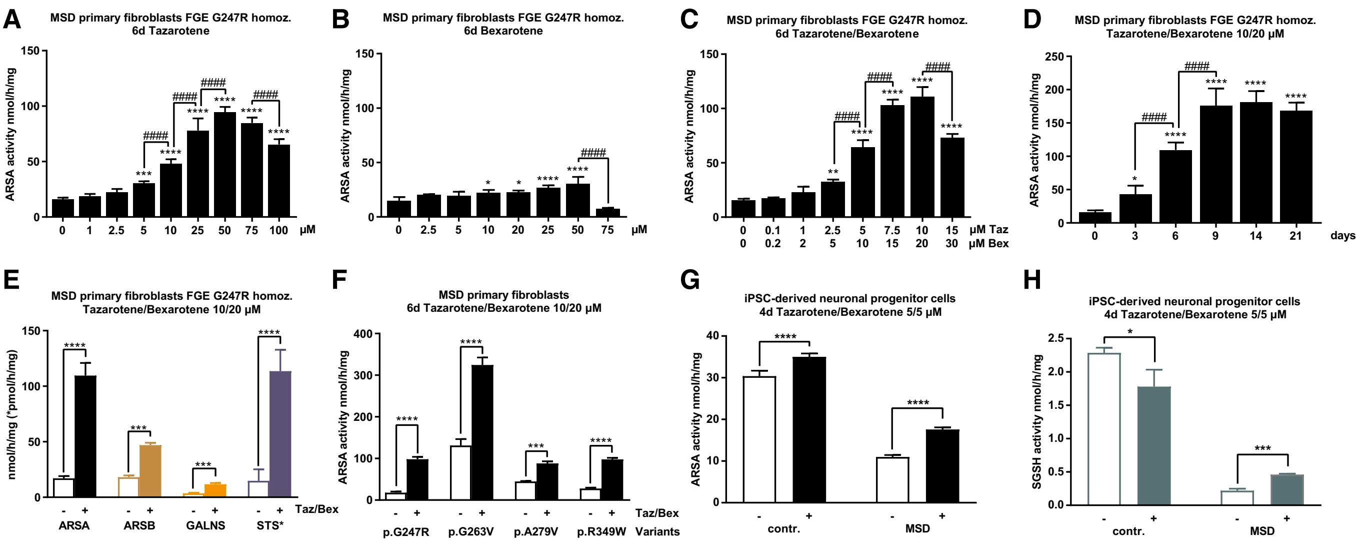

Figure 2. Tazarotene and bexarotene increase sulfatase activities in MSD primary fibroblasts and MSD iPSC-derived NPCs.

A ARSA activity quantification after treatment of MSD primary fibroblasts (variant FGE Gly247Arg homozygous) with increasing concentrations of tazarotene for 6 days. Data represent mean ± SD of 3–7 independent experiments (biological replicates). One-way ANOVA followed by the Tukey's test for multiple comparisons. Displayed are significance levels for the next significant difference between adjacent concentrations. #### $P < 0.0001$. Difference against 0 µM control: ***$P < 0.001$, ****$P < 0.0001$. See details on *P*-values in Appendix Table S10.

B ARSA activity quantification after treatment of MSD primary fibroblasts (variant FGE Gly247Arg homozygous) with increasing concentrations of bexarotene for 6 days. Data represent mean ± SD of 3–9 independent experiments (biological replicates). One-way ANOVA followed by the Tukey's test for multiple comparisons. Displayed are significance levels for the next significant difference between adjacent concentrations. #### $P < 0.0001$. Difference against 0 µM control: *$P < 0.05$, ****$P < 0.0001$. See details on *P*-values in Appendix Table S11.

C ARSA activity quantification after simultaneous treatment of MSD primary fibroblasts (variant FGE Gly247Arg homozygous) with increasing concentrations of tazarotene and bexarotene in a fixed combination of 1:2 for 6 days. Data represent mean ± SD of 3–6 independent experiments (biological replicates). One-way ANOVA followed by the Tukey's test for multiple comparisons. Displayed are significance levels for the next significant difference between adjacent concentrations. #### $P < 0.0001$. Difference against 0/0 µM control: **$P < 0.01$, ****$P < 0.0001$. See details on *P*-values in Appendix Table S12.

D Analysis and quantification of a time-dependent increase in ARSA activity in MSD primary fibroblasts (variant FGE Gly247Arg homozygous) simultaneously treated with 10 and 20 µM tazarotene and bexarotene, respectively, up to 21 days. Data represent mean ± SD of 3–6 independent experiments (biological replicates). One-way ANOVA followed by the Tukey's test for multiple comparisons. Displayed are significance levels for the next significant difference between adjacent treatment times. #### $P < 0.0001$. Difference against 0 days control: *$P < 0.05$, ****$P < 0.0001$. See details on *P*-values in Appendix Table S13.

E Analysis and quantification of increased sulfatase activities different to ARSA, namely ARSB, GALNS, and STS in MSD primary fibroblasts (variant FGE Gly247Arg homozygous) after 6 days of simultaneous treatment with tazarotene/bexarotene 10/20 µM. Data represent mean ± SD of 3–6 independent experiments (biological replicates). One-way ANOVA followed by the Tukey's test for multiple comparisons. ***$P < 0.001$, ****$P < 0.0001$. See details on *P*-values in Appendix Table S14.

F Quantification of ARSA activities in MSD primary fibroblasts with different homozygous *SUMF1* mutations (FGE Gly247Arg, FGE Gly263Val, FGE Ala279Val, FGE Arg349Trp) after 6 days of simultaneous treatment with tazarotene/bexarotene 10/20 µM. Data represent mean ± SD of three independent experiments (biological replicates). One-way ANOVA followed by the Tukey's test for multiple comparisons. ****$P < 0.0001$. See details on *P*-values in Appendix Table S15.

G Quantification of ARSA activity in MSD patient-derived iPSCs differentiated into NPCs and unaffected control NPCs controls. Simultaneous treatment with 5 µM tazarotene and 5 µM bexarotene for 4 days. Data represent mean ± SD of six independent experiments (biological replicates). Unpaired *t*-test. ****$P < 0.0001$. See details on *P*-values in Appendix Table S16.

H Quantification of SGSH activity in MSD patient-derived iPSCs differentiated into NPCs and unaffected control NPCs controls. Simultaneous treatment with 5 µM tazarotene and 5 µM bexarotene for 4 days. Data represent mean ± SD of three independent experiments (biological replicates). Unpaired *t*-test. *$P < 0.05$, ***$P < 0.001$. See details on *P*-values in Appendix Table S17.

Source data are available online for this figure.

(LAMP1) integrated fluorescence density in MSD and control fibroblasts (Fig 3A and B). Vehicle-treated MSD fibroblasts displayed increased LAMP1 integrated fluorescence density as compared to control fibroblasts. Upon treatment with tazarotene and bexarotene we observed a significant reduction in LAMP1 integrated fluorescence intensity compared with DMSO-treated MSD fibroblasts. Fluorescence intensity of tazarotene- and bexarotene-treated control fibroblasts was unchanged compared with DMSO-treated control fibroblasts (integrated fluorescence density control fibroblasts: DMSO 7.4×10^8, Taz/Bex 7.9×10^8; MSD fibroblasts: DMSO 3.7×10^9, Taz/Bex 1.9×10^9, Fig 3C). In addition, the size of lysosomes in MSD fibroblasts was increased compared with non-MSD fibroblasts under DMSO conditions. We observed a significant reduction in lysosomal size upon treatment with tazarotene and bexarotene as compared to untreated MSD fibroblasts. Again, we detected no differences in tazarotene and bexarotene versus DMSO-treated control fibroblasts (control fibroblasts: DMSO: 1.23 µm, ±0.1 µm, Taz/Bex: 1.14 µm, ±0.1 µm, MSD fibroblasts: DMSO: 6.9 µm, ±2.5 µm, Taz/Bex: 2.47 µm, ±1.2 µm; Fig 3D).

In MSD patients, glycosaminoglycans (GAGs) and sulfatides accumulate in tissues and organs (Guerra *et al*, 1990; Macaulay *et al*, 1998). Although we did not detect any sulfatides in MSD or

control fibroblasts, we detected five different GAG subspecies in control and MSD primary fibroblasts by adapting a mass spectrometry method for GAG detection for its use in lysates from fibroblasts (Fuller *et al*, 2004; see Materials and Methods for details). The amount of all five GAG subspecies was increased in one MSD primary fibroblasts line (MSD2, p.Gly247Arg) compared with unaffected control fibroblasts. The amount of four different GAG subspecies was elevated in two more MSD fibroblast lines compared with control fibroblasts (MSD1, p.Gly247Arg, MSD3 p.Ala279Val; Fig 3E and F, and Table EV1).

To evaluate whether tazarotene and bexarotene could reduce GAG accumulation, cells were treated for 21 days to allow sufficient time for the clearance of accumulated storage material. We detected a significant reduction in all glycosaminoglycan subspecies in three primary MSD fibroblast lines compared with DMSO treatment (Fig 3E and F, and Table EV1).

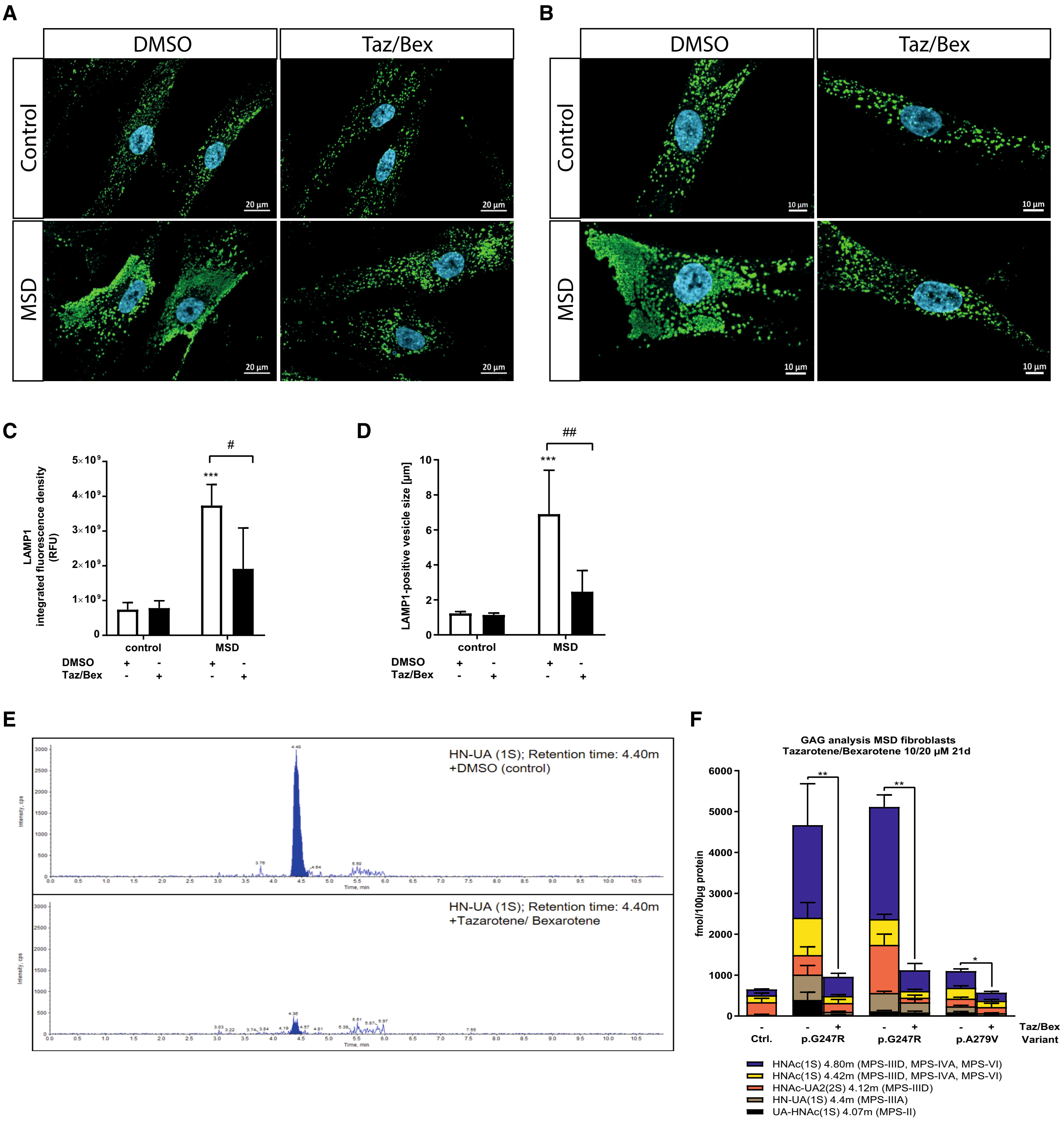

Figure 3.

 © 2023 The Authors

◀ **Figure 3. Tazarotene and bexarotene reverse lysosomal pathophysiology in MSD primary fibroblasts.**

A Representative confocal images of control and MSD fibroblasts with either tazarotene/bexarotene (10/20 μM, 6 days). Labelling with anti-LAMP1 antibody (green fluorescence) and DAPI (nuclei, blue). Scale bar = 20 μm.

B Representative confocal images of control and MSD fibroblasts with either tazarotene/bexarotene (10/20 μM, 6 days). Labelling with anti-LAMP1 antibody (green fluorescence) and DAPI (nuclei, blue). Scale bar = 10 μm.

C Quantification of the total intensity of LAMP1-green fluorescence. $N = 20$ images and 13 z-series optical sections per condition with a step size of 0.26 μm, displayed at maximum extension and automated equalization of brightness. Data represent mean ± SD of three independent experiments (biological replicates). One-way ANOVA followed by the Tukey's test for multiple comparisons. ***$P < 0.001$ (DMSO-treated MSD cells compared with DMSO-treated control cells), #$P < 0.05$ (MSD DMSO vs. MSD treated). RFU, relative fluorescence units. See details on P-values in Appendix Table S18.

D Quantification of LAMP1-green fluorescence vesicle size (μm). $N = 20$ images and 13 z-series optical sections per condition with a step size of 0.26 μm, displayed at maximum extension and automated equalization of brightness. Data represent mean ± SD of three independent experiments (biological replicates). One-way ANOVA followed by the Tukey's test for multiple comparisons. ***$P < 0.001$ (DMSO-treated MSD cells compared with DMSO-treated control cells), ## $P < 0.01$ (MSD DMSO vs. MSD treated). See details on P-values in Appendix Table S19.

E Representative spectra of the heparan sulfate-derived oligosaccharide HN-UA(1 S) as GAG marker analyzed via mass spectrometry in DMSO (control) treated MSD primary fibroblasts (variant FGE Gly247Arg homozygous, upper panel) and in the same cell line simultaneously treated with 10 and 20 μM tazarotene and bexarotene for 21 days (lower panel). Integrated peak areas correspond to the amount of HN-UA(1S).

F Quantification of specific oligosaccharide markers for GAG species in three different MSD primary fibroblast lines with FGE variants as indicated and one control fibroblast line after 21-day treatment with tazarotene/bexarotene. 10/20 μM. Data represent mean ± SD of 3–8 independent experiments (biological replicates). The unpaired t-test compares DMSO conditions of control and MSD fibroblasts and treated and DMSO control conditions in every MSD cell line for every marker. Details on significance levels for each marker are summarized in Table EV1. The lowest significance levels among individual markers for treatment and DMSO condition in MSD fibroblast lines are displayed. *$P < 0.05$, **$P < 0.01$.

Source data are available online for this figure.

Tazarotene and bexarotene work via retinoic acid receptors and induce gene expression in MSD patient cells

Retinoids bind to the retinoic acid receptors RAR and RXR, which, after homo- or heterodimerization, bind to DNA elements in promotors and initiate transcription (di Masi *et al*, 2015). To elucidate, which retinoic acid receptors are involved in the treatment response, MSDi cells were pretreated with AGN193109, a pan-RAR antagonist (Standeven *et al*, 1996), and HX531, a pan-RXR antagonist (Yotsumoto *et al*, 2005), followed by additional treatment with tazarotene and bexarotene alone or in combination, respectively. Incubation of MSDi cells with increasing concentrations of AGN193109 as single agent did not affect ARSA activity. Tazarotene-induced ARSA activity increase was abolished upon AGN193109 treatment. Bexarotene treatment showed no significant increase in ARSA activity in this experiment and no detectable changes with additional AGN193109 treatment. Tazarotene/bexarotene-induced ARSA activity increase was abolished upon AGN193109 treatment in a dose-dependent manner but still higher than tazarotene alone except for the highest AGN193109 concentration (Fig 4A). HX531 as single agent showed minimal agonistic function and increased ARSA activities at 20 μM concentration. Tazarotene-induced ARSA activity increase was abolished but only at the highest HX531 concentration (20 μM). Again, bexarotene-only treatment did not increase ARSA activity with no significant changes upon HX531 treatment. However, increasing HX531 doses

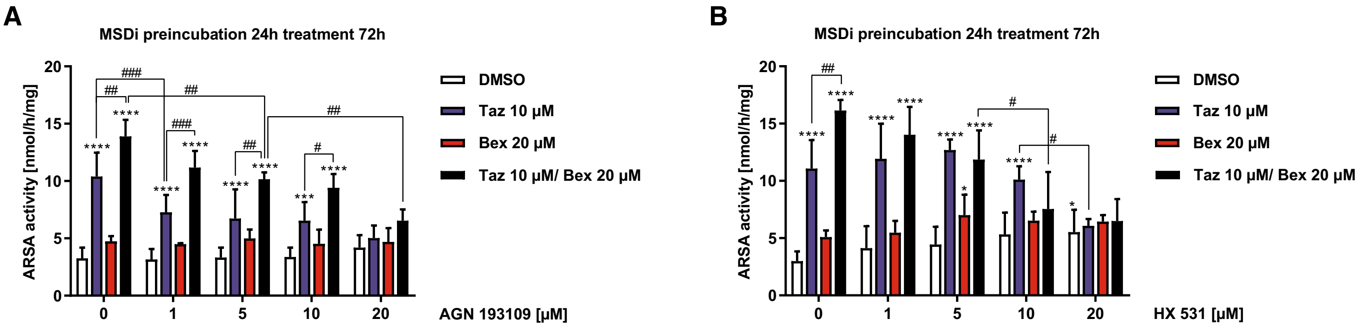

Figure 4. Tazarotene and bexarotene work through retinoid receptors in MSD fibroblasts.

A ARSA activity quantification upon treatment of MSDi cells with 10 μM tazarotene, 20 μM bexarotene, and 10 μM tazarotene and 20 μM bexarotene in combination with increasing concentrations of the pan-RAR receptor antagonist AGN 193109 (24 h pretreatment) for 72 h. Data represent mean ± SD of 3–11 independent experiments (biological replicates). Two-way ANOVA followed by the Tukey's test for multiple comparisons. Displayed are significance levels for the next significant difference between adjacent concentrations/conditions. # $P < 0.05$, ## $P < 0.01$, ### $P < 0.001$. Difference against 0 μM DMSO control: **$P < 0.01$, ***$P < 0.001$, ****$P < 0.0001$. See details on P-values in Appendix Table S20.

B ARSA activity quantification upon treatment of MSDi cells with 10 μM tazarotene, 20 μM bexarotene, and 10 μM tazarotene and 20 μM bexarotene in combination with increasing concentrations of the pan-RXR receptor antagonist HX 531 (24 h pretreatment) for 72 h. Data represent mean ± SD of 4–14 independent experiments (biological replicates). Two-way ANOVA followed by Tukey's test for multiple comparisons. Displayed are significance levels for the next significant difference between adjacent concentrations/conditions. # $P < 0.05$, ## $P < 0.01$. Difference against 0 μM DMSO control: ****$P < 0.0001$. See details on P-values in Appendix Table S21.

Source data are available online for this figure.

reduced the ARSA activity increase by tazarotene/bexarotene thereby abolishing the additional ARSA activity increase compared with tazarotene-only treatment (Fig 4B). In summary, blocking of RAR receptors inhibited ARSA activity increase through tazarotene/bexarotene and tazarotene, RXR receptor blocking predominantly inhibited tazarotene/bexarotene response. No significant differences were detected with the blocking of both receptors and treatment with bexarotene only.

To explore how tazarotene/bexarotene treatment affected gene expression in MSD cells we subjected treated and untreated MSD and control fibroblasts to RNAseq analysis and were able to analyze the expression of 16,385 genes. Selected genes, well-known to react to retinoic acid treatment (Napoli, 2017), showed increased transcription upon tazarotene/bexarotene treatment thereby indicating a successful treatment response. RNA expression of *RARRES1*, *CYP26B1*, and *RARB* was concordantly significantly increased for both MSD and control fibroblasts upon treatment compared with untreated controls. RNA expression of *RARRES 2* and *3* showed a concordant trend towards increased expression. RNA expression of other RAR and RXR receptors was unchanged except for *RARA* that was only increased in treated control fibroblasts and *RXRA* that was decreased in treated MSD fibroblasts. No expression of *RXRG* could be detected in either condition (Fig EV3A).

We assumed that increased transcription of sulfatases genes, known interacting partners of FGE, or *SUMF1*, the only known activating factor for sulfatases, could be an underlying cause for the sulfatase activity increase upon tazarotene and bexarotene treatment in MSD cells. However, RNA-expression analysis revealed significantly decreased *SUMF1* transcription in MSD fibroblasts upon treatment and no changes in transcription levels of detectable genes for FGE-interacting partners *SUMF2*, *P4HB*, *ERP44*, and *FURIN* (Fig EV3B). From 17 encoded sulfatases in the human genome, all transcripts except four (*ARSE*, *ARSF*, *ARSH*, *ARSK*) could be detected. Transcription of those sulfatases that showed increased catalytic activity upon tazarotene/bexarotene treatment was unchanged (*ARSA*, *ARSB*, *GALNS*), while *STS* transcription was significantly reduced. Among other sulfatases, *ARSI* and *SULF2* showed a trend towards increased transcription, and *SULF1* was the only sulfatase with increased transcription in MSD fibroblasts upon treatment (Fig EV3C).

To gain further insights into differential gene expression, we performed a weighted correlation network (WGCN) co-expression analysis from the RNAseq data and identified 16 co-expression modules. Using the Eigen-expression values of these modules for comparison, we detected four clusters that exhibited significant differences among groups, namely the yellow, brown, red, and pink modules. Whereas the red and pink clusters showed concordant expression in untreated control and MSD cells followed by concordant deregulation upon treatment (Fig EV4A and C), the yellow and brown clusters, most interestingly, showed significant differences in the deregulation of genes between MSD and control fibroblasts: The yellow cluster was downregulated when comparing DMSO-treated MSD patient cells (disease condition) to DMSO-treated control fibroblasts (normal condition) and this difference was ameliorated after tazarotene/bexarotene treatment (Fig 5A). These data suggest that the tazarotene/bexarotene treatment helps to reinstate physiological gene expression. GO-term and pathway analysis showed that genes of the yellow cluster represent the nuclear and mitochondrial compartment and pathways linked to metabolic processes and HIF-1 signaling (Fig 5B). The genes of the brown cluster were significantly downregulated in tazarotene/bexarotene-treated patient cells only (Fig 5C) and also represent mitochondria, while the functional pathways are mainly linked to pathological conditions (Fig 5D). Genes in the red cluster were significantly downregulated upon treatment in control and patient cells and were linked to organelle transport and intracellular signaling pathways (Fig EV4A and B). Genes in the pink cluster showed a significantly increased expression in treated control fibroblasts and MSD fibroblasts. Pathway analysis revealed intracellular signaling pathways, too, in addition to pathologies resulting from infectious diseases and genes representing intra- and extracellular vesicles (Fig EV4C and D).

In an attempt to further identify genes and pathways that are mediating sulfatase activity restoration upon treatment, we compared the transcriptional response of tazarotene ("positive" for sulfatase activity restoration) and the response of a retinoid that is incapable ("negative") of restoring sulfatase activities in MSD cells (Fig 1E). Through a set of preparatory experiments, we chose adapalene to serve as a control retinoid that provokes expression of retinoid targets genes in MSD cells without increasing sulfatase activities (please see Appendix Supplementary Results for details, and Figs S8A and B, S9A–D, and S10). We treated seven MSD fibroblast lines with either adapalene, tazarotene, or DMSO (control) in triplicates for 6 days and referred all samples to total RNA sequencing and differential gene expression analysis (Appendix Fig S11A). We identified the expression of 10,992 genes. For quality control, we analyzed a subset of retinoid target genes, which were significantly upregulated (*RARB, CYP26B1, RARRES1*) for both tazarotene and adapalene treatment compared with DMSO conditions, indicating a positive treatment response (Appendix Fig S11B). Analyzing all cell lines we found 1,042 genes differentially regulated when we compared gene expression between tazarotene and adapalene treatment (Appendix Fig S11C). GO biological process pathway analysis of the significantly differentially expressed genes by tazarotene (positive retinoid) revealed sterol synthesis and cholesterol synthesis pathways (Appendix Fig S11D) whereas pathways regulated by adapalene were mostly developmental pathways (Appendix Fig S11D). When comparing tazarotene- and adapalene-induced gene expression against DMSO conditions (untreated), respectively, 2,268 genes were regulated upon tazarotene treatment and 2004 upon adapalene treatment compared with DMSO conditions. 1,620 genes were identically regulated by both tazarotene or adapalene treatment, whereas 684 genes were exclusively regulated upon tazarotene treatment and 384 genes exclusively upon adapalene treatment (Fig EV4E and Appendix Fig S11F and G). GO biological pathway analysis of genes exclusively regulated by adapalene treatment identified phosphatidylinositol-mediated signaling pathways (Appendix Fig S11G). We focused on genes exclusively regulated upon tazarotene treatment because of its positive action on sulfatase activity restoration and identified 313 upregulated and 335 downregulated genes. GO biological process pathway analysis in the group of genes exclusively regulated by tazarotene treatment identified again sterol and cholesterol biosynthesis (Fig EV4E). Upregulated individual genes in these pathways, among others, were *SREBPF1*, *SREBF2*, and *INSIG1* (Appendix Fig S11H) coding for sterol regulatory element-binding proteins 1 and 2 (SREBP1, SREBP2) and insulin-induced gene proteins (INSIG). Together with the SREBP cleavage-activating

 © 2023 The Authors

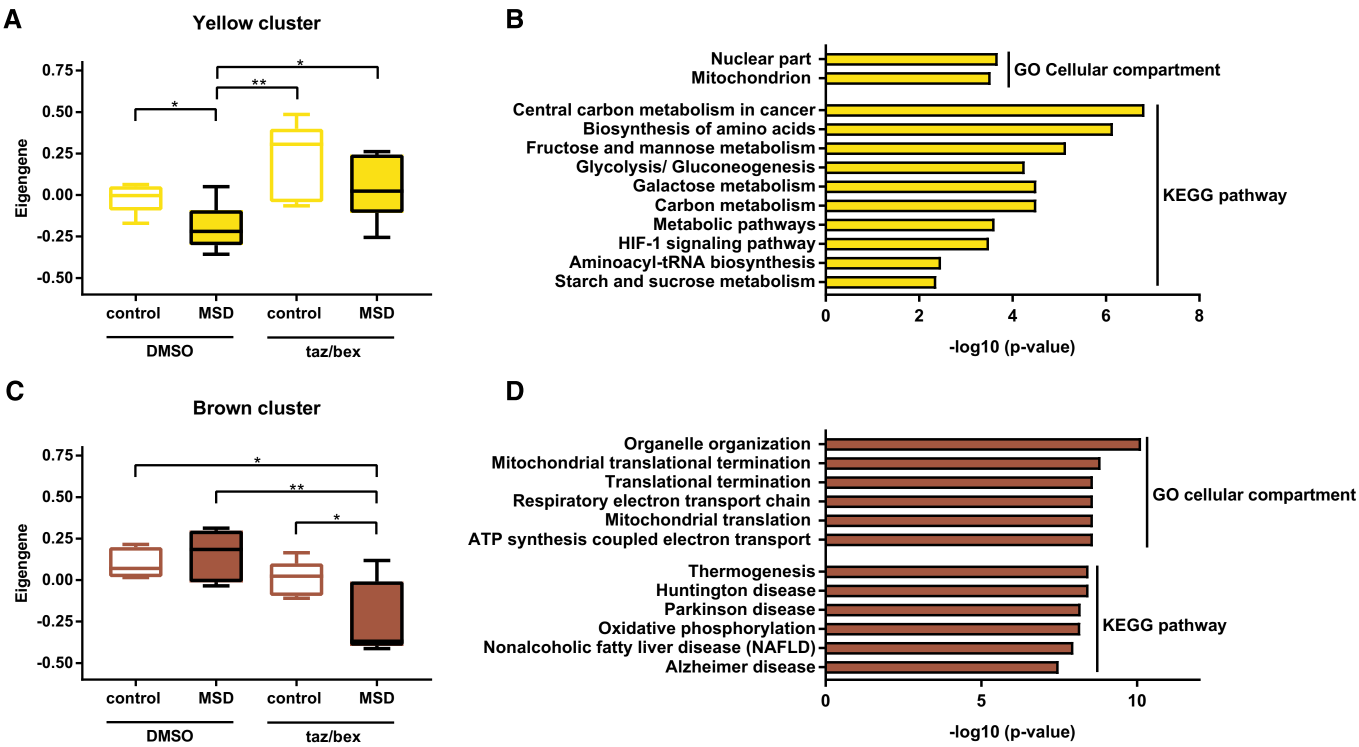

Figure 5. Transcription response of tazarotene and bexarotene treatment in MSD fibroblasts.

A Eigengene analysis of the yellow gene cluster as determined by WGCN analysis after RNA sequencing of six different MSD primary fibroblast lines and five different control fibroblast lines and treatment with tazarotene/bexarotene 10/20 µM or DMSO only, respectively, for 6 days. Data represent min to max box and whisker blots of Eigengene values ± SD of three independent experiments (biological replicates). One-way ANOVA test followed by Tukey's test for multiple comparisons. *$P < 0.05$, **$P < 0.01$. See details on *P*-values in Appendix Table S22.

B GO and KEGG pathway analysis of genes in the yellow cluster and log10 value of *P*-values.

C Eigengene analysis of the yellow gene cluster as determined by WGCN analysis after RNA sequencing of six different MSD primary fibroblast lines and five different control fibroblast lines and treatment with tazarotene/bexarotene 10/20 µM or DMSO only, respectively, for 6 days. Data represent min to max box and whisker blots of Eigengene values ± SD of three independent experiments (biological replicates). One-way ANOVA test followed by Tukey's test for multiple comparisons. *$P < 0.05$, **$P < 0.01$. See details on *P*-values in Appendix Table S23.

D GO and KEGG pathway analysis of genes in the brown cluster and log10 value of *P*-values.

Source data are available online for this figure.

protein (SCAP), these proteins are part of the SREBP-SCAP-INSIG complex in the ER sensing and controlling ER and cellular cholesterol content (Brown *et al*, 2018; Yang *et al*, 2002). In order to reveal any functional involvement of the complex in tazarotene-mediated sulfatase activity restoration we treated immortalized MSD cells with tazarotene and fatostatin for 3 days. Fatostatin suppresses SCAP/SREBP translocation (Cheng *et al*, 2018). Whereas treatment with increasing concentrations of fatostatin did not increase ARSA activity, tazarotene did. Simultaneous treatment with tazarotene (10 µM) and increasing fatostatin concentrations caused a dose-dependent ARSA activity decrease (Appendix Fig S11I).

Tazarotene and bexarotene require residual FGE function and increase the half-life of MSD-causing FGE variants

To further elucidate how tazarotene/bexarotene increase sulfatase activities and improve lysosomal pathology in MSD cells despite no changes in the transcription of respective genes, we analyzed whether treatment led to an increase in sulfatase protein levels. ARSA and GALNS protein levels were unaltered upon treatment of MSD fibroblasts with tazarotene/bexarotene using standard concentrations and treatment times (Figs 6A and EV5A, lower panels). However, when we analyzed specific ARSA activity by normalizing sulfatase activity to the ARSA protein amount, specific ARSA activity increased significantly in MSD fibroblasts after 6 days of tazarotene and tazarotene/bexarotene treatment (Fig 6A upper panel). Specific GALNS activity showed a trend towards increased activity (Fig EV5A upper panel). The increase in sulfatase activities without changes in protein expression levels suggests that tazarotene and bexarotene positively influence the activation process of sulfatases and act to boost FGE activity, the only known enzyme to activate cellular sulfatases (Cosma *et al*, 2003). To explore whether FGE mediates the response to tazarotene and bexarotene in MSD cells, we used ARPE19 retinal pigment epithelial cells with CRISPR/Cas9 generated *SUMF1*-gene knock-out (ARPE19 SUMF1$^{-/-}$, Appendix Fig S12) and appropriate controls (ARPE19 wt, MSDi) and treated with increasing concentrations of tazarotene. Remarkably, no increase in ARSA activity was observed when *SUMF1* knock-out

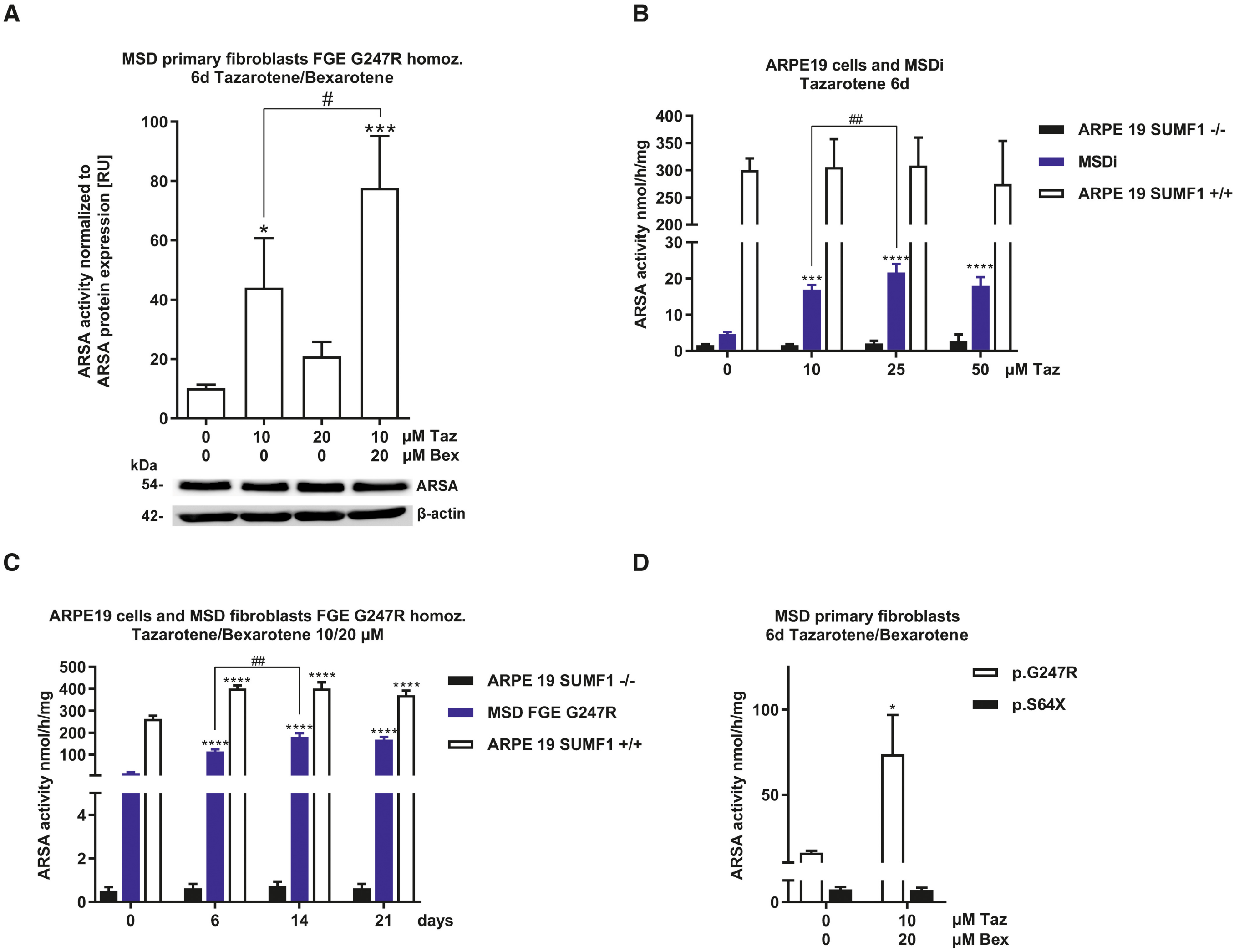

Figure 6. Tazarotene and bexarotene treatment response in MSD fibroblasts requires residual FGE function.

A ARSA protein amount quantification after treatment of MSD primary fibroblasts (variant FGE Gly247Arg homozygous) with tazarotene, bexarotene, and tazarotene/bexarotene in combination for 6 days referred to β-actin amounts and normalization of ARSA activity based on ARSA protein amount (specific ARSA activity). Data represent mean ± SD of three independent experiments (biological replicates). One-way ANOVA followed by Tukey's test for multiple comparisons. Displayed are significance levels for the next significant difference between adjacent concentrations. # $P < 0.05$. Difference against 0/0 µM control: * $P < 0.05$, *** $P < 0.001$. See details on P-values in Appendix Table S24.

B Quantification of ARSA activities in CRISPR/Cas9 generated ARPE19 SUMF1 −/− cells and appropriate controls (ARPE19 wild-type, MSDi) after 6 days of simultaneous treatment with increasing concentration of tazarotene. Data represent mean ± SD of three independent experiments (biological replicates). One-way ANOVA followed by Tukey's test for multiple comparisons. Displayed are significance levels for the next significant difference between adjacent concentrations. ## $P < 0.01$. Difference against 0 µM control: ***$P < 0.001$, ****$P < 0.0001$. See details on P-values in Appendix Table S25.

C Quantification of ARSA activities in CRISPR/Cas9 generated ARPE19 SUMF1 −/− cells and appropriate controls (ARPE19 wild-type, MSD primary fibroblasts (variant FGE Gly247Arg homozygous)) after 6 days of simultaneous treatment with tazarotene/bexarotene 10/20 µM for up to 21 days. Data represent mean ± SD of three independent experiments (biological replicates). One-way ANOVA followed by Tukey's test for multiple comparisons. Displayed are significance levels for the next significant difference between adjacent concentrations. ## $P < 0.01$. Difference against 0 days control: ****$P < 0.0001$. See details on P-values in Appendix Table S26.

D ARSA activity quantification after simultaneous treatment of MSD primary fibroblasts (variants FGE Gly247Arg homozygous, FGE Ser64Ter homozygous) with tazarotene and bexarotene. Treatment time 6 days. Data represent mean ± SD of three independent experiments (biological replicates). One-way ANOVA followed by Tukey's test for multiple comparisons. *$P < 0.05$. See details on P-values in Appendix Table S27.

Source data are available online for this figure.

cells were treated with tazarotene (Fig 6B). Treatment with tazarotene and bexarotene at standard concentrations up to 21 days also failed to increase ARSA activity in ARPE19 *SUMF1*$^{-/-}$ cells (Fig 6C). In addition, treatment of a previously described primary MSD patient-derived fibroblast line with a homozygous stop mutation and no FGE expression (FGE p.Ser64Ter; Schlotawa *et al*, 2019) also did not lead to any increase in ARSA activity (Fig 6D).

The majority of MSD cases are caused by hypomorphic *SUMF1* mutations resulting in instability and early degradation of FGE variants (Schlotawa *et al*, 2011). Increased intracellular half-life of

 © 2023 The Authors

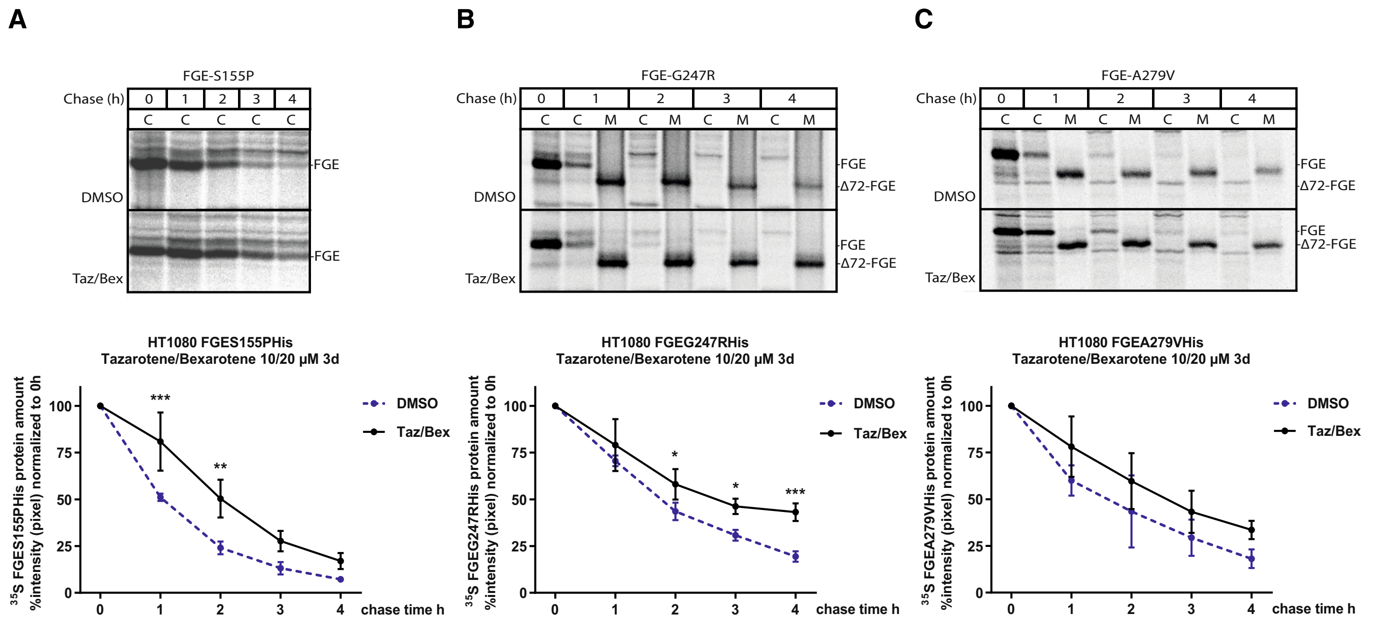

Figure 7. Improved FGE variant protein stability after tazarotene and bexarotene treatment.

A Pulse-chase-experiment in HT1080 FGE Ser155Pro cells after pretreatment with tazarotene/bexarotene and DMSO (control) for 3 days. Upper panel: representative autoradiogram of intracellular (C) ^{35}S isotope labeled FGE protein in either condition with a chase time of 4 h. Lower panel: quantification of ^{35}S isotope labeled intracellular FGE protein amounts. Data represent mean ± SD of three independent experiments (biological replicates). One-way ANOVA followed by Tukey's test for multiple comparisons. **$P < 0.01$, ***$P < 0.001$. See details on P-values in Appendix Table S28.

B Pulse-chase-experiment in HT 1080 FGE Gly247Arg cells after pretreatment with tazarotene/bexarotene and DMSO (control) for 3 days. Upper panel: representative autoradiogram of intracellular (full-length FGE, C) and cleaved and secreted (Δ72 FGE, M) ^{35}S isotope labeled FGE protein in either condition with a chase time of 4 h. Lower panel: quantification of ^{35}S isotope labeled intracellular and secreted FGE protein amounts. Data represent mean ± SD of three independent experiments (biological replicates). One-way ANOVA followed by Tukey's test for multiple comparisons. *$P < 0.05$, ***$P < 0.001$. See details on P-values in Appendix Table S29.

C Pulse-chase-experiment in HT 1080 FGE Ala279Val cells after pretreatment with tazarotene/bexarotene and DMSO (control) for 3 days. Upper panel: representative autoradiogram of intracellular (full-length FGE, C) and cleaved and secreted (Δ72 FGE, M) ^{35}S isotope labeled FGE protein in either condition with a chase time of 4 h. Lower panel: quantification of ^{35}S isotope labeled intracellular and secreted FGE protein amounts. Data represent mean ± SD of three independent experiments (biological replicates). One-way ANOVA followed by Tukey's test for multiple comparisons. See details on P-values in Appendix Table S30.

Source data are available online for this figure.

FGE variants has been shown to correlate with increased sulfatase activities. PDI is a pivotal interacting partner of FGE that preferentially binds misfolded FGE proteins, impairs their residual enzyme activity, and determines their fate by early degradation (Schlotawa *et al*, 2018). We analyzed the role of PDI in treated MSD cells. In addition to unchanged PDI transcription (*PH4B*, see above), we could not detect any differences in PDI protein expression upon tazarotene/bexarotene treatment (see Appendix Supplementary Results for details and Fig S13; Schlotawa *et al*, 2018). However, tazarotene/bexarotene treatment decreased a PDI-mediated inhibition of FGE variants' residual activity (Appendix Supplementary Results and Fig S14A–D).

Such an increase in FGE activity could be a result of less PDI interaction due to improved FGE variant protein stability. We finally assessed whether the FGE half-life changed upon tazarotene and bexarotene treatment performing a previously described pulse-chase experiment with HT1080 cells stably expressing FGE variants as a cell model (Schlotawa *et al*, 2011, 2018). Because wild-type FGE and variant FGE, depending on the type of mutation, is also secreted upon overexpression we assessed levels of intracellular and secreted FGE protein. FGE half-life was determined after 3 days of treatment with tazarotene/bexarotene 10/20 µM and DMSO controls. All cell lines except HT1080-FGESer155Pro secreted a truncated form of FGE and the protein half-life, calculated from intracellular and secreted FGE protein amounts, significantly increased for FGESer155Pro (2-fold, Fig 7A) and FGE Gly247Arg (1.5-fold, Fig 7B). The half-life of FGE Ala279Val showed a trend towards an increase (1.7-fold, Fig 7C). Half-life of FGE wild-type was unchanged upon treatment (Fig EV5B).

Discussion

MSD is a fatal and untreatable disease. To develop treatment approaches for MSD, we performed a screen of FDA-approved drugs and discovered the retinoic acid derivative tazarotene to partially restore ARSA activity in MSD patient cells. Subsequent testing revealed that tazarotene in combination with another retinoic acid derivative, bexarotene, partially but significantly restored different lysosomal and nonlysosomal sulfatase activities in MSD patient-derived fibroblasts, independent of the disease-causing *SUMF1* mutation. Treatment effects were both dose- and time-dependent. Only cell lines with homozygous null alleles and no FGE expression failed to respond to treatment suggesting an FGE-mediated

© 2023 The Authors

mechanism of drug response (see below). Drug treatment reduced pathologic accumulation of total GAGs, reduced lysosomal size, and normalized lysosomal positioning. The treatment effect was also observed in NPCs differentiated from MSD patient-derived iPSCs, which indicates a molecular mechanism and treatment response across multiple cell types. Taken together, we provide *in vitro* evidence for the first potential small molecule therapy that could translate into a promising strategy for MSD patients *in vivo* given sustained supportive data in subsequent analysis. Nevertheless, the identification of targets, mediating tazarotene and bexarotene response in MSD cells that are amenable to alternative small molecule treatment with more favorable unwanted effects, is desirable.

Molecular mechanisms of tazarotene and bexarotene in MSD

Tazarotene and bexarotene are both retinoids that bind to the retinoic acid receptors RAR and RXR, respectively. Ligands that bind to RXR receptors are also called rexinoids. For each class of receptor, three subtypes α, β, and γ with multiple isoforms exist. Upon ligand binding, the majority of RARs hetero-dimerize with RXRs, although the formation of homodimers has also been observed. In its canonical mode of action, RARs and RXRs recruit co-activating complexes or co-suppressors and bind to retinoic acid response elements (RARE) in DNA promotor regions thereby regulating transcription. Additionally, RAR/RXR heterodimers activate kinase cascades converging at the nucleus where they regulate transcription through the activation of co-activators and -repressors (di Masi *et al*, 2015).

Among all retinoids tested on MSDi cells, tazarotene was most effective in restoring sulfatase activities. Tazarotene is known to preferentially bind to RAR-β and -γ thereby initiating heterodimerization with RXR receptors. Bexarotene preferably binds to RXR receptors (Miller *et al*, 1997). In MSDi cells, bexarotene treatment response was relatively minor compared with tazarotene treatment and nearly absent in MSD primary fibroblasts, but, interestingly, the combination of tazarotene/bexarotene yielded the highest increase in sulfatase activities. Pretreatment of cells with the pan-RXR antagonist HX531 did not alter the sulfatase activity response to bexarotene treatment but reduced the tazarotene-mediated ARSA activity increase in a dose-dependent manner and the tazarotene/bexarotene response. However, pretreatment with a pan-RAR antagonist abrogated the response to tazarotene and tazarotene/bexarotene suggesting that RAR receptors are indispensable for mediating drug-induced sulfatase responses in MSD cells. Based on these results we speculate that sulfatase activation in MSD cells is mediated through RAR/RXR heterodimers. These observations are consistent with previous studies of other systems showing increased efficacy of combination retinoid/rexinoid therapy (Evans & Mangelsdorf, 2014; le Maire *et al*, 2019). However, specific drug-receptor relationships are complicated by the fact that RAR and RXR ligands could be less receptor-subtype specific at high concentrations, antagonists could exert agonistic functions, and receptor subtypes show redundancy (di Masi *et al*, 2015). Future research on the retinoic acid response in MSD will be critical, as well as worthwhile because the identification of receptors, and co-regulators could reveal additional or alternative potential downstream targets for therapeutic intervention.

Transcriptome data from this study and cellular compartment analysis of regulated genes identify mitochondria, vesicles and vesicular, and organelle trafficking. All compartments are closely linked to lysosomal function and pathology (Trivedi *et al*, 2020). In addition, genes known to be related to pathological conditions like Alzheimer's, Parkinson's, and Huntington's disease, indicative of severe cellular pathology, were downregulated in treated MSD fibroblasts (Lloyd-Evans & Haslett, 2016). Moreover, treatment with tazarotene/bexarotene normalizes the expression of deregulated genes in metabolic and signaling pathways in MSD fibroblasts with the reinstatement of a physiological expression pattern. In summary, the transcriptome analysis reveals improved cellular pathophysiology upon tazarotene/bexarotene treatment in MSD fibroblasts.

The interesting observation that the restoration of sulfatase activities in MSD cells was not general but restricted to a few retinoids was used to analyze differences between the transcriptional responses of a "positive" and a "negative" retinoid. We performed a set of preparatory experiments to identify adapalene as "negative" retinoid. We validated that the compound used is able to enter the cells, binds to RAR and RXR receptors and provokes a transcription response on established targets. However, we did not assess and compare properties like drug stability in cell culture medium, concentrations of drug metabolites in cells, or binding kinetics for the drugs and resulting in an impact on transcriptional response. Cell culture conditions, especially the presence of fetal calf serum and BSA in media, have been analyzed to beneficially influence the stability of retinoids from older pharmacological generations (Klaassen *et al*, 1999). Data for 3rd generation retinoids like tazarotene and bexarotene are missing. Although we cannot exclude that our experimental approach reveals nonphysiological effects, the preparatory measures for selecting the "negative" retinoid and an identical transcription response on known gene targets of retinoids, we propose that the clear differences in transcriptional response between tazarotene and adapalene treatment are physiological effects rather than artifacts. Focussing the transcriptome analysis on genes that are exclusively upregulated by tazarotene such an approach identified genes encoding members of the SREBP-SCAP-INSIG complex. The complex's intrinsic function is the transcriptional regulation of sterol and cholesterol synthesis and the sensing of lipid contents in ER membranes (Brown *et al*, 2018). This function, controlled by feedback mechanisms and cellular transcription pathways could be functionally linked to cholesterol storage in MSD cells (Eid *et al*, 2017). Cholesterol storage resulting from impaired digestion and redistribution of membrane contents is a known downstream effect in various lysosomal storage disorders (Platt *et al*, 2012). Details for tazarotene influencing the SREBP-SCAP-INSIG complex in the MSD cellular context could be manifold given the orchestrated self-regulation of the complex (McPherson & Gauthier, 2004) and will be the subject of future analysis. Treatment with fatostatin, an inhibitor of SREBP translocation, revealed blocking of the ARSA activity restoration by tazarotene (see Appendix Supplementary Results) though details are yet to be elucidated in MSD cells. Besides effects indirectly mediated by increased transcription, direct effects of retinoids on mitochondria, autophagy or intracellular signaling pathways have been described: RAR-β promotes mitochondrial membrane depolarization and transport in neurites through HIF1α (Trigo *et al*, 2019). RARRES1 (retinoic acid response element 1), a primary response protein of tazarotene, induces autophagy in cervical cells through TMEM192, a lysosomal

 © 2023 The Authors

membrane protein (Shyu *et al*, 2016). Bexarotene treatment showed an effect on autophagy and mitophagy in Presenilin 1 deficient iPSC-derived neural stem cells (Martin-Maestro *et al*, 2019). Furthermore, retinoic acids have been described to facilitate the mannose-6-phosphate receptor-dependent intracellular trafficking of lysosomal hydrolases (Kang *et al*, 1998). Direct retinoid effects apart from transcriptional control may therefore contribute to an improved cellular pathology in MSD cells.

Upregulated transcription of *SUMF1*, FGE-interacting proteins, and sulfatases initiated by tazarotene and bexarotene could have been an obvious explanation for downstream drug effects in MSD cells. However, the addition of tazarotene/bexarotene did not alter the transcription levels of these targets. Furthermore, we found no increase in sulfatase protein levels, suggesting that these drugs do not act by increasing the cellular half-life or intracellular retention of sulfatases. Despite no changes in sulfatase protein amounts, we did detect an increase in sulfatase activities. Such an effect requires FGE function, which is the only protein that is known to activate sulfatases (Cosma *et al*, 2003; Dierks *et al*, 2003). Our observations strongly suggest that tazarotene/bexarotene act to increase the activity of FGE. FGE misfolding due to *SUMF1* mutations affects its stability followed by accelerated degradation. Despite the residual catalytic activity of FGE variants, accelerated degradation results in impaired sulfatase activities (Schlotawa *et al*, 2013). PDI has been identified to play a pivotal role in mediating FGEs intracellular degradation. Decelerated degradation and prolonged half-life of misfolded FGE results in higher sulfatase activities (Schlotawa *et al*, 2018). Here, treatment with tazarotene and bexarotene increased the half-life of misfolded FGE, likely enhancing residual FGE activity that partially restores sulfatase activities in MSD cells. This hypothesis is further supported by the observation that tazarotene/bexarotene failed to increase sulfatase activation in cells that lack FGE. Tazarotene/bexarotene-induced increased FGE variant half-life could result from several factors including impaired protein degradation, improved protein folding, or both. Interestingly, based on our results, PDI seems not to be a direct target of tazarotene/bexarotene treatment. However, the effects on ER quality control mechanisms like the unfolded protein response or beneficial induction of ER stress (Mollereau *et al*, 2014) could be possible explanations. If existing functional links between ER stress and regulation of lipid metabolism involving the SREBP-SCAP-INISG complex (Moncan *et al*, 2021) connect FGE stabilization and regulation of sterol and cholesterol pathway regulation, both induced by tazarotene treatment in MSD cells, need to be elucidated by future experiments.

Because retinoids mediate a plethora of intracellular actions via a multitude of intracellular pathways, our discoveries, yet to be described mechanistically, are not comprehensive. (di Masi *et al*, 2015). Direct effects on FGE and additional indirect retinoid mechanisms would have likely contributed to the restoration of sulfatase activities in combination with the amelioration of cellular pathophysiology in MSD cells. We could demonstrate that cell quantity and cultivation time did not affect ARSA activity in MSD cells and treatment effects were reproducible across different MSD model cell lines and experimental conditions. However, any influence of *in vitro* conditions on cellular mechanisms cannot entirely be ruled out. Future studies, that aim to delineate the molecular mechanism (s) of retinoid treatment in MSD, will be necessary to identify direct targets for alternative therapeutic intervention.

Potential as a therapy for MSD patients

Tazarotene and bexarotene are both currently used in clinical applications. Bexarotene is approved for the treatment of cutaneous T-cell lymphoma, while tazarotene is used for topical skin treatment of psoriasis, acne, and photodamage (Duvic *et al*, 2001a; Talpur *et al*, 2009). Tazarotene has successfully passed phase III trials as an oral treatment for psoriasis but has not been approved for this application because of existing treatment alternatives and concerns about possible unwanted side effects intrinsic to the group of retinoids in general (Carlson, 2004).

Reported unwanted side effects include teratogenicity, liver toxicity, hyperlipidaemia, impairment of endocrine, visual and auditory function, and bone changes including mineralization, bone growth, hyperostosis, premature growth plate closure, and ligament calcification (David *et al*, 1988).

Nevertheless, the side effects in the completed phase III trials for oral tazarotene and bexarotene have been reported as mild (Duvic *et al*, 2001b; Weindl *et al*, 2006). None of the drugs has been tested in trials on children. *In vitro*, retinoids could affect cell growth, differentiation, and death (di Masi *et al*, 2015). Although we detected reduced cell proliferation of MSD cells at concentrations increasing sulfatase activities in our experiments, but we did not detect apoptosis rendering tazarotene and bexarotene treatment safe at least *in vitro*.

This study provides several lines of evidence that tazarotene and the combination tazarotene/bexarotene could be beneficial in MSD. Pharmacokinetics from phase I trial for tazarotene and bexarotene in adult probands with advanced cancer showed no toxicity at plasma concentrations that restored sulfatase activities *in vitro* (Miller *et al*, 1997; Jones *et al*, 2003). The active form of tazarotene in the systemic circulation, tazarotenic acid, also increased ARSA activity in MSDi cells. The observed time-dependent increase in sulfatase activities upon tazarotene/bexarotene treatment in MSD fibroblasts is promising because even sustained low concentrations of tazarotene/bexarotene could result in meaningful clinical improvement in MSD patients. As a notable example, slightly increasing enzyme stability has proven efficacious in another lysosomal storage disorder, Fabry disease. Specifically, a small molecule induced increase in residual activities of alpha-galactosidase A variants by 3% *in vitro* correlated with meaningful results in Fabry patients (Wu *et al*, 2011; Germain *et al*, 2012). Retinoic acid derivatives are able to cross the blood–brain barrier (Dos Santos Guilherme *et al*, 2019) and tazarotene and bexarotene increased activities of sulfatases in undifferentiated NPCs in a proof-of-principle experiment as described above. However, if both drugs would work on neurons and astrocytes *in vitro* and *in vivo* and would be able to reach the central nervous system, the organ system predominantly contributing to the clinical presentation of MSD patients (Ahrens-Nicklas *et al*, 2018), the evidence needed to be generated that drug concentrations in the CNS will be high enough to result in effective sulfatase activation. Data from healthy subjects treated with standard concentrations of bexarotene revealed a low penetrance of the drug into the CNS and concentrations of only approximately 20 nM (Ghosal *et al*, 2016).

Based on current knowledge more than 95% of MSD patients harbor at least one *SUMF1* missense allele and therefore may benefit from tazarotene/bexarotene treatment, which, according to our

© 2023 The Authors

data, could act by stabilization of hypomorphic FGE variants (Schlotawa *et al*, 2020). Interestingly, topical tazarotene treatment improved X-linked ichthyosis, caused by deficiency of steroid sulfatase, activated by FGE, and retinoid treatment increased STS activity via RARα and RXR receptors involving PI3 kinase and ERK-MAP kinase pathways in myeloid leukemia cells that have been discussed to evolve from FGE activity increase (Hofmann *et al*, 1999; Hughes *et al*, 2006). Although further experimental evidence supporting this hypothesis is missing, it may be possible that the intracellular mechanisms mediating the response to retinoic acid treatment in myeloid leukemia cells are similar to the action of tazarotene and bexarotene in MSD cells (Hughes *et al*, 2006).

While we aimed for identifying licensed drugs through drug screening that could be repurposed to treat MSD time- and effort-efficiently and identified tazarotene and bexarotene, the clinical application of these drugs requires a careful approach. *In vivo* studies should reveal the true potential of tazarotene and bexarotene treatment in MSD. Whereas toxicity data for both tazarotene and bexarotene have been generated as a prerequisite for the licensing of either drug the proof of principle that our *in vitro* data prove true in organisms are yet to be generated. The first step would be preclinical proof in suitable MSD animal models. A *SUMF1* knock-out mouse model and a *SUMF1* knock-out zebrafish model are not amenable to treatment as they both lack hypomorph FGE variants but are recently described new MSD mouse models with hypomorphic *SUMF1* variants (Settembre *et al*, 2007; Fleming *et al*, 2022; Sorrentino *et al*, 2022). Assuming a positive outcome of preclinical assessment, treatment of MSD patients with tazarotene and bexarotene and especially its combination would require all phases of clinical research involving MSD patients in childhood. In addition, a new formulation of either drug suitable for children and oral treatment would need to be tested. If either drug would be amenable to drug repurposing would mostly rely on further proof-of-principle results.

Conclusion and future perspectives

In conclusion, the data presented here might be a first step towards the development of a future therapy for MSD. Future *in vivo* studies of tazarotene and bexarotene are needed to evaluate systemic efficacy and overall adverse events or toxicity.

Moreover, this study reveals the hitherto unknown role and molecular mechanisms of retinoids in the pathophysiology of MSD and potentially other related LSDs. The identification of more mechanistic details unraveling alternative treatment targets should be the subject of further research.

Materials and Methods

Cell culture

Cell lines used were grown in cell culture as previously described and regularly checked to exclude mycoplasma contamination (Schlotawa *et al*, 2011, 2018). Andrea Ballabio kindly provided MSDi cells. Primary fibroblasts were grown from historical MSD patient samples collected for diagnostic purpose and approved for their use in research projects by the local IRB board (IRB board UMG Goettingen, amendments 3/9/17 and 33/2/21). Please see details on the generation of MSD patient-derived iPSCs and the origin of ARPE 19 cells further down.

Arylsulfatase A 96-well screening assay

We adapted Geng's protocol for high-throughput screening in MLD for its use in MSD (Geng *et al*, 2011). MSDi cells (*SUMF1* variant c.463C > T, p.Ser155Pro) were plated out in transparent 96-well plates (Sarstedt, Nürnbrecht, Germany) at a density of 5×10^4 cells in 200 µl cell culture medium per well. In two wells, MSDi cells stably expressing C-terminally 6-his tagged wild-type FGE (MSDi-FGEHis) were plated out at the same density and served as a positive control for rescued MSD cells (Schlotawa *et al*, 2018). After settling for 2 h 2 µl of each drug from the library (1 mM stocks in DMSO, see above) was added to the cell culture medium (200 µl) towards a final drug concentration of 10 µM and DMSO content of 1%. Controls were treated with DMSO only (see Appendix Fig S1A for the plate design). Cells were incubated for 48 h and then washed twice with Dulbecco PBS (Sigma-Aldrich Merck, Darmstadt Germany). After complete removal of PBS, 40 µl of lysis buffer (Cell lytic M, Sigma-Aldrich Merck, Darmstadt Germany + protease inhibitor Roche Complete easypack, Sigma-Aldrich Merck, Darmstadt Germany) was added, and plates were incubated for 2 h on ice allowing complete cell lysis. After cell lysis 40 µl of substrate buffer (10 mM p-nitrocatechol sulfate (pNCS, Sigma-Aldrich Merck, Darmstadt Germany), 0.5 M sodium-acetate pH 5.0, 0.5 mM sodium-pyrophosphate, 1.7 M sodium-chloride) were added to each well except wells A1-H1 and A12-D12, which were used for generating an optical density and extinction standard curve. Plates were shaken for 2 h at 300 rpm on an orbital shaker and incubated for 16 h at 37°C, 5% CO^2, and > 98% humidity. Finally, 120 µl of 1 N NaOH was supplied to each well to stop the enzymatic reaction. A pNC product (Sigma-Aldrich Merck, Darmstadt Germany) dilution series in substrate buffer (A1, B1: no pNC, C1, D1: pNC 20 µM, E1, F1: pNC 78 µM, G1, H1: pNC 156 µM, A12, B12: 313 µM, C12, D12: 625 µM) was added after supplying 120 µl of 1 N NaOH to prevent pNCS turnover by cell lysates. Plates were centrifuged at 1,160 *g* for 15 min. Supernatant (190 µl) from each well was transferred to a new 96-well plate without touching the bottom of the original 96-well plate to avoid suction of remaining cell debris. Air bubbles were manually removed, and the optical density and extinction were analyzed at 515 nm using a plate reader (Synergy Mx, BioTek, Winooski, USA). All pipetting was done using calibrated multi-channel pipettes (Eppendorf, Hamburg, Germany).

Drug treatment

LifeArc (London, UK) supplied a screening library in 96-well plates with 785 licensed drugs dissolved in 100% DMSO at a concentration of 1 mM of each drug (see Dataset EV1 for details on drug library). Hit drugs were identified from the primary screen, and additional drugs were purchased from commercial suppliers (purity ≥ 98%, Appendix Table S1) and used as supplied. All drugs were dissolved in DMSO at 100 or 10 mM stocks according to solubility and stored in aliquots at −80°C until usage. Working concentrations for cellular assays were generated by further dilution with DMSO. The maximum final amount of DMSO in the screening assay was 1%,

 © 2023 The Authors

while all secondary screens and mechanistic studies employed DMSO contents ≤ 0.1%. Drugs in DMSO were applied to the cell culture medium resulting in final concentrations as indicated between 1 and 100 μM. Media supplemented with DMSO alone served as a control treatment. Treatment times varied between a minimum of 24 h and a maximum of 21 days with the renewal of medium and drugs every 3 days and splitting and plating out when cells were confluent.

Western blotting

MSDi cells and MSD primary fibroblasts were incubated with 10 μM tazarotene, 20 μM bexarotene, or a combination of both for 3 or 6 days. Cells were collected and lysed in ice-cold lysis buffer containing protease inhibitor (50 mM Tris, 300 mM NaCl, 5 mM EDTA, 1% Triton X-100, 1% NP-40, 1% Protease inhibitor mix, Roche, Mannheim, Germany). After clearing by centrifugation (16,000 *g*, 5 min) at 4°C protein concentration was determined by BCA assay (Interchim, Montluçon, France). Total protein amounts between 10 and 40 μg were resolved by SDS–polyacrylamide gel electrophoresis (12% gel for FGE and 10% gel for ARSA, GALNS, and SGSH protein detection), then transferred onto a nitrocellulose membrane (GE Healthcare Life Science, Pittsburgh, USA) and blocked 1 h at room temperature in 5% nonfat milk in TBS-T (20 mM Tris, 150 mM NaCl, 0.1% Tween 20 (v/v), pH 7.6). Immunodetection was performed by incubation with primary antibodies against ARSA (polyclonal anti-rabbit, HPA005554, Sigma-Aldrich, St. Louis, USA; dilution 1:1,000), GALNS (polyclonal anti-rabbit, PA5-22098, ThermoFisher Scientific, Waltham, USA; dilution 1:1,000), PARP (monoclonal anti-mouse, sc-74,470, Santa Cruz Biotechnology, Dallas, USA; dilution 1:1,000), PARP-cleaved (monoclonal anti-rabbit, mAB #5625, Cell Signaling Technology, Danvers, USA; dilution 1:1,000), and beta-actin (monoclonal anti-rabbit, mAB #5625, Cell Signaling Technology, Danvers, USA; dilution 1:1,000) followed by incubation with species specific HRP-conjugated secondary antibodies (goat anti-rabbit, 111-035-003, Jackson Immunoresearch, West Grove, USA; dilution 1:5,000; goat anti-mouse, 115-035-146, Jackson Immunoresearch, West Grove, USA; dilution 1:5,000). Blots were visualized using the Lumi-Light chemiluminescence detection kit (Roche, Mannheim, Germany) and captured by the chemiluminescence detection system (GE FujiFilm LAS-4000 Luminescent Image Analyzer, GE Healthcare Life Science, Pittsburgh, USA).

Lysosomal enzyme activity assays

Arylsulfatase A, arylsulfatase B, galactose-6-sulfate sulfatase activity, and sulfamidase activity were determined following previously published protocols (Baum *et al*, 1959; Steckel *et al*, 1983; van Diggelen *et al*, 1990; Karpova *et al*, 1996). For arylsulfatase C activity analysis fibroblasts from a confluent 75 cm^2 flask were harvested after washing with PBS and lysed in 100 μl ice-cold NaCl 0.9% plus 0.1% (v/v) Triton X-100 and sonication (3 × 10 s). After centrifugation in a tabletop centrifuge at 1,000 *g* at 4°C the protein concentration of the supernatant was determined by BCA assay (see above). Supernatant (40 μg), diluted to a final volume of 50 μl in BSA/NaCl/TX buffer (0.2% BSA (m/v), 0.9% NaCl (m/v), 0.1% Triton X-100 (v/v)), was incubated with 50 μl of substrate buffer (100 mM NaH_2PO_4, 1 mM K4-MUF (7-hydroxy-4-methyl-coumarin)-sulfate (Sigma-Aldrich Merck, Darmstadt, Germany)) pH 8 at 37°C for 18 h in a black 96-well reaction plate (Greiner bio-one, Kremsmünster, Austria). Wells with 50 μl substrate buffer and 50 μl substrate buffer plus 3 mM dehydroepiandosteronsulfate and wells with substrate buffer and BSA/NaCl/TX solution without cell lysate served as negative controls. A product dilution series of 4-methylumbelliferone (4-MU; Sigma-Aldrich Merck, Darmstadt, Germany) served as standard. All reactions were stopped by adding 120 μl 0.5 M EDTA pH 11.2–12.0 per well. Readout was done using a fluorescent plate reader (Synergy Mx, BioTek, Winooski, USA) with excitation at 360 nm and emission at 460 nm. For β-hexosaminidase A + B activity, cells were prepared as described above. Cell lysates (2 μg) were diluted with substrate buffer (0.1 M citrate–phosphate pH 4.5, 2 mM 4-MU-2-acetoamido-2-deoxy-β-D-glucoside, Calbiochem Merck, Darmstadt, Germany) to 40 μl final volume in wells of a black 96-well plate. A standard product dilution series with 4-MU (Sigma-Aldrich Merck, Darmstadt, Germany) diluted in H_2O plus 0.05 M Tris–pH 8.0 was added. After incubation of 30 min at 37°C the reaction was stopped with 150 μl stop buffer (0.17 M glycine-carbonate), and plates were centrifuged for 15 min at 1,160 *g*. Readout was done using a fluorescent plate reader (Synergy Mx, BioTek, Winooski, USA) with excitation at 360 nm and emission at 460 nm. The same protocol was used for β-galactosidase activity with a different substrate buffer (0.1 M citrate–phosphate pH 4.5, 2 mM 4-MU-β-D-galactopyranoside). Activities were determined by referring to changes in OD or fluorescence, respectively, to total protein amounts. For the calculation of specific sulfatase activities, the sulfatase amount in cell lysates was determined in Western blots after quantification of the intensities of specific bands using ImageJ software. Activities were expressed as changes in OD or fluorescence divided by the amount of protein as determined by quantification of Western blots and incubation time.

Immunofluorescence

Fibroblasts were treated with tazarotene 10 μM and bexarotene 20 μM for 6 days. Cells were plated on cover slips on day 6 in a 24-well plate (Greiner bio-one, Kremsmünster, Austria) and allowed to attach for 24 h maintaining treatment conditions. Controls were treated with DMSO only. After washing with PBS, cells were fixed with 4% (v/v) PFA (Süsse, Gudensberg, Germany) in PBS for 20 min at 37°C, washed once again with PBS at 37°C and incubated for 10 min with 50 mM NH_4Cl. Next, cover slips were washed 3 × 5 min with PBS and incubated for 1 h with 10% horse serum (v/v, Gibco, Carlsbad, USA) and 0.2% saponin (m/v, Sigma-Aldrich Merck, Darmstadt, Germany) in PBS, followed by two washes with PBS/0.1% saponin (m/v). Cover slips were incubated for 1 h at room temperature with anti-LAMP1 mouse monoclonal antibody (BD Biosciences, San Jose, USA, 1:500 dilution in PBS/0.1% saponin (m/v)), washed 3× (PBS/0.1% saponin (m/v)), and incubated with Alexa Fluor 488 conjugated goat anti-mouse secondary antibody (MoBiTec, Göttingen, Germany; 1:1000 in PBS/0.1% saponin (m/v)) for 45 min. Cover slips were finally washed 3× (PBS/0.1% saponin) and 2× with PBS and mounted on slides with prolonged gold mountant +/− DAPI (Invitrogen, Carlsbad, USA). Fluorescence microscopy was performed using a Zeiss Definite Focus.2 confocal inverted microscope (Zeiss, Göttingen, Germany). Images were taken with a Plan-APOCHROMAT 63 × 1.4 numerical

© 2023 The Authors

aperture oil-immersion objective (Zeiss, Göttingen, Germany) using the ApoTome system. For each image, 13 z-series optical sections were collected with a step size of 0.26 μm. Z-series are displayed as maximum z-projections, and brightness and contrast were adjusted identically for each image set using ZEN Pro software (Zeiss, Göttingen, Germany). The total fluorescence intensity of the cells and the size of the LAMP1-positive particles were analyzed from a minimum of 20 cells for each treatment (n = 3 independent experiments (biological replicates)).

Glycosaminoglycan quantification

Glycosaminoglycan analysis was performed by adapting a protocol established by Fuller *et al* (2004). MSD primary fibroblasts and control fibroblasts were grown in T75 cell culture flasks (CellStar, Greiner bio-one, Kremsmünster, Austria) with tazarotene/bexarotene 10/20 μM or DMSO as the control for 21 days. Cells from confluent flasks were harvested, and protein concentration was measured by BCA assay after lysis of 1/5th of the cells. 4/5th of the cells were frozen at −20°C and stored until further processing.

After thawing, cell pellets were resuspended in 50 μl PBS per 120 μg total protein. Fifty microliter of each sample was dried using a centrifugal concentrator under vacuum and reconstituted in 100 μl of 0.25 M PMP solution (0.25 M 1-phenyl-3-methyl-5-pyrazolone (PMP)) in 0.4 M ammonia solution (11.95 ml of MeOH and 2.59 ml of ammonium hydroxide (28–30% ammonia) added to 35.5 ml MilliQ water (pH 9.5–10) containing 1 μM of internal standard (chondroitin disaccharide di-4 S [CAS 136144-56-4], Carbosynth Ref: OC28898)). Samples were vortexed, sonicated, and mixed prior to 90 min incubation on a PCR thermocycler at 70°C and cooling for 10 min. Samples were acidified with 500 μl of 0.2 M formic acid, and PMP was extracted from the acidified samples by adding 500 μl chloroform and shaking for 1 min. Samples were centrifuged for 5 min at 13,000 g to separate the layers and the bottom organic layer was discarded. The procedure was repeated four times for each sample to completely remove PMP. The remaining aqueous layer (600 μl for each sample) was concentrated to 80 μl using a centrifugal concentrator under vacuum. After centrifugation for an additional 5 min at 13,000 g the supernatant (at least 60 μl) of every sample was referred to LC–MS/MS analysis on an Agilent UPLC system (Agilent Pursuit 3 PFP 2.0 ×100 mm 3 μm Column (Agilent, Santa Clara, USA)) and AB Sciex 6500 TQ Mass Spec System (Sciex, Framingham, USA).

Cell proliferation analysis

MSDi cells and MSD primary fibroblasts were seeded at a concentration of 3,000 and 2,000 cells/well, respectively, in 96-well microplates and allowed to attach for 24 h. Cells were incubated with 10 μM tazarotene, 20 μM bexarotene, or a combination of both for 3 days (MSDi) or 6 days (fibroblasts). Cell proliferation during incubation was determined by XTT [sodium 3′-[1-(phenylaminocarbonyl)-3,4-tetrazolium]-bis (4-methoxy-6-nitro) benzene sulfonic acid hydrate] assay according to the manufacturer's protocol (AppliChem, Darmstadt, Germany). Absorbance was measured at 450 nm (reference wavelength 650 nm). Proliferation was expressed as a percentage of control cells treated with DMSO. Manual counts of cells were performed after trypsinization of cells, centrifugation, and resuspension of cell pellets in 5 ml PBS. Ten microliter of the suspension was pipetted onto a Neubauer counting chamber followed by manual cell counting using a light microscope.

iPSC generation

The generation of an iPSC line from a MSD patient was done following a previously published protocol (Maguire *et al*, 2016). In brief, blood was collected from a patient with MSD harboring compound heterozygote *SUMF1* variants (c.463T > C, p.Ser155Pro/ c.1034G > A, p.Arg345His). This research was approved by the Institutional Review Board at the Children's Hospital of Philadelphia (IRB #09-00742) Cellular reprogramming was performed using ficoll-purified mononuclear cells from whole blood that were expanded for transduction with Sendai viral vectors expressing human OCT3/4, SOX2, KLF4, and cMYC according to the manufacturer's instructions (ThermoFisher Scientific). Transduced cells were plated on culture dishes containing murine embryonic fibroblasts (MEFs) and maintained in a medium containing 10 ng/ml bFGF. The medium was replenished daily for 3 weeks. Cells were maintained in these conditions until uniform colonies were generated and colonies were mechanically isolated for expansion on MEFs. Single colony subcloning was performed at early passages and tested for clearance of the Sendai reprogramming vectors using real-time RT–PCR (Appendix Fig S7A). The authentication of each clone confirming identity to the original patient cells was performed by DNA fingerprinting using PCR (Appendix Fig S7B). Mutation verification was also performed on genomic DNA by PCR amplification and sequence analyses (Appendix Fig S7C). Karyotype analysis was performed by Cell Line Genetics (Madison, WI). Stemness surface markers were performed by flow cytometry and mycoplasma was tested by PCR (Appendix Fig S7D and E).

Differentiation of iPSCs into neural progenitor cells

Differentiation of iPSCs into NPCs was initiated, as previously described (Maguire *et al*, 2016) with indicated modification. Briefly, cultures were treated with daily media changes containing SB431542 (10 μM; Tocris), LDN193189 (1 μM, Tocris), and *endo*-IWR1 (1.5 μM; Tocris) and supplemented with B27 without vitamin A (Invitrogen) and passaged at days 4 and 8 of differentiation. From days 8 to 14 of differentiation, NPCs were expanded in Invitrogen neural expansion media, containing Neural Induction Supplement in Advanced DMEM/F12 and Neurobasal Medium (Invitrogen, per manufacturer instructions). On Day 14, NPCs were cryopreserved after confirmation of NPC identity with > 90% expression of Forse-1.

NPC drug treatment

For drug treatments, NPCs were retrieved into neural expansion media containing 5 μM Y-27632 (Tocris), followed by 4 days of daily media changes containing tazarotene (5 μM) and bexarotene (5 μM) or the equivalent concentration of DMSO (0.01%) in neural expansion media. NPCs were subsequently harvested using accutase, washed with PBS, pelleted, and frozen at −80°C until analysis. All conditions were performed in triplicate.

© 2023 The Authors

ARPE19 *SUMF1*$^{-/-}$ cell line generation

ARPE19 (ATCC, Manassas, USA, Cat. No. CRL-2302) cells were referred to CRISPR/Cas9-mediated knock-out of the *SUMF1* gene to generate the ARPE19 SUMF1$^{-/-}$ cell line. The gRNA sequence was determined by using the CRISPOR online tool (http://crispor.tefor.net/crispor.py; Concordet & Haeussler, 2018) and selected based on the lowest off-target score. The gRNA with the 5′-3′ sequence CCCTTGCGGGTTCTTGCGGCTGC was used in an "all-in-one" vector additionally encoding Cas9 linked to green-fluorescent protein (Cas9-GFP) (Sigma-Aldrich, St. Louis, USA). Plasmid-DNA was electroporated into ARPE19 cells using the Amaxa system and a nucleofection kit (Lonza, Basel, Switzerland, Cat. No. VCA-1003) following the manufacturers' instructions. GFP-positive cells were sorted by fluorescent-activated-cell-sorting (FACS) into 96-well plates. Single-cell derived colonies were screened for deletion mutations in the *SUMF1* gene after extraction of genomic DNA, amplification of the target region by PCR (forward primer hSUMF1KOup, 5′-3′-sequence: cagcgccaaagaagtacctg, reverse primer hSUMF1KOlow, 5′-3′-sequence: tcggaggaatcgatggagc), followed by Sanger sequencing using the same primers. A cell clone carrying a homozygous deletion in the *SUMF1* gene (c.139delCG, p.Ala47GlyfsTer74) leading to a premature stop codon was selected and expanded. Cells of the respective clone were subjected to cell lysis, protein estimation, and Western Blot analysis as described above using a SUMF1 antibody (R&D Systems, Minneapolis, USA, Cat No AF3680) to verify absent FGE protein expression (Appendix Fig S12).

cDNA synthesis and RT–PCR

Total RNA was isolated using NucleoSpin RNA preparation kit (Macherey-Nagel, Düren, Germany). After quality control by optical density (OD) measurement, 1 or 2 μg of RNA was reverse transcribed using SuperScript III First-strand Synthesis system for RT–PCR (Invitrogen, Karlsruhe, Germany) according to the manufacturer's instruction. Real-Time PCR analysis for *CYP26B1*, *RARB*, *RARRES1*, *RARRES2*, and *RARRES3* was performed using Quant Studio 3 Real-Time PCR System (ThermoFisher Scientific, Braunschweig, Germany) according to the manufacturer's recommendations in reactions containing 20 ng cDNA. The gene expression of targeted genes was normalized to the housekeeping gene as indicated in the figure legends. Primer sequences are given in Appendix Table S2. Relative gene expression was analyzed by QuantStudio Design and Analysis software v1.4.3 (ThermoFisher Scientific, Braunschweig, Germany) and quantified using the $\Delta\Delta C_t$ method.

Transcriptome and pathway analysis

For our first experiment (comparison of tazarotene/bexarotene treatment to untreated condition), six different MSD fibroblast lines and five control fibroblast lines were treated with DMSO or tazarotene/bexarotene 10/20 μM for 6 days in triplicates. Cells were harvested and processed for RNA isolation and RNA sequencing as described (Martinez Hernandez *et al*, 2018). cDNA libraries were established using a TrueSeq Stranded Total RNA library kit (#20020596, Illumina) and sequenced using a Illumina HiSeq 2000. For expression analysis, reads were mapped to the human genome (hg38) using STAR aligner (v.2.7.3a). Mapped reads were sorted and indexed with SAMtools (v.1.10) and gene counts were generated with Featurecounts (v.1.5.1). Low-quality samples were removed from the analyses. Sequencing data from the first experiment was analyzed using co-expression analysis, given our interest in finding clusters of genes that had similar correlation patterns. In specific, the so-called WGCNA (weighted-gene co-expression analysis) was performed with the homonymous R-package (v.1.68) and applying the following steps: (i) the analysis was done on normalized expression values obtained with DESeq2 (v.1.68). (ii) genes were filtered out from the analysis if they did not have, on average, at least 20 normalized counts per sample. (iii) the power parameter of the network topology was estimated with the function "pickSoftThreshold." Finally, gene clusters were inferred with the function "blockwiseModules" with parameters "maxBlockSiz=7000, power=14, minModuleSize=60, and mergeCutHeight=0.25." Gene ontology enrichment analysis on resulting clusters was performed with the ShinyGO online webtool (v.0.61) hosted by the University of South Dakota, US (http://bioinformatics.sdstate.edu/go/).

For our second experiment (comparison of tazarotene to adapalene treatment), seven different MSD fibroblast lines were treated with tazarotene 10 μM or adapalene 5 μM, respectively, for 6 days in triplicates. DMSO-treated cells served as a control condition. Sequencing data from the second experiment were analyzed using a differential gene expression (DGE) approach and for such purpose, the aforementioned DESeq2 package was used. Samples were quality controlled using PCA analysis on gene count data, with specific gene counts themselves filtered out if they had less than 30 reads on average across all samples. The resulting count matrix was normalized and used as input for DGE. The design function took into account that samples came from seven different cell lines in triplicates. Tazarotene and adapalene treatments were compared using the contrast function embedded within the DESeq2 package.

PDI expression and STS-specific activity assay

PDI expression in MSDi cells and inducible expression of STS and FGE for the determination of STS-specific activity by western blots and STS-activity assays were done as described before (Schlotawa *et al*, 2018) following tazarotene/bexarotene treatment for a total time of 3 days.

Pulse-Chase experiments

HT1080 cell lines stably expressing FGE variants Ser155Pro, Gly247Arg and Ala279Val were either treated with 10 μM tazarotene and 20 μM bexarotene or DMSO (as control) for 3 days prior to the start of pulse-chase experiments. Of note, the presence of the drug or DMSO was maintained in all supplemented media throughout the experiment. After starving for 1 h in a medium depleted of methionine and cysteine, the cells were pulsed with ^{35}S-methionine/cysteine (Hartmann Analytic) for 30 min. Cells and media were collected after incubation for various time points in unlabeled medium (chase). Cell lysis, FGE immunoprecipitation from cell lysate and media, SDS–PAGE and autoradiography, and image analysis using ImageJ software were as previously described (Schlotawa *et al*, 2018).

© 2023 The Authors

Image quantification

Western Blots and immunofluorescence images were not blinded quantitatively analyzed using Fiji software (Schindelin *et al*, 2012). Statistical analysis was performed using Prism (GraphPad software, San Diego, USA).

Statistical analysis

Results were calculated from a minimum of three independent experiments (biological replicates), and replicate measurements were summarized as mean values in respective calculations. Exemptions in the number of experiments are indicated in respective figure captions. All statistical analysis was performed using Prism (GraphPad software, San Diego, USA). Comparison of two independent experimental conditions was done using an unpaired *t*-test, and paired *t*-tests were used for dependent variables. Comparison of multiple experimental conditions (> 2) was executed by one-way ANOVA tests followed by the Tukey's multiple comparison test according to the program's settings. Two-way ANOVA followed by the Tukey's multiple comparison test was used when results depended on two parameters (treatment vs. untreated and time in pulse-chase experiments). For nonlinear regression calculation, drug concentrations were transformed to log10, manually referring activity responses to DMSO treatment as log −2 concentrations, and calculated using the program's predefined settings. Data were expressed and displayed as mean and standard deviation. Significance levels were displayed as follows: *$P < 0.05$, **$P > 0.01$, ***$P < 0.001$, ****$P < 0.0001$ or # $P < 0.05$, ## $P < 0.01$, ### $P < 0.001$, #### $P < 0.0001$, respectively, for differences indicated in the figure legends. Details on *P*-values of significant differences only for all figures are summarized in Appendix Tables S3–S46.

Data availability

RNAseq data are available via the GEO database (GEO accession GSE205555 and GSE205556, https://www.ncbi.nlm.nih.gov/geo/).

Expanded View for this article is available online.

Acknowledgements

We remember Thomas Dierks, who unexpectedly passed away during this research project, and thank him for his irreplaceable work on MSD. We thank LifeArc for the provision of the FDA-approved drug library and Roberto Zanchi for helping with the application process. We are grateful to Kathrin Schreiber and Tanja Wilke for excellent technical assistance in developing and running the screening assay, as well as other experiments. We appreciate Ralph Krätzner's input by fruitful discussions and carefully reading the manuscript. We thank MSD Action Foundation and United MSD Foundation and especially Alan Finglas and Amber Olsen for continuous support and implementation of MSD research projects. JG received funding from the Deutsche Forschungsgemeinschaft (German Research Foundation, DFG) grant AB 1234/1-1, GA 354/14-1, Transregional Collaborative Research Center (GRC) 274 "checkpoints of CNS recovery," and Germany's Excellence Strategy (EXC 2067/1, JG). MK received grants from the Göttingen College for Translational Medicine, Ministry of Science and Culture, Lower Saxony, Germany. MHG received a grant from the National Institutes of Health (R01 DK067859). Open Access funding enabled and organized by ProjektDEAL.

The paper explained

Problem
No curative therapy exists for Multiple Sulfatase Deficiency (MSD), an ultra-rare lysosomal disorder. MSD is caused by a defect of the post-translational activation of all cellular sulfatases through the formylglycine-generating enzyme (FGE) in the endoplasmic reticulum. FGE is encoded by the *SUMF1* gene and *SUMF1* mutations in MSD patients lead to impaired function of FGE and reduced or absent sulfatase activities in every cell. MSD patients present with a combination of signs and symptoms of single sulfatase deficiencies like developmental delay, neurodegeneration, skeletal abnormalities, and ichthyosis among others in a progressive, very severe disease.

Results
We developed a high-throughput screening assay using MSD patient-derived fibroblasts and investigated the rescue of the enzymatic function of one defective sulfatase, arylsulfatase A (ARSA), as primary readout. Applying the assay we screened a library of 785 licensed drugs and detected two retinoids, tazarotene and bexarotene to increase ARSA activity. In subsequent analysis, both drugs proved to be effective in reversing cellular pathology in MSD fibroblasts and neuronal progenitor cells. Both drugs work via the stabilization of misfolded FGE proteins to increase sulfatase activities in MSD cells.

Impact
Our study reveals a new mechanism of retinoids in MSD pathology. Furthermore, we identified the first described agents to correct MSD pathology *in vitro*. Our data lay the basis for future research on therapeutic approaches for MSD and the identification of targets that mediate retinoid treatment response.

Author contributions

Lars Schlotawa: Conceptualization; data curation; formal analysis; supervision; validation; investigation; visualization; methodology; writing – original draft; project administration; writing – review and editing. **Karolina Tyka:** Conceptualization; data curation; formal analysis; supervision; validation; investigation; visualization; methodology; writing – review and editing. **Matthias Kettwig:** Conceptualization; data curation; formal analysis; supervision; validation; investigation; visualization; methodology; writing – review and editing. **Rebecca C Ahrens-Nicklas:** Investigation. **Matthias Baud:** Conceptualization; validation; writing – review and editing. **Tea Berulava:** Data curation; formal analysis; validation; investigation; visualization; methodology; writing – original draft. **Nicola Brunetti-Pierri:** Validation; investigation; writing – review and editing. **Alyssa Gagne:** Validation; methodology; writing – review and editing. **Zackary M Herbst:** Data curation; formal analysis; validation; investigation; methodology; writing – original draft; writing – review and editing. **Jean A Maguire:** Data curation; formal analysis; validation; investigation; methodology; writing – review and editing. **Jlenia Monfregola:** Resources; validation; methodology; writing – original draft; writing – review and editing. **Tonatiuh Pena:** Data curation; formal analysis; validation; investigation; visualization; methodology; writing – original draft; writing – review and editing. **Karthikeyan Radhakrishnan:** Conceptualization; data curation; formal analysis; supervision; validation; investigation; visualization; methodology; writing – review and editing. **Sophie Schröder:** Data curation; formal analysis; investigation; visualization; writing – review and editing. **Elisa A Waxman:** Data curation; formal analysis; validation;

 © 2023 The Authors

investigation; visualization; methodology; writing – original draft. **Andrea Ballabio:** Resources; supervision; validation; methodology; writing – review and editing. **Thomas Dierks:** Resources; supervision; validation. **André Fischer:** Supervision; validation; investigation; visualization; methodology; writing – original draft; writing – review and editing. **Deborah L French:** Supervision; investigation; methodology; writing – review and editing. **Michael H Gelb:** Supervision; validation; investigation; methodology; writing – review and editing. **Jutta Gärtner:** Conceptualization; formal analysis; supervision; funding acquisition; validation; methodology; writing – original draft; project administration; writing – review and editing.

Disclosure and competing interests statement

LS, MK, JG, TD, KR, and MB have filed a patent application based on parts of the results from this study (US patent application number 16591051). AB is a co-founder of CASMA Therapeutics and Advisory Board member of Avilar Therapeutics and of Next Generation Diagnostics. MHG is a consultant for PerkinElmer Corp. and a co-founder of GelbChem LLC. ZMH is a consultant for GelbChem LLC. All other authors declare that they have no conflict of interest.

For more information

MSD Action Foundation: www.savingdylan.com; United MSD Foundation: https://curemsd.org

References

Adang LA, Schlotawa L, Groeschel S, Kehrer C, Harzer K, Staretz-Chacham O, Silva TO, Schwartz IVD, Gartner J, De Castro M *et al* (2020) Natural history of multiple sulfatase deficiency: retrospective phenotyping and functional variant analysis to characterize an ultra-rare disease. *J Inherit Metab Dis* 43: 1298–1309

Ahrens-Nicklas R, Schlotawa L, Ballabio A, Brunetti-Pierri N, De Castro M, Dierks T, Eichler F, Ficicioglu C, Finglas A, Gaertner J *et al* (2018) Complex care of individuals with multiple sulfatase deficiency: clinical cases and consensus statement. *Mol Genet Metab* 123: 337–346

Baum H, Dodgson KS, Spencer B (1959) The assay of arylsulphatases A and B in human urine. *Clin Chim Acta* 4: 453–455

Brown MS, Radhakrishnan A, Goldstein JL (2018) Retrospective on cholesterol homeostasis: the central role of scap. *Annu Rev Biochem* 87: 783–807

Busche A, Hennermann JB, Burger F, Proquitte H, Dierks T, von Arnim-Baas A, Horn D (2009) Neonatal manifestation of multiple sulfatase deficiency. *Eur J Pediatr* 168: 969–973

Cappuccio G, Alagia M, Brunetti-Pierri N (2020) A systematic cross-sectional survey of multiple sulfatase deficiency. *Mol Genet Metab* 130: 283–288

Carlson B (2004) Payers following FDA effort to integrate pharmacogenomics into drug development. *Biotechnol Healthc* 1: 12–14

Cheng X, Li J, Guo D (2018) SCAP/SREBPs are central players in lipid metabolism and novel metabolic targets in cancer therapy. *Curr Top Med Chem* 18: 484–493

Concordet JP, Haeussler M (2018) CRISPOR: intuitive guide selection for CRISPR/Cas9 genome editing experiments and screens. *Nucleic Acids Res* 46: W242–W245

Cosma MP, Pepe S, Annunziata I, Newbold RF, Grompe M, Parenti G, Ballabio A (2003) The multiple sulfatase deficiency gene encodes an essential and limiting factor for the activity of sulfatases. *Cell* 113: 445–456

David M, Hodak E, Lowe NJ (1988) Adverse effects of retinoids. *Med Toxicol Adverse Drug Exp* 3: 273–288

di Masi A, Leboffe L, De Marinis E, Pagano F, Cicconi L, Rochette-Egly C, Lo-Coco F, Ascenzi P, Nervi C (2015) Retinoic acid receptors: from molecular mechanisms to cancer therapy. *Mol Aspects Med* 41: 1–115

Dierks T, Schmidt B, Borissenko LV, Peng J, Preusser A, Mariappan M, von Figura K (2003) Multiple sulfatase deficiency is caused by mutations in the gene encoding the human C(alpha)-formylglycine generating enzyme. *Cell* 113: 435–444

Dierks T, Dickmanns A, Preusser-Kunze A, Schmidt B, Mariappan M, von Figura K, Ficner R, Rudolph MG (2005) Molecular basis for multiple sulfatase deficiency and mechanism for formylglycine generation of the human formylglycine-generating enzyme. *Cell* 121: 541–552

Diez-Roux G, Ballabio A (2005) Sulfatases and human disease. *Annu Rev Genomics Hum Genet* 6: 355–379

Dos Santos Guilherme M, Stoye NM, Rose-John S, Garbers C, Fellgiebel A, Endres K (2019) The synthetic retinoid acitretin increases IL-6 in the central nervous system of Alzheimer disease model mice and human patients. *Front Aging Neurosci* 11: 182

Duriez PJ, Shah GM (1997) Cleavage of poly(ADP-ribose) polymerase: a sensitive parameter to study cell death. *Biochem Cell Biol* 75: 337–349

Duvic M, Hymes K, Heald P, Breneman D, Martin AG, Myskowski P, Crowley C, Yocum RC, Bexarotene Worldwide Study Group (2001a) Bexarotene is effective and safe for treatment of refractory advanced-stage cutaneous T-cell lymphoma: multinational phase II-III trial results. *J Clin Oncol* 19: 2456–2471

Duvic M, Martin AG, Kim Y, Olsen E, Wood GS, Crowley CA, Yocum RC, Worldwide Bexarotene Study Group (2001b) Phase 2 and 3 clinical trial of oral bexarotene (Targretin capsules) for the treatment of refractory or persistent early-stage cutaneous T-cell lymphoma. *Arch Dermatol* 137: 581–593

Eid W, Dauner K, Courtney KC, Gagnon A, Parks RJ, Sorisky A, Zha X (2017) mTORC1 activates SREBP-2 by suppressing cholesterol trafficking to lysosomes in mammalian cells. *Proc Natl Acad Sci* 114: 7999–8004

Evans RM, Mangelsdorf DJ (2014) Nuclear receptors, RXR, and the big bang. *Cell* 157: 255–266

Fleming A, Xuan LZ, Sanchez-Elexpuru G, Williams SV, Windell D, Gelb MH, Herbst ZM, Schlotawa L, Rubinsztein DC (2022) Unexpected phenotype reversion and survival in a zebrafish model of multiple sulfatase deficiency. *Front Cell Dev Biol* 10: 843079

Fuller M, Rozaklis T, Ramsay SL, Hopwood JJ, Meikle PJ (2004) Disease-specific markers for the mucopolysaccharidoses. *Pediatr Res* 56: 733–738

Geng H, Whiteley G, Ribbens J, Zheng W, Southall N, Hu X, Marugan JJ, Ferrer M, Maegawa GH (2011) Novel patient cell-based HTS assay for identification of small molecules for a lysosomal storage disease. *PLoS ONE* 6: e29504

Germain DP, Giugliani R, Hughes DA, Mehta A, Nicholls K, Barisoni L, Jennette CJ, Bragat A, Castelli J, Sitaraman S *et al* (2012) Safety and pharmacodynamic effects of a pharmacological chaperone on alpha-galactosidase a activity and globotriaosylceramide clearance in Fabry disease: report from two phase 2 clinical studies. *Orphanet J Rare Dis* 7: 91

Ghosal K, Haag M, Verghese PB, West T, Veenstra T, Braunstein JB, Bateman RJ, Holtzman DM, Landreth GE (2016) A randomized controlled study to evaluate the effect of bexarotene on amyloid-beta and apolipoprotein E metabolism in healthy subjects. *Alzheimers Dement (N Y)* 2: 110–120

Guerra WF, Verity MA, Fluharty AL, Nguyen HT, Philippart M (1990) Multiple sulfatase deficiency: clinical, neuropathological, ultrastructural and biochemical studies. *J Neuropathol Exp Neurol* 49: 406–423

© 2023 The Authors

Hofmann B, Stege H, Ruzicka T, Lehmann P (1999) Effect of topical tazarotene in the treatment of congenital ichthyoses. *Br J Dermatol* 141: 642 – 646

Hughes PJ, Zhao Y, Chandraratna RA, Brown G (2006) Retinoid-mediated stimulation of steroid sulfatase activity in myeloid leukemic cell lines requires RARalpha and RXR and involves the phosphoinositide 3-kinase and ERK-MAP kinase pathways. *J Cell Biochem* 97: 327 – 350

Jones PH, Burnett RD, Fainaru I, Nadolny P, Walker P, Yu Z, Tang-Liu D, Ganesan TS, Talbot DC, Harris AL *et al* (2003) A phase 1 study of tazarotene in adults with advanced cancer. *Br J Cancer* 89: 808 – 815

Kang JX, Bell J, Leaf A, Beard RL, Chandraratna RA (1998) Retinoic acid alters the intracellular trafficking of the mannose-6-phosphate/insulin-like growth factor II receptor and lysosomal enzymes. *Proc Natl Acad Sci USA* 95: 13687 – 13691

Karpova EA, Voznyi Ya V, Keulemans JL, Hoogeveen AT, Winchester B, Tsvetkova IV, van Diggelen OP (1996) A fluorimetric enzyme assay for the diagnosis of Sanfilippo disease type a (MPS IIIA). *J Inherit Metab Dis* 19: 278 – 285

Khalil S, Bardawil T, Stephan C, Darwiche N, Abbas O, Kibbi AG, Nemer G, Kurban M (2017) Retinoids: a journey from the molecular structures and mechanisms of action to clinical uses in dermatology and adverse effects. *J Dermatolog Treat* 28: 684 – 696

Klaassen I, Brakenhoff RH, Smeets SJ, Snow GB, Braakhuis BJ (1999) Considerations for in vitro retinoid experiments: importance of protein interaction. *Biochim Biophys Acta* 1427: 265 – 275

le Maire A, Teyssier C, Balaguer P, Bourguet W, Germain P (2019) Regulation of RXR-RAR heterodimers by RXR- and RAR-specific ligands and their combinations. *Cell* 8: 1392

Lloyd-Evans E, Haslett LJ (2016) The lysosomal storage disease continuum with ageing-related neurodegenerative disease. *Ageing Res Rev* 32: 104 – 121

Macaulay RJ, Lowry NJ, Casey RE (1998) Pathologic findings of multiple sulfatase deficiency reflect the pattern of enzyme deficiencies. *Pediatr Neurol* 19: 372 – 376

Maguire JA, Gagne AL, Jobaliya CD, Gandre-Babbe S, Gadue P, French DL (2016) Generation of human control iPS cell line CHOPWT10 from healthy adult peripheral blood mononuclear cells. *Stem Cell Res* 16: 338 – 341

Martin-Maestro P, Sproul A, Martinez H, Paquet D, Gerges M, Noggle S, Starkov AA (2019) Autophagy induction by bexarotene promotes mitophagy in presenilin 1 familial Alzheimer's disease iPSC-derived neural stem cells. *Mol Neurobiol* 56: 8220 – 8236

Martinez Hernandez A, Urbanke H, Gillman AL, Lee J, Ryazanov S, Agbemenyah HY, Benito E, Jain G, Kaurani L, Grigorian G, et al. (2018) The diphenylpyrazole compound anle138b blocks Abeta channels and rescues disease phenotypes in a mouse model for amyloid pathology. *EMBO Mol Med* 10: 32 – 47

McPherson R, Gauthier A (2004) Molecular regulation of SREBP function: the Insig-SCAP connection and isoform-specific modulation of lipid synthesis. *Biochem Cell Biol* 82: 201 – 211

Miller VA, Benedetti FM, Rigas JR, Verret AL, Pfister DG, Straus D, Kris MG, Crisp M, Heyman R, Loewen GR *et al* (1997) Initial clinical trial of a selective retinoid X receptor ligand, LGD1069. *J Clin Oncol* 15: 790 – 795

Mollereau B, Manie S, Napoletano F (2014) Getting the better of ER stress. *J Cell Commun Signal* 8: 311 – 321

Moncan M, Mnich K, Blomme A, Almanza A, Samali A, Gorman AM (2021) Regulation of lipid metabolism by the unfolded protein response. *J Cell Mol Med* 25: 1359 – 1370

Napoli JL (2017) Cellular retinoid binding-proteins, CRBP, CRABP, FABP5: effects on retinoid metabolism, function and related diseases. *Pharmacol Ther* 173: 19 – 33

Platt FM, Boland B, van der Spoel AC (2012) The cell biology of disease: lysosomal storage disorders: the cellular impact of lysosomal dysfunction. *J Cell Biol* 199: 723 – 734

Pushpakom S, Iorio F, Eyers PA, Escott KJ, Hopper S, Wells A, Doig A, Guilliams T, Latimer J, McNamee C *et al* (2019) Drug repurposing: progress, challenges and recommendations. *Nat Rev Drug Discov* 18: 41 – 58

Schindelin J, Arganda-Carreras I, Frise E, Kaynig V, Longair M, Pietzsch T, Preibisch S, Rueden C, Saalfeld S, Schmid B *et al* (2012) Fiji: an open-source platform for biological-image analysis. *Nat Methods* 9: 676 – 682

Schlotawa L, Ennemann EC, Radhakrishnan K, Schmidt B, Chakrapani A, Christen HJ, Moser H, Steinmann B, Dierks T, Gartner J (2011) SUMF1 mutations affecting stability and activity of formylglycine generating enzyme predict clinical outcome in multiple sulfatase deficiency. *Eur J Hum Genet* 19: 253 – 261

Schlotawa L, Radhakrishnan K, Baumgartner M, Schmid R, Schmidt B, Dierks T, Gartner J (2013) Rapid degradation of an active formylglycine generating enzyme variant leads to a late infantile severe form of multiple sulfatase deficiency. *Eur J Hum Genet* 21: 1020 – 1023

Schlotawa L, Wachs M, Bernhard O, Mayer FJ, Dierks T, Schmidt B, Radhakrishnan K (2018) Recognition and ER quality control of misfolded Formylglycine-generating enzyme by protein disulfide isomerase. *Cell Rep* 24: 27 – 37

Schlotawa L, Dierks T, Christoph S, Cloppenburg E, Ohlenbusch A, Korenke GC, Gartner J (2019) Severe neonatal multiple sulfatase deficiency presenting with hydrops fetalis in a preterm birth patient. *JIMD Rep* 49: 48 – 52

Schlotawa L, Preiskorn J, Ahrens-Nicklas R, Schiller S, Adang LA, Gartner J, Friede T (2020) A systematic review and meta-analysis of published cases reveals the natural disease history in multiple sulfatase deficiency. *J Inherit Metab Dis* 43: 1288 – 1297

Settembre C, Annunziata I, Spampanato C, Zarcone D, Cobellis G, Nusco E, Zito E, Tacchetti C, Cosma MP, Ballabio A (2007) Systemic inflammation and neurodegeneration in a mouse model of multiple sulfatase deficiency. *Proc Natl Acad Sci USA* 104: 4506 – 4511

Shyu RY, Wang CH, Wu CC, Chen ML, Lee MC, Wang LK, Jiang SY, Tsai FM (2016) Tazarotene-induced gene 1 enhanced cervical cell autophagy through transmembrane protein 192. *Mol Cells* 39: 877 – 887

Sorrentino NC, Presa M, Attanasio S, Cacace V, Sofia M, Zuberi A, Ryan J, Ray S, Petkovic I, Radhakrishnan K *et al* (2022) New mouse models with hypomorphic SUMF1 variants mimic attenuated forms of multiple sulfatase deficiency. *J Inherit Metab Dis* https://doi.org/10.1002/jimd.12577

Standeven AM, Johnson AT, Escobar M, Chandraratna RA (1996) Specific antagonist of retinoid toxicity in mice. *Toxicol Appl Pharmacol* 138: 169 – 175

Steckel F, Hasilik A, von Figura K (1983) Biosynthesis and maturation of arylsulfatase B in normal and mutant cultured human fibroblasts. *J Biol Chem* 258: 14322 – 14326

Strittmatter SM (2014) Overcoming drug development bottlenecks with repurposing: old drugs learn new tricks. *Nat Med* 20: 590 – 591

Talpur R, Cox K, Duvic M (2009) Efficacy and safety of topical tazarotene: a review. *Expert Opin Drug Metab Toxicol* 5: 195 – 210

Tang-Liu DD, Matsumoto RM, Usansky JI (1999) Clinical pharmacokinetics and drug metabolism of tazarotene: a novel topical treatment for acne and psoriasis. *Clin Pharmacokinet* 37: 273 – 287

Trigo D, Goncalves MB, Corcoran JPT (2019) The regulation of mitochondrial dynamics in neurite outgrowth by retinoic acid receptor beta signaling. *FASEB J* 33: 7225 – 7235

Trivedi PC, Bartlett JJ, Pulinilkunnil T (2020) Lysosomal biology and function: modern view of cellular debris bin. *Cell* 9: 1131

 © 2023 The Authors

van Diggelen OP, Zhao H, Kleijer WJ, Janse HC, Poorthuis BJ, van Pelt J, Kamerling JP, Galjaard H (1990) A fluorimetric enzyme assay for the diagnosis of Morquio disease type A (MPS IV a). *Clin Chim Acta* 187: 131 – 139

Verheyen S, Blatterer J, Speicher MR, Bhavani GS, Boons GJ, Ilse MB, Andrae D, Spross J, Vaz FM, Kircher SG *et al* (2021) Novel subtype of mucopolysaccharidosis caused by arylsulfatase K (ARSK) deficiency. *J Med Genet* 59: 957 – 964

Weindl G, Roeder A, Schafer-Korting M, Schaller M, Korting HC (2006) Receptor-selective retinoids for psoriasis: focus on tazarotene. *Am J Clin Dermatol* 7: 85 – 97

Wu X, Katz E, Della Valle MC, Mascioli K, Flanagan JJ, Castelli JP, Schiffmann R, Boudes P, Lockhart DJ, Valenzano KJ *et al* (2011) A pharmacogenetic approach to identify mutant forms of alpha-galactosidase a that respond to a pharmacological chaperone for Fabry disease. *Hum Mutat* 32: 965 – 977

Xu M, Liu K, Swaroop M, Sun W, Dehdashti SJ, McKew JC, Zheng W (2014) A phenotypic compound screening assay for lysosomal storage diseases. *J Biomol Screen* 19: 168 – 175

Yang T, Espenshade PJ, Wright ME, Yabe D, Gong Y, Aebersold R, Goldstein JL, Brown MS (2002) Crucial step in cholesterol homeostasis: sterols promote binding of SCAP to INSIG-1, a membrane protein that facilitates retention of SREBPs in ER. *Cell* 110: 489 – 500

Yotsumoto T, Naitoh T, Kanaki T, Tsuruzoe N (2005) A retinoid X receptor antagonist, HX531, improves leptin resistance without increasing plasma leptin level in KK-ay mice under normal dietary conditions. *Metabolism* 54: 573 – 578

License: This is an open access article under the terms of the Creative Commons Attribution License, which permits use, distribution and reproduction in any medium, provided the original work is properly cited.

Expanded View Figures

Figure EV1. Hit drug evaluation on immortalized MSD fibroblasts.

A ARSA activity quantification (nmol/h/mg) after treatment of MSDi cells in 25 cm^2 cell culture flasks with a selection of four positive hit drugs at a final concentration of 10 μM for 6 days. Data represent mean ± SD of seven independent experiments (biological replicates). One-way ANOVA followed by Tukey's test for multiple comparisons. Difference against negative control: ****$P < 0.0001$. See details on P-values in Appendix Table S31.

B GALNS activity quantification (nmol/h/mg) after treatment of MSDi cells with a selection of four positive hit drugs at a final concentration of 10 μM for 3 days. Data represent mean ± SD of five independent experiments (biological replicates). One-way ANOVA followed by Tukey's test for multiple comparisons. Difference against negative control: **$P < 0.001$. See details on P-values in Appendix Table S32.

C GALNS activity quantification (nmol/h/mg) after treatment of MSDi cells with a selection of four positive hit drugs at a final concentration of 10 μM for 6 days. Data represent mean ± SD of five independent experiments (biological replicates). One-way ANOVA followed by Tukey's test for multiple comparisons. Difference against negative control: * $P < 0.05$, ** $P < 0.001$. See details on P-values in Appendix Table S33.

D β-galactosidase (betaGAL) activity quantification (nmol/h/mg) after treatment of MSDi cells with a selection of four positive hit drugs at a final concentration of 10 μM for 3 days. Data represent mean ± SD of five independent experiments (biological replicates). One-way ANOVA followed by Tukey's test for multiple comparisons.

E ß-hexosaminidase A and B (betaHEXAB) activity quantification (nmol/h/mg) after treatment of MSDi cells with a selection of four positive hit drugs at a final concentration of 10 μM for 3 days. Data represent mean ± SD of five independent experiments (biological replicates). One-way ANOVA followed by Tukey's test for multiple comparisons.

F Dose-response curve of ARSA activity calculated from data displayed in Fig 1C (MSDi cells, tazarotene treatment) by nonlinear regression analysis. Drug concentrations are displayed after transformation into log10 values and baseline activity (negative control, DMSO-only treatment) was manually referred to log-2. Dots and error bars represent mean ± SD.

G Dose-response curve of GALNS activity calculated from data displayed in Fig 1D (MSDi cells, tazarotene treatment) by nonlinear regression analysis. Drug concentrations are displayed after transformation into log10 values and baseline activity (negative control, DMSO-only treatment) was manually referred to log-2. Dots and error bars represent mean ± SD.

H Dose-response curve of ARSA activity calculated from data displayed in Fig 1F (MSDi cells, bexarotene treatment) by nonlinear regression analysis. Drug concentrations are displayed after transformation into log10 values and baseline activity (negative control, DMSO-only treatment) was manually referred to log-2. Dots and error bars represent mean ± SD.

I Dose-response curve of ARSA activity calculated from data displayed in Fig 1G (MSDi cells, tazarotene/bexarotene treatment) by nonlinear regression analysis. Drug concentrations are displayed after transformation into log10 values and baseline activity (negative control, DMSO-only treatment) was manually referred to log-2. Dots and error bars represent mean ± SD.

Source data are available online for this figure.

 © 2023 The Authors

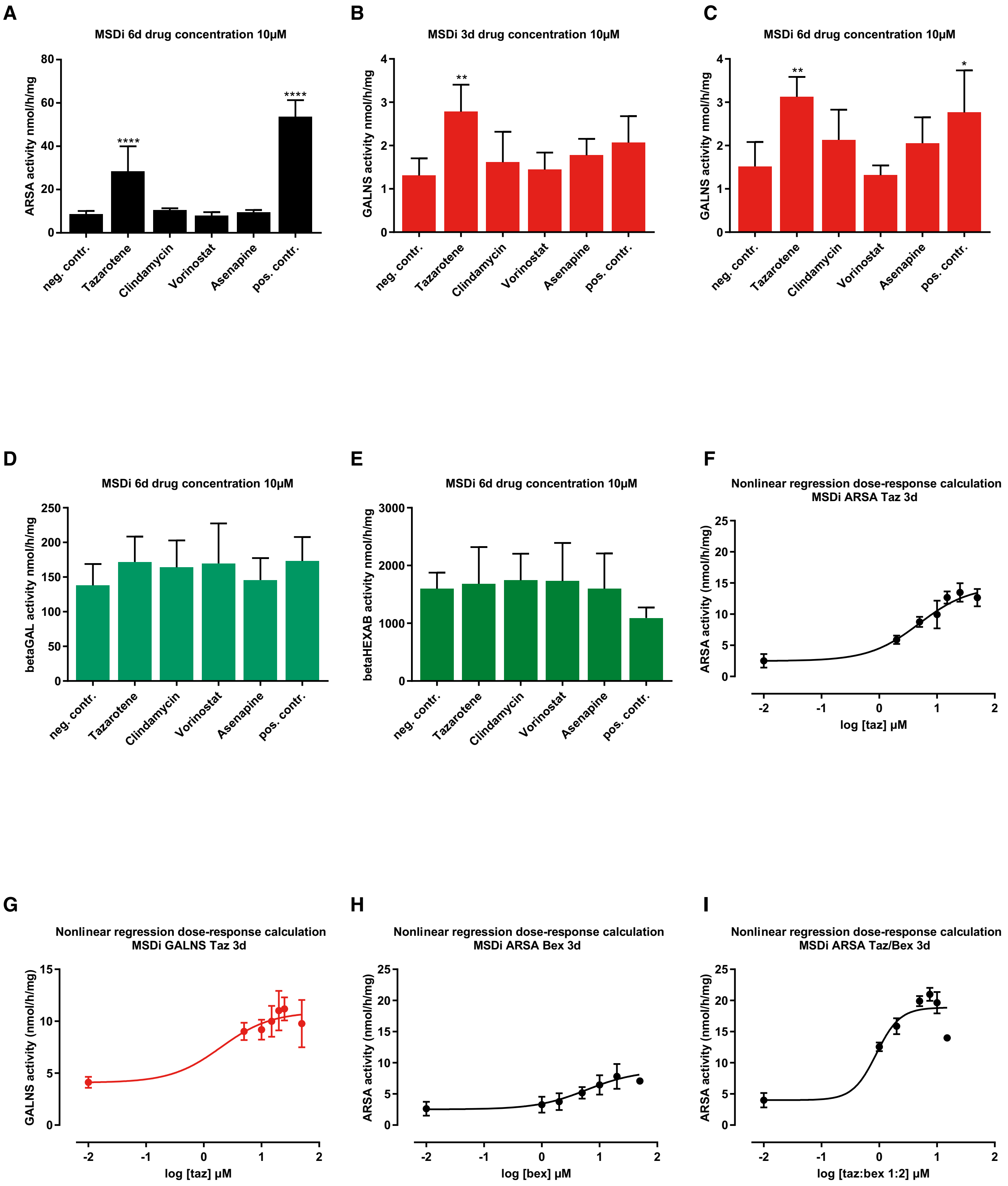

Figure EV1.

© 2023 The Authors

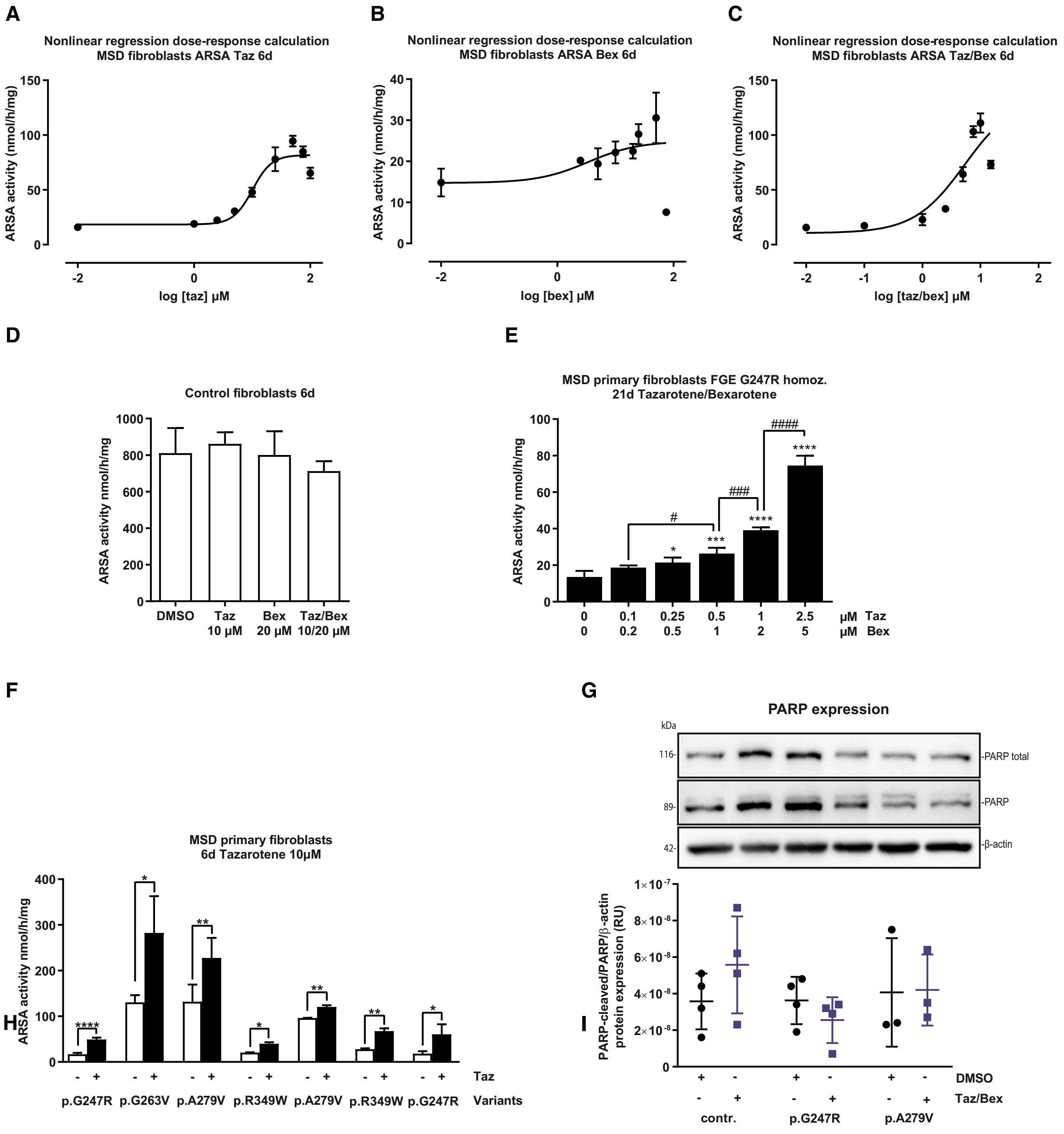

Figure EV2.

 © 2023 The Authors

Figure EV2. Treatment response and toxicity assessment.

A Dose-response curve of ARSA activity calculated from data displayed in Fig 2A (variant FGE Gly247Arg homozygous, tazarotene treatment) by nonlinear regression analysis. Drug concentrations are displayed after transformation into log10 values and baseline activity (negative control, DMSO-only treatment) was manually referred to log-2. Dots and error bars represent mean ± SD.

B Dose-response curve of ARSA activity calculated from data displayed in Fig 2B (variant FGE Gly247Arg homozygous, bexarotene treatment) by nonlinear regression analysis. Drug concentrations are displayed after transformation into log10 values and baseline activity (negative control, DMSO-only treatment) was manually referred to log-2. Dots and error bars represent mean ± SD.

C Dose-response curve of ARSA activity calculated from data displayed in Fig 2C (variant FGE Gly247Arg homozygous, tazarotene/bexarotene treatment) by nonlinear regression analysis. Drug concentrations are displayed after transformation into log10 values and baseline activity (negative control, DMSO-only treatment) was manually referred to log-2. Dots and error bars represent mean ± SD.

D ARSA activity quantification after treatment of five different control, non-MSD, fibroblast lines with tazarotene, bexarotene, and tazarotene/bexarotene in combination for 6 days referred to β-actin amounts and calculation of ARSA activity based on ARSA protein amount (specific ARSA activity). Data represent mean ± SD of five independent experiments (biological replicates) in duplicates to determine the range of normal ARSA activities and treatment response as the basis for the calculation of residual activities in MSD fibroblasts.

E ARSA activity quantification after simultaneous treatment of MSD primary fibroblasts (variant FGE Gly247Arg homozygous) with increasing concentrations of tazarotene and bexarotene in a fixed combination of 1:2 for 21 days. Data represent mean ± SD of four independent experiments (biological replicates). One-way ANOVA followed by Tukey's test for multiple comparisons. Displayed are significance levels for the next significant difference between adjacent concentrations/conditions. # $P < 0.05$, ### $P < 0.001$, #### $P < 0.0001$. Difference against 0/0 μM control: *$P < 0.05$, ***$P < 0.001$, ****$P < 0.0001$. See details on *P*-values in Appendix Table S34.

F Quantification of ARSA activities in MSD primary fibroblasts with different homozygous *SUMF1* mutations (FGE Gly247Arg, FGE Gly263Val, FGE Ala279Val, FGE Arg349Trp) after 6 days of treatment with tazarotene 10 μM. Data represent mean ± SD of 2–5 independent experiments (biological replicates). One-way ANOVA followed by Tukey's test for multiple comparisons. *$P < 0.05$, **$P < 0.01$, ****$P < 0.0001$. See details on *P*-values in Appendix Table S35.

G Upper panel: Representative pictures of Western Blot analysis of (PARP) and cleaved PARP in tazarotene/bexarotene-treated MSD primary fibroblasts (variant FGE Gly247Arg, FGE Ala279Val, homozygous) and control fibroblasts. β-actin expression served as loading control. Lower panel: Quantification of protein amounts from western blots displayed as ratio cleaved PARP to total PARP expression normalized to β-actin. Data represent mean ± SD of 3–4 independent experiments (biological replicates). Unpaired *t*-tests. No statistical differences. RU, relative units.

Source data are available online for this figure.

Figure EV3. Transcriptional response of MSD and retinoic acid gene targets upon tazarotene/bexarotene treatment.

A Gene expression analysis of genes in relation to retinoic acid receptor signaling of six different MSD primary fibroblast lines and five different control fibroblast lines after 6 days of treatment with tazarotene/bexarotene (10/20 μM) and DMSO, respectively. Changes in RPKM (reads per kilobase million) are displayed as mean ± SD of three independent experiments (biological replicates). One-way ANOVA test followed by Tukey's test for multiple comparisons. *$P < 0.05$, **$P < 0.01$, ***$P < 0.001$, ****$P < 0.0001$. See details on *P*-values in Appendix Table S36.

B Gene expression analysis of *SUMF1*- and FGE-interacting partners (nonsulfatases) of six different MSD primary fibroblast lines and five different control fibroblast lines after 6 days of treatment with tazarotene/bexarotene (10/20 μM) and DMSO, respectively. Changes in RPKM (reads per kilobase million) are displayed as mean ± SD of three independent experiments (biological replicates). One-way ANOVA test followed by Tukey's test for multiple comparisons. *$P < 0.05$. See details on *P*-values in Appendix Table S37.

C Gene expression analysis of sulfatases of six different MSD primary fibroblast lines and five different control fibroblast lines after 6 days of treatment with tazarotene/bexarotene (10/20 μM) and DMSO, respectively. Changes in RPKM (reads per kilobase million) are displayed as mean ± SD of three independent experiments (biological replicates). One-way ANOVA test followed by Tukey's test for multiple comparisons. *$P < 0.05$, ****$P < 0.0001$. See details on *P*-values in Appendix Table S38.

Source data are available online for this figure.

© 2023 The Authors

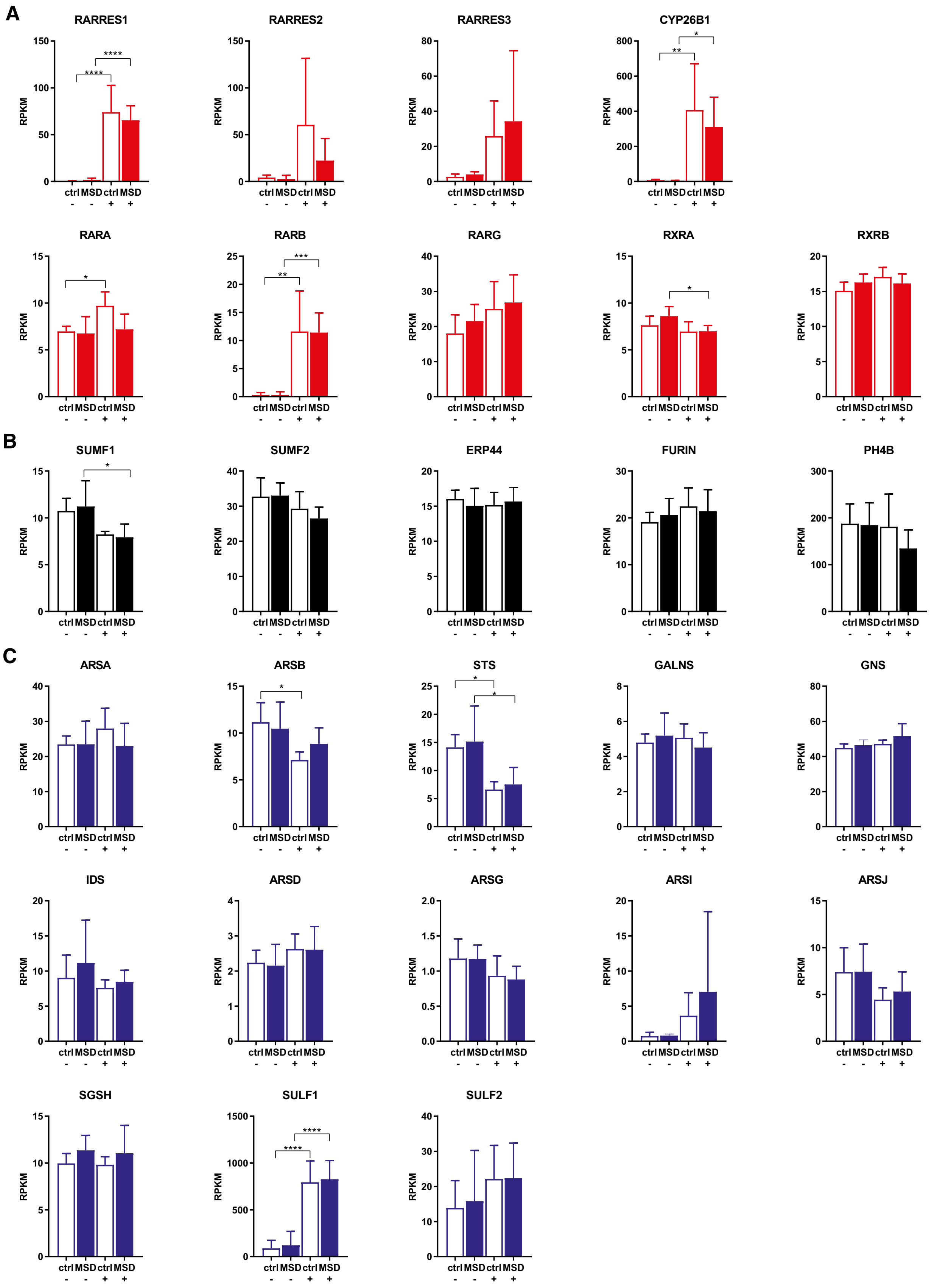

Figure EV3.

© 2023 The Authors

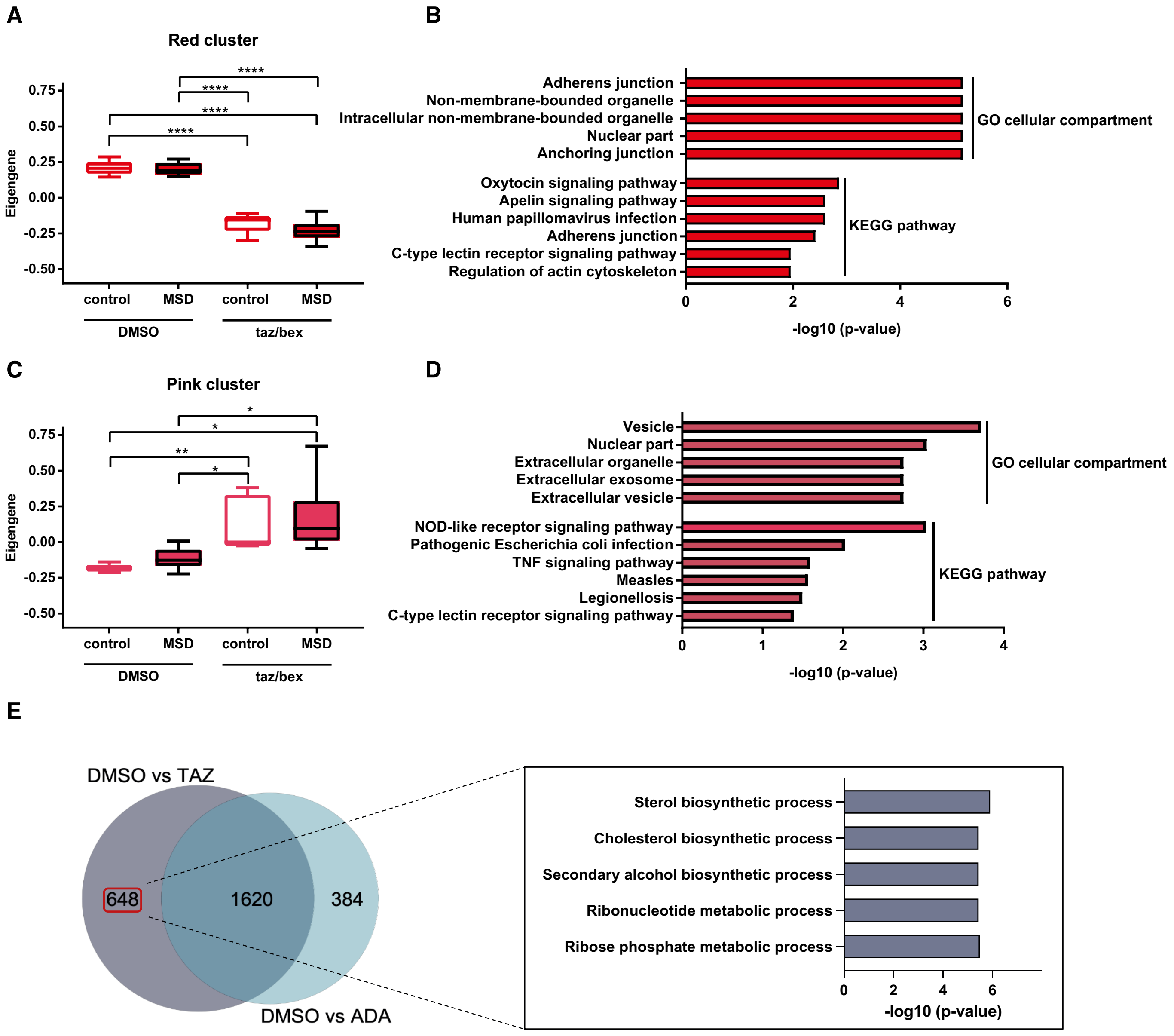

Figure EV4. Transcriptional response upon tazarotene and bexarotene treatment in MSD and control fibroblasts and differential transcriptional response in MSD fibroblasts upon treatment with tazarotene and adapalene.

A Eigengene analysis of the red gene cluster as determined by WGCN analysis after RNA sequencing of six different MSD primary fibroblast lines and five different control fibroblast lines and treatment with tazarotene/ bexarotene 10/20 μM or DMSO only, respectively, for 6 days. Data represent min to max box and whisker blots of Eigengene values ± SD of three independent experiments (biological replicates). One-way ANOVA test followed by Tukey's test for multiple comparisons. ****$P < 0.0001$. See details on P-values in Appendix Table S39.

B GO and KEGG pathway analysis of genes in the red cluster and log10 value of P-values. GO and KEGG pathway analysis of genes in the yellow cluster and their log10 value of P-values as the display for changes in gene expression.

C Eigengene analysis of the red gene cluster as determined by WGCN analysis after RNA sequencing of six different MSD primary fibroblast lines and five different control fibroblast lines and treatment with tazarotene/bexarotene 10/20 μM or DMSO only, respectively, for 6 days. Data represent min to max box and whisker blots of Eigengene values ± SD of three independent experiments (biological replicates). One-way ANOVA test followed by Tukey's test for multiple comparisons. *$P < 0.05$, **$P < 0.01$. See details on P-values in Appendix Table S40.

D GO and KEGG pathway analysis of genes in the red cluster and log10 value of P-values.

E Differential gene expression analysis after treatment of seven MSD primary fibroblast lines with tazarotene (sulfatase activity response) and adapalene (no sulfatase activity response) in triplicates for 6 days. Treatment with DMSO served as a negative control. Venn diagram and number of exclusively regulated genes for tazarotene treatment (TAZ) versus DMSO condition (left) and adapalene treatment (ADA) versus DMSO (right), as well as the number of overlapping genes identically regulated by both tazarotene and adapalene. GO pathway analysis and log10 value of P-values for tazarotene-only regulated genes.

Source data are available online for this figure.

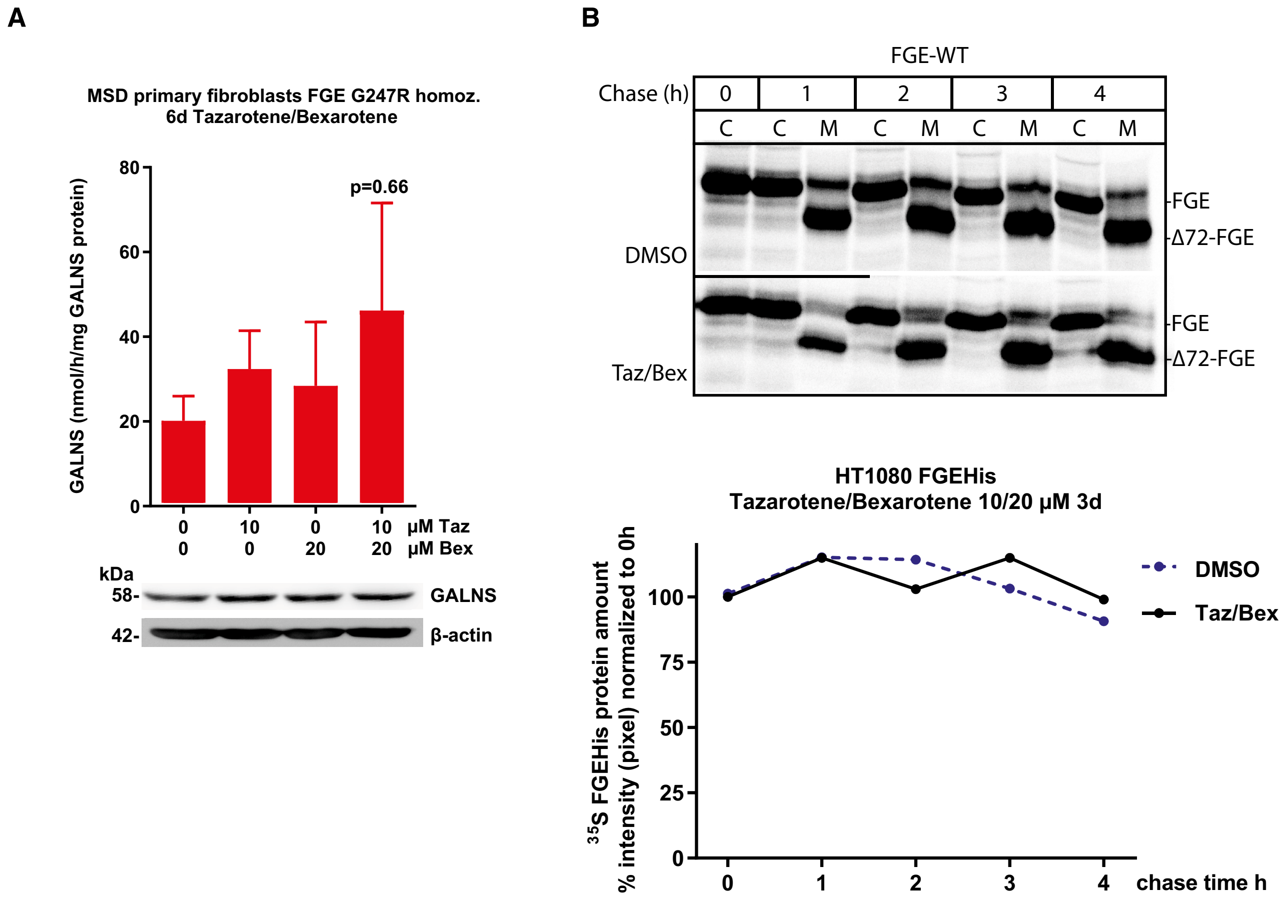

Figure EV5. Sulfatase-specific activity and protein expression and protein stability of wildtype FGE upon treatment.

A GALNS protein amount quantification after treatment of MSD primary fibroblasts (variant FGE Gly247Arg homozygous) with tazarotene, bexarotene, and tazarotene/bexarotene in combination for 6 days referred to β-actin amounts and calculation of GALNS activity based on GALNS protein amount (specific GALNS activity). Data represent mean ± SD three independent experiments (biological replicates). One-way ANOVA followed by Tukey's test for multiple comparisons.

B Pulse-chase-experiment in HT 1080 FGE wild-type (wt) cells after pretreatment with tazarotene/bexarotene and DMSO (control) for 3 days. Upper panel: autoradiogram of intracellular (full-length FGE, C) and cleaved and secreted (Δ72 FGE, M) ^{35}S isotope labeled FGE protein in either condition with a chase time of 4 h. Lower panel: Quantification of the autoradiogram, $n = 1$ experiment.

Source data are available online for this figure.

 © 2023 The Authors

Appendix

Content:

Appendix supplementary results, pages 1-6

Appendix supplementary figures, pages 7-19

Appendix supplementary figure legends, pages 20-28

Appendix supplementary tables, pages 29-67

Appendix supplementary results

Effects of tazarotene and bexarotene treatment on cell proliferation in MSD and control fibroblasts

MSD fibroblasts (p.Gly247Arg) and control fibroblasts were plated out at day 0 at standard cell quantity ($9x10^5$, medium) and additional quantities ($4.5x10^5$, low; $13.5x10^5$, high) and grown in presence of 0.1 % DMSO (v/v). Cells were harvested on day 3, 6, and 9, and analysed for cell count, total protein amount and ARSA activity in order to measure effects of cell density on all parameters. In parallel, MSD fibroblasts (p.Gly247Arg) and control fibroblasts were grown under standard conditions ($9x10^5$ at day 0) treated with 10 µM tazarotene, 20 µM bexarotene, and 10/20 µM tazarotene/bexarotene, respectively. Cells were harvested at the same time points for analysis of the same parameters.

We detected a time dependent significant increase of cell quantity in DMSO only treated MSD fibroblasts compared to day 0 independent of the starting cell quantity. Medium and high cell quantities led to a plateauing increase of cell quantity from day 3 onwards. Cell counts at day 9 were not significantly different in DMSO treated MSD fibroblasts (Appendix Fig. S5A, Appendix Table S3). Control fibroblasts also showed a time dependent significant increase of cell quantity in DMSO only treated independent of the starting cell quantity. The increase plateaued from day 3 onwards for DMSO treated cells plated at medium and high

quantity whereas low quantity cells showed persistent increase of cell number until day 9. Cell counts at day 9 were not significantly different in DMSO treated control fibroblasts (Appendix Fig. S5B, Appendix Table S4) Please note that MSD fibroblasts grew faster than control fibroblasts (Appendix Fig. S5A,B; Appendix Table S3,S4). Treatment with tazarotene, bexarotene and tazarotene/bexarotene, respectively, significantly influenced cell count in both lines with strongest effects in tazarotene/bexarotene treated cells (MSD fibroblasts day 9, DMSO medium: 37.8 x 10^5 cells, Taz: 24.9 x 10^5 cells, Bex: 26.3 x 10^5 cells, Taz/Bex: 20.1x10x105 cells).

Total protein also increased over time in MSD and control fibroblasts under DMSO conditions independent of cell quantity at start. There were no significant differences in total protein amount in DMSO treated MSD fibroblasts at day 6 and 9 and no differences for control fibroblasts from day 3-9 (Appendix Fig. S5C,D; Appendix Table S5). However, under treatment conditions, total protein amounts were significantly reduced in control fibroblasts treated with tazarotene or tazarotene/bexarotene at day 6 and at day 9 compared to DMSO treated cells plated at medium quantity (Appendix Fig. S5D, Appendix Table S6). Interestingly, protein amounts in treated MSD fibroblasts were only significantly reduced at day 6 when treated with tazarotene/bexarotene and compared to DMSO treated cells plated at medium and high quantity. This reduction resolved over time and neither treatment showed significant differences in total protein amount in MSD fibroblasts compared to for DMSO treated cells plated at medium and high quantity (Appendix Fig. S5C, Appendix Table S5).

ARSA activity did not show a time and cell-quantity dependent increase in MSD fibroblasts under DMSO only conditions (Appendix Fig. S5E, Appendix Table S7). However, there was a significant increase of ARSA activity starting at day 3 for tazarotene/bexarotene treated cells compared to DMSO conditions. From day 6 onwards tazarotene treatment led to a significant increase of ARSA activity compared to DMSO conditions. The highest increase

for tazarotene treated cells was 4.5-fold (day 9) and 7.8-fold for tazarotene/bexarotene (day 9), significantly higher than tazarotene treated cells (Appendix Fig. S5E, Appendix Table S7). Interestingly, endogenous ARSA activity was significantly increased at day 6 and 9 between DMSO only treated control fibroblasts plated at low quantity compared to for DMSO treated cells plated at medium and high quantity DMSO medium and high conditions (maximum 1.8-fold increase). Treatment with tazarotene, bexarotene or tazarotene/bexarotene led to no further increase compared to for DMSO treated cells plated at medium quantity conditions (Appendix Fig. S5F, Appendix Table S8).

To validate cell proliferation with a method that does not rely on maunual cell counting, we performed XTT assays in control and MSD fibroblasts. Of note, xtt assays are dependent on cellular metabolism, which in turn is influenced by cell count, size and protein amount. 6-day treatment with tazarotene, bexarotene, and tazarotene/bexarotene resulted in reduced cell proliferation of control fibroblasts when compared to cells treated with DMSO only. Changes in treated MSD fibroblasts were not significantly different (Appendix Fig. S6 A,C). Treatment with different concentrations of tazarotene/bexarotene for 6 days showed significantly reduced cell proliferation compared to DMSO treatment starting at 1/2 μM tazarotene/bexarotene in control fibroblasts (minimal proliferation 65% of DMSO control at 5/10 μM tazarotene/bexarotene). Again, no significant differences appeared in MSD primary fibroblasts (Appendix Fig. S6 B,D).

Adapalene increases transcription of retinoid target genes via RAR receptors without increasing sulfatase activities in MSD primary fibroblast cell lines

To identify a retinoid that worked on MSD cells but did not increase sulfatase activities we treated primary MSD fibroblasts (p.Gly247Arg) with a selection of retinoids representing generations of pharmacological development (1st generation tretinoin, 2nd generation

acitretin, 3rd generation adapalene) for six days at concentrations of 10 µM (adapalene 5 µM) and analysed three different sulfatase activities (ARSA, GALNS, STS). Tazarotene treatment (10 µM) served as positive control, DMSO treated cells as negative control. Neither retinoid was able to increase ARSA, GALNS, or STS activities (Appendix Fig. S7A). Next, we used the same retinoids and treatment conditions to analyse gene expression of retinoid targets by RT-PCR to prove that the selected retinoids entered treated cells and were effective without increasing sulfatase activities. Tretinoin, acitretein, and adapalene were all able to increase *RARB*, *CYP26B1*, and *RARRES1-3* gene expression compared to untreated controls. Significant differences of gene expression were seen for *CYP26B1* and *RARRES3* that was highest upon tazarotene treatment. *RARB*, *RARRES1* and *RARRES2* showed increased but no significantly different gene expression regardless of the retinoid used (Appendix Fig. S7B). Based on the results we chose to use adapalene, which belongs to the same pharmacological group of retinoid development like tazarotene and bexarotene (3rd generation retinoids), as a control retinoid for further experiments. Next, we wanted to rule out that we have missed the right dose for an effect on sulfatase activities in MSD fibroblasts and treated cells with four different concentrations of adapalene (1, 5, 10, 20 µM) for six days. Again, tazarotene treatment served as control. Neither ARSA nor GALNS activity increase could be detected at either concentration used (appendix figure S8A). Gene expression analysis of retinoid targets upon treatment with increasing concentrations showed increased expression levels at all four adapalene concentrations compared to DMSO conditions but significantly reduced expression levels at 20 µM concentration compared to lower concentrations likely because of visual adapalene toxicity on cells. *RARRES3* showed a trend towards differences only (Appendix Fig. S8B). Based on these results we chose a submaximal concentration of adapalene (5µM) for further experiments to avoid toxicity. Treatment of eight different MSD fibroblast lines with adapalene showed no ARSA activity increase regardless of the *SUMF1* mutation (Appendix Fig. S8C thereby ruling out mutation specific differences. A refined dose-

concentration analysis with adapalene treatment of MSD fibroblasts with concentrations starting as low as 100 nM and up tp 20 µM for six days still excluded any ARSA activity increase (Appendix Fig. S8D).Finally, we pretreated cells for 24 hours with the pan-RAR antagonist AGN193109 followed by 72 hours of simultaneous treatment with adapalene (control: tazarotene). Incubation with AGN193109 abrogated both adapalene and tazarotene induced retinoid target gene expression in MSD fibroblasts (Appendix Fig. S9) thereby proving that adapalene, like tazarotene, increased retinoid target gene expression in MSD fibroblasts via RAR receptors.

Tazarotene/bexarotene treatment did not change endogenous PDI protein expression but decreased PDI-mediated inhibition of FGE variants residual activity

PDI has been described as a pivotal interacting partner of FGE-variants with impact on residual FGE and dependent sulfatase activities by binding to misfolded FGE variants and referring them to early degradation (Schlotawa *et al*, 2018). In order to see if tazarotene/bexarotene treatment reduces PDI expression resulting in less FGE variant binding and increased FGE activity we analysed three different MSD primary fibroblast lines treated with either DMSO or tazarotene/bexarotene treatment for six days and one control fibroblast line by western blot. No differences could be detected thereby ruling out an effect of tazarotene/bexarotene on PDI expression (Appendix Fig. S12).

To further analyse how PDI interaction with FGE variants changes upon tazarotene/bexarotene treatment we repeated an experiment from our previous publication (Schlotawa *et al.*, 2018). We were aiming to see differences on the activation of steroid sulfatase (STS) by FGE-Ser155Pro variant with and without PDI co-expression and treatment of cells with either DMSO (vehicle control) or tazarotene/bexarotene (Appendix Fig. S13A,B). As compared to DMSO treated cells, tazarotene/bexarotene consistently led to an

increase in STS activity with and without FGE or FGE-variant co-expression. When compared to respective DMSO controls, in FGE-Ser155Pro alone expressing cells, tazarotene/bexarotene treatment led to an approx. 4.5-fold increase in activity while co-expression of PDI in drug treated cells led to a 7.5-fold increase (Appendix Fig. S13C). However, note that, this apparent increase in activity upon PDI co-expression in drug treated cells is due to the decrease in activity of FGE-Ser155Pro when PDI is co-expressed in DMSO treated cells. In DMSO treated cells, co-expression of PDI and FGE-Ser155Pro led to a 3-fold loss of Ser155Pro variants activity, in agreement to our previous work. Such a loss in activity is also observed in drug treated cells, however, only to 2-fold (Appendix Figure S13D). This indicates that co-expression of PDI has a minor effect on prohibiting FGE-Ser155Pro induced STS activation when cells are treated with tazarotene/bexarotene compared to DMSO treated cells. Supported by our data that tazarotene/bexarotene treatment improves the intracellular stability of FGE variants (Fig. 4), we think that it is plausible that tazarotene/bexarotene treatment reduces the proportion of misfolded FGE variants that bind to PDI and increase the proportion able to escape PDI recognition due to improved folding. This results in increased FGE functionality and the observed increase in sulfatase activities upon tazarotene/bexarotene treatment.

Appendix supplementary figures

Appendix figure S1

A

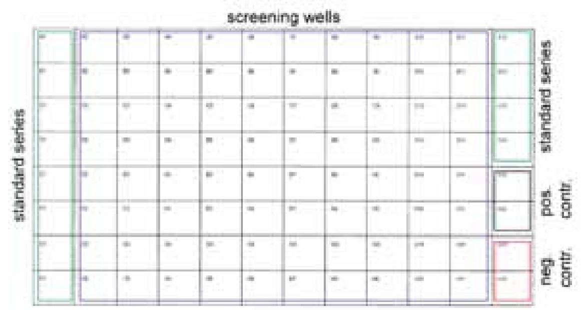

B

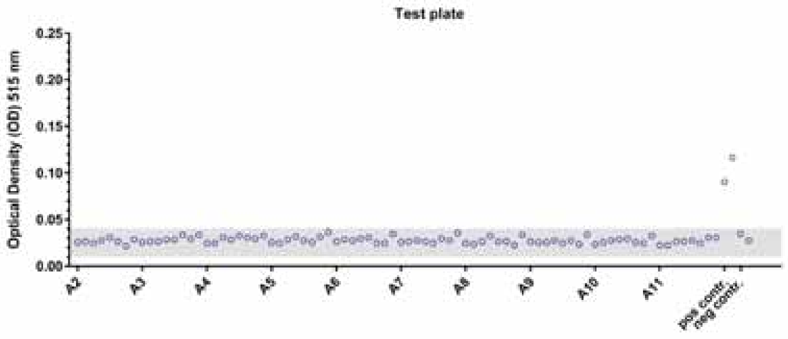

C

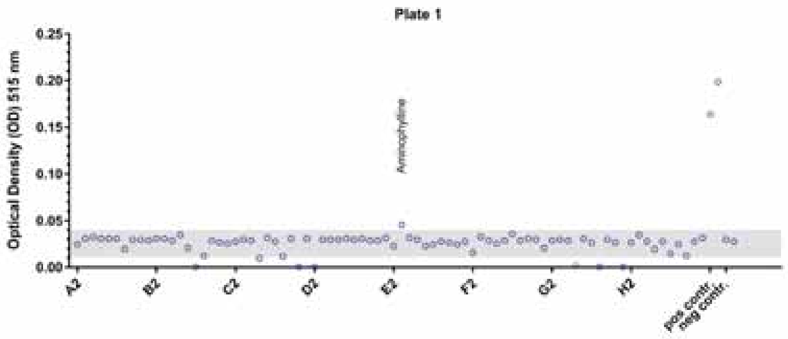

D

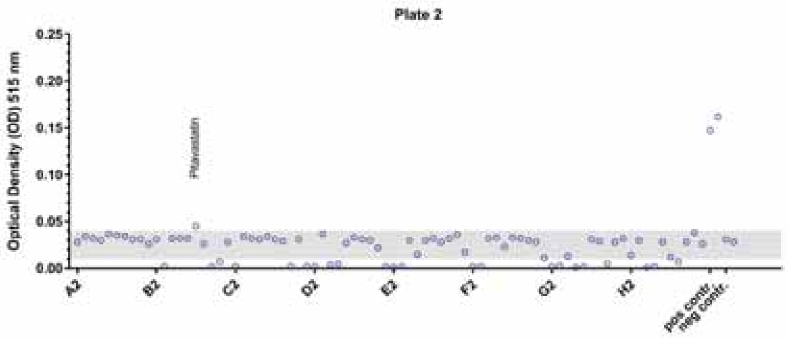

Appendix figure S2

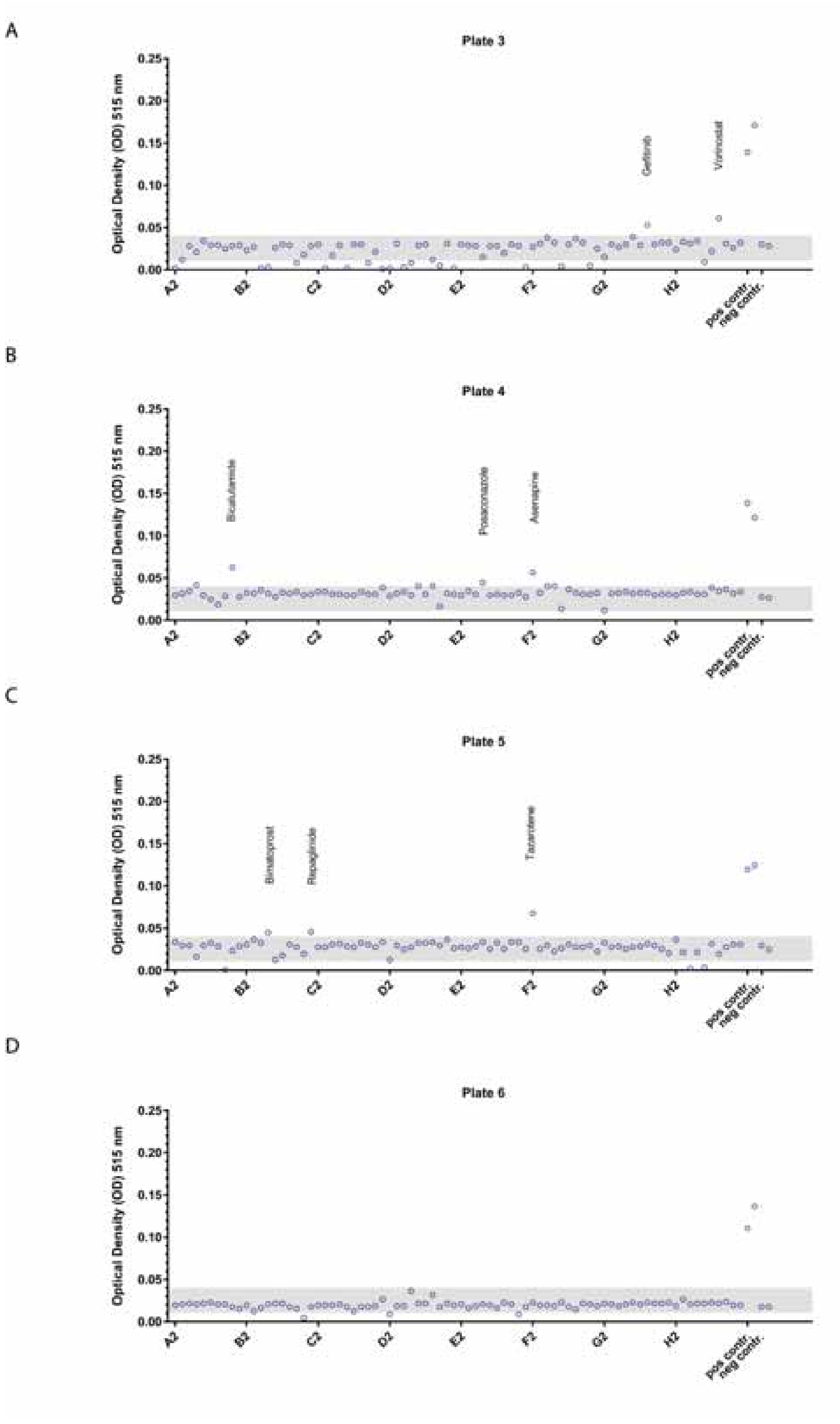

Appendix figure S3

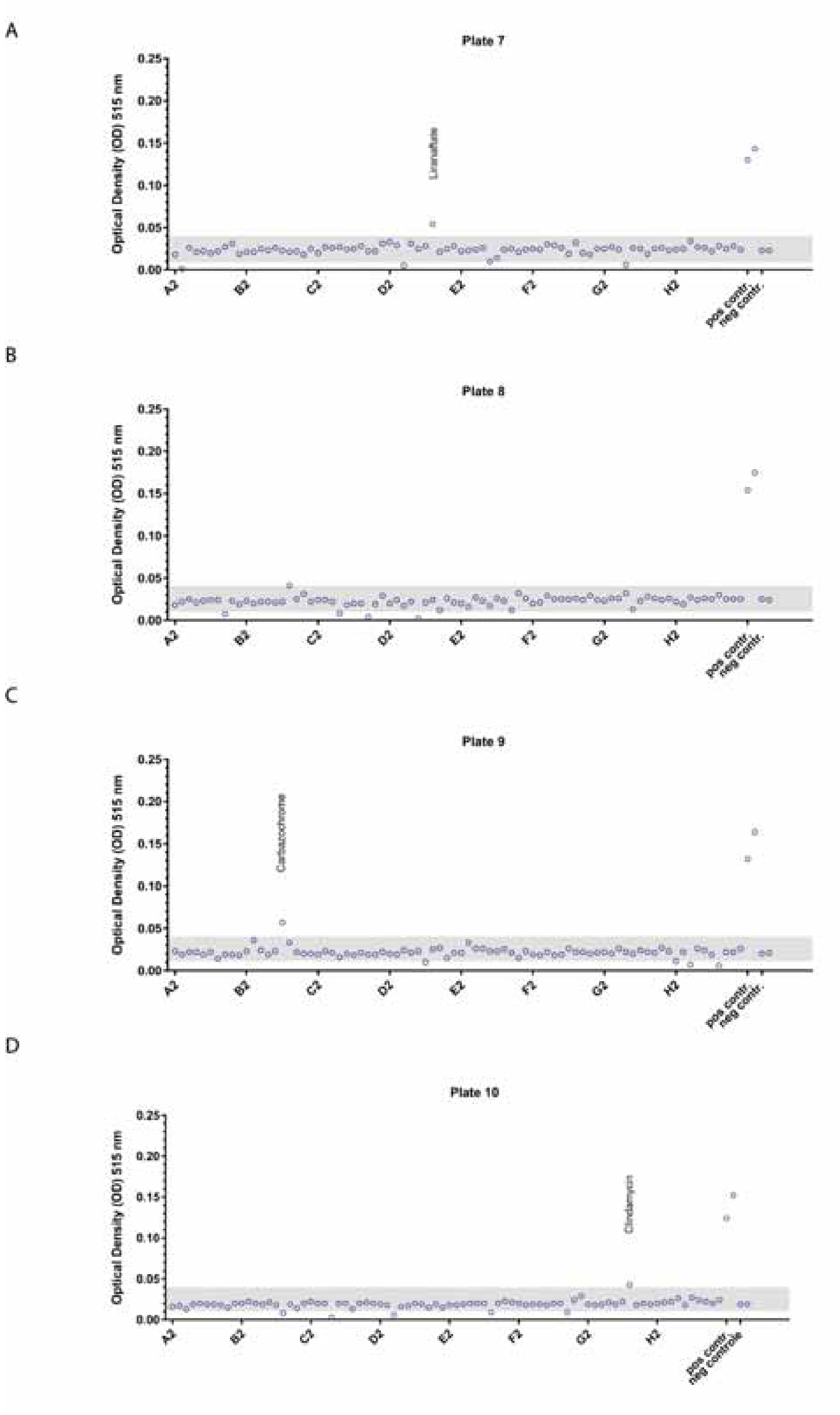

Appendix figure S4

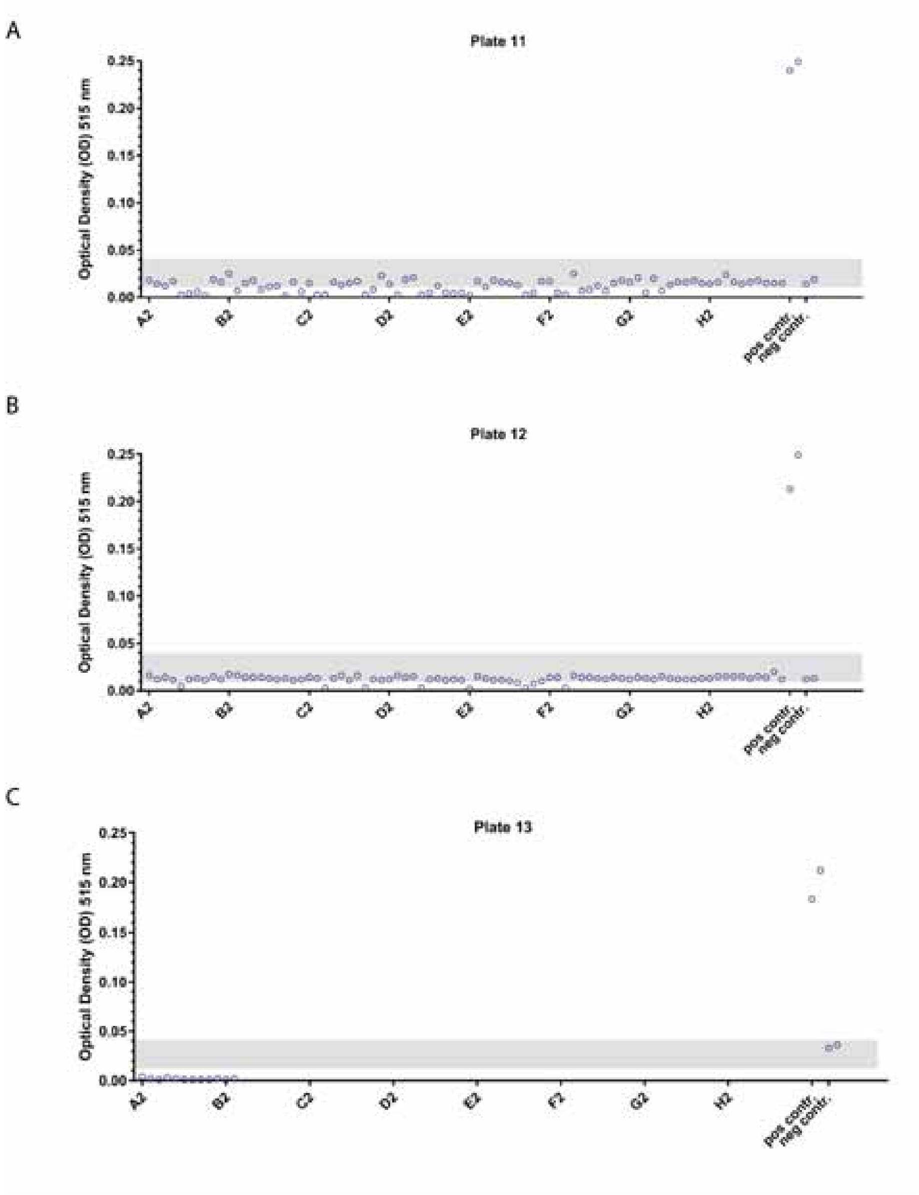

Appendix figure S5

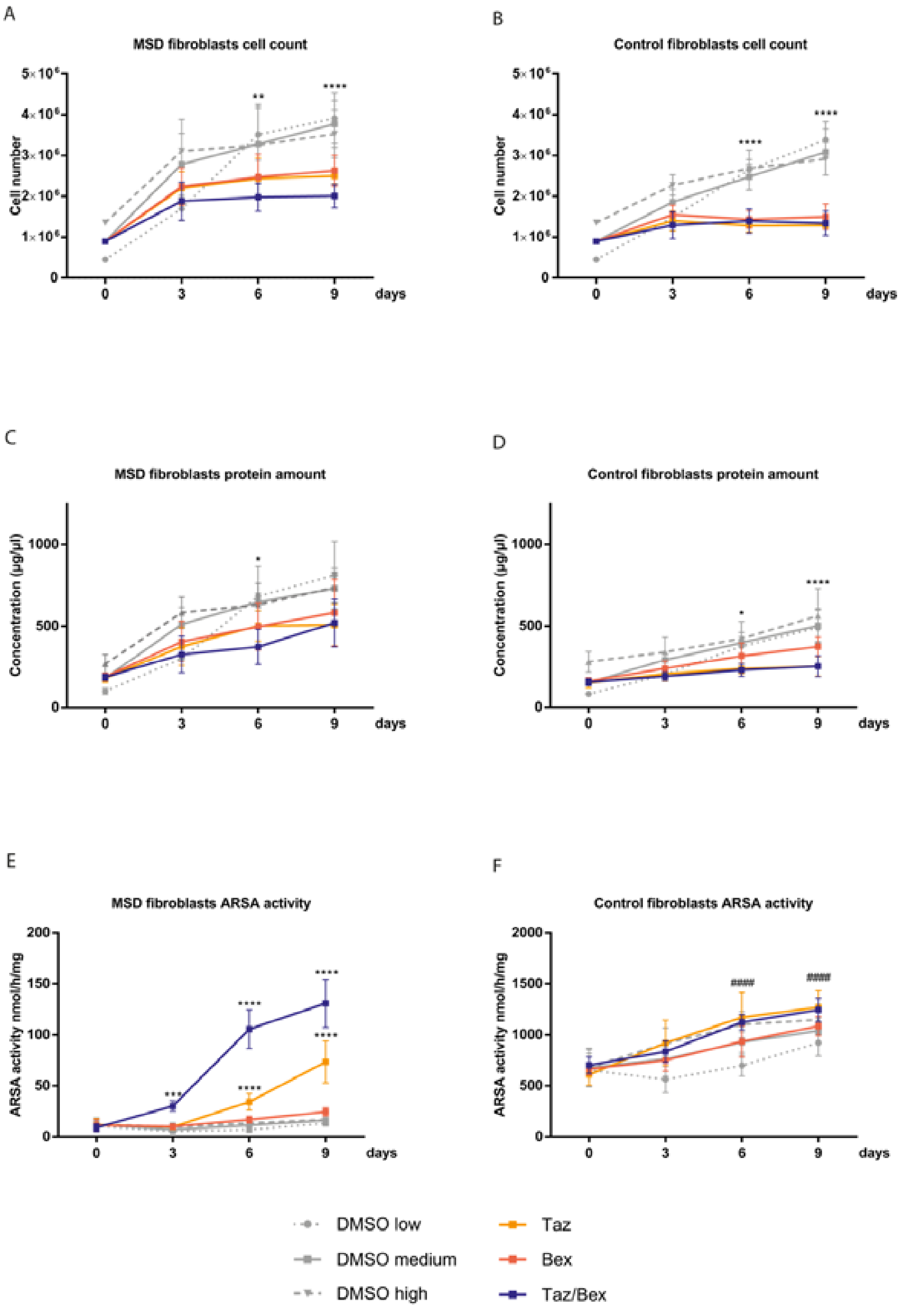

Appendix figure S6

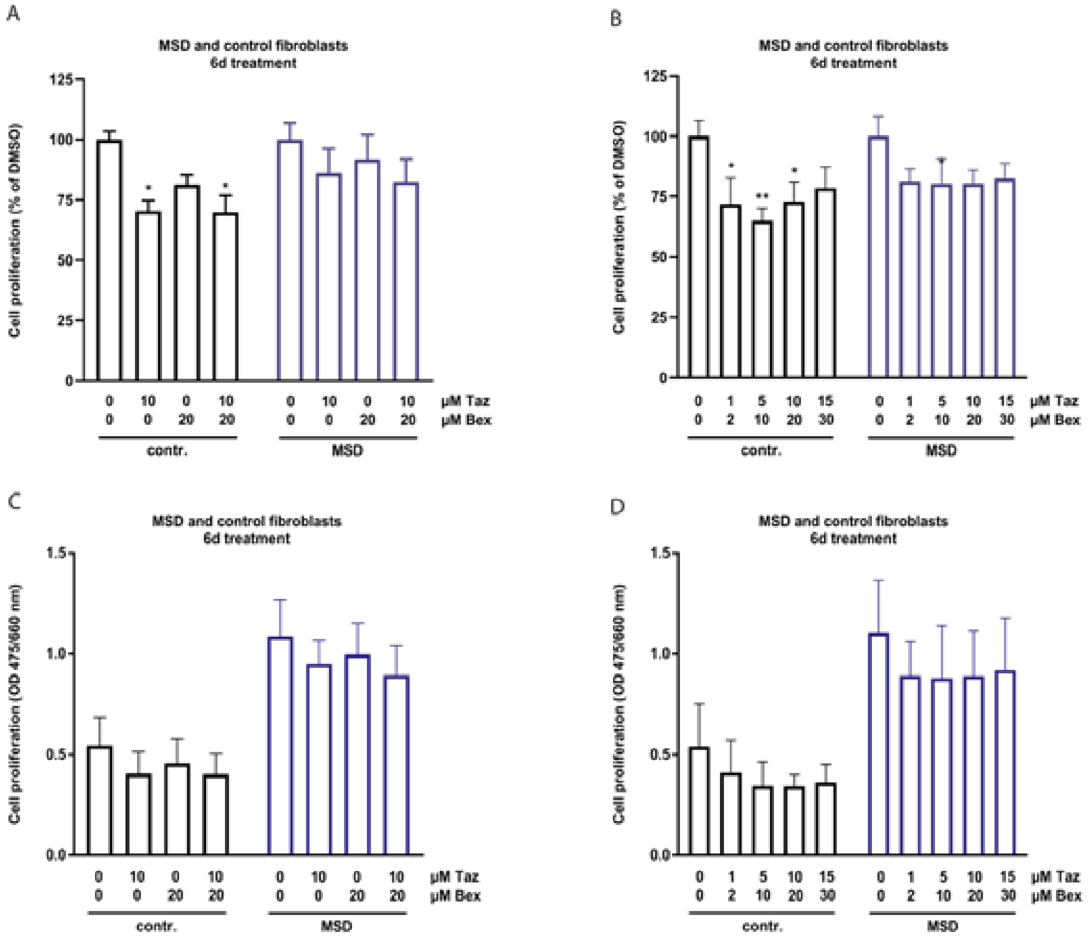

Appendix figure S7

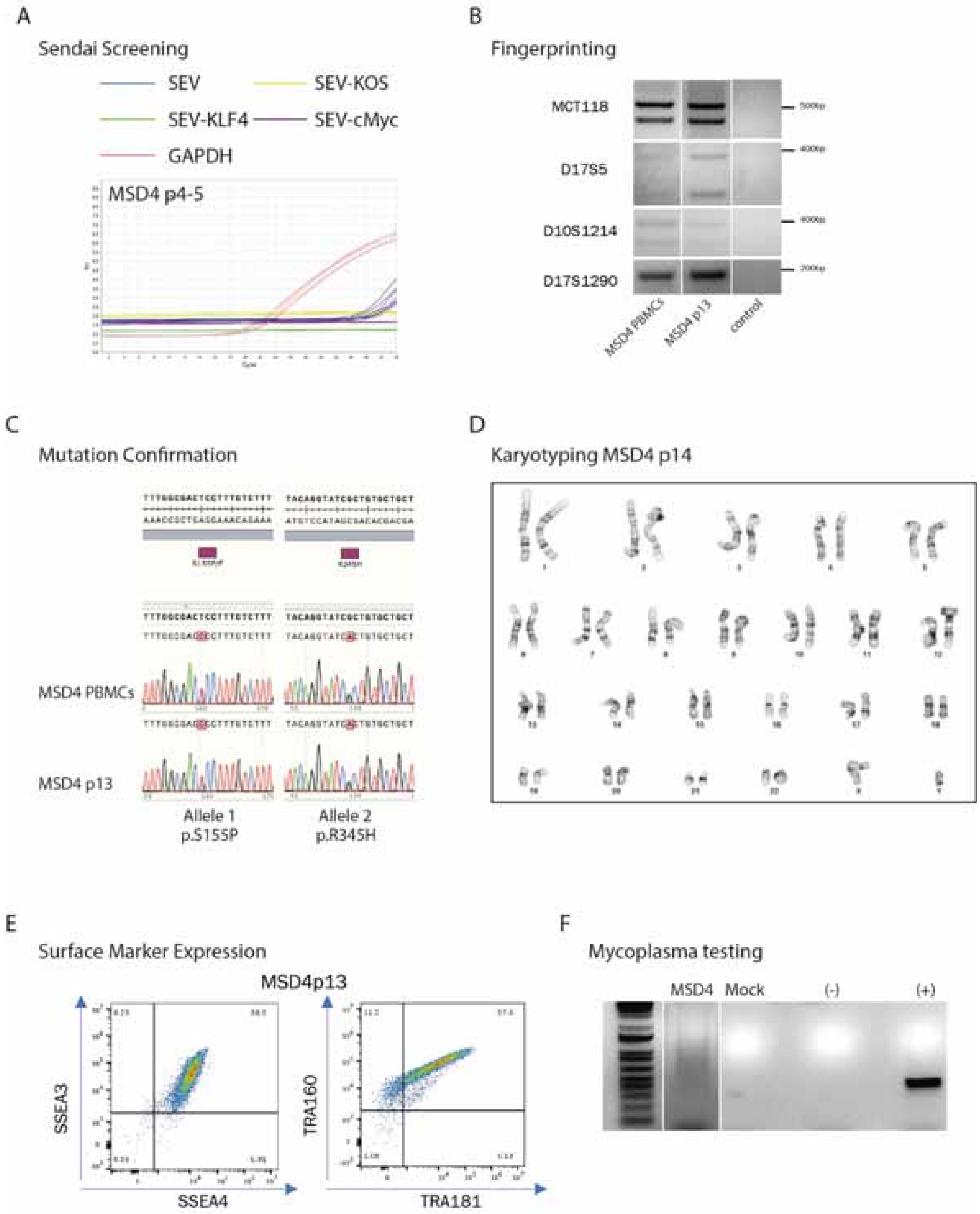

Appendix figure S8

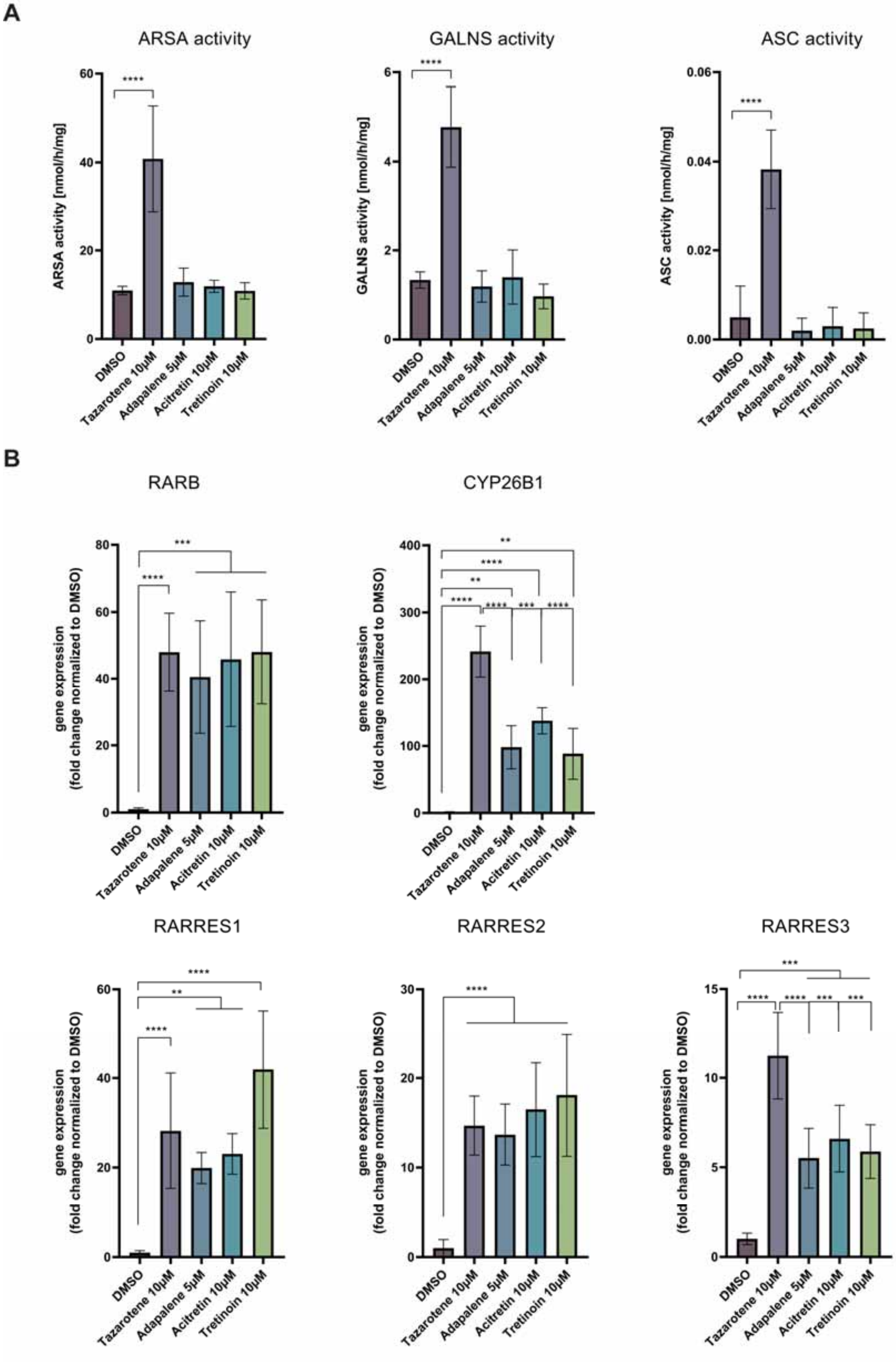

Appendix figure S9

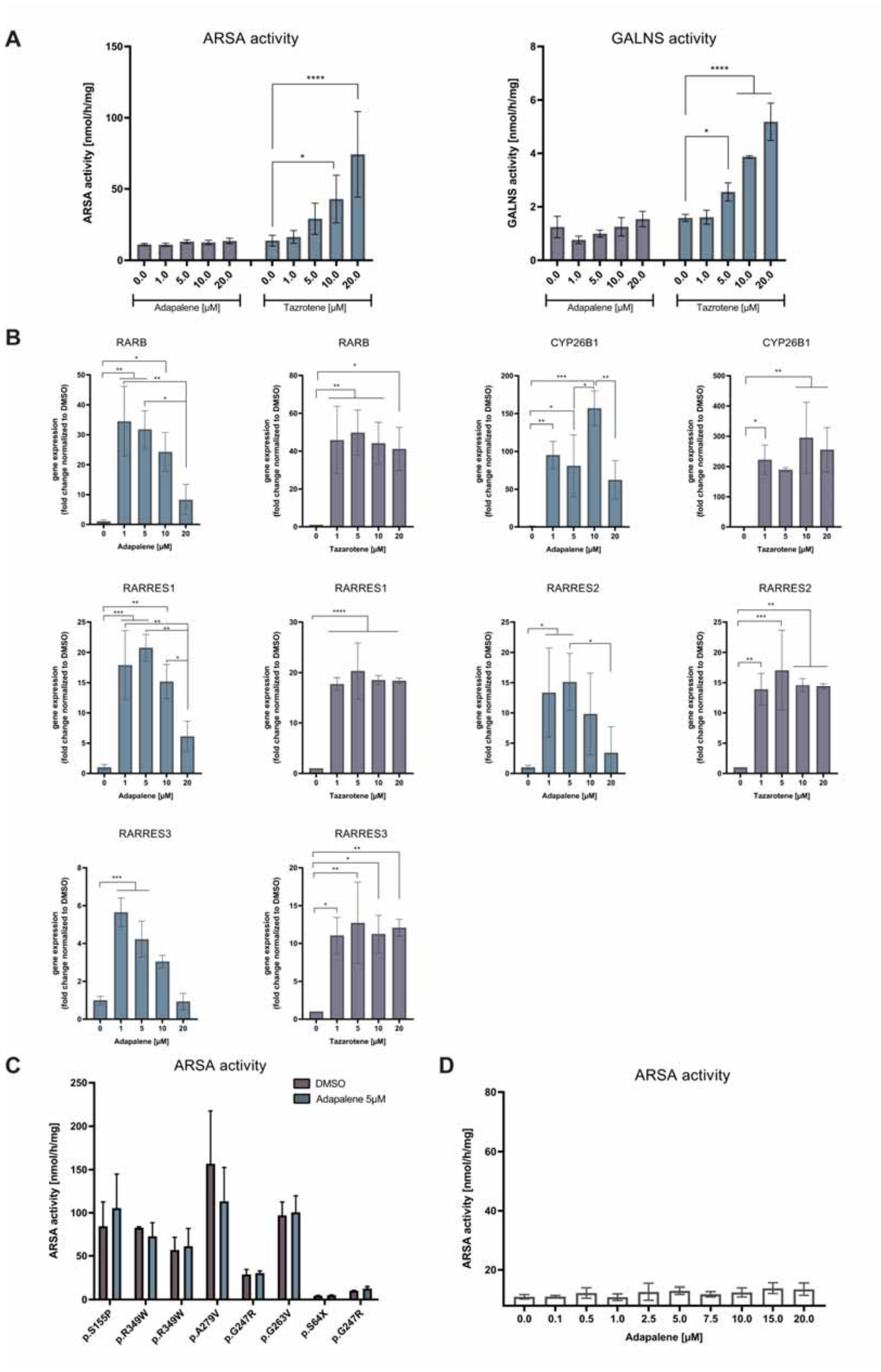

Appendix figure S10

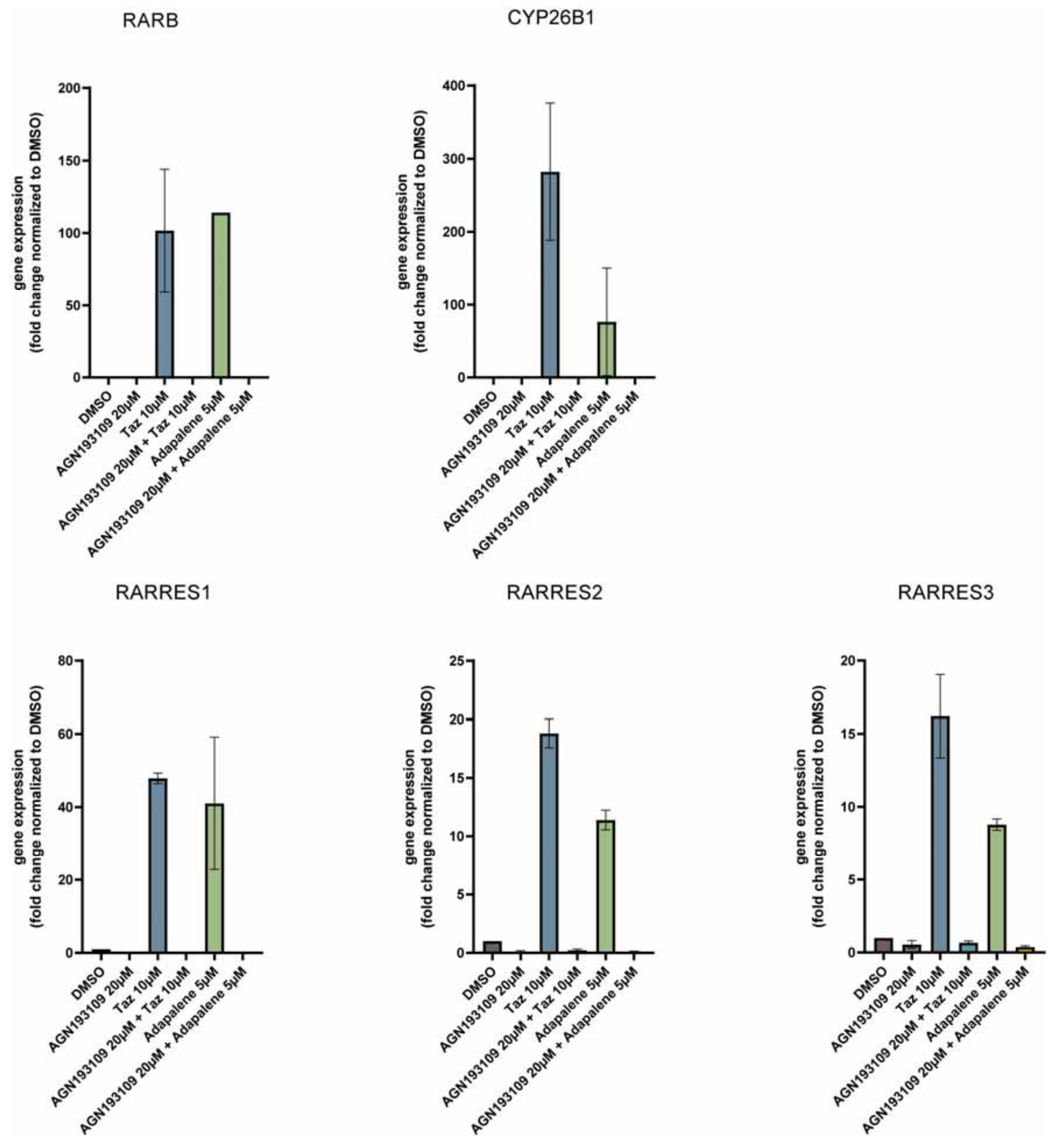

Appendix figure S11

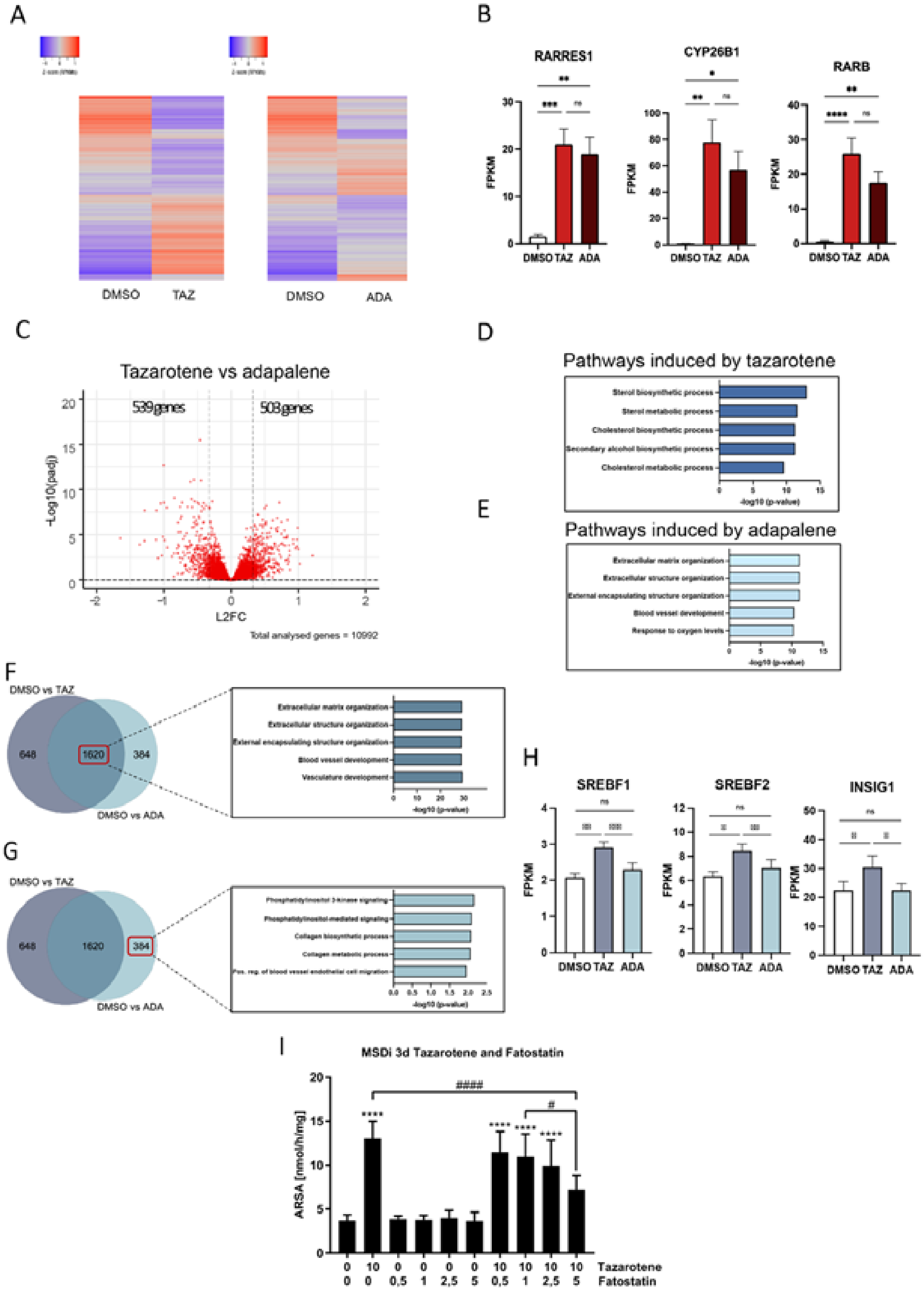

Appendix figure S12

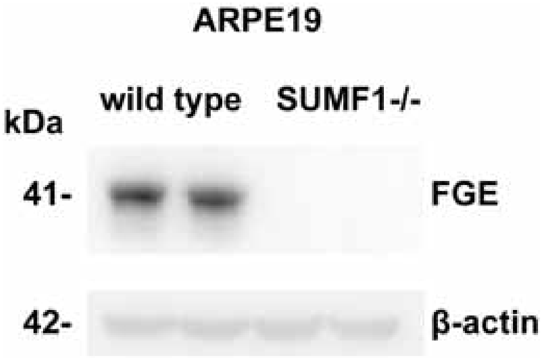

Appendix figure S13

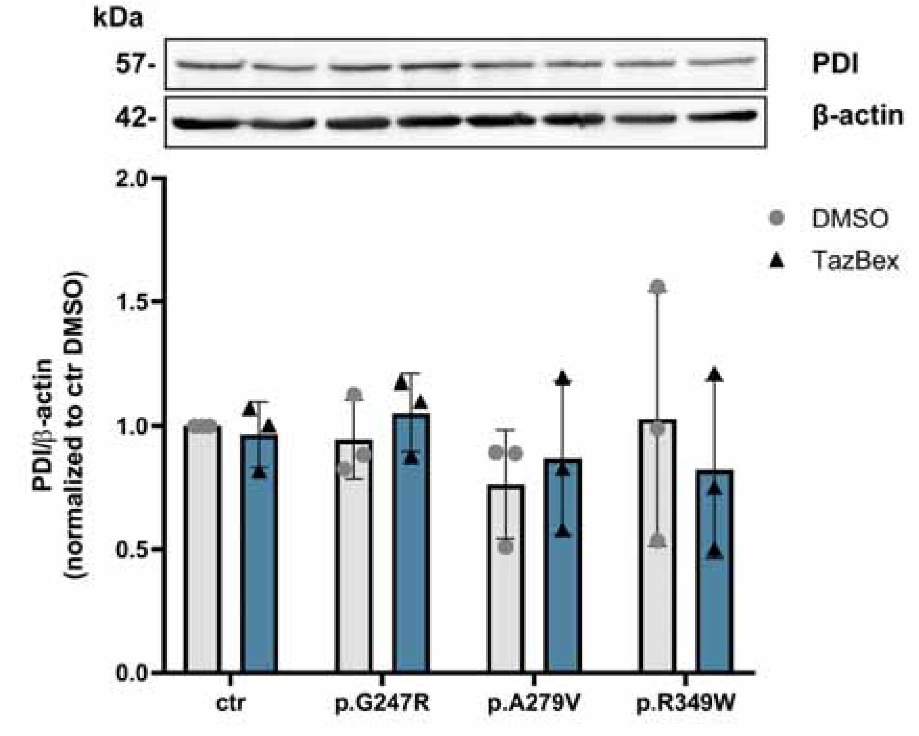

Appendix figure S14

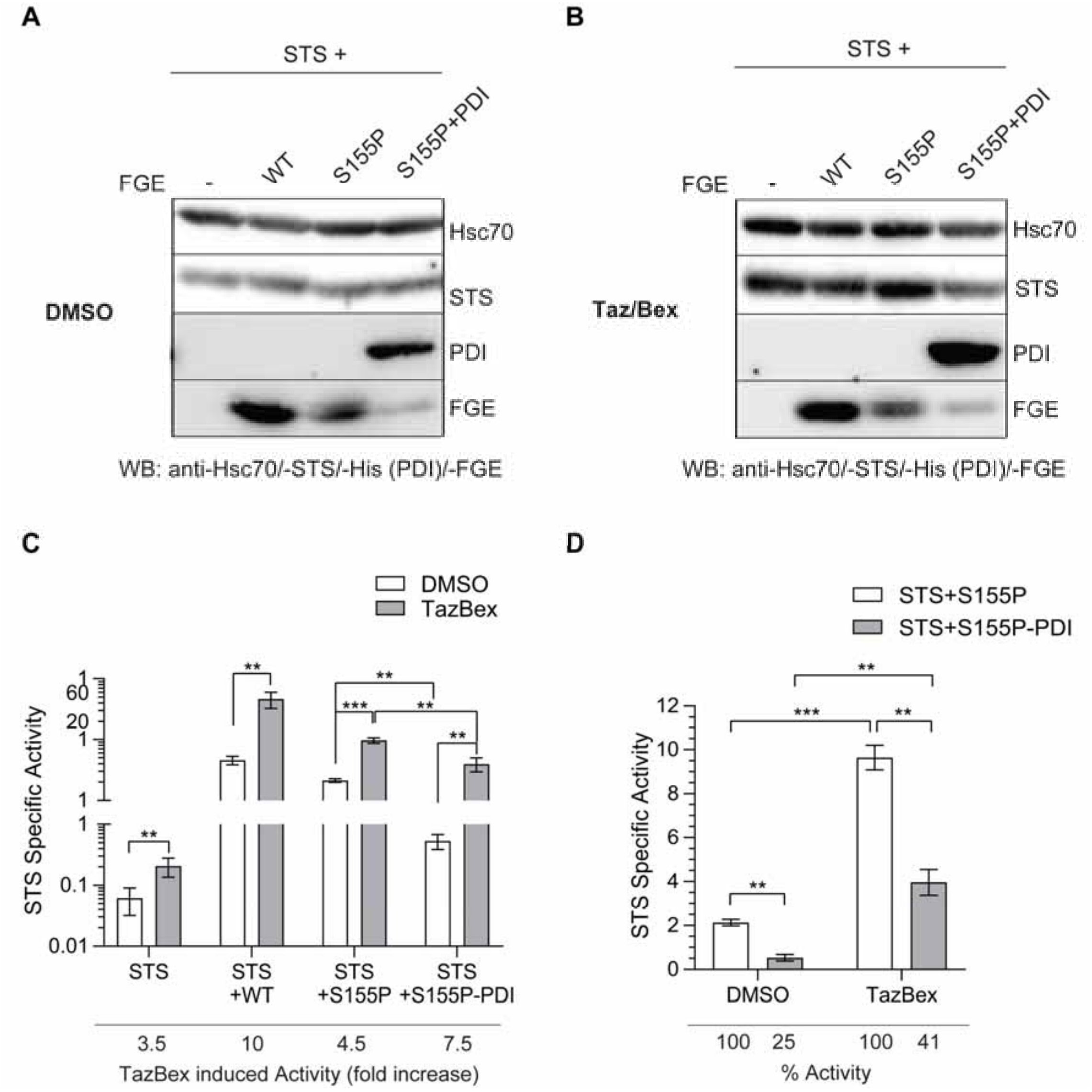

Appendix supplementary figure legends

Appendix figure S1: FDA approved drug library screen on MSD cells – plate layout, test plate and screening results plates 1 and 2.

(**A**) Plate layout for the 96-well ARSA screening assay. (**B**) Representative plot of a screening plate with DMSO only treatment. ARSA activity is displayed as OD (y-axis) in every well as circle (x-axis). The grey colored area represents the upper and lower limit of baseline ARSA activity. N = 1 plate with 84 independent ARSA activity assays. (**C, D**) Original plots from screening a library of 785 licensed drugs with 80 drugs per plate at a final concentration of 10 μM in DMSO (DMSO concentration 1% per well) and incubation for 48 hours. Drugs that exceeded the upper OD limit of baseline were annotated as hits. N = 1 experiment per well.

Appendix figure S2: FDA approved drug library screen on MSD cells – screening results plates 3-6.

(**A-D**) Original plots from screening a library of 785 licensed drugs with 80 drugs per plate at a final concentration of 10 μM in DMSO (DMSO concentration 1% per well) and incubation for 48 hours. Drugs that exceeded the upper OD limit of baseline were annotated as hits. N = 1 experiment per well.

Appendix figure S3: FDA approved drug library screen on MSD cells – screening results plates 7-10.

(**A-D**) Original plots from screening a library of 785 licensed drugs with 80 drugs per plate at a final concentration of 10 μM in DMSO (DMSO concentration 1% per well) and incubation

for 48 hours. Drugs that exceeded the upper OD limit of baseline were annotated as hits. N = 1 experiment per well.

Appendix figure S4: FDA approved drug library screen on MSD cells – rescreening and counterscreen plates 11-13

(**A**) Original plot from rescreening of drugs with ODs below the lower activity range in the initial screen because of toxicity. Cells were incubated at 1 µM final concentration of each drug for 48 hours. N = 1 experiment per well. (**B**) Original plot from rescreening of drugs with ODs below the lower activity range in the initial screen because of toxicity. Cells were incubated at 0,1 µM final concentration of each drug for 48 hours. N = 1 experiment per well. (**C**) Original plot from counter screening of hit drugs at a concentration of 10 µM per well (1% DMSO content) for 48 hours devoid of cells. (N= 1 experiment per well).

Appendix figure S5: Comparison of cell count, total protein content, and ARSA activity changes over time in MSD and control fibroblasts under treatment and control conditions

(**A**) Manual cell count of MSD primary fibroblasts (FGE p.Gly247Arg) plated out at day 0 at standard cell quantities (medium) and additional low and high cell quantitiy. Cells were grown for 3, 6, and 9 days in presence of 0.1% (v/v) DMSO or at standard treatment conditions (medium cell quantity and 10 µM tazarotene, 20 µM bexarotene or 10/20 µM tazarotene/bexarotene, respectively). (**B**) Manual cell count of control fibroblasts plated out at day 0 at standard cell quantities (medium) and additional low and high cell quantitiy. Cells were grown for 3, 6, and 9 days in presence of DMSO or at standard treatment conditions (medium cell quantity and 10 µM tazarotene, 20 µM bexarotene or 10/20 µM tazarotene/bexarotene, respectively). (**C**) Total protein amount of MSD primary fibroblasts

(FGE p.Gly247Arg) plated out at day 0 at standard cell quantities (medium) and additional low and high cell quantitiy. Cells were grown for 3, 6, and 9 days in presence of DMSO or at standard treatment conditions (medium cell quantity and 10 µM tazarotene, 20 µM bexarotene or 10/20 µM tazarotene/bexarotene, respectively). (**D**) Total protein amount of control fibroblasts plated out at day 0 at standard cell quantities (medium) and additional low and high cell quantitiy. Cells were grown for 3, 6, and 9 days in presence of DMSO or at standard treatment conditions (medium cell quantity and 10 µM tazarotene, 20 µM bexarotene or 10/20 µM tazarotene/bexarotene, respectively). (**E**) ARSA activity in MSD primary fibroblasts (FGE p.Gly247Arg) plated out at day 0 at standard cell quantities (medium) and additional low and high cell quantitiy. Cells were grown for 3, 6, and 9 days in presence of DMSO or at standard treatment conditions (medium cell quantity and 10 µM tazarotene, 20 µM bexarotene or 10/20 µM tazarotene/bexarotene, respectively). (**F**) ARSA activity in control fibroblasts plated out at day 0 at standard cell quantities (medium) and additional low and high cell quantitiy. Cells were grown for 3, 6, and 9 days in presence of DMSO or at standard treatment conditions (medium cell quantity middle and 10 µM tazarotene, 20 µM bexarotene or 10/20 µM tazarotene/bexarotene, respectively). All data represent mean ±SD of 5-6 independent experiments. Two-way ANOVA followed by Tukey's test for multiple comparisons. Panel (A-E): Difference of tazarotene/bexarotene treatment against 0/0 µM treatment at the same time point, * $p<0.05$, ** $p<0.01$, *** $p<0.001$, **** $p<0.0001$. Panel F: Difference of DMSO low at day 9 against DMSO low at day 0. ** $p<0.01$, *** $p<0.001$, #### $p<0.0001$. Please see Appendix tables S41-S46 for full list of statistics of all comparisons (significant differences only).

Appendix figure S6: Cell proliferation assessed by XTT-assay

(**A**) Quantification of cell proliferation of MSD primary fibroblasts (variant FGE Gly247Arg homozygous) and control fibroblasts after 6 days of treatment with tazarotene, bexarotene, and tazrotene/bexarotene assessed by XTT assay (see materials and methods). Data represent mean ±SD of 8-20 independent experiments displayed as percentage of DMSO treated cells (negative control, 100% cell proliferation). One-way ANOVA followed by Tukey's test for multiple comparisons. * $p<0.05$. (**B**) Quantification of cell proliferation of MSD primary fibroblasts (variant FGE Gly247Arg homozygous) and control fibroblasts after 6 days of simultaneous treatment with increasing concentrations of tazarotene and bexarotene in a fixed combination of 1:2 assessed by XTT assay (see materials and methods). (**C,D**) Raw OD data without normalization to DMSO treatment for panel A and B, respectively. Statistical analysis was performed on normalized data only. All data represent mean ±SD of 8-12 independent experiments displayed as percentage of DMSO treated cells (negative control, 100% cell proliferation). One-way ANOVA followed by Tukey's test for multiple comparisons. * $p<0.05$, ** $p<0.01$. (**I**)

Appendix figure S7: MSD iPSC line characterization.

(**A**) Representative RT-PCR plot confirming the clearance of Sendai viral vectors in MSD4 iPSC at passages 4-5 by RT-PCR. (**B**) Representative PCR products using primer sets detecting short tandem repeat (STR) sequences from DNA fingerprinting of MSD patient derived iPSCs in comparison to PBMCs used for reprogramming. (**C**) Representative pictures of sequence analysis of *SUMF1* mutations in patient PBMCs and iPSC line. (**D**) Representative picture of G-band analysis for karyotyping MSD patient derived iPSCs (MSD4 p14). Normal karyotype (46,XY). (**E**) Representative flow cytometry plots of the expression of the stem cell surface markerson MSD4 iPSCs. SSEA3/4 and Tra160/81 were all

>95%. , as shown by flow cytometry. (**F**) Representative gel of PCR testing for mycoplasma infection in MSD4 cells and mock, positive and negative controls, respectively.

Appendix figure S8: Sulfatase activities and transcriptional response of retinoids targets in MSD fibroblasts

(A) Treatment of MSD primary fibroblasts (FGE p.Gly247Arg) for six days with 10 µM final concentration of four different retinoids. Only tazarotene significantly increased ARSA, GALNS, and ASC (steroidsulfatase) activities. Data represent mean ±SD of 3 independent experiments. One-way ANOVA followed by Tukey's test for multiple comparisons. Difference against DMSO control: **** $p<0.0001$. (**B**) Transcriptional response (gene expression) as determined by RT-PCR of selected targets of retinoids in MSD primary fibroblasts (FGE p.Gly247Arg) for six days with 10 µM final concentration of four different retinoids. Data represent mean ±SD of 3 independent experiments. One-way ANOVA followed by Tukey's test for multiple comparisons. ** $p<0.01$, *** $p<0.001$, **** $p<0.0001$,

Appendix figure S9: Sulfatase activities and retinoid target gene expression upon different doses of adapalene and treatment response in different MSD primary fibroblast lines.

(A) Treatment of MSD primary fibroblasts (FGE p.Gly247Arg) for six days with differeet concentrations of adapalene and tazarotene as control. Adapalene did not increase ARSA and GALNS activities. Data represent mean ±SD of 3 independent experiments. One-way ANOVA followed by Tukey's test for multiple comparisons. Difference against DMSO control: * $p<0.05$, **** $p<0.0001$. **(B)** Retinoid target gene expression in MSD primary fibroblasts (FGE p.Gly247Arg) after six days treatment with increasing concentrations of

adapalene and tazarotene. Adapalene was able to increase retinoid target gene expression. Data represent mean ±SD of 3 independent experiments. One-way ANOVA followed by Tukey's test for multiple comparisons. Difference against DMSO control: * p<0.05, *** p<0.01, *** p< 0.01, **** p<0.0001. **(C)** Treatment of eight MSD primary fibroblasts lines for six days with 5 μM adapalene. Adapalene did not increase ARSA activity in either cell line. Data represent mean ±SD of 3 independent experiments. Unpaired t-test. **(D)** Refined dose analysis of adapalene treatment in MSD primary fibroblasts (FGE p.Gly247Arg) for six days. Adapalene concentrations between 100 nM and 20 μM did not increase ARSA activity. Data represent mean ±SD of 3 independent experiments. One-way ANOVA followed by Tukey's test for multiple comparisons.

Appendix figure S10: Adapalene increased retinoid target gene expression is mediated via RAR receptors.

24 hours pre-treatment of of MSD primary fibroblasts (FGE p.Gly247Arg) with the pan-RAR receptor antagonist AGN193109 followed by treatment with 10 μM tazarotene and 5 μM adaplane, respectively, for 72 hours. Retinoid target gene expression was analysed by RT-PCR. AGN193109 treatment abrogated tazarotene and adapalene induced gene expression. Data represent mean ±SD of 3 independent experiments. RARB and adapalene control n=1 experiment.

Appendix figure S11: Total RNAseq and differential gene expression from MSD fibroblast lines treated with tazarotene, adapalene, and DMSO.

(**A**) Heatmaps illustrating differences in gene expression between seven different MSD fibroblast lines treated with tazarotene, adapalene, and DMSOin triplicates, respectively, and

analysed by total RNA sequencing (RNAseq). Differential gene expresion was analysed versus treatment condition and DMSO, respectively. (**B**) Expression levels indicated as fragments per kilobase million (FPKM) of retinioid response markers in all cell lines and treatment conditions. Data represent mean from one total RNAseq analysis in triplicates. Paired t-test. Difference against DMSO control: * $p<0.05$, *** $p<0.01$, *** $p< 0.01$, **** $p<0.0001$. (**C**) Transcriptomic changes of tazarotene and adapalene terated samples displayed as volcano plot and number of up- and down-regulated genes (tazarotene versus adapalene). (**D,E**) GO pathway analysis of genes differentially regulated after comparison of tazarotene and adapalene treatment and log10 value of p-values. (**F,G**) Venn diagram and number of exclusively regulated genes for tazarotene treatment (TAZ) versus DMSO condition (left) and adapalene treatment (ADA) versus DMSO (right) as well as number of overlapping genes identically regulated by both tazarotene and adapalene. (**F**) Subsequent GO pathway analysis and log10 value of p-values for identically regulated genes. (**G**) GO pathway analysis and log10 value of p-values for adapalene only regulated genes. (**H**) Expression levels indicated as fragments per kilobase million (FPKM) of tazarotene only regulated genes in all cell lines. Data represent mean from one total RNAseq analysis in triplicates. Paired t-test. Difference against DMSO control: * $p<0.05$, ** $p<0.01$, *** $p< 0.01$. (**I**) Quantification of ARSA activities in ARSA activity quantification after treatment of MSDi cells with increasing concentrations of fatostatin with and without simultaneous treatment of 10 µM tazarotene for 3 days. Data represent mean ±SD of 4 independent experiments. One-way ANOVA followed by Tukey's test for multiple comparisons. Displayed are significance levels for significant differences between tazarotene control and adjacent concentrations of combined tazarotene and fatostatin treatment. # $p<0.05$, #### $p<0.0001$. Difference against 0/0 µM control: **** $p<0.0001$.

Appendix figure S12: SUMF1 -/- cell line evaluation

Representative western blot pictures of CRISPR/Cas9 generated ARPE19 SUMF1 -/- cells and ARPE19 wild type control cells. No FGE-expression at 41 kDa in knock-out cells using an anti-FGE antibody. Detection of β-actin served as loading control.

Appendix figure S13: PDI protein expression upon tazarotene/bexarotene treatment in MSD cell lines

Representative western blot pictures and quantification of PDI protein expression in three MSD primary fibroblast cell lines and one control fibroblast line treated with tazarotene 10 μM and bexarotene 20 μM for six days compared to DMSO treated cells. Detection of β-actin served as loading control. Data represent mean ±SD of 3 independent experiments. One-way ANOVA followed by Tukey's test for multiple comparisons. No statistical significant differences were detected.

Appendix figure S14: Expression of PDI mitigates tazarotene/bexarotene-mediated effect of sulfatase activation.

(A,B) MSDi cells were treated with either DMSO or tazarotene 10 μM and bexarotene 20 μM for two days and transiently co-transfected with pBI-plasmids that express either steroid sulfatase (STS) alone or together with FGE-WT (wildtype), FGE-Ser155Pro variant or FGE-Ser155Pro and His-tagged PDI from a bi-directional doxycycline inducible promotor. 4h post-transfection, expression was induced with 1 μg/ml doxycycline for 24 h and cells harvested. Of note, either DMSO or tazarotene/bexarotene were present in all steps till the cells were harvested. STS activity assay was performed in cell lysates and equal amount of total protein from all lysates were resolved in SDS-PAGE and western blot probed with anti-STS, anti-FGE, anti-His and anti-Hsc70 (as loading control). The panels show a representative result from one experiment. (**C,D**) STS specific activity calculated based on quantification of

western blot signals that correspond to STS (after normalization to that of Hsc70). N=3 independet experiments, error bars represent mean ±SEM. One-way ANOVA followed by Tukey’s test for multiple comparisons. ** $p<0.01$, *** $p<0.001$.

Appendix supplementary tables

Appendix table S1: List of drugs, suppliers and catalog numbers

Drug	Supplier	Cat. No.
Vorinostat	Sigma-Aldrich	SML0061
Clindamycine	Sigma-Aldrich	C5269
Asenapine	Sigma-Aldrich	A7861
Tazarotene	Sigma-Aldrich	T7080
Vitamine A	Sigma-Aldrich	7235-407
9-cis retinoic acid	Sigma-Aldrich	R4643
Isotretinoin	Sigma-Aldrich	R3255
Acitretin	Sigma-Aldrich	44707
Etretinate	Toronto Research Chemicals	E938000
Adapalene	Sigma-Aldrich	A7486
Bexarotene	Sigma-Aldrich	SML0282
Tazarotenic acid	Sigma-Aldrich	SML1619
Fatostatin	Sigma-Aldrich	341329
AGN 193109	Tocris	5758
HX 531	Sigma-Aldrich	SML2170
DMSO	Serva	39757

Appendix table S2: RT-PCR primers and primer sequences (5’>3’)

beta-actin	Fwd	TGACCCAGATCATGTTTGAG
beta-actin	Rev	ATCACGATGCCAGTGGTA
RARB	Fwd	GTCACCGAGATAAGAACTGTGTTA
RARB	Rev	ACTCAGCTGTCATTTCATAGCTCTC
CYP26B1	Fwd	TGGACCTCCTCATTGAGAGCA
CYP26B1	Rev	GGCATAGGCCGCAAAGATCA
RARRES1	Fwd	AAACCCCTTGGAAATAGTCAGC
RARRES1	Rev	GGAAAGCCAAATCCCAGATGAG
RARRES2	Fwd	AGAAACCCGAGTGCAAAGTCA
RARRES2	Rev	AGAAACCCGAGTGCAAAGTCA
RARRES3	Fwd	AACAGTGCAGAGGTGAAACGG
RARRES3	Rev	GTTGGTACTCATGGTCCAAGC

Appendix table S3: Mean values and significance levels for Figure 1 panel B (significant differences only)

**Comparison**	**Mean 1**	**Mean 2**	**Summary**	**Adjusted P Value**
neg. contr. vs. Tazarotene	6,917	14,72	**	0,0016
neg. contr. vs. pos. contr.	6,917	40,27	****	<0,0001
Tazarotene vs. Clindamycin	14,72	7,814	**	0,0064
Tazarotene vs. Vorinostat	14,72	6,957	**	0,0017
Tazarotene vs. pos. contr.	14,72	40,27	****	<0,0001
Clindamycin vs. pos. contr.	7,814	40,27	****	<0,0001
Vorinostat vs. pos. contr.	6,957	40,27	****	<0,0001
Asenapine vs. pos. contr.	9,617	40,27	****	<0,0001

Appendix table S4: Mean values and significance levels for Figure 1 panel C (significant differences only)

**Comparison**	**Mean 1**	**Mean 2**	**Summary**	**Adjusted P Value**
0 vs. 2	2,511	5,895	**	0,0076
0 vs. 5	2,511	8,759	****	<0,0001
0 vs. 10	2,511	9,942	****	<0,0001
0 vs. 15	2,511	12,67	****	<0,0001
0 vs. 25	2,511	13,48	****	<0,0001
0 vs. 50	2,511	12,66	****	<0,0001
2 vs. 5	5,895	8,759	*	0,0474
2 vs. 10	5,895	9,942	***	0,001
2 vs. 15	5,895	12,67	****	<0,0001
2 vs. 25	5,895	13,48	****	<0,0001
2 vs. 50	5,895	12,66	****	<0,0001
5 vs. 15	8,759	12,67	**	0,0071
5 vs. 25	8,759	13,48	***	0,0008
5 vs. 50	8,759	12,66	**	0,0074
10 vs. 25	9,942	13,48	*	0,0136

Appendix table S5: Mean values and significance levels for Figure 1 panel D (significant differences only)

**Comparison**	**Mean 1**	**Mean 2**	**Summary**	**Adjusted P Value**

0 vs. 5	4,121	9,026	**	0,0014
0 vs. 10	4,121	9,185	***	0,0009
0 vs. 15	4,121	9,998	***	0,0002
0 vs. 20	4,121	11,02	****	<0,0001
0 vs. 25	4,121	11,19	****	<0,0001
0 vs. 50	4,121	9,775	***	0,0003

Appendix table S6: Mean values and significance levels for Figure 1 panel E (significant differences only)

**Comparison**	**Mean 1**	**Mean 2**	**Summary**	**Adjusted P Value**
DMSO vs. Isotretinoin	3,303	5,667	**	0,0031
DMSO vs. Tazarotene	3,303	8,72	****	<0,0001
DMSO vs. Bexarotene	3,303	6,2	***	0,0003
DMSO vs. Tazarotenic acid	3,303	5,577	**	0,0046
Vitamin A vs. Etretinate	4,797	2,41	**	0,0028
Vitamin A vs. Tazarotene	4,797	8,72	****	<0,0001
9-cis-retinoic acid vs. Etretinate	4,893	2,41	**	0,0018
9-cis-retinoic acid vs. Tazarotene	4,893	8,72	****	<0,0001
Isotretinoin vs. Etretinate	5,667	2,41	****	<0,0001
Isotretinoin vs. Adapalene 1μM	5,667	3,33	**	0,0035
Isotretinoin vs. Tazarotene	5,667	8,72	***	0,0001
Acitretin vs. Tazarotene	3,963	8,72	****	<0,0001
Acitretin vs. Bexarotene	3,963	6,2	**	0,0054
Etretinate vs. Tazarotene	2,41	8,72	****	<0,0001
Etretinate vs. Bexarotene	2,41	6,2	****	<0,0001
Etretinate vs. Tazarotenic acid	2,41	5,577	****	<0,0001
Adapalene 1μM vs. Tazarotene	3,33	8,72	****	<0,0001
Adapalene 1μM vs. Bexarotene	3,33	6,2	***	0,0003
Adapalene 1μM vs. Tazarotenic acid	3,33	5,577	**	0,0052
Tazarotene vs. Bexarotene	8,72	6,2	**	0,0015
Tazarotene vs. Tazarotenic acid	8,72	5,577	****	<0,0001

Appendix table S7: Mean values and significance levels for Figure 1 panel F (significant differences only)

**Comparison**	**Mean 1**	**Mean 2**	**Summary**	**Adjusted P Value**
0 vs. 5	2,633	5,175	*	0,0253
0 vs. 10	2,633	6,451	****	<0,0001
0 vs. 20	2,633	7,802	****	<0,0001

0 vs. 50	2,633	7,067	***	0,0008
1 vs. 10	3,283	6,451	*	0,0307
1 vs. 20	3,283	7,802	***	0,0006
1 vs. 50	3,283	7,067	*	0,0416
2 vs. 20	3,75	7,802	**	0,0025
5 vs. 20	5,175	7,802	*	0,0189

Appendix table S8: Mean values and significance levels for Figure 1 panel G (significant differences only)

Comparison	Mean 1	Mean 2	Summary	Adjusted P Value
0/0 vs 1/2	4,515	12,56	****	<0,0001
0/0 vs 2/4	4,515	15,88	****	<0,0001
0/0 vs 5/10	4,515	19,9	****	<0,0001
0/0 vs 7.5/15	4,515	20,98	****	<0,0001
0/0 vs 10/20	4,515	19,65	****	<0,0001
0/0 vs 15/30	4,515	14	****	<0,0001
1/2 vs 2/4	12,56	15,88	***	0,0003
1/2 vs 5/10	12,56	19,9	****	<0,0001
1/2 vs 7.5/15	12,56	20,98	****	<0,0001
1/2 vs 10/20	12,56	19,65	****	<0,0001
2/4 vs 5/10	15,88	19,9	****	<0,0001
2/4 vs 7.5/15	15,88	20,98	****	<0,0001
2/4 vs 10/20	15,88	19,65	***	0,0007
5/10 vs 15/30	19,9	14	****	<0,0001
7.5/15 vs 15/30	20,98	14	****	<0,0001
10/20 vs 15/30	19,65	14	****	<0,0001

Appendix table S9: Mean values and significance levels for Figure 1 panel H (significant differences only)

Comparison	Mean 1	Mean 2	Summary	Adjusted P Value
0 vs. 2	3,415	8,71	*	0,0196
0 vs. 3	3,415	14,29	****	<0,0001
0 vs. 6	3,415	20,06	****	<0,0001
0 vs. 9	3,415	25,76	****	<0,0001
0 vs. 12	3,415	15,18	****	<0,0001
2 vs. 3	8,71	14,29	*	0,0123
2 vs. 6	8,71	20,06	****	<0,0001
2 vs. 9	8,71	25,76	****	<0,0001

2 vs. 12	8,71	15,18	**	0,0081
3 vs. 6	14,29	20,06	***	0,0002
3 vs. 9	14,29	25,76	****	<0,0001
6 vs. 9	20,06	25,76	*	0,0132
6 vs. 12	20,06	15,18	*	0,011
9 vs. 12	25,76	15,18	****	<0,0001

Appendix table S10: Mean values and significance levels for Figure 2 panel A (significant differences only)

**Comparison**	**Mean 1**	**Mean 2**	**Summary**	**Adjusted P Value**
0 vs. 5	15,9	30,45	***	0,001
0 vs. 10	15,9	47,89	****	<0,0001
0 vs. 25	15,9	78	****	<0,0001
0 vs. 50	15,9	94,45	****	<0,0001
0 vs. 75	15,9	84,8	****	<0,0001
0 vs. 100	15,9	65,39	****	<0,0001
1 vs. 10	18,91	47,89	****	<0,0001
1 vs. 25	18,91	78	****	<0,0001
1 vs. 50	18,91	94,45	****	<0,0001
1 vs. 75	18,91	84,8	****	<0,0001
1 vs. 100	18,91	65,39	****	<0,0001
2.5 vs. 10	22,34	47,89	****	<0,0001
2.5 vs. 25	22,34	78	****	<0,0001
2.5 vs. 50	22,34	94,45	****	<0,0001
2.5 vs. 75	22,34	84,8	****	<0,0001
2.5 vs. 100	22,34	65,39	****	<0,0001
5 vs. 10	30,45	47,89	****	<0,0001
5 vs. 25	30,45	78	****	<0,0001
5 vs. 50	30,45	94,45	****	<0,0001
5 vs. 75	30,45	84,8	****	<0,0001
5 vs. 100	30,45	65,39	****	<0,0001
10 vs. 25	47,89	78	****	<0,0001
10 vs. 50	47,89	94,45	****	<0,0001
10 vs. 75	47,89	84,8	****	<0,0001
10 vs. 100	47,89	65,39	****	<0,0001
25 vs. 50	78	94,45	***	0,0009
25 vs. 100	78	65,39	*	0,0193
50 vs. 100	94,45	65,39	****	<0,0001
75 vs. 100	84,8	65,39	***	0,0003

Appendix table S11: Mean values and significance levels for Figure 2 panel B (significant differences only)

**Comparison**	**Mean 1**	**Mean 2**	**Summary**	**Adjusted P Value**
0 vs. 10	14,84	22,17	*	0,0112
0 vs. 20	14,84	22,48	*	0,0156
0 vs. 25	14,84	26,6	****	<0,0001
0 vs. 50	14,84	30,58	****	<0,0001
2.5 vs. 50	20,2	30,58	**	0,0032
2.5 vs. 75	20,2	7,603	**	0,0017
5 vs. 25	19,37	26,6	**	0,0018
5 vs. 50	19,37	30,58	****	<0,0001
5 vs. 75	19,37	7,603	***	0,0002
10 vs. 50	22,17	30,58	**	0,0061
10 vs. 75	22,17	7,603	****	<0,0001
20 vs. 50	22,48	30,58	*	0,0177
20 vs. 75	22,48	7,603	****	<0,0001
25 vs. 75	26,6	7,603	****	<0,0001
50 vs. 75	30,58	7,603	****	<0,0001

Appendix table S12: Mean values and significance levels for Figure 2 panel C (significant differences only)

**Comparison**	**Mean 1**	**Mean 2**	**Summary**	**Adjusted P Value**
0/0 vs 2/5	15,59	32,75	**	0,0016
0/0 vs 5/10	15,59	64,17	****	<0,0001
0/0 vs 7/5	15,59	103,1	****	<0,0001
0/0 vs 10/20	15,59	111	****	<0,0001
0/0 vs 15/30	15,59	73,08	****	<0,0001
0.1/0.2 vs 2.5/5	17,28	32,75	*	0,021
0.1/0.2 vs 5/10	17,28	64,17	****	<0,0001
0.1/0.2 vs 7.5/10	17,28	103,1	****	<0,0001
0.1/0.2 vs 10/20	17,28	111	****	<0,0001
0.1/0.2 vs 15/30	17,28	73,08	****	<0,0001
1/2 vs 5/10	22,83	64,17	****	<0,0001
1/2 vs 7/5	22,83	103,1	****	<0,0001
1/2 vs 10/20	22,83	111	****	<0,0001
1/2 vs 15/30	22,83	73,08	****	<0,0001
2.5/5 vs 5/10	32,75	64,17	****	<0,0001
2.5/5 vs 7.5/15	32,75	103,1	****	<0,0001
2.5/5 vs 10/20	32,75	111	****	<0,0001
2.5/5 vs 15/30	32,75	73,08	****	<0,0001
5/10 vs 7/5	64,17	103,1	****	<0,0001

5/10 vs 10/20	64,17	111	****	<0,0001
7.5/15 vs 15/30	103,1	73,08	****	<0,0001
10/20 vs 15/30	111	73,08	****	<0,0001

Appendix table S13: Mean values and significance levels for Figure 2 panel D (significant differences only)

**Comparison**	**Mean 1**	**Mean 2**	**Summary**	**Adjusted P Value**
0 vs. 3	16,09	43,1	*	0,0443
0 vs. 6	16,09	109,5	****	<0,0001
0 vs. 9	16,09	175,8	****	<0,0001
0 vs. 14	16,09	181,2	****	<0,0001
0 vs. 21	16,09	168,3	****	<0,0001
3 vs. 6	43,1	109,5	****	<0,0001
3 vs. 9	43,1	175,8	****	<0,0001
3 vs. 14	43,1	181,2	****	<0,0001
3 vs. 21	43,1	168,3	****	<0,0001
6 vs. 9	109,5	175,8	****	<0,0001
6 vs. 14	109,5	181,2	****	<0,0001
6 vs. 21	109,5	168,3	***	0,0001

Appendix table S14: Mean values and significance levels for Figure 2 panel E (significant differences only)

**Comparison**	**Mean 1**	**Mean 2**	**Summary**	**Adjusted P Value**
ARSA - vs. +	17,06	109,5	****	<0,0001
ARSB - vs. +	18,09	46,96	***	0,0001
GALNS - vs. +	3,54	11,67	***	0,0003
STS - vs. +	14,8	113,6	****	<0,0001

Appendix table S15: Mean values and significance levels for Figure 2 panel F (significant differences only)

**Comparison**	**Mean 1**	**Mean 2**	**Summary**	**Adjusted P Value**
p.G247R - vs. +	17,71	98,09	****	<0,0001
p.G263V - vs. +	131,2	324,5	****	<0,0001
p.A279V - vs. +	45	88	***	0,0005

p.R349W - vs. +	27,89	97,87	****	<0,0001

Appendix table S16: Mean values and significance levels for Figure 2 panel G (significant differences only)

**Comparison**	**Mean 1**	**Mean 2**	**Summary**	**Adjusted P Value**
contr. - vs. +	30,38	35,02	****	<0,0001
MSD - vs. +	10,97	17,57	****	<0,0001

Appendix table S17: Mean values and significance levels for Figure 2 panel H (significant differences only)

**Comparison**	**Mean 1**	**Mean 2**	**Summary**	**Adjusted P Value**
contr. - vs. +	2,287	1,78	*	0,0303
MSD - vs. +	0,219	0,4617	***	0,0002

Appendix table S18: Mean values and significance levels for Figure 3 panel C (significant differences only)

**Comparison**	**Mean 1**	**Mean 2**	**Summary**	**Adjusted P Value**
ctr dmso vs. MSD dmso	740085758	3733811299	***	0,0002
MSD dmso vs. ctr tazbex	3733811299	785298854	***	0,0002
MSD dmso vs. msd tazbex	3733811299	1912504979	*	0,0115

Appendix table S19: Mean values and significance levels for Figure 3 panel D (significant differences only)

**Comparison**	**Mean 1**	**Mean 2**	**Summary**	**Adjusted P Value**
ctr dmso vs. MSD dmso	740085758	3733811299	***	0,0002
MSD dmso vs. ctr tazbex	3733811299	785298854	***	0,0002
MSD dmso vs. msd tazbex	3733811299	1912504979	*	0,0115

Appendix table S20: Mean values and significance levels for Figure 4 panel A (significant differences only)

**Comparison**	**Mean 1**	**Mean 2**	**Summary**	**Adjusted P Value**
0:DMSO vs. 0:Tazarotene 10 µM	3,263	10,38	****	<0,0001
0:DMSO vs. 0:Tazarotene 10 µM Bexarotene 20µM	3,263	13,89	****	<0,0001
0:DMSO vs. 1:Tazarotene 10 µM	3,263	7,273	****	<0,0001
0:DMSO vs. 1:Tazarotene 10 µM Bexarotene 20µM	3,263	11,18	****	<0,0001
0:DMSO vs. 5:Tazarotene 10 µM	3,263	6,725	****	<0,0001
0:DMSO vs. 5:Tazarotene 10 µM Bexarotene 20µM	3,263	10,16	****	<0,0001
0:DMSO vs. 10:Tazarotene 10 µM	3,263	6,546	****	<0,0001
0:DMSO vs. 10:Tazarotene 10 µM Bexarotene 20µM	3,263	9,414	****	<0,0001
0:DMSO vs. 20:Tazarotene 10 µM Bexarotene 20µM	3,263	6,536	**	0,0021
0:Tazarotene 10 µM vs. 0:Bexarotene 20 µM	10,38	4,74	****	<0,0001
0:Tazarotene 10 µM vs. 0:Tazarotene 10 µM Bexarotene 20µM	10,38	13,89	**	0,0011
0:Tazarotene 10 µM vs. 1:DMSO	10,38	3,151	****	<0,0001
0:Tazarotene 10 µM vs. 1:Tazarotene 10 µM	10,38	7,273	***	0,0004
0:Tazarotene 10 µM vs. 1:Bexarotene 20 µM	10,38	4,493	****	<0,0001
0:Tazarotene 10 µM vs. 5:DMSO	10,38	3,326	****	<0,0001
0:Tazarotene 10 µM vs. 5:Tazarotene 10 µM	10,38	6,725	****	<0,0001
0:Tazarotene 10 µM vs. 5:Bexarotene 20 µM	10,38	4,983	****	<0,0001
0:Tazarotene 10 µM vs. 10:DMSO	10,38	3,378	****	<0,0001
0:Tazarotene 10 µM vs. 10:Tazarotene 10 µM	10,38	6,546	****	<0,0001
0:Tazarotene 10 µM vs. 10:Bexarotene 20 µM	10,38	4,517	****	<0,0001
0:Tazarotene 10 µM vs. 20:DMSO	10,38	4,195	****	<0,0001
0:Tazarotene 10 µM vs. 20:Tazarotene 10 µM	10,38	5,021	****	<0,0001
0:Tazarotene 10 µM vs. 20:Bexarotene 20 µM	10,38	4,693	****	<0,0001
0:Tazarotene 10 µM vs. 20:Tazarotene 10 µM Bexarotene 20µM	10,38	6,536	***	0,0002
0:Bexarotene 20 µM vs. 0:Tazarotene 10 µM Bexarotene 20µM	4,74	13,89	****	<0,0001
0:Bexarotene 20 µM vs. 1:Tazarotene 10 µM Bexarotene 20µM	4,74	11,18	****	<0,0001
0:Bexarotene 20 µM vs. 5:Tazarotene 10 µM Bexarotene 20µM	4,74	10,16	****	<0,0001
0:Bexarotene 20 µM vs. 10:Tazarotene 10 µM Bexarotene 20µM	4,74	9,414	***	0,0008
0:Tazarotene 10 µM Bexarotene 20µM vs. 1:DMSO	13,89	3,151	****	<0,0001

0:Tazarotene 10 µM Bexarotene 20µM vs. 1:Tazarotene 10 µM	13,89	7,273	****	<0,0001
0:Tazarotene 10 µM Bexarotene 20µM vs. 1:Bexarotene 20 µM	13,89	4,493	****	<0,0001
0:Tazarotene 10 µM Bexarotene 20µM vs. 5:DMSO	13,89	3,326	****	<0,0001
0:Tazarotene 10 µM Bexarotene 20µM vs. 5:Tazarotene 10 µM	13,89	6,725	****	<0,0001
0:Tazarotene 10 µM Bexarotene 20µM vs. 5:Bexarotene 20 µM	13,89	4,983	****	<0,0001
0:Tazarotene 10 µM Bexarotene 20µM vs. 5:Tazarotene 10 µM Bexarotene 20µM	13,89	10,16	**	0,0034
0:Tazarotene 10 µM Bexarotene 20µM vs. 10:DMSO	13,89	3,378	****	<0,0001
0:Tazarotene 10 µM Bexarotene 20µM vs. 10:Tazarotene 10 µM	13,89	6,546	****	<0,0001
0:Tazarotene 10 µM Bexarotene 20µM vs. 10:Bexarotene 20 µM	13,89	4,517	****	<0,0001
0:Tazarotene 10 µM Bexarotene 20µM vs. 10:Tazarotene 10 µM Bexarotene 20µM	13,89	9,414	****	<0,0001
0:Tazarotene 10 µM Bexarotene 20µM vs. 20:DMSO	13,89	4,195	****	<0,0001
0:Tazarotene 10 µM Bexarotene 20µM vs. 20:Tazarotene 10 µM	13,89	5,021	****	<0,0001
0:Tazarotene 10 µM Bexarotene 20µM vs. 20:Bexarotene 20 µM	13,89	4,693	****	<0,0001
0:Tazarotene 10 µM Bexarotene 20µM vs. 20:Tazarotene 10 µM Bexarotene 20µM	13,89	6,536	****	<0,0001
1:DMSO vs. 1:Tazarotene 10 µM	3,151	7,273	****	<0,0001
1:DMSO vs. 1:Tazarotene 10 µM Bexarotene 20µM	3,151	11,18	****	<0,0001
1:DMSO vs. 5:Tazarotene 10 µM	3,151	6,725	****	<0,0001
1:DMSO vs. 5:Tazarotene 10 µM Bexarotene 20µM	3,151	10,16	****	<0,0001
1:DMSO vs. 10:Tazarotene 10 µM	3,151	6,546	****	<0,0001
1:DMSO vs. 10:Tazarotene 10 µM Bexarotene 20µM	3,151	9,414	****	<0,0001
1:DMSO vs. 20:Tazarotene 10 µM Bexarotene 20µM	3,151	6,536	**	0,0011
1:Tazarotene 10 µM vs. 1:Tazarotene 10 µM Bexarotene 20µM	7,273	11,18	***	0,0001
1:Tazarotene 10 µM vs. 5:DMSO	7,273	3,326	****	<0,0001
1:Tazarotene 10 µM vs. 5:Tazarotene 10 µM Bexarotene 20µM	7,273	10,16	*	0,0229
1:Tazarotene 10 µM vs. 10:DMSO	7,273	3,378	****	<0,0001

1:Tazarotene 10 µM vs. 20:DMSO	7,273	4,195	***	0,0002
1:Bexarotene 20 µM vs. 1:Tazarotene 10 µM Bexarotene 20µM	4,493	11,18	****	<0,0001
1:Bexarotene 20 µM vs. 5:Tazarotene 10 µM Bexarotene 20µM	4,493	10,16	****	<0,0001
1:Bexarotene 20 µM vs. 10:Tazarotene 10 µM Bexarotene 20µM	4,493	9,414	***	0,0003
1:Tazarotene 10 µM Bexarotene 20µM vs. 5:DMSO	11,18	3,326	****	<0,0001
1:Tazarotene 10 µM Bexarotene 20µM vs. 5:Tazarotene 10 µM	11,18	6,725	****	<0,0001
1:Tazarotene 10 µM Bexarotene 20µM vs. 5:Bexarotene 20 µM	11,18	4,983	****	<0,0001
1:Tazarotene 10 µM Bexarotene 20µM vs. 10:DMSO	11,18	3,378	****	<0,0001
1:Tazarotene 10 µM Bexarotene 20µM vs. 10:Tazarotene 10 µM	11,18	6,546	****	<0,0001
1:Tazarotene 10 µM Bexarotene 20µM vs. 10:Bexarotene 20 µM	11,18	4,517	****	<0,0001
1:Tazarotene 10 µM Bexarotene 20µM vs. 20:DMSO	11,18	4,195	****	<0,0001
1:Tazarotene 10 µM Bexarotene 20µM vs. 20:Tazarotene 10 µM	11,18	5,021	****	<0,0001
1:Tazarotene 10 µM Bexarotene 20µM vs. 20:Bexarotene 20 µM	11,18	4,693	****	<0,0001
1:Tazarotene 10 µM Bexarotene 20µM vs. 20:Tazarotene 10 µM Bexarotene 20µM	11,18	6,536	****	<0,0001
5:DMSO vs. 5:Tazarotene 10 µM	3,326	6,725	****	<0,0001
5:DMSO vs. 5:Tazarotene 10 µM Bexarotene 20µM	3,326	10,16	****	<0,0001
5:DMSO vs. 10:Tazarotene 10 µM	3,326	6,546	****	<0,0001
5:DMSO vs. 10:Tazarotene 10 µM Bexarotene 20µM	3,326	9,414	****	<0,0001
5:DMSO vs. 20:Tazarotene 10 µM Bexarotene 20µM	3,326	6,536	**	0,0029
5:Tazarotene 10 µM vs. 5:Tazarotene 10 µM Bexarotene 20µM	6,725	10,16	**	0,0016
5:Tazarotene 10 µM vs. 10:DMSO	6,725	3,378	****	<0,0001
5:Tazarotene 10 µM vs. 20:DMSO	6,725	4,195	**	0,007
5:Bexarotene 20 µM vs. 5:Tazarotene 10 µM Bexarotene 20µM	4,983	10,16	****	<0,0001
5:Bexarotene 20 µM vs. 10:Tazarotene 10 µM Bexarotene 20µM	4,983	9,414	**	0,0021
5:Tazarotene 10 µM Bexarotene 20µM vs. 10:DMSO	10,16	3,378	****	<0,0001
5:Tazarotene 10 µM Bexarotene 20µM vs. 10:Tazarotene 10 µM	10,16	6,546	***	0,0006

5:Tazarotene 10 µM Bexarotene 20µM vs. 10:Bexarotene 20 µM	10,16	4,517	****	<0,0001
5:Tazarotene 10 µM Bexarotene 20µM vs. 20:DMSO	10,16	4,195	****	<0,0001
5:Tazarotene 10 µM Bexarotene 20µM vs. 20:Tazarotene 10 µM	10,16	5,021	****	<0,0001
5:Tazarotene 10 µM Bexarotene 20µM vs. 20:Bexarotene 20 µM	10,16	4,693	****	<0,0001
5:Tazarotene 10 µM Bexarotene 20µM vs. 20:Tazarotene 10 µM Bexarotene 20µM	10,16	6,536	**	0,0054
10:DMSO vs. 10:Tazarotene 10 µM	3,378	6,546	***	0,0001
10:DMSO vs. 10:Tazarotene 10 µM Bexarotene 20µM	3,378	9,414	****	<0,0001
10:DMSO vs. 20:Tazarotene 10 µM Bexarotene 20µM	3,378	6,536	**	0,0038
10:Tazarotene 10 µM vs. 10:Tazarotene 10 µM Bexarotene 20µM	6,546	9,414	*	0,0244
10:Tazarotene 10 µM vs. 20:DMSO	6,546	4,195	*	0,0197
10:Bexarotene 20 µM vs. 10:Tazarotene 10 µM Bexarotene 20µM	4,517	9,414	***	0,0003
10:Tazarotene 10 µM Bexarotene 20µM vs. 20:DMSO	9,414	4,195	****	<0,0001
10:Tazarotene 10 µM Bexarotene 20µM vs. 20:Tazarotene 10 µM	9,414	5,021	****	<0,0001
10:Tazarotene 10 µM Bexarotene 20µM vs. 20:Bexarotene 20 µM	9,414	4,693	***	0,0006

Appendix table S21: Mean values and significance levels for Figure 4 panel B (significant differences only)

**Comparison**	**Mean 1**	**Mean 2**	**Summary**	**Adjusted P Value**
0:DMSO vs. 0:Tazarotene 10 µM	2,984	11,07	****	<0,0001
0:DMSO vs. 0:Tazarotene 10 µM Bexarotene 20µM	2,984	16,14	****	<0,0001
0:DMSO vs. 1:Tazarotene 10 µM	2,984	11,93	****	<0,0001
0:DMSO vs. 1:Tazarotene 10 µM Bexarotene 20µM	2,984	14,02	****	<0,0001
0:DMSO vs. 5:Tazarotene 10 µM	2,984	12,7	****	<0,0001
0:DMSO vs. 5:Bexarotene 20 µM	2,984	7,002	*	0,0176
0:DMSO vs. 5:Tazarotene 10 µM Bexarotene 20µM	2,984	11,86	****	<0,0001
0:DMSO vs. 10:Tazarotene 10 µM	2,984	10,11	****	<0,0001

0:DMSO vs. 10:Tazarotene 10 µM Bexarotene 20µM	2,984	7,54	***	0,0005
0:DMSO vs. 20:DMSO	2,984	5,528	*	0,0336
0:DMSO vs. 20:Tazarotene 10 µM Bexarotene 20µM	2,984	6,49	*	0,0337
0:Tazarotene 10 µM vs. 0:Bexarotene 20 µM	11,07	5,085	***	0,0002
0:Tazarotene 10 µM vs. 0:Tazarotene 10 µM Bexarotene 20µM	11,07	16,14	**	0,0013
0:Tazarotene 10 µM vs. 1:DMSO	11,07	4,131	****	<0,0001
0:Tazarotene 10 µM vs. 1:Bexarotene 20 µM	11,07	5,477	***	0,0007
0:Tazarotene 10 µM vs. 5:DMSO	11,07	4,441	****	<0,0001
0:Tazarotene 10 µM vs. 10:DMSO	11,07	5,317	****	<0,0001
0:Tazarotene 10 µM vs. 10:Bexarotene 20 µM	11,07	6,532	*	0,0197
0:Tazarotene 10 µM vs. 20:DMSO	11,07	5,528	****	<0,0001
0:Tazarotene 10 µM vs. 20:Tazarotene 10 µM	11,07	6,067	***	0,0007
0:Tazarotene 10 µM vs. 20:Bexarotene 20 µM	11,07	6,448	*	0,0154
0:Tazarotene 10 µM vs. 20:Tazarotene 10 µM Bexarotene 20µM	11,07	6,49	**	0,007
0:Bexarotene 20 µM vs. 0:Tazarotene 10 µM Bexarotene 20µM	5,085	16,14	****	<0,0001
0:Bexarotene 20 µM vs. 1:Tazarotene 10 µM	5,085	11,93	****	<0,0001
0:Bexarotene 20 µM vs. 1:Tazarotene 10 µM Bexarotene 20µM	5,085	14,02	****	<0,0001
0:Bexarotene 20 µM vs. 5:Tazarotene 10 µM	5,085	12,7	****	<0,0001
0:Bexarotene 20 µM vs. 5:Tazarotene 10 µM Bexarotene 20µM	5,085	11,86	****	<0,0001
0:Bexarotene 20 µM vs. 10:Tazarotene 10 µM	5,085	10,11	**	0,0044
0:Tazarotene 10 µM Bexarotene 20µM vs. 1:DMSO	16,14	4,131	****	<0,0001
0:Tazarotene 10 µM Bexarotene 20µM vs. 1:Tazarotene 10 µM	16,14	11,93	*	0,0226
0:Tazarotene 10 µM Bexarotene 20µM vs. 1:Bexarotene 20 µM	16,14	5,477	****	<0,0001
0:Tazarotene 10 µM Bexarotene 20µM vs. 5:DMSO	16,14	4,441	****	<0,0001
0:Tazarotene 10 µM Bexarotene 20µM vs. 5:Tazarotene 10 µM Bexarotene 20µM	16,14	11,86	*	0,0321
0:Tazarotene 10 µM Bexarotene 20µM vs. 10:DMSO	16,14	5,317	****	<0,0001
0:Tazarotene 10 µM Bexarotene 20µM vs. 10:Tazarotene 10 µM	16,14	10,11	****	<0,0001
0:Tazarotene 10 µM Bexarotene 20µM vs. 10:Bexarotene 20 µM	16,14	6,532	****	<0,0001
0:Tazarotene 10 µM Bexarotene 20µM vs. 10:Tazarotene 10 µM Bexarotene 20µM	16,14	7,54	****	<0,0001

0:Tazarotene 10 µM Bexarotene 20µM vs. 20:DMSO	16,14	5,528	****	<0,0001
0:Tazarotene 10 µM Bexarotene 20µM vs. 20:Tazarotene 10 µM	16,14	6,067	****	<0,0001
0:Tazarotene 10 µM Bexarotene 20µM vs. 20:Bexarotene 20 µM	16,14	6,448	****	<0,0001
0:Tazarotene 10 µM Bexarotene 20µM vs. 20:Tazarotene 10 µM Bexarotene 20µM	16,14	6,49	****	<0,0001
1:DMSO vs. 1:Tazarotene 10 µM	4,131	11,93	****	<0,0001
1:DMSO vs. 1:Tazarotene 10 µM Bexarotene 20µM	4,131	14,02	****	<0,0001
1:DMSO vs. 5:Tazarotene 10 µM	4,131	12,7	****	<0,0001
1:DMSO vs. 5:Tazarotene 10 µM Bexarotene 20µM	4,131	11,86	****	<0,0001
1:DMSO vs. 10:Tazarotene 10 µM	4,131	10,11	****	<0,0001
1:DMSO vs. 10:Tazarotene 10 µM Bexarotene 20µM	4,131	7,54	*	0,0464
1:Tazarotene 10 µM vs. 1:Bexarotene 20 µM	11,93	5,477	****	<0,0001
1:Tazarotene 10 µM vs. 5:DMSO	11,93	4,441	****	<0,0001
1:Tazarotene 10 µM vs. 5:Bexarotene 20 µM	11,93	7,002	**	0,0061
1:Tazarotene 10 µM vs. 10:DMSO	11,93	5,317	****	<0,0001
1:Tazarotene 10 µM vs. 10:Bexarotene 20 µM	11,93	6,532	**	0,0013
1:Tazarotene 10 µM vs. 10:Tazarotene 10 µM Bexarotene 20µM	11,93	7,54	*	0,0131
1:Tazarotene 10 µM vs. 20:DMSO	11,93	5,528	****	<0,0001
1:Tazarotene 10 µM vs. 20:Tazarotene 10 µM	11,93	6,067	****	<0,0001
1:Tazarotene 10 µM vs. 20:Bexarotene 20 µM	11,93	6,448	***	0,001
1:Tazarotene 10 µM vs. 20:Tazarotene 10 µM Bexarotene 20µM	11,93	6,49	***	0,0003
1:Bexarotene 20 µM vs. 1:Tazarotene 10 µM Bexarotene 20µM	5,477	14,02	****	<0,0001
1:Bexarotene 20 µM vs. 5:Tazarotene 10 µM	5,477	12,7	****	<0,0001
1:Bexarotene 20 µM vs. 5:Tazarotene 10 µM Bexarotene 20µM	5,477	11,86	****	<0,0001
1:Bexarotene 20 µM vs. 10:Tazarotene 10 µM	5,477	10,11	*	0,0147
1:Tazarotene 10 µM Bexarotene 20µM vs. 5:DMSO	14,02	4,441	****	<0,0001
1:Tazarotene 10 µM Bexarotene 20µM vs. 5:Bexarotene 20 µM	14,02	7,002	****	<0,0001
1:Tazarotene 10 µM Bexarotene 20µM vs. 10:DMSO	14,02	5,317	****	<0,0001
1:Tazarotene 10 µM Bexarotene 20µM vs. 10:Bexarotene 20 µM	14,02	6,532	****	<0,0001
1:Tazarotene 10 µM Bexarotene 20µM vs. 10:Tazarotene 10 µM Bexarotene 20µM	14,02	7,54	****	<0,0001

1:Tazarotene 10 µM Bexarotene 20µM vs. 20:DMSO	14,02	5,528	****	<0,0001
1:Tazarotene 10 µM Bexarotene 20µM vs. 20:Tazarotene 10 µM	14,02	6,067	****	<0,0001
1:Tazarotene 10 µM Bexarotene 20µM vs. 20:Bexarotene 20 µM	14,02	6,448	****	<0,0001
1:Tazarotene 10 µM Bexarotene 20µM vs. 20:Tazarotene 10 µM Bexarotene 20µM	14,02	6,49	****	<0,0001
5:DMSO vs. 5:Tazarotene 10 µM	4,441	12,7	****	<0,0001
5:DMSO vs. 5:Tazarotene 10 µM Bexarotene 20µM	4,441	11,86	****	<0,0001
5:DMSO vs. 10:Tazarotene 10 µM	4,441	10,11	****	<0,0001
5:Tazarotene 10 µM vs. 5:Bexarotene 20 µM	12,7	7,002	***	0,0005
5:Tazarotene 10 µM vs. 10:DMSO	12,7	5,317	****	<0,0001
5:Tazarotene 10 µM vs. 10:Bexarotene 20 µM	12,7	6,532	****	<0,0001
5:Tazarotene 10 µM vs. 10:Tazarotene 10 µM Bexarotene 20µM	12,7	7,54	***	0,0009
5:Tazarotene 10 µM vs. 20:DMSO	12,7	5,528	****	<0,0001
5:Tazarotene 10 µM vs. 20:Tazarotene 10 µM	12,7	6,067	****	<0,0001
5:Tazarotene 10 µM vs. 20:Bexarotene 20 µM	12,7	6,448	****	<0,0001
5:Tazarotene 10 µM vs. 20:Tazarotene 10 µM Bexarotene 20µM	12,7	6,49	****	<0,0001
5:Bexarotene 20 µM vs. 5:Tazarotene 10 µM Bexarotene 20µM	7,002	11,86	*	0,013
5:Tazarotene 10 µM Bexarotene 20µM vs. 10:DMSO	11,86	5,317	****	<0,0001
5:Tazarotene 10 µM Bexarotene 20µM vs. 10:Bexarotene 20 µM	11,86	6,532	**	0,0032
5:Tazarotene 10 µM Bexarotene 20µM vs. 10:Tazarotene 10 µM Bexarotene 20µM	11,86	7,54	*	0,0279
5:Tazarotene 10 µM Bexarotene 20µM vs. 20:DMSO	11,86	5,528	****	<0,0001
5:Tazarotene 10 µM Bexarotene 20µM vs. 20:Tazarotene 10 µM	11,86	6,067	****	<0,0001
5:Tazarotene 10 µM Bexarotene 20µM vs. 20:Bexarotene 20 µM	11,86	6,448	**	0,0024
5:Tazarotene 10 µM Bexarotene 20µM vs. 20:Tazarotene 10 µM Bexarotene 20µM	11,86	6,49	***	0,001
10:DMSO vs. 10:Tazarotene 10 µM	5,317	10,11	****	<0,0001
10:Tazarotene 10 µM vs. 20:DMSO	10,11	5,528	***	0,0001
10:Tazarotene 10 µM vs. 20:Tazarotene 10 µM	10,11	6,067	*	0,0203

Appendix table S22: Mean values and significance levels for Figure 5 panel A (significant differences only)

Comparison	Mean 1	Mean 2	Summary	Adjusted P Value
control DMSO vs. MSD DMSO	-0,01671	-0,1948	*	0,0411
MSD DMSO vs. control taz/bex	-0,1948	0,2035	**	0,0074
MSD DMSO vs. MSD taz/bex	-0,1948	0,0391	*	0,0404

Appendix table S23: Mean values and significance levels for Figure 5 panel C (significant differences only)

Comparison	Mean 1	Mean 2	Summary	Adjusted P Value
control DMSO vs. MSD taz/bex	0,1004	-0,245	*	0,0107
MSD DMSO vs. MSD taz/bex	0,1558	-0,245	**	0,0045
control taz/bex vs. MSD taz/bex	0,006763	-0,245	*	0,0478

Appendix table S24: Mean values and significance levels for Figure 6 panel A (significant differences only)

Comparison	Mean 1	Mean 2	Summary	Adjusted P Value
0/0 vs 10/0	10,13	44	*	0,0402
0/0 vs.10/20	10,13	77,65	***	0,0007
10/0 vs. 10/20	44	77,65	*	0,0414
20/0 vs. 10/20	20,91	77,65	**	0,0022

Appendix table S25: Mean values and significance levels for Figure 6 panel B (significant differences only)

Comparison	Mean 1	Mean 2	Summary	Adjusted P Value
MSDi				
0 vs. 10	4,693	14,64	***	0,0001
0 vs. 25	4,693	21,09	****	<0,0001
0 vs. 50	4,693	19,18	****	<0,0001
10 vs. 25	14,64	21,09	**	0,0048

Appendix table S26: Mean values and significance levels for Figure 6 panel C (significant differences only)

Comparison	Mean 1	Mean 2	Summary	Adjusted P Value
MSD FGE G247R				
0 vs. 6	15,88	113,9	****	<0,0001
0 vs. 14	15,88	181,2	****	<0,0001
0 vs. 21	15,88	168,3	****	<0,0001
6 vs. 14	113,9	181,2	****	<0,0001
6 vs. 21	113,9	168,3	***	0,0003
ARPE 19 SUMF1 +/+				
0 vs. 6	263,5	401,1	****	<0,0001
0 vs. 14	263,5	401,4	****	<0,0001
0 vs. 21	263,5	369,8	****	<0,0001
6 vs. 21	401,1	369,8	*	0,0421
14 vs. 21	401,4	369,8	*	0,0399

Appendix table S27: Mean values and significance levels for Figure 6 panel D (significant differences only)

Comparison	Mean 1	Mean 2	Summary	Adjusted P Value
p.G247R 0/0 vs. 10/20	15,97	73,76	*	0,0124

Appendix table S28: Mean values and significance levels for Figure 7 panel A (significant differences only)

Comparison	Mean 1	Mean 2	Summary	Adjusted P Value
DMSO0 vs. DMSO1	100	51,07	****	<0,0001
DMSO0 vs. DMSO2	100	24,04	****	<0,0001
DMSO0 vs. DMSO3	100	13,15	****	<0,0001
DMSO0 vs. DMSO4	100	7,216	****	<0,0001
DMSO0 vs. TB1	100	80,88	*	0,0427
DMSO0 vs. TB2	100	50,37	****	<0,0001
DMSO0 vs. TB3	100	27,69	****	<0,0001
DMSO0 vs. TB4	100	16,98	****	<0,0001
DMSO1 vs. DMSO2	51,07	24,04	**	0,0017
DMSO1 vs. DMSO3	51,07	13,15	****	<0,0001

DMSO1 vs. DMSO4	51,07	7,216	****	<0,0001
DMSO1 vs. TB0	51,07	100	****	<0,0001
DMSO1 vs. TB1	51,07	80,88	***	0,0005
DMSO1 vs. TB3	51,07	27,69	**	0,0077
DMSO1 vs. TB4	51,07	16,98	****	<0,0001
DMSO2 vs. TB0	24,04	100	****	<0,0001
DMSO2 vs. TB1	24,04	80,88	****	<0,0001
DMSO2 vs. TB2	24,04	50,37	**	0,0023
DMSO3 vs. TB0	13,15	100	****	<0,0001
DMSO3 vs. TB1	13,15	80,88	****	<0,0001
DMSO3 vs. TB2	13,15	50,37	****	<0,0001
DMSO4 vs. TB0	7,216	100	****	<0,0001
DMSO4 vs. TB1	7,216	80,88	****	<0,0001
DMSO4 vs. TB2	7,216	50,37	****	<0,0001
DMSO4 vs. TB3	7,216	27,69	*	0,0251
TB0 vs. TB1	100	80,88	*	0,0427
TB0 vs. TB2	100	50,37	****	<0,0001
TB0 vs. TB3	100	27,69	****	<0,0001
TB0 vs. TB4	100	16,98	****	<0,0001
TB1 vs. TB2	80,88	50,37	***	0,0004
TB1 vs. TB3	80,88	27,69	****	<0,0001
TB1 vs. TB4	80,88	16,98	****	<0,0001
TB2 vs. TB3	50,37	27,69	*	0,0102
TB2 vs. TB4	50,37	16,98	***	0,0001

Appendix table S29: Mean values and significance levels for Figure 7 panel B (significant differences only)

**Comparison**	**Mean 1**	**Mean 2**	**Summary**	**Adjusted P Value**
DMSO0 vs. DMSO1	100	70,59	***	0,0002
DMSO0 vs. Taz/Bex1	100	79,03	**	0,0086
DMSO0 vs. DMSO2	100	43,51	****	<0,0001
DMSO0 vs. Taz/Bex2	100	58,05	****	<0,0001
DMSO0 vs. DMSO3	100	30,77	****	<0,0001
DMSO0 vs. Taz/Bex3	100	46,25	****	<0,0001
DMSO0 vs. DMSO4	100	19,43	****	<0,0001
DMSO0 vs. Taz/Bex4	100	43,13	****	<0,0001
Taz/Bex0 vs. DMSO1	100	70,59	***	0,0002
Taz/Bex0 vs. Taz/Bex1	100	79,03	**	0,0086
Taz/Bex0 vs. DMSO2	100	43,51	****	<0,0001
Taz/Bex0 vs. Taz/Bex2	100	58,05	****	<0,0001
Taz/Bex0 vs. DMSO3	100	30,77	****	<0,0001
Taz/Bex0 vs. Taz/Bex3	100	46,25	****	<0,0001
Taz/Bex0 vs. DMSO4	100	19,43	****	<0,0001

Taz/Bex0 vs. Taz/Bex4	100	43,13	****	<0,0001
DMSO1 vs. DMSO2	70,59	43,51	***	0,0005
DMSO1 vs. DMSO3	70,59	30,77	****	<0,0001
DMSO1 vs. Taz/Bex3	70,59	46,25	**	0,0018
DMSO1 vs. DMSO4	70,59	19,43	****	<0,0001
DMSO1 vs. Taz/Bex4	70,59	43,13	***	0,0004
Taz/Bex1 vs. DMSO2	79,03	43,51	****	<0,0001
Taz/Bex1 vs. Taz/Bex2	79,03	58,05	**	0,0085
Taz/Bex vs. DMSO3	79,03	30,77	****	<0,0001
Taz/Bex1 vs. Taz/Bex3	79,03	46,25	****	<0,0001
Taz/Bex1 vs. DMSO4	79,03	19,43	****	<0,0001
Taz/Bex1 vs. Taz/Bex4	79,03	43,13	****	<0,0001
DMSO2 vs. DMSO4	43,51	19,43	**	0,0021
Taz/Bex2 vs. DMSO3	58,05	30,77	***	0,0005
Taz/Bex2 vs. DMSO4	58,05	19,43	****	<0,0001
Taz/Bex3 vs. DMSO4	46,25	19,43	***	0,0006
DMSO4 vs. Taz/Bex4	19,43	43,13	**	0,0025

Appendix table S30: Mean values and significance levels for Figure 7 panel C (significant differences only)

**Comparison**	**Mean 1**	**Mean 2**	**Summary**	**Adjusted P Value**
DMSO0 vs. DMSO1	100	60,06	**	0,0068
DMSO0 vs. DMSO2	100	43,42	***	0,0001
DMSO0 vs. Taz/Bex2	100	59,69	**	0,0062
DMSO0 vs. DMSO3	100	29,36	****	<0,0001
DMSO0 vs. Taz/Bex3	100	43,26	***	0,0001
DMSO0 vs. DMSO4	100	18,16	****	<0,0001
DMSO0 vs. Taz/Bex4	100	33,55	****	<0,0001
Taz/Bex0 vs. DMSO1	100	60,06	**	0,0068
Taz/Bex0 vs. DMSO2	100	43,42	***	0,0001
Taz/Bex0 vs. Taz/Bex2	100	59,69	**	0,0062
Taz/Bex0 vs. DMSO3	100	29,36	****	<0,0001
Taz/Bex0 vs. Taz/Bex3	100	43,26	***	0,0001
Taz/Bex0 vs. DMSO4	100	18,16	****	<0,0001
Taz/Bex0 vs. Taz/Bex4	100	33,55	****	<0,0001
DMSO1 vs. DMSO4	60,06	18,16	**	0,0042
Taz/Bex1 vs. DMSO2	78,12	43,42	*	0,0241
Taz/Bex1 vs. DMSO3	78,12	29,36	***	0,0008
Taz/Bex1 vs. Taz/Bex3	78,12	43,26	*	0,0232
Taz/Bex1 vs. DMSO4	78,12	18,16	****	<0,0001
Taz/Bex1 vs. Taz/Bex4	78,12	33,55	**	0,0022
Taz/Bex2 vs. DMSO4	59,69	18,16	**	0,0046

Appendix table S31: Mean values and significance levels for EV Figure 1 panel A (significant differences only)

Comparison	Mean 1	Mean 2	Summary	Adjusted P Value
neg. contr. vs. Tazarotene	8,696	28,37	****	<0,0001
neg. contr. vs. pos. contr.	8,696	53,63	****	<0,0001
Tazarotene vs. Clindamycin	28,37	10,46	****	<0,0001
Tazarotene vs. Vorinostat	28,37	7,913	****	<0,0001
Tazarotene vs. Asenapine	28,37	9,489	****	<0,0001
Tazarotene vs. pos. contr.	28,37	53,63	****	<0,0001
Clindamycin vs. pos. contr.	10,46	53,63	****	<0,0001
Vorinostat vs. pos. contr.	7,913	53,63	****	<0,0001
Asenapine vs. pos. contr.	9,489	53,63	****	<0,0001

Appendix table S32: Mean values and significance levels for EV Figure 1 panel B (significant differences only)

Comparison	Mean 1	Mean 2	Summary	Adjusted P Value
neg. contr. vs. Tazarotene	1,307	2,782	**	0,0025
Tazarotene vs. Clindamycin	2,782	1,616	*	0,0223
Tazarotene vs. Vorinostat	2,782	1,445	**	0,0068

Appendix table S33: Mean values and significance levels for EV Figure 1 panel C (significant differences only)

Comparison	Mean 1	Mean 2	Summary	Adjusted P Value
neg. contr. vs. Tazarotene	1,517	3,125	**	0,0055
neg. contr. vs. pos. contr.	1,517	2,766	*	0,0446
Tazarotene vs. Vorinostat	3,125	1,315	**	0,0016
Vorinostat vs. pos. contr.	1,315	2,766	*	0,0141

Appendix table S34: Mean values and significance levels for EV Figure 2 panel E (significant differences only)

Comparison	Mean 1	Mean 2	Summary	Adjusted P Value

0/0 vs 0.25/0.5	13,43	21,47	*	0,0252
0/0 vs 0.5/1	13,43	26,38	***	0,0003
0/0 vs 1/2	13,43	39,13	****	<0,0001
0/0 vs 2.5/5	13,43	74,57	****	<0,0001
0.1/0.2 vs 0.5/1	18,71	26,38	*	0,0352
0.1/0.2 vs 1/2	18,71	39,13	****	<0,0001
0.1/0.2 vs 2.5/5	18,71	74,57	****	<0,0001
0.25/0.5 vs 1/2	21,47	39,13	****	<0,0001
0.25/0.5 vs 2.5/5	21,47	74,57	****	<0,0001
0.5/1 vs 1/2	26,38	39,13	***	0,0003
0.5/1 vs 2.5/5	26,38	74,57	****	<0,0001
1/2 vs 2.5/5	39,13	74,57	****	<0,0001

Appendix table S35: Mean values and significance levels for EV Figure 2 panel F (significant differences only)

**Comparison**	**Mean 1**	**Mean 2**	**Summary**	**Adjusted P Value**
p.G247R - vs. +	17,0	49,2	****	<0,0001
p.G263V - vs. +	130,5	282,4	****	<0,0001
p.A279V - vs. +	132,0	227,8	***	0,0007
p.R349W - vs. +	20,5	39,8	*	0,0152
p.A279V - vs. +	96,0	120,6	**	0,001
p.R349W - vs. +	27,9	67,5	**	0,0015
p.G247R - vs. +	18,1	60,3	*	0,0132

Appendix table S36: Mean values and significance levels for EV Figure 3 panel A (significant differences only)

**Comparison**	**Mean 1**	**Mean 2**	**Summary**	**Adjusted P Value**
RARRES1				
ctrl- vs. ctrl+	0,5626	73,99	****	<0,0001
ctrl- vs. MSD+	0,5626	65,22	****	<0,0001
MSD- vs. ctrl+	1,696	73,99	****	<0,0001
MSD- vs. MSD+	1,696	65,22	****	<0,0001
CYP26B1				
ctrl- vs. ctrl+	7,032	406,7	**	0,0033
ctrl- vs. MSD+	7,032	309,4	*	0,0209
MSD- vs. ctrl+	4,105	406,7	**	0,0021

MSD- vs. MSD+	4,105	309,4	*	0,014

Appendix table S37: Mean values and significance levels for EV Figure 3 panel B (significant differences only)

**Comparison**	**Mean 1**	**Mean 2**	**Summary**	**Adjusted P Value**
SUMF1				
MSD- vs. MSD+	11,21	7,926	*	0,0213

Appendix table S38: Mean values and significance levels for EV Figure 3 panel C (significant differences only)

**Comparison**	**Mean 1**	**Mean 2**	**Summary**	**Adjusted P Value**
ARSB				
ctrl- vs. ctrl+	11,16	7,122	*	0,0278
STS				
ctrl- vs. ctrl+	14,12	6,614	*	0,0331
MSD- vs. ctrl+	15,14	6,614	*	0,0101
MSD- vs. MSD+	15,14	7,528	*	0,0165
SULF1				
ctrl- vs. ctrl+	89,04	793,3	****	<0,0001
ctrl- vs. MSD+	89,04	826,2	****	<0,0001
MSD- vs. ctrl+	121,3	793,3	****	<0,0001
MSD- vs. MSD+	121,3	826,2	****	<0,0001

Appendix table S39: Mean values and significance levels for EV Figure 4 panel A (significant differences only)

**Comparison**	**Mean 1**	**Mean 2**	**Summary**	**Adjusted P Value**
control DMSO vs. MSD DMSO	0,2	0,2	ns	0,9972
control DMSO vs. control taz/bex	0,2085	-0,1749	****	<0,0001
control DMSO vs. MSD taz/bex	0,2085	-0,2289	****	<0,0001
MSD DMSO vs. control taz/bex	0,2009	-0,1749	****	<0,0001
MSD DMSO vs. MSD taz/bex	0,2009	-0,2289	****	<0,0001
control taz/bex vs. MSD taz/bex	-0,1749	-0,2289	ns	0,518

Appendix table S40: Mean values and significance levels for EV Figure 4 panel C (significant differences only)

**Comparison**	**Mean 1**	**Mean 2**	**Summary**	**Adjusted P Value**
control DMSO vs. control taz/bex	-0,1835	0,1222	**	0,0081
control DMSO vs. MSD taz/bex	-0,1835	0,1664	*	0,0147
MSD DMSO vs. control taz/bex	-0,1153	0,1222	*	0,0215
MSD DMSO vs. MSD taz/bex	-0,1153	0,1664	*	0,0275

Appendix table S41: Mean values and significance levels for MSD fibroblast cell count comparison (Appendix fig. S5A, significant differences only)

**Comparison**	**Mean 1**	**Mean 2**	**Summary**	**Adjusted P Value**
0d:MSD DMSO low vs. 3d:MSD DMSO low	450000	1693000	**	0,008
0d:MSD DMSO low vs. 3d:MSD DMSO medium	450000	2787167	****	<0,0001
0d:MSD DMSO low vs. 3d:MSD DMSO high	450000	3110833	****	<0,0001
0d:MSD DMSO low vs. 3d:MSD Taz	450000	2204167	****	<0,0001
0d:MSD DMSO low vs. 3d:MSD Bex	450000	2237500	****	<0,0001
0d:MSD DMSO low vs. 3d:MSD Taz/Bex	450000	1879167	***	0,0007
0d:MSD DMSO low vs. 6d:MSD DMSO low	450000	3514000	****	<0,0001
0d:MSD DMSO low vs. 6d:MSD DMSO medium	450000	3279333	****	<0,0001
0d:MSD DMSO low vs. 6d:MSD DMSO high	450000	3255167	****	<0,0001
0d:MSD DMSO low vs. 6d:MSD Taz	450000	2434333	****	<0,0001
0d:MSD DMSO low vs. 6d:MSD Bex	450000	2474333	****	<0,0001
0d:MSD DMSO low vs. 6d:MSD Taz/Bex	450000	1977167	***	0,0002
0d:MSD DMSO low vs. 9d:MSD DMSO low	450000	3909667	****	<0,0001
0d:MSD DMSO low vs. 9d:MSD DMSO medium	450000	3771250	****	<0,0001
0d:MSD DMSO low vs. 9d:MSD DMSO high	450000	3527833	****	<0,0001
0d:MSD DMSO low vs. 9d:MSD Taz	450000	2496667	****	<0,0001
0d:MSD DMSO low vs. 9d:MSD Bex	450000	2628833	****	<0,0001
0d:MSD DMSO low vs. 9d:MSD Taz/Bex	450000	2005917	***	0,0001
0d:MSD DMSO medium vs. 3d:MSD DMSO medium	900000	2787167	****	<0,0001
0d:MSD DMSO medium vs. 3d:MSD DMSO high	900000	3110833	****	<0,0001
0d:MSD DMSO medium vs. 3d:MSD Taz	900000	2204167	**	0,0037
0d:MSD DMSO medium vs. 3d:MSD Bex	900000	2237500	**	0,0024

0d:MSD DMSO medium vs. 6d:MSD DMSO low	900000	3514000	****	<0,0001
0d:MSD DMSO medium vs. 6d:MSD DMSO medium	900000	3279333	****	<0,0001
0d:MSD DMSO medium vs. 6d:MSD DMSO high	900000	3255167	****	<0,0001
0d:MSD DMSO medium vs. 6d:MSD Taz	900000	2434333	***	0,0001
0d:MSD DMSO medium vs. 6d:MSD Bex	900000	2474333	****	<0,0001
0d:MSD DMSO medium vs. 9d:MSD DMSO low	900000	3909667	****	<0,0001
0d:MSD DMSO medium vs. 9d:MSD DMSO medium	900000	3771250	****	<0,0001
0d:MSD DMSO medium vs. 9d:MSD DMSO high	900000	3527833	****	<0,0001
0d:MSD DMSO medium vs. 9d:MSD Taz	900000	2496667	****	<0,0001
0d:MSD DMSO medium vs. 9d:MSD Bex	900000	2628833	****	<0,0001
0d:MSD DMSO medium vs. 9d:MSD Taz/Bex	900000	2005917	*	0,0389
0d:MSD DMSO high vs. 3d:MSD DMSO medium	1350000	2787167	***	0,0006
0d:MSD DMSO high vs. 3d:MSD DMSO high	1350000	3110833	****	<0,0001
0d:MSD DMSO high vs. 6d:MSD DMSO low	1350000	3514000	****	<0,0001
0d:MSD DMSO high vs. 6d:MSD DMSO medium	1350000	3279333	****	<0,0001
0d:MSD DMSO high vs. 6d:MSD DMSO high	1350000	3255167	****	<0,0001
0d:MSD DMSO high vs. 6d:MSD Taz	1350000	2434333	*	0,0488
0d:MSD DMSO high vs. 6d:MSD Bex	1350000	2474333	*	0,0318
0d:MSD DMSO high vs. 9d:MSD DMSO low	1350000	3909667	****	<0,0001
0d:MSD DMSO high vs. 9d:MSD DMSO medium	1350000	3771250	****	<0,0001
0d:MSD DMSO high vs. 9d:MSD DMSO high	1350000	3527833	****	<0,0001
0d:MSD DMSO high vs. 9d:MSD Taz	1350000	2496667	*	0,0248
0d:MSD DMSO high vs. 9d:MSD Bex	1350000	2628833	**	0,0051
0d:MSD Taz vs. 3d:MSD DMSO medium	900000	2787167	****	<0,0001
0d:MSD Taz vs. 3d:MSD DMSO high	900000	3110833	****	<0,0001
0d:MSD Taz vs. 3d:MSD Taz	900000	2204167	**	0,0037
0d:MSD Taz vs. 3d:MSD Bex	900000	2237500	**	0,0024
0d:MSD Taz vs. 6d:MSD DMSO low	900000	3514000	****	<0,0001
0d:MSD Taz vs. 6d:MSD DMSO medium	900000	3279333	****	<0,0001
0d:MSD Taz vs. 6d:MSD DMSO high	900000	3255167	****	<0,0001
0d:MSD Taz vs. 6d:MSD Taz	900000	2434333	***	0,0001
0d:MSD Taz vs. 6d:MSD Bex	900000	2474333	****	<0,0001
0d:MSD Taz vs. 9d:MSD DMSO low	900000	3909667	****	<0,0001
0d:MSD Taz vs. 9d:MSD DMSO medium	900000	3771250	****	<0,0001
0d:MSD Taz vs. 9d:MSD DMSO high	900000	3527833	****	<0,0001
0d:MSD Taz vs. 9d:MSD Taz	900000	2496667	****	<0,0001
0d:MSD Taz vs. 9d:MSD Bex	900000	2628833	****	<0,0001
0d:MSD Taz vs. 9d:MSD Taz/Bex	900000	2005917	*	0,0389
0d:MSD Bex vs. 3d:MSD DMSO medium	900000	2787167	****	<0,0001

0d:MSD Bex vs. 3d:MSD DMSO high	900000	3110833	****	<0,0001
0d:MSD Bex vs. 3d:MSD Taz	900000	2204167	**	0,0037
0d:MSD Bex vs. 3d:MSD Bex	900000	2237500	**	0,0024
0d:MSD Bex vs. 6d:MSD DMSO low	900000	3514000	****	<0,0001
0d:MSD Bex vs. 6d:MSD DMSO medium	900000	3279333	****	<0,0001
0d:MSD Bex vs. 6d:MSD DMSO high	900000	3255167	****	<0,0001
0d:MSD Bex vs. 6d:MSD Taz	900000	2434333	***	0,0001
0d:MSD Bex vs. 6d:MSD Bex	900000	2474333	****	<0,0001
0d:MSD Bex vs. 9d:MSD DMSO low	900000	3909667	****	<0,0001
0d:MSD Bex vs. 9d:MSD DMSO medium	900000	3771250	****	<0,0001
0d:MSD Bex vs. 9d:MSD DMSO high	900000	3527833	****	<0,0001
0d:MSD Bex vs. 9d:MSD Taz	900000	2496667	****	<0,0001
0d:MSD Bex vs. 9d:MSD Bex	900000	2628833	****	<0,0001
0d:MSD Bex vs. 9d:MSD Taz/Bex	900000	2005917	*	0,0389
0d:MSD Taz/Bex vs. 3d:MSD DMSO medium	900000	2787167	****	<0,0001
0d:MSD Taz/Bex vs. 3d:MSD DMSO high	900000	3110833	****	<0,0001
0d:MSD Taz/Bex vs. 3d:MSD Taz	900000	2204167	**	0,0037
0d:MSD Taz/Bex vs. 3d:MSD Bex	900000	2237500	**	0,0024
0d:MSD Taz/Bex vs. 6d:MSD DMSO low	900000	3514000	****	<0,0001
0d:MSD Taz/Bex vs. 6d:MSD DMSO medium	900000	3279333	****	<0,0001
0d:MSD Taz/Bex vs. 6d:MSD DMSO high	900000	3255167	****	<0,0001
0d:MSD Taz/Bex vs. 6d:MSD Taz	900000	2434333	***	0,0001
0d:MSD Taz/Bex vs. 6d:MSD Bex	900000	2474333	****	<0,0001
0d:MSD Taz/Bex vs. 9d:MSD DMSO low	900000	3909667	****	<0,0001
0d:MSD Taz/Bex vs. 9d:MSD DMSO medium	900000	3771250	****	<0,0001
0d:MSD Taz/Bex vs. 9d:MSD DMSO high	900000	3527833	****	<0,0001
0d:MSD Taz/Bex vs. 9d:MSD Taz	900000	2496667	****	<0,0001
0d:MSD Taz/Bex vs. 9d:MSD Bex	900000	2628833	****	<0,0001
0d:MSD Taz/Bex vs. 9d:MSD Taz/Bex	900000	2005917	*	0,0389
3d:MSD DMSO low vs. 3d:MSD DMSO medium	1693000	2787167	*	0,044
3d:MSD DMSO low vs. 3d:MSD DMSO high	1693000	3110833	***	0,0008
3d:MSD DMSO low vs. 6d:MSD DMSO low	1693000	3514000	****	<0,0001
3d:MSD DMSO low vs. 6d:MSD DMSO medium	1693000	3279333	****	<0,0001
3d:MSD DMSO low vs. 6d:MSD DMSO high	1693000	3255167	****	<0,0001
3d:MSD DMSO low vs. 9d:MSD DMSO low	1693000	3909667	****	<0,0001
3d:MSD DMSO low vs. 9d:MSD DMSO medium	1693000	3771250	****	<0,0001
3d:MSD DMSO low vs. 9d:MSD DMSO high	1693000	3527833	****	<0,0001
3d:MSD DMSO medium vs. 9d:MSD DMSO low	2787167	3909667	*	0,0325
3d:MSD DMSO high vs. 3d:MSD Taz/Bex	3110833	1879167	**	0,0092
3d:MSD DMSO high vs. 6d:MSD Taz/Bex	3110833	1977167	*	0,0287
3d:MSD DMSO high vs. 9d:MSD Taz/Bex	3110833	2005917	*	0,0393
3d:MSD Taz vs. 6d:MSD DMSO low	2204167	3514000	**	0,0034
3d:MSD Taz vs. 9d:MSD DMSO low	2204167	3909667	****	<0,0001
3d:MSD Taz vs. 9d:MSD DMSO medium	2204167	3771250	****	<0,0001

3d:MSD Taz vs. 9d:MSD DMSO high	2204167	3527833	**	0,0028
3d:MSD Bex vs. 6d:MSD DMSO low	2237500	3514000	**	0,0052
3d:MSD Bex vs. 9d:MSD DMSO low	2237500	3909667	****	<0,0001
3d:MSD Bex vs. 9d:MSD DMSO medium	2237500	3771250	***	0,0001
3d:MSD Bex vs. 9d:MSD DMSO high	2237500	3527833	**	0,0044
3d:MSD Taz/Bex vs. 6d:MSD DMSO low	1879167	3514000	****	<0,0001
3d:MSD Taz/Bex vs. 6d:MSD DMSO medium	1879167	3279333	**	0,001
3d:MSD Taz/Bex vs. 6d:MSD DMSO high	1879167	3255167	**	0,0014
3d:MSD Taz/Bex vs. 9d:MSD DMSO low	1879167	3909667	****	<0,0001
3d:MSD Taz/Bex vs. 9d:MSD DMSO medium	1879167	3771250	****	<0,0001
3d:MSD Taz/Bex vs. 9d:MSD DMSO high	1879167	3527833	****	<0,0001
6d:MSD DMSO low vs. 6d:MSD Taz/Bex	3514000	1977167	***	0,0001
6d:MSD DMSO low vs. 9d:MSD Taz/Bex	3514000	2005917	***	0,0002
6d:MSD DMSO medium vs. 6d:MSD Taz/Bex	3279333	1977167	**	0,0038
6d:MSD DMSO medium vs. 9d:MSD Taz/Bex	3279333	2005917	**	0,0054
6d:MSD DMSO high vs. 6d:MSD Taz/Bex	3255167	1977167	**	0,0051
6d:MSD DMSO high vs. 9d:MSD Taz/Bex	3255167	2005917	**	0,0074
6d:MSD Taz vs. 9d:MSD DMSO low	2434333	3909667	***	0,0003
6d:MSD Taz vs. 9d:MSD DMSO medium	2434333	3771250	**	0,0024
6d:MSD Taz vs. 9d:MSD DMSO high	2434333	3527833	*	0,0444
6d:MSD Bex vs. 9d:MSD DMSO low	2474333	3909667	***	0,0006
6d:MSD Bex vs. 9d:MSD DMSO medium	2474333	3771250	**	0,004
6d:MSD Taz/Bex vs. 9d:MSD DMSO low	1977167	3909667	****	<0,0001
6d:MSD Taz/Bex vs. 9d:MSD DMSO medium	1977167	3771250	****	<0,0001
6d:MSD Taz/Bex vs. 9d:MSD DMSO high	1977167	3527833	***	0,0001
9d:MSD DMSO low vs. 9d:MSD Taz	3909667	2496667	***	0,0008
9d:MSD DMSO low vs. 9d:MSD Bex	3909667	2628833	**	0,0049
9d:MSD DMSO low vs. 9d:MSD Taz/Bex	3909667	2005917	****	<0,0001
9d:MSD DMSO medium vs. 9d:MSD Taz	3771250	2496667	**	0,0054
9d:MSD DMSO medium vs. 9d:MSD Bex	3771250	2628833	*	0,0261
9d:MSD DMSO medium vs. 9d:MSD Taz/Bex	3771250	2005917	****	<0,0001
9d:MSD DMSO high vs. 9d:MSD Taz/Bex	3527833	2005917	***	0,0002

Appendix table S42: Mean values and significance levels for control fibroblast cell count comparison (Appendix fig. S5B, significant differences only)

**Comparison**	**Mean 1**	**Mean 2**	**Summary**	**Adjusted P Value**
0d:Ctrl. DMSO low vs. 0d:Ctrl. DMSO high	450000	1350000	***	0,0005
0d:Ctrl. DMSO low vs. 3d:Ctrl. DMSO low	450000	1483800	****	<0,0001
0d:Ctrl. DMSO low vs. 3d:Ctrl. DMSO medium	450000	1854200	****	<0,0001
0d:Ctrl. DMSO low vs. 3d:Ctrl. DMSO high	450000	2272200	****	<0,0001
0d:Ctrl. DMSO low vs. 3d:Ctrl. Taz	450000	1394200	***	0,0002
0d:Ctrl. DMSO low vs. 3d:Ctrl. Bex	450000	1548200	****	<0,0001
0d:Ctrl. DMSO low vs. 3d:Ctrl. Taz/Bex	450000	1293200	**	0,0018

0d:Ctrl. DMSO low vs. 6d:Ctrl. DMSO low	450000	2646000	****	<0,0001
0d:Ctrl. DMSO low vs. 6d:Ctrl. DMSO medium	450000	2476000	****	<0,0001
0d:Ctrl. DMSO low vs. 6d:Ctrl. DMSO high	450000	2682000	****	<0,0001
0d:Ctrl. DMSO low vs. 6d:Ctrl. Taz	450000	1285200	**	0,0021
0d:Ctrl. DMSO low vs. 6d:Ctrl. Bex	450000	1436800	****	<0,0001
0d:Ctrl. DMSO low vs. 6d:Ctrl. Taz/Bex	450000	1393400	***	0,0002
0d:Ctrl. DMSO low vs. 9d:Ctrl. DMSO low	450000	3385000	****	<0,0001
0d:Ctrl. DMSO low vs. 9d:Ctrl. DMSO medium	450000	3084600	****	<0,0001
0d:Ctrl. DMSO low vs. 9d:Ctrl. DMSO high	450000	2919600	****	<0,0001
0d:Ctrl. DMSO low vs. 9d:Ctrl. Taz	450000	1288600	**	0,0019
0d:Ctrl. DMSO low vs. 9d:Ctrl. Bex	450000	1498400	****	<0,0001
0d:Ctrl. DMSO low vs. 9d:Ctrl. Taz/Bex	450000	1340600	***	0,0006
0d:Ctrl. DMSO medium vs. 3d:Ctrl. DMSO medium	900000	1854200	***	0,0001
0d:Ctrl. DMSO medium vs. 3d:Ctrl. DMSO high	900000	2272200	****	<0,0001
0d:Ctrl. DMSO medium vs. 6d:Ctrl. DMSO low	900000	2646000	****	<0,0001
0d:Ctrl. DMSO medium vs. 6d:Ctrl. DMSO medium	900000	2476000	****	<0,0001
0d:Ctrl. DMSO medium vs. 6d:Ctrl. DMSO high	900000	2682000	****	<0,0001
0d:Ctrl. DMSO medium vs. 9d:Ctrl. DMSO low	900000	3385000	****	<0,0001
0d:Ctrl. DMSO medium vs. 9d:Ctrl. DMSO medium	900000	3084600	****	<0,0001
0d:Ctrl. DMSO medium vs. 9d:Ctrl. DMSO high	900000	2919600	****	<0,0001
0d:Ctrl. DMSO high vs. 3d:Ctrl. DMSO high	1350000	2272200	***	0,0003
0d:Ctrl. DMSO high vs. 6d:Ctrl. DMSO low	1350000	2646000	****	<0,0001
0d:Ctrl. DMSO high vs. 6d:Ctrl. DMSO medium	1350000	2476000	****	<0,0001
0d:Ctrl. DMSO high vs. 6d:Ctrl. DMSO high	1350000	2682000	****	<0,0001
0d:Ctrl. DMSO high vs. 9d:Ctrl. DMSO low	1350000	3385000	****	<0,0001
0d:Ctrl. DMSO high vs. 9d:Ctrl. DMSO medium	1350000	3084600	****	<0,0001
0d:Ctrl. DMSO high vs. 9d:Ctrl. DMSO high	1350000	2919600	****	<0,0001
0d:Ctrl. Taz vs. 3d:Ctrl. DMSO medium	900000	1854200	***	0,0001
0d:Ctrl. Taz vs. 3d:Ctrl. DMSO high	900000	2272200	****	<0,0001
0d:Ctrl. Taz vs. 6d:Ctrl. DMSO low	900000	2646000	****	<0,0001
0d:Ctrl. Taz vs. 6d:Ctrl. DMSO medium	900000	2476000	****	<0,0001
0d:Ctrl. Taz vs. 6d:Ctrl. DMSO high	900000	2682000	****	<0,0001
0d:Ctrl. Taz vs. 9d:Ctrl. DMSO low	900000	3385000	****	<0,0001
0d:Ctrl. Taz vs. 9d:Ctrl. DMSO medium	900000	3084600	****	<0,0001
0d:Ctrl. Taz vs. 9d:Ctrl. DMSO high	900000	2919600	****	<0,0001
0d:Ctrl. Bex vs. 3d:Ctrl. DMSO medium	900000	1854200	***	0,0001
0d:Ctrl. Bex vs. 3d:Ctrl. DMSO high	900000	2272200	****	<0,0001
0d:Ctrl. Bex vs. 6d:Ctrl. DMSO low	900000	2646000	****	<0,0001
0d:Ctrl. Bex vs. 6d:Ctrl. DMSO medium	900000	2476000	****	<0,0001
0d:Ctrl. Bex vs. 6d:Ctrl. DMSO high	900000	2682000	****	<0,0001
0d:Ctrl. Bex vs. 9d:Ctrl. DMSO low	900000	3385000	****	<0,0001
0d:Ctrl. Bex vs. 9d:Ctrl. DMSO medium	900000	3084600	****	<0,0001
0d:Ctrl. Bex vs. 9d:Ctrl. DMSO high	900000	2919600	****	<0,0001
0d:Ctrl. Taz/Bex vs. 3d:Ctrl. DMSO medium	900000	1854200	***	0,0001

0d:Ctrl. Taz/Bex vs. 3d:Ctrl. DMSO high	900000	2272200	****	<0,0001
0d:Ctrl. Taz/Bex vs. 6d:Ctrl. DMSO low	900000	2646000	****	<0,0001
0d:Ctrl. Taz/Bex vs. 6d:Ctrl. DMSO medium	900000	2476000	****	<0,0001
0d:Ctrl. Taz/Bex vs. 6d:Ctrl. DMSO high	900000	2682000	****	<0,0001
0d:Ctrl. Taz/Bex vs. 9d:Ctrl. DMSO low	900000	3385000	****	<0,0001
0d:Ctrl. Taz/Bex vs. 9d:Ctrl. DMSO medium	900000	3084600	****	<0,0001
0d:Ctrl. Taz/Bex vs. 9d:Ctrl. DMSO high	900000	2919600	****	<0,0001
3d:Ctrl. DMSO low vs. 3d:Ctrl. DMSO high	1483800	2272200	**	0,0055
3d:Ctrl. DMSO low vs. 6d:Ctrl. DMSO low	1483800	2646000	****	<0,0001
3d:Ctrl. DMSO low vs. 6d:Ctrl. DMSO medium	1483800	2476000	****	<0,0001
3d:Ctrl. DMSO low vs. 6d:Ctrl. DMSO high	1483800	2682000	****	<0,0001
3d:Ctrl. DMSO low vs. 9d:Ctrl. DMSO low	1483800	3385000	****	<0,0001
3d:Ctrl. DMSO low vs. 9d:Ctrl. DMSO medium	1483800	3084600	****	<0,0001
3d:Ctrl. DMSO low vs. 9d:Ctrl. DMSO high	1483800	2919600	****	<0,0001
3d:Ctrl. DMSO medium vs. 6d:Ctrl. DMSO low	1854200	2646000	**	0,0051
3d:Ctrl. DMSO medium vs. 6d:Ctrl. DMSO high	1854200	2682000	**	0,0024
3d:Ctrl. DMSO medium vs. 9d:Ctrl. DMSO low	1854200	3385000	****	<0,0001
3d:Ctrl. DMSO medium vs. 9d:Ctrl. DMSO medium	1854200	3084600	****	<0,0001
3d:Ctrl. DMSO medium vs. 9d:Ctrl. DMSO high	1854200	2919600	****	<0,0001
3d:Ctrl. DMSO high vs. 3d:Ctrl. Taz	2272200	1394200	***	0,0008
3d:Ctrl. DMSO high vs. 3d:Ctrl. Bex	2272200	1548200	*	0,0189
3d:Ctrl. DMSO high vs. 3d:Ctrl. Taz/Bex	2272200	1293200	****	<0,0001
3d:Ctrl. DMSO high vs. 6d:Ctrl. Taz	2272200	1285200	****	<0,0001
3d:Ctrl. DMSO high vs. 6d:Ctrl. Bex	2272200	1436800	**	0,0021
3d:Ctrl. DMSO high vs. 6d:Ctrl. Taz/Bex	2272200	1393400	***	0,0008
3d:Ctrl. DMSO high vs. 9d:Ctrl. DMSO low	2272200	3385000	****	<0,0001
3d:Ctrl. DMSO high vs. 9d:Ctrl. DMSO medium	2272200	3084600	**	0,0034
3d:Ctrl. DMSO high vs. 9d:Ctrl. Taz	2272200	1288600	****	<0,0001
3d:Ctrl. DMSO high vs. 9d:Ctrl. Bex	2272200	1498400	**	0,0073
3d:Ctrl. DMSO high vs. 9d:Ctrl. Taz/Bex	2272200	1340600	***	0,0003
3d:Ctrl. Taz vs. 6d:Ctrl. DMSO low	1394200	2646000	****	<0,0001
3d:Ctrl. Taz vs. 6d:Ctrl. DMSO medium	1394200	2476000	****	<0,0001
3d:Ctrl. Taz vs. 6d:Ctrl. DMSO high	1394200	2682000	****	<0,0001
3d:Ctrl. Taz vs. 9d:Ctrl. DMSO low	1394200	3385000	****	<0,0001
3d:Ctrl. Taz vs. 9d:Ctrl. DMSO medium	1394200	3084600	****	<0,0001
3d:Ctrl. Taz vs. 9d:Ctrl. DMSO high	1394200	2919600	****	<0,0001
3d:Ctrl. Bex vs. 6d:Ctrl. DMSO low	1548200	2646000	****	<0,0001
3d:Ctrl. Bex vs. 6d:Ctrl. DMSO medium	1548200	2476000	***	0,0003
3d:Ctrl. Bex vs. 6d:Ctrl. DMSO high	1548200	2682000	****	<0,0001
3d:Ctrl. Bex vs. 9d:Ctrl. DMSO low	1548200	3385000	****	<0,0001
3d:Ctrl. Bex vs. 9d:Ctrl. DMSO medium	1548200	3084600	****	<0,0001
3d:Ctrl. Bex vs. 9d:Ctrl. DMSO high	1548200	2919600	****	<0,0001
3d:Ctrl. Taz/Bex vs. 6d:Ctrl. DMSO low	1293200	2646000	****	<0,0001
3d:Ctrl. Taz/Bex vs. 6d:Ctrl. DMSO medium	1293200	2476000	****	<0,0001
3d:Ctrl. Taz/Bex vs. 6d:Ctrl. DMSO high	1293200	2682000	****	<0,0001
3d:Ctrl. Taz/Bex vs. 9d:Ctrl. DMSO low	1293200	3385000	****	<0,0001

3d:Ctrl. Taz/Bex vs. 9d:Ctrl. DMSO medium	1293200	3084600	****	<0,0001
3d:Ctrl. Taz/Bex vs. 9d:Ctrl. DMSO high	1293200	2919600	****	<0,0001
6d:Ctrl. DMSO low vs. 6d:Ctrl. Taz	2646000	1285200	****	<0,0001
6d:Ctrl. DMSO low vs. 6d:Ctrl. Bex	2646000	1436800	****	<0,0001
6d:Ctrl. DMSO low vs. 6d:Ctrl. Taz/Bex	2646000	1393400	****	<0,0001
6d:Ctrl. DMSO low vs. 9d:Ctrl. DMSO low	2646000	3385000	*	0,0143
6d:Ctrl. DMSO low vs. 9d:Ctrl. Taz	2646000	1288600	****	<0,0001
6d:Ctrl. DMSO low vs. 9d:Ctrl. Bex	2646000	1498400	****	<0,0001
6d:Ctrl. DMSO low vs. 9d:Ctrl. Taz/Bex	2646000	1340600	****	<0,0001
6d:Ctrl. DMSO medium vs. 6d:Ctrl. Taz	2476000	1285200	****	<0,0001
6d:Ctrl. DMSO medium vs. 6d:Ctrl. Bex	2476000	1436800	****	<0,0001
6d:Ctrl. DMSO medium vs. 6d:Ctrl. Taz/Bex	2476000	1393400	****	<0,0001
6d:Ctrl. DMSO medium vs. 9d:Ctrl. DMSO low	2476000	3385000	***	0,0004
6d:Ctrl. DMSO medium vs. 9d:Ctrl. Taz	2476000	1288600	****	<0,0001
6d:Ctrl. DMSO medium vs. 9d:Ctrl. Bex	2476000	1498400	****	<0,0001
6d:Ctrl. DMSO medium vs. 9d:Ctrl. Taz/Bex	2476000	1340600	****	<0,0001
6d:Ctrl. DMSO high vs. 6d:Ctrl. Taz	2682000	1285200	****	<0,0001
6d:Ctrl. DMSO high vs. 6d:Ctrl. Bex	2682000	1436800	****	<0,0001
6d:Ctrl. DMSO high vs. 6d:Ctrl. Taz/Bex	2682000	1393400	****	<0,0001
6d:Ctrl. DMSO high vs. 9d:Ctrl. DMSO low	2682000	3385000	*	0,0275
6d:Ctrl. DMSO high vs. 9d:Ctrl. Taz	2682000	1288600	****	<0,0001
6d:Ctrl. DMSO high vs. 9d:Ctrl. Bex	2682000	1498400	****	<0,0001
6d:Ctrl. DMSO high vs. 9d:Ctrl. Taz/Bex	2682000	1340600	****	<0,0001
6d:Ctrl. Taz vs. 9d:Ctrl. DMSO low	1285200	3385000	****	<0,0001
6d:Ctrl. Taz vs. 9d:Ctrl. DMSO medium	1285200	3084600	****	<0,0001
6d:Ctrl. Taz vs. 9d:Ctrl. DMSO high	1285200	2919600	****	<0,0001
6d:Ctrl. Bex vs. 9d:Ctrl. DMSO low	1436800	3385000	****	<0,0001
6d:Ctrl. Bex vs. 9d:Ctrl. DMSO medium	1436800	3084600	****	<0,0001
6d:Ctrl. Bex vs. 9d:Ctrl. DMSO high	1436800	2919600	****	<0,0001
6d:Ctrl. Taz/Bex vs. 9d:Ctrl. DMSO low	1393400	3385000	****	<0,0001
6d:Ctrl. Taz/Bex vs. 9d:Ctrl. DMSO medium	1393400	3084600	****	<0,0001
6d:Ctrl. Taz/Bex vs. 9d:Ctrl. DMSO high	1393400	2919600	****	<0,0001
9d:Ctrl. DMSO low vs. 9d:Ctrl. Taz	3385000	1288600	****	<0,0001
9d:Ctrl. DMSO low vs. 9d:Ctrl. Bex	3385000	1498400	****	<0,0001
9d:Ctrl. DMSO low vs. 9d:Ctrl. Taz/Bex	3385000	1340600	****	<0,0001
9d:Ctrl. DMSO medium vs. 9d:Ctrl. Taz	3084600	1288600	****	<0,0001
9d:Ctrl. DMSO medium vs. 9d:Ctrl. Bex	3084600	1498400	****	<0,0001
9d:Ctrl. DMSO medium vs. 9d:Ctrl. Taz/Bex	3084600	1340600	****	<0,0001
9d:Ctrl. DMSO high vs. 9d:Ctrl. Taz	2919600	1288600	****	<0,0001
9d:Ctrl. DMSO high vs. 9d:Ctrl. Bex	2919600	1498400	****	<0,0001
9d:Ctrl. DMSO high vs. 9d:Ctrl. Taz/Bex	2919600	1340600	****	<0,0001

Appendix table S43: Mean values and significance levels for MSD fibroblast total protein comparison (Appendix fig. S5C, significant differences only)

Comparison	Mean 1	Mean 2	Summary	Adjusted P Value
0d:MSD DMSO low vs. 3d:MSD DMSO medium	99,51	512,1	****	<0,0001
0d:MSD DMSO low vs. 3d:MSD DMSO high	99,51	583,4	****	<0,0001
0d:MSD DMSO low vs. 3d:MSD Taz	99,51	375,5	*	0,012
0d:MSD DMSO low vs. 3d:MSD Bex	99,51	405,2	**	0,0023
0d:MSD DMSO low vs. 6d:MSD DMSO low	99,51	683,8	****	<0,0001
0d:MSD DMSO low vs. 6d:MSD DMSO medium	99,51	648	****	<0,0001
0d:MSD DMSO low vs. 6d:MSD DMSO high	99,51	632,2	****	<0,0001
0d:MSD DMSO low vs. 6d:MSD Taz	99,51	502,1	****	<0,0001
0d:MSD DMSO low vs. 6d:MSD Bex	99,51	497,2	****	<0,0001
0d:MSD DMSO low vs. 6d:MSD Taz/Bex	99,51	373,4	*	0,0134
0d:MSD DMSO low vs. 9d:MSD DMSO low	99,51	811,7	****	<0,0001
0d:MSD DMSO low vs. 9d:MSD DMSO medium	99,51	731,3	****	<0,0001
0d:MSD DMSO low vs. 9d:MSD DMSO high	99,51	737,2	****	<0,0001
0d:MSD DMSO low vs. 9d:MSD Taz	99,51	507,8	****	<0,0001
0d:MSD DMSO low vs. 9d:MSD Bex	99,51	582,8	****	<0,0001
0d:MSD DMSO low vs. 9d:MSD Taz/Bex	99,51	520,4	****	<0,0001
0d:MSD DMSO medium vs. 3d:MSD DMSO medium	181,1	512,1	***	0,0005
0d:MSD DMSO medium vs. 3d:MSD DMSO high	181,1	583,4	****	<0,0001
0d:MSD DMSO medium vs. 6d:MSD DMSO low	181,1	683,8	****	<0,0001
0d:MSD DMSO medium vs. 6d:MSD DMSO medium	181,1	648	****	<0,0001
0d:MSD DMSO medium vs. 6d:MSD DMSO high	181,1	632,2	****	<0,0001
0d:MSD DMSO medium vs. 6d:MSD Taz	181,1	502,1	**	0,0023
0d:MSD DMSO medium vs. 6d:MSD Bex	181,1	497,2	**	0,0012
0d:MSD DMSO medium vs. 9d:MSD DMSO low	181,1	811,7	****	<0,0001
0d:MSD DMSO medium vs. 9d:MSD DMSO medium	181,1	731,3	****	<0,0001
0d:MSD DMSO medium vs. 9d:MSD DMSO high	181,1	737,2	****	<0,0001
0d:MSD DMSO medium vs. 9d:MSD Taz	181,1	507,8	**	0,0016
0d:MSD DMSO medium vs. 9d:MSD Bex	181,1	582,8	****	<0,0001
0d:MSD DMSO medium vs. 9d:MSD Taz/Bex	181,1	520,4	***	0,0003
0d:MSD DMSO high vs. 3d:MSD DMSO high	268,4	583,4	**	0,0013
0d:MSD DMSO high vs. 6d:MSD DMSO low	268,4	683,8	****	<0,0001
0d:MSD DMSO high vs. 6d:MSD DMSO medium	268,4	648	****	<0,0001
0d:MSD DMSO high vs. 6d:MSD DMSO high	268,4	632,2	****	<0,0001
0d:MSD DMSO high vs. 9d:MSD DMSO low	268,4	811,7	****	<0,0001

0d:MSD DMSO high vs. 9d:MSD DMSO medium	268,4	731,3	****	<0,0001
0d:MSD DMSO high vs. 9d:MSD DMSO high	268,4	737,2	****	<0,0001
0d:MSD DMSO high vs. 9d:MSD Bex	268,4	582,8	**	0,0014
0d:MSD DMSO high vs. 9d:MSD Taz/Bex	268,4	520,4	*	0,0396
0d:MSD Taz vs. 3d:MSD DMSO medium	178,4	512,1	***	0,0004
0d:MSD Taz vs. 3d:MSD DMSO high	178,4	583,4	****	<0,0001
0d:MSD Taz vs. 6d:MSD DMSO low	178,4	683,8	****	<0,0001
0d:MSD Taz vs. 6d:MSD DMSO medium	178,4	648	****	<0,0001
0d:MSD Taz vs. 6d:MSD DMSO high	178,4	632,2	****	<0,0001
0d:MSD Taz vs. 6d:MSD Taz	178,4	502,1	**	0,0019
0d:MSD Taz vs. 6d:MSD Bex	178,4	497,2	**	0,0011
0d:MSD Taz vs. 9d:MSD DMSO low	178,4	811,7	****	<0,0001
0d:MSD Taz vs. 9d:MSD DMSO medium	178,4	731,3	****	<0,0001
0d:MSD Taz vs. 9d:MSD DMSO high	178,4	737,2	****	<0,0001
0d:MSD Taz vs. 9d:MSD Taz	178,4	507,8	**	0,0014
0d:MSD Taz vs. 9d:MSD Bex	178,4	582,8	****	<0,0001
0d:MSD Taz vs. 9d:MSD Taz/Bex	178,4	520,4	***	0,0002
0d:MSD Bex vs. 3d:MSD DMSO medium	187,7	512,1	***	0,0008
0d:MSD Bex vs. 3d:MSD DMSO high	187,7	583,4	****	<0,0001
0d:MSD Bex vs. 6d:MSD DMSO low	187,7	683,8	****	<0,0001
0d:MSD Bex vs. 6d:MSD DMSO medium	187,7	648	****	<0,0001
0d:MSD Bex vs. 6d:MSD DMSO high	187,7	632,2	****	<0,0001
0d:MSD Bex vs. 6d:MSD Taz	187,7	502,1	**	0,0032
0d:MSD Bex vs. 6d:MSD Bex	187,7	497,2	**	0,0018
0d:MSD Bex vs. 9d:MSD DMSO low	187,7	811,7	****	<0,0001
0d:MSD Bex vs. 9d:MSD DMSO medium	187,7	731,3	****	<0,0001
0d:MSD Bex vs. 9d:MSD DMSO high	187,7	737,2	****	<0,0001
0d:MSD Bex vs. 9d:MSD Taz	187,7	507,8	**	0,0024
0d:MSD Bex vs. 9d:MSD Bex	187,7	582,8	****	<0,0001
0d:MSD Bex vs. 9d:MSD Taz/Bex	187,7	520,4	***	0,0005
0d:MSD Taz/Bex vs. 3d:MSD DMSO medium	185,9	512,1	***	0,0007
0d:MSD Taz/Bex vs. 3d:MSD DMSO high	185,9	583,4	****	<0,0001
0d:MSD Taz/Bex vs. 6d:MSD DMSO low	185,9	683,8	****	<0,0001
0d:MSD Taz/Bex vs. 6d:MSD DMSO medium	185,9	648	****	<0,0001
0d:MSD Taz/Bex vs. 6d:MSD DMSO high	185,9	632,2	****	<0,0001
0d:MSD Taz/Bex vs. 6d:MSD Taz	185,9	502,1	**	0,0029
0d:MSD Taz/Bex vs. 6d:MSD Bex	185,9	497,2	**	0,0017
0d:MSD Taz/Bex vs. 9d:MSD DMSO low	185,9	811,7	****	<0,0001
0d:MSD Taz/Bex vs. 9d:MSD DMSO medium	185,9	731,3	****	<0,0001
0d:MSD Taz/Bex vs. 9d:MSD DMSO high	185,9	737,2	****	<0,0001
0d:MSD Taz/Bex vs. 9d:MSD Taz	185,9	507,8	**	0,0022
0d:MSD Taz/Bex vs. 9d:MSD Bex	185,9	582,8	****	<0,0001
0d:MSD Taz/Bex vs. 9d:MSD Taz/Bex	185,9	520,4	***	0,0004
3d:MSD DMSO low vs. 3d:MSD DMSO high	300,4	583,4	**	0,0082
3d:MSD DMSO low vs. 6d:MSD DMSO low	300,4	683,8	****	<0,0001
3d:MSD DMSO low vs. 6d:MSD DMSO	300,4	648	***	0,0002

medium				
3d:MSD DMSO low vs. 6d:MSD DMSO high	300,4	632,2	***	0,0005
3d:MSD DMSO low vs. 9d:MSD DMSO low	300,4	811,7	****	<0,0001
3d:MSD DMSO low vs. 9d:MSD DMSO medium	300,4	731,3	****	<0,0001
3d:MSD DMSO low vs. 9d:MSD DMSO high	300,4	737,2	****	<0,0001
3d:MSD DMSO low vs. 9d:MSD Bex	300,4	582,8	**	0,0085
3d:MSD DMSO medium vs. 9d:MSD DMSO low	512,1	811,7	**	0,0033
3d:MSD DMSO high vs. 3d:MSD Taz/Bex	583,4	326,5	*	0,0313
3d:MSD Taz vs. 6d:MSD DMSO low	375,5	683,8	**	0,002
3d:MSD Taz vs. 6d:MSD DMSO medium	375,5	648	*	0,0144
3d:MSD Taz vs. 6d:MSD DMSO high	375,5	632,2	*	0,0317
3d:MSD Taz vs. 9d:MSD DMSO low	375,5	811,7	****	<0,0001
3d:MSD Taz vs. 9d:MSD DMSO medium	375,5	731,3	***	0,0001
3d:MSD Taz vs. 9d:MSD DMSO high	375,5	737,2	****	<0,0001
3d:MSD Bex vs. 6d:MSD DMSO low	405,2	683,8	*	0,0104
3d:MSD Bex vs. 9d:MSD DMSO low	405,2	811,7	****	<0,0001
3d:MSD Bex vs. 9d:MSD DMSO medium	405,2	731,3	***	0,0007
3d:MSD Bex vs. 9d:MSD DMSO high	405,2	737,2	***	0,0005
3d:MSD Taz/Bex vs. 6d:MSD DMSO low	326,5	683,8	****	<0,0001
3d:MSD Taz/Bex vs. 6d:MSD DMSO medium	326,5	648	***	0,0009
3d:MSD Taz/Bex vs. 6d:MSD DMSO high	326,5	632,2	**	0,0023
3d:MSD Taz/Bex vs. 9d:MSD DMSO low	326,5	811,7	****	<0,0001
3d:MSD Taz/Bex vs. 9d:MSD DMSO medium	326,5	731,3	****	<0,0001
3d:MSD Taz/Bex vs. 9d:MSD DMSO high	326,5	737,2	****	<0,0001
3d:MSD Taz/Bex vs. 9d:MSD Bex	326,5	582,8	*	0,0322
6d:MSD DMSO low vs. 6d:MSD Taz/Bex	683,8	373,4	**	0,0017
6d:MSD DMSO medium vs. 6d:MSD Taz/Bex	648	373,4	*	0,0129
6d:MSD DMSO high vs. 6d:MSD Taz/Bex	632,2	373,4	*	0,0286
6d:MSD Taz vs. 9d:MSD DMSO low	502,1	811,7	**	0,0042
6d:MSD Bex vs. 9d:MSD DMSO low	497,2	811,7	**	0,0014
6d:MSD Taz/Bex vs. 9d:MSD DMSO low	373,4	811,7	****	<0,0001
6d:MSD Taz/Bex vs. 9d:MSD DMSO medium	373,4	731,3	****	<0,0001
6d:MSD Taz/Bex vs. 9d:MSD DMSO high	373,4	737,2	****	<0,0001
9d:MSD DMSO low vs. 9d:MSD Taz	811,7	507,8	**	0,0057
9d:MSD DMSO low vs. 9d:MSD Taz/Bex	811,7	520,4	**	0,0052

Appendix table S44: Mean values and significance levels for control fibroblast total protein comparison (Appendix fig. S5D, significant differences only)

**Comparison**	**Mean 1**	**Mean 2**	**Summary**	**Adjusted P Value**
0d:Ctrl. DMSO low vs. 0d:Ctrl. DMSO high	81,28	279,2	***	0,0009
0d:Ctrl. DMSO low vs. 3d:Ctrl. DMSO medium	81,28	291,5	***	0,0002

0d:Ctrl. DMSO low vs. 3d:Ctrl. DMSO high	81,28	340,4	****	<0,0001
0d:Ctrl. DMSO low vs. 6d:Ctrl. DMSO low	81,28	376,2	****	<0,0001
0d:Ctrl. DMSO low vs. 6d:Ctrl. DMSO medium	81,28	397,2	****	<0,0001
0d:Ctrl. DMSO low vs. 6d:Ctrl. DMSO high	81,28	422,7	****	<0,0001
0d:Ctrl. DMSO low vs. 6d:Ctrl. Bex	81,28	316,4	****	<0,0001
0d:Ctrl. DMSO low vs. 9d:Ctrl. DMSO low	81,28	488,7	****	<0,0001
0d:Ctrl. DMSO low vs. 9d:Ctrl. DMSO medium	81,28	501,3	****	<0,0001
0d:Ctrl. DMSO low vs. 9d:Ctrl. DMSO high	81,28	563,2	****	<0,0001
0d:Ctrl. DMSO low vs. 9d:Ctrl. Taz	81,28	251,4	*	0,0215
0d:Ctrl. DMSO low vs. 9d:Ctrl. Bex	81,28	373,4	****	<0,0001
0d:Ctrl. DMSO low vs. 9d:Ctrl. Taz/Bex	81,28	251,7	*	0,0209
0d:Ctrl. DMSO medium vs. 3d:Ctrl. DMSO high	150,9	340,4	**	0,0019
0d:Ctrl. DMSO medium vs. 6d:Ctrl. DMSO low	150,9	376,2	****	<0,0001
0d:Ctrl. DMSO medium vs. 6d:Ctrl. DMSO medium	150,9	397,2	****	<0,0001
0d:Ctrl. DMSO medium vs. 6d:Ctrl. DMSO high	150,9	422,7	****	<0,0001
0d:Ctrl. DMSO medium vs. 6d:Ctrl. Bex	150,9	316,4	*	0,0303
0d:Ctrl. DMSO medium vs. 9d:Ctrl. DMSO low	150,9	488,7	****	<0,0001
0d:Ctrl. DMSO medium vs. 9d:Ctrl. DMSO medium	150,9	501,3	****	<0,0001
0d:Ctrl. DMSO medium vs. 9d:Ctrl. DMSO high	150,9	563,2	****	<0,0001
0d:Ctrl. DMSO medium vs. 9d:Ctrl. Bex	150,9	373,4	***	0,0002
0d:Ctrl. DMSO high vs. 9d:Ctrl. DMSO low	279,2	488,7	***	0,0003
0d:Ctrl. DMSO high vs. 9d:Ctrl. DMSO medium	279,2	501,3	****	<0,0001
0d:Ctrl. DMSO high vs. 9d:Ctrl. DMSO high	279,2	563,2	****	<0,0001
0d:Ctrl. Taz vs. 3d:Ctrl. DMSO high	149,4	340,4	**	0,0038
0d:Ctrl. Taz vs. 6d:Ctrl. DMSO low	149,4	376,2	***	0,0001
0d:Ctrl. Taz vs. 6d:Ctrl. DMSO medium	149,4	397,2	****	<0,0001
0d:Ctrl. Taz vs. 6d:Ctrl. DMSO high	149,4	422,7	****	<0,0001
0d:Ctrl. Taz vs. 6d:Ctrl. Bex	149,4	316,4	*	0,0457
0d:Ctrl. Taz vs. 9d:Ctrl. DMSO low	149,4	488,7	****	<0,0001
0d:Ctrl. Taz vs. 9d:Ctrl. DMSO medium	149,4	501,3	****	<0,0001
0d:Ctrl. Taz vs. 9d:Ctrl. DMSO high	149,4	563,2	****	<0,0001
0d:Ctrl. Taz vs. 9d:Ctrl. Bex	149,4	373,4	***	0,0004
0d:Ctrl. Bex vs. 3d:Ctrl. DMSO high	159,6	340,4	**	0,0091
0d:Ctrl. Bex vs. 6d:Ctrl. DMSO low	159,6	376,2	***	0,0004
0d:Ctrl. Bex vs. 6d:Ctrl. DMSO medium	159,6	397,2	****	<0,0001
0d:Ctrl. Bex vs. 6d:Ctrl. DMSO high	159,6	422,7	****	<0,0001
0d:Ctrl. Bex vs. 9d:Ctrl. DMSO low	159,6	488,7	****	<0,0001
0d:Ctrl. Bex vs. 9d:Ctrl. DMSO medium	159,6	501,3	****	<0,0001
0d:Ctrl. Bex vs. 9d:Ctrl. DMSO high	159,6	563,2	****	<0,0001
0d:Ctrl. Bex vs. 9d:Ctrl. Bex	159,6	373,4	**	0,0011
0d:Ctrl. Taz/Bex vs. 3d:Ctrl. DMSO high	154,4	340,4	**	0,0059
0d:Ctrl. Taz/Bex vs. 6d:Ctrl. DMSO low	154,4	376,2	***	0,0002
0d:Ctrl. Taz/Bex vs. 6d:Ctrl. DMSO medium	154,4	397,2	****	<0,0001
0d:Ctrl. Taz/Bex vs. 6d:Ctrl. DMSO high	154,4	422,7	****	<0,0001
0d:Ctrl. Taz/Bex vs. 9d:Ctrl. DMSO low	154,4	488,7	****	<0,0001

0d:Ctrl. Taz/Bex vs. 9d:Ctrl. DMSO medium	154,4	501,3	****	<0,0001
0d:Ctrl. Taz/Bex vs. 9d:Ctrl. DMSO high	154,4	563,2	****	<0,0001
0d:Ctrl. Taz/Bex vs. 9d:Ctrl. Bex	154,4	373,4	***	0,0007
3d:Ctrl. DMSO low vs. 6d:Ctrl. DMSO low	200,4	376,2	**	0,0068
3d:Ctrl. DMSO low vs. 6d:Ctrl. DMSO medium	200,4	397,2	***	0,001
3d:Ctrl. DMSO low vs. 6d:Ctrl. DMSO high	200,4	422,7	****	<0,0001
3d:Ctrl. DMSO low vs. 9d:Ctrl. DMSO low	200,4	488,7	****	<0,0001
3d:Ctrl. DMSO low vs. 9d:Ctrl. DMSO medium	200,4	501,3	****	<0,0001
3d:Ctrl. DMSO low vs. 9d:Ctrl. DMSO high	200,4	563,2	****	<0,0001
3d:Ctrl. DMSO low vs. 9d:Ctrl. Bex	200,4	373,4	*	0,0172
3d:Ctrl. DMSO medium vs. 9d:Ctrl. DMSO low	291,5	488,7	***	0,0009
3d:Ctrl. DMSO medium vs. 9d:Ctrl. DMSO medium	291,5	501,3	***	0,0003
3d:Ctrl. DMSO medium vs. 9d:Ctrl. DMSO high	291,5	563,2	****	<0,0001
3d:Ctrl. DMSO high vs. 9d:Ctrl. DMSO medium	340,4	501,3	*	0,024
3d:Ctrl. DMSO high vs. 9d:Ctrl. DMSO high	340,4	563,2	****	<0,0001
3d:Ctrl. Taz vs. 6d:Ctrl. DMSO low	205,3	376,2	*	0,0201
3d:Ctrl. Taz vs. 6d:Ctrl. DMSO medium	205,3	397,2	**	0,0036
3d:Ctrl. Taz vs. 6d:Ctrl. DMSO high	205,3	422,7	***	0,0003
3d:Ctrl. Taz vs. 9d:Ctrl. DMSO low	205,3	488,7	****	<0,0001
3d:Ctrl. Taz vs. 9d:Ctrl. DMSO medium	205,3	501,3	****	<0,0001
3d:Ctrl. Taz vs. 9d:Ctrl. DMSO high	205,3	563,2	****	<0,0001
3d:Ctrl. Taz vs. 9d:Ctrl. Bex	205,3	373,4	*	0,0424
3d:Ctrl. Bex vs. 6d:Ctrl. DMSO medium	238,1	397,2	*	0,0488
3d:Ctrl. Bex vs. 6d:Ctrl. DMSO high	238,1	422,7	**	0,0067
3d:Ctrl. Bex vs. 9d:Ctrl. DMSO low	238,1	488,7	****	<0,0001
3d:Ctrl. Bex vs. 9d:Ctrl. DMSO medium	238,1	501,3	****	<0,0001
3d:Ctrl. Bex vs. 9d:Ctrl. DMSO high	238,1	563,2	****	<0,0001
3d:Ctrl. Taz/Bex vs. 6d:Ctrl. DMSO low	190,1	376,2	**	0,0058
3d:Ctrl. Taz/Bex vs. 6d:Ctrl. DMSO medium	190,1	397,2	***	0,0009
3d:Ctrl. Taz/Bex vs. 6d:Ctrl. DMSO high	190,1	422,7	****	<0,0001
3d:Ctrl. Taz/Bex vs. 9d:Ctrl. DMSO low	190,1	488,7	****	<0,0001
3d:Ctrl. Taz/Bex vs. 9d:Ctrl. DMSO medium	190,1	501,3	****	<0,0001
3d:Ctrl. Taz/Bex vs. 9d:Ctrl. DMSO high	190,1	563,2	****	<0,0001
3d:Ctrl. Taz/Bex vs. 9d:Ctrl. Bex	190,1	373,4	*	0,0141
6d:Ctrl. DMSO low vs. 9d:Ctrl. DMSO high	376,2	563,2	**	0,0025
6d:Ctrl. DMSO medium vs. 6d:Ctrl. Taz	397,2	236,8	*	0,0444
6d:Ctrl. DMSO medium vs. 6d:Ctrl. Taz/Bex	397,2	228,3	*	0,0236
6d:Ctrl. DMSO medium vs. 9d:Ctrl. DMSO high	397,2	563,2	*	0,0157
6d:Ctrl. DMSO high vs. 6d:Ctrl. Taz	422,7	236,8	**	0,006
6d:Ctrl. DMSO high vs. 6d:Ctrl. Taz/Bex	422,7	228,3	**	0,0029
6d:Ctrl. DMSO high vs. 9d:Ctrl. Taz	422,7	251,4	*	0,0196
6d:Ctrl. DMSO high vs. 9d:Ctrl. Taz/Bex	422,7	251,7	*	0,0202
6d:Ctrl. Taz vs. 9d:Ctrl. DMSO low	236,8	488,7	****	<0,0001
6d:Ctrl. Taz vs. 9d:Ctrl. DMSO medium	236,8	501,3	****	<0,0001
6d:Ctrl. Taz vs. 9d:Ctrl. DMSO high	236,8	563,2	****	<0,0001
6d:Ctrl. Bex vs. 9d:Ctrl. DMSO low	316,4	488,7	*	0,0181

6d:Ctrl. Bex vs. 9d:Ctrl. DMSO medium	316,4	501,3	**	0,0065
6d:Ctrl. Bex vs. 9d:Ctrl. DMSO high	316,4	563,2	****	<0,0001
6d:Ctrl. Taz/Bex vs. 9d:Ctrl. DMSO low	228,3	488,7	****	<0,0001
6d:Ctrl. Taz/Bex vs. 9d:Ctrl. DMSO medium	228,3	501,3	****	<0,0001
6d:Ctrl. Taz/Bex vs. 9d:Ctrl. DMSO high	228,3	563,2	****	<0,0001
9d:Ctrl. DMSO low vs. 9d:Ctrl. Taz	488,7	251,4	****	<0,0001
9d:Ctrl. DMSO low vs. 9d:Ctrl. Taz/Bex	488,7	251,7	****	<0,0001
9d:Ctrl. DMSO medium vs. 9d:Ctrl. Taz	501,3	251,4	****	<0,0001
9d:Ctrl. DMSO medium vs. 9d:Ctrl. Taz/Bex	501,3	251,7	****	<0,0001
9d:Ctrl. DMSO high vs. 9d:Ctrl. Taz	563,2	251,4	****	<0,0001
9d:Ctrl. DMSO high vs. 9d:Ctrl. Bex	563,2	373,4	**	0,0043
9d:Ctrl. DMSO high vs. 9d:Ctrl. Taz/Bex	563,2	251,7	****	<0,0001

Appendix table S45: Mean values and significance levels for MSD fibroblast ARSA activity comparison (Appendix fig. S5E, significant differences only)

**Comparison**	**Mean 1**	**Mean 2**	**Summary**	**Adjusted P Value**
0d:MSD DMSO low vs. 3d:MSD Taz/Bex	10,36	30,16	*	0,0159
0d:MSD DMSO low vs. 6d:MSD Taz	10,36	34,32	***	0,0006
0d:MSD DMSO low vs. 6d:MSD Taz/Bex	10,36	105,6	****	<0,0001
0d:MSD DMSO low vs. 9d:MSD Taz	10,36	73,39	****	<0,0001
0d:MSD DMSO low vs. 9d:MSD Taz/Bex	10,36	130,8	****	<0,0001
0d:MSD DMSO medium vs. 6d:MSD Taz	12,1	34,32	**	0,0026
0d:MSD DMSO medium vs. 6d:MSD Taz/Bex	12,1	105,6	****	<0,0001
0d:MSD DMSO medium vs. 9d:MSD Taz	12,1	73,39	****	<0,0001
0d:MSD DMSO medium vs. 9d:MSD Taz/Bex	12,1	130,8	****	<0,0001
0d:MSD DMSO high vs. 6d:MSD Taz	12,28	34,32	**	0,003
0d:MSD DMSO high vs. 6d:MSD Taz/Bex	12,28	105,6	****	<0,0001
0d:MSD DMSO high vs. 9d:MSD Taz	12,28	73,39	****	<0,0001
0d:MSD DMSO high vs. 9d:MSD Taz/Bex	12,28	130,8	****	<0,0001
0d:MSD Taz vs. 3d:MSD Taz/Bex	11,59	30,16	*	0,0363
0d:MSD Taz vs. 6d:MSD Taz	11,59	34,32	**	0,0017
0d:MSD Taz vs. 6d:MSD Taz/Bex	11,59	105,6	****	<0,0001
0d:MSD Taz vs. 9d:MSD Taz	11,59	73,39	****	<0,0001
0d:MSD Taz vs. 9d:MSD Taz/Bex	11,59	130,8	****	<0,0001
0d:MSD Bex vs. 3d:MSD Taz/Bex	11,23	30,16	*	0,0287
0d:MSD Bex vs. 6d:MSD Taz	11,23	34,32	**	0,0013
0d:MSD Bex vs. 6d:MSD Taz/Bex	11,23	105,6	****	<0,0001
0d:MSD Bex vs. 9d:MSD Taz	11,23	73,39	****	<0,0001
0d:MSD Bex vs. 9d:MSD Taz/Bex	11,23	130,8	****	<0,0001
0d:MSD Taz/Bex vs. 3d:MSD Taz/Bex	9,065	30,16	**	0,0062
0d:MSD Taz/Bex vs. 6d:MSD Taz	9,065	34,32	***	0,0002
0d:MSD Taz/Bex vs. 6d:MSD Taz/Bex	9,065	105,6	****	<0,0001
0d:MSD Taz/Bex vs. 9d:MSD Taz	9,065	73,39	****	<0,0001

0d:MSD Taz/Bex vs. 9d:MSD Taz/Bex	9,065	130,8	****	<0,0001
3d:MSD DMSO low vs. 3d:MSD Taz/Bex	5,343	30,16	***	0,0003
3d:MSD DMSO low vs. 6d:MSD Taz	5,343	34,32	****	<0,0001
3d:MSD DMSO low vs. 6d:MSD Taz/Bex	5,343	105,6	****	<0,0001
3d:MSD DMSO low vs. 9d:MSD Taz	5,343	73,39	****	<0,0001
3d:MSD DMSO low vs. 9d:MSD Bex	5,343	24,37	*	0,027
3d:MSD DMSO low vs. 9d:MSD Taz/Bex	5,343	130,8	****	<0,0001
3d:MSD DMSO medium vs. 3d:MSD Taz/Bex	7,02	30,16	**	0,0012
3d:MSD DMSO medium vs. 6d:MSD Taz	7,02	34,32	****	<0,0001
3d:MSD DMSO medium vs. 6d:MSD Taz/Bex	7,02	105,6	****	<0,0001
3d:MSD DMSO medium vs. 9d:MSD Taz	7,02	73,39	****	<0,0001
3d:MSD DMSO medium vs. 9d:MSD Taz/Bex	7,02	130,8	****	<0,0001
3d:MSD DMSO high vs. 3d:MSD Taz/Bex	9,503	30,16	**	0,0086
3d:MSD DMSO high vs. 6d:MSD Taz	9,503	34,32	***	0,0003
3d:MSD DMSO high vs. 6d:MSD Taz/Bex	9,503	105,6	****	<0,0001
3d:MSD DMSO high vs. 9d:MSD Taz	9,503	73,39	****	<0,0001
3d:MSD DMSO high vs. 9d:MSD Taz/Bex	9,503	130,8	****	<0,0001
3d:MSD Taz vs. 3d:MSD Taz/Bex	9,762	30,16	*	0,0103
3d:MSD Taz vs. 6d:MSD Taz	9,762	34,32	***	0,0004
3d:MSD Taz vs. 6d:MSD Taz/Bex	9,762	105,6	****	<0,0001
3d:MSD Taz vs. 9d:MSD Taz	9,762	73,39	****	<0,0001
3d:MSD Taz vs. 9d:MSD Taz/Bex	9,762	130,8	****	<0,0001
3d:MSD Bex vs. 3d:MSD Taz/Bex	10,12	30,16	*	0,0134
3d:MSD Bex vs. 6d:MSD Taz	10,12	34,32	***	0,0005
3d:MSD Bex vs. 6d:MSD Taz/Bex	10,12	105,6	****	<0,0001
3d:MSD Bex vs. 9d:MSD Taz	10,12	73,39	****	<0,0001
3d:MSD Bex vs. 9d:MSD Taz/Bex	10,12	130,8	****	<0,0001
3d:MSD Taz/Bex vs. 6d:MSD DMSO low	30,16	7,14	**	0,0014
3d:MSD Taz/Bex vs. 6d:MSD DMSO medium	30,16	11,21	*	0,0284
3d:MSD Taz/Bex vs. 6d:MSD Taz/Bex	30,16	105,6	****	<0,0001
3d:MSD Taz/Bex vs. 9d:MSD Taz	30,16	73,39	****	<0,0001
3d:MSD Taz/Bex vs. 9d:MSD Taz/Bex	30,16	130,8	****	<0,0001
6d:MSD DMSO low vs. 6d:MSD Taz	7,14	34,32	****	<0,0001
6d:MSD DMSO low vs. 6d:MSD Taz/Bex	7,14	105,6	****	<0,0001
6d:MSD DMSO low vs. 9d:MSD Taz	7,14	73,39	****	<0,0001
6d:MSD DMSO low vs. 9d:MSD Taz/Bex	7,14	130,8	****	<0,0001
6d:MSD DMSO medium vs. 6d:MSD Taz	11,21	34,32	**	0,0013
6d:MSD DMSO medium vs. 6d:MSD Taz/Bex	11,21	105,6	****	<0,0001
6d:MSD DMSO medium vs. 9d:MSD Taz	11,21	73,39	****	<0,0001
6d:MSD DMSO medium vs. 9d:MSD Taz/Bex	11,21	130,8	****	<0,0001
6d:MSD DMSO high vs. 6d:MSD Taz	13,55	34,32	**	0,0079
6d:MSD DMSO high vs. 6d:MSD Taz/Bex	13,55	105,6	****	<0,0001
6d:MSD DMSO high vs. 9d:MSD Taz	13,55	73,39	****	<0,0001
6d:MSD DMSO high vs. 9d:MSD Taz/Bex	13,55	130,8	****	<0,0001
6d:MSD Taz vs. 6d:MSD Taz/Bex	34,32	105,6	****	<0,0001
6d:MSD Taz vs. 9d:MSD DMSO low	34,32	13,27	**	0,0064
6d:MSD Taz vs. 9d:MSD DMSO medium	34,32	16,23	*	0,0493

6d:MSD Taz vs. 9d:MSD Taz	34,32	73,39	****	<0,0001
6d:MSD Taz vs. 9d:MSD Taz/Bex	34,32	130,8	****	<0,0001
6d:MSD Bex vs. 6d:MSD Taz/Bex	16,87	105,6	****	<0,0001
6d:MSD Bex vs. 9d:MSD Taz	16,87	73,39	****	<0,0001
6d:MSD Bex vs. 9d:MSD Taz/Bex	16,87	130,8	****	<0,0001
6d:MSD Taz/Bex vs. 9d:MSD DMSO low	105,6	13,27	****	<0,0001
6d:MSD Taz/Bex vs. 9d:MSD DMSO medium	105,6	16,23	****	<0,0001
6d:MSD Taz/Bex vs. 9d:MSD DMSO high	105,6	16,67	****	<0,0001
6d:MSD Taz/Bex vs. 9d:MSD Taz	105,6	73,39	****	<0,0001
6d:MSD Taz/Bex vs. 9d:MSD Bex	105,6	24,37	****	<0,0001
6d:MSD Taz/Bex vs. 9d:MSD Taz/Bex	105,6	130,8	***	0,0002
9d:MSD DMSO low vs. 9d:MSD Taz	13,27	73,39	****	<0,0001
9d:MSD DMSO low vs. 9d:MSD Taz/Bex	13,27	130,8	****	<0,0001
9d:MSD DMSO medium vs. 9d:MSD Taz	16,23	73,39	****	<0,0001
9d:MSD DMSO medium vs. 9d:MSD Taz/Bex	16,23	130,8	****	<0,0001
9d:MSD DMSO high vs. 9d:MSD Taz	16,67	73,39	****	<0,0001
9d:MSD DMSO high vs. 9d:MSD Taz/Bex	16,67	130,8	****	<0,0001
9d:MSD Taz vs. 9d:MSD Bex	73,39	24,37	****	<0,0001
9d:MSD Taz vs. 9d:MSD Taz/Bex	73,39	130,8	****	<0,0001
9d:MSD Bex vs. 9d:MSD Taz/Bex	24,37	130,8	****	<0,0001

Appendix table S46: Mean values and significance levels for control fibroblast ARSA activity comparison (Appendix fig. S5F, significant differences only)

**Comparison**	**Mean 1**	**Mean 2**	**Summary**	**Adjusted P Value**
0d:Ctrl. DMSO low vs. 6d:Ctrl. DMSO high	654,9	1102	****	<0,0001
0d:Ctrl. DMSO low vs. 6d:Ctrl. Taz	654,9	1171	****	<0,0001
0d:Ctrl. DMSO low vs. 6d:Ctrl. Taz/Bex	654,9	1124	****	<0,0001
0d:Ctrl. DMSO low vs. 9d:Ctrl. DMSO medium	654,9	1040	**	0,0019
0d:Ctrl. DMSO low vs. 9d:Ctrl. DMSO high	654,9	1147	****	<0,0001
0d:Ctrl. DMSO low vs. 9d:Ctrl. Taz	654,9	1271	****	<0,0001
0d:Ctrl. DMSO low vs. 9d:Ctrl. Bex	654,9	1087	***	0,0005
0d:Ctrl. DMSO low vs. 9d:Ctrl. Taz/Bex	654,9	1244	****	<0,0001
0d:Ctrl. DMSO medium vs. 6d:Ctrl. DMSO high	679,3	1102	***	0,0003
0d:Ctrl. DMSO medium vs. 6d:Ctrl. Taz	679,3	1171	****	<0,0001
0d:Ctrl. DMSO medium vs. 6d:Ctrl. Taz/Bex	679,3	1124	***	0,0003
0d:Ctrl. DMSO medium vs. 9d:Ctrl. DMSO medium	679,3	1040	**	0,0059
0d:Ctrl. DMSO medium vs. 9d:Ctrl. DMSO high	679,3	1147	****	<0,0001
0d:Ctrl. DMSO medium vs. 9d:Ctrl. Taz	679,3	1271	****	<0,0001
0d:Ctrl. DMSO medium vs. 9d:Ctrl. Bex	679,3	1087	**	0,0017
0d:Ctrl. DMSO medium vs. 9d:Ctrl. Taz/Bex	679,3	1244	****	<0,0001
0d:Ctrl. DMSO high vs. 6d:Ctrl. DMSO high	680,6	1102	***	0,0003
0d:Ctrl. DMSO high vs. 6d:Ctrl. Taz	680,6	1171	****	<0,0001
0d:Ctrl. DMSO high vs. 6d:Ctrl. Taz/Bex	680,6	1124	***	0,0003

0d:Ctrl. DMSO high vs. 9d:Ctrl. DMSO medium	680,6	1040	**	0,0063
0d:Ctrl. DMSO high vs. 9d:Ctrl. DMSO high	680,6	1147	****	<0,0001
0d:Ctrl. DMSO high vs. 9d:Ctrl. Taz	680,6	1271	****	<0,0001
0d:Ctrl. DMSO high vs. 9d:Ctrl. Bex	680,6	1087	**	0,0018
0d:Ctrl. DMSO high vs. 9d:Ctrl. Taz/Bex	680,6	1244	****	<0,0001
0d:Ctrl. Taz vs. 3d:Ctrl. Taz	609,6	921	*	0,0437
0d:Ctrl. Taz vs. 6d:Ctrl. DMSO medium	609,6	927,9	*	0,0338
0d:Ctrl. Taz vs. 6d:Ctrl. DMSO high	609,6	1102	****	<0,0001
0d:Ctrl. Taz vs. 6d:Ctrl. Taz	609,6	1171	****	<0,0001
0d:Ctrl. Taz vs. 6d:Ctrl. Bex	609,6	935,8	*	0,0444
0d:Ctrl. Taz vs. 6d:Ctrl. Taz/Bex	609,6	1124	****	<0,0001
0d:Ctrl. Taz vs. 9d:Ctrl. DMSO low	609,6	922,1	*	0,042
0d:Ctrl. Taz vs. 9d:Ctrl. DMSO medium	609,6	1040	***	0,0002
0d:Ctrl. Taz vs. 9d:Ctrl. DMSO high	609,6	1147	****	<0,0001
0d:Ctrl. Taz vs. 9d:Ctrl. Taz	609,6	1271	****	<0,0001
0d:Ctrl. Taz vs. 9d:Ctrl. Bex	609,6	1087	****	<0,0001
0d:Ctrl. Taz vs. 9d:Ctrl. Taz/Bex	609,6	1244	****	<0,0001
0d:Ctrl. Bex vs. 6d:Ctrl. DMSO high	664,4	1102	***	0,0004
0d:Ctrl. Bex vs. 6d:Ctrl. Taz	664,4	1171	****	<0,0001
0d:Ctrl. Bex vs. 6d:Ctrl. Taz/Bex	664,4	1124	***	0,0004
0d:Ctrl. Bex vs. 9d:Ctrl. DMSO medium	664,4	1040	**	0,0066
0d:Ctrl. Bex vs. 9d:Ctrl. DMSO high	664,4	1147	****	<0,0001
0d:Ctrl. Bex vs. 9d:Ctrl. Taz	664,4	1271	****	<0,0001
0d:Ctrl. Bex vs. 9d:Ctrl. Bex	664,4	1087	**	0,0019
0d:Ctrl. Bex vs. 9d:Ctrl. Taz/Bex	664,4	1244	****	<0,0001
0d:Ctrl. Taz/Bex vs. 6d:Ctrl. DMSO high	702,5	1102	**	0,0024
0d:Ctrl. Taz/Bex vs. 6d:Ctrl. Taz	702,5	1171	***	0,0002
0d:Ctrl. Taz/Bex vs. 6d:Ctrl. Taz/Bex	702,5	1124	**	0,002
0d:Ctrl. Taz/Bex vs. 9d:Ctrl. DMSO medium	702,5	1040	*	0,0294
0d:Ctrl. Taz/Bex vs. 9d:Ctrl. DMSO high	702,5	1147	***	0,0003
0d:Ctrl. Taz/Bex vs. 9d:Ctrl. Taz	702,5	1271	****	<0,0001
0d:Ctrl. Taz/Bex vs. 9d:Ctrl. Bex	702,5	1087	**	0,0091
0d:Ctrl. Taz/Bex vs. 9d:Ctrl. Taz/Bex	702,5	1244	****	<0,0001
3d:Ctrl. DMSO low vs. 3d:Ctrl. DMSO high	562,8	913,5	**	0,0091
3d:Ctrl. DMSO low vs. 3d:Ctrl. Taz	562,8	921	**	0,0066
3d:Ctrl. DMSO low vs. 6d:Ctrl. DMSO medium	562,8	927,9	**	0,0049
3d:Ctrl. DMSO low vs. 6d:Ctrl. DMSO high	562,8	1102	****	<0,0001
3d:Ctrl. DMSO low vs. 6d:Ctrl. Taz	562,8	1171	****	<0,0001
3d:Ctrl. DMSO low vs. 6d:Ctrl. Bex	562,8	935,8	**	0,0074
3d:Ctrl. DMSO low vs. 6d:Ctrl. Taz/Bex	562,8	1124	****	<0,0001
3d:Ctrl. DMSO low vs. 9d:Ctrl. DMSO low	562,8	922,1	**	0,0063
3d:Ctrl. DMSO low vs. 9d:Ctrl. DMSO medium	562,8	1040	****	<0,0001
3d:Ctrl. DMSO low vs. 9d:Ctrl. DMSO high	562,8	1147	****	<0,0001
3d:Ctrl. DMSO low vs. 9d:Ctrl. Taz	562,8	1271	****	<0,0001
3d:Ctrl. DMSO low vs. 9d:Ctrl. Bex	562,8	1087	****	<0,0001
3d:Ctrl. DMSO low vs. 9d:Ctrl. Taz/Bex	562,8	1244	****	<0,0001
3d:Ctrl. DMSO medium vs. 6d:Ctrl. DMSO high	762,2	1102	*	0,0143

3d:Ctrl. DMSO medium vs. 6d:Ctrl. Taz	762,2	1171	**	0,0016
3d:Ctrl. DMSO medium vs. 6d:Ctrl. Taz/Bex	762,2	1124	*	0,0116
3d:Ctrl. DMSO medium vs. 9d:Ctrl. DMSO high	762,2	1147	**	0,002
3d:Ctrl. DMSO medium vs. 9d:Ctrl. Taz	762,2	1271	****	<0,0001
3d:Ctrl. DMSO medium vs. 9d:Ctrl. Bex	762,2	1087	*	0,0472
3d:Ctrl. DMSO medium vs. 9d:Ctrl. Taz/Bex	762,2	1244	****	<0,0001
3d:Ctrl. DMSO high vs. 9d:Ctrl. Taz	913,5	1271	*	0,0138
3d:Ctrl. DMSO high vs. 9d:Ctrl. Taz/Bex	913,5	1244	*	0,038
3d:Ctrl. Taz vs. 9d:Ctrl. Taz	921	1271	*	0,0185
3d:Ctrl. Taz vs. 9d:Ctrl. Taz/Bex	921	1244	*	0,0495
3d:Ctrl. Bex vs. 6d:Ctrl. DMSO high	751,4	1102	*	0,0179
3d:Ctrl. Bex vs. 6d:Ctrl. Taz	751,4	1171	**	0,0022
3d:Ctrl. Bex vs. 6d:Ctrl. Taz/Bex	751,4	1124	*	0,0142
3d:Ctrl. Bex vs. 9d:Ctrl. DMSO high	751,4	1147	**	0,0028
3d:Ctrl. Bex vs. 9d:Ctrl. Taz	751,4	1271	****	<0,0001
3d:Ctrl. Bex vs. 9d:Ctrl. Taz/Bex	751,4	1244	****	<0,0001
3d:Ctrl. Taz/Bex vs. 9d:Ctrl. Taz	836,1	1271	**	0,0011
3d:Ctrl. Taz/Bex vs. 9d:Ctrl. Taz/Bex	836,1	1244	**	0,0035
6d:Ctrl. DMSO low vs. 6d:Ctrl. DMSO high	699	1102	***	0,0008
6d:Ctrl. DMSO low vs. 6d:Ctrl. Taz	699	1171	****	<0,0001
6d:Ctrl. DMSO low vs. 6d:Ctrl. Taz/Bex	699	1124	***	0,0007
6d:Ctrl. DMSO low vs. 9d:Ctrl. DMSO medium	699	1040	*	0,0137
6d:Ctrl. DMSO low vs. 9d:Ctrl. DMSO high	699	1147	****	<0,0001
6d:Ctrl. DMSO low vs. 9d:Ctrl. Taz	699	1271	****	<0,0001
6d:Ctrl. DMSO low vs. 9d:Ctrl. Bex	699	1087	**	0,004
6d:Ctrl. DMSO low vs. 9d:Ctrl. Taz/Bex	699	1244	****	<0,0001
6d:Ctrl. DMSO medium vs. 9d:Ctrl. Taz	927,9	1271	*	0,0241
9d:Ctrl. DMSO low vs. 9d:Ctrl. Taz	922,1	1271	*	0,0193
