# Supplementary figures and images for: Drug screening identifies tazarotene and bexarotene as therapeutic agents in multiple sulfatase deficiency
Source: EMBO Mol Med. 2023 Feb 15;15(3):e14837. doi: 10.15252/emmm.202114837 (PMC9994482; doi:10.15252/emmm.202114837)

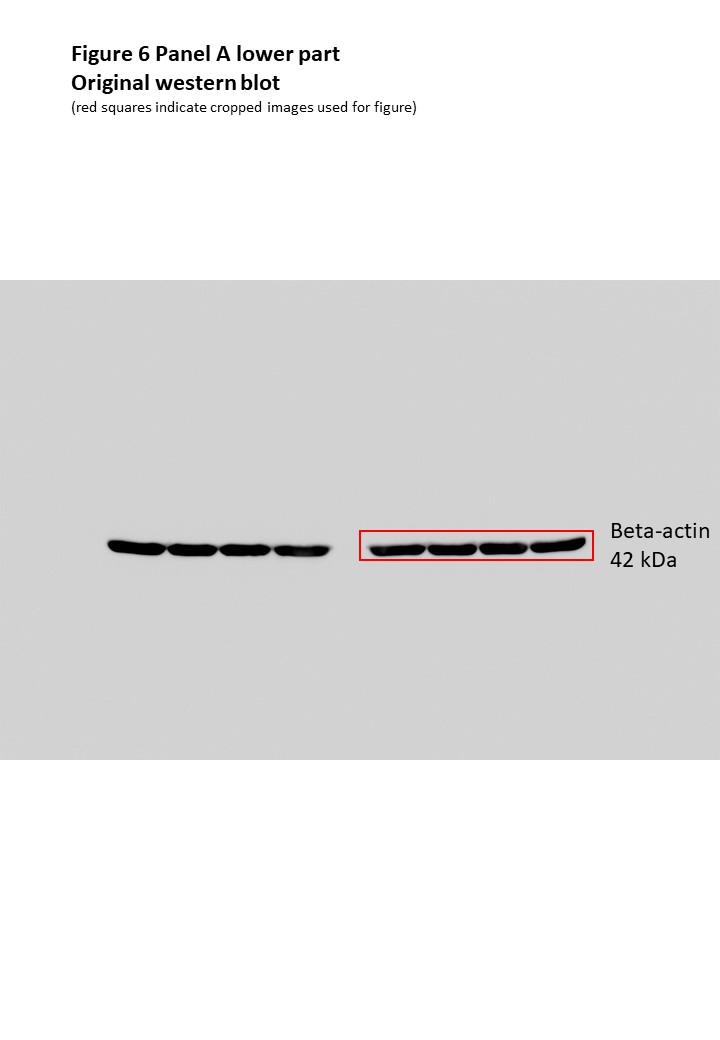

Supplement: Supplementary file 12 — Source Data for Figure 6 [file EMMM-15-e14837-s002.zip › Source data Fig 6/Fig 6 panel A lower part original western blot.JPG]

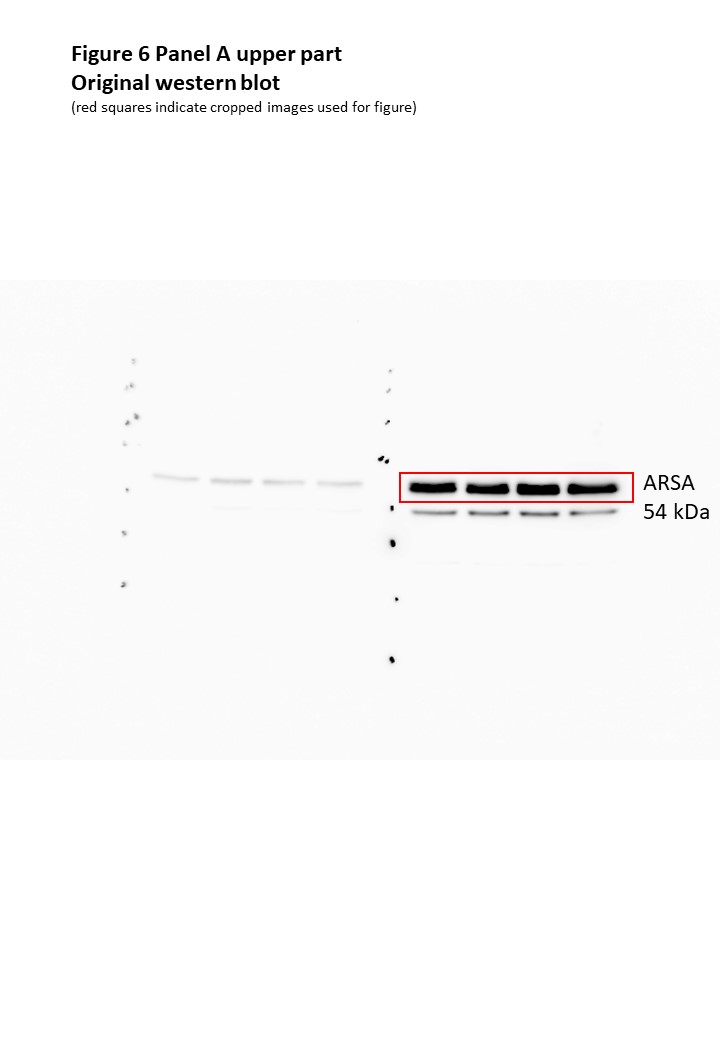

Supplement: Supplementary file 12 — Source Data for Figure 6 [file EMMM-15-e14837-s002.zip › Source data Fig 6/Fig 6 panel A upper part original western blot.JPG]

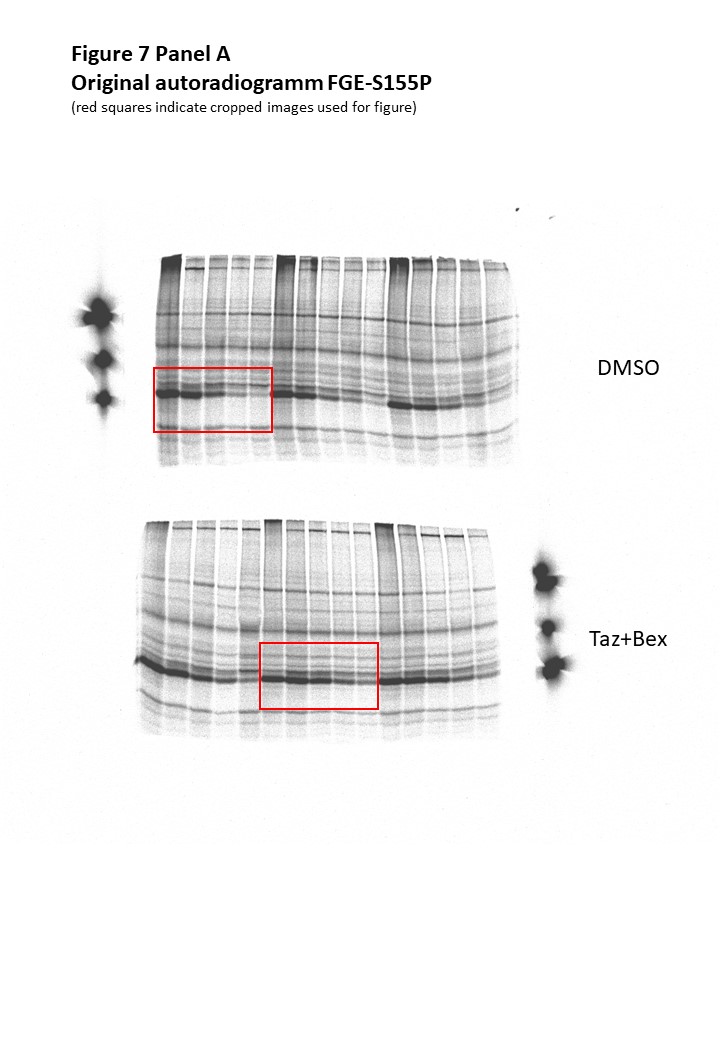

Supplement: Supplementary file 13 — Source Data for Figure 7 [file EMMM-15-e14837-s001.zip › Source data Fig 7/Original autoradiogrammFig 7 panel A.JPG]

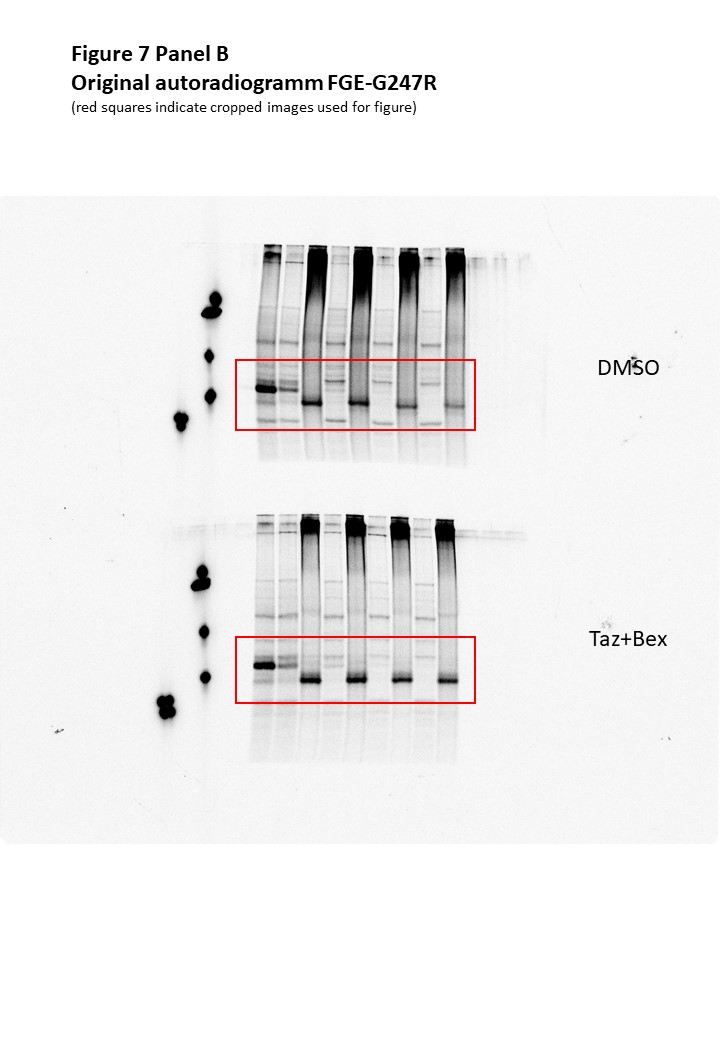

Supplement: Supplementary file 13 — Source Data for Figure 7 [file EMMM-15-e14837-s001.zip › Source data Fig 7/Original autoradiogrammFig 7 panel B.JPG]

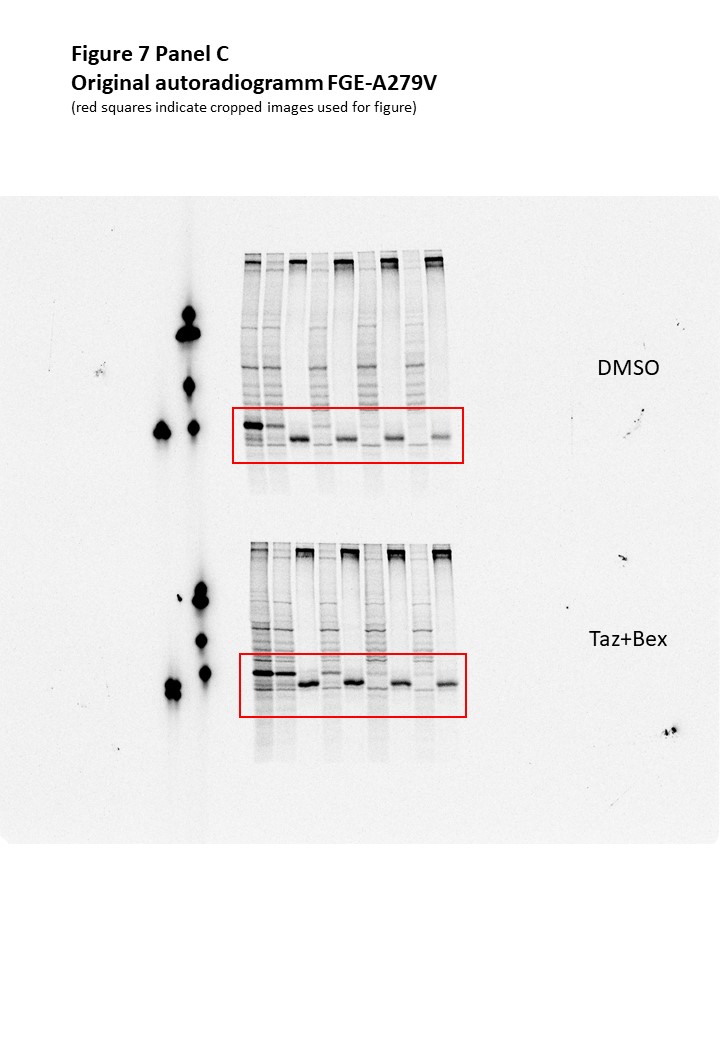

Supplement: Supplementary file 13 — Source Data for Figure 7 [file EMMM-15-e14837-s001.zip › Source data Fig 7/Original autoradiogrammFig 7 panel C.JPG]
