# Supplementary material for: Drug screening identifies tazarotene and bexarotene as therapeutic agents in multiple sulfatase deficiency
Source: EMBO Mol Med. 2023 Feb 15;15(3):e14837. doi: 10.15252/emmm.202114837 (PMC9994482; doi:10.15252/emmm.202114837)
Supplement: Supplementary file 14 [file EMMM-15-e14837-s014.pdf]

EMBO Molecular Medicine

Drug screening identifies tazarotene and bexarotene as therapeutic agents in Multiple Sulfatase Deficiency

Lars Schlotawa, Karolina Tyka, Matthias Kettwig, Rebecca Ahrens-Nicklas, Matthias Baud, Tea Berulava, Nicola Brunetti-Pierri, Alyssa Gagne, Zackary Herbst, Jean Maguire, Jlenia Monfregola, Tonatiuh Pena Centeno, Karthikeyan Radhakrishnan, Sophie Schröder, Elisa Waxman, Andrea Ballabio, Thomas Dierks, André Fischer, Deborah French, Michael Gelb, and Jutta Gärtner
DOI: 10.15252/emmm.202114837

Corresponding author: Lars Schlotawa (lars.schlotawa@med.uni-goettingen.de)

Review Timeline:

Submission Date:	12th Jul 21
Editorial Decision:	4th Aug 21
Revision Received:	7th Jun 22
Editorial Decision:	6th Jul 22
Revision Received:	8th Sep 22
Editorial Decision:	5th Oct 22
Revision Received:	9th Dec 22
Accepted:	9th Jan 23

Editor: Lise Roth

Transaction Report:

(Note: With the exception of the correction of typographical or spelling errors that could be a source of ambiguity, letters and reports are not edited. Depending on transfer agreements, referee reports obtained elsewhere may or may not be included in this compilation. Referee reports are anonymous unless the Referee chooses to sign their reports.)

4th Aug 2021

Dear Dr. Schlotawa,

Thank you for the submission of your manuscript to EMBO Molecular Medicine. We have now heard back from the three referees who agreed to evaluate your manuscript. As you will see below, the reviewers raise substantial concerns on your work, which preclude its publication in EMBO Molecular Medicine in its current form.

In particular, the referees mention the lack of mechanistic understanding downstream of retinoic acid receptors. Upon cross-commenting, referee #3 suggested:
"An RNAseq experiment comparing changes caused by a drug that ameliorates the cellular phenotype (e.g. tazarotene) with one that does not (e.g. etritinate or acitretin) would potentially address this and importantly add insight into the molecular mechanism and potentially identify downstream sites for more precise therapeutic intervention."

If you feel you can satisfactorily address this point and those listed by the referees, you may wish to submit a revised version of your manuscript. Please attach a covering letter giving details of the way in which you have handled each of the points raised by the referees. A revised manuscript will once again be subject to review and we cannot guarantee at this stage that the eventual outcome will be favorable.

EMBO Molecular Medicine encourages a single round of revision only and therefore, acceptance or rejection of the manuscript will depend on the completeness of your responses included in the next, final version of the manuscript. Should you find that the requested revisions are not feasible within the constraints outlined here and prefer, therefore, to submit your paper elsewhere, we would welcome a message to this effect.

When submitting your revised manuscript, please carefully review the instructions that follow below. We perform an initial quality control of all revised manuscripts before re-review; failure to include requested items will delay the evaluation of your revision.

We require:

1) A .docx formatted version of the manuscript text (including legends for main figures, EV figures and tables). Please make sure that the changes are highlighted to be clearly visible.

2) Individual production quality figure files as .eps, .tif, .jpg (one file per figure). For guidance, download the 'Figure Guide PDF' (https://www.embopress.org/page/journal/17574684/authorguide#figureformat).

3) A .docx formatted letter INCLUDING the reviewers' reports and your detailed point-by-point responses to their comments. As part of the EMBO Press transparent editorial process, the point-by-point response is part of the Review Process File (RPF), which will be published alongside your paper.

4) A complete author checklist, which you can download from our author guidelines (https://www.embopress.org/page/journal/17574684/authorguide#submissionofrevisions). Please insert information in the checklist that is also reflected in the manuscript. The completed author checklist will also be part of the RPF.

5) It is mandatory to include a 'Data Availability' section after the Materials and Methods. Before submitting your revision, primary datasets produced in this study need to be deposited in an appropriate public database, and the accession numbers and database listed under 'Data Availability'. Please remember to provide a reviewer password if the datasets are not yet public (see https://www.embopress.org/page/journal/17574684/authorguide#dataavailability).

In case you have no data that requires deposition in a public database, please state so in this section. Note that the Data Availability Section is restricted to new primary data that are part of this study.

6) For data quantification: please specify the name of the statistical test used to generate error bars and P values, the number (n) of independent experiments (specify technical or biological replicates) underlying each data point and the test used to calculate p-values in each figure legend. The figure legends should contain a basic description of n, P and the test applied. Graphs must include a description of the bars and the error bars (s.d., s.e.m.).

7) We would also encourage you to include the source data for figure panels that show essential data. Numerical data should be provided as individual .xls or .csv files (including a tab describing the data). For blots or microscopy, uncropped images should be submitted (using a zip archive if multiple images need to be supplied for one panel). Additional information on source data and instruction on how to label the files are available at
.

8) Our journal encourages inclusion of *data citations in the reference list* to directly cite datasets that were re-used and obtained from public databases. Data citations in the article text are distinct from normal bibliographical citations and should directly link to the database records from which the data can be accessed. In the main text, data citations are formatted as follows: "Data ref: Smith et al, 2001" or "Data ref: NCBI Sequence Read Archive PRJNA342805, 2017". In the Reference list, data citations must be labeled with "[DATASET]". A data reference must provide the database name, accession number/identifiers and a resolvable link to the landing page from which the data can be accessed at the end of the reference. Further instructions are available at .

9) We replaced Supplementary Information with Expanded View (EV) Figures and Tables that are collapsible/expandable online. A maximum of 5 EV Figures can be typeset. EV Figures should be cited as 'Figure EV1, Figure EV2" etc... in the text and their respective legends should be included in the main text after the legends of regular figures.

- For the figures that you do NOT wish to display as Expanded View figures, they should be bundled together with their legends in a single PDF file called *Appendix*, which should start with a short Table of Content. Appendix figures should be referred to in the main text as: "Appendix Figure S1, Appendix Figure S2" etc.

- Additional Tables/Datasets should be labeled and referred to as Table EV1, Dataset EV1, etc. Legends have to be provided in a separate tab in case of .xls files. Alternatively, the legend can be supplied as a separate text file (README) and zipped together with the Table/Dataset file.

See detailed instructions here:
.

10) Every published paper now includes a 'Synopsis' to further enhance discoverability. Synopses are displayed on the journal webpage and are freely accessible to all readers. They include a short stand first (maximum of 300 characters, including space) as well as 2-5 one-sentences bullet points that summarizes the paper. Please write the bullet points to summarize the key NEW findings. They should be designed to be complementary to the abstract - i.e. not repeat the same text. We encourage inclusion of key acronyms and quantitative information (maximum of 30 words / bullet point). Please use the passive voice. Please attach these in a separate file or send them by email, we will incorporate them accordingly.

Please also suggest a striking image or visual abstract to illustrate your article as a PNG file 550 px wide x 300-600 px high.

11) As part of the EMBO Publications transparent editorial process initiative (see our Editorial at http://embomolmed.embopress.org/content/2/9/329), EMBO Molecular Medicine will publish online a Review Process File (RPF) to accompany accepted manuscripts.
In the event of acceptance, this file will be published in conjunction with your paper and will include the anonymous referee reports, your point-by-point response and all pertinent correspondence relating to the manuscript. Let us know whether you agree with the publication of the RPF and as here, if you want to remove or not any figures from it prior to publication.
Please note that the Authors checklist will be published at the end of the RPF.

EMBO Molecular Medicine has a "scooping protection" policy, whereby similar findings that are published by others during review or revision are not a criterion for rejection. Should you decide to submit a revised version, I do ask that you get in touch after three months if you have not completed it, to update us on the status.

I look forward to receiving your revised manuscript.

Yours sincerely,

Lise Roth

Lise Roth, PhD
Editor
EMBO Molecular Medicine

***** Reviewer's comments *****

Referee #1 (Comments on Novelty/Model System for Author):

This manuscript has used a drug screen in fibroblasts, and extend the results for selected targets in hiPSC-derived neuronal progenitor cells (NPCs). This is a good start, but progenitor cells are not the target cells for this disease. No attempt has been made to test differentiated derivatives of NPCs.

Referee #1 (Remarks for Author):

The manuscript by Schlotawa et al describe a screen with FDA approved drugs in fibroblasts from an MSD patient, and identify synthetic retinoid-derivatives as potential compounds that could increase sulfatase activity.

I have a major problem with the approach and the conclusions of this work. First, it is widely known that active retinoid derivatives have a wide range of biological activities; it is probably harder to find that retinoids do not affect a certain process than the opposite. The author elaborate on this in a short paragraph in the discussion, but they don't take this into account in their work. There are so many possible explanations for the findings in this paper that have not been addressed at all that it is not possible to make a proper interpretation. One of the most obvious explanations that I can come up with (which is certainly not the only one) is that the retinoid derivatives affect growth, proliferation, and differentiation of cells in culture. It is also widely known that the enzyme activities of lysosomal enzymes in vitro strongly depend on cell density, proliferation rates, and cell culture conditions. This is the reason that diagnostic centers have rigorously standardized protocols for cell cultures from which they measure enzyme activities. The same reasoning applies to accumulation of storage products in lysosomal disorders: these are most prominent in post-mitotic cells. The reason why lysosomal enzyme activities depend so strongly on culture conditions is to date still unclear, and may involve multiple intracellular pathways involved in transcription, protein processing. post-translational modification and lysosomal targeting.

At the same time, the authors do not provide a plausible explanation for the effects that they observe. Treatment with retinoid receptor agonists and antagonists of cells in culture does not address the issue raised above. Apart from that, I think that it is far too speculative to propose that these findings could provide a basis for the treatment of MSD, also given the risks associated with retinoid treatment.

Overall, my suggestion would be to tune down the tone of the manuscript with respect to its implications, to introduce and discuss the work in a more careful manner, and to submit this work to a more specialized journal.

Referee #2 (Comments on Novelty/Model System for Author):

The manuscript contains a significant amount of data which were all generated based on multiple independent experiments. There are no obvious flaws in statistical evaluation. This is the first description of a feasible therapeutic approach for this ultra rare disease. The results may very well lead to clinical translation in near future. Therefore medical novelty and impact of the data are high.

Referee #2 (Remarks for Author):

This paper is a significant contribution to therapy of multiple sulfatase deficiency, an ultra-rare disease. More generally the work may well serve as a template for research on other diseases of comparable low frequency. The manuscript presents many sound data with appropiate statistical evaluation. The data allow the conclusion that certain retinoids which are already clinically approved may be valuable drugs to treat this devastating disease. The data provide a sound basis for clinical trials in near future. I recommend the acceptance and publication.

However, a few minor issues need to be addressed. In particular, occasionally the conclusions in the results section do not really comply exactly with the data shown in the respective figures. At first glance this criticism may appear harsh. However, it only takes a precise adjustment of the wording in the text to reflect what is actually supported by the figures. There is no doubt about the quality of the data presented in the figures only their description is not entirely correct.

In figure 2D (just as one example) the authors conclude that maximum activity of ARSA was reached after 14 days. This conclusion is not really supported by the data. A maximum at 14 days would require that values for 14 days are significantly different from the enzyme activity at 8 days and 21 days, respectively. That, however, is not the case or the respective significance levels are not shown. The data as shown allow the conclusion that the maximum effect is reached at 8 days and remains constant thereafter. If the maximum is indeed reached at 14 days the respective significance values must be included. Similar criticism also applies to other figures and the respective conclusions.
Figure 1 C : Maximum effect of tazarotene is not reached at 25 µM (perhaps already at 10 µM)
Similarly Figure 1D, 1F, 1G, 1 H, 2A, 2B, 2C,

Figure EV2G and H. The figure does not show a statistically significant effect on the proliferation of MSD fibroblasts. Thus, it cannot be concluded that the effect is more obvious in control fibroblasts (page 9 bottom). It is only seen in controls and not in MSD.

In summary, the conclusions in the text should precisely comply with the data shown in the figures.

In figure 2 J there is no increased LAMPI staining in vehicle treated MSD fibroblast as compared to control fibroblasts. The pictures shown also suggest that there are smaller lysosomes in MSD fibroblasts as compared to controls. Figures also suggest that in the lower panel of figure 2J there is more LAMPI staining in the Taz/Bex treated MSD cells and that the lysosomes are much larger in the treated MSD cells. For the reader there is some contradiction between the immunofluorescence pictures and figures 2K and L.

Significance levels are missing in figure 2N.

If HX531 increases ARSA activity only at 10µM and 20µM the respective significance levels must be displayed in figure 3B.

Labelling of the y-axis in figure 3G suggests that the ARSA specific activity was determined as per mg ARSA protein. However, the data (and the methods section) suggest that ARSA protein amount was only determined by Western Blot and not by absolute quantification of ARSA as e.g. µg. Also the molecular mass of ARSA of 54 kDa appears rather small and would correspond to the deglycosylated protein. Given the three N-glycosylation sites the mass should be at least 60kDa than 54 kDa.

EV Figure 1 A: Why is ARSA activity shown as OD515 rather than in nmol/h x mg as in other figures?

Figure 1 G typo in panel G bexarotene

Page 38 line 3 delete full stop after in.

Referee #3 (Comments on Novelty/Model System for Author):

This is an interesting study directed at identifying novel treatments for a rare disorder, multiple sulfatase deficiencies (MSD), caused by variants in the SUMF1 gene encoding the formylglycine generating enzyme through a screen of clinically available drugs on an MSD patient derived cell line. The authors provide convincing evidence for beneficial effect of a subset of retinoids and focus tazarotene (identified in initial screen) and bexarotene (identified in retinoid specific screen). Unfortunately, they do not provide convincing details of more specific molecular mechanisms downstream of the retinoic acid receptors. This is particular importance as while tazarotene and bexarotene are used clinically, at least bexarotene is unlikely to reach necessary levels in the central nervous system for the effect on MSD patholphysiology when used at the doses currently used for cutaneous T cell lymphoma.

Referee #3 (Remarks for Author):

The authors provide data demonstrating the successful identification of tazarotene in a screen of MSD patient derived immortalized cell line with clinically available drugs for compounds that lead to increase in ARSA activity. In a screen directed at other retinouids, they identify bexarotene and identify an optimal combination ratio of tazarotene and bexarotene. They incorporate appropriate confirmatory assays (N-sulfoglucosamine sulfohydrolase activity and reduction in LAMP1 expression). In terms of mechanism, the authors also convincingly demonstrate the need for retinoic acid receptors in the effect of Tazarotene/bexarotene and demonstrate that the effect is mediated by increasing the stability of disease associated FGE isoforms. They state "both drugs would be able to target the organ system predominantly contributing to the clinical presentation of MSD patients". However, Ghosal et al, Alzheimer's & Dementia: Translational Research & Clinical Interventions Volume 2, Issue 2, June 2016, Pages 110-120 found peak CSF levels of bexaraotene in the 10-30 nanomolar range in humans (Ghosal et al, Alzheimer's & Dementia: Translational Research & Clinical Interventions Volume 2, Issue 2, June 2016, Pages 110-120) in patients treated with standard dose used for treatment of cutaneous T-cell lymphoma. Because of this, it is concerning that they are not able to identify more specific molecular mechanisms downstream of retinoic acid receptors. Given their previous work on the role of PDI in stability of FGE variants, it is surprising that they did not determine whether the effect tazarotene and bexarotene requires PDI expression. More insight might also be gained from comparing gene expression changes associated with retinoids that did not have an effect on ARSA activity with the combination of tazarotene and bexarotene.

Detailed point by point responses to reviewers comments

Referee #1 (Comments on Novelty/Model System for Author):

This manuscript has used a drug screen in fibroblasts, and extend the results for selected targets in hiPSC-derived neuronal progenitor cells (NPCs). This is a good start, but progenitor cells are not the target cells for this disease. No attempt has been made to test differentiated derivatives of NPCs.

Referee #1 (Remarks for Author):

The manuscript by Schlotawa et al describe a screen with FDA approved drugs in fibroblasts from an MSD patient, and identify synthetic retinoid-derivatives as potential compounds that could increase sulfatase activity.

I have a major problem with the approach and the conclusions of this work. First, it is widely known that active retinoid derivatives have a wide range of biological activities; it is probably harder to find that retinoids do not affect a certain process than the opposite. The author elaborate on this in a short paragraph in the discussion, but they don't take this into account in their work. There are so many possible explanations for the findings in this paper that have not been addressed at all that it is not possible to make a proper interpretation. One of the most obvious explanations that I can come up with (which is certainly not the only one) is that the retinoid derivatives affect growth, proliferation, and differentiation of cells in culture. It is also widely known that the enzyme activities of lysosomal enzymes in vitro strongly depend on cell density, proliferation rates, and cell culture conditions. This is the reason that diagnostic centers have rigorously standardized protocols for cell cultures from which they measure enzyme activities. The same reasoning applies to accumulation of storage products in lysosomal disorders: these are most prominent in post-mitotic cells. The reason why lysosomal enzyme activities depend so strongly on culture conditions is to date still unclear, and may involve multiple intracellular pathways involved in transcription, protein processing. post-translational modification and lysosomal targeting.

At the same time, the authors do not provide a plausible explanation for the effects that they observe. Treatment with retinoid receptor agonists and antagonists of cells in culture does not address the issue raised above. Apart from that, I think that it is far too speculative to propose that these findings could provide a basis for the treatment of MSD, also given the risks associated with retinoid treatment.

Overall, my suggestion would be to tune down the tone of the manuscript with respect to its implications, to introduce and discuss the work in a more careful manner, and to submit this work to a more specialized journal.

We appreciate the honest criticism of referee #1. We fully agree that retinoids have a wide range of different biological activities and agree that multiple cellular mechanisms could be responsible for the effects described in our work. The other two referees have also mentioned this. To reveal more details on the mode of action we added additional experiments and results in the revised version of the manuscript providing more insight into the cellular mechanism of retinoid treatment in MSD cells. For example, we have added data on a new transcriptome analysis that reveals a remarkably strong upregulation of sterol and cholesterol synthesis pathways involving the ER resident SREBP-SCAP-INSIG- complex. This complex might provide a functional link between retinoid treatment, cholesterol homeostasis and ER stress affecting protein folding in MSD cells (please see details further below). Still this discovery is not a fully comprehensive explanation. More additional work will be necessary to elucidate the mechanisms of action and, importantly, ideally identify alternative targets that could be suitable for drug treatment to avoid unwanted effects of retinoid treatment.
We also fully agree with the referee that cell culture conditions contribute to cell growth and division, lysosomal enzyme activities, lysosomal positioning, and storage materials in cultivated cells. We addressed this issue by carefully introducing control conditions in our experiments and the use of multiple different MSD cell lines of different origin (fibroblasts and iPSC derived NPCs). We cannot entirely exclude any effect of influencing factors on the results. However, the corrective effect of the drug treatment is robustly reproducible and responsive to different culture conditions encompassing various cell lines, treatment protocols, different retinoids, as well as agonists, antagonists and inhibitors. Effects were even reproducible in different laboratories. Respecting the remarks, we are still convinced that our data reflect a biological response and rather not a false-positive outcome of culture conditions. Nevertheless, we fully agree that our in vitro findings need to be confirmed by in vivo experiments.

To consider the referee's criticisms we rewrote parts of the discussion section (see changes in red colour in the revised manuscript):

We changed the sentence on page 17 and 18 in the discussion part:
"*Taken together we provide in vitro evidence for the first potential small molecule therapy that could translate into a promising strategy for MSD patients in vivo given sustained supportive data in subsequent analysis. Nevertheless, identification of targets, mediating the tazarotene and bexarotene response in MSD cells that are amenable to alternative small molecule treatment with more favourable unwanted effects, is desirable.*"

In addition, we added a small paragraph on page 22 to reflect the referee's criticism on in vitro retinoid treatment:
"*Because retinoids mediate a plethora of intracellular actions via a multitude of intracellular pathways, our discoveries, yet to be described mechanistically, are not comprehensive. (di Masi et al., 2015). Direct effects on FGE as well as additional indirect retinoid mechanisms would have likely contributed to the restoration of sulfatase activities in combination with amelioration of cellular pathophysiology in MSD cells. Although we have shown a reproducible effect across various model cell lines and different experimental conditions, the influence of certain conditions (for example in vitro cell culture) cannot be ruled out. Future studies, that aim to delineate the molecular mechanism(s) of retinoid treatment in MSD, will be necessary to identify direct targets for alternative therapeutic intervention.*"

We rewrote part of the last paragraph of the discussion (conclusion and future perspectives) as follows (page 24/25):
"*In conclusion, the data presented here could have the potential for a new repurposed-drug based therapy for MSD, wherein tazarotene/bexarotene, if successful in subsequent analysis in vivo, could become a treatment option*."

Referee #2 (Comments on Novelty/Model System for Author):

The manuscript contains a significant amount of data, which were all generated based on multiple independent experiments. There are no obvious flaws in statistical evaluation. This is the first description of a feasible therapeutic approach for this ultra-rare disease. The results may very well lead to clinical translation in near future. Therefore, medical novelty and impact of the data are high.

We thank referee #2 for the positive evaluation of our manuscript.

Referee #2 (Remarks for Author):

This paper is a significant contribution to therapy of multiple sulfatase deficiency, an ultra-rare disease. More generally, the work may well serve as a template for research on other diseases of comparable low frequency. The manuscript presents many sound data with appropriate statistical evaluation. The data allow the conclusion that certain retinoids, which are already clinically approved, may be valuable drugs to treat this devastating disease. The data provide a sound basis for clinical trials in near future. I recommend the acceptance and publication.

Again, we are grateful for the overall positive evaluation of our manuscript.

However, a few minor issues need to be addressed. In particular, occasionally the conclusions in the results section do not really comply exactly with the data shown in the respective figures. At first glance, this criticism may appear harsh. However, it only takes a precise adjustment of the wording in the text to reflect what is actually supported by the figures. There is no doubt about the quality of the data presented in the figures only their description is not entirely correct.

We appreciate the referee's comment on the precision of the wording in the manuscript in relation to the figures and data. We carefully revised all figures, data and significance levels and made changes to Fig. 1-3 and EV1, 2, and 4. Accordingly we changed the text in the results part of the manuscript, the discussion, and the figure legends where appropriate, aiming for the highest precision and compliance of data and description. Please see details of the changes highlighted in red in the revised version of the manuscript.

In figure 2D (just as one example) the authors conclude that maximum activity of ARSA was reached after 14 days. This conclusion is not really supported by the data. A maximum at 14 days would require that values for 14 days are significantly different from the enzyme activity at 8 days and 21 days, respectively. That, however, is not the case or the respective significance levels are not shown. The data as shown allow the conclusion that the maximum effect is reached at 8 days and remains constant thereafter. If the maximum is indeed reached at 14 days the respective significance values must be included.
Similar criticism also applies to other figures and the respective conclusions.
Figure 1 C : Maximum effect of tazarotene is not reached at 25 µM (perhaps already at 10 µM)
Similarly Figure 1D, 1F, 1G, 1 H, 2A, 2B, 2C,

We appreciate the comment on the wording of data interpretation and fully agree with the referee's remark. In order to improve the results and their description we added additional labels for significance levels when activities were significantly different to adjacent concentrations or treatment times, respectively. The annotation of significance levels of tazarotene and bexarotene treated cells versus controls (DMSO) was left unchanged. Please see the revised Fig. 1 C, F, G, H, Fig. 2 A, B, C, D, and Fig EV2E. We adjusted the wording in the text, e.g. we deleted the indication of a dose with maximum activity increase when this was not supported by the data and any fold increase of sulfatase activities at a maximum drug dose because this was not explicitly determined by statistical means. Accordingly, we changed the description of conclusions in the results part and figure legends for more precision. Please see the edited wording highlighted on pages 5-8 for details.

Figure EV2G and H. The figure does not show a statistically significant effect on the proliferation of MSD fibroblasts. Thus, it cannot be concluded that the effect is more obvious in control fibroblasts (page 9 bottom). It is only seen in controls and not in MSD.

We appreciate this comment. We changed the description of the results part with details on the proliferation of MSD fibroblasts. Please see page 8 for changes.

In summary, the conclusions in the text should precisely comply with the data shown in the figures.

Please see our comment above and the corrections and edits in either text or figure.

In figure 2J there is no increased LAMPI staining in vehicle treated MSD fibroblast as compared to control fibroblasts. The pictures shown also suggest that there are smaller lysosomes in MSD fibroblasts as compared to controls. Figures also suggest that in the lower panel of figure 2J there is

more LAMPI staining in the Taz/Bex treated MSD cells and that the lysosomes are much larger in the treated MSD cells. For the reader there is some contradiction between the immunofluorescence pictures and figures 2K and L.

We tried to clarify the raised issue by improving the quality of the selected, representative pictures in figures 2 I and J. We hope that the pictures in Fig 2 now represent better the quantification of LAMP1 integrated fluorescence density (Fig 2K) and LAMP1-positive vesicle size (Fig. 2J) that is based on the quantification of all pictures from the experiments as described in the materials and methods section. We also took care of adjusting the results part where we exchanged the term “staining” against “LAMP1 integrated fluorescence density”. Please see the highlighted text on page 9.

Significance levels are missing in figure 2N.

We added significance levels for figure 2N. Please see the revised figure 2 with the changes requested. We also revised table EV1 and added significance levels for the comparison of GAG subspecies under DMSO conditions. In addition, we rewrote the corresponding paragraph in the results section and made adjustments in the figure legend. Please see page 10 of the revised manuscript (highlighted).

If HX531 increases ARSA activity only at 10 µM and 20 µM the respective significance levels must be displayed in figure 3B.

We thank the reviewer for this comment and made general edits to Fig. 3A and B. We added more significance levels in both figures following the reviewer’s first introductive suggestion on the precision of the data. We adjusted the respective description in the results part and the figure legends where appropriate. Please see page 11 of the revised manuscript (highlighted).

Labelling of the y-axis in figure 3G suggests that the ARSA specific activity was determined as per mg ARSA protein. However, the data (and the methods section) suggest that ARSA protein amount was only determined by Western Blot and not by absolute quantification of ARSA as e.g. µg. Also the molecular mass of ARSA of 54 kDa appears rather small and would correspond to the deglycosylated protein. Given the three N-glycosylation sites, the mass should be at least 60kDa than 54 kDa.

Again, we appreciate this comment. Indeed, the ARSA activity was calculated based on the signal intensity of bands visualized in an ARSA Western Blot. This blot did not use defined amounts of ARSA as reference that would allow a proper quantification of signals in µg. It rather determines the signal intensity of ARSA protein in every treatment condition in reference to the DMSO treatment condition. We changed the labelling of the y-axis in Fig 3G and after re-arrangement of figures in the new Fig EV5A (GALNS activity based on GALNS protein amount), added additional significance levels, and adjusted the explanation in the figure legend.
In addition, we thank the referee for the comment on the molecular mass of ARSA that was indeed 60 kDa rather than 54 kDa. We changed the labelling in the revised figure 3 panel G.

EV Figure 1 A: Why is ARSA activity shown as OD515 rather than in nmol/h x mg as in other figures?

We apologise for the wrong panel A in Fig. EV1 that resulted from a copy and paste error from a previous manuscript version. Please see the revised Fig. EV1 as well as the corrections in the figure legend and the results part. The same error appeared in Fig 1B and has been corrected accordingly together with corrections in the figure legend and the text (page 5, highlighted)

Figure 1 G typo in panel G bexarotene

We corrected the typo in Figure 1G

Page 38 line 3 delete full stop after in.

We deleted the full stop.

Referee #3 (Comments on Novelty/Model System for Author):

This is an interesting study directed at identifying novel treatments for a rare disorder, multiple sulfatase deficiencies (MSD), caused by variants in the SUMF1 gene encoding the formylglycine generating enzyme through a screen of clinically available drugs on an MSD patient derived cell line. The authors provide convincing evidence for beneficial effect of a subset of retinoids and focus tazarotene (identified in initial screen) and bexarotene (identified in retinoid specific screen). Unfortunately, they do not provide convincing details of more specific molecular mechanisms downstream of the retinoic acid receptors. This is particular importance as while tazarotene and bexarotene are used clinically, at least bexarotene is unlikely to reach necessary levels in the central nervous system for the effect on MSD pathophysiology when used at the doses currently used for cutaneous T cell lymphoma.

Referee #3 (Remarks for Author):

The authors provide data demonstrating the successful identification of tazarotene in a screen of MSD patient derived immortalized cell line with clinically available drugs for compounds that lead to increase in ARSA activity. In a screen directed at other retinoids, they identify bexarotene and identify an optimal combination ratio of tazarotene and bexarotene. They incorporate appropriate confirmatory assays (N-sulfoglucosamine sulfohydrolase activity and reduction in LAMP1 expression). In terms of mechanism, the authors also convincingly demonstrate the need for retinoic acid receptors in the effect of Tazarotene/bexarotene and demonstrate that the effect is mediated by increasing the stability of disease associated FGE isoforms.

They state "both drugs would be able to target the organ system predominantly contributing to the clinical presentation of MSD patients". However, Ghosal et al, Alzheimer's & Dementia: Translational Research & Clinical Interventions Volume 2, Issue 2, June 2016, Pages 110-120 found peak CSF levels of bexaraotene in the 10-30 nanomolar range in humans (Ghosal et al, Alzheimer's & Dementia: Translational Research & Clinical Interventions Volume 2, Issue 2, June 2016, Pages 110-120) in patients treated with standard dose used for treatment of cutaneous T-cell lymphoma. More insight might also be gained from comparing gene expression changes associated with retinoids that did not have an effect on ARSA activity with the combination of tazarotene and bexarotene.

We thank the referee for his/her valuable comments on the paper and we appreciate the questions, remarks and concerns. We admit that bexarotene concentrations as low as 20-30 nmol of bexarotene have not been used in our in-vitro experiments. Anyway, we could detect a response to tazarotene/bexarotene treatment at lower concentrations if we increased the treatment time, indicating that at least the in-vitro treatment of MSD fibroblasts was time- and dose dependent.

Taking into account that the in-vivo concentrations of tazarotene in CSF are not known, active concentrations might be variable in different tissues and body fluids and even low drug concentrations might be beneficial in a long-term treatment. Evidence for a benefit of the described treatment would need to be generated in suitable in-vivo treatment models and trials.
The statement that "both drugs would be able to target the organ system predominantly contributing to the clinical presentation of MSD patients" refers to the observation that we were able to re-establish sulfatase activities in neuronal progenitor cells from MSD patient derived iPSC rather than achievable CSF concentrations of either drug. However, we agree that our conclusion is optimistic in knowledge of the data on the detectable amounts of bexarotene in CSF.

To avoid overarching statements and provide clarity we rewrote this part as follows (see highlighted text on page 24):
"However, although both drugs would be able to reach the central nervous system, the organ system predominantly contributing to the clinical presentation of MSD patients (Ahrens-Nicklas et al., 2018), and were effective over time at lower concentrations, evidence needs to be generated that drug concentrations in the CNS will be high enough to result in effective sulfatase activation. Data from healthy subjects treated with standard concentrations of bexarotene revealed a low penetrance of the drug into the CNS and concentrations of only approximately 20 nM (Ghosal et al, 2016)."

Because of this, it is concerning that they are not able to identify more specific molecular mechanisms downstream of retinoic acid receptors.

We thank the referee for bringing up this important point and fully agree that the identification of suitable targets downstream of retinoic acid receptors and use of alternative compounds for improved treatment efficacy should be the final aim also circumventing unwanted effects of retinoid acid derivatives. We considered the referee's concern and followed his/her suggestion on new experiments thereby providing additional data that we added to the revised version of the manuscript (please see details below). Furthermore, we added a statement to the discussion part on page 25 as last sentence:
"*Identification of more mechanistic details unravelling alternative treatment targets should be the subject of further research.*"

Given their previous work on the role of PDI in stability of FGE variants, it is surprising that they did not determine whether the effect tazarotene and bexarotene requires PDI expression.

We understand that the referee is referring to our previous work (Schlotawa et al., Cell Reports, 2018) where we have shown PDI's involvement as a disease modifier in MSD that specifically recognizes misfolded FGE variants. PDI facilitates variant degradation thereby severely reducing their intracellular stability and thus their residual activity.
Data in our present manuscript show FGE variant stabilization or decreased degradation upon tazarotene/bexarotene treatment (Fig. 4). The same observation was true after PDI knockdown and reduced PDI expression in our previous work (Schlotawa et al., Cell Reports, 2018; Fig. 2F) and we wondered if tazarotene/bexarotene treatment in the present study affected PDI expression. However, in the present study, we detected neither transcriptional regulation nor any change in PDI protein expression upon tazarotene/bexarotene treatment of MSD fibroblasts. In the submitted manuscript, data on PDI transcription were included into Fig. EV3B (*PH4B*) and the results part on page 13. We did not include western blot data because there were no obvious changes in PDI protein expression between treated and untreated MSD cells. Following the referee's comment, we now

added a western blot analysis as appendix Fig. S11 and described PDI protein expression in the results part on page 16 (see below).

Nevertheless, addressing the referee's remark, we investigated PDIs role in MSD after tazarotene/bexarotene treatment in more detail. We repeated an experiment from our previous abovementioned work on the activation of steroid sulfatase (STS) by FGE-S155P variant with and without PDI co-expression. We used the same experimental conditions but now upon treatment of cells with either DMSO (vehicle control) or tazarotene/bexarotene. As compared to DMSO treated cells, tazarotene/bexarotene consistently led to an increase in STS activity with and without FGE co-expression. When compared to respective DMSO controls, in FGE-S155P alone expressing cells, tazarotene/bexarotene treatment led to an approx. 4.5-fold increase in activity while co-expression of PDI in drug treated cells led to a 7.5-fold increase. However, note that, this apparent increase in activity upon PDI co-expression in drug treated cells is due to the decrease in activity of FGE-S155P when PDI is co-expressed in DMSO treated cells. As shown in appendix Fig. S8D, in control cells, co-expression of PDI results in a 3-fold loss of S155P variants activity, in agreement to our previous work. Such a loss in activity is also observed in drug treated cells, however, only to 2-fold. This indicates that co-expression of PDI has a minor effect on prohibiting FGE-S155P induced STS activation when cell are treated with tazarotene/bexarotene compared to DMSO treated cells. Also supported by our data that tazarotene/bexarotene treatment improves the intracellular stability of FGE variants (Fig. 4) we conclude that it is plausible that tazarotene/bexarotene treatment reduces the proportion of misfolded FGE variants able to escape PDI recognition by improving their folding. Consequently, PDI-escaping FGE variants are available to activate sulfatases.

We added the new results on PDI in a short paragraph on page 16 of the revised manuscript and connected the results with the observations on FGE stability (Fig. 4):
"PDI is a pivotal interacting partner of FGE that preferentially binds misfolded FGE proteins, impairs their residual enzyme activity and determines their fate by early degradation (Schlotawa et al., 2018). We analysed the role of PDI in treated MSD cells. In addition to unchanged PDI transcription (PH4B, see above), we could not detect any differences in PDI protein expression upon tazarotene/bexarotene treatment (see appendix results for details, Appendix figure S11). (Schlotawa et al., 2018). However, tazarotene/bexarotene treatment decreased a PDI-mediated inhibition of FGE variants residual activity (appendix results, Appendix Fig. S12).
Such increase in FGE activity could be a result of less PDI interaction due to improved FGE variant protein stability. We finally assessed if the FGE half-life changed upon tazarotene and bexarotene treatment performing a previously described pulse-chase experiment with HT1080 cells stably expressing FGE variants as a cell model (Schlotawa et al., 2011; Schlotawa et al., 2018). Because wild type FGE and variant FGE, depending on the type of mutation, is also secreted upon overexpression we assessed levels of intracellular and secreted FGE protein. FGE half-life was determined after three days of treatment with tazarotene/bexarotene 10/20 µM and DMSO controls. All cell lines except HT1080-FGESer155Pro secreted a truncated form of FGE and the protein half-life, calculated from intracellular and secreted FGE protein amounts, significantly increased for FGESer155Pro (2-fold, Fig. 4A) and FGE Gly247Arg (1.5-fold, Fig 4B). The half-life of FGE Ala279Val showed a trend towards an increase (1.7-fold, Fig 4C). Half-life of FGE wild type was unchanged upon treatment (Fig EV5B)."

We added details on the PDI results as appendix results, appendix figures S11, S12, and additional materials and methods in the revised appendix to the manuscript.

We also rewrote a part of the discussion at page 21 and 22:

“Our observations strongly suggest that tazarotene/bexarotene act to increase the activity of FGE. FGE misfolding due to SUMF1 mutations affects its stability followed by accelerated degradation. Despite residual catalytic activity of FGE variants, accelerated degradation results in impaired sulfatase activities (Schlotawa et al, 2013). PDI has been identified to play a pivotal role in mediating FGEs intracellular degradation. Decelerated degradation and prolonged half-life of misfolded FGE results in higher sulfatase activities (Schlotawa et al., 2018). Here, treatment with tazarotene and bexarotene increased the half-life of misfolded FGE, likely enhancing residual FGE activity that partially restores sulfatase activities in MSD cells. This hypothesis is further supported by the observation that tazarotene/bexarotene failed to increase sulfatase activation in cells that lack FGE. Tazarotene/bexarotene- induced increased FGE variant half-life could result from several factors including impaired protein degradation, improved protein folding, or both. Interestingly, based on our results, PDI seems not be a direct target of tazarotene/bexarotene treatment. However, effects on ER quality control mechanisms like the unfolded protein response or beneficial induction of ER stress (Mollereau et al, 2014) could be possible explanations. If existing functional links between ER stress and regulation of lipid metabolism involving the SREBP-SCAP-INISG complex (Moncan et al, 2021) connect FGE stabilization and regulation of sterol and cholesterol pathway regulation, both induced by tazarotene treatment in MSD cells, need to be elucidated by future experiments.”

An RNAseq experiment comparing changes caused by a drug that ameliorates the cellular phenotype (e.g. tazarotene) with one that does not (e.g. etritinate or acitretin) would potentially address this and importantly add insight into the molecular mechanism and potentially identify downstream sites for more precise therapeutic intervention.

We thank the referee for his/her excellent suggestion of such intriguing experimental setup. We performed an additional RNAseq experiment to analyse differences on gene expression between MSD fibroblasts treated with tazarotene and a selected retinoid that failed to restore sulfatase activities (referred to as “negative retinoid”). In summary, we were able to identify tazarotene-specific enriched upregulation of genes belonging to sterol and cholesterol synthesis pathways, in particular the SREBP-SCAP-INSIG complex involved in sensing and regulation of the ER lipid content and discuss the implications of these findings in MSD context.

In order to identify a “negative control” retinoid we performed a set of preparatory experiments. First, we treated primary MSD fibroblasts (p.Gly247Arg) with 5 or 10 µM of selected retinoids from each generation of pharmacological development (1st generation: tretinoin, 2nd generation: acitretin, 3rd generation: adapalene) for six days and analysed three different sulfatase activities (ARSA, GALNS, STS). Parallel treatments with tazarotene (10 µM) and DMSO served as positive and negative controls, respectively. In contrast to tazarotene treatment, addition of either tretinoin, acitretin or adapalene did not increase activities of ARSA, GLANS, or STS.
Gene expression analysis by qPCR showed an increase in the expression of retinoid target genes *RARB, CYP26B1*, and *RARRES1-3* upon treatment of MSD fibroblasts with tretinoin, acitretin and adapalene compared to DMSO treatment. This confirmed that the drugs induced intended “retinoid” effects in MSD primary fibroblasts, however, without sulfatase activity restoration.
We chose adapalene for further experiments because it belongs to the same pharmacological group of retinoids like tazarotene and bexarotene (3rd generation retinoids). As a first step, we treated MSD fibroblasts with four different concentrations of adapalene (1, 5, 10, 20 µM) for six days. All tested concentrations resulted in no increase in activation of sulfatase activities but resulted in upregulation of the target genes confirming our initial observation. Of note, 20 µM was visibly toxic to the cells. Next, treatment of eight MSD fibroblast lines with different *SUMF1* mutations for six days with 5 µM adapalene showed no ARSA activity increase thereby ruling out mutation specific effects. Finally, we

incubated MSD fibroblasts with the pan-RAR antagonist AGN193109 followed by treatment with adapalane and tazarotene, respectively. Such treatment abrogated both adapalene and tazarotene induced retinoid target gene expression in MSD fibroblasts indicating that both retinoids work via RAR receptors in a comparable fashion. Based on these results we chose adapalene as a suitable negative control retinoid for total RNA sequencing in MSD cells.

Based on the results from the preparatory experiments we treated seven different MSD primary fibroblast cell lines for six days with adapalene, tazarotene and DMSO. After RNA extraction, all samples were referred to total RNA sequencing and bioinformatics analysis as described in the materials and methods section. To verify the treatment quality we analysed again a subset of retinoid target genes. *RARB, CYP26B1*, and *RARRES1* were significantly upregulated upon both tazarotene or adapalene treatment compared to DMSO, indicating a positive treatment response. In all cell lines, we first compared gene expression between tazarotene and adapalene treatment and identified 1042 differentially regulated genes. Pathway analysis of tazarotene regulated genes revealed enrichment of sterol and cholesterol biosynthesis pathways, whereas adapalene mostly regulated developmental pathways. When analysing differential gene expression of each treatment condition against DMSO we found 2268 regulated genes in all cell lines upon tazarotene treatment and 2004 upon adapalene treatment, respectively. 1620 genes were identically regulated by both tazarotene or adapalene treatment, whereas 684 genes were exclusively regulated upon tazarotene treatment and 384 genes exclusively upon adapalene treatment. We focused on genes exclusively regulated upon tazarotene treatment to identify pathways and processes that could mediate sulfatase activation. Of interest, the most enriched GO terms and pathways in the group of tazarotene-regulated genes were again steroid or cholesterol biosynthesis. Enriched upregulated genes in these pathways were *SREBPF1*, *SREBPF2* and *INSIG1* as members of the SREBP-SCAP-INSIG complex among others. The complex's intrinsic function is the transcriptional regulation of sterol and cholesterol synthesis and sensing of lipid contents in ER membranes (Brown et al., 2018). This finding is of particular interest as defective cholesterol re-distribution in cellular vesicles has been proposed as tertiary storage pathology for a number of lysosomal storage disorders (Platt et al., 2012). This finding might provide a functional link between cholesterol sensing and synthesis to sulfatase activity restoration in MSD cells given the complex (self-) regulation of SREBP-SCAP-INSIG function. In order to provide at least first preliminary evidence for a function of the SREBP-SCAP-INSIG complex in tazarotene mediated sulfatase activity restoration in MSD cells, we treated immortalized MSD fibroblasts (MSDi) with increasing concentrations of fatostatin, a SCAP inhibitor. Whereas fatostatin alone displayed no effects on ARSA activity in MSDi cells, combined treatment with tazarotene inhibited tazarotene induced ARSA restoration in a dose dependent way.

To incorporate our findings into the revised manuscript we added details on the additional experimental procedures under materials and methods on pages 31 and 32 as well as under appendix supplementary materials and methods. Results of the preparatory experiments were added as appendix supplementary results, illustrated by new appendix figures and appendix figure legends (appendix figures S6-S8).

We incorporated a new paragraph into the main results section on pages 13-15:
"In an attempt to further identify genes and pathways that are mediating sulfatase activity restoration upon treatment we compared transcriptional response of tazarotene ("positive" for sulfatase activity restoration) and the response of a retinoid that is incapable ("negative") of restoring sulfatase activities in MSD cells (Fig 1E). Through a set of preparatory experiments, we chose adapalene to serve as a control retinoid that provokes expression of retinoid targets genes in MSD cells without increasing sulfatase activities (please see appendix results for details, Appendix Fig.

S6-S8). We treated seven MSD fibroblast lines with either adapalene, tazarotene, or DMSO (control) in triplicates for six days and referred all samples to total RNA sequencing and differential gene expression analysis (Appendix Fig. S9A). We identified expression of 10992 genes. For quality control, we analysed a subset of retinoid target genes, which were significantly upregulated (RARB, CYP26B1, RARRES1) for both tazarotene and adapalene treatment compared to DMSO conditions, indicating a positive treatment response (Appendix Fig. S9B). Analysing all cell lines we found 1042 genes differentially regulated when we compared gene expression between tazarotene and adapalene treatment (Appendix Fig. S9C). GO biological process pathway analysis of the significantly differentially expressed genes by tazarotene (positive retinoid) revealed sterol synthesis and cholesterol synthesis pathways (Appendix Fig S9D) whereas pathways regulated by adapalene were mostly developmental pathways (Appendix Fig. S9D). When comparing tazarotene and adapalene induced gene expression against DMSO conditions (untreated), respectively, 2268 genes were regulated upon tazarotene treatment and 2004 upon adapalene treatment compared to DMSO conditions. 1620 genes were identically regulated by both tazarotene or adapalene treatment, whereas 684 genes were exclusively regulated upon tazarotene treatment and 384 genes exclusively upon adapalene treatment (Fig. EV4E, Appendix Fig. S9F,G). GO biological pathway analysis of genes exclusively regulated by adapalene treatment identified phosphatidylinositol mediated signalling pathways (Appendix Fig. S9G). We focused on genes exclusively regulated upon tazarotene treatment because of its positive action on sulfatase activity restoration and identified 313 upregulated and 335 downregulated genes. GO biological process pathway analysis in the group of genes exclusively regulated by tazarotene treatment identified again sterol and cholesterol biosynthesis (Fig. EV4E). Upregulated individual genes in these pathways, among others, were SREBPF1, SREBF2 and INSIG1 (Appendix Fig. S9H) coding for sterol regulatory element-binding proteins 1and 2 (SREBP1, SREBP2) and insulin-induced gene proteins proteins (INSIG). Together with the SREBP cleavage-activating protein (SCAP) these proteins are part of the SREBP-SCAP-INSIG complex in the ER sensing and controlling ER and cellular cholesterol content (Brown et al, 2018; Yang et al, 2002). In order to reveal any functional involvement of the complex in tazarotene mediated sulfatase activity restoration we treated immortalized MSD cells with tazarotene and fatostatin for three days. Fatostatin suppresses SCAP/SREBP translocation (Cheng et al, 2018). Whereas treatment with increasing concentrations of fatostatin did not increase ARSA activity, tazarotene did. Simultaneous treatment with tazarotene (10 µM) and increasing fatostatin concentrations caused a dose dependent ARSA activity decrease (Appendix Fig. S9I).”

We added the main findings of the transcriptome analysis as new panel E to Fig. EV4 and appendix figure S9, accompanied by new figure legends.

We also added a paragraph, discussing the new results at page 19 and 20:
“*The interesting observation that the restoration of sulfatase activities in MSD cells was not general but restricted to a few retinoids was used to analyse differences between the transcriptional responses of a “positive” and a “negative” retinoid. Although we cannot exclude an interference of the phosphoinositol kinase pathway, which was transcriptionally regulated exclusively by adapalene treatment, thereby silencing potential adapalene regulated cellular mechanisms capable of restoring sulfatase activities we focused our transcriptome analysis on tazarotene. Such approach identified genes encoding members of the SREBP-SCAP-INSIG complex to be upregulated exclusively by tazarotene. The complex’s intrinsic function is the transcriptional regulation of sterol and cholesterol synthesis and sensing of lipid contents in ER membranes (Brown et al., 2018). This function, controlled by feedback mechanisms and cellular transcription pathways could be functionally linked to cholesterol storage in MSD cells (Eid et al, 2017). Cholesterol storage resulting from impaired digestion and redistribution of membrane contents is a known downstream effect in various*

lysosomal storage disorders (Platt et al, 2012). Details for tazarotene influencing the SREBP-SCAP-INSIG complex in the MSD cellular context could be manifold given the orchestrated self-regulation of the complex (McPherson & Gauthier, 2004) and will be subject of future analysis. Treatment with fatostatin, an inhibitor of SREBP translocation, revealed blocking of the ARSA activity restoration by tazarotene (see appendix results) though details are yet to be elucidated in MSD cells."

All RNAseq data are accessible via the GEO database (https://www.ncbi.nlm.nih.gov/geo) using the accession GSE205555, access token ytepqamwzzahled (initial RNAseq: MSD fibroblasts vs. control fibroblasts, treatment: tazarotene/bexarotene vs. DMSO), and GSE205556, access token etmjwgymdxarfcn (second RNAseq: MSD fibroblasts, treatment: tazarotene vs. adapalene vs. DMSO).

Again, we thank the referee for suggesting this experiment that is likely to reveal first insights into a cellular mechanism how tazarotene/bexarotene mediate restoration of sulfatase activities in MSD. While we were able to partially reveal new mechanistic targets, we still fell short on providing easy to use drug alternatives to mimic tazarotene/bexarotene treatment. Still more details need to be elucidated in further research and reported in subsequent publications.

6th Jul 2022

Dear Dr. Schlotawa,

Thank you for submitting your revised manuscript to EMBO Molecular Medicine.
We have now received the reports from the three referees who re-reviewed your manuscript. As you will see, while referees #2 and #3 are overall satisfied with the revisions, referee #1 still raises major concerns on your work. As addressing all the remaining concerns would require considerable work and considering that at EMBO Press we usually allow a single round of major revisions, (but noting that two referees are now satisfied), we further discussed with the referees the revisions required to achieve publication level.
We would thus like to propose to revise the manuscript according to the following plan:
- provide additional information requested where needed (cell proliferation without normalization, total protein concentrations upon treatment, cell proliferation, density and protein content)
- discuss the differences in stability of different retinoids as a possible explanation for the differential effects observed in the manuscript discussion (vs. true differential physiological effects)
- describe in the discussion the necessary steps prior clinical application, given the toxicity of retinoids.

Please be aware that this will be the last chance for you to address these points.

Additionally, please also address the following editorial issues:
- Please address the queries from our data editors in the "Data edited ms file" in track changes mode and use this file for any further modification.
- Conflict of interest: We updated our journal's competing interests policy in January 2022 and request authors to consider both actual and perceived competing interests. Please review the policy https://www.embopress.org/competing-interests and update your competing interests if necessary.
- "References and Notes" should be corrected to "References".
- The heading "Figure Legends" is missing.
- Figures: please make sure that all figures and figure panels are referenced in the text (callouts for Fig. 3J and for several panels from the Appendix figures are missing).
- The Table EV1 legend should be removed from the main manuscript file and added to the table file in a separate tab.
- In the Appendix, the table of content should include the page numbers. Please remove the results and material and methods from the appendix and insert it in the main text.
- Thank you for providing Source Data. Please upload them to have one file per figure. Please also provide the source data (original blots) for figure EV2.
- As part of the EMBO Publications transparent editorial process initiative (see our Editorial at http://embomolmed.embopress.org/content/2/9/329), EMBO Molecular Medicine will publish online a Review Process File (RPF) to accompany accepted manuscripts. In the event of acceptance, this file will be published in conjunction with your paper and will include the anonymous referee reports, your point-by-point response and all pertinent correspondence relating to the manuscript. Let us know whether you agree with the publication of the RPF and as here, if you want to remove or not any figures from it prior to publication. Please note that the Authors checklist will be published at the end of the RPF.

I look forward to receiving your revised manuscript.

With kind regards,

Lise Roth

Lise Roth, PhD
Editor
EMBO Molecular Medicine

***** Reviewer's comments *****

Referee #1 (Comments on Novelty/Model System for Author):

no in vivo data, claims that these retinoids could be useful for patients are overstated

Referee #1 (Remarks for Author):

The authors have performed additional experiments showing that the effects of retinoids can not be explained by effects on cell proliferation. In this respect, it is unclear what the actual cell proliferation rates were in the cell lines investigated, as these are now plotted as % of DMSO. Please provide the actual cell proliferation data without normalisation as well.

The authors have not addressed my comment on using undifferentiated NPCs. The effects of retinoids are already much smaller in NPCs compared to fibroblasts; how would this be in neurons and astrocytes derived from NPCs, as these are the relevant cell types? Have the authors tried this, what was the outcome, and can they make at least a statement on this?

In general, enzyme activities are expressed per total protein. What happened to total protein concentrations upon treatment?

In Fig 2MN, how were the effects on proliferation, cell density in absolute terms during this period? How was protein content during this period or at the end?

In addition, they have performed an RNAseq experiment in which they compared an 'inactive' retinoid with an 'active' retinoid. There is a conceptual problem with the interpretation of this experiment in this manuscript. The mechanism by which retinoids act is via activation of nuclear receptors. All these retinoids have different stabilities in vitro, they are oxygen and light sensitive and degrade within hours. That is likely the reason why natural retinoids have lower activity in the assays in this manuscript while the synthetic ones have higher activity as these likely have higher stability. This means that the differences that are now picked up by a 'positive' retinoid versus a 'negative' retinoid likely reflect the different stabilities of these retinoids in culture and hence the kinetics of gene expression and mRNA stability. The real players involved may therefore remain hidden when comparing 'active' and 'inactive' retinoid treatment, and may be present somewhere in the bulk of retinoid responsive genes when comparing retinoid treatment with mock treatment. The experiment is therefore a deviation from the main point, which is: how do retinoids increase FGE activity?

The authors have added another experiment, in which they showed that retinoids increase the stability of FGE. This now provides a plausible explanation for the observed effects. It also makes sense that there is a requirement of residual FGE activity for the effects of retinoids. It hints to a rather simple mechanism in which players involved in turnover of less active/misfolded variants of FGE have a central role. This seems to be a key point in the possible mechanism and deserves follow up experiments to identify and characterise the retinoid-responsive players involved.

In the discussion, I still think the possibility to treat MSD with retinoids is overstated and premature given the lack of in vivo experiments and the toxicity of retinoids in general.

Referee #2 (Comments on Novelty/Model System for Author):

My criticism has been adequately addressed

Referee #3 (Comments on Novelty/Model System for Author):

This is a revised manuscript describing identification of possible treatments for a rare disorder, multiple sulfatase deficiencies (MSD), caused by variants in the SUMF1 gene encoding the formylglycine generating enzyme through a screen of clinically available drugs on an MSD patient derived cell line. The authors provide convincing evidence for beneficial effect of a subset of retinoids on MSD cell lines with hypomorphic SUMF1 mutations and focus tazarotene (identified in initial screen) and bexarotene (identified in retinoid specific screen). They additionally identify potential specific molecular mechanisms downstream of the retinoic acid receptors by comparing RNAseq profiles of MSD primary fibroblasts lines treated with adapalene (an ineffective 3rd generation retinoid) and tazarotene. Pathway analysis of genes exclusively regulated by tazarotene treatment identified sterol and cholesterol biosynthesis pathways. Since the authors demonstrated that RXR and RAR antagonists HX 531 and AGN 193109 block the effect of tazarotene on ARSA activity, it would be interesting to know whether these antagonists block the effect of tazarotene on upregulation of sterol and cholesterol biosynthesis pathways, but is arguably beyond the scope of this manuscript.

Referee #3 (Remarks for Author):

This is a very interesting study that lays the groundwork for further characterization of the mechanisms by which a subset of

retinoids attenuate the effects of hypomorphic variants in the SUMF1 gene associated with MSD.

Referee #1 (Comments on Novelty/Model System for Author):

no in vivo data, claims that these retinoids could be useful for patients are overstated

Thank you very much for this comment. We tried to adjust the respective part of the discussion again. Please see the changes that we made to the discussion part and our revised paragraph following the request of the editor further above.

Referee #1 (Remarks for Author):

The authors have performed additional experiments showing that the effects of retinoids cannot be explained by effects on cell proliferation. In this respect, it is unclear what the actual cell proliferation rates were in the cell lines investigated, as these are now plotted as % of DMSO. Please provide the actual cell proliferation data without normalisation as well.

We appreciate the referee's comment on agreeing that the effects of tazarotene and bexarotene cannot be explained by different cell proliferation rates. With this second revision, we provide non-normalised data on cell proliferation for the respective experiment. Raw data on proliferation between MSD primary fibroblasts and control fibroblasts show clear inert differences between the two cell lines used in the experiments even for control conditions. This is why we used normalization to DMSO conditions to make a comparison of the differences provoked by tazarotene and bexarotene treatment in either fibroblast line easier. To illustrate all results, we combined normalised and non-normalised data of the experiments in the new Appendix Figure S6 and removed the original normalized data from figure EV2.

In order to provide cell counts and total protein amounts upon treatment we designed and performed an additional experiment. We analysed cell quantity, total protein content, and ARSA activity in MSD and control fibroblasts under standard cell culture conditions and DMSO treatment over the course of nine days. In parallel the same cell lines were treated with tazarotene, bexarotene and tazarotene/bexarotene for analysis of the same parameters.
We would like to argue that tazarotene and bexarotene treatment reduced cell proliferation of MSD and control fibroblasts. In our experiments, cell quantities assessed by manual counting were significantly lower for both MSD- and control fibroblasts upon treatment whereas data generated by XTT assays only showed significant differences in cell proliferation upon treatment for control fibroblasts. Considering that XTT assays are metabolic assays influenced by cell count, cell size, and protein content, we assume that the different experimental approaches account for the different results. Total protein amounts correspond to reduced cell quantity. Interestingly, we could not detect significant differences in total protein amounts in MSD fibroblasts in either experimental condition. Anyway, endogenous ARSA activity in MSD fibroblasts under DMSO conditions was not influenced by treatment time and cell quantity and did not show significant differences. A significant increase occurred in MSD fibroblasts treated with tazarotene or tazarotene/bexarotene, respectively. Furthermore, we could prove that cell quantity and cultivation time had a positive influence on ARSA activity in control fibroblasts.

Please see the modified appendix, new Appendix Fig. S5, and new Appendix Tables S3-S8 for a detailed description of the experiment and the results. In addition, please see more details on changes in the results part and the discussion of the main manuscript above in the point-by-point response to the editor's comments.

The authors have not addressed my comment on using undifferentiated NPCs. The effects of retinoids
are already much smaller in NPCs compared to fibroblasts; how would this be in neurons and astrocytes derived from NPCs, as these are the relevant cell types? Have the authors tried this, what was the outcome, and can they make at least a statement on this?

Unfortunately, we have not further differentiated NPCs into neurons and astrocytes and treated them with tazarotene and bexarotene. We agree that both cell lines are most relevant cell types for MSD but at this stage, we can only speculate on the effects of such treatment on sulfatase activities in either cell type. The use of NPCs in our experiments served as proof of principle that tazarotene and bexarotene treatment work across cell types different to fibroblasts. Further experiments on iPSC derived neurons and astrocytes or even other cells from tissues with contribution to MSD pathology are highly desirable and subject to future experiments.

We edited a sentence to the discussion part at page 24 addressing the point raised:

Retinoic acid derivatives are able to cross the blood brain barrier (Dos Santos Guilherme et al, 2019) and tazarotene and bexarotene increased activities of sulfatases in undifferentiated NPCs in a proof of principle experiment as described above. However, if both drugs would work on neurons and astrocytes in vitro and in vivo and would be able to reach the central nervous system, the organ system predominantly contributing to the clinical presentation of MSD patients (Ahrens-Nicklas et al., 2018), evidence needed to be generated that drug concentrations in the CNS will be high enough to result in effective sulfatase activation.

In general, enzyme activities are expressed per total protein. What happened to total protein concentrations upon treatment?

Please see our response to the first point further above.

In Fig 2MN, how were the effects on proliferation, cell density in absolute terms during this period? How was protein content during this period or at the end?

We did not assess cell proliferation rates and cell density in absolute terms in this experiment but determined the whole protein amount of cell lysates from both the treatment conditions at the end of the experiment. The total Protein content was used to quantify GAG subspecies content as described in the materials and methods part. We added total protein amounts of control and MSD cell lines of either treatment condition into table EV1.

In addition, they have performed an RNAseq experiment in which they compared an 'inactive' retinoid with an 'active' retinoid. There is a conceptual problem with the interpretation of this experiment in this manuscript. The mechanism by which retinoids act is via activation of nuclear receptors. All these retinoids have different stabilities in vitro, they are oxygen and light sensitive and degrade within hours. That is likely the reason why natural retinoids have lower activity in the assays in this manuscript while the synthetic ones have higher activity as these likely have higher stability. This means that the differences that are now picked up by a 'positive' retinoid versus a 'negative' retinoid likely reflect the different stabilities of these retinoids in culture and hence the kinetics of gene expression and mRNA stability. The real players involved may therefore remain hidden when comparing 'active' and 'inactive' retinoid treatment, and may be present somewhere in the bulk of retinoid responsive genes when comparing retinoid treatment with mock treatment. The experiment is therefore a deviation from the main point, which is: how do retinoids increase FGE activity?

The experiment, suggested and requested in the first revision to our manuscript, was designed to identify mechanisms and targets upstream of the effect on FGE variant stabilisation resulting from tazarotene and bexarotene treatment. We would argue that the data from this experiment add

valuable content to the manuscript. We therefore kept the data in the final version of the manuscript but extended the discussion on the experimental approach and likely caveats of the results.
However, we appreciate the comment and fully agree that the influencing factors mentioned might well contribute to the displayed results. Nevertheless, we took cautions to adjust the comparability of the two retinoids used in this experiment by a set of preparatory experiments prior to the transcriptome analysis itself. The results are displayed and described in the appendix to this manuscript. We chose adapalene as it belongs to the same generation of pharmacological retinoid development like tazarotene (3rd generation). We assume, though we have to admit that we did not test it, that stabilities might be more similar compared to retinoids from earlier generations as mentioned in the comment above. Next, we approved adapalene's capability to increase transcription of known retinoid target genes in MSD fibroblasts via retinoic acid receptors. Although, we did not assess and compare concentrations of the drugs or metabolites in the cells, we agree that uptake, binding kinetics and metabolism in MSD cells could be different with impact on the transcriptional response of the two drugs. Taking this into account, we were aware that the analysis will not necessarily reveal true differences of action but rather differences that are only provoked by uptake and binding kinetics and effective concentrations in cells. However, we were able to identify rather clear differential gene expression for genes identifying the described pathway and were able, though very preliminary, to revert sulfatase activation by chemically interfering with the SREBP-SCAP-INSIG pathway. We therefore opine that this experiment has helped to identify an underlying potential pathway which could be the starting point to answer the question how to retinoids increase FGE activity.

Please see details modifications in the discussion part in the point-by-point responses to the editor's suggestions above.

The authors have added another experiment, in which they showed that retinoids increase the stability of FGE. This now provides a plausible explanation for the observed effects. It also makes sense that there is a requirement of residual FGE activity for the effects of retinoids. It hints to a rather simple mechanism in which players involved in turnover of less active/misfolded variants of FGE have a central role. This seems to be a key point in the possible mechanism and deserves follow up experiments to identify and characterise the retinoid-responsive players involved.

We agree with the referee's comment that tazarotene and bexarotene work via stabilization of variant FGE thereby increasing its residual functionality, which seems to be sufficient to activate sulfatases in MSD fibroblasts. We will aim for identifying interacting partners of misfolded FGE in follow-up studies. Likely, these interacting partners are members of ER quality control and/or the ER unfolded protein response mechanisms, whose interaction with FGE could be positively or negatively influenced due to tazarotene/bexarotene treatment. Ideally, mechanisms mediating FGE variant stabilization through tazarotene/bexarotene will be amenable to alternative activation by compounds different to retinoids.

In the discussion, I still think the possibility to treat MSD with retinoids is overstated and premature given the lack of in vivo experiments and the toxicity of retinoids in general.

We added a paragraph to the discussion part discussing the necessary steps necessary prior to clinical application that need to prove tazarotene's and bexarotene's potential in MSD in vivo. We hope that the new paragraph clarifies what needs to be done prior to any clinical application of tazarotene and bexarotene in MSD. Please see our detailed reply to the points raised by the editor, further above in this letter.

Referee #2 (Comments on Novelty/Model System for Author):

My criticism has been adequately addressed

We appreciate the referee's comment and are grateful for the helpful criticism.

Referee #3 (Comments on Novelty/Model System for Author):

This is a revised manuscript describing identification of possible treatments for a rare disorder, multiple sulfatase deficiencies (MSD), caused by variants in the SUMF1 gene encoding the formylglycine generating enzyme through a screen of clinically available drugs on an MSD patient derived cell line. The authors provide convincing evidence for beneficial effect of a subset of retinoids on MSD cell lines with hypomorphic SUMF1 mutations and focus tazarotene (identified in initial screen) and bexarotene
(identified in retinoid specific screen). They additionally identify potential specific molecular mechanisms downstream of the retinoic acid receptors by comparing RNAseq profiles of MSD primary fibroblasts lines treated with adapalene (an ineffective 3rd generation retinoid) and tazarotene. Pathway analysis of genes exclusively regulated by tazarotene treatment identified sterol and cholesterol biosynthesis pathways. Since the authors demonstrated that RXR and RAR antagonists HX 531 and AGN 193109 block the effect of tazarotene on ARSA activity, it would be interesting to know whether these antagonists block the effect of tazarotene on upregulation of sterol and cholesterol biosynthesis pathways, but is arguably beyond the scope of this manuscript.

We thank the referee for his positive comments and the suggestion of more experiments to elucidate the recent findings on the pathways upregulated upon tazarotene treatment. Such experiments are on a longer list of things to do to understand better the mode of action of tazarotene and bexarotene in MSD and will hopefully become part of additional publications in the future.

Referee #3 (Remarks for Author):

This is a very interesting study that lays the groundwork for further characterization of the mechanisms by which a subset of retinoids attenuate the effects of hypomorphic variants in the SUMF1 gene associated with MSD.

Again, we thank referee #3 for the comment. We can assure that we were excited about the upregulation of sterol and cholesterol biosynthesis pathways upon tazarotene and bexarotene treatment and will design and perform follow-up experiments to elucidate further details, including the use of RAR and RXR antagonists as suggested.

5th Oct 2022

Dear Dr. Schlotawa,

Thank you for the submission of your revised manuscript to EMBO Molecular Medicine. We have received the enclosed report from referee #1 who re-reviewed your manuscript. As you will see, this referee is now supportive of publication, and I am therefore pleased to inform you that we will be able to accept your manuscript once the following editorial points will be addressed:

1/ Main manuscript text:
- Your text was cross-checked for similarities with other manuscripts, and resemblances were found with other texts. Please modify your abstract accordingly (see the highlighted text in the attached document).
- Please remove the red text, and only keep in track changes mode any new modifications.
- Carefully check your text for spelling/grammatical errors (including The Paper Explained).
- Material and methods:
o Please indicate the origin of the cells.
o Please provide the antibodies concentrations.
- Data availability: Please remove the sentence "All data are available as source data linked to the main figures, expanded view figures or Appendix Fig. S1-S4." Please note that the data are currently private and must be released to the public before acceptance of the manuscript.
- Author contribution: CRediT has replaced the traditional author contributions section because it offers a systematic machine-readable author contributions format that allows for more effective research assessment. Please remove the Authors Contributions from the manuscript and use the free text boxes beneath each contributing author's name in our system to add specific details on the author's contribution. More information is available in our guide to authors.
- Please rename the "conflict of interest" section "Disclosure statement and competing interests": We updated our journal's competing interests policy in January 2022 and request authors to consider both actual and perceived competing interests. Please review the policy https://www.embopress.org/competing-interests and update your competing interests if necessary.

2/ Figures and Appendix:
- Several figures have a lot of panels. You might want to limit the number of figure panels and increase instead the number of figures to 7-8.
- As we do not have length limitation with the manuscript text, you may want to include (part of) the supplementary results currently in the Appendix to the main manuscript text.
- Thank you for providing exact p values in the appendix. Please link the appendix tables to the figures, and make sure exact p values are provided for all figures.
- The appendix table of content should include page numbers.
- In the Appendix, it would be good (though not a requirement) if the figure legends were underneath each figure.

3/ Checklist: please complete the "Sample definition and in-laboratory replication" section.

4/ Thank you for providing Source Data. Please zip them together so as to have 1 file per figure. Regarding Fig. EV2 source data, please annotate the blots.

5/ Every published paper now includes a 'Synopsis' to further enhance discoverability. Synopses are displayed on the journal webpage and are freely accessible to all readers. They include a short stand first (maximum of 300 characters, including space) as well as 2-5 one-sentences bullet points that summarizes the paper. Please write the bullet points to summarize the key NEW findings. They should be designed to be complementary to the abstract - i.e. not repeat the same text. We encourage inclusion of key acronyms and quantitative information (maximum of 30 words / bullet point). Please use the passive voice. Please attach these in a separate file or send them by email, we will incorporate them accordingly.

Please also suggest a striking image or visual abstract to illustrate your article as a PNG file 550 px wide x 300-600 px high.

6/ We note that you agree with the publication of the Review Process File.

I look forward to receiving your revised manuscript.

Yours sincerely,

Lise Roth

Lise Roth, PhD
Senior Editor
EMBO Molecular Medicine

***** Reviewer's comments *****

Referee #1 (Comments on Novelty/Model System for Author):

the authors have gone through great lengths to investigate new therapeutics for

MSD Referee #1 (Remarks for Author):

The authors have adequately addressed my comments.

The authors addressed the minor editorial issues.

9th Jan 2023

Dear Dr. Schlotawa,

Thank you for sending your revised synopsis text and image, and please accept my apologies for the delay in getting back to you during this busy time of the year.
I am pleased to inform you that your manuscript is accepted for publication and is now being sent to our publisher to be included in the next available issue of EMBO Molecular Medicine!

We would like to remind you that as part of the EMBO Publications transparent editorial process initiative, EMBO Molecular Medicine will publish a Review Process File online to accompany accepted manuscripts. If you do NOT want the file to be published or would like to exclude figures, please immediately inform the editorial office via e-mail.

Please read below for additional IMPORTANT information regarding your article, its publication and the production process.

Congratulations on your interesting work,

With kind regards,

Lise Roth

Lise Roth, Ph.D
Senior Editor
EMBO Molecular Medicine

Follow us on Twitter @EmboMolMed
Sign up for eTOCs at embopress.org/alertsfeeds

*** *** *** IMPORTANT INFORMATION *** *** ***

SPEED OF PUBLICATION
The journal aims for rapid publication of papers, using using the advance online publication "Early View" to expedite the process: A properly copy-edited and formatted version will be published as "Early View" after the proofs have been corrected. Please help the Editors and publisher avoid delays by providing e-mail address(es), telephone and fax numbers at which author(s) can be contacted.

Should you be planning a Press Release on your article, please get in contact with embomolmed@wiley.com as early as possible, in order to coordinate publication and release dates.

LICENSE AND PAYMENT:

All articles published in EMBO Molecular Medicine are fully open access: immediately and freely available to read, download and share.

EMBO Molecular Medicine charges an article processing charge (APC) to cover the publication costs. You, as the corresponding author for this manuscript, should have already received a quote with the article processing fee separately. Please let us know in case this quote has not been received.

Once your article is at Wiley for editorial production you will receive an email from Wiley's Author Services system, which will ask you to log in and will present you with the publication license form for completion. Within the same system the publication fee can be paid by credit card, an invoice, pro forma invoice or purchase order can be requested.

Payment of the publication charge and the signed Open Access Agreement form must be received before the article can be published online.

PROOFS

You will receive the proofs by e-mail approximately 2 weeks after all relevant files have been sent o our Production Office.

Please return them within 48 hours and if there should be any problems, please contact the production office at embopressproduction@wiley.com.

Please inform us if there is likely to be any difficulty in reaching you at the above address at that time. Failure to meet our deadlines may result in a delay of publication.

All further communications concerning your paper proofs should quote reference number EMM-2021-14837-V4 and be directed to the production office at embopressproduction@wiley.com.

EMBO Press Author Checklist

Corresponding Author Name: Lars Schlotawa	
Journal Submitted to: EMBO Molecular Medicine	
Manuscript Number: EMM-2021-14837	

USEFUL LINKS FOR COMPLETING THIS FORM
The EMBO Journal - Author Guidelines
EMBO Reports - Author Guidelines
Molecular Systems Biology - Author Guidelines
EMBO Molecular Medicine - Author Guidelines

Reporting Checklist for Life Science Articles (updated January 2022)
This checklist is adapted from Materials Design Analysis Reporting (MDAR) Checklist for Authors. MDAR establishes a minimum set of requirements in transparent reporting in the life sciences (see Statement of Task: 10.31222/osf.io/9sm4x). Please follow the journal's guidelines in preparing your manuscript.
Please note that a copy of this checklist will be published alongside your article.

Abridged guidelines for figures
1. Data
The data shown in figures should satisfy the following conditions:
- the data were obtained and processed according to the field's best practice and are presented to reflect the results of the experiments in an accurate and unbiased manner.
- ideally, figure panels should include only measurements that are directly comparable to each other and obtained with the same assay.
- plots include clearly labeled error bars for independent experiments and sample sizes. Unless justified, error bars should not be shown for technical replicates.
- if n<5, the individual data points from each experiment should be plotted. Any statistical test employed should be justified.
- Source Data should be included to report the data underlying figures according to the guidelines set out in the authorship guidelines on Data Presentation.

2. Captions
Each figure caption should contain the following information, for each panel where they are relevant:
- a specification of the experimental system investigated (eg cell line, species name).
- the assay(s) and method(s) used to carry out the reported observations and measurements.
- an explicit mention of the biological and chemical entity(ies) that are being measured.
- an explicit mention of the biological and chemical entity(ies) that are altered/varied/perturbed in a controlled manner.
- the exact sample size (n) for each experimental group/condition, given as a number, not a range;
- a description of the sample collection allowing the reader to understand whether the samples represent technical or biological replicates (including how many animals, litters, cultures, etc.).
- a statement of how many times the experiment shown was independently replicated in the laboratory.
- definitions of statistical methods and measures:
  - common tests, such as t-test (please specify whether paired vs. unpaired), simple χ2 tests, Wilcoxon and Mann-Whitney tests, can be unambiguously identified by name only, but more complex techniques should be described in the methods section;
  - are tests one-sided or two-sided?
  - are there adjustments for multiple comparisons?
  - exact statistical test results, e.g., P values = x but not P values < x;
  - definition of 'center values' as median or average;
  - definition of error bars as s.d. or s.e.m.

Please complete ALL of the questions below.
Select "Not Applicable" only when the requested information is not relevant for your study.

Materials

Newly Created Materials	Information included in the manuscript?	In which section is the information available? (Reagents and Tools Table, Materials and Methods, Figures, Data Availability Section)
New materials and reagents need to be available; do any restrictions apply?	Not Applicable	

Antibodies	Information included in the manuscript?	In which section is the information available? (Reagents and Tools Table, Materials and Methods, Figures, Data Availability Section)
For **antibodies** provide the following information:<br>- Commercial antibodies: RRID (if possible) or supplier name, catalogue number and or/clone number<br>- Non-commercial: RRID or citation	Yes	Materials and Methods

DNA and RNA sequences	Information included in the manuscript?	In which section is the information available? (Reagents and Tools Table, Materials and Methods, Figures, Data Availability Section)
**Short novel DNA or RNA including primers, probes**: provide the sequences.	Yes	Appendix supplementary tables

Cell materials	Information included in the manuscript?	In which section is the information available? (Reagents and Tools Table, Materials and Methods, Figures, Data Availability Section)
**Cell lines:** Provide species information, strain. Provide accession number in repository **OR** supplier name, catalog number, clone number, and/**OR** RRID.	Yes	Materials and Methods
**Primary cultures:** Provide species, strain, sex of origin, genetic modification status.	Not Applicable	
Report if the cell lines were recently **authenticated** (e.g., by STR profiling) and tested for mycoplasma contamination.	Yes	Materials and Methods, Appendix

Experimental animals	Information included in the manuscript?	In which section is the information available? (Reagents and Tools Table, Materials and Methods, Figures, Data Availability Section)
**Laboratory animals or Model organisms:** Provide species, strain, sex, age, genetic modification status. Provide accession number in repository **OR** supplier name, catalog number, clone number, **OR** RRID.	Not Applicable	
**Animal observed in or captured from the field:** Provide species, sex, and age where possible.	Not Applicable	
Please detail **housing and husbandry conditions**.	Not Applicable	

Plants and microbes	Information included in the manuscript?	In which section is the information available? (Reagents and Tools Table, Materials and Methods, Figures, Data Availability Section)
**Plants:** provide species and strain, ecotype and cultivar where relevant, unique accession number if available, and source (including location for collected wild specimens).	Not Applicable	
**Microbes:** provide species and strain, unique accession number if available, and source.	Not Applicable	

Human research participants	Information included in the manuscript?	In which section is the information available? (Reagents and Tools Table, Materials and Methods, Figures, Data Availability Section)
If collected and within the bounds of privacy constraints report on age, sex and gender or ethnicity for all study participants.	Not Applicable	

Core facilities	Information included in the manuscript?	In which section is the information available? (Reagents and Tools Table, Materials and Methods, Figures, Data Availability Section)
If your work benefited from core facilities, was their service mentioned in the acknowledgments section?	Not Applicable	

Design

Study protocol	Information included in the manuscript?	In which section is the information available? (Reagents and Tools Table, Materials and Methods, Figures, Data Availability Section)
If study protocol has been **pre-registered, provide DOI in the manuscript**. For clinical trials, provide the trial registration number **OR** cite DOI.	Not Applicable	
Report the **clinical trial registration number** (at ClinicalTrials.gov or equivalent), where applicable.	Not Applicable	

Laboratory protocol	Information included in the manuscript?	In which section is the information available? (Reagents and Tools Table, Materials and Methods, Figures, Data Availability Section)
Provide DOI OR other citation details if **external detailed step-by-step protocols** are available.	Not Applicable	

Experimental study design and statistics	Information included in the manuscript?	In which section is the information available? (Reagents and Tools Table, Materials and Methods, Figures, Data Availability Section)
Include a statement about **sample size** estimate even if no statistical methods were used.	Yes	Materials and Methods
Were any steps taken to minimize the effects of subjective bias when allocating animals/samples to treatment (e.g. **randomization procedure**)? If yes, have they been described?	Not Applicable	
Include a statement about **blinding** even if no blinding was done.	Yes	Materials and Methods
Describe **inclusion/exclusion criteria** if samples or animals were excluded from the analysis. Were the criteria pre-established?<br><br>If sample or data points were omitted from analysis, report if this was due to attrition or intentional exclusion and provide justification.	Not Applicable	
For every figure, are **statistical tests** justified as appropriate? Do the data meet the assumptions of the tests (e.g., normal distribution)? Describe any methods used to assess it. Is there an estimate of variation within each group of data? Is the variance similar between the groups that are being statistically compared?	Yes	Materials and Methods

Sample definition and in-laboratory replication	Information included in the manuscript?	In which section is the information available? (Reagents and Tools Table, Materials and Methods, Figures, Data Availability Section)
In the figure legends: state number of times the experiment was **replicated** in laboratory.	Yes	Materials and Methods, Figure Legends
In the figure legends: define whether data describe **technical or biological replicates**.	Yes	Materials and Methods, Figure Legends

Ethics

Ethics	Information included in the manuscript?	In which section is the information available? (Reagents and Tools Table, Materials and Methods, Figures, Data Availability Section)
Studies involving **human participants**: State details of **authority granting ethics approval** (IRB or equivalent committee(s), provide reference number for approval.	Not Applicable	
Studies involving **human participants**: Include a statement confirming that **informed consent** was obtained from all subjects and that the experiments conformed to the principles set out in the WMA Declaration of Helsinki and the Department of Health and Human Services Belmont Report.	Not Applicable	
Studies involving **human participants:** For publication of **patient photos**, include a statement confirming that consent to publish was obtained.	Not Applicable	
Studies involving experimental **animals**: State details of **authority granting ethics approva**l (IRB or equivalent committee(s), provide reference number for approval. Include a statement of compliance with ethical regulations.	Not Applicable	
Studies involving **specimen and field samples:** State if relevant **permits** obtained, provide details of authority approving study; if none were required, explain why.	Not Applicable	

Dual Use Research of Concern (DURC)	Information included in the manuscript?	In which section is the information available? (Reagents and Tools Table, Materials and Methods, Figures, Data Availability Section)
Could your study fall under dual use research restrictions? Please check biosecurity documents and list of **select agents and toxins** (CDC): https://www.selectagents.gov/sat/list.htm	Not Applicable	
If you used a select agent, is the security level of the lab appropriate and reported in the manuscript?	Not Applicable	
If a study is subject to dual use research of concern regulations, is the name of the **authority granting approval and reference number** for the regulatory approval provided in the manuscript?	Not Applicable	

Reporting

The MDAR framework recommends adoption of discipline-specific guidelines, established and endorsed through community initiatives. Journals have their own policy about requiring specific guidelines and recommendations to complement MDAR.

Adherence to community standards	Information included in the manuscript?	In which section is the information available? (Reagents and Tools Table, Materials and Methods, Figures, Data Availability Section)
State if relevant guidelines or checklists (e.g., **ICMJE, MIBBI, ARRIVE, PRISMA**) have been followed or provided.	Not Applicable	
For **tumor marker prognostic studies,** we recommend that you follow the **REMARK** reporting guidelines (see link list at top right). See author guidelines, under 'Reporting Guidelines'. Please confirm you have followed these guidelines.	Not Applicable	
For **phase II and III randomized controlled trials**, please refer to the **CONSORT** flow diagram (see link list at top right) and submit the CONSORT checklist (see link list at top right) with your submission. See author guidelines, under 'Reporting Guidelines'. Please confirm you have submitted this list.	Not Applicable	

Data Availability

Data availability	Information included in the manuscript?	In which section is the information available? (Reagents and Tools Table, Materials and Methods, Figures, Data Availability Section)
Have **primary datasets** been deposited according to the journal's guidelines (see 'Data Deposition' section) and the respective accession numbers provided in the Data Availability Section?	Yes	Source data have been submitted with the manuscript and its revision, RNAseq datasets have been submitted, acces number still pending, all details in the data availability section
Were **human clinical and genomic datasets** deposited in a public access-controlled repository in accordance to ethical obligations to the patients and to the applicable consent agreement?	Not Applicable	
Are **computational models** that are central and integral to a study available without restrictions in a machine-readable form? Were the relevant accession numbers or links provided?	Not Applicable	
If publicly available data were reused, provide the respective **data citations in the reference list**.	Not Applicable	
